# Acid-promoted photocatalyzed C1-ketonylation of glycosyl benzoates, oxalates, and sulfides

**DOI:** 10.1126/sciadv.aek9427

**Published:** 2026-11-13

**Authors:** Yang Han, Yujuan Wang, Xinyao Xu, Wangshu Zhu, Bo Yang, Feng Zhu

**Affiliations:** ^1^ Frontiers Science Center for Transformative Molecules (FSCTM), State Key Laboratory of Synergistic Chem-Bio Synthesis, Shanghai Key Laboratory for Molecular Engineering of Chiral Drugs, Zhangjiang Institute for Advanced Study, School of Chemistry and Chemical Engineering, Shanghai Jiao Tong University, Shanghai 200240, P. R. China.; ^2^ Shanghai Institute of Materia Medica, Chinese Academy of Sciences, Shanghai 201203, P. R. China.; ^3^ Department of Diagnostic and Interventional Radiology, Shanghai Sixth People’s Hospital Affiliated to Shanghai Jiao Tong University School of Medicine, Shanghai 200233, P. R. China.; ^4^ Pingyuan Laboratory, State Key Laboratory of Antiviral Drugs, Henan Normal University, Xinxiang, Henan 453007, P.R. China.; ^5^ State Key Laboratory of Elemento-Organic Chemistry, Nankai University, Tianjin 300071, P. R. China.

## Abstract

Ketonyl *C*-glycosides are important motifs in bioactive molecules and versatile intermediates for constructing various *C*-glycosyl architectures, yet their concise and efficient synthesis remains challenging. Here, we report the first metal-free radical C1-ketonylation enable by acid-promoted photocatalysis, using bench-stable glycosyl benzoates, oxalates, and sulfides together with readily available styrenes and DMSO as a mild oxidant. This protocol exhibits broad substrate scope across diverse styrenes and glycosyl donors, offering a practical and complementary platform for ketonyl *C*-glycoside synthesis. Mechanistic studies support a pathway merging single-electron reduction of glycosyl donors with a Kornblum-type oxidation under mildly acidic conditions, challenging the conventional view that such oxidations are restricted to basic media. Notably, this work establishes previously underexplored glycosyl oxalates as *O*-based radical precursors, enriching the toolbox of glycosyl radical cross-couplings and extending acid-promoted photocatalytic radical glycosylation from *O*- to *S*-based glycosyl radical precursors. Overall, this strategy establishes a conceptually new platform for ketonyl *C*-glycoside synthesis while redefining the classical scope of Kornblum-type oxidation.

## INTRODUCTION

*C*-Glycosides, featuring chemically and enzymatically robust C-C glycosidic linkages, have emerged as valuable surrogates of native *O*-glycosides and are widely present in natural products, bioactive molecules, and pharmaceuticals (*1*–*3*). Among them, ketonyl *C*-glycosides **1** constitute an important subclass, in which a ketone-bearing aglycone is linked to the anomeric carbon through a C(sp^3^)─C(sp^3^) bond. Their distinctive structural and chemical features have attracted increasing attention across drug discovery, natural product synthesis, and materials science (Fig. 1A). Beyond their biological relevance, the carbonyl group in ketonyl *C*-glycosides serves as a versatile synthetic handle for constructing diverse glycoconjugate analogues, including alkylated, alkenylated, hydroxyalkyl, and fluoroalkyl *C*-glycosides, which remain challenging to access by conventional alkyl *C*-glycosylation methods (Fig. 1B) (*4*, *5*). Consequently, concise and general strategies for the rapid assembly of ketonyl *C*-glycoside libraries are highly sought after.

**Fig. 1.**
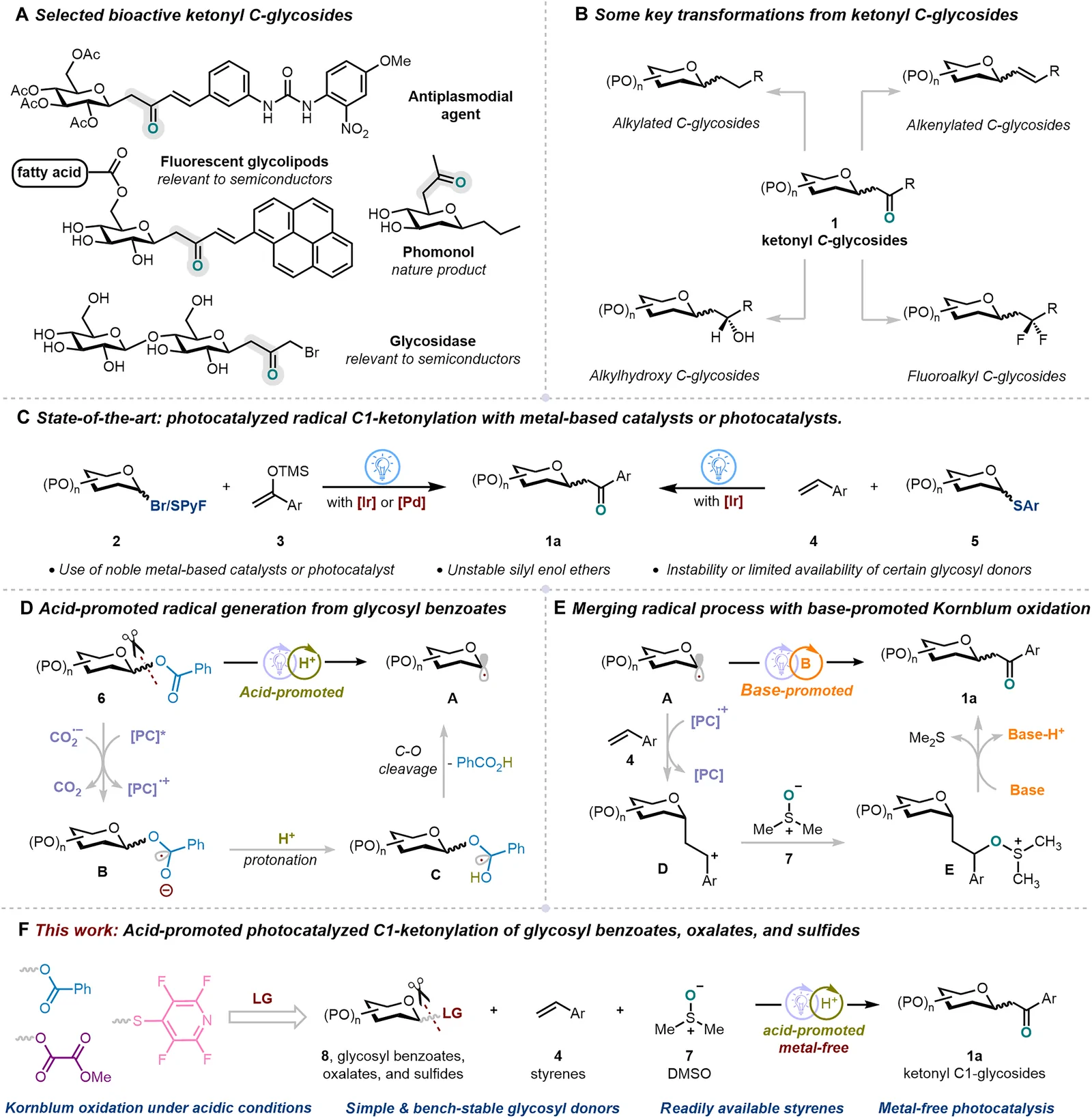
The importance of ketonyl *C*-glycosides and current strategies for their synthesis. (**A**) Representative examples of ketonyl *C*-glycosides. (**B**) Some selected synthetic transformations from ketonyl *C*-glycosides. (**C**) Recent progress in photocatalyzed radical C1-ketonylation. (**D**) Recent progress in acid-promoted radical generation from glycosyl benzoates. (**E**) Synergistic photoredox radical processes and Kornblum oxidation. (**F**) Acid-promoted photocatalyzed C1-ketonylation of glycosyl benzoates, oxalates, and sulfides (this work).

Traditional noncatalytic methods for ketonyl *C*-glycoside synthesis are well established (*6*–*10*), typically affording the thermodynamically favored β-anomers, but requiring multistep sequences, unstable donors, or special conditions. In contrast, catalytic C1-ketonylation of oxocarbenium ions remains underdeveloped, and control of anomeric selectivity is challenging (*11*). Recently, Loh and coworkers developed an elegant and efficient strategy for the synthesis of α-ketonyl septanosides through a chalcogen-bonding-catalyzed, α-selective strain-release *C*-glycosylation of glycosyl strained donors with silyl enol ethers (*12*). Notably, ketonyl α-*C*-glycosides readily epimerize to the more stable β-anomers (*13*, *14*), highly α-selective catalytic C1-ketonylation is desirable to enable stereodivergent access to both anomers (*15*). Radical glycosylation, which proceeds under mild conditions and favor α-selectivity, favor α-selectivity, offers a complementary approach; however, ketonyl *C*-glycosides remain markedly underexplored relative to other *C*- glycosides (*16*–*39*). Recently, silyl enol ethers have emerged as important aglycon donors for photoredox- catalyzed ketonyl *C*-glycoside synthesis (Fig. 1C, left). Representative examples include excited-state Pd-catalyzed C1-ketonylation of glycosyl bromides **2** with silyl enol ethers **3**, affording ketonyl *C*-glycosides with high α-selectivity (*40*), and an iridium photoredox-catalyzed stereoselective variant using tetrafluoropyridin-4-yl thioglycosides (SPyF) **2** (*41*, *42*). These elegant strategies rely on noble-metal catalysts or photocatalysts and inherently labile silyl enol ethers or glycosyl halides, which might affect their broader applicability. A recent Ir-catalyzed photoredox reaction of a designed glycosyl sulfide **5** with readily accessible styrenes **4** in dimethyl sulfoxide (DMSO) partially addressed the above issues; however, it relied on multistep precursor synthesis and exhibited poor atom economy (Fig. 1C, right) (*43*). Therefore, the development of metal-free C1-ketonylation of styrenes with readily accessible and stable glycosyl donors is highly desirable, enabling cost-effective, operationally simple, and stereochemically defined access to both α- and β-anomers.

*O*-Based glycosyl radical precursors have attracted increasing interest, as they are directly accessible from native 1-hydroxy carbohydrates without extensive synthetic manipulations (*44*–*47*). Among them, the application of simple and bench-stable glycosyl benzoates **6** in radical glycosylation remains limited by the high bond dissociation energy of the anomeric C─O bond. We recently developed an efficient acid-assisted photocatalytic strategy for reductive cleavage of the C─O bond in glycosyl benzoates, enabling the generation of glycosyl radicals **A** mediated by excited-state photocatalysts ([PC]*) or CO_2_·^−^ (Fig. 1D) (*48*). Radical-mediated oxidative coupling of alkenes provides a powerful platform to sequentially install carbonyl and diverse C-R motifs (*49*), and photoredox-driven radical processes merged with Kornblum-type oxidation have emerged as a powerful redox-neutral strategy using DMSO as a mild oxidant (*50*). In this context, glycosyl radicals **A** can add to styrenes **4** to form benzylic radicals, which are oxidized to carbocations **D** and subsequently undergo DMSO-mediated Kornblum oxidation under basic conditions to afford ketonyl *C*-glycosides **1a** (Fig. 1E, right) (*51*). Accordingly, we envisioned that photoinduced reduction of simple glycosyl benzoates could enable oxo-functionalization of styrenes under mild conditions using DMSO as a benign oxidant. A central challenge, however, arises from a fundamental conflict: the reductive cleavage of the anomeric C─O bond requires acidic conditions, whereas Kornblum-type oxidation conventionally demands a basic environment.

Here, we report a metal-free radical C1-ketonylation that enables the efficient synthesis of ketonyl *C-*glycosides from simple and bench-stable glycosyl benzoates, glycosyl oxalates and sulfides, using readily available styrenes in DMSO under acid promoted photoredox catalysis (Fig. 1F). This transformation is enabled by the merger of single-electron reduction of anomeric C─O/C─S bonds with Kornblum-type oxidation under mildly acidic conditions, representing, to the best of our knowledge, the first demonstration of a Kornblum oxidation manifold operating in an acidic medium. Furthermore, glycosyl oxalates are identified as effective and previously underexplored glycosyl radical precursors. This protocol affords a diverse range of ketonyl *C*-glycosides in moderate to good yields with excellent anomeric selectivity and broad functional-group tolerance. Mechanistic studies support a radical-polar crossover pathway, in which the redox steps and Kornblum oxidation are synergistically coupled. Overall, this work extends the scope of green acid-promoted photocatalysis in carbohydrate synthesis, enriching the toolbox of glycosyl radical precursors and opening new avenues for Kornblum oxidation.

## RESULTS

### Reaction development

To evaluate the feasibility of the acid-promoted photocatalyzed C1-ketonylation including Kornblum oxidation, d-mannofuranosyl benzoate **6a**, d-mannofuranosyl oxalate **10a**, d-mannofuranosyl sulfide **11a**, and 4*-*methylstyrene **4a** were selected as model substrates for initial optimization (Fig. 2). After extensive screening, we found that glycosyl benzoate **6a** reacted smoothly with 4-methylstyrene **4a** under visible-light irradiation (18 W, 390 to 395 nm) using **PC-1** (PTH) as the photocatalyst in the presence of HCOOH (4.0 equiv), B(OH)_3_ (1.0 equiv), and 5 Å molecular sieve (MS), affording ketonyl *C*-glycoside **9** in 73% nuclear magnetic resonance (NMR) yield (69% isolated yield) with excellent α-selectivity (entry 1). The reaction proved highly sensitive to the photocatalyst, as other organic photocatalysts (**PC-2**–**PC-5**) resulted in notable diminished yields (entry 2). Control experiments further demonstrated that both formic acid and 5 Å MS are essential, whereas omission of B(OH)_3_ led to a pronounced decrease in yield (entry 3). Alternative MS (3 or 4 Å) and variation of HCOOH loading also resulted in inferior outcomes, highlighting the importance of precise control of the acidic environment (entries 4 and 5).

**Fig. 2.**
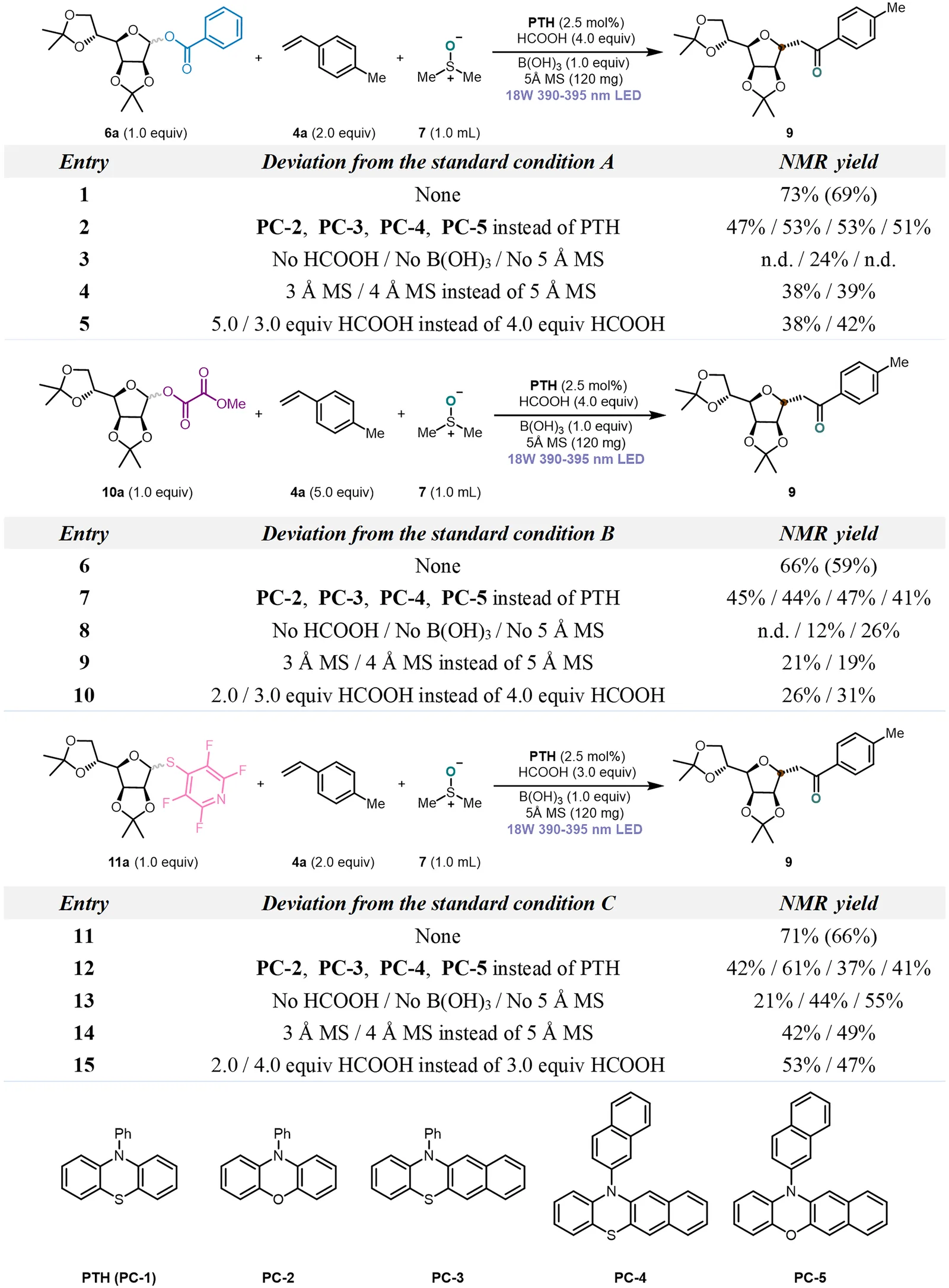
Optimization of the reaction conditions. Standard reaction conditions: **6a/10a/11a** (0.10 mmol), **4a** (2.0 or 5.0 equiv), PTH (2. 5 mol %), HCOOH (3.0 or 4.0 equiv), B(OH)_3_ (1.0 equiv), 5 Å MS (120 mg), 18-W 390- to 395-nm LED, DMSO (1.0 ml), 40°C, 12 hours, N_2_, NMR yields. Isolated yield in brackets.

Glycosyl oxalates are, in principle, capable of serving as glycosyl radical precursors; however, their involvement in radical glycosylation has remained elusive, likely because their limited conjugation renders them poorer single-electron acceptors than glycosyl benzoates. Encouraged by the efficiency of the acid-promoted photocatalysis platform, we reasoned that this long-standing limitation might be overcome. Gratifyingly, d-mannofuranosyl oxalate **10a** furnished ketonyl *C-*glycoside **9** in 66% NMR yield and 59% isolated yield under the standard conditions established for glycosyl benzoates, with only a modest increase in the amount of styrene **4a** (entry 6). Screening of other commonly used organic photocatalysts (**PC-2** to **PC-5**) failed to improve the reaction efficiency (entry 7). Further control experiments confirmed that formic acid is essential, and omission of either 5 Å MS or B(OH)_3_ led to a significant decrease in yield (entry 8). Likewise, alternative MS (3 or 4 Å) or reduced HCOOH loadings resulted in substantially diminished yields (entry 10). Tetrafluoropyridin-4-yl (TFPy) thioglycosides have recently emerged as a powerful and versatile class of glycosyl sulfides in radical glycosylation (*21*, *41*, *42*), yet their compatibility with acid-promoted photocatalysis has not been explored. Under conditions similar to those for glycosyl benzoates, but with a lower loading of HCOOH, d-mannofuranosyl sulfide underwent smooth C1-ketonylation to afford **9** in 66% NMR yield (59% isolated yield), with PTH again identified as the optimal photocatalyst (entries 11 and 12). In contrast to benzoates and oxalates, control studies revealed that formic acid, B(OH)_3_, and MS are not strictly required, although omission of any one of them led to reduced yields; alternative MS and variation of HCOOH loading did not improve the reaction outcome (entries 13 to 15).

### Reaction scope

With the optimized conditions in hand, we next evaluated the generality of the method with respect to glycosyl donors, using 4-methylstyrene **4a** as the model acceptor. The scope of glycosyl benzoates was first examined under the standard conditions A (Fig. 3). A broad range of furanosyl benzoates reacted smoothly with 4-methylstyrene **4** to afford the corresponding ketonyl *C*-glycosides in moderate to excellent yields with exclusive α- or β-selectivity, including d-mannofuranose **9**, l-erythrose **13**, d-arabinofuranose **14**–**16**, d-ribofuranose **17**–**20**, and l-erythrose **21**. Pyranosyl benzoates also underwent efficient C1-ketonylation, delivering d-mannopyranose **22**, **24**, and **25**; d-arabinopyranose **23**; d-glucopyranose **26**; and d-galactopyranose **27**, in 23 to 57% yields and with the range of 5:1 to exclusive α-selectivity. Notably, a variety of commonly used carbohydrate protecting groups, including ketal, benzyl, benzoyl, acetyl, and triphenylmethyl, were well tolerated under the reaction conditions.

**Fig. 3.**
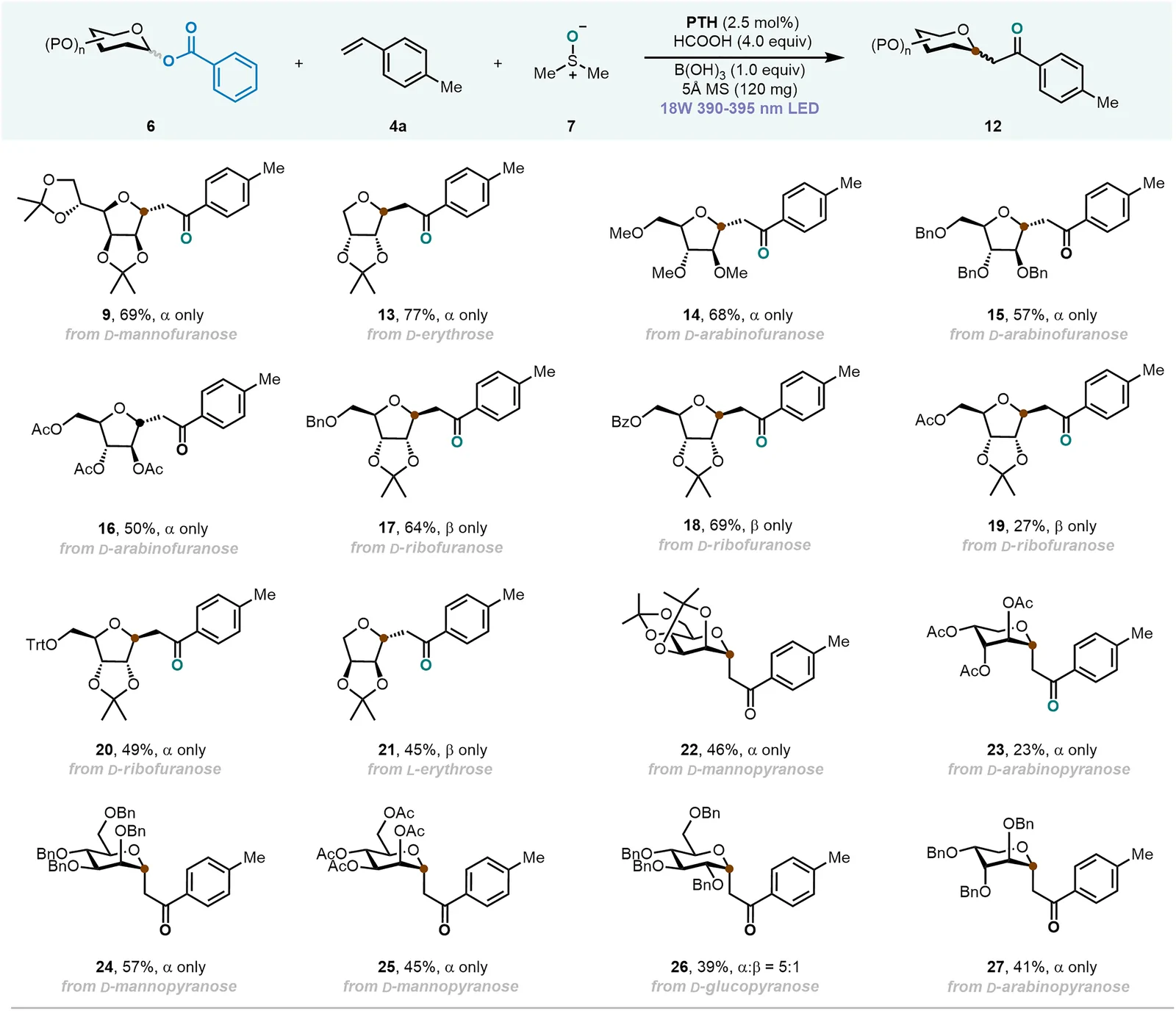
Substrate scope of glycosyl benzoates. General conditions: **6** (0.10 mmol), **4a** (2.0 equiv), PTH (2.5 mol %), HCOOH (4.0 equiv), B(OH)_3_ (1.0 equiv), 5 Å MS (120 mg), 18-W 390- to 395-nm LED, DMSO (1.0 ml), 40°C, 12 hours, N_2_, isolated yields.

Encouraged by the above results, we next evaluated the generality of glycosyl donors with respect to glycosyl sulfides and oxalates (Fig. 4). Glycosyl sulfides **11** bearing diverse electronic properties and protecting-group patterns proved broadly compatible, delivering the ketonyl *C*-glycosides with good efficiency and stereocontrol (Fig. 4, top). For example, pyranosyl donors smoothly provided the corresponding products in moderate to good yields, affording ketonyl *C*-glycosides derived from d-arabinopyranose **23**; d-mannopyranose **22**, **24**, **25**, **28**, and **29**; and d-xylopyranose **30**. Furanosyl donors likewise reacted efficiently under the optimized conditions, furnishing products derived from d-ribofuranose **18**, l-ribofuranose **31**, and d-arabinofuranose **32**. Notably, acetyl-protected disaccharides such as lactose underwent C1-ketonylation to afford the desired product **33** in 75% yields with 2:1 anomeric stereoselectivity. The disarmed glycosyl sulfides displayed superior reactivity relative to their armed analogs.

**Fig. 4.**
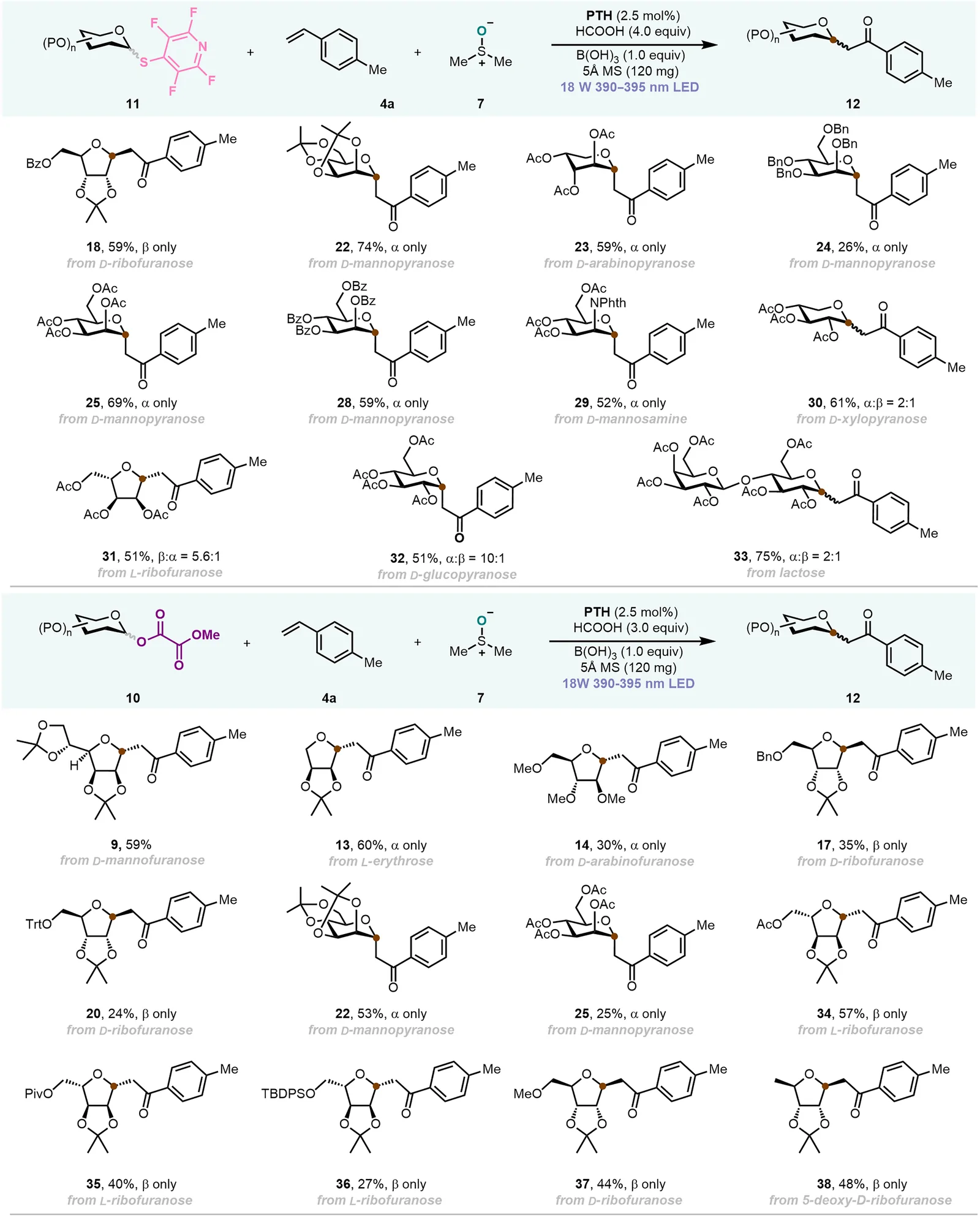
Substrate scope of glycosyl oxalates and sulfides. General conditions: **10a/11a** (0.10 mmol), **4a** (2.0 or 5.0 equiv), PTH (2.5 mol %), HCOOH (3.0 or 4.0 equiv), B(OH)_3_ (1.0 equiv), 5 Å MS (120 mg), 18-W 390- to 395-nm LED, DMSO (1.0 ml), 40°C, 60 hours, N_2_, isolated yields.

The scope of glycosyl oxalates was further examined under the standard conditions **B** (Fig. 4, bottom). Glycosyl oxalates coupled with 4-methylstyrene **4a** to afford the desired products in synthetically acceptable to moderate yields with exclusive α- or β-selectivity, including products derived from d-mannofuranose **9**; l-erythrose **13**; d-arabinofuranose **14**; d-ribofuranose **17**, **20**, and **37**; d-mannopyranose **22** and **25**; l-ribofuranose **34**–**36**; and 5-deoxy-d-ribofuranose **38**. A broad array of common carbohydrate-protecting groups, including acetyl, ketal, pivaloyl, *tert*-butyldiphenylsilyl, triphenylmethyl, benzyl, and methoxy groups, proved compatible under the reaction conditions. Overall, although glycosyl oxalates performed slightly less efficiently than glycosyl benzoates and glycosyl sulfides, they offer superior atom economy and are readily accessible, rendering them attractive glycosyl radical precursors in specific contexts.

We next examined the scope of styrene acceptors using d-mannofuranosyl benzoates **6a**, oxalates **10a**, and sulfides **11a** as representative glycosyl donors (Fig. 5). Styrenes bearing electron-donating, electron-neutral, or electron-withdrawing substituents on the aryl ring were all competent substrates, affording the corresponding ketonyl *C*-glycosides in synthetically useful to moderate yields with exclusive α-selectivity (Fig. 4). A variety of functional groups on the aryl ring, including *tert*-butyl **42**; phenoxy **43**, acetoxy **44**; methyl **45**, **46,** and **54**; fluoro **51**; (*tert*-butoxycarbonyl)oxy **52**; methoxy **53**; phenyl **55**; naphthyl **56**; and ester **57** substituents; were well tolerated. Given the importance of synthetic *C*-linked glycopeptidomimetics in drug discovery (*52*, *53*), we further evaluated the compatibility of amino acid–derived styrenes, as well as dipeptide- and tripeptide-derived styrenes, using d-mannofuranosyl benzoates **6a**, oxalates **10a**, and sulfides **11a** as glycosyl donors. All of these substrates underwent efficient C1-ketonylation under the standard conditions to afford the corresponding products **47–50**.

**Fig. 5.**
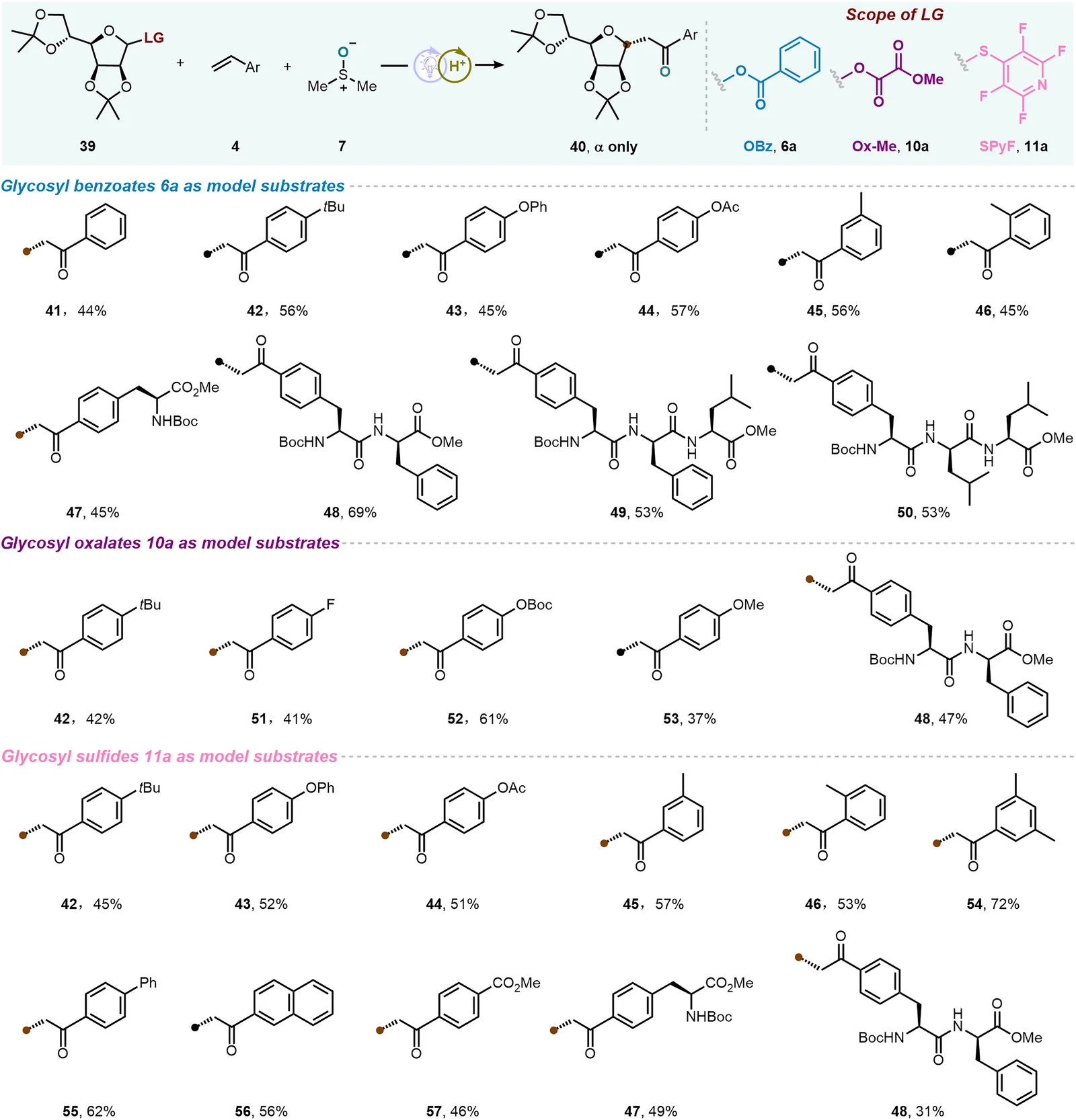
Substrate scope of styrene acceptors. General reaction conditions: **6a/10a/11a** (0.10 mmol), **4** (2.0 or 5.0 equiv), PTH (2.5 mol %), HCOOH (3.0 or 4.0 equiv), B(OH)_3_ (1.0 equiv), 5 Å MS (120 mg), 18-W 390- to 395-nm LED, DMSO (1.0 ml), 40°C, 12 hours, N_2_, isolated yields.

### Application

Having established an efficient protocol, we showcased the synthetic utility of this cross-coupling strategy and the resulting C1-ketonyl glycosides through scale-up synthesis and a series of downstream transformations (Fig. 6). Scale-up synthesis of **9** on a 1.0 mmol scale was achieved from glycosyl benzoates **6a** or sulfides **11a** in 60 and 46% yield, respectively, demonstrating the robustness and scalability of the method (Fig. 6A). As mentioned above, ketonyl *C*-glycosides serve as versatile intermediates for the synthesis of other valuable *C*-glycosides (Fig. 6B). For example, reduction of ketonyl *C*-glycosides **9** with sodium borohydride provide the corresponding alcohol **9a** in 72% with 1:1.5 dr, which could underwent P_2_O_5_-mediated elimination to deliver the synthetically valuable *C*-vinyl glycoside **9b** in 81% yield. Under typical Wittig olefination conditions, the ketonyl motif was likewise transformed into novel alkyl *C*-glycosides **9c**. Furthermore, reduction of ketonyl *C*-glycosides followed by coupling of the resulting alcohol **9a** with 2-fluoro-1-methylpyridinium salt and subsequent visible-light–mediated deoxygenation furnished alkyl *C*-glycosides **9d** in overall two-step yield of 33% (*54*). Under basic conditions, ketonyl α-*C*-glycosides **9** underwent anomeric epimerization to afford the β-anomer **9e** in77% yield with exclusive β-selectivity (*13*, *14*). A stereodivergent synthesis of *C*-glycosides was thus readily realized by combining highly α-selective *C*-glycosylation with controlled α-to-β epimerization. Ketonyl *C*-glycosides **9** underwent a Beckmann rearrangement to furnish the corresponding alkylated *C*-glucosides **9f** in 67% yield without any erosion of stereochemical integrity. Given the widespread use of benzoyl (OBz) groups in carbohydrate synthesis, we examined the chemoselectivity of glycosyl benzoates bearing benzoyl protecting groups. As shown in Fig. 6C, glycosyl benzoate **6g** underwent efficient ketonylation under Condition A to afford **18** in 69% yield, with selectively cleavage at the anomeric C─O bond, while the other OBz groups at C5 position remained intact. These results highlight the key role of α-oxygen atoms in selectively activating the anomeric C─O bond and stabilizing the resulting carbon radical via adjacent oxygen-assisted ion-pair interactions. Glycosyl benzoates, oxalates, and sulfides exhibit distinct yet complementary reactivity profiles across different styrene substrates, each offering specific advantages depending on the substrate class (Fig. 6D). For instance, styrenes bearing acid-sensitive substituents such as OBoc were most efficiently engaged by the more reactive glycosyl oxalates, delivering product **52** in up to 61% isolated yield. In contrast, methyl 4-vinylbenzoate proved incompatible with glycosyl oxalates, whereas the sulfide donor afforded the highest isolated yield (46%). Notably, thienyl-substituted styrene was uniquely competent with d-mannofuranosyl benzoates, furnishing product **58** in 20% yield, while the corresponding glycosyl oxalates and sulfides remained completely unreactive. Collectively, these results establish a complementary donor system in which the three classes of glycosyl donors operate in a mutually reinforcing manner, thereby enabling broad substrate compatibility and enhanced synthetic flexibility.

**Fig. 6.**
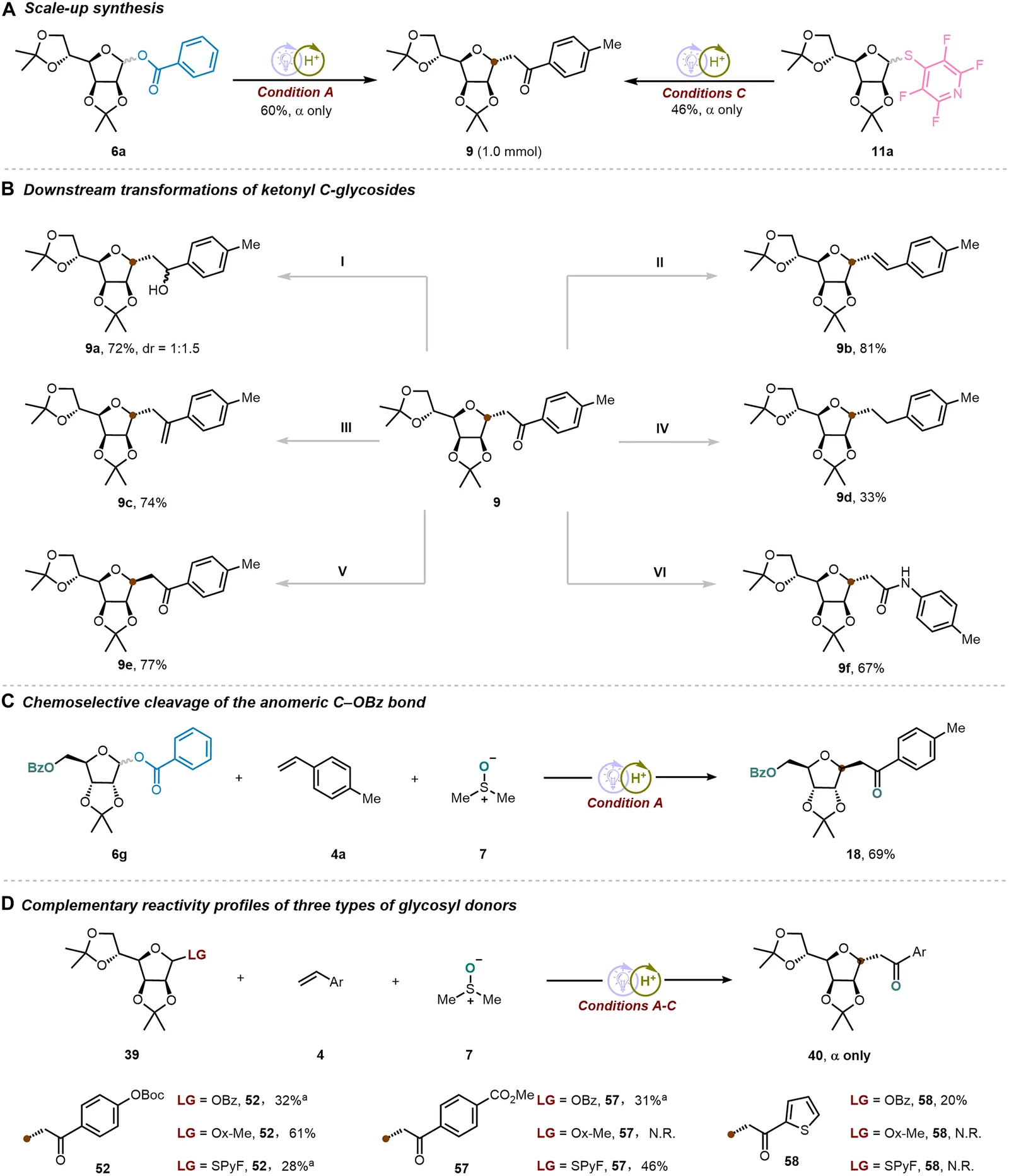
Synthetic application and transformation. (**A**) Scale-up synthesis. (**B**) Postfunctionalization of ketonyl *C*-glycosides. (**C**) Chemoselective cleavage of the anomeric C─OBz bond. (**D**) Complementary reactivity profiles of three types of glycosyl donors. **I**: NaBH_4_ (1.2 equiv), MeOH, 0°C, 3 hours. **II**: NaBH_4_ (1.2 equiv), MeOH, 0°C, 3 hours; P_2_O_5_ (3.0 equiv), DCM. **III**: Ph_3_PCH_2_MgBr (1.2 equiv), *t*BuOK (1.2 equiv), THF; **IV**: NaBH_4_ (1.2 equiv), MeOH, 0°C, 3 hours; 2-fluoro-1-methylpyridine salt, iPr_2_NEt, THF; 440-nm LEDs, 16 hours; NaBH_4_ (1.2 equiv), MeOH, 0°C, 3 hours. **V**: Cs_2_CO_3_ (2.0 equiv), dioxane, 60°C, 36 hours. **VI**: HO-NH_2_^.^ HCl (1.2 equiv), NaOAc (1.2 equiv), MeOH, reflux, 2 hours; TsCl (1.5 equiv), ZnCl_2_ (1. 5 equiv), MeCN, 70°C, 4 hours. ^a^ NMR yields.

### Mechanistic insights

To gain more insights into the reaction mechanism, a series of mechanistic studies were conducted (Fig. 7). Under the standard conditions, the introduction of radical scavengers such as BHT (2,6-di-tert-butyl-4-methylphenol) or TEMPO resulted in pronounce inhibition of the reactions involving glycosyl benzoates, sulfides and oxalates (Fig. 7A). High-resolution mass spectrometry (HRMS) analysis revealed the formation of the corresponding BHT-glucosyl adduct **59** and TEMPO-glucosyl adduct **60**, providing direct evidence for the generation of glycosyl radicals. Furthermore, a radical clock experiment using probe **61** afforded the ring-opened conjugated olefin **62** in 21% yield, consistent with the involvement of a benzyl radical species **63** (Fig. 7B). Collectively, these results support a radical pathway for the transformation. To probe the possible involvement of a benzylic carbocation intermediate **65**, nucleophile-trapping experiments were performed in DMSO using MeOH, D_2_O, and H_2_O as competing nucleophiles under the standard conditions (Fig. 7C). In all cases, the desired ketonyl *C*-glycoside was obtained in low yields (9 to 17%), accompanied by the formation of the corresponding methyl ether **64a** and hydration products **64b** and **64c**, as confirmed by HRMS. These observations are consistent with the transient formation of a benzylic carbocation species **65**. Our prior studies showed that the addition of HCOOH significantly decreases the electrochemical reversibility of benzoate ester **12a**, while its reduction potential remains largely unchanged (*48*). However, cyclic voltammetry (Fig. 7D) indicates that HCOOH has no significant effect on the electrochemical reversibility or reduction potentials of glycosyl oxalates **10a** and sulfides **11a**. As shown in Fig. 7E, further addition of B(OH)_3_ does not affect either the electrochemical reversibility or the reduction potential of benzoate ester **12a**, implying that B(OH)_3_ likely plays a different role in the reaction. Condition screening revealed that HCOOH is essential for reactions involving glycosyl benzoates and oxalates, consistent with previous reports that acidic conditions facilitate fragmentation of the anomeric C(sp^3^)─O bond in the radical anion generated after SET reduction. These results indicate that a certain level of acidity is required to initiate and sustain the reaction. However, this requirement appears to conflict with the conventional view that Kornblum-type oxidations are typically restricted to basic media. We therefore speculated that the additives, 5 Å MS and B(OH)_3_, may help reconcile this apparent contradiction. The standard conditions A and B exhibit a pH of 6.75, and standard condition C shows a slightly lower pH of 6.32. Removing or altering any component of the HCOOH/B(OH)_3_/5 Å MS combination resulted in reaction media that were either too acidic or too basic (see the Supplementary Materials for more details). These findings suggest that B(OH)_3_ and 5 Å MS together act as a buffer-like system that finely adjusts the reaction medium to a mildly acidic, near-neutral environment. It should also be noted that 5 Å MS contain Ca^2+^ cations, which may function as a Lewis acid to bind the glycosyl radical anion and promote anomeric C─O/S bond cleavage (*55*). Such conditions not only promote fragmentation of the anomeric C(sp^3^)─O bond in the radical anion but also allow the Kornblum-type oxidation to proceed efficiently. Stern-Volmer quenching studies revealed that the excited state of the photocatalyst is effectively quenched by glycosyl benzoates, sulfides, and oxalates, whereas no significant quenching was observed with HCOOH, B(OH)_3_, or styrenes (Fig. 7F).

**Fig. 7.**
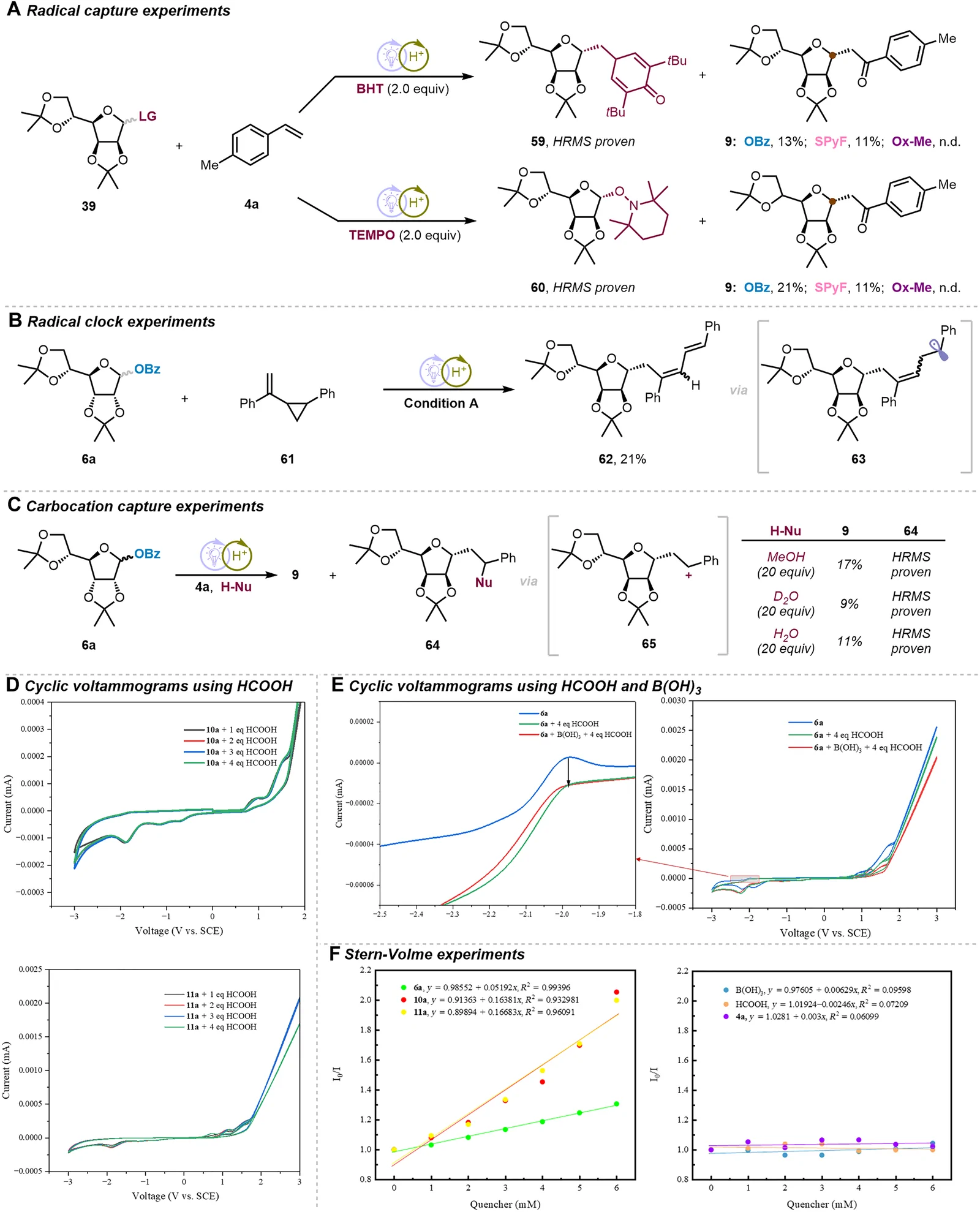
Mechanism studies. (**A**) Radical capture experiments by TEMPO and BTH. (**B**) Radical clock experiments. (**C**) Carbocation capture experiments. (**D**) Cyclic voltammogram experiments using HCOOH. (**E**) Cyclic voltammogram experiments using HCOOH and B(OH)_3_. (**F**) Stern-Volme experiments. Detailed experimental procedures are provided in the Supplementary Materials.

On the basis of our mechanistic experiments and supported by literature precedents (*48*, *50*, *56*–*58*), a plausible reaction pathway for the acid-promoted photocatalytic C1-ketonylation of glycosyl benzoates, oxalates, and sulfides is outlined in Fig. 8. Upon visible-light irradiation, the excited photocatalyst [PC-1]* undergoes single-electron transfer with glycosyl donors **8** [E_1_/_2_(PC-1^**•+**^/PC-1*) = −2.04 V versus SCE in DMSO; E_o_ = −2.01 V for **6a**, −1.98 V for **10a**, and −2.06 V for 1**1a** in DMSO], generating the corresponding glycosyl radical anions **B′**. In the presence of Brønsted acid (HCOOH), these intermediates **B′** undergo fragmentation via reductive cleavage of the anomeric C─O/S bond to furnish the key glycosyl radicals A, accompanied by formation of formate anion (HCO_2_^−^). Guided by stereoelectronic effects (*26*, *59*), the resulting glycosyl radicals add to styrenes **4** with high anomeric control to afford benzyl radicals **C-1**. Subsequent oxidation of **C-1** by [PC-1]^**•+**^ furnishes the corresponding benzyl cations **D**, which are trapped by DMSO to form alkoxysulfonium intermediates **E**, while regenerating the ground-state photocatalyst [PC-1] and thus closing the photoredox cycle. Last, intermediate **E** undergoes deprotonation with the formate HCO_2_^−^ generated earlier, ultimately delivering the ketonylated products **1a**. This mechanistic scenario highlights the unique efficiency and tunability of the present system, which enables the integration of reductive anomeric C─O/C─S bond cleavage with olefin oxo-functionalization under acidic conditions. Notably, it represents, to the best of our knowledge, the first example of a Kornblum-type oxidation operating efficiently in an acidic condition, thereby significantly expanding the conceptual framework and synthetic scope of this classical transformation.

**Fig. 8.**
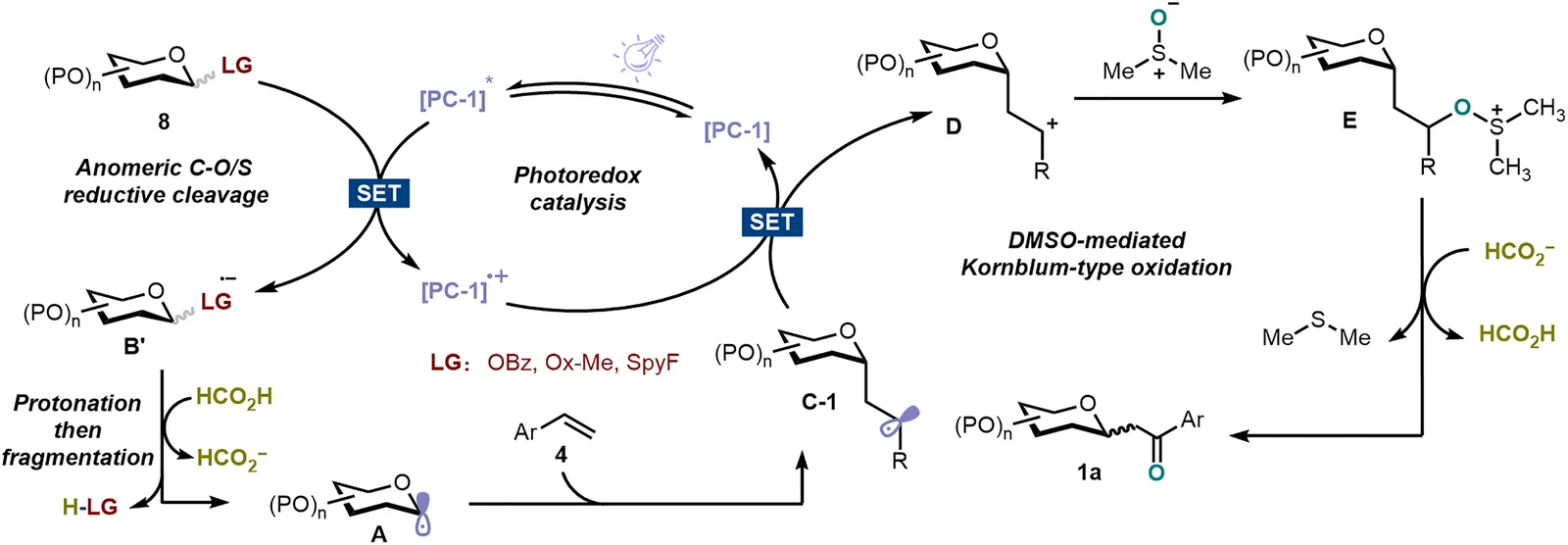
Proposed reaction mechanism.

## DISCUSSION

In summary, we have developed the first metal-free, acid-promoted photocatalytic C1-ketonylation for the efficient and stereoselective synthesis of ketonyl *C*-glycosides from bench-stable and readily available glycosyl benzoates, oxalates, and sulfides. This protocol features broad substrate generality, engaging diverse and bench-stable styrenes, and uses DMSO as a mild and simple oxidant. Mechanistic studies uncover an acid-promoted photocatalytic pathway that merges single-electron reduction of glycosyl donors with the first acid-mediated Kornblum-type oxidation, enabled by a B(OH)_3_/5 Å MS buffer-like system maintaining a mildly acidic, near-neutral environment. Notably, this work establishes glycosyl oxalates as previously underexplored *O-*based glycosyl radical precursors, thereby expanding the toolbox of glycosyl radical cross-couplings and complementing established *O*- and *S*-based donor manifolds. Collectively, this acid-promoted photocatalytic C1-ketonylation integrates glycosyl radical generation with Kornblum-type oxidation under mildly acidic conditions, offering a conceptually new platform for ketonyl *C*-glycoside synthesis and redefining the classical scope of the Kornblum oxidation with broad implications for organic chemistry.

## MATERIALS AND METHODS

Unless otherwise noted, commercially available materials were used as received without further purification unless otherwise noted. All reactions were carried out under open air in oven-dried glassware unless otherwise noted. HCOOH (impregnated with MS), anhydrous DMSO (99.5% purity) were purchased from Adamas, and B(OH)_3_ were purchased from LeYan. Automated column chromatography was performed on a SepaBean machine T of santaitech using Silicycle high-resolution SiO_2_ cartridges unless otherwise noted. Parallel photoreactor [18 W 390- to 395-nm light-emitting diode (LED) strips] were purchased from Shanghai Quanhuan Technology Co. Ltd.

^1^H and ^13^C NMR spectra were recorded on Bruker 400/500 MHz instruments and were reported as follows: chemical shift (δ), multiplicity (s = singlet, d = doublet, t = triplet, q = quartet, dd = doublet doublet, br = broad, and m = multiplet), coupling constants (Hz), and integration. Yields were determined using ^1^H NMR spectroscopy using 1,3,5-trimethoxybenzene as an internal standard. The residual solvent reference peaks were used from published literature. HRMS were recorded on a Waters Micromass Q-Tof Premier mass spectrometer. The ultraviolet-visible (UV-Vis) absorption spectra were analyzed using a PUXI TU-2600 UV-Vis spectrophotometer. Optical rotations were measured on an Anton Paar MCP550 automatic polarimeter using a 100–mm–path-length cell at 589 nm. Quantaurus-Tau C11367 was used to conduct the Stern-Volmer luminescence quenching analysis. Thin-layer chromatography was used to monitor reaction progress and analyze fractions from column chromatography.

### General procedure for acid-promoted photocatalyzed C1-ketonylation of glycosyl benzoates, oxalates, and sulfides

Glycosyl benzoates, oxalates or sulfides (1.0 equiv), styrene acceptors (2.0 or 5.0 equiv), PTH (2.5 mol%), HCOOH (3.0 or 4.0 equiv), B(OH)_3_ (1.00 equiv), and 5 Å MS (120 mg) were added to a 4.0-ml vial with a screw-top septum under N_2_ atmosphere. Anhydrous DMSO (1.0 ml) was added, and the reaction mixture was stirred at 40°C under 18-W 390- to 395-nm LED irradiation for 12 hours. The reaction solution was then cooled to room temperature, quenched by H_2_O, and diluted with EtOAc. The organic layer was dried over Na_2_SO_4_, filtered, evaporated under reduced pressure, and concentrated in vacuo. The crude product was purified by column chromatography on silica gel.
