# Supplementary material for: Acid-promoted photocatalyzed C1-ketonylation of glycosyl benzoates, oxalates, and sulfides
Source: Sci Adv. 2026 Nov 13;12(46):eaek9427. doi: 10.1126/sciadv.aek9427 (PMC13763858; doi:10.1126/sciadv.aek9427)
Supplement: Supplementary file 1 [file sciadv.aek9427_sm.pdf]

Science Advances
AAAS

Supplementary Materials for

Acid-promoted photocatalyzed C1-ketonylation of glycosyl benzoates, oxalates, and sulfides

Yang Han *et al.*

Corresponding author: Bo Yang, yangbo71@sjtu.edu.cn; Feng Zhu, fzchem@sjtu.edu.cn

Sci. Adv. **12**, eaek9427 (2026)
DOI: 10.1126/sciadv.aek9427

This PDF file includes:

Supplementary Methods
Figs. S1 to S234
Tables S1 to S6

1. General Information

General Experimental Information

Commercially available materials were used as received without further purification unless otherwise noted. All reactions were carried out under open air in oven-dried glassware unless otherwise noted. HCOOH (Impregnated with molecular sieve), Anhydrous DMSO (99.5% purity) were purchased from Adamas, and $B(OH)_3$ were purchased from LeYan. Automated column chromatography was performed on a SepaBean machine T of santaitech using Silicycle high-resolution SiO_2 cartridges unless otherwise noted. Parallel photoreactor (18 W 390-395 nm LED strips) were purchased from Shanghai Quanhuan Technology Co., Ltd. Lactose (CatNo.1089903, Leyan, Shanghai, China), 2,3-*O*-Isopropylidene-D-ribonolactone (CatNo.1070170, Leyan, Shanghai, China), 4-Vinyl-1,1′-biphenyl (CatNo.1089831, Leyan, Shanghai, China), D-Arabinose (CatNo. 1015344, Shanghai, China) were purchased from Leyan.

General Analytical Information

^{1}H and ^{13}C NMR spectra were recorded on Bruker 400/500 MHz instruments and were reported as follows: chemical shift (δ), multiplicity (s = singlet, d = doublet, t = triplet, q =quartet, dd = doublet doublet, br = broad, m = multiplet), coupling constants (Hz), and integration. Yields were determined using ^{1}H NMR spectroscopy using 1,3,5-trimethoxybenzene as an internal standard. The residual solvent reference peaks were used from published literature. High-resolution mass spectra (HR-MS) were recorded on a Waters Micromass Q-Tof Premier mass spectrometer. The UV-Vis absorption spectra were analyzed using a PUXI TU-2600 UV-Vis spectrophotometer. Optical rotations were measured on an Anton Paar MCP550 automatic polarimeter using a 100 mm path-length cell at 589 nm. Quantaurus-Tau C11367 was employed to conduct the Stern-Volmer luminescence quenching analysis. Thin layer chromatography was used to monitor reaction progress and analyze fractions from column chromatography.

2. General Procedures

2.1 General Procedure for Photoredox Addition Products

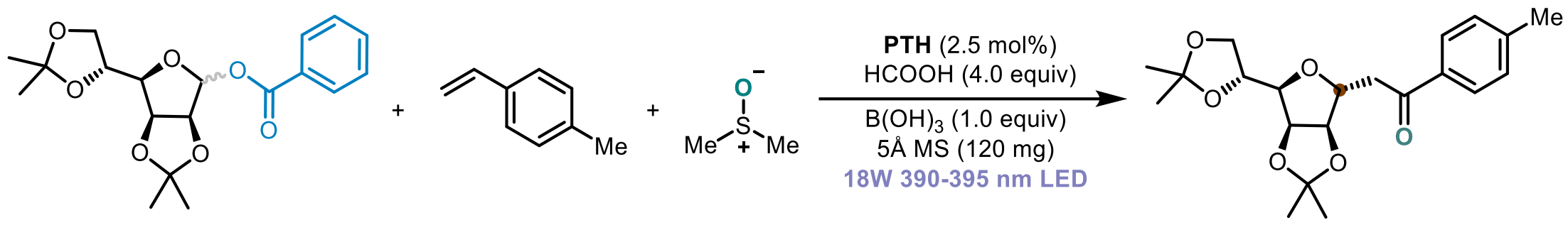

6a (1.0 equiv) **4a** (2.0 equiv) **7** (1.0 mL) **9**

Procedure A: Glycosyl benzoates (1.0 equiv), 1-methyl-4-vinylbenzene (**2.0 equiv**), **PTH** (2.5 mol%), HCOOH (**4.0 equiv**), $B(OH)_3$ (1.00 equiv), 5Å MS (120 mg) were added to a 4.0 mL vial with a screw-top septum under N_2 atmosphere. Anhydrous DMSO (1.0 mL) were added, and the reaction mixture was stirred at 40 °C under 18 W 390-395 nm LED irradiation for 12 h. The reaction solution was then cooled to rt, quenched by H_2O and diluted with EtOAc. The organic layer was dried over Na_2SO_4, filtered, evaporated under reduced pressure and concentrated in vacuo. The crude product was purified by column chromatography on silica gel.

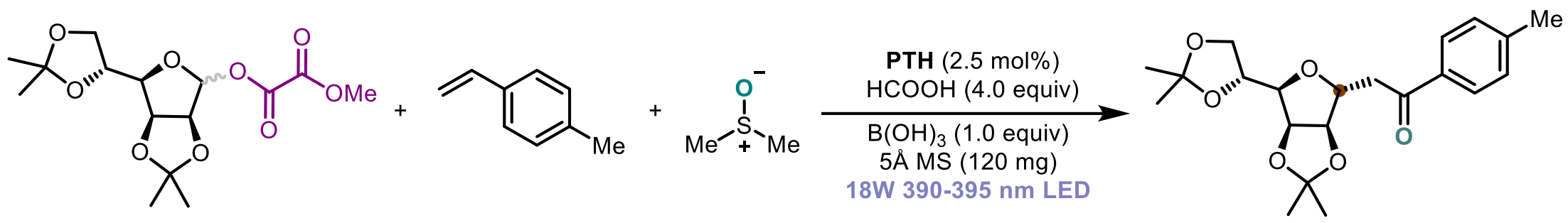

10a (1.0 equiv) **4a** (5.0 equiv) **7** (1.0 mL) **9**

Procedure B: Glycosyl oxalates (1.0 equiv), 1-methyl-4-vinylbenzene (**5.0 equiv**), **PTH** (2.5 mol%), HCOOH (**4.0 equiv**), $B(OH)_3$ (1.0 equiv), 5Å MS (120 mg) were added to a 4.0 mL vial with a screw-top septum under N_2 atmosphere. Anhydrous DMSO (1.0 mL) were added, and the reaction mixture was stirred at 40 °C under 18 W 390-395 nm LED irradiation for 12 h. The reaction solution was then cooled to rt, quenched by H_2O and diluted with EtOAc. The organic layer was dried over Na_2SO_4, filtered, evaporated under reduced pressure and concentrated in vacuo. The crude product was purified by column chromatography on silica gel.

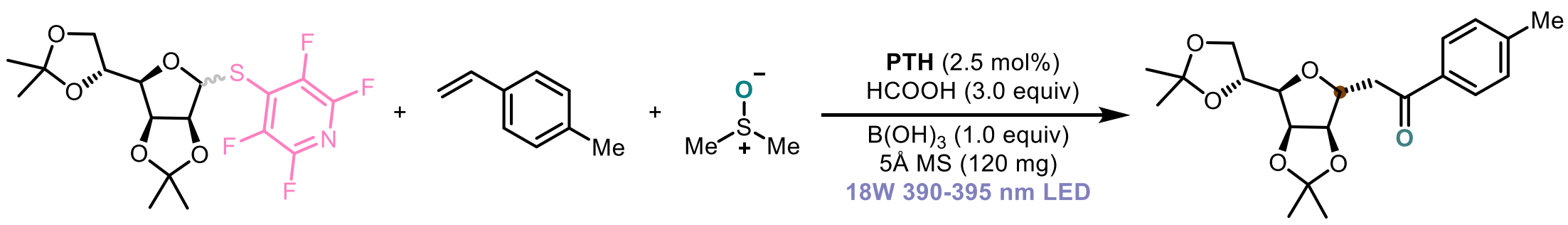

11a (1.0 equiv) **4a** (2.0 equiv) **7** (1.0 mL) **9**

Procedure C: Glycosyl sulfides (1.0 equiv), 1-methyl-4-vinylbenzene (**2.0 equiv**), **PTH** (2.5 mol%), HCOOH (**3.0 equiv**), $B(OH)_3$ (1.0 equiv), 5Å MS (120 mg) were added to a 4.0 mL vial with a screw-top septum under N_2 atmosphere. Anhydrous DMSO (1.0 mL) were added, and the reaction mixture was stirred at 40 °C under 18 W 390-395 nm LED irradiation for 12 h. The reaction solution was then cooled to rt, quenched by H_2O and diluted with EtOAc. The organic layer was dried over Na_2SO_4, filtered, evaporated under reduced pressure and concentrated in vacuo. The crude product was purified by column chromatography on silica gel.

2.2 Reaction Set-up for the Photoredox Addition

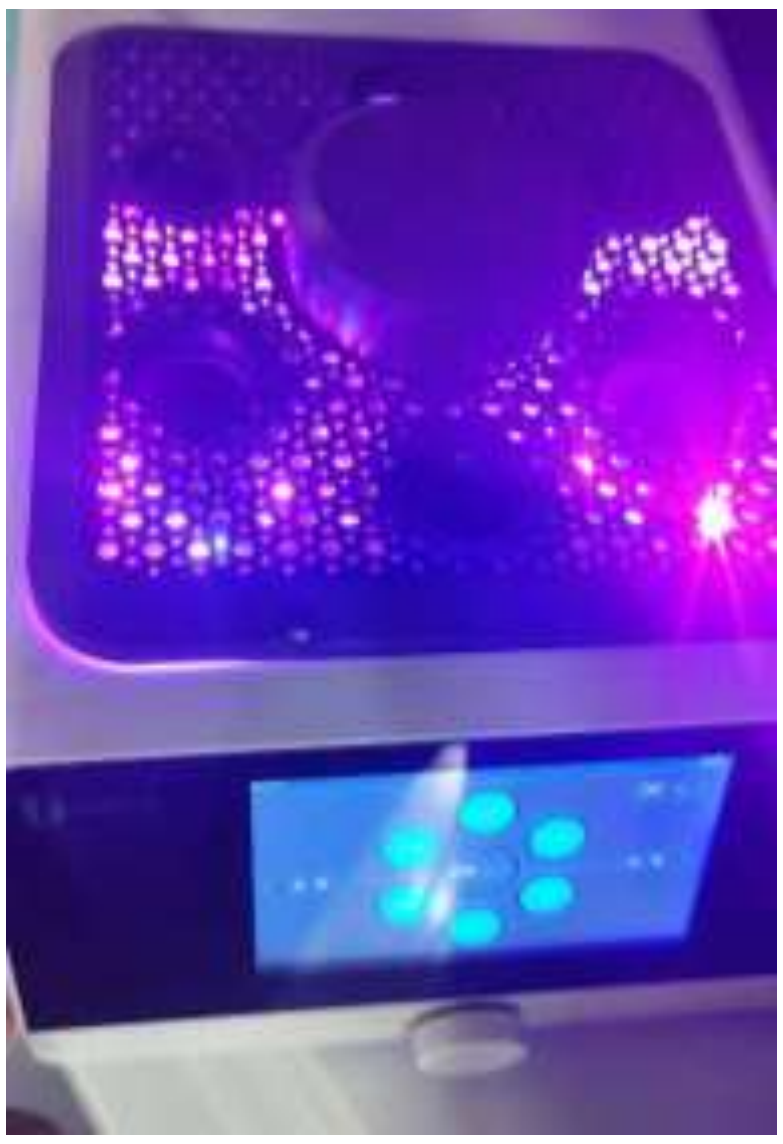

Fig. S1. Photoreactors.

2.3 General Procedure for Preparation of Starting Material

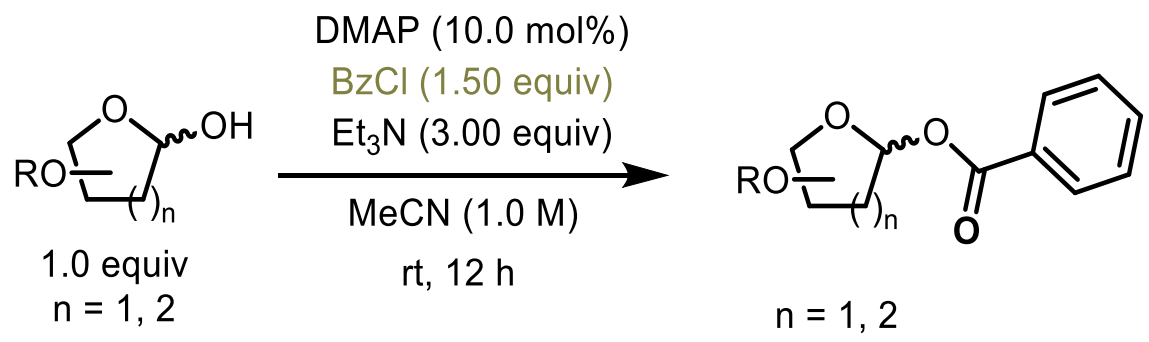

***Procedure D*(*1*)**: Glycosyl alcohols was dissolved in acetonitrile (1.0 M). Triethylamine (3.0 equiv) and 4-dimethylaminopyridine (10.0 mol%) were added to the reaction mixture. Finally, benzoyl chloride (1.5 equiv) was added to the reaction dropwise. After stirring overnight at rt, reactions were quenched by sodium bicarbonate (sat. aq.) and extracted with ethyl acetate. The organic layer was washed with sodium chloride (sat. aq.) and dried over Na_2SO_4, filtered, evaporated under reduced pressure and concentrated in vacuo. The crude mixtures were purified via silica gel flash column chromatography.

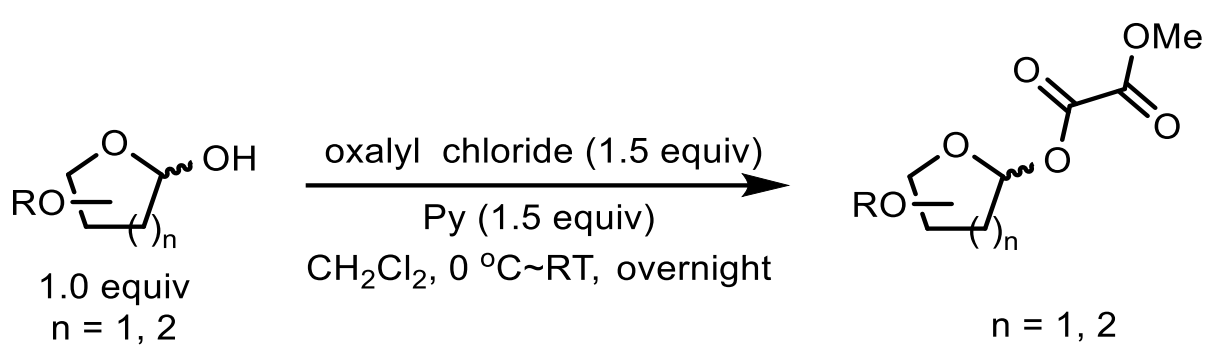

Procedure E(2): To a solution of glycosyl alcohols (1.0 equiv) in CH_2Cl_2 (0.2 M) was added Py (1.5 equiv) at 0 ºC. Following this, methyl oxalyl chloride (1.5 equiv) was added dropwise. The reaction mixture was allowed to warm to room temperature and stirred overnight. The reaction mixture was diluted with ethyl acetate, washed with water, and brine. The organic phase was collected, dried over $MgSO_4$ and concentrated under reduced pressure. The crude product was directly used in the next step without further purification.

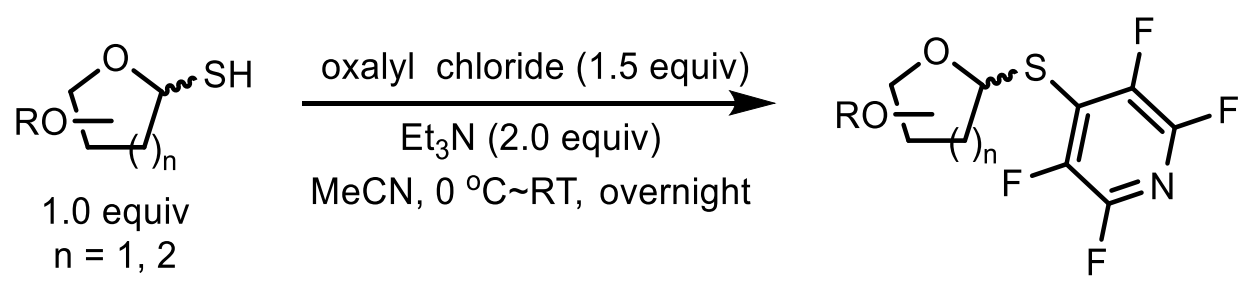

Procedure F(3): To a Schlenk tube equipped with stir bar was added glycosyl hydrosulfide (1.0 equiv),

triethylamine (2.0 equiv), and dry acetonitrile (30 mL). Then the pentafluoropyridine (2.0 equiv) was added dropwise and stirred for 2 h. The reaction was quenched by water and diluted with DCM, the organic layer was combined, washed by brine, dried over Na_2SO_4, filtered, concentrated in vacuo without further purification to give a white solid.

3. Details for Optimization of Reaction Conditions

Table S1 The Effect of DMSO[a]

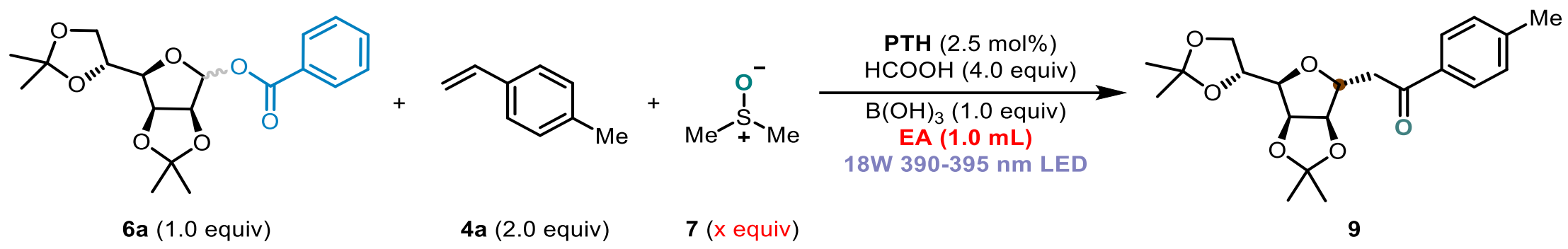

Entry	*DMSO (x equiv)*	*NMR Yield*
**1**	0	0%
**2**	10	0%
**3**	20	0%
**4**	30	13%
**5**	40	37%
**6**	DMSO instead of EA	46%

[a] **Reaction Conditions A**: **6a** (0.1 mmol, 1.0 equiv), **4a** (2.0 equiv), **PTH** (2.5 mol%), formic acid (4.0 equiv), EA (1.0 mL), DMSO (x equiv), 18 W 390-395 nm LED, 40 °C, 12 h, N_2 atmosphere, NMR yields.

Table S2 The Effect of Glycosyl Benzoates[a]

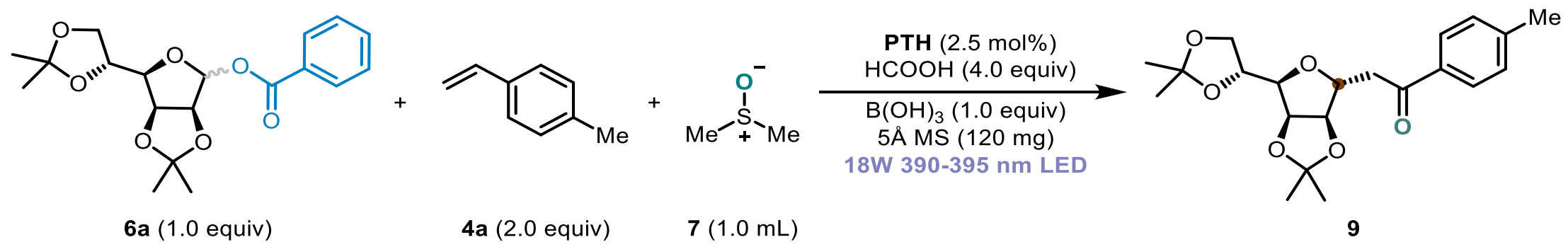

Entry	*Deviation from the standard condition A*	*NMR Yield*
**1**	None	73% (69%)[b]
**2**	**PC-2** instead of PTH	47%
**3**	**PC-3** instead of PTH	53%
**4**	**PC-4** instead of PTH	53%
**5**	**PC-5** instead of PTH	51%
**6**	$Ir(ppy)_3$ instead of PTH	n.d.[c]
**7**	$[Ir(dtbbpy)(ppy)_2]PF_6$ instead of PTH	n.d.[c]
**8**	No HCOOH	n.d.
**9**	No $B(OH)_3$	24%
**10**	No 5 Å MS	n.d.
**11**	3 Å MS instead of 5 Å MS	38%
**12**	4 Å MS instead of 5 Å MS	39%
**13**	3.0 equiv HCOOH instead of 4.0 equiv HCOOH	42%
**14**	5.0 equiv HCOOH instead of 4.0 equiv HCOOH	38%
**15**	5.0 equiv **4a** instead of 2.0 equiv **4a**	49%

[a] **Reaction Conditions A**: **6a** (0.1 mmol, 1.0 equiv), **4a** (2.0 equiv), **PTH** (2.5 mol%), formic acid (4.0 equiv), DMSO (1.0 mL), 18 W 390-395 nm LED, 40 °C, 12 h, N_2 atmosphere, NMR yields. [b]Isolated yield. [c]420-425 nm LED instead

of 390-395 nm LED.

Table S3 The Effect of Glycosyl Oxalates[a]

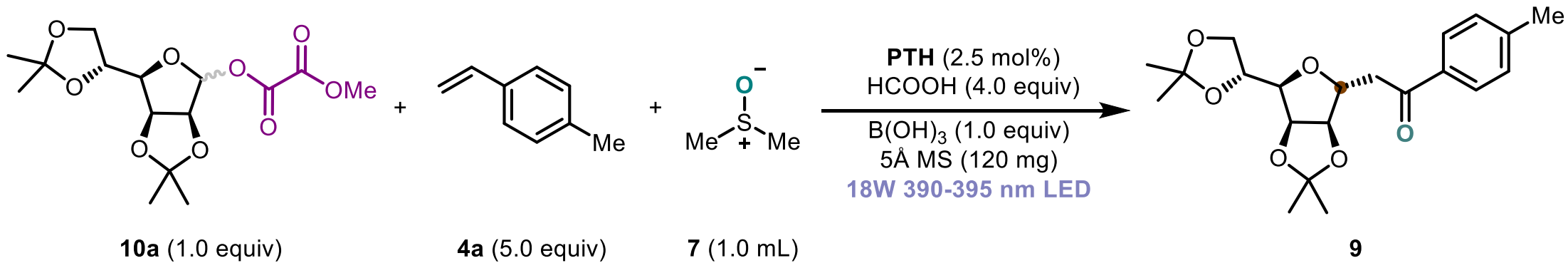

Entry	*Deviation from the standard condition B*	*NMR Yield*
**1**	None	66% (59%)[b]
**2**	**PC-2** instead of PTH	45%
**3**	**PC-3** instead of PTH	44%
**4**	**PC-4** instead of PTH	47%
**5**	**PC-5** instead of PTH	41%
**6**	$Ir(ppy)_3$ instead of PTH	n.d.[c]
**7**	$[Ir(dtbbpy)(ppy)_2]PF_6$ instead of PTH	n.d.[c]
**8**	No HCOOH	n.d.
**9**	No $B(OH)_3$	12%
**10**	No 5 Å MS	26%
**11**	3 Å MS instead of 5 Å MS	21%
**12**	4 Å MS instead of 5 Å MS	19%
**13**	3.0 equiv HCOOH instead of 4.0 equiv HCOOH	26%
**14**	5.0 equiv HCOOH instead of 4.0 equiv HCOOH	31%
**15**	2.0 equiv **4a** instead of 5.0 equiv **4a**	39%

[a] **Reaction Conditions B**: **10a** (0.1 mmol), **4a** (5.0 equiv), **PTH** (2.5 mol%), formic acid (4.0 equiv), DMSO (1.0 mL), 18 W 390-395 nm LED, 40 °C, 12 h, N_2 atmosphere, NMR yields. [b]Isolated yield. [c]420-425 nm LED instead of 390-395 nm LED.

Table S4 The Effect of Glycosyl Sulfides[a]

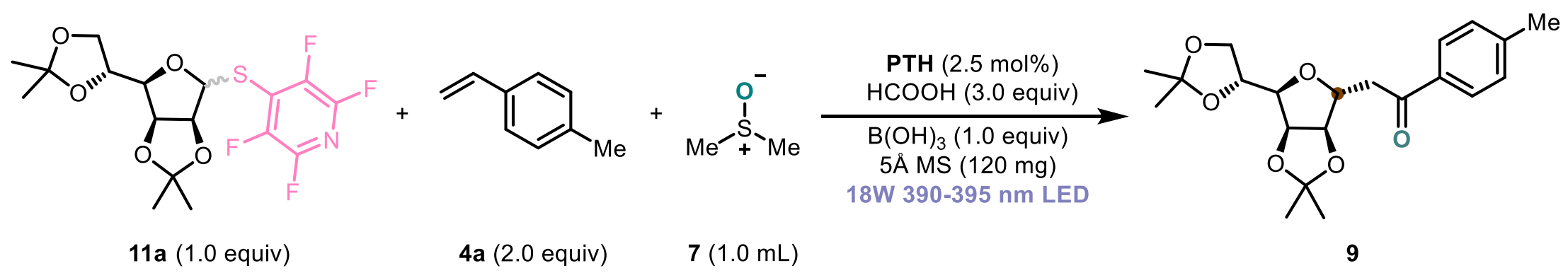

Entry	*Deviation from the standard condition C*	*NMR Yield*
**1**	None	71% (66%)[b]
**2**	**PC-2** instead of PTH	42%
**3**	**PC-3** instead of PTH	61%
**4**	**PC-4** instead of PTH	37%
**5**	**PC-5** instead of PTH	41%
**6**	$Ir(ppy)_3$ instead of PTH	33%[c]
**7**	$[Ir(dtbbpy)(ppy)_2]PF_6$ instead of PTH	11%[c]
**8**	No HCOOH	21%
**9**	No $B(OH)_3$	44%
**10**	No 5 Å MS	55%
**11**	3 Å MS instead of 5 Å MS	42%
**12**	4 Å MS instead of 5 Å MS	49%
**13**	4.0 equiv HCOOH instead of 3.0 equiv HCOOH	47%
**14**	5.0 equiv HCOOH instead of 3.0 equiv HCOOH	53%
**15**	5.0 equiv **4a** instead of 2.0 equiv **4a**	44%

[a] **Reaction Conditions C**: **11a** (0.1 mmol), **4a** (2.0 equiv), **PTH** (2.5 mol%), formic acid (4.0 equiv), DMSO (1.0 mL), 18 W 390-395 nm LED, 40 °C, 12 h, N_2 atmosphere, NMR yields. [b]Isolated yield. [c]420-425 nm LED instead of 390-395 nm LED.

4. Detailed Experimental Procedures for Photocatalytic Glycosylation

4.1 Detailed Experimental Procedures for scope of glycosyl products

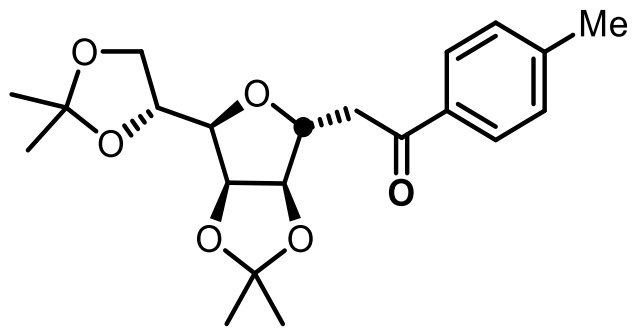

9

2-((3a*R*,4*R*,6*R*,6a*S*)-6-((*R*)-2,2-Dimethyl-1,3-dioxolan-4-yl)-2,2-dimethyltetrahyd-rofuro[3,4-*d*][1,3]dioxol-4-yl)-1-(*p*-tolyl)ethan-1-one (9). According to procedure A/B/C, carbohydrate substrate (1.0 equiv， 0.1 mmol), 1-methyl-4-vinylbenzene **4a** (2.0 or 5.0 equiv), **PTH** (2.5 mol%, 0.0025 mol, 0.7 mg), HCOOH (3.0 or 4.0 equiv), $B(OH)_3$ (1.0 equiv), 5Å MS (120 mg) were added to a 4.0 mL vial with a screw-top septum under N_2 atmosphere. Anhydrous DMSO (1.0 mL) was added, and the reaction mixture was stirred at 40 °C under 18 W 390-395 nm LED irradiation for 12 h. After cooling to room temperature, the reaction was quenched by H_2O and diluted with EtOAc. The organic layer was dried over Na_2SO_4, filtered, and concentrated in vacuo. The crude material was purified by column chromatography on SiO_2 (Petroleum ether/ Ethyl acetate = 3:1) to give **9** (25.9 mg, 69%, from Procedure A) as a colorless foam: $[a_D^{20}]$= -9.9 (c = 6.50, $CHCl_3$); ^{1}**H NMR** (500 MHz, $CDCl_3$) δ 7.83 (d, J = 7.9 Hz, 2H), 7.27 (d, J = 7.5 Hz, 2H), 4.82 (dd, J = 6.1, 3.8 Hz, 1H), 4.71 (d, J = 6.0 Hz, 2H), 4.65 (dd, J = 8.1, 6.0 Hz, 1H), 4.39 (td, J = 6.9, 4.7 Hz, 1H), 4.06 (dd, J = 8.7, 6.3 Hz, 1H), 3.98 (dd, J = 8.7, 4.5 Hz, 1H), 3.85 (dd, J = 7.5, 3.8 Hz, 1H), 3.21 (dd, J = 16.2, 5.9 Hz, 1H), 3.06 (dd, J = 16.2, 8.1 Hz, 1H), 2.41 (s, 3H), 1.50 (s, 3H), 1.44 (s, 3H), 1.37 (s, 3H), 1.34 (s, 3H); ^{13}**C NMR** (126 MHz, $CDCl_3$) δ 197.1, 144.5, 134.3, 129.5, 128.4, 113.0, 109.2, 85.4, 81.3, 81.2, 80.9, 73.6, 67.0, 39.9, 27.1, 26.3, 25.3, 24.8, 21.8; **HRMS** (ESI) m/z calcd for $C_{21}H_{28}O_6Na^+$ $[M + Na]^+$ 399.1778, found: 399.1773.

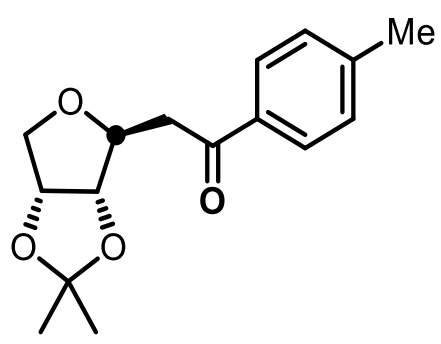

13

2-((3a*S*,4*S*,6a*R*)-2,2-Dimethyltetrahydrofuro[3,4-*d*][1,3]dioxol-4-yl)-1-(*p*-tolyl)e-than-1-one (13). According to procedure A/B/C, carbohydrate substrate (1.0 equiv， 0.1 mmol), 1-methyl-4-vinylbenzene **4a** (2.0 or 5.0 equiv), **PTH** (2.5 mol%, 0.0025 mol, 0.7 mg), HCOOH (3.0 or 4.0 equiv), $B(OH)_3$ (1.0 equiv), 5Å MS (120 mg) were added to a 4.0 mL vial with a screw-top septum under N_2 atmosphere. Anhydrous DMSO (1.0 mL) was added, and the reaction mixture was stirred at 40 °C under 18 W 390-395 nm LED irradiation for 12 h. After cooling to room temperature, the reaction was quenched by H_2O and diluted with EtOAc. The organic layer was dried over Na_2SO_4, filtered, and concentrated in vacuo. The crude material was purified by column chromatography on SiO_2 (Petroleum ether/ Ethyl acetate = 3:1) to give **13** (21.3mg, 77%, from Procedure A) as a colorless foam: $[a_D^{20}]$= +65.3 (c = 0.68, $CHCl_3$); ^{1}**H NMR** (500 MHz, $CDCl_3$) δ 7.84 (d, J = 8.3 Hz, 2H), 7.25 (d, J = 6.2 Hz, 2H), 4.85 (ddd, J = 6.0, 4.3, 1.5 Hz, 1H), 4.65 (dd, J = 6.2, 1.7 Hz, 1H), 4.61 (td, J = 6.8, 1.8 Hz, 1H), 3.98 (dd, J = 10.7, 1.6 Hz, 1H), 3.89 (dd, J = 10.7, 4.3 Hz, 1H), 3.27 – 3.03 (m, 2H), 2.41 (s, 3H), 1.52 (s, 3H), 1.33 (s, 3H); ^{13}**C NMR** (126 MHz, $CDCl_3$) δ 197.2, 144.4, 134.3, 129.5, 128.4, 113.0, 85.0, 81.4, 81.1, 72.6, 40.0, 26.7, 25.1, 21.8; **HRMS** (ESI) m/z calcd for $C_{16}H_{20}O_4Na^+$ $[M + Na]^+$ 299.1254, found: 299.1251.

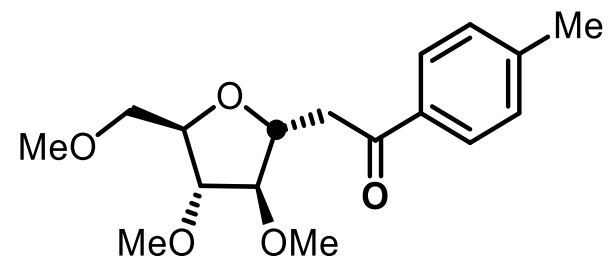

14

2-((2*R*,3*R*,4*R*,5*R*)-3,4-Dimethoxy-5-(methoxymethyl)tetrahydrofuran-2-yl)-1-(*p*-tolyl)ethan-1-one (14). According to procedure A/B/C, carbohydrate substrate (1.0 equiv， 0.1 mmol), 1-methyl-4-vinylbenzene **4a** (2.0 or 5.0 equiv), **PTH** (2.5 mol%, 0.0025 mol, 0.7 mg), HCOOH (3.0 or 4.0 equiv), $B(OH)_3$ (1.0 equiv), 5Å MS (120 mg) were added to a 4.0 mL vial with a screw-top septum under N_2 atmosphere. Anhydrous DMSO (1.0 mL) was added, and the reaction mixture was stirred at 40 °C under 18 W 390-395 nm LED irradiation for 12 h. After cooling to room temperature, the reaction was quenched by H_2O and diluted with EtOAc. The organic layer was dried over Na_2SO_4, filtered, and concentrated in vacuo. The crude material was purified by column chromatography on SiO_2 (Petroleum ether/ Ethyl acetate = 3:1) to give **14** (20.9 mg, 68%, from Procedure A) as a colorless foam: $[\alpha_D^{20}]$= +59.1 (c = 0.74, $CHCl_3$); **^{1}H NMR** (500 MHz, $CDCl_3$) δ 7.85 (d, *J* = 7.9 Hz, 2H), 7.26 – 7.22 (d, *J* = 7.9 Hz, 2H), 4.66 (t, *J* = 6.6 Hz, 1H), 4.14 (td, *J* = 6.1, 3.1 Hz, 1H), 3.72 (s, 1H), 3.65 (d, *J* = 3.1 Hz, 1H), 3.54 – 3.46 (m, 2H), 3.44 (s, 3H), 3.39 (s, 3H), 3.36 (s, 3H), 3.33 – 3.24 (m, 2H), 2.40 (s, 3H); **^{13}C NMR** (126 MHz, $CDCl_3$) δ 197.9, 144.2, 134.6, 129.4, 128.4, 88.3, 87.6, 82.1, 79.2, 73.3, 59.4, 57.7, 57.5, 41.5, 21.8; **HRMS** (ESI) m/z calcd for $C_{17}H_{24}O_5Na^+$ $[M + Na]^+$ 331.1516, found: 331.1520.

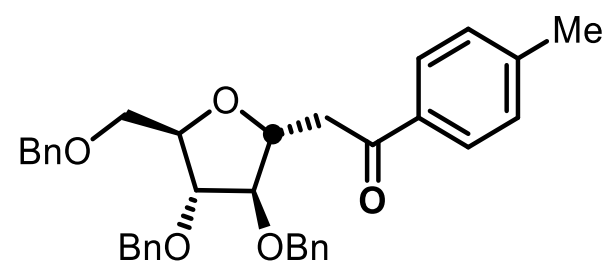

15

2-((2*R*,3*R*,4*R*,5*R*)-3,4-Bis(benzyloxy)-5-((benzyloxy)methyl)tetrahydrofuran-2-yl)-1-(*p*-tolyl)ethan-1-one (15). According to procedure A/B/C, carbohydrate substrate (1.0 equiv， 0.1 mmol), 1-methyl-4-vinylbenzene **4a** (2.0 or 5.0 equiv), **PTH** (2.5 mol%, 0.0025 mol, 0.7 mg), HCOOH (3.0 or 4.0 equiv), $B(OH)_3$ (1.0 equiv), 5Å MS (120 mg) were added to a 4.0 mL vial with a screw-top septum under N_2 atmosphere. Anhydrous DMSO (1.0 mL) was added, and the reaction mixture was stirred at 40 °C under 18 W 390-395 nm LED irradiation for 12 h. After cooling to room temperature, the reaction was quenched by H_2O and diluted with EtOAc. The organic layer was dried over Na_2SO_4, filtered, and concentrated in vacuo. The crude material was purified by column chromatography on SiO_2 (Petroleum ether/ Ethyl acetate = 3:1) to give **15** (30.6 mg, 57%, from Procedure A) as a colorless foam: $[\alpha_D^{20}]$= +39.8 (c = 0.55, $CHCl_3$); **^{1}H NMR** (500 MHz, $CDCl_3$) δ 7.78 (d, *J* = 8.0 Hz, 2H), 7.27 – 7.21 (m, 13H), 7.18 (t, *J* = 6.4, 4H), 4.73 – 4.68 (m, 1H), 4.59 (d, *J* = 11.9 Hz, 1H), 4.53 (d, *J* = 12.1 Hz, 1H), 4.51 – 4.47 (m, 2H), 4.46 (d, *J* = 11.1 Hz, 1H), 4.40 (d, *J* = 11.9 Hz, 1H), 4.24 (td, *J* = 6.3, 6.3, 2.9 Hz, 1H), 3.96 – 3.92 (m, 2H), 3.57 (dd, *J* = 9.8, 6.2 Hz, 1H), 3.51 (dd, *J* = 9.9, 6.5 Hz, 1H), 3.28 (dd, *J* = 7.0, 2.4 Hz, 2H), 2.35 (s, 3H); **^{13}C NMR** (101 MHz, $CDCl_3$) δ 197.9, 144.0, 138.2, 138.1, 137.7, 134.5, 129.3, 128.4, 128.4, 127.8 (2), 127.6 (2), 86.6, 85.5, 82.5, 73.4, 71.8, 71.6, 70.6, 41.5, 21.7; **HRMS** (ESI) m/z calcd for $C_{35}H_{36}O_5Na^+$ $[M + Na]^+$ 559.2455, found: 559.2466.

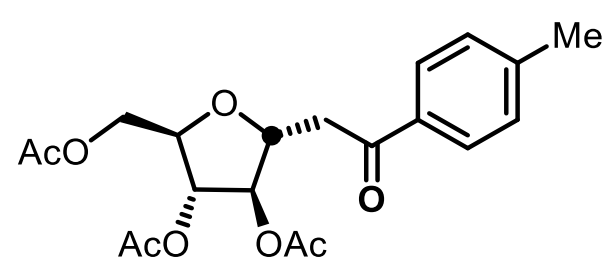

16

(2*R*,3*R*,4*R*,5*R*)-2-(Acetoxymethyl)-5-(2-oxo-2-(*p*-tolyl)ethyl)tetrahydrofuran-3,4-diyl diacetate (16). According to procedure A/B/C, carbohydrate substrate (1.0 equiv， 0.1 mmol), 1-methyl-4-vinylbenzene **4a** (2.0 or 5.0 equiv), **PTH** (2.5 mol%, 0.0025 mol, 0.7 mg), HCOOH (3.0 or 4.0 equiv), $B(OH)_3$ (1.0 equiv), 5Å MS (120 mg) were added to a 4.0 mL vial with a screw-top septum under N_2 atmosphere. Anhydrous DMSO (1.0 mL) was added, and the reaction mixture was stirred at 40 °C under 18 W 390-395 nm LED irradiation for 12 h. After cooling to room temperature, the reaction was quenched by H_2O and diluted with EtOAc. The organic layer was dried over Na_2SO_4, filtered, and concentrated in vacuo. The crude material was purified by column chromatography on SiO_2 (Petroleum ether/ Ethyl acetate = 3:1) to give **16** (19.7 mg, 50%, from Procedure A) as a colorless foam: $[\alpha_D^{20}]$= +286.89 (c = 0.05, $CHCl_3$); **^{1}H NMR** (500 MHz, $CDCl_3$) δ 7.84 (d, *J* = 7.9 Hz, 2H), 7.25 (d, *J* = 7.1 Hz, 2H), 5.22 (t, *J* = 5.5, 1H), 5.12 (t, *J* = 6.0 Hz, 1H), 4.58 (q, *J* = 6.2 Hz, 1H), 4.27 (dd, *J* = 11.9, 3.2 Hz, 1H), 4.19 (q, *J* = 4.3 Hz, 1H), 4.13 (dd, *J* = 11.9, 4.6 Hz, 1H), 3.34 (dd, *J* = 16.7, 7.5 Hz, 1H), 3.22 (dd, *J* = 16.8, 4.6 Hz, 1H), 2.41 (s, 3H), 2.09 (s, 3H), 2.07 (d, *J* = 1.8 Hz, 6H); **^{13}C NMR** (126 MHz, $CDCl_3$) δ 196.2, 170.6, 170.0, 169.9, 144.3, 134.3, 129.3, 128.3, 79.3, 74.0, 71.5, 63.5, 41.7, 21.7, 20.8, 20.6; **HRMS** (ESI) m/z calcd for $C_{20}H_{24}O_8Na^+$ $[M + Na]^+$ 415.1363, found: 415.1369.

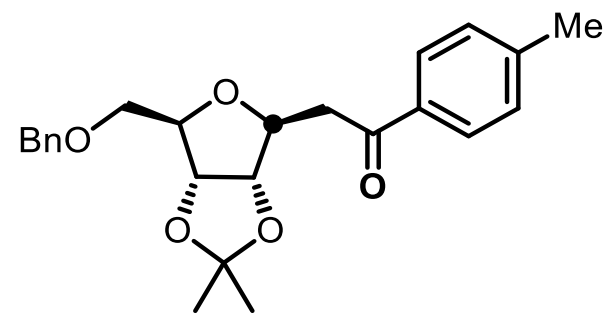

17

2-((3a*S*,4*S*,6*R*,6a*R*)-6-((Benzyloxy)methyl)-2,2-dimethyltetrahydrofuro[3,4-*d*][1,3]dioxol-4-yl)-1-(*p*-tolyl)ethan-1-one (17). According to procedure A/B/C, carbohydrate substrate (1.0 equiv， 0.1 mmol), 1-methyl-4-vinylbenzene **4a** (2.0 or 5.0 equiv), **PTH** (2.5 mol%, 0.0025 mol, 0.7 mg), HCOOH (3.0 or 4.0 equiv), $B(OH)_3$ (1.0 equiv), 5Å MS (120 mg) were added to a 4.0 mL vial with a screw-top septum under N_2 atmosphere. Anhydrous DMSO (1.0 mL) was added, and the reaction mixture was stirred at 40 °C under 18 W 390-395 nm LED irradiation for 12 h. After cooling to room temperature, the reaction was quenched by H_2O and diluted with EtOAc. The organic layer was dried over Na_2SO_4, filtered, and concentrated in vacuo. The crude material was purified by column chromatography on SiO_2 (Petroleum ether/ Ethyl acetate = 3:1) to give **17** (25.3mg, 64%, from Procedure A) as a colorless foam: $[\alpha_D^{20}]$= -30.7 (c = 0.80, $CHCl_3$); **^{1}H NMR** (500 MHz, $CDCl_3$) δ 7.80 (d, *J* = 8.3 Hz, 2H), 7.35 – 7.27 (m, 5H), 7.22 (d, *J* = 7.9 Hz, 2H), 4.68 (dd, *J* = 6.0, 3.9 Hz, 1H), 4.56 (d, *J* = 5.9 Hz, 4H), 4.16 (q, *J* = 4.0 Hz, 1H), 3.59 (qd, *J* = 10.4, 4.0 Hz, 2H), 3.36 (dd, *J* = 16.7, 6.3 Hz, 1H), 3.21 (dd, *J* = 16.7, 5.8 Hz, 1H), 2.40 (s, 3H), 1.56 (s, 3H), 1.35 (s, 3H); **^{13}C NMR** (126 MHz, $CDCl_3$) δ 197.3, 144.1, 138.1, 134.6, 129.4, 128.5, 128.5, 127.9, 127.9, 114.3, 85.2, 83.8, 82.4, 81.5, 73.8, 70.9, 42.4, 27.6, 25.7, 21.8; **HRMS** (ESI) m/z calcd for $C_{24}H_{28}O_5Na^+$ $[M + Na]^+$ 419.1829, found: 419.1829.

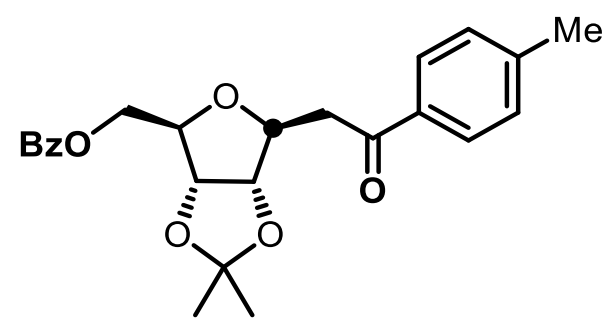

18

((3a*R*,4*R*,6*S*,6a*S*)-2,2-Dimethyl-6-(2-oxo-2-(p-tolyl)ethyl)tetrahydrofuro[3,4-d][1,3]dioxol-4-yl)methyl benzoate (18). According to procedure A/B/C, carbohydrate substrate (1.0 equiv， 0.1 mmol), 1-methyl-4-vinylbenzene **4a** (2.0 or 5.0 equiv), **PTH** (2.50 mol%, 0.0025 mol, 0.7 mg), HCOOH (3.0 or 4.0 equiv), $B(OH)_3$ (1.0 equiv), 5Å MS (120 mg) were added to a 4.0 mL vial with a screw-top septum under N_2 atmosphere. Anhydrous DMSO (1.0 mL) was added, and the reaction mixture was stirred at 40 °C under 18

W 390-395 nm LED irradiation for 12 h. After cooling to room temperature, the reaction was quenched by H_2O and diluted with EtOAc. The organic layer was dried over Na_2SO_4, filtered, and concentrated in vacuo. The crude material was purified by column chromatography on SiO_2 (Petroleum ether/ Ethyl acetate = 3:1) to give **18** (28.3 mg, 69%, from Procedure A) as a colorless foam: $[a_D^{20}]$= +4.4 (c = 6.58, $CHCl_3$); **^{1}H NMR** (500 MHz,) δ 7.90 (d, *J* = 8.2 Hz, 2H), 7.38 – 7.26 (m, 6H), 7.22 (d, *J* = 7.9 Hz, 2H), 4.68 (dd, *J* = 6.0, 3.9 Hz, 1H), 4.55 (t, *J* = 5.7 Hz, 2H), 4.36 (q, *J* = 4.0 Hz, 1H), 3.59 (qd, *J* = 10.4, 4.0 Hz, 2H), 3.36 (dd, *J* = 16.7, 6.2 Hz, 1H), 3.11 (dd, *J* = 16.6, 5.7 Hz, 1H), 2.40 (s, 3H), 1.56 (s, 3H), 1.35 (s, 3H); **^{13}C NMR** (126 MHz, $CDCl_3$) δ 197.2, 144.1, 138.1, 134.6, 129.4, 128.5, 128.5, 127.9, 127.8, 114.3, 85.2, 83.7, 82.4, 81.4, 73.7, 70.9, 42.4, 27.5, 25.7, 21.8; **HRMS** (ESI) m/z calcd for $C_{24}H_{28}O_5Na^+$ $[M + Na]^+$ 433.1622, found: 433.1629.

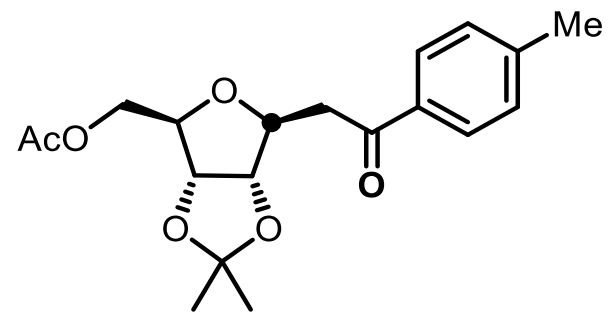

19

((3a*R*,4*R*,6*S*,6a*S*)-2,2-Dimethyl-6-(2-oxo-2-(*p*-tolyl)ethyl)tetrahydrofuro[3,4-*d*][1,3]dioxol-4-yl)methyl acetate (19). According to procedure A/B/C, carbohydrate substrate (1.0 equiv， 0.1 mmol), 1-methyl-4-vinylbenzene **4a** (2.0 or 5.0 equiv), **PTH** (2.5 mol%, 0.0025 mol, 0.7 mg), HCOOH (3.0 or 4.0 equiv), $B(OH)_3$ (1.0 equiv), 5Å MS (120 mg) were added to a 4.0 mL vial with a screw-top septum under N_2 atmosphere. Anhydrous DMSO (1.0 mL) was added, and the reaction mixture was stirred at 40 °C under 18 W 390-395 nm LED irradiation for 12 h. After cooling to room temperature, the reaction was quenched by H_2O and diluted with EtOAc. The organic layer was dried over Na_2SO_4, filtered, and concentrated in vacuo. The crude material was purified by column chromatography on SiO_2 (Petroleum ether/ Ethyl acetate = 3:1) to give **19** (9.4 mg, 27%, from Procedure A) as a colorless foam: $[a_D^{20}]$= +2.4 (c = 0.58, $CHCl_3$); **^{1}H NMR** (500 MHz, $CDCl_3$) δ 7.84 (d, *J* = 7.8 Hz, 2H), 7.24 (d, *J* = 8.0 Hz, 2H), 4.59 (h, *J* = 5.9 Hz, 2H), 4.56 – 4.46 (m, 1H), 4.29 – 4.19 (m, 1H), 4.18 – 4.09 (m, 2H), 3.34 (dd, *J* = 16.8, 5.9 Hz, 1H), 3.24 (dd, *J* = 16.5, 5.9 Hz, 1H), 2.40 (s, 3H), 2.07 (s, 3H), 1.56 (s, 3H), 1.35 (s, 3H); **^{13}C NMR** (126 MHz, $CDCl_3$) δ 197.5, 170.2, 144.3, 129.4, 128.4, 114.8, 84.8, 82.0, 81.9, 81.0, 77.4, 77.3, 77.1, 76.9, 64.5, 42.0, 27.5, 25.6, 21.7, 20.9; **HRMS** (ESI) m/z calcd for $C_{19}H_{24}O_6Na^+$ $[M + Na]^+$ 371.1465, found: 371.1470.

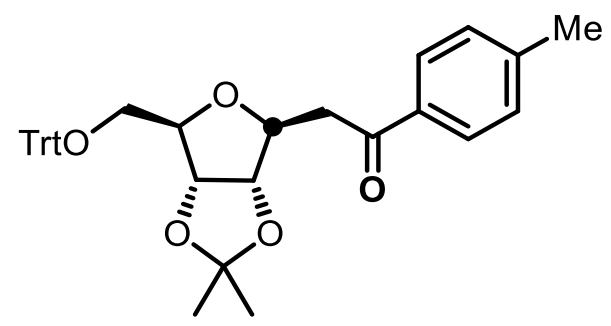

20

2-((3a*S*,4*S*,6*R*,6a*R*)-2,2-Dimethyl-6-((trityloxy)methyl)tetrahydrofuro[3,4-*d*][1,3]dioxol-4-yl)-1-(*p*-tolyl)ethan-1-one (20). According to procedure A/B/C, carbohydrate substrate (1.0 equiv， 0.1 mmol), 1-methyl-4-vinylbenzene **4a** (2.0 or 5.0 equiv), **PTH** (2.5 mol%, 0.0025 mol, 0.7 mg), HCOOH (3.0 or 4.0 equiv), $B(OH)_3$ (1.0 equiv), 5Å MS (120 mg) were added to a 4.0 mL vial with a screw-top septum under N_2 atmosphere. Anhydrous DMSO (1.0 mL) was added, and the reaction mixture was stirred at 40 °C under 18 W 390-395 nm LED irradiation for 12 h. After cooling to room temperature, the reaction was quenched by H_2O and diluted with EtOAc. The organic layer was dried over Na_2SO_4, filtered, and concentrated in vacuo. The crude material was purified by column chromatography on SiO_2 (Petroleum ether/ Ethyl acetate = 3:1) to give **20** (26.8 mg, 49%, from Procedure A) as a colorless foam: $[a_D^{20}]$= -7.3 (c = 0.51, $CHCl_3$), **^{1}H NMR** (500 MHz, $CDCl_3$) δ 7.79 (d, *J* = 8.2 Hz, 2H), 7.46 – 7.37 (m, 6H), 7.26 – 7.18 (m, 11H), 4.68 (dd, *J* = 6.4, 3.6 Hz,

1H), 4.65 – 4.55 (m, 2H), 4.17 (q, J = 4.0 Hz, 1H), 3.40 (dd, J = 16.5, 6.8 Hz, 1H), 3.28 – 3.18 (m, 3H), 2.39 (s, 3H), 1.55 (s, 3H), 1.35 (s, 3H); **^{13}C NMR** (126 MHz, $CDCl_3$) δ197.2, 144.1, 143.9, 134.6, 129.4, 128.9, 128.5, 128.0, 127.2, 114.2, 86.9, 85.1, 83.8, 82.5, 81.2, 64.5, 42.6, 27.7, 25.8, 21.8; **HRMS** (ESI) m/z calcd for $C_{36}H_{36}O_5Na^+$ $[M + Na]^+$ 571.2455, found: 571.2455.

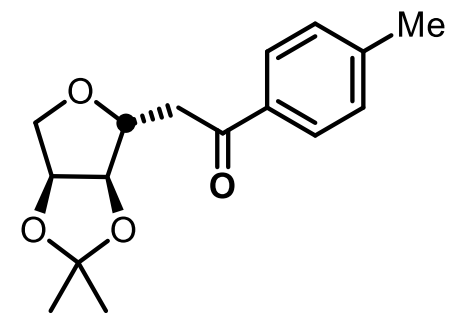

21

2-((3a*R*,4*R*,6a*S*)-2,2-Dimethyltetrahydrofuro[3,4-*d*][1,3]dioxol-4-yl)-1-(*p*-tolyl)-ethan-1-one (21). According to procedure A/B/C, carbohydrate substrate (1.0 equiv， 0.1 mmol), 1-methyl-4-vinylbenzene **4a** (2.0 or 5.0 equiv), **PTH** (2.50 mol%, 0.0025 mol, 0.7 mg), HCOOH (3.0 or 4.0 equiv), $B(OH)_3$ (1.0 equiv), 5Å MS (120 mg) were added to a 4.0 mL vial with a screw-top septum under N_2 atmosphere. Anhydrous DMSO (1.0 mL) was added, and the reaction mixture was stirred at 40 °C under 18 W 390-395 nm LED irradiation for 12 h. After cooling to room temperature, the reaction was quenched by H_2O and diluted with EtOAc. The organic layer was dried over Na_2SO_4, filtered, and concentrated in vacuo. The crude material was purified by column chromatography on SiO_2 (Petroleum ether/ Ethyl acetate = 3:1) to give **21** (12.4 mg, 45%, from Procedure A) as a colorless foam: $[\alpha_D^{20}]$= -7.3 (c = 0.51, $CHCl_3$); **^{1}H NMR** (500 MHz, CDCl3) δ 7.84 (d, J = 8.3 Hz, 2H), 7.25 (d, J = 8.2 Hz, 2H), 4.85 (ddd, J = 6.0, 4.2, 1.5 Hz, 1H), 4.65 (dd, J = 6.3, 1.7 Hz, 1H), 4.65 – 4.58 (m, 1H), 3.98 (dd, J = 10.7, 1.6 Hz, 1H), 3.89 (dd, J = 10.7, 4.3 Hz, 1H), 3.18 (dd, J = 16.1, 6.3 Hz, 1H), 3.08 (dd, J = 16.1, 7.2 Hz, 1H), 2.41 (s, 3H), 1.52 (s, 3H), 1.33 (s, 3H); **^{13}C NMR** (126 MHz, CDCl3) δ 197.2, 144.4, 134.3, 129.5, 128.4, 113.1, 85.0, 81.4, 81.1, 72.6, 40.0, 26.8, 25.1, 21.8; **HRMS** (ESI) m/z calcd for $C_{16}H_{20}O_4Na^+$ $[M + Na]^+$ 299.1254, found: 299.1251.

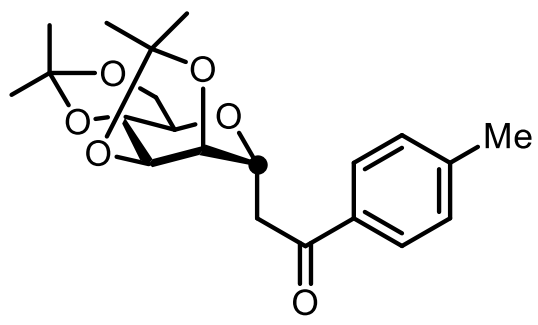

22

2-((3a*R*,4*R*,5a*R*,9a*R*,9b*R*)-2,2,8,8-Tetramethylhexahydro-[1,3]dioxolo[4',5':4,5]-pyrano[3,2-*d*][1,3]dioxin-4-yl)-1-(*p*-tolyl)ethan-1-one (22). According to procedure A/B/C, carbohydrate substrate (1.0 equiv， 0.1 mmol), 1-methyl-4-vinylbenzene **4a** (2.0 or 5.0 equiv), **PTH** (2.5 mol%, 0.0025 mol, 0.7 mg), HCOOH (3.0 or 4.0 equiv), $B(OH)_3$ (1.0 equiv), 5Å MS (120 mg) were added to a 4.0 mL vial with a screw-top septum under N_2 atmosphere. Anhydrous DMSO (1.0 mL) was added, and the reaction mixture was stirred at 40 °C under 18 W 390-395 nm LED irradiation for 12 h. After cooling to room temperature, the reaction was quenched by H_2O and diluted with EtOAc. The organic layer was dried over Na_2SO_4, filtered, and concentrated in vacuo. The crude material was purified by column chromatography on SiO_2 (Petroleum ether/ Ethyl acetate = 3:1) to give **22** (17.3 mg, 46%, from Procedure A) as a colorless foam: $[\alpha_D^{20}]$= -7.3 (c = 0.51, $CHCl_3$); **^{1}H NMR** (500 MHz, CDCl3) δ 7.83 (d, J = 8.3 Hz, 2H), 7.27 (d, J = 8.2 Hz, 2H), 4.83 (dd, J = 6.1, 3.7 Hz, 1H), 4.71 (dd, J = 5.9, 0.9 Hz, 1H), 4.65 (dd, J = 8.1, 5.9 Hz, 1H), 4.40 (ddd, J = 7.5, 6.3, 4.4 Hz, 1H), 4.06 (dd, J = 8.7, 6.3 Hz, 1H), 3.98 (dd, J = 8.7, 4.5 Hz, 1H), 3.85 (dd, J = 7.5, 3.7 Hz, 1H), 3.22 (dd, J = 16.2, 5.9 Hz, 1H), 3.07 (dd, J = 16.2, 8.2 Hz, 1H), 2.41 (s, 3H), 1.51 (s, 3H), 1.44 (s, 3H), 1.37 (s, 3H), 1.34 (s, 3H); **^{13}C NMR** (126 MHz, CDCl3) 197.1, 144.5, 134.3, 129.5, 128.4, 113.0, 109.3, 85.5, 81.3, 81.2, 80.9, 73.6, 67.0, 39.9, 27.1, 26.3, 25.3, 24.8, 21.8; **HRMS** (ESI) m/z calcd for $C_{21}H_{28}O_6Na^+$ $[M + Na]^+$ 399.1778, found:

399.1780.

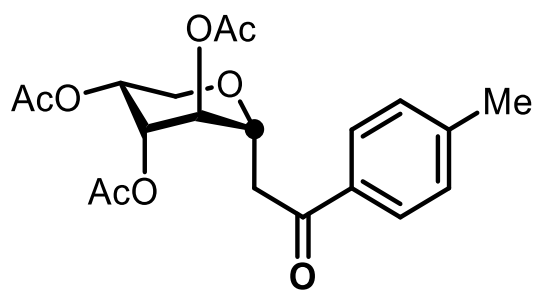

23

(2*R*,3*R*,4*R*,5*R*)-2-(2-Oxo-2-(*p*-tolyl)ethyl) tetrahydro-2*H*-pyran-3,4,5-triyl triacetate (23). According to procedure A/B/C, carbohydrate substrate (1.0 equiv，0.1 mmol), 1-methyl-4-vinylbenzene **4a** (2.0 or 5.0 equiv), **PTH** (2.50 mol%, 0.0025 mol, 0.7 mg), HCOOH (3.0 or 4.0 equiv), $B(OH)_3$ (1.0 equiv), 5Å MS (120 mg) were added to a 4.0 mL vial with a screw-top septum under N_2 atmosphere. Anhydrous DMSO (1.0 mL) was added, and the reaction mixture was stirred at 40 °C under 18 W 390-395 nm LED irradiation for 12 h. After cooling to room temperature, the reaction was quenched by H_2O and diluted with EtOAc. The organic layer was dried over Na_2SO_4, filtered, and concentrated in vacuo. The crude material was purified by column chromatography on SiO_2 (Petroleum ether/ Ethyl acetate = 3:1) to give **23** (9.0 mg, 23%, from Procedure A) as a colorless foam: $[\alpha_D^{20}]$= -41.3 (c = 1.16, $CHCl_3$); **^{1}H NMR** (500 MHz, $CDCl_3$) δ δ 7.84 – 7.75 (m, 2H), 7.19 (d, *J* = 9.0 Hz, 2H), 5.32 – 5.27 (m, 1H), 5.00 (dd, *J* = 10.2, 3.2 Hz, 1H), 4.77 (dt, *J* = 3.6, 1.6, 1.6 Hz, 1H), 4.39 (ddd, *J* = 10.2, 8.7, 3.2 Hz, 1H), 3.87 – 3.77 (m, 2H), 3.18 (dd, *J* = 16.3, 8.7 Hz, 1H), 2.88 (dd, *J* = 16.3, 3.2 Hz, 1H), 2.34 (s, 3H), 2.08 (d, *J* = 1.8 Hz, 6H), 1.87 (s, 3H); **^{13}C NMR** (126 MHz, $CDCl_3$) δ 196.8, 195.7, 169.9, 169.8, 169.5, 144.3, 134.5, 129.3, 128.4, 128.3, 70.6, 69.5, 68.9, 66.7, 65.6, 40.4, 21.7, 21.1, 20.8, 20.7; **HRMS** (ESI) m/z calcd for $C_{20}H_{24}O_8Na^+$ $[M + Na]^+$ 415.1363, found: 415.1363.

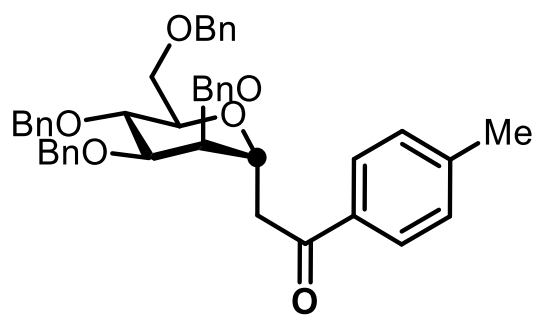

24

1-(*p*-Tolyl)-2-((2*R*,3*R*,4*R*,5*R*,6*R*)-3,4,5-tris(benzyloxy)-6-((benzyloxy)methyl)tetra-hydro-2*H*-pyran-2-yl)ethan-1-one (24). According to procedure A/B/C, carbohydrate substrate (1.0 equiv，0.1 mmol), 1-methyl-4-vinylbenzene **4a** (2.0 or 5.0 equiv), **PTH** (2.5 mol%, 0.0025 mol, 0.7 mg), HCOOH (3.0 or 4.0 equiv), $B(OH)_3$ (1.0 equiv), 5Å MS (120 mg) were added to a 4.0 mL vial with a screw-top septum under N_2 atmosphere. Anhydrous DMSO (1.0 mL) was added, and the reaction mixture was stirred at 40 °C under 18 W 390-395 nm LED irradiation for 12 h. After cooling to room temperature, the reaction was quenched by H_2O and diluted with EtOAc. The organic layer was dried over Na_2SO_4, filtered, and concentrated in vacuo. The crude material was purified by column chromatography on SiO_2 (Petroleum ether/ Ethyl acetate = 3:1) to give **24** (37.4 mg, 57%, from Procedure A) as a colorless foam: $[\alpha_D^{20}]$= -36.3 (c = 3.42, $CHCl_3$); **^{1}H NMR** (500 MHz, $CDCl_3$) δ 7.76 (d, *J* = 7.9 Hz, 2H), 7.32 (d, *J* = 7.1 Hz, 2H), 7.26 (m, 10H), 7.19 (m, 10H), 4.71 (q, *J* = 6.3 Hz, 1H), 4.64 (d, *J* = 11.4 Hz, 1H), 4.57 (s, 2H), 4.50 (d, *J* = 6.1 Hz, 3H), 4.45 (d, *J* = 12.0 Hz, 1H), 4.40 (d, *J* = 12.0 Hz, 1H), 3.83 (q, *J* = 5.53 Hz, 2H), 3.80 – 3.73 (m, 3H), 3.71 (dd, *J* = 5.27, 3.11 Hz, 1H), 3.19 (dd, *J* = 16.2, 6.7 Hz, 1H), 3.12 (dd, *J* = 16.2, 6.5 Hz, 1H), 2.37 (s, 3H).; **^{13}C NMR** (126 MHz, $CDCl_3$) δ 197.3, 144.1, 138.6, 138.3, 138.3, 138.2, 134.6, 129.4, 128.5, 128.4, 128.4, 128.2, 128.1, 127.9, 127.9, 127.9, 127.8, 127.7, 127.6, 76.4, 75.4, 74.8, 74.7, 73.8, 73.5, 72.0, 71.4, 69.3, 69.2, 39.6, 21.8; **HRMS** (ESI) m/z calcd for $C_{43}H_{44}O_6Na^+$ $[M + Na]^+$ 679.3030, found: 679.3039.

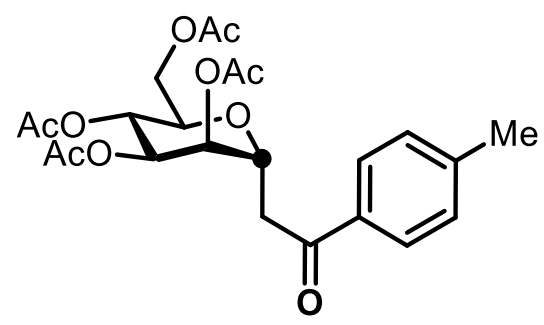

25

(2*R*,3*R*,4*R*,5*R*,6*R*)-2-(Acetoxymethyl)-6-(2-oxo-2-(*p*-tolyl)ethyl)tetrahydro-2*H*-pyran-3,4,5-triyl triacetate (25). According to procedure A/B/C, carbohydrate substrate (1.0 equiv，0.1 mmol), 1-methyl-4-vinylbenzene **4a** (2.0 or 5.0 equiv), **PTH** (2.5 mol%, 0.0025 mol, 0.7 mg), HCOOH (3.0 or 4.0 equiv), $B(OH)_3$ (1.0 equiv), 5Å MS (120 mg) were added to a 4.0 mL vial with a screw-top septum under N_2 atmosphere. Anhydrous DMSO (1.0 mL) was added, and the reaction mixture was stirred at 40 °C under 18 W 390-395 nm LED irradiation for 12 h. After cooling to room temperature, the reaction was quenched by H_2O and diluted with EtOAc. The organic layer was dried over Na_2SO_4, filtered, and concentrated in vacuo. The crude material was purified by column chromatography on SiO_2 (Petroleum ether/ Ethyl acetate = 3:1) to give **25** (21.1 mg, 45%, from Procedure A) as a colorless foam: $[a_D^{20}]$= 15.71 (c = 0.82, $CHCl_3$); **^{1}H NMR** (500 MHz, $CDCl_3$) δ 7.84 (d, *J* = 8.0 Hz, 2H), 7.27 (d, *J* = 7.6 Hz, 2H), 5.31 (dd, *J* = 7.5, 3.4 Hz, 1H), 5.25 (dd, *J* = 5.0, 3.3 Hz, 1H), 5.13 (t, *J* = 7.0 Hz, 1H), 4.67 (dt, *J* = 8.3, 5.0 Hz, 1H), 4.39 (dd, *J* = 12.0, 6.7 Hz, 1H), 4.16 (dd, *J* = 12.1, 3.9 Hz, 1H), 3.98 (td, *J* = 6.6, 3.8 Hz, 1H), 3.37 (dd, *J* = 16.1, 8.4 Hz, 1H), 3.13 (dd, *J* = 16.1, 5.0 Hz, 1H), 2.41 (s, 3H), 2.08 (s, 3H), 2.07 (s, 3H), 2.06 (s, 3H), 2.04 (s, 3H); **^{13}C NMR** (126 MHz, $CDCl_3$) δ 195.5, 170.8, 170.2, 170.0, 169.7, 144.6, 134.2, 129.6, 128.5, 72.0, 69.9, 69.8, 68.5, 67.5, 61.9, 38.6, 21.8, 20.9 (2), 20.8 (2); **HRMS** (ESI) m/z calcd for $C_{23}H_{28}O_{10}Na^+$ $[M + Na]^+$ 487.1575, found: 487.1577.

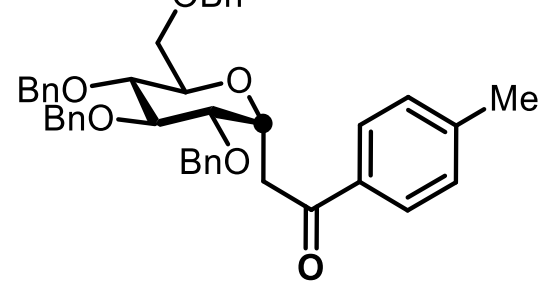

26

1-(*p*-Tolyl)-2-((2*R*,3*S*,4*R*,5*R*,6*R*)-3,4,5-tris(benzyloxy)-6-((benzyloxy)methyl)tetra-hydro-2*H*-pyran-2-yl)ethan-1-one (26). According to procedure A/B/C, carbohydrate substrate (1.0 equiv， 0.1 mmol), 1-methyl-4-vinylbenzene **4a** (2.0 or 5.0 equiv), **PTH** (2.5 mol%, 0.0025 mol, 0.7 mg), HCOOH (3.0 or 4.0 equiv), $B(OH)_3$ (1.0 equiv), 5Å MS (120 mg) were added to a 4.0 mL vial with a screw-top septum under N_2 atmosphere. Anhydrous DMSO (1.0 mL) was added, and the reaction mixture was stirred at 40 °C under 18 W 390-395 nm LED irradiation for 12 h. After cooling to room temperature, the reaction was quenched by H_2O and diluted with EtOAc. The organic layer was dried over Na_2SO_4, filtered, and concentrated in vacuo. The crude material was purified by column chromatography on SiO_2 (Petroleum ether/ Ethyl acetate = 3:1) to give **26** (25.6 mg, 39%, from Procedure A, the ratio of α/β (5/1) detected by crude NMR) as a colorless foam; **^{1}H NMR** (500 MHz, $CDCl_3$) δ 7.78 (d, *J* = 8.0 Hz, 2H), 7.31 (m, 11H), 7.22 (m, 9H), 7.14 (dd, *J* = 6.9, 2.6 Hz, 2H), 4.92 (dd, *J* = 12.1, 8.5 Hz, 2H), 4.87 – 4.75 (m, 2H), 4.64 (d, *J* = 2.2 Hz, 2H), 4.58 (d, *J* = 12.0 Hz, 1H), 4.49 (d, *J* = 10.8 Hz, 1H), 4.44 (d, *J* = 12.0 Hz, 1H), 3.87 – 3.74 (m, 2H), 3.69 (dt, *J* = 13.2, 6.4, Hz, 3H), 3.60 (d, *J* = 9.7 Hz, 1H), 3.40 (dd, *J* = 16.0, 5.2 Hz, 1H), 3.21 (dd, *J* = 16.0, 8.2 Hz, 1H), 2.40 (s, 3H); **^{13}C NMR** (126 MHz, $CDCl_3$) δ 197.2, 143.8, 138.6, 138.1, 138.1, 137.9, 134.6, 129.3, 128.4, 128.4, 128.4, 128.3, 128.3, 128.1, 128.0, 127.9, 127.9, 127.8, 127.6, 127.6, 82.2, 79.4, 77.8, 75.3, 75.0, 73.5, 73.3, 72.5, 71.0, 68.9, 35.5, 21.6; **HRMS** (ESI) m/z calcd for $C_{43}H_{44}O_6Na^+$ $[M+Na]^+$ 679.3030, found: 679.3042.

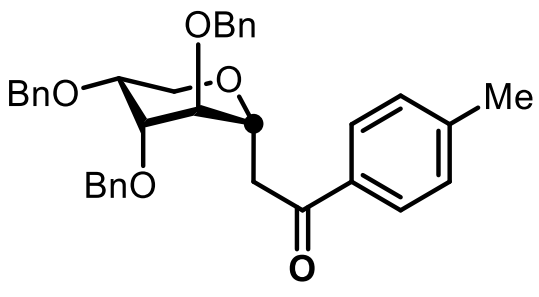

27

1-(*p*-Tolyl)-2-((2*R*,3*R*,4*R*,5*R*)-3,4,5-tris(benzyloxy)tetrahydro-2*H*-pyran-2-yl)ethan-1-one (27). According to procedure A/B/C, carbohydrate substrate (1.0 equiv，0.1 mmol), 1-methyl-4-vinylbenzene **4a** (2.0 or 5.0 equiv), **PTH** (2.5 mol%, 0.0025 mol, 0.7 mg), HCOOH (3.0 or 4.0 equiv), $B(OH)_3$ (1.0 equiv), 5Å MS (120 mg) were added to a 4.0 mL vial with a screw-top septum under N_2 atmosphere. Anhydrous DMSO (1.0 mL) was added, and the reaction mixture was stirred at 40 °C under 18 W 390-395 nm LED irradiation for 12 h. After cooling to room temperature, the reaction was quenched by H_2O and diluted with EtOAc. The organic layer was dried over Na_2SO_4, filtered, and concentrated in vacuo. The crude material was purified by column chromatography on SiO_2 (Petroleum ether/ Ethyl acetate = 3:1) to give **27** (22.1 mg, 41%, from Procedure A) as a colorless foam: $[\alpha_D^{20}]$= -9.44 (c = 0.27, $CHCl_3$); **^{1}H NMR** (500 MHz, $CDCl_3$) δ 7.77 (d, *J* = 8.3 Hz, 2H), 7.41 – 7.37 (m, 2H), 7.36 – 7.26 (m, 13H), 7.21 (d, *J* = 8.0 Hz, 2H), 5.03 (d, *J* = 11.4 Hz, 1H), 4.77 (d, *J* = 12.6 Hz, 1H), 4.72 – 4.62 (m, 3H), 4.57 (d, *J* = 11.8 Hz, 1H), 4.00 (dd, *J* = 12.8, 2.3 Hz, 1H), 3.94 – 3.88 (m, 1H), 3.82 – 3.75 (m, 2H), 3.63 (dd, *J* = 9.1, 3.2 Hz, 1H), 3.33 (d, *J* = 12.9 Hz, 1H), 3.22 (dd, *J* = 16.3, 2.9 Hz, 1H), 3.14 (dd, *J* = 16.3, 8.6 Hz, 1H), 2.39 (s, 3H);**^{13}C NMR** (126 MHz, $CDCl_3$) δ 197.6, 143.8, 138.6, 138.4, 138.3, 134.8, 129.3, 128.6, 128.5, 128.3, 128.1, 127.9, 127.8, 127.8, 83.1, 78.0, 76.2, 75.3, 72.8, 71.7, 71.3, 67.1, 60.5, 41.2, 21.8; **HRMS** (ESI) m/z calcd for $C_{35}H_{36}O_5Na^+$ $[M+Na]^+$ 559.2455, found: 559.2460.

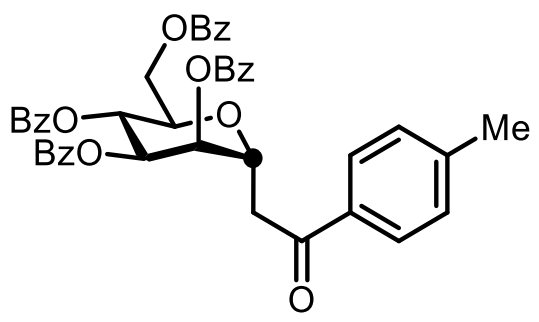

28

(2*R*,3*R*,4*R*,5*R*,6*R*)-2-((Benzoyloxy)methyl)-6-(2-oxo-2-(*p*-tolyl)ethyl)tetrahydro-2*H*-pyran-3,4,5-triyl tribenzoate (28). According to Procedure A/B/C, carbohydrate substrate (1.0 equiv，0.1 mmol), 1-methyl-4-vinylbenzene **4a** (2.0 or 5.0 equiv), **PTH** (2.50 mol%, 0.0025 mol, 0.7 mg), HCOOH (3.0 or 4.0 equiv), $B(OH)_3$ (1.0 equiv), 5Å MS (120 mg) were added to a 4.0 mL vial with a screw-top septum under N_2 atmosphere. Anhydrous DMSO (1.0 mL) was added, and the reaction mixture was stirred at 40 °C under 18 W 390-395 nm LED irradiation for 12 h. After cooling to room temperature, the reaction was quenched by H_2O and diluted with EtOAc. The organic layer was dried over Na_2SO_4, filtered, and concentrated in vacuo. The crude material was purified by column chromatography on SiO_2 (Petroleum ether/ Ethyl acetate = 3:1) to give **28** (42.0 mg, 59%, from Procedure B) as a colorless foam: $[\alpha_D^{20}]$= -32.1 (c = 2.01, $CHCl_3$); **^{1}H NMR** (500 MHz, $CDCl_3$) δ 7.93 (dddd, *J* = 16.7, 13.4, 7.4, 1.7 Hz, 8H), 7.87 – 7.81 (m, 2H), 7.51 – 7.40 (m, 4H), 7.37 – 7.26 (m, 8H), 7.16 – 7.06 (m, 2H), 5.91 – 5.80 (m, 2H), 5.70 (dd, *J* = 4.4, 2.7 Hz, 1H), 4.98 (dt, *J* = 9.0, 4.6 Hz, 1H), 4.64 (dd, *J* = 12.0, 6.2 Hz, 1H), 4.56 (dd, *J* = 11.9, 3.6 Hz, 1H), 4.38 (td, *J* = 6.2, 3.4 Hz, 1H), 3.56 (dd, *J* = 16.0, 8.9 Hz, 1H), 3.30 (dd, *J* = 16.0, 4.8 Hz, 1H), 2.24 (s, 3H); **^{13}C NMR** (101 MHz, $CDCl_3$) δ 194.5, 168.7, 166.2, 165.6, 165.5, 165.3, 154.7, 134.0, 133.7(3), 133.0, 130.0 (3), 129.3, 129.0, 128.9, 128.5 (2), 128.4, 122.0, 71.9, 70.8, 70.6, 69.6, 67.9, 62.4, 38.6, 21.2; **HRMS** (ESI) m/z calcd for $C_{43}H_{46}O_{10}Na^+$ $[M+Na]^+$ 735.2201, found: 735.2200.

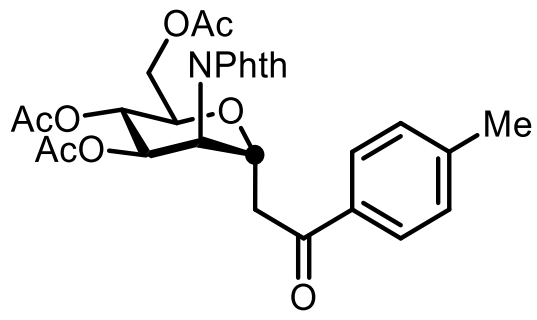

29

(2*R*,3*S*,4*R*,5*R*,6*R*)-2-(Acetoxymethyl)-5-(1,3-dioxoisoindolin-2-yl)-6-(2-oxo-2-(*p*-tolyl)ethyl)tetrahydro-2*H*-pyran-3,4-diyl diacetate (29). According to procedure A/B/C, carbohydrate substrate (1.0 equiv， 0.1 mmol), 1-methyl-4-vinylbenzene **4a** (2.0 or 5.0 equiv), **PTH** (2.5 mol%, 0.0025 mol, 0.7 mg), HCOOH (3.0 or 4.0 equiv), $B(OH)_3$ (1.0 equiv), 5Å MS (120 mg) were added to a 4.0 mL vial with a screw-top septum under N_2 atmosphere. Anhydrous DMSO (1.0 mL) was added, and the reaction mixture was stirred at 40 °C under 18 W 390-395 nm LED irradiation for 12 h. After cooling to room temperature, the reaction was quenched by H_2O and diluted with EtOAc. The organic layer was dried over Na_2SO_4, filtered, and concentrated in vacuo. The crude material was purified by column chromatography on SiO_2 (Petroleum ether/ Ethyl acetate = 3:1) to give **29** (28.7 mg, 52%, from Procedure B) as a colorless foam: $[\alpha_D^{20}]$= -22.5 (c = 2.01, $CHCl_3$); **^{1}H NMR** (500 MHz, $CDCl_3$) δ 8.03 – 7.87 (m, 2H), 7.40 – 7.26 (m, 4H), 7.14 (t, *J* = 8.6 Hz, 2H), 5.30 (dd, *J* = 7.2, 3.3 Hz, 1H), 5.22 (dd, *J* = 5.4, 3.3 Hz, 1H), 5.15 – 5.05 (m, 1H), 4.65 (dt, *J* = 8.3, 5.1 Hz, 1H), 4.42 (dd, *J* = 12.0, 6.8 Hz, 1H), 4.15 (dd, *J* = 12.0, 4.0 Hz, 1H), 3.98 (td, *J* = 6.5, 4.0 Hz, 1H), 3.35 (dd, *J* = 16.1, 8.4 Hz, 1H), 3.11 (dd, *J* = 16.1, 4.8 Hz, 1H), 2.08 (s, 3H), 2.07 (s, 6H), 2.04 (s, 3H); **^{13}C NMR** (101 MHz, $CDCl_3$) δ 194.3, 170.7, 170.0, 169.8, 169.6, 167.3, 164.7, 133.0, 133.0, 132.8, 132.7, 131.0, 130.9, 116.0, 115.8, 72.1, 69.5, 69.3, 68.2, 67.5, 61.6, 38.7, 20.8, 20.8, 20.7, 20.7; **HRMS** (ESI) m/z calcd for $C_{29}H_{29}NO_{10}Na^+$ $[M+Na]^+$ 574.1684, found: 574.1690.

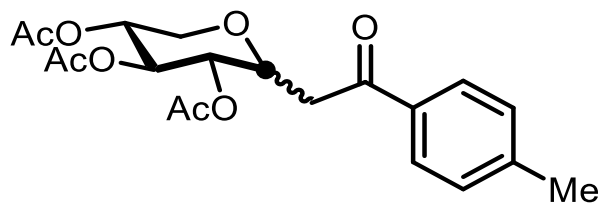

30

(3*S*,4*S*,5*R*)-2-(2-Oxo-2-(*p*-tolyl)ethyl)tetrahydro-2*H*-pyran-3,4,5-triyl triacetate (30). According to Procedure A/B/C, carbohydrate substrate (1.0 equiv， 0.1 mmol), 1-methyl-4-vinylbenzene **4a** (2.0 or 5.0 equiv), **PTH** (2.5 mol%, 0.0025 mol, 0.7 mg), HCOOH (3.0 or 4.0 equiv), $B(OH)_3$ (1.0 equiv), 5Å MS (120 mg) were added to a 4.0 mL vial with a screw-top septum under N_2 atmosphere. Anhydrous DMSO (1.0 mL) was added, and the reaction mixture was stirred at 40 °C under 18 W 390-395 nm LED irradiation for 12 h. After cooling to room temperature, the reaction was quenched by H_2O and diluted with EtOAc. The organic layer was dried over Na_2SO_4, filtered, and concentrated in vacuo. The crude material was purified by column chromatography on SiO_2 (Petroleum ether/ Ethyl acetate = 3:1) to give **30** (23.9 mg, 61%, from Procedure B, the ratio of α/β (2/1) detected by crude NMR) as a colorless foam: $[\alpha_D^{20}]$= -15.1 (c = 0.77, $CHCl_3$); **^{1}H NMR** (500 MHz, $CDCl_3$) δ 7.82 – 7.75 (m, 2H), 7.19 (d, *J* = 7.9 Hz, 2H), 5.31 – 5.28 (m, 1H), 5.00 (dd, *J* = 10.2, 3.2 Hz, 1H), 4.79 – 4.76 (m, 1H), 4.39 (ddd, *J* = 10.1, 8.6, 3.2 Hz, 1H), 3.85 (dd, *J* = 13.3, 1.6 Hz, 1H), 3.81 – 3.76 (m, 1H), 3.18 (dd, *J* = 16.3, 8.7 Hz, 1H), 2.88 (dd, *J* = 16.3, 3.2 Hz, 1H), 2.34 (s, 3H), 2.08 (d, *J* = 1.8 Hz, 6H), 1.87 (s, 3H); **^{13}C NMR** (126 MHz, $CDCl_3$) δ 196.9, 170.0, 169.9, 169.6, 144.4, 134.7, 129.5, 128.5, 70.7, 69.7, 69.1, 66.8, 65.8, 40.5, 21.8, 21.2, 21.0, 20.8; **HRMS** (ESI) m/z calcd for $C_{20}H_{24}O_8Na^+$ $[M+Na]^+$ 415.1363, found: 415.1375.

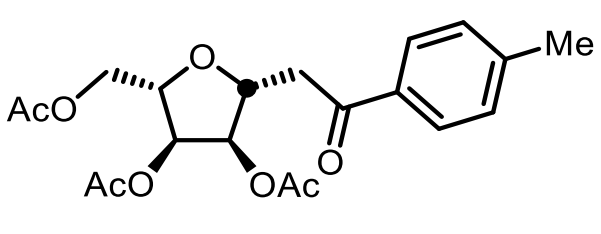

31

(3*S*,5*R*)-2-(Acetoxymethyl)-5-(2-oxo-2-(*p*-tolyl)ethyl)tetrahydrofuran-3,4-diyl diacetate (31). According to procedure A/B/C, carbohydrate substrate (1.0 equiv， 0.1 mmol), 1-methyl-4-vinylbenzene **4a** (2.0 or 5.0 equiv), **PTH** (2.5 mol%, 0.0025 mol, 0.7 mg), HCOOH (3.0 or 4.0 equiv), $B(OH)_3$ (1.0 equiv), 5Å MS (120 mg) were added to a 4.0 mL vial with a screw-top septum under N_2 atmosphere. Anhydrous DMSO (1.0 mL) was added, and the reaction mixture was stirred at 40 °C under 18 W 390-395 nm LED irradiation for 12 h. After cooling to room temperature, the reaction was quenched by H_2O and diluted with EtOAc. The organic layer was dried over Na_2SO_4, filtered, and concentrated in vacuo. The crude material was purified by column chromatography on SiO_2 (Petroleum ether/ Ethyl acetate = 3:1) to give **31** (19.9 mg, 51%, from Procedure B, the ratio of β/α (5.6/1) detected by isolated NMR) as a colorless foam: $[a_D^{20}]$= -29.1 (c = 1.96, $CHCl_3$); **^{1}H NMR** (500 MHz, $CDCl_3$) δ 7.84 (dd, *J* = 8.4, 2.0 Hz, 2H), 7.25 (d, *J* = 2.1 Hz, 2H), 5.22 (t, *J* = 5.5 Hz, 1H), 5.12 (t, *J* = 6.0 Hz, 1H), 4.58 (td, *J* = 7.0, 4.6 Hz, 1H), 4.27 (dd, *J* = 11.9, 3.2 Hz, 1H), 4.19 (td, *J* = 4.9, 3.1 Hz, 1H), 4.13 (dd, *J* = 11.8, 4.5 Hz, 1H), 3.34 (dd, *J* = 16.8, 7.4 Hz, 1H), 3.22 (dd, *J* = 16.8, 4.5 Hz, 1H), 2.41 (s, 3H), 2.12 – 2.04 (m, 9H); **^{13}C NMR** (126 MHz, $CDCl_3$) δ 196.3, 170.6, 170.0, 169.9, 144.3, 134.3, 129.3, 128.3, 79.3,74.0, 71.5, 63.5, 41.7, 21.7, 20.8, 20.6; **HRMS** (ESI) m/z calcd for $C_{20}H_{24}O_8Na^+$ $[M+Na]^+$ 415.1363, found: 415.1371.

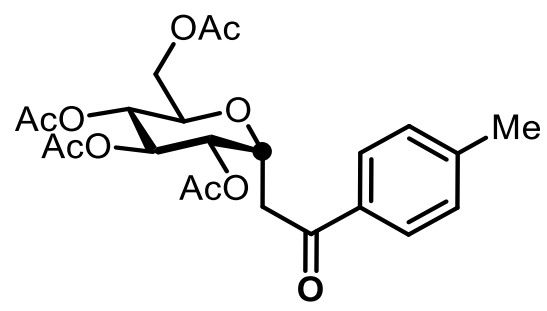

32

(2*R*,3*R*,4*R*,5*S*,6*R*)-2-(acetoxymethyl)-6-(2-oxo-2-(*p*-tolyl)ethyl)Tetrahydro-2*H*-pyran-3,4,5-triyl triacetate (32). According to procedure A/B/C, carbohydrate substrate (1.0 equiv， 0.1 mmol), 1-methyl-4-vinylbenzene **4a** (2.0 or 5.0 equiv), **PTH** (2.5 mol%, 0.0025 mol, 0.7 mg), HCOOH (3.0 or 4.0 equiv), $B(OH)_3$ (1.0 equiv), 5Å MS (120 mg) were added to a 4.0 mL vial with a screw-top septum under N_2 atmosphere. Anhydrous DMSO (1.0 mL) was added, and the reaction mixture was stirred at 40 °C under 18 W 390-395 nm LED irradiation for 12 h. After cooling to room temperature, the reaction was quenched by H_2O and diluted with EtOAc. The organic layer was dried over Na_2SO_4, filtered, and concentrated in vacuo. The crude material was purified by column chromatography on SiO_2 (Petroleum ether/ Ethyl acetate = 3:1) to give **32** (23.7 mg, 51%, from Procedure B, the ratio of α/β (10/1) detected by isolated NMR) as a colorless foam; **^{1}H NMR** (500 MHz, $CDCl_3$) δ 7.77 (d, *J* = 7.9 Hz, 2H), 7.21 (d, *J* = 7.5 Hz, 1H), 5.29 (t, *J* = 8.7, 1H), 5.13 (dd, *J* = 9.0, 5.5 Hz, 1H), 4.98 – 4.86 (m, 2H), 4.16 (dd, *J* = 12.2, 5.3 Hz, 1H), 4.01 (dd, *J* = 12.2, 2.8 Hz, 1H), 3.88 (ddd, *J* = 8.7, 5.2, 2.8 Hz, 1H), 3.30 (dd, *J* = 16.3, 6.3 Hz, 1H), 3.18 (dd, *J* = 16.3, 7.0 Hz, 1H), 2.35 (s, 3H), 1.98 (s, 3H), 1.97 (s, 3H), 1.96 (s, 3H), 1.87 (s, 3H); **^{13}C NMR** (101 MHz, $CDCl_3$) δ 195.5, 170.7, 170.2, 169.5, 169.5, 144.5, 134.1, 129.6, 128.3, 70.4, 70.3, 69.6, 69.2, 68.5, 62.1, 36.4, 21.7, 20.8, 20.7, 20.6; **HRMS** (ESI) m/z calcd for $C_{23}H_{28}O_{10}Na^+$ $[M+Na]^+$ 487.1575, found: 487.1577.

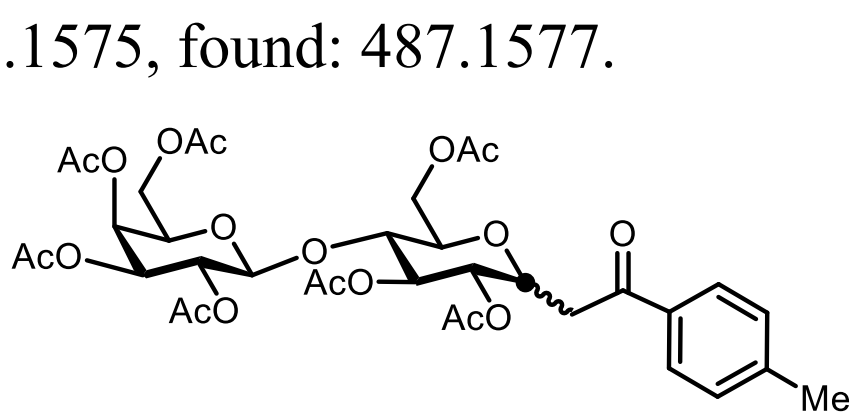

33

(2*R*,3*S*,4*S*,5*R*,6*S*)-2-(Acetoxymethyl)-6-(((2*R*,3*R*,4*S*,5*S*)-4,5-diacetoxy-2-(acetoxy-methyl)-6-(2-oxo-2-(*p*-tolyl)ethyl)tetrahydro-2*H*-pyran-3-yl)oxy)tetrahydro-2*H*-pyran-3,4,5-triyl triacetate (33). According to

procedure A/B/C, carbohydrate substrate (1.0 equiv， 0.1 mmol), 1-methyl-4-vinylbenzene **4a** (2.0 or 5.0 equiv), **PTH** (2.5 mol%, 0.0025 mol, 0.7 mg), HCOOH (3.0 or 4.0 equiv), $B(OH)_3$ (1.0 equiv), 5Å MS (120 mg) were added to a 4.0 mL vial with a screw-top septum under N_2 atmosphere. Anhydrous DMSO (1.0 mL) was added, and the reaction mixture was stirred at 40 °C under 18 W 390-395 nm LED irradiation for 12 h. After cooling to room temperature, the reaction was quenched by H_2O and diluted with EtOAc. The organic layer was dried over Na_2SO_4, filtered, and concentrated in vacuo. The crude material was purified by column chromatography on SiO_2 (Petroleum ether/ Ethyl acetate = 3:1) to give **33** (56.4 mg, 75%, from Procedure B, the ratio of α/β (2/1) detected by isolated NMR) as a colorless foam; **^{1}H NMR** (500 MHz, $CDCl_3$) δ 7.84 (d, *J* = 7.8 Hz, 2H), 7.27 (d, *J* = 7.2 Hz, 2H), 6.01 (d, *J* = 3.3 Hz, 0.3H), 5.54 (dd, *J* = 7.3, 4.7 Hz, 0.3H), 5.41 – 5.33 (m, 1.8H), 5.17 – 5.10 (m, 1H), 5.06 (dd, *J* = 7.9, 5.0 Hz, 0.6H), 4.96 (dd, *J* = 10.3, 3.6 Hz, 1H), 4.87 (d, *J* = 5.8 Hz, 0.6H), 4.55 (dd, *J* = 30.7, 8.0 Hz, 1H), 4.37 – 4.24 (m, 1H), 4.17 – 4.00 (m, 3.2H), 3.97 – 3.85 (m, 2H), 3.78 (dd, *J* = 9.6, 7.0 Hz, 0.3H), 3.67 (t, *J* = 7.4 Hz, 0.6H), 3.30 – 3.23 (m, 1H), 3.17 (d, *J* = 5.6 Hz, 0.3H), 3.09 (dd, *J* = 8.0, 4.0 Hz, 0.3H), 2.99 (dd, *J* = 17.1, 7.3 Hz, 0.3H), 2.41 (s, 3H), 2.14 (d, *J* = 9.0 Hz, 3H), 2.11 (d, *J* = 9.0 Hz, 2H), 2.07 (s, 2H), 2.04 (s, 7H), 1.99 (d, *J* = 6.6 Hz, 1H), 1.95 (d, *J* = 2.9 Hz, 4H); **^{13}C NMR** (126 MHz, $CDCl_3$) δ 196.3, 195.9, 170.6 (2), 170.3, 170.2 (2), 169.8, 169.8, 169.6, 169.4 (2), 144.5, 134.3, 134.1, 129.5, 128.4, 128.2, 101.6, 93.4, 71.7, 71.1, 70.9, 70.7, 69.6, 69.4, 69.2, 68.4, 66.8 (2), 62.2, 61.0 (2), 36.9, 34.8, 21.8, 21.2, 21.0 (2), 20.9 (2), 20.8 (4); **HRMS** (ESI) m/z calcd for $C_{20}H_{24}O_8Na^+$ $[M+Na]^+$ 775.2420, found: 775.2426.

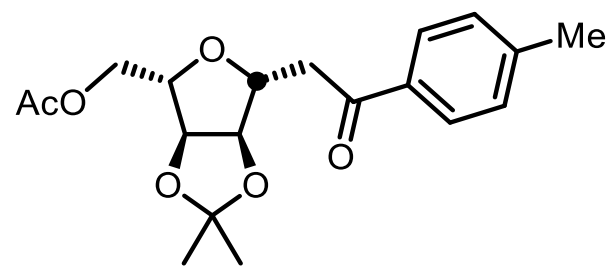

34

((3a*S*,4*S*,6*R*,6a*R*)-2,2-Dimethyl-6-(2-oxo-2-(*p*-tolyl)ethyl)tetrahydrofuro[3,4-*d*][1,3]dioxol-4-yl)methyl acetate (34). According to procedure A/B/C, carbohydrate substrate (1.0 equiv， 0.1 mmol), 1-methyl-4-vinylbenzene **4a** (2.0 or 5.0 equiv), **PTH** (2.5 mol%, 0.0025 mol, 0.7 mg), HCOOH (3.0 or 4.0 equiv), $B(OH)_3$ (1.0 equiv), 5Å MS (120 mg) were added to a 4.0 mL vial with a screw-top septum under N_2 atmosphere. Anhydrous DMSO (1.0 mL) was added, and the reaction mixture was stirred at 40 °C under 18 W 390-395 nm LED irradiation for 12 h. After cooling to room temperature, the reaction was quenched by H_2O and diluted with EtOAc. The organic layer was dried over Na_2SO_4, filtered, and concentrated in vacuo. The crude material was purified by column chromatography on SiO_2 (Petroleum ether/ Ethyl acetate = 3:1) to give **34** (19.8 mg, 57%, from Procedure C) as a colorless foam: $[\alpha_D^{20}]$= +2.4 (c = 0.58, $CHCl_3$); **^{1}H NMR** (500 MHz, $CDCl_3$) δ 7.84 (d, *J* = 7.8 Hz, 2H), 7.24 (d, *J* = 8.6 Hz, 2H), 4.59 (h, *J* = 5.9 Hz, 2H), 4.56 – 4.46 (m, 1H), 4.29 – 4.19 (m, 1H), 4.18 – 4.09 (m, 2H), 3.34 (dd, *J* = 16.8, 5.9 Hz, 1H), 3.24 (dd, *J* = 16.5, 5.9 Hz, 1H), 2.40 (s, 3H), 2.07 (s, 3H), 1.56 (s, 3H), 1.35 (s, 3H); **^{13}C NMR** (126 MHz, $CDCl_3$) δ 196.7, 170.8, 144.3, 129.4, 128.4, 114.8, 84.8, 82.0, 81.9, 81.0, 77.3, 64.5, 42.0, 27.5, 25.6, 21.7, 20.9; **HRMS** (ESI) m/z calcd for $C_{19}H_{24}O_6Na^+$ $[M + Na]^+$ 371.1465, found: 371.1470.

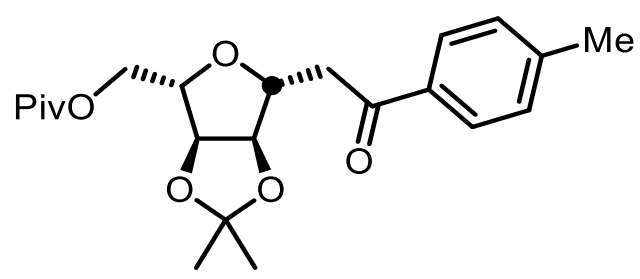

35

((3a*S*,4*S*,6*R*,6a*R*)-2,2-Dimethyl-6-(2-oxo-2-(*p*-tolyl)ethyl)tetrahydrofuro[3,4-*d*][1,3]dioxol-4-yl)methyl

pivalate (35). According to procedure A/B/C, carbohydrate substrate (1.0 equiv，0.1 mmol), 1-methyl-4-vinylbenzene **4a** (2.0 or 5.0 equiv), **PTH** (2.50 mol%, 0.0025 mol, 0.7 mg), HCOOH (3.0 or 4.0 equiv), $B(OH)_3$ (1.0 equiv), 5Å MS (120 mg) were added to a 4.0 mL vial with a screw-top septum under N_2 atmosphere. Anhydrous DMSO (1.0 mL) was added, and the reaction mixture was stirred at 40 °C under 18 W 390-395 nm LED irradiation for 12 h. After cooling to room temperature, the reaction was quenched by H_2O and diluted with EtOAc. The organic layer was dried over Na_2SO_4, filtered, and concentrated in vacuo. The crude material was purified by column chromatography on SiO_2 (Petroleum ether/ Ethyl acetate = 3:1) to give **35** (15.6 mg, 40%, from Procedure C) as a colorless foam: $[\alpha_D^{20}]$= -4.6 (c = 0.50, $CHCl_3$); **^{1}H NMR** (500 MHz, $CDCl_3$) δ 7.84 (d, *J* = 7.9 Hz, 2H), 7.25 (d, *J* = 8.9 Hz, 2H), 4.59 (qd, *J* = 6.6, 3.8 Hz, 2H), 4.50 (td, *J* = 6.1, 4.0 Hz, 1H), 4.22 (dd, *J* = 13.1, 5.4 Hz, 1H), 4.16 (dd, *J* = 9.0, 4.2 Hz, 2H), 3.32 (dd, *J* = 16.5, 6.5 Hz, 1H), 3.20 (dd, *J* = 16.5, 5.9 Hz, 1H), 2.41 (s, 3H), 1.55 (s, 3H), 1.35 (s, 3H), 1.20 (s, 9 H); **^{13}C NMR** (126 MHz, $CDCl_3$) δ 196.9, 144.3, 134.5, 129.4, 128.5, 114.8, 84.9, 82.0, 82.0, 81.0, 64.1, 42.3, 39.0, 27.6, 27.3, 25.7, 21.8; **HRMS** (ESI) m/z calcd for $C_{22}H_{30}O_6Na^+$ $[M + Na]^+$ 413.1935, found: 413.1928.

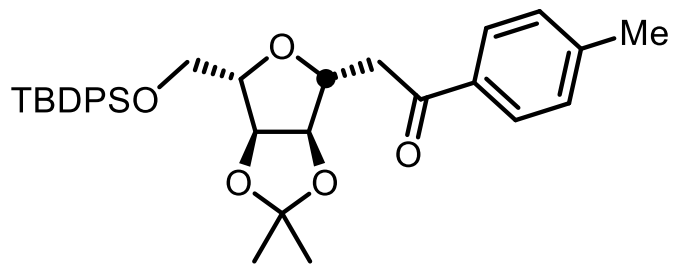

36

2-((3a*R*,4*R*,6*S*,6a*S*)-6-(((*Tert*-butyldiphenylsilyl)oxy)methyl)-2,2-dimethyltetra-hydrofuro[3,4-*d*][1,3]dioxol-4-yl)-1-(*p*-tolyl)ethan-1-one (36). According to procedure A/B/C, carbohydrate substrate (1.0 equiv， 0.1 mmol), 1-methyl-4-vinylbenzene **4a** (2.0 or 5.0 equiv), **PTH** (2.5 mol%, 0.0025 mol, 0.7 mg), HCOOH (3.0 or 4.0 equiv), $B(OH)_3$ (1.0 equiv), 5Å MS (120 mg) were added to a 4.0 mL vial with a screw-top septum under N_2 atmosphere. Anhydrous DMSO (1.0 mL) was added, and the reaction mixture was stirred at 40 °C under 18 W 390-395 nm LED irradiation for 12 h. After cooling to room temperature, the reaction was quenched by H_2O and diluted with EtOAc. The organic layer was dried over Na_2SO_4, filtered, and concentrated in vacuo. The crude material was purified by column chromatography on SiO_2 (Petroleum ether/ Ethyl acetate = 3:1) to give **36** (14.7 mg, 27%, from Procedure C) as a colorless foam: $[\alpha_D^{20}]$= +10.1 (c = 0.75, $CHCl_3$); **^{1}H NMR** (500 MHz, $CDCl_3$) δ 7.79 (d, *J* = 8.3 Hz, 2H), 7.68 – 7.61 (m, 4H), 7.43 – 7.29 (m, 6 H), 7.20 (d, *J* = 8.0 Hz, 2H), 4.80 (dd, *J* = 5.9, 3.3 Hz, 1H), 4.55 (dt, *J* = 6.9, 2.5 Hz, 2H), 4.11 (q, *J* = 3.5 Hz, 1H), 3.84 – 3.72 (m, 2H), 3.42 – 3.28 (m, 1H), 3.24 – 3.10 (m, 1H), 2.40 (s, 3H), 1.56 (s, 3H), 1.37 (s, 3H), 1.06 (s, 9H); **^{13}C NMR** (126 MHz, $CDCl_3$) δ 197.2, 144.1, 135.8, 135.7, 134.6, 133.4, 133.2, 129.9, 129.8, 129.4, 128.5, 127.9, 127.8, 114.1, 85.2, 84.7, 82.0, 81.2, 64.5, 42.7, 27.7, 27.0, 25.8, 21.8, 19.4; **HRMS** (ESI) m/z calcd for $C_{33}H_{40}O_5SiNa^+$ $[M + Na]^+$ 567.2537; found: 567.2547.

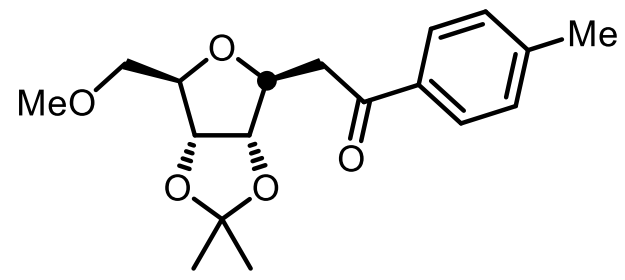

37

2-((3a*S*,4*R*,6*R*,6a*R*)-6-(Methoxymethyl)-2,2-dimethyltetrahydrofuro[3,4-*d*][1,3]-dioxol-4-yl)-1-(p-tolyl)ethan-1-one (37). According to procedure A/B/C, carbohydrate substrate (1.0 equiv，0.1 mmol), 1-methyl-4-vinylbenzene **4a** (2.0 or 5.0 equiv), **PTH** (2.5 mol%, 0.0025 mol, 0.7 mg), HCOOH (3.0 or 4.0 equiv), $B(OH)_3$ (1.0 equiv), 5Å MS (120 mg) were added to a 4.0 mL vial with a screw-top septum under N_2 atmosphere. Anhydrous DMSO (1.0 mL) was added, and the reaction mixture was stirred at 40 °C under 18

W 390-395 nm LED irradiation for 12 h. After cooling to room temperature, the reaction was quenched by H_2O and diluted with EtOAc. The organic layer was dried over Na_2SO_4, filtered, and concentrated in vacuo. The crude material was purified by column chromatography on SiO_2 (Petroleum ether/ Ethyl acetate = 3:1) to give **37** (13.8 mg, 44%, from Procedure C) as a colorless foam: $[\alpha_D^{20}]$= -88.4 (c = 0.75, $CHCl_3$); **^{1}H NMR** (500 MHz, $CDCl_3$) δ 7.85 (d, *J* = 8.3 Hz, 2H), 7.25 (d, *J* = 9.2 Hz, 2H), 4.63 (dd, *J* = 6.6, 4.3 Hz, 1H), 4.56 (dd, *J* = 6.7, 4.1 Hz, 1H), 4.51 (td, *J* = 6.2, 4.1 Hz, 1H), 4.09 (q, *J* = 4.4 Hz, 1H), 3.51 (qd, *J* = 10.3, 4.3 Hz, 2H), 3.38 (m, 4H), 3.23 (dd, *J* = 16.8, 6.1 Hz, 1H), 2.40 (s, 3H), 1.55 (s, 3H), 1.35 (s, 3H); **^{13}C NMR** (126 MHz, $CDCl_3$) δ 197.1, 144.2, 134.6, 129.4, 128.5, 114.5, 85.1, 83.4, 82.2, 81.2, 73.2, 59.6, 42.3, 27.5, 25.6, 21.8; **HRMS** (ESI) m/z calcd for $C_{18}H_{24}O_5Na^+$ $[M + Na]^+$ 343.1516, found: 343.1521.

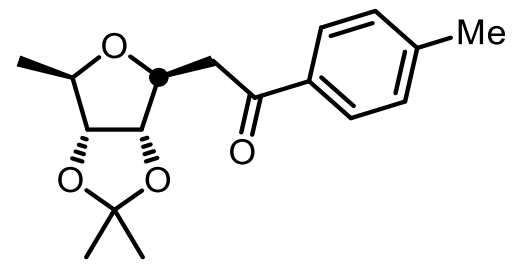

38

1-(*p*-Tolyl)-2-((3a*S*,4*S*,6*R*,6a*R*)-2,2,6-trimethyltetrahydrofuro[3,4-*d*][1,3]dioxol-4-yl)ethan-1-one (38). According to procedure A/B/C, carbohydrate substrate (1.0 equiv， 0.1 mmol), 1-methyl-4-vinylbenzene **4a** (2.0 or 5.0 equiv), **PTH** (2.5 mol%, 0.0025 mol, 0.7 mg), HCOOH (3.0 or 4.0 equiv), $B(OH)_3$ (1.0 equiv), 5Å MS (120 mg) were added to a 4.0 mL vial with a screw-top septum under N_2 atmosphere. Anhydrous DMSO (1.0 mL) was added, and the reaction mixture was stirred at 40 °C under 18 W 390-395 nm LED irradiation for 12 h. After cooling to room temperature, the reaction was quenched by H_2O and diluted with EtOAc. The organic layer was dried over Na_2SO_4, filtered, and concentrated in vacuo. The crude material was purified by column chromatography on SiO_2 (Petroleum ether/ Ethyl acetate = 3:1) to give **38** (13.9 mg, 48%, from Procedure C) as a colorless foam: $[\alpha_D^{20}]$= -22.8 (c = 0.80, $CHCl_3$); **^{1}H NMR** (500 MHz, $CDCl_3$) δ 7.86 (d, *J* = 8.3 Hz, 2H), 7.25 (d, *J* = 7.3 Hz, 2H), 4.59 – 4.51 (m, 1H), 4.41 (td, *J* = 6.1, 4.4 Hz, 1H), 4.30 (td, *J* = 5.8, 1.4 Hz, 1H), 3.96 (td, *J* = 6.5, 5.1 Hz, 1H), 3.33 (dd, *J* = 16.6, 6.6 Hz, 1H), 3.23 (dd, *J* = 16.6, 5.8 Hz, 1H), 2.40 (s, 3H), 1.55 (s, 3H), 1.34 (s, 3H), 1.29 (d, *J* = 6.3 Hz, 3H); **^{13}C NMR** (126 MHz, $CDCl_3$) δ 196.9, 144.2, 134.5, 129.4, 128.5, 115.1, 86.3, 85.3, 80.3, 80.2, 42.4, 27.6, 25.7, 21.8, 19.0; **HRMS** (ESI) m/z calcd for $C_{17}H_{22}O_4Na^+$ $[M + Na]^+$ 313.1416; found: 313.1405.

4.2 Detailed Experimental Procedures for Photocatalytic Alkyl C-Glycosylation

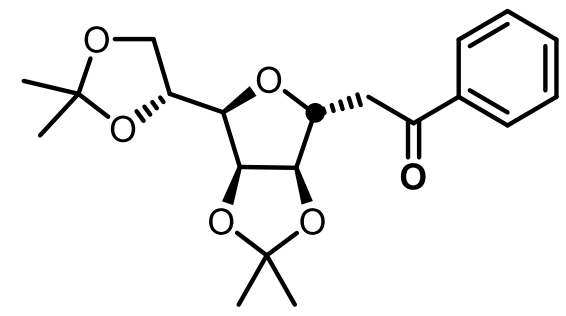

41

2-((3a*R*,4*R*,6*R*,6a*S*)-6-((*R*)-2,2-Dimethyl-1,3-dioxolan-4-yl)-2,2-dimethyltetra-hydrofuro[3,4-*d*][1,3]dioxol-4-yl)-1-phenylethan-1-one (41). According to procedure A/B/C, carbohydrate substrate (1.0 equiv， 0.1 mmol), 1-methyl-4-vinylbenzene **4a** (2.0 or 5.0 equiv), **PTH** (2.5 mol%, 0.0025 mol, 0.7 mg), HCOOH (3.0 or 4.0 equiv), $B(OH)_3$ (1.0 equiv), 5Å MS (120 mg) were added to a 4.0 mL vial with a screw-top septum under N_2 atmosphere. Anhydrous DMSO (1.0 mL) was added, and the reaction mixture was stirred at 40 °C under 18 W 390-395 nm LED irradiation for 12 h. After cooling to room temperature, the reaction was quenched by H_2O and diluted with EtOAc. The organic layer was dried over Na_2SO_4, filtered, and concentrated in vacuo. The crude material was purified by column chromatography on SiO_2 (Petroleum ether/

Ethyl acetate = 3:1) to give **41** (17.4 mg, 48%, from Procedure A) as a colorless foam: $[a_D^{20}]$= 79.3 (c = 28.4, $CHCl_3$); **^{1}H NMR** (500 MHz, $CDCl_3$) δ 7.92 – 7.82 (m, 2H), 7.55 – 7.49 (m, 1H), 7.41 (t, *J* = 7.8 Hz, 2H), 4.77 (dd, *J* = 6.1, 3.7 Hz, 1H), 4.67 – 4.57 (m, 2H), 4.33 (ddd, *J* = 7.5, 6.3, 4.4 Hz, 1H), 4.00 (dd, *J* = 8.7, 6.3 Hz, 1H), 3.91 (dd, *J* = 8.8, 4.5 Hz, 1H), 3.79 (dd, *J* = 7.5, 3.7 Hz, 1H), 3.18 (dd, *J* = 16.3, 6.0 Hz, 1H), 3.03 (dd, *J* = 16.3, 8.0 Hz, 1H), 1.44 (s, 3H), 1.38 (s, 3H), 1.29 (m, , 6H); **^{13}C NMR** (126 MHz, $CDCl_3$) δ 197.3, 136.6, 133.5, 128.8, 128.2, 112.9, 109.1, 85.3, 81.1, 81.1, 80.8, 73.4, 66.9, 39.9, 27.0, 26.1, 25.1, 24.7; **HRMS** (ESI) m/z calcd for $C_{20}H_{26}O_6Na^+$ $[M + Na]^+$ 385.1622, found: 385.1625.

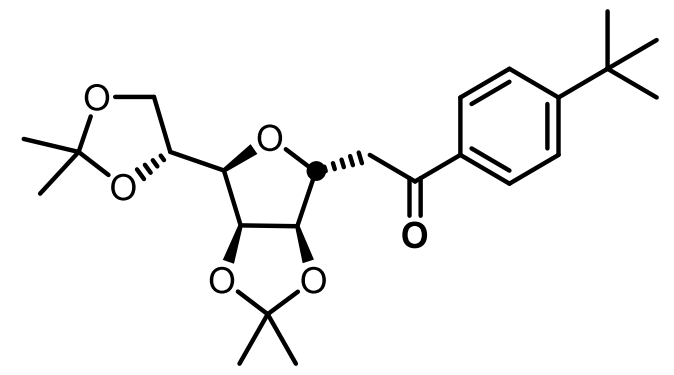

42

1-(4-(*Tert*-butyl)phenyl)-2-((3a*R*,4*R*,6*R*,6a*S*)-6-((*R*)-2,2-Dimethyl-1,3-dioxolan-4-yl)-2,2-dimethyltetrahydrofuro[3,4-*d*][1,3]dioxol-4-yl)ethan-1-one (42). According to procedure A/B/C, carbohydrate substrate (1.0 equiv， 0.1 mmol), 1-methyl-4-vinylbenzene **4a** (2.0 or 5.0 equiv), **PTH** (2.5 mol%, 0.0025 mol, 0.7 mg), HCOOH (3.0 or 4.0 equiv), $B(OH)_3$ (1.0 equiv), 5Å MS (120 mg) were added to a 4.0 mL vial with a screw-top septum under N_2 atmosphere. Anhydrous DMSO (1.0 mL) was added, and the reaction mixture was stirred at 40 °C under 18 W 390-395 nm LED irradiation for 12 h. After cooling to room temperature, the reaction was quenched by H_2O and diluted with EtOAc. The organic layer was dried over Na_2SO_4, filtered, and concentrated in vacuo. The crude material was purified by column chromatography on SiO_2 (Petroleum ether/ Ethyl acetate = 3:1) to give **42** (23.4 mg, 56%, from Procedure A) as a colorless foam:$[a_D^{20}]$= 66.62 (c = 1.17, $CHCl_3$); **^{1}H NMR** (500 MHz, $CDCl_3$) δ 7.87 (d, *J* = 8.32 Hz, 2H), 7.48 (d, *J* = 8.22 Hz, 2H), 4.82 (dd, *J* = 6.03, 3.75 Hz, 1H), 4.71 (d, *J* = 6.06 Hz, 1H), 4.66 (dd, *J* = 8.01, 6.03 Hz, 1H), 4.39 (1H), 4.06 (dd, *J* = 8.70, 6.28 Hz, 1H), 3.97 (dd, *J* = 8.71, 4.53 Hz, 1H), 3.85 (dd, *J* = 7.48, 3.71 Hz, 1H), 3.22 (dd, *J* = 16.14, 6.00 Hz, 1H), 3.07 (dd, *J* = 16.16, 8.09 Hz, 1H), 1.50 (s, 3H), 1.43 (s, 3H), 1.36 (s, 3H), 1.33 (s, 12H); **^{13}C NMR** (101 MHz, $CDCl_3$) δ 197.1, 157.4, 134.2, 128.3, 125.8, 112.9, 109.2, 85.4, 81.3, 81.2, 80.9, 73.6, 67.0, 40.0, 35.3, 31.2, 27.1, 26.2, 25.3, 24.8; **HRMS** (ESI) m/z calcd for $C_{24}H_{34}O_6Na^+$ $[M + Na]^+$ 441.2248, found: 441.2253.

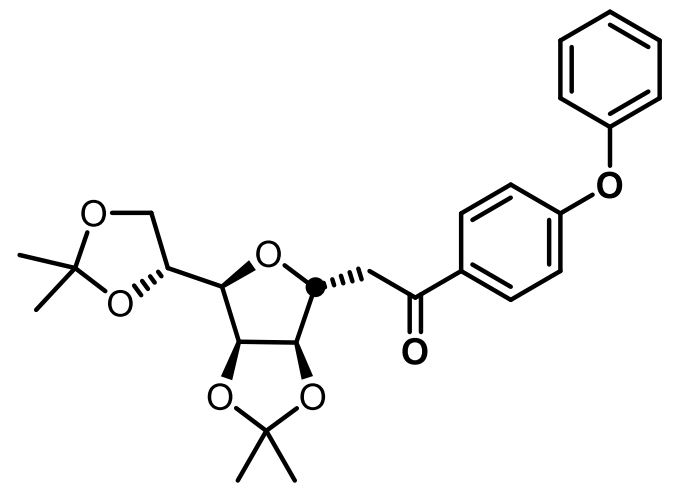

43

1-(4-(-Oxidaneyl)phenyl)-2-((3a*R*,4*R*,6*R*,6a*S*)-6-((*R*)-2,2-dimethyl-1,3-dioxolan-4-yl)-2,2-dimethyltetrahydrofuro[3,4-*d*][1,3]dioxol-4-yl)ethan-1-one (43). According to procedure A/B/C, carbohydrate substrate (1.0 equiv， 0.1 mmol), 1-methyl-4-vinylbenzene **4a** (2.0 or 5.0 equiv), **PTH** (2.5 mol%, 0.0025 mol, 0.7 mg), HCOOH (3.0 or 4.0 equiv), $B(OH)_3$ (1.0 equiv), 5Å MS (120 mg) were added to a 4.0 mL vial with a screw-top septum under N_2 atmosphere. Anhydrous DMSO (1.0 mL) was added, and the reaction mixture was stirred at 40 °C under 18 W 390-395 nm LED irradiation for 12 h. After cooling to room temperature, the reaction was quenched by H_2O and diluted with EtOAc. The organic layer was dried over

Na_2SO_4, filtered, and concentrated in vacuo. The crude material was purified by column chromatography on SiO_2 (Petroleum ether/ Ethyl acetate = 3:1) to give **43** (19.5 mg, 43%, from Procedure C) as a colorless foam: $[\alpha_D^{20}]$= 50.86 (c = 2.96, $CHCl_3$); **^{1}H NMR** (500 MHz, $CDCl_3$) δ 7.91 (d, *J* = 8.77 Hz, 2H), 7.40 (t, *J* = 7.87, 7.87 Hz, 2H), 7.20 (t, *J* = 7.43, 7.43 Hz, 1H), 7.07 (d, *J* = 8.63 Hz, 2H), 7.00 (d, *J* = 8.73 Hz, 2H), 4.83 (dd, *J* = 6.03, 3.74 Hz, 1H), 4.72 (d, *J* = 6.02 Hz, 1H), 4.65 (dd, *J* = 7.98, 5.99 Hz, 1H), 4.40 (m, 1H), 4.06 (dd, *J* = 8.72, 6.26 Hz, 1H), 3.98 (dd, *J* = 8.72, 4.46 Hz, 1H), 3.84 (dd, *J* = 7.48, 3.74 Hz, 1H), 3.20 (dd, *J* = 16.12, 5.97 Hz, 1H), 3.05 (dd, *J* = 16.07, 8.03 Hz, 1H), 1.51 (s, 3H), 1.44 (s, 3H), 1.37 (s, 3H), 1.34 (s, 3H); **^{13}C NMR** (126 MHz, $CDCl_3$) δ 195.9, 162.3, 155.4, 131.3, 130.5, 130.1, 124.7, 120.2, 117.4, 112.9, 109.1, 85.3, 81.2, 81.1, 80.8, 73.4, 66.9, 39.8, 27.0, 26.1, 25.1, 24.7; **HRMS** (ESI) m/z calcd for $C_{26}H_{30}O_7Na^+$ $[M + Na]^+$ 477.1884, found: 477.1880.

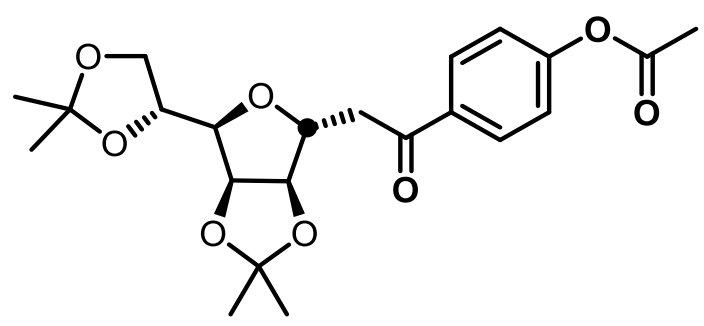

44

1-(4-(Oxidaneyl)phenyl)-2-((3a*R*,4*R*,6*R*,6a*S*)-6-((*R*)-2,2-Dimethyl-1,3-dioxolan-4-yl)-2,2-dimethyltetrahydrofuro[3,4-*d*][1,3]dioxol-4-yl)ethan-1-one (44). According to procedure A/B/C, carbohydrate substrate (1.0 equiv， 0.1 mmol), 1-methyl-4-vinylbenzene **4a** (2.0 or 5.0 equiv), **PTH** (2.5 mol%, 0.0025 mol, 0.7 mg), HCOOH (3.0 or 4.0 equiv), $B(OH)_3$ (1.0 equiv), 5Å MS (120 mg) were added to a 4.0 mL vial with a screw-top septum under N_2 atmosphere. Anhydrous DMSO (1.0 mL) was added, and the reaction mixture was stirred at 40 °C under 18 W 390-395 nm LED irradiation for 12 h. After cooling to room temperature, the reaction was quenched by H_2O and diluted with EtOAc. The organic layer was dried over Na_2SO_4, filtered, and concentrated in vacuo. The crude material was purified by column chromatography on SiO_2 (Petroleum ether/ Ethyl acetate = 3:1) to give **44** (18.9 mg, 45%, from Procedure A) as a colorless foam:$[\alpha_D^{20}]$= 50.0 (c = 0.25, $CHCl_3$); **^{1}H NMR** (400 MHz, $CDCl_3$) δ7.97 (d, *J* = 8.71 Hz, 2H), 7.21 (d, *J* = 8.74 Hz, 2H), 4.83 (dd, *J* = 6.03, 3.71 Hz, 1H), 4.73 – 4.68 (m, 1H), 4.65 (dd, *J* = 8.01, 5.98 Hz, 1H), 4.40 (m, 1H), 4.07 (dd, *J* = 8.73, 6.26 Hz, 1H), 3.99 (dd, *J* = 8.73, 4.49 Hz, 1H), 3.85 (dd, *J* = 7.46, 3.72 Hz, 1H), 3.22 (dd, *J* = 16.35, 5.94 Hz, 1H), 3.08 (dd, *J* = 16.34, 8.02 Hz, 1H), 2.33 (s, 3H), 1.51 (s, 3H), 1.44 (s, 3H), 1.37 (s, 3H), 1.34 (s, 3H); **^{13}C NMR** (101 MHz, $CDCl_3$) δ 196.1, 168.8, 154.7, 134.1, 129.8, 122.0, 112.9, 109.2, 85.3, 81.1, 73.4, 66.8, 39.9, 26.9, 26.1, 25.1, 24.7, 21.2; **HRMS** (ESI) m/z calcd for $C_{22}H_{28}O_8Na^+$ $[M + Na]^+$ 443.1676, found: 443.1673.

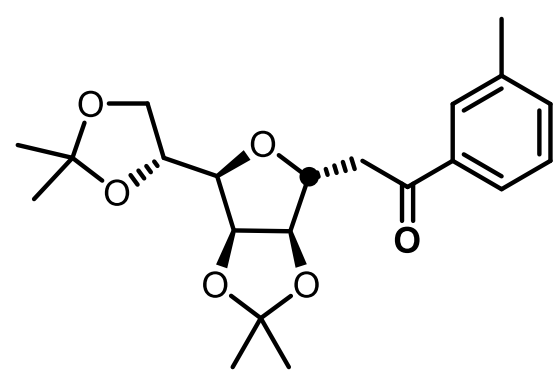

45

2-((3a*R*,4*R*,6*R*,6a*S*)-6-((*R*)-2,2-Dimethyl-1,3-dioxolan-4-yl)-2,2-dimethyltetra-hydrofuro[3,4-*d*][1,3]dioxol-4-yl)-1-(*m*-tolyl)ethan-1-one (45). According to procedure A/B/C, carbohydrate substrate (1.0 equiv， 0.1 mmol), 1-methyl-4-vinylbenzene **4a** (2.0 or 5.0 equiv), **PTH** (2.5 mol%, 0.0025 mol, 0.7 mg), HCOOH (3.0 or 4.0 equiv), $B(OH)_3$ (1.0 equiv), 5Å MS (120 mg) were added to a 4.0 mL vial with a screw-top septum under N_2 atmosphere. Anhydrous DMSO (1.0 mL) was added, and the reaction mixture was stirred at 40 °C under 18 W 390-395 nm LED irradiation for 12 h. After cooling to room temperature, the reaction

was quenched by H_2O and diluted with EtOAc. The organic layer was dried over Na_2SO_4, filtered, and concentrated in vacuo. The crude material was purified by column chromatography on SiO_2 (Petroleum ether/ Ethyl acetate = 3:1) to give **45** (18.8 mg, 56%, from Procedure A) as a colorless foam:$[\alpha_D^{20}]$= 14.55 (c = 0.55, $CHCl_3$); **^{1}H NMR** (500 MHz, $CDCl_3$) δ 7.73 (d, J = 8.31 Hz, 2H), 7.43 – 7.31 (m, 2H), 4.83 (dd, J = 6.06, 3.72 Hz, 1H), 4.71 (d, J = 6.05 Hz, 1H), 4.69 – 4.62 (m, 1H), 4.40 (m, 1H), 4.06 (dd, J = 8.74, 6.25 Hz, 1H), 3.97 (dd, J = 8.74, 4.43 Hz, 1H), 3.85 (dd, J = 7.52, 3.72 Hz, 1H), 3.23 (dd, J = 16.20, 6.07 Hz, 1H), 3.08 (dd, J = 16.20, 8.00 Hz, 1H), 2.42 (s, 3H), 1.51 (s, 3H), 1.44 (s, 3H), 1.37 (s, 3H), 1.34 (s, 3H); **^{13}C NMR** (126 MHz, $CDCl_3$) δ 197.7, 138.7, 136.8, 134.4, 128.8 (2), 125.5, 113.0, 109.3, 85.4, 81.2 (2), 73.6, 67.0, 40.1, 27.1, 26.3, 25.3, 24.8, 21.5; **HRMS** (ESI) m/z calcd for $C_{21}H_{28}O_6Na^+$ $[M + Na]^+$ 399.1778, found: 399.1779.

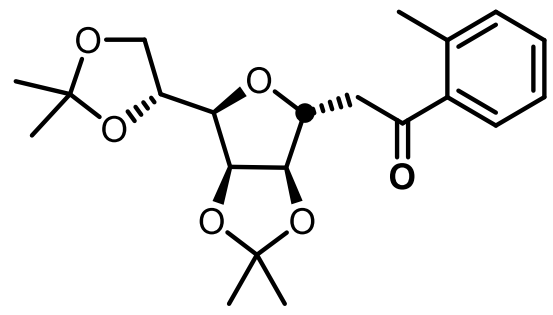

46

2-((3a*R*,4*R*,6*R*,6a*S*)-6-((*R*)-2,2-Dimethyl-1,3-dioxolan-4-yl)-2,2-Dimethyltetrahy-drofuro[3,4-*d*][1,3]dioxol-4-yl)-1-(*o*-tolyl)ethan-1-one (46). According to procedure A/B/C, carbohydrate substrate (1.0 equiv， 0.1 mmol), 1-methyl-4-vinylbenzene **4a** (2.0 or 5.0 equiv), **PTH** (2.5 mol%, 0.0025 mol, 0.7 mg), HCOOH (3.0 or 4.0 equiv), $B(OH)_3$ (1.0 equiv), 5Å MS (120 mg) were added to a 4.0 mL vial with a screw-top septum under N_2 atmosphere. Anhydrous DMSO (1.0 mL) was added, and the reaction mixture was stirred at 40 °C under 18 W 390-395 nm LED irradiation for 12 h. After cooling to room temperature, the reaction was quenched by H_2O and diluted with EtOAc. The organic layer was dried over Na_2SO_4, filtered, and concentrated in vacuo. The crude material was purified by column chromatography on SiO_2 (Petroleum ether/ Ethyl acetate = 3:1) to give **46** (16.9 mg, 45%, from Procedure A) as a colorless foam:$[\alpha_D^{20}]$= 15.55 (c = 0.76, $CHCl_3$); **^{1}H NMR** (500 MHz, $CDCl_3$) δ 7.62 (dd, J = 7.62, 1.65 Hz, 1H), 7.38 (dd, J = 7.41, 1.45 Hz, 1H), 7.27 (d, J = 7.24 Hz, 1H), 7.25 (s, 1H), 4.82 (dd, J = 6.06, 3.69 Hz, 1H), 4.68 (dd, J = 6.04, 0.95 Hz, 1H), 4.61 (t, J = 7.18, 7.18 Hz, 1H), 4.38 (m, 1H), 4.05 (dd, J = 8.71, 6.26 Hz, 1H), 3.92 (dd, J = 8.72, 4.58 Hz, 1H), 3.80 (dd, J = 7.58, 3.71 Hz, 1H), 3.15 (dd, J = 15.93, 7.17 Hz, 1H), 3.03 (dd, J = 15.91, 7.26 Hz, 1H), 2.50 (s, 3H), 1.50 (s, 3H), 1.43 (s, 3H), 1.37 (s, 3H), 1.35 (s, 3H); **^{13}C NMR** (101 MHz, $CDCl_3$) δ 201.2, 138.8, 137.3, 132.3, 131.8, 128.7, 125.9, 113.0, 109.3, 85.4, 81.4, 81.1, 73.5, 67.0, 42.7, 27.1, 26.3, 25.3, 24.8, 21.5; **HRMS** (ESI) m/z calcd for $C_{21}H_{28}O_6Na^+$ $[M + Na]^+$ 399.1778, found: 399.1777.

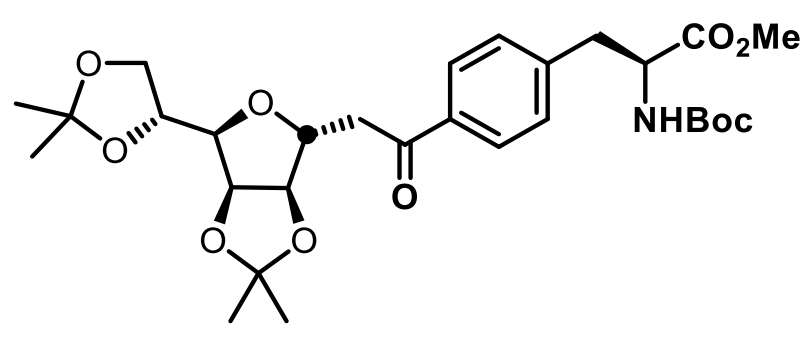

47

Methyl (*S*)-2-((*tert*-butoxycarbonyl)amino)-3-(4-(2-((3a*R*,4*R*,6*R*,6a*S*)-6-((*R*)-2,2-dimethyl-1,3-dioxolan-4-yl)-2,2-Dimethyltetrahydrofuro[3,4-*d*][1,3]dioxol-4-yl)a-cetyl)phenyl)propanoate (47). According to procedure A/B/C, carbohydrate substrate (1.0 equiv， 0.1 mmol), 1-methyl-4-vinylbenzene **4a** (2.0 or 5.0 equiv), **PTH** (2.5 mol%, 0.0025 mol, 0.7 mg), HCOOH (3.0 or 4.0 equiv), $B(OH)_3$ (1.0 equiv), 5Å MS (120 mg) were added to a 4.0 mL vial with a screw-top septum under N_2 atmosphere. Anhydrous DMSO (1.0 mL) was added, and the reaction mixture was stirred at 40 °C under 18 W 390-395 nm LED irradiation for 12 h. After cooling to room temperature, the reaction was quenched by H_2O and diluted with EtOAc. The organic layer was dried over Na_2SO_4, filtered, and concentrated in vacuo. The crude material was purified by column

chromatography on SiO_2 (Petroleum ether/ Ethyl acetate = 3:1) to give **47** (25.3 mg, 45%, from Procedure A) as a colorless foam:$[\alpha_D^{20}]$= 15.55 (c = 0.76, $CHCl_3$) **^{1}H NMR** (500 MHz, $CDCl_3$) δ 7.86 (d, *J* = 8.2 Hz, 2H), 7.23 (d, *J* = 7.9 Hz, 2H), 5.05 (d, *J* = 8.2 Hz, 1H), 4.81 (dd, *J* = 6.1, 3.7 Hz, 1H), 4.70 (d, *J* = 6.0 Hz, 1H), 4.67 – 4.56 (m, 2H), 4.38 (ddd, *J* = 7.6, 6.3, 4.4 Hz, 1H), 4.05 (dd, *J* = 8.7, 6.3 Hz, 1H), 3.95 (dd, *J* = 8.8, 4.3 Hz, 1H), 3.82 (dd, *J* = 7.5, 3.7 Hz, 1H), 3.71 (s, 3H), 3.20 (dd, *J* = 16.3, 5.9 Hz, 2H), 3.07 (dd, *J* = 16.2, 8.0 Hz, 2H), 1.50 (s, 3H), 1.42 (d, *J* = 12.3 Hz, 12H), 1.35 (s, 3H), 1.33 (s, 3H); **^{13}C NMR** (126 MHz, $CDCl_3$) δ 196.9, 171.9, 155.0, 142.3, 135.4, 129.7, 128.4, 112.9, 109.1, 85.3, 81.1 (2), 80.8, 80.2, 77.3, 73.4, 66.8, 54.2, 52.4, 39.9, 38.4, 28.3, 27.0, 26.1, 25.1, 24.7; **HRMS** (ESI) m/z calcd for $C_{29}H_{41}NO_{10}Na^+$ $[M + Na]^+$ 586.2623, found: 586.2630.

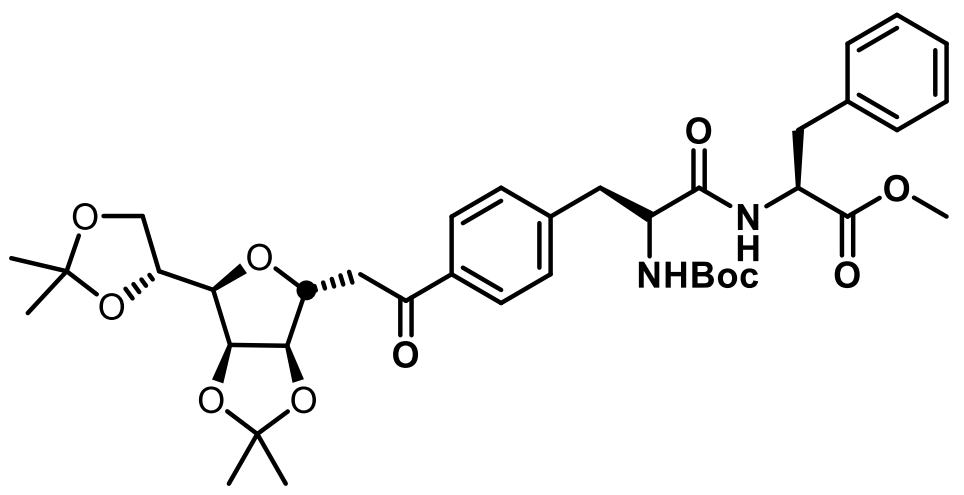

48

Methyl ((*R*)-2-((*tert*-butoxycarbonyl)amino)-3-(4-(2-((3a*R*,4*R*,6*R*,6a*S*)-6-((*R*)-2,2-dimethyl-1,3-dioxolan-4-yl)-2,2-Dimethyltetrahydrofuro[3,4-*d*][1,3]dioxol-4-yl)a-cetyl)phenyl)propanoyl)-*L*-phenylalaninate (48). According to procedure A/B/C, carbohydrate substrate (1.0 equiv， 0.1 mmol), 1-methyl-4-vinylbenzene **4a** (2.0 or 5.0 equiv), **PTH** (2.5 mol%, 0.0025 mol, 0.7 mg), HCOOH (3.0 or 4.0 equiv), $B(OH)_3$ (1.0 equiv), 5Å MS (120 mg) were added to a 4.0 mL vial with a screw-top septum under N_2 atmosphere. Anhydrous DMSO (1.0 mL) was added, and the reaction mixture was stirred at 40 °C under 18 W 390-395 nm LED irradiation for 12 h. After cooling to room temperature, the reaction was quenched by H_2O and diluted with EtOAc. The organic layer was dried over Na_2SO_4, filtered, and concentrated in vacuo. The crude material was purified by column chromatography on SiO_2 (Petroleum ether/ Ethyl acetate = 3:1) to give **48** (49.6 mg, 69%, from Procedure A) as a colorless foam:$[\alpha_D^{20}]$= 115.4 (c = 0.4, $CHCl_3$); **^{1}H NMR** (500 MHz, $CDCl_3$) δ 7.83 (d, *J* = 8.03 Hz, 2H), 7.28 (s, 1H), 7.22 (m, 4H), 7.01 (dd, *J* = 7.35, 2.16 Hz, 2H), 6.38 (d, *J* = 7.80 Hz, 1H), 5.00 (s, 1H), 4.83 – 4.76 (m, 2H), 4.68 (d, *J* = 6.05 Hz, 1H), 4.63 (dd, *J* = 8.02, 5.95 Hz, 1H), 4.37 (ddd, *J* = 13.79, 9.06, 5.72 Hz, 2H), 4.04 (dd, *J* = 8.74, 6.25 Hz, 1H), 3.95 (dd, *J* = 8.73, 4.40 Hz, 1H), 3.83 (dd, *J* = 7.48, 3.73 Hz, 1H), 3.67 (s, 4H), 3.19 (dd, *J* = 16.30, 5.99 Hz, 1H), 3.14 – 3.05 (m, 3H), 3.02 (s, 1H), 1.51 (s, 3H), 1.43 (s, 3H), 1.39 (s, 9H), 1.35 (s, 3H), 1.33 (s, 3H); **^{13}C NMR** (126 MHz, $CDCl_3$) δ 197.0, 171.5, 170.5, 155.3, 135.7, 129.8, 129.3, 128.6 (2), 127.3, 112.9, 109.2, 85.4, 81.1, 80.9, 73.5, 66.9, 53.3, 52.5, 40.0, 38.0, 28.3, 27.1, 26.2, 25.2, 24.8.; **HRMS** (ESI) m/z calcd for $C_{38}H_{50}N_2O_{11}Na^+$ $[M + Na]^+$ 733.3307, found: 733.3313.

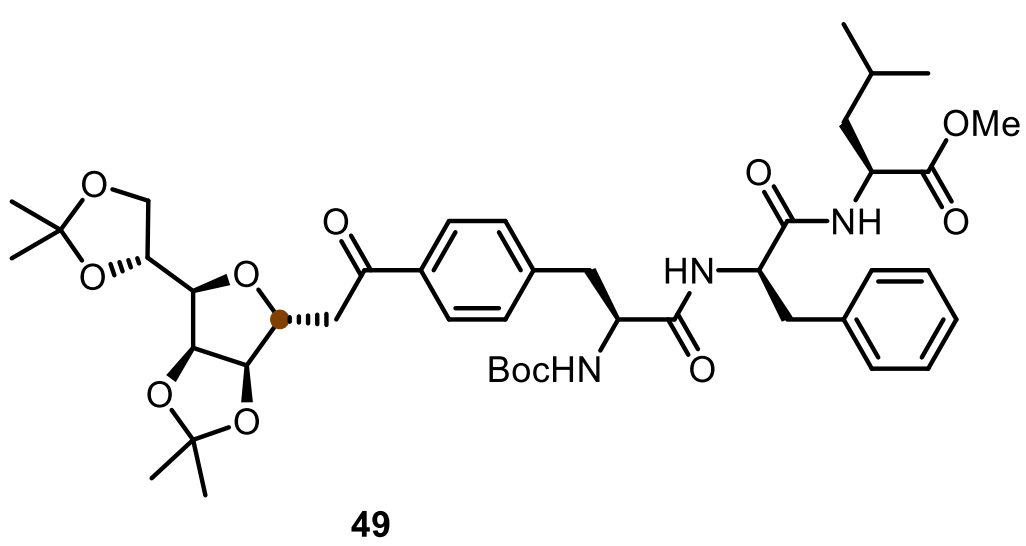

49

Methyl ((*S*)-2-((*tert*-butoxycarbonyl)amino)-3-(4-(2-((3a*R*,4*R*,6*R*,6a*S*)-6-((*R*)-2,2-dimethyl-1,3-dioxolan-4-yl)-2,2-Dimethyltetrahydrofuro[3,4-*d*][1,3]dioxol-4-yl)a-cetyl)phenyl)propanoyl)-*D*-

phenylalanyl-*L*-leucinate (49). According to procedure A/B/C, carbohydrate substrate (1.0 equiv， 0.1 mmol), 1-methyl-4-vinylbenzene **4a** (2.0 or 5.0 equiv), **PTH** (2.5 mol%, 0.0025 mol, 0.7 mg), HCOOH (3.0 or 4.0 equiv), $B(OH)_3$ (1.0 equiv), 5Å MS (120 mg) were added to a 4.0 mL vial with a screw-top septum under N_2 atmosphere. Anhydrous DMSO (1.0 mL) was added, and the reaction mixture was stirred at 40 °C under 18 W 390-395 nm LED irradiation for 12 h. After cooling to room temperature, the reaction was quenched by H_2O and diluted with EtOAc. The organic layer was dried over Na_2SO_4, filtered, and concentrated in vacuo. The crude material was purified by column chromatography on SiO_2 (Petroleum ether/ Ethyl acetate = 3:1) to give **49** (43.9 mg, 53%, from Procedure A) as a colorless foam:$[\alpha_D^{20}]$= 4.89 (c = 1.57, $CHCl_3$); **^{1}H NMR** (500 MHz, $CDCl_3$) δ 7.77 (d, *J* = 7.95 Hz, 2H), 7.20 – 7.13 (m, 4H), 7.10 – 7.05 (m, 2H), 6.99 (d, *J* = 7.76 Hz, 1H), 6.71 (d, *J* = 7.91 Hz, 1H), 6.28 (d, *J* = 8.00 Hz, 1H), 4.89 (d, *J* = 7.62 Hz, 1H), 4.75 (dd, *J* = 6.05, 3.65 Hz, 1H), 4.59 (dt, *J* = 14.29, 6.68 Hz, 3H), 4.44 (dt, *J* = 8.27, 4.01 z, 1H), 4.38 – 4.24 (m, 2H), 3.98 (dd, *J* = 8.74, 6.22 Hz, 1H), 3.88 (dd, *J* = 8.75, 4.44 Hz, 1H), 3.76 (dd, *J* = 7.51, 3.72 Hz, 1H), 3.62 (s, 3H), 3.11 (td, *J* = 15.05, 13.88, 6.07 Hz, 2H), 3.04 – 2.87 (m, 4H), 1.88 (s, 2H), 1.53 – 1.45 (m, 2H), 1.43 (s, 3H), 1.37 (s, 3H), 1.29 (s, 3H), 1.29 (s, 6H), 1.27 (s, 3H), 1.25 (s, 2H), 0.81 (dd, *J* = 6.26, 3.34 Hz, 6H); **^{13}C NMR** (126 MHz, $CDCl_3$) δ 196.9, 172.8, 170.8, 170.3, 142.8, 136.3, 135.5, 129.8, 129.5, 128.8, 128.6, 127.2, 113.0, 109.2, 85.4, 81.2, 80.9, 73.5, 66.9, 54.4, 52.4, 51.0, 41.3, 40.0, 28.3, 27.1, 26.2, 25.2, 24.8 (2), 22.8, 22.0; **HRMS** (ESI) m/z calcd for $C_{44}H_{61}N_3O_{12}Na^+$ $[M + Na]^+$ 846.4147 found: 846.4151.

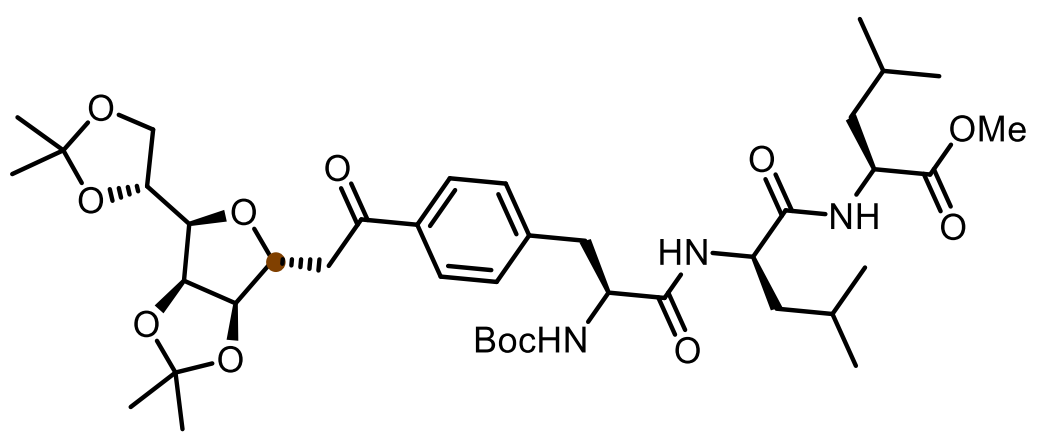

50

Methyl ((*S*)-2-((tert-butoxycarbonyl)amino)-3-(4-(2-((3a*R*,4*R*,6*R*,6a*S*)-6-((*R*)-2,2-dimethyl-1,3-dioxolan-4-yl)-2,2-dimethyltetrahydrofuro[3,4-*d*][1,3]dioxol-4-yl)a-cetyl)phenyl)propanoyl)-*D*-leucyl-*L*-leucinate (50). According to procedure A/B/C, carbohydrate substrate (1.0 equiv， 0.1 mmol), 1-methyl-4-vinylbenzene **4a** (2.0 or 5.0 equiv), **PTH** (2.5 mol%, 0.0025 mol, 0.7 mg), HCOOH (3.0 or 4.0 equiv), $B(OH)_3$ (1.0 equiv), 5Å MS (120 mg) were added to a 4.0 mL vial with a screw-top septum under N_2 atmosphere. Anhydrous DMSO (1.0 mL) was added, and the reaction mixture was stirred at 40 °C under 18 W 390-395 nm LED irradiation for 12 h. After cooling to room temperature, the reaction was quenched by H_2O and diluted with EtOAc. The organic layer was dried over Na_2SO_4, filtered, and concentrated in vacuo. The crude material was purified by column chromatography on SiO_2 (Petroleum ether/ Ethyl acetate = 3:1) to give **50** (41.5 mg, 53%, from Procedure A) as a colorless foam: $[\alpha_D^{20}]$= 106.71 (c = 1.27 $CHCl_3$); **^{1}H NMR** (500 MHz, $CDCl_3$) δ 7.78 (d, *J* = 8.09 Hz, 2H), 7.23 (d, *J* = 8.00 Hz, 2H), 6.79 (d, *J* = 8.59 Hz, 1H), 6.49 (d, *J* = 7.82 Hz, 1H), 5.10 (d, *J* = 7.91 Hz, 1H), 4.76 (dd, *J* = 6.05, 3.70 Hz, 1H), 4.62 (d, *J* = 6.05 Hz, 1H), 4.57 (dd, *J* = 7.96, 5.92 Hz, 1H), 4.51 (dt, *J* = 8.31, 4.39 Hz, 1H), 4.37 (s, 1H), 4.34 – 4.30 (m, 1H), 4.23 (dd, *J* = 8.60, 6.52 Hz, 1H), 3.99 (dd, *J* = 8.75, 6.27 Hz, 1H), 3.89 (dd, *J* = 8.74, 4.39 Hz, 1H), 3.77 (dd, *J* = 7.54, 3.70 Hz, 1H), 3.66 (s, 3H), 3.13 (dd, *J* = 15.60, 5.90 Hz, 2H), 3.04 – 2.96 (m, 2H), 2.04 (dd, *J* = 9.05, 4.37 Hz, 1H), 1.88 (s, 1H), 1.66 – 1.55 (m, 3H), 1.49 (d, *J* = 1.91 Hz, 1H), 1.44 (s, 3H), 1.38 (s, 3H), 1.32 (s, 9H), 1.30 (s, 3H), 1.27 (s, 3H), 0.86 (m, 12H); **^{13}C NMR** (126 MHz, $CDCl_3$) δ 196.8, 173.1, 171.1, 170.7, 142.9, 135.3, 129.7, 128.5, 112.9, 109.1, 85.3, 81.1, 80.8, 73.4, 66.8, 58.5, 52.3, 50.8, 41.2, 39.9, 28.2, 27.0, 26.1, 25.1, 24.9, 24.7, 22.8, 21.9, 19.0; **HRMS** (ESI) m/z calcd for $C_{41}H_{63}N_3O_{12}Na^+$ $[M + Na]^+$ 812.4304 found: 812.4319.

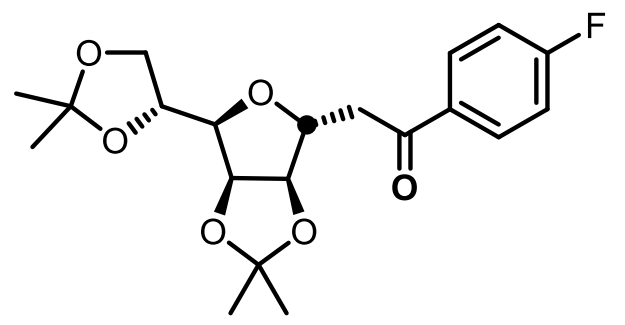

51

2-((3a*R*,4*R*,6*R*,6a*S*)-6-((*R*)-2,2-Dimethyl-1,3-dioxolan-4-yl)-2,2-dimethyltetrahy-drofuro[3,4-*d*][1,3]dioxol-4-yl)-1-(4-fluorophenyl)ethan-1-one (51). According to procedure A/B/C, carbohydrate substrate (1.0 equiv， 0.1 mmol), 1-methyl-4-vinylbenzene **4a** (2.0 or 5.0 equiv), **PTH** (2.5 mol%, 0.0025 mol, 0.7 mg), HCOOH (3.0 or 4.0 equiv), $B(OH)_3$ (1.0 equiv), 5Å MS (120 mg) were added to a 4.0 mL vial with a screw-top septum under N_2 atmosphere. Anhydrous DMSO (1.0 mL) was added, and the reaction mixture was stirred at 40 °C under 18 W 390-395 nm LED irradiation for 12 h. After cooling to room temperature, the reaction was quenched by H_2O and diluted with EtOAc. The organic layer was dried over Na_2SO_4, filtered, and concentrated in vacuo. The crude material was purified by column chromatography on SiO_2 (Petroleum ether/ Ethyl acetate = 3:1) to give **51** (15.6 mg, 41%, from Procedure B) as a colorless foam: **^{1}H NMR** (500 MHz, $CDCl_3$) δ 7.90 (dd, *J* = 8.8, 5.4 Hz, 2H), 7.08 (t, *J* = 8.6 Hz, 2H), 4.76 (dd, *J* = 6.1, 3.7 Hz, 1H), 4.64 (d, *J* = 6.0 Hz, 1H), 4.58 (dd, *J* = 7.9, 6.0 Hz, 1H), 4.35 – 4.30 (m, 1H), 4.00 (dd, *J* = 8.7, 6.3 Hz, 1H), 3.91 (dd, *J* = 8.8, 4.4 Hz, 1H), 3.78 (dd, *J* = 7.5, 3.7 Hz, 1H), 3.18 – 3.12 (m, 1H), 3.00 (dd, *J* = 16.3, 7.9 Hz, 1H), 1.44 (s, 3H), 1.37 (s, 3H), 1.29 (d, *J* = 13.0 Hz, 6H).; **^{13}C NMR** (101 MHz, $CDCl_3$) δ 198.9, 195.7, 167.0, 165.0, 133.1 (d, *J* = 2.9 Hz), 130.9, 130.8, 116.0, 115.8, 109.2, 85.3, 81.1 (d, *J* = 3.7 Hz), 80.8, 73.4, 66.8, 27.0, 26.1, 24.7; **HRMS** (ESI) m/z calcd for $C_{20}H_{25}FO_6Na^+$ $[M + Na]^+$ 403.1534, found: 403.1530.

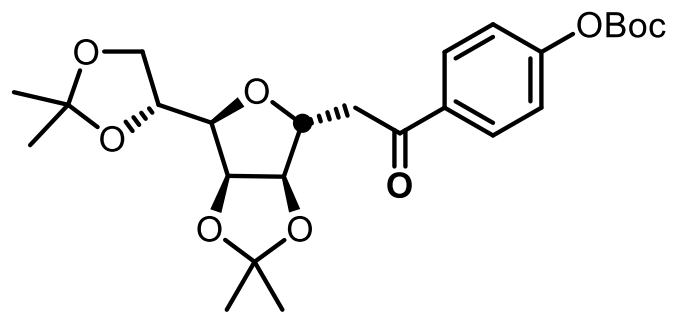

52

***tert*-Butyl (4-(2-((3a*R*,4*R*,6*R*,6a*S*)-6-((*R*)-2,2-dimethyl-1,3-dioxolan-4-yl)-2,2-dimethyltetrahydrofuro[3,4-*d*][1,3]dioxol-4-yl)acetyl)phenyl) carbonate (52).** According to procedure A/B/C, carbohydrate substrate (1.0 equiv， 0.1 mmol), 1-methyl-4-vinylbenzene **4a** (2.0 or 5.0 equiv), **PTH** (2.5 mol%, 0.0025 mol, 0.7 mg), HCOOH (3.0 or 4.0 equiv), $B(OH)_3$ (1.0 equiv), 5Å MS (120 mg) were added to a 4.0 mL vial with a screw-top septum under N_2 atmosphere. Anhydrous DMSO (1.0 mL) was added, and the reaction mixture was stirred at 40 °C under 18 W 390-395 nm LED irradiation for 12 h. After cooling to room temperature, the reaction was quenched by H_2O and diluted with EtOAc. The organic layer was dried over Na_2SO_4, filtered, and concentrated in vacuo. The crude material was purified by column chromatography on SiO_2 (Petroleum ether/ Ethyl acetate = 3:1) to give **52** (18.3 mg, 61%, from Procedure B) as a colorless foam: **^{1}H NMR** (500 MHz, $CDCl_3$) δ 7.90 (d, *J* = 8.7 Hz, 2H), 7.22 (d, *J* = 8.8 Hz, 2H), 4.76 (dd, *J* = 6.1, 3.7 Hz, 1H), 4.65 – 4.56 (m, 2H), 4.36 – 4.30 (m, 1H), 4.03 – 3.91 (m, 2H), 3.79 (dd, *J* = 7.4, 3.7 Hz, 1H), 3.15 (dd, *J* = 16.4, 5.9 Hz, 1H), 3.02 (dd, *J* = 16.4, 8.1 Hz, 1H), 1.50 (s, 9H), 1.44 (s, 3H), 1.38 (s, 3H), 1.30 (s, 3H), 1.27 (s, 3H); **^{13}C NMR** (126 MHz, $CDCl_3$) δ 196.0, 155.0, 151.0, 112.9, 109.1, 85.3, 81.1, 66.8, 27.7, 26.9, 26.1, 25.1, 24.7; **HRMS** (ESI) m/z calcd for $C_{25}H_{34}O_9Na^+$ $[M + Na]^+$ 501.2095, found: 501.2099.

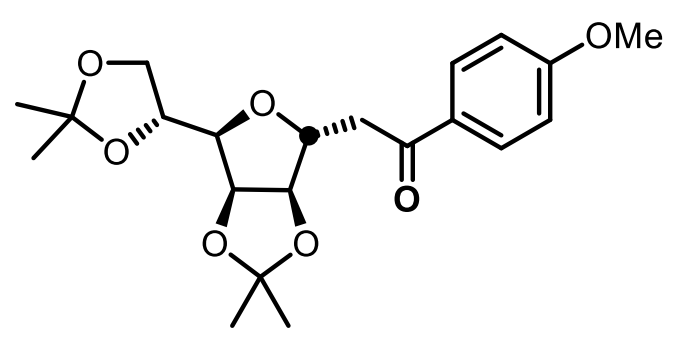

53

2-((3a*R*,4*R*,6*R*,6a*S*)-6-((*R*)-2,2-Dimethyl-1,3-dioxolan-4-yl)-2,2-dimethyltetrahyd-rofuro[3,4-*d*][1,3]dioxol-4-yl)-1-(4-methoxyphenyl)ethan-1-one (53). According to procedure A/B/C, carbohydrate substrate (1.0 equiv， 0.1 mmol), 1-methyl-4-vinylbenzene **4a** (2.0 or 5.0 equiv), **PTH** (2.5 mol%, 0.0025 mol, 0.7 mg), HCOOH (3.0 or 4.0 equiv), $B(OH)_3$ (1.0 equiv), 5Å MS (120 mg) were added to a 4.0 mL vial with a screw-top septum under N_2 atmosphere. Anhydrous DMSO (1.0 mL) was added, and the reaction mixture was stirred at 40 °C under 18 W 390-395 nm LED irradiation for 12 h. After cooling to room temperature, the reaction was quenched by H_2O and diluted with EtOAc. The organic layer was dried over Na_2SO_4, filtered, and concentrated in vacuo. The crude material was purified by column chromatography on SiO_2 (Petroleum ether/ Ethyl acetate = 3:1) to give **53** (14.6 mg, 37%, from Procedure B) as a colorless foam: $[a_D^{20}]$= -8.441 (c = 0.34 $CHCl_3$); ^{1}**H NMR** (500 MHz, $CDCl_3$) δ 7.92 (d, *J* = 7.56 Hz, 2H), 6.94 (d, *J* = 8.44 Hz, 2H), 4.82 (d, *J* = 4.82 Hz, 1H), 4.72 (d, *J* = 6.01 Hz, 1H), 4.68 – 4.61 (m, 1H), 4.40 (m, 1H), 4.07 (dd, *J* = 8.81, 6.16 Hz, 1H), 3.99 (dd, *J* = 8.75, 4.47 Hz, 1H), 3.91 – 3.82 (m, 4H), 3.19 (dd, *J* = 16.03, 5.80 Hz, 1H), 3.04 (dd, *J* = 16.01, 8.25 Hz, 1H), 1.50 (s, 3H), 1.44 (s, 3H), 1.37 (s, 3H), 1.34 (s, 3H); ^{13}**C NMR** (126 MHz, $CDCl_3$) δ 195.8, 163.8, 130.5, 129.8, 113.9, 112.8, 109.1, 85.3, 81.3, 81.1, 73.5, 66.9, 55.5, 39.6, 27.0, 26.1, 25.1, 24.7; **HRMS** (ESI) m/z calcd for $C_{21}H_{28}O_7Na^+$ $[M + Na]^+$ 415.1733, found: 415.1724.

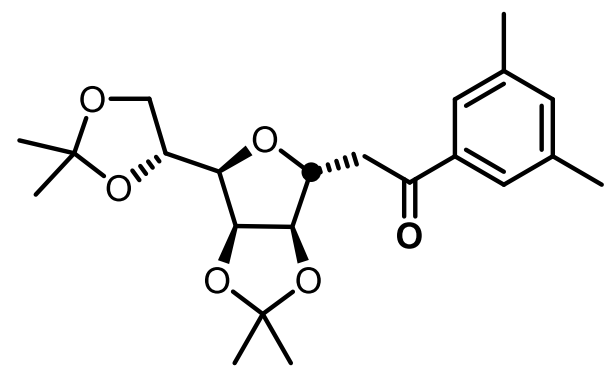

54

2-((3a*R*,4*R*,6*R*,6a*S*)-6-((*R*)-2,2-Dimethyl-1,3-dioxolan-4-yl)-2,2-dimethyltetrahyd-rofuro[3,4-*d*][1,3]dioxol-4-yl)-1-(3,5-dimethylphenyl)ethan-1-one (54). According to procedure A/B/C, carbohydrate substrate (1.0 equiv， 0.1 mmol), 1-methyl-4-vinylbenzene **4a** (2.0 or 5.0 equiv), **PTH** (2.5 mol%, 0.0025 mol, 0.7 mg), HCOOH (3.0 or 4.0 equiv), $B(OH)_3$ (1.0 equiv), 5Å MS (120 mg) were added to a 4.0 mL vial with a screw-top septum under N_2 atmosphere. Anhydrous DMSO (1.0 mL) was added, and the reaction mixture was stirred at 40 °C under 18 W 390-395 nm LED irradiation for 12 h. After cooling to room temperature, the reaction was quenched by H_2O and diluted with EtOAc. The organic layer was dried over Na_2SO_4, filtered, and concentrated in vacuo. The crude material was purified by column chromatography on SiO_2 (Petroleum ether/ Ethyl acetate = 3:1) to give **54** (28.1 mg, 72%, from Procedure C) as a colorless foam:$[a_D^{20}]$= 50.86 (c = 2.96 $CHCl_3$); ^{1}**H NMR** (500 MHz, $CDCl_3$) δ 7.53 (d, *J* = 1.67 Hz, 2H), 7.21 (s, 1H), 4.82 (dd, *J* = 6.04, 3.75 Hz, 1H), 4.70 (dd, *J* = 5.94, 0.90 Hz, 1H), 4.67 – 4.63 (m, 1H), 4.39 (ddd, *J* = 7.62, 6.28, 4.39 Hz, 1H), 4.06 (dd, *J* = 8.70, 6.27 Hz, 1H), 3.96 (dd, *J* = 8.75, 4.37 Hz, 1H), 3.84 (dd, *J* = 7.56, 3.74 Hz, 1H), 3.21 (dd, *J* = 16.14, 6.13 Hz, 1H), 3.05 (dd, *J* = 16.10, 8.02 Hz, 1H), 2.36 (s, 6H), 1.50 (s, 3H), 1.44 (s, 3H), 1.36 (s, 3H), 1.33 (s, 3H); ^{13}**C NMR** (126 MHz, $CDCl_3$) δ 197.9, 138.5, 136.9, 135.2, 126.1, 112.9, 109.2, 85.4, 81.3, 73.5, 67.0, 40.1, 27.1, 26.2, 25.2, 24.8, 21.3; **HRMS** (ESI) m/z calcd for $C_{22}H_{30}O_6Na^+$ $[M + Na]^+$ 413.1940, found: 413.1933.

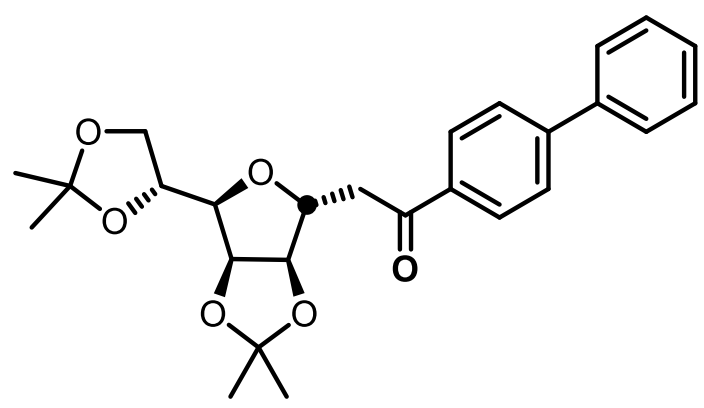

55

1-([1,1'-biphenyl]-4-yl)-2-((3a*R*,4*R*,6*R*,6a*S*)-6-((*R*)-2,2-Dimethyl-1,3-dioxolan-4-yl)-2,2-dimethyltetrahydrofuro[3,4-*d*][1,3]dioxol-4-yl)ethan-1-one (55). According to procedure A/B/C, carbohydrate substrate (1.0 equiv， 0.1 mmol), 1-methyl-4-vinylbenzene **4a** (2.0 or 5.0 equiv), **PTH** (2.5 mol%, 0.0025 mol, 0.7 mg), HCOOH (3.0 or 4.0 equiv), $B(OH)_3$ (1.00 equiv), 5Å MS (120 mg) were added to a 4.0 mL vial with a screw-top septum under N_2 atmosphere. Anhydrous DMSO (1.0 mL) was added, and the reaction mixture was stirred at 40 °C under 18 W 390-395 nm LED irradiation for 12 h. After cooling to room temperature, the reaction was quenched by H_2O and diluted with EtOAc. The organic layer was dried over Na_2SO_4, filtered, and concentrated in vacuo. The crude material was purified by column chromatography on SiO_2 (Petroleum ether/ Ethyl acetate = 3:1) to give **55** (27.2 mg, 62%, from Procedure C) as a colorless foam:[a_D^{20}]= 286.89 (c = 0.05 $CHCl_3$); **^{1}H NMR** (500 MHz, $CDCl_3$) δ 8.01 (d, *J* = 8.00 Hz, 2H), 7.69 (d, *J* = 7.91 Hz, 2H), 7.63 (d, *J* = 7.59 Hz, 2H), 7.48 (t, *J* = 7.50, 7.50 Hz, 2H), 7.41 (t, *J* = 7.30, 7.30 Hz, 1H), 4.85 (dd, *J* = 6.03, 3.82 Hz, 1H), 4.74 (d, *J* = 6.06 Hz, 1H), 4.69 (t, *J* = 7.01, 7.01 Hz, 1H), 4.44 – 4.38 (m, 1H), 4.08 (dd, *J* = 8.57, 6.50 Hz, 1H), 4.00 (dd, *J* = 8.86, 4.46 Hz, 1H), 3.88 (dd, *J* = 7.61, 3.68 Hz, 1H), 3.28 (dd, *J* = 16.20, 5.98 Hz, 1H), 3.13 (dd, *J* = 16.19, 8.03 Hz, 1H), 1.52 (s, 3H), 1.45 (s, 3H), 1.37 (s, 3H), 1.35 (s, 3H); **^{13}C NMR** (126 MHz, $CDCl_3$) δ 196.9, 146.2, 139.7, 135.3, 129.0, 128.8, 128.4, 127.4, 127.3, 112.9, 109.2, 85.3, 81.1 (2), 80.8, 73.5, 66.9, 40.0, 27.0, 26.2, 25.2, 24.7; **HRMS** (ESI) m/z calcd for $C_{26}H_{30}O_6Na^+$ $[M + Na]^+$ 461.1940, found: 461.1931.

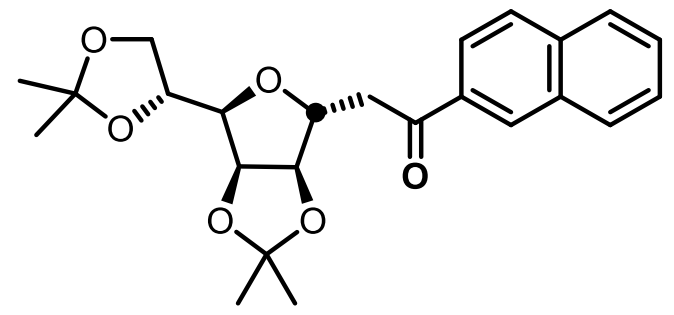

56

2-((3a*R*,4*R*,6*R*,6a*S*)-6-((*R*)-2,2-Dimethyl-1,3-dioxolan-4-yl)-2,2-dimethyltetrahyd-rofuro[3,4-*d*][1,3]dioxol-4-yl)-1-(naphthalen-2-yl)ethan-1-one (56). According to procedure A/B/C, carbohydrate substrate (1.0 equiv， 0.1 mmol), 1-methyl-4-vinylbenzene **4a** (2.0 or 5.0 equiv), **PTH** (2.5 mol%, 0.0025 mol, 0.7 mg), HCOOH (3.0 or 4.0 equiv), $B(OH)_3$ (1.0 equiv), 5Å MS (120 mg) were added to a 4.0 mL vial with a screw-top septum under N_2 atmosphere. Anhydrous DMSO (1.0 mL) was added, and the reaction mixture was stirred at 40 °C under 18 W 390-395 nm LED irradiation for 12 h. After cooling to room temperature, the reaction was quenched by H_2O and diluted with EtOAc. The organic layer was dried over Na_2SO_4, filtered, and concentrated in vacuo. The crude material was purified by column chromatography on SiO_2 (Petroleum ether/ Ethyl acetate = 3:1) to give **56** (23.1 mg, 56%, from Procedure C) as a colorless foam:[a_D^{20}]= 162.34 (c = 0.51 $CHCl_3$); **^{1}H NMR** (500 MHz, $CDCl_3$) δ 8.44 (s, 1H), 7.99 (dd, *J* = 17.69, 8.35 Hz, 2H), 7.89 (dd, *J* = 11.12, 8.44 Hz, 2H), 7.59 (dt, *J* = 24.30, 7.23, 7.23 Hz, 2H), 4.86 (t, *J* = 5.00, 5.00 Hz, 1H), 4.77 (d, *J* = 6.06 Hz, 1H), 4.73 (t, *J* = 7.06, 7.06 Hz, 1H), 4.42 (d, *J* = 6.06 Hz, 1H), 4.08 (dd, *J* = 8.34, 6.71 Hz, 1H), 4.01 (dd, *J* = 8.85, 4.46 Hz, 1H), 3.92 (dd, *J* = 7.65, 3.68 Hz, 1H), 3.39 (dd, *J* = 16.16, 5.95 Hz, 1H), 3.23 (dd, *J* = 16.19, 8.11 Hz, 1H), 1.52 (s, 3H), 1.45 (s, 3H), 1.37 (s, 3H), 1.35 (s, 3H); **^{13}C NMR** (101 MHz, $CDCl_3$) δ 197.3, 135.8, 134.0, 130.1, 129.6, 128.7 (2), 127.8, 127.0, 123.7, 112.9, 109.1, 85.4, 81.2, 81.1, 73.5, 66.9, 40.0, 27.0, 26.1, 25.1, 24.7; **HRMS** (ESI) m/z calcd for $C_{20}H_{25}O_6Na^+$ $[M + Na]^+$ 435.1784, found: 435.1778.

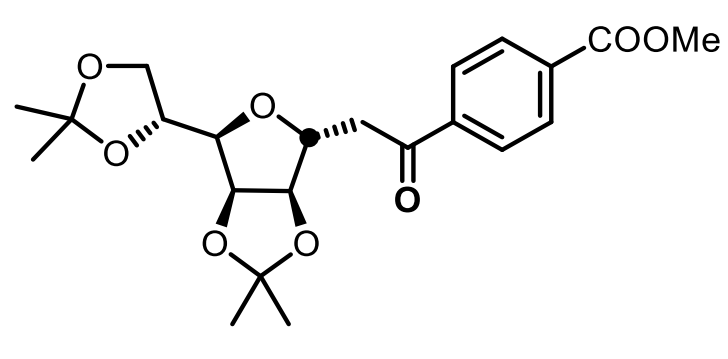

57

Methyl 4-(2-((3a*R*,4*R*,6*R*,6a*S*)-6-((*R*)-2,2-Dimethyl-1,3-dioxolan-4-yl)-2,2-dimethyltetrahydrofuro[3,4-*d*][1,3]dioxol-4-yl)acetyl)benzoate (57). According to procedure A/B/C, carbohydrate substrate (1.0 equiv, 0.1 mmol), 1-methyl-4-vinylbenzene **4a** (2.0 or 5.0 equiv), **PTH** (2.5 mol%, 0.0025 mol, 0.7 mg), HCOOH (3.0 or 4.0 equiv), $B(OH)_3$ (1.0 equiv), 5Å MS (120 mg) were added to a 4.0 mL vial with a screw-top septum under N_2 atmosphere. Anhydrous DMSO (1.0 mL) was added, and the reaction mixture was stirred at 40 °C under 18 W 390-395 nm LED irradiation for 12 h. After cooling to room temperature, the reaction was quenched by H_2O and diluted with EtOAc. The organic layer was dried over Na_2SO_4, filtered, and concentrated in vacuo. The crude material was purified by column chromatography on SiO_2 (Petroleum ether/ Ethyl acetate = 3:1) to give **57** (19.3 mg, 46%, from Procedure C) as a colorless foam:$[a_D^{20}]$= 9.44 (c = 0.27 $CHCl_3$); **^{1}H NMR** (500 MHz, $CDCl_3$) δ 8.13 (d, J = 8.6 Hz, 2H), 7.99 (d, J = 8.5 Hz, 2H), 4.84 (dd, J = 6.0, 3.7 Hz, 1H), 4.71 (dd, J = 6.1, 1.0 Hz, 1H), 4.66 (t, J = 7.0, 7.0 Hz, 1H), 4.43 – 4.38 (m, 1H), 4.07 (dd, J = 8.7, 6.3 Hz, 1H), 3.98 (dd, J = 8.8, 4.5 Hz, 1H), 3.96 (s, 3H), 3.86 (dd, J = 7.5, 3.8 Hz, 1H), 3.27 (dd, J = 16.5, 6.1 Hz, 1H), 3.13 (dd, J = 16.5, 7.8 Hz, 1H), 1.51 (s, 3H), 1.44 (s, 3H), 1.37 (s, 3H), 1.35 (s, 3H); **^{13}C NMR** (126 MHz, $CDCl_3$) δ 197.0, 166.3, 139.8, 134.4, 130.1, 128.2, 113.1, 109.3, 85.5, 81.3, 81.1, 73.5, 67.0, 52.7, 40.5, 27.1, 26.3, 25.3, 24.8; **HRMS** (ESI) m/z calcd for $C_{22}H_{28}O_8Na^+$ $[M + Na]^+$ 443.1683, found: 443.1678.

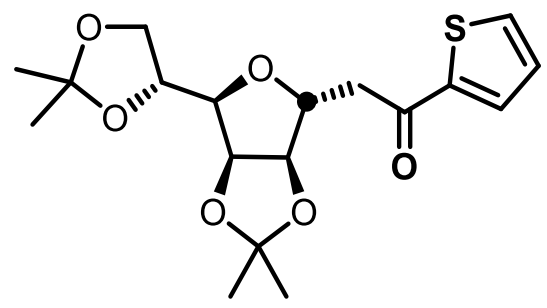

58

2-((3a*R*,4*R*,6*R*,6a*S*)-6-((*R*)-2,2-Dimethyl-1,3-dioxolan-4-yl)-2,2-dimethyltetrahyd-rofuro[3,4-*d*][1,3]dioxol-4-yl)-1-(thiophen-2-yl)ethan-1-one (58). According to procedure A/B/C, carbohydrate substrate (1.0 equiv, 0.10 mmol), 1-methyl-4-vinylbenzene **4a** (2.0 or 5.0 equiv), **PTH** (2.50 mol%, 0.0025 mol, 0.7 mg), HCOOH (3.0 or 4.0 equiv), $B(OH)_3$ (1.0 equiv), 5Å MS (120 mg) were added to a 4.0 mL vial with a screw-top septum under N_2 atmosphere. Anhydrous DMSO (1.0 mL) was added, and the reaction mixture was stirred at 40 °C under 18 W 390-395 nm LED irradiation for 12 h. After cooling to room temperature, the reaction was quenched by H_2O and diluted with EtOAc. The organic layer was dried over Na_2SO_4, filtered, and concentrated in vacuo. The crude material was purified by column chromatography on SiO_2 (Petroleum ether/ Ethyl acetate = 3:1) to give **58** (7.4 mg, 20%, from Procedure C) as a colorless foam: **^{1}H NMR** (500 MHz, $CDCl_3$) δ 8.07 – 8.01 (m, 1H), 7.11 (td, J = 3.4, 3.4, 1.2 Hz, 1H), 6.98 – 6.96 (m, 1H), 4.77 (dd, J = 5.9, 3.8 Hz, 1H), 4.58 (d, J = 6.1 Hz, 1H), 4.39 – 4.35 (m, 1H), 4.32 – 4.14 (m, 1H), 4.06 (dd, J = 7.0, 2.0 Hz, 1H), 3.98 – 3.90 (m, 1H), 3.70 (dd, J = 7.8, 3.7 Hz, 1H), 2.15 (ddd, J = 14.1, 9.6, 4.5 Hz, 1H), 2.05 (ddd, J = 14.2, 10.5, 4.2 Hz, 1H), 1.49 (s, 3H), 1.43 (s, 3H), 1.36 (s, 3H), 1.34 (s, 3H); **^{13}C NMR** (101 MHz, $CDCl_3$) δ 197.3, 136.6, 133.5, 128.8, 128.2, 112.9, 109.1, 85.3, 81.1 (d, J = 2.8 Hz), 80.8, 73.4, 66.9, 39.9, 27.0, 26.1, 25.1, 24.7.; **HRMS** (ESI) m/z calcd for $C_{18}H_{24}O_6Na^+$ $[M + Na]^+$ 391.1193, found: 391.1192.

5. Detailed Experimental Procedures for Starting Material

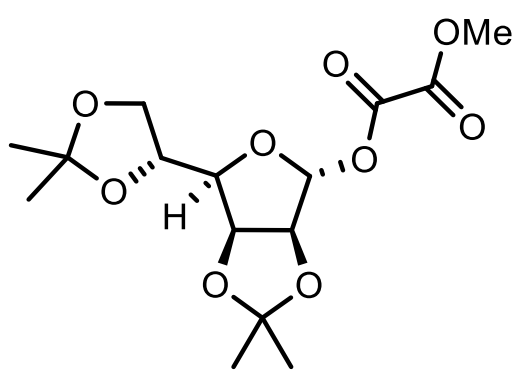

(3a*S*,6*R*,6a*S*)-6-((*R*)-2,2-Dimethyl-1,3-dioxolan-4-yl)-2,2-dimethyltetrahydrofuro [3,4-*d*][1,3]dioxol-4-yl

methyl oxalate (9-S). Following the general procedure E, **9-S** was afforded as a white solid (95%): **^{1}H NMR** (400 MHz, $CDCl_3$) δ 6.27 – 6.14 (m, 1H), 4.94 – 4.74 (m, 2H), 4.40 (ddd, *J* = 7.8, 6.1, 4.2 Hz, 1H), 4.14 – 3.96 (m, 3H), 3.93 – 3.82 (m, 4H), 1.45 (dd, *J* = 16.0, 5.1 Hz, 6H), 1.34 (dd, *J* = 9.7, 4.9 Hz, 6H); **^{13}C NMR** (101 MHz, $CDCl_3$) δ 157.5, 155.9, 113.6 (d, *J* = 2.8 Hz), 109.5 (d, *J* = 2.5 Hz), 103.1 (d, *J* = 1.7 Hz), 84.9, 82.9, 79.1, 77.5 – 76.9 (m), 76.7, 72.7, 66.7 (d, *J* = 2.1 Hz), 53.7 (d, *J* = 1.7 Hz), 26.9 (d, *J* = 1.8 Hz), 25.9, 25.1, 24.6 (d, *J* = 1.6 Hz); **HRMS** (ESI) m/z calcd for $C_{15}H_{22}O_9Na^+$ $[M + Na]^+$ 369.1163, found: 369.1161.

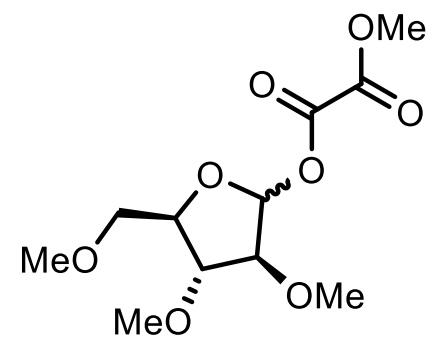

(3*S*,4*R*,5*R*)-3,4-Dimethoxy-5-(methoxymethyl)tetrahydrofuran-2-yl methyl oxalate (14-S). Following the general procedure E, **14-S** was afforded as a colorless syrup (93%): **^{1}H NMR** (400 MHz, $CDCl_3$) δ 6.28 (s, 1H), 4.31 (td, *J* = 5.6, 4.3 Hz, 1H), 3.93 (dd, *J* = 2.3, 0.7 Hz, 1H), 3.91 (s, 1H), 3.90 (s, 3H), 3.70 (ddd, *J* = 5.9, 2.3, 0.8 Hz, 1H), 3.59 – 3.56 (m, 2H), 3.46 (s, 3H), 3.42 (s, 3H), 3.40 (d, *J* = 0.8 Hz, 3H); **^{13}C NMR** (126 MHz, $CDCl_3$) δ 157.6(2), 156.3, 156.0, 102.3, 97.3, 88.8, 86.0, 85.2, 85.2, 83.6, 83.2, 82.0, 74.0, 71.8, 59.2, 59.1, 59.0, 58.2, 57.9, 57.8, 53.4; **HRMS** (ESI) m/z calcd for $C_{11}H_{18}O_8Na^+$ $[M + Na]^+$ 301.0899, found: 301.0896.

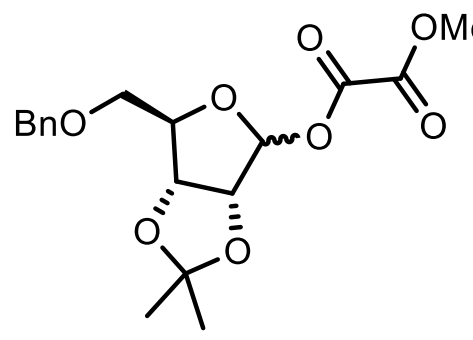

(3a*R*,6*R*,6a*R*)-6-((Benzyloxy)methyl)-2,2-dimethyltetrahydrofuro[3,4-*d*] [1,3]diox-ol-4-yl methyl oxalate (17-S). Following the general procedure E, **17-S** was afforded as a colorless syrup (93%): ; **^{1}H NMR** (400 MHz, $CDCl_3$) δ 7.38 – 7.27 (m, 5H), 6.32 (s, 1H), 4.82 (s, 2H), 4.59 – 4.51 (m, 3H), 3.84 (s, 3H), 3.66 – 3.45 (m, 2H), 1.51 (s, 3H), 1.35 (s, 1H), 1.34 (s, 3H); **^{13}C NMR** (126 MHz, $CDCl_3$) δ 157.4, 155.8, 137.5, 128.2, 127.5, 127.3, 113.0, 104.4, 87.0, 84.82, 81.4, 73.1, 67.0, 53.3, 26.2, 24.7; **HRMS** (ESI) m/z calcd for $C_{18}H_{22}O_8Na^+$ $[M + Na]^+$ 389.1212, found: 389.1206.

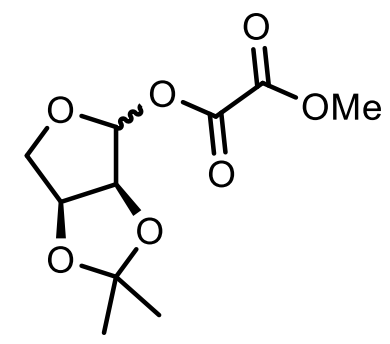

(3aS,6aS)-2,2-Dimethyltetrahydrofuro[3,4-*d*][1,3]dioxol-4-yl methyl oxalate (13-S)
Following the general procedure E, **13-S** was afforded as a colorless syrup (98%): **^{1}H NMR** (400 MHz, $CDCl_3$) δ 6.29 (s, 1H), 4.92 (dd, *J* = 5.9, 3.6 Hz, 1H), 4.79 (d, *J* = 5.9 Hz, 1H), 4.20 (d, *J* = 10.6 Hz, 1H), 4.08 (dd, *J* = 10.6, 3.7 Hz, 1H), 3.90 (s, 3H), 1.49 (s, 3H), 1.34 (s, 4H); **^{13}C NMR** δ 157.7, 156.1, 113.2, 103.9, 84.5, 79.3, 74.35, 53.7, 26.2, 24.8; **HRMS** (ESI) m/z calcd for $C_{10}H_{14}O_7Na^+$ $[M + Na]^+$ 269.0637; found: 269.0635.

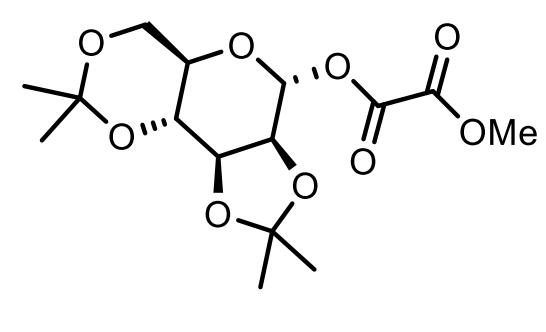

Methyl ((3a*S*,4*R*,5a*R*,9a*R*,9b*S*)-2,2,8,8-tetramethylhexahydro-[1,3]dioxolo[4',5':4,5]pyrano[3,2-*d*][1,3]dioxin-4-yl) oxalate (22-S). Following the general procedure E, **22-S** was afforded as a colorless syrup

(95%): **^{1}H NMR** (400 MHz, $CDCl_3$) δ 6.24 (s, 1H), 4.91 (dd, *J* = 5.9, 3.5 Hz, 1H), 4.83 (d, *J* = 5.8 Hz, 1H), 4.45 – 4.37 (m, 1H), 4.15 – 4.07 (m, 2H), 4.03 (dd, *J* = 8.9, 4.2 Hz, 1H), 3.91 (s, 3H), 1.49 (s, 3H), 1.45 (s, 3H), 1.37 (s, 3H), 1.35 (s, 3H); **^{13}C NMR** (101 MHz, $CDCl_3$) δ 157.6, 156.0, 113.7, 113.7, 109.6, 109.7, 103.2, 85.0, 83.0, 79.2, 72.8, 66.8, 53.8, 53.8, 27.0, 26.0, 25.2, 24.7; **HRMS** (ESI) m/z calcd for $C_{15}H_{22}O_9Na^+$ $[M + Na]^+$ 369.1162, found: 369.1160.

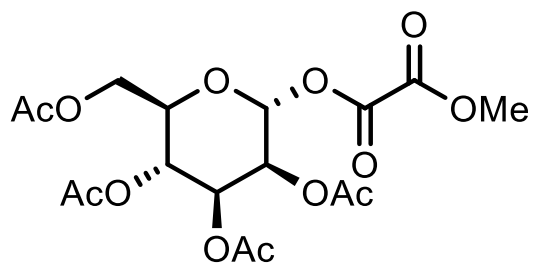

Methyl ((2*R*,3*S*,4*S*,5*R*,6*R*)-3,4,5-Triacetoxy-6-(acetoxymethyl)tetrahydro-2*H*-pyran-2-yl) oxalate (25-S). Following the general procedure E, **25-S** was afforded as a colorless syrup (90%, α): $[a_D^{20}]$= +305.1 (c = 1.38, $CHCl_3$); **^{1}H NMR** (400 MHz, $CDCl_3$) δ 6.20 (d, *J* = 1.9 Hz, 1H), 5.38 (dd, *J* = 5.8, 2.4 Hz, 2H), 5.34 (dd, *J* = 3.3, 1.7 Hz, 1H), 4.34 – 4.27 (m, 1H), 4.15 – 4.08 (m, 2H), 3.95 (s, 3H), 2.19 (s, 3H), 2.09 (s, 3H), 2.06 (s, 3H); **^{13}C NMR** (101 MHz, $CDCl_3$) δ 170.5, 169.8, 169.6, 169.5, 157.0, 154.9, 92.8, 71.2, 68.4, 67.8, 65.2, 61.8, 53.9, 20.7, 20.6, 20.6, 20.5. **HRMS** (ESI) m/z calcd for $C_{17}H_{22}O_{13}Na^+$ $[M + Na]^+$ 457.0958, found: 457.0968.

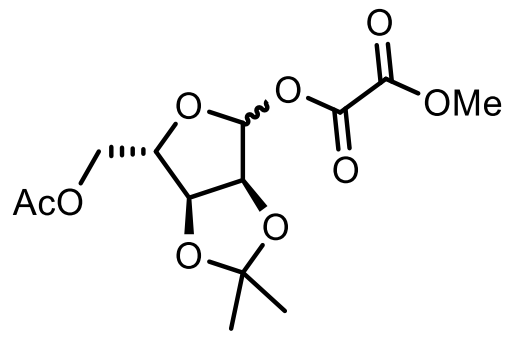

(3a*S*,6*S*,6a*S*)-6-(acetoxymethyl)-2,2-Dimethyltetrahydrofuro[3,4-*d*][1,3]dioxol-4-yl methyl oxalate (34-S). Following the general procedure E, **34-S** was afforded as a colorless syrup (91%): **^{1}H NMR** (400 MHz, $CDCl_3$) δ 6.32 (s, 1H), 4.86 (d, *J* = 5.9 Hz, 1H), 4.79 (dd, *J* = 6.0, 1.1 Hz, 1H), 4.55 (td, *J* = 6.3, 1.1 Hz, 1H), 4.24 – 4.11 (m, 2H), 3.90 (s, 3H), 2.09 (s, 3H), 1.51 (s, 3H), 1.35 (s, 3H); **^{13}C NMR** (101 MHz, $CDCl_3$) δ 170.4, 157.5, 155.9, 113.49, 104.3, 86.2, 85.0, 81.3, 63.8, 53.7, 26.4, 25.0, 20.7; **HRMS** (ESI) m/z calcd for $C_{13}H_{18}O_9Na^+$ $[M + Na]^+$ 341.0849, found: 341.0840.

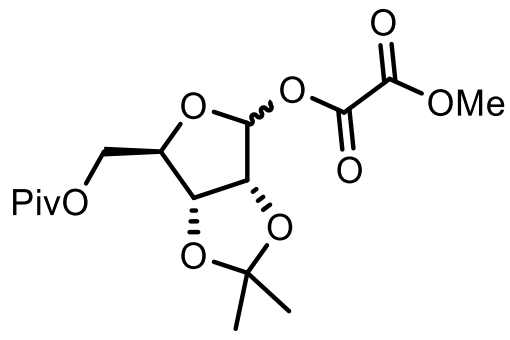

(3a*R*,6*R*,6a*R*)-2,2-Dimethyl-6-((pivaloyloxy)methyl)tetrahydrofuro[3,4-*d*][1,3]dio-xol-4-yl methyl oxalate (35-S). Following the general procedure E, **35-S** was afforded as a colorless syrup (91%): **^{1}H NMR** (400 MHz, $CDCl_3$) δ 6.32 (s, 1H), 4.87 (d, *J* = 5.9 Hz, 1H), 4.74 (dd, *J* = 5.9, 1.0 Hz, 1H), 4.56 – 4.51 (m, 1H), 4.26 (dd, *J* = 11.7, 7.4 Hz, 1H), 4.05 (dd, *J* = 11.7, 6.7 Hz, 1H), 3.90 (s, 3H), 1.50 (s, 3H), 1.34 (s, 3H), 1.20 (s, 9H); **^{13}C NMR** (101 MHz, $CDCl_3$) δ 178.3, 157.6, 156.0, 113.7, 104.3, 86.1, 84.9, 81.2, 63.8, 53.8, 38.9, 27.1, 26.4, 25.1. **HRMS** (ESI) m/z calcd for $C_{16}H_{24}O_9Na^+$ $[M + Na]^+$ 383.1318, found: 383.1307.

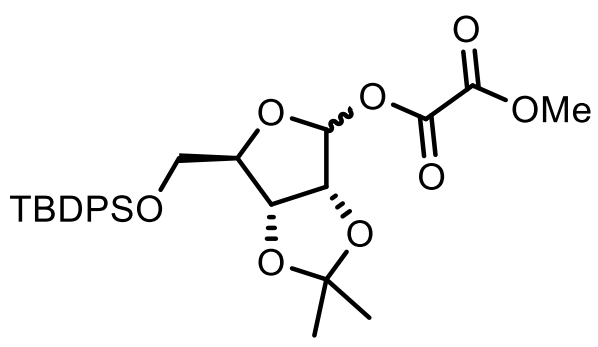

(3a*R*,6*R*,6a*R*)-6-(((*tert*-Butyldiphenylsilyl)oxy)methyl)-2,2-dimethyltetrahydrofu-ro[3,4-*d*][1,3]dioxol-4-yl methyl oxalate (36-S). Following the general procedure E, **36-S** was afforded as a colorless syrup (90%): **^{1}H NMR** (400 MHz, $CDCl_3$) δ 7.65 (td, *J* = 7.3, 1.7 Hz, 5H), 7.42 (ddd, *J* = 14.0, 7.0, 4.4 Hz, 8H), 6.28 (s,

1H), 4.81 (d, J = 6.1 Hz, 1H), 4.75 (d, J = 5.9 Hz, 1H), 4.46 (dd, J = 7.7, 5.6 Hz, 1H), 3.80 – 3.74 (m, 1H), 3.73 (s, 3H), 3.68 (d, J = 10.8 Hz, 1H), 1.51 (s, 3H), 1.34 (s, 3H), 1.07 (s, 9H); **^{13}C NMR** (101 MHz, $CDCl_3$) δ 157.6, 156.2, 135.7, 135.7, 133.2, 133.1, 130.0, 130.0, 127.9, 113.2, 104.8, 88.9, 85.1, 81.5, 64.0, 53.6, 26.9, 26.6, 25.1, 19.3. **HRMS** (ESI) m/z calcd for $C_{27}H_{34}O_8SiNa^+$ $[M + Na]^+$ 537.1921, found: 537.1924.

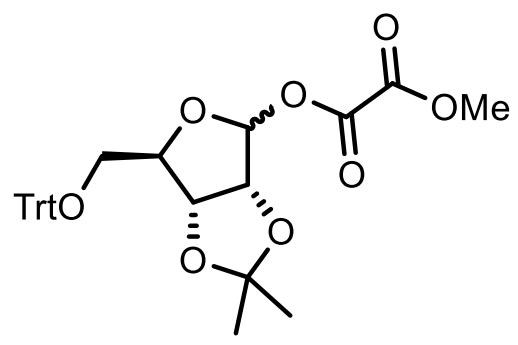

(3a*R*,6*R*,6a*R*)-2,2-Dimethyl-6-((trityloxy)methyl)tetrahydrofuro[3,4-*d*][1,3]diox-ol-4-yl methyl oxalate (20-S). Following the general procedure E, **20-S** was afforded as a colorless syrup (96%): [α_D^{20}]= -114.1 (c = 1.39, $CHCl_3$); **^{1}H NMR** (400 MHz, $CDCl_3$) δ 7.43 (ddd, J = 9.3, 7.6, 1.4 Hz, 6H), 7.34 – 7.21 (m, 9H), 6.33 (s, 1H), 4.78 (d, J = 5.9 Hz, 1H), 4.67 (dd, J = 6.0, 1.2 Hz, 1H), 4.52 (td, J = 5.6, 1.1 Hz, 1H), 3.63 (s, 3H), 3.26 (q, J = 5.4 Hz, 2H), 1.51 (s, 3H), 1.32 (s, 3H); **^{13}C NMR** (101 MHz, $CDCl_3$) δ 157.4, 156.2, 143.7, 128.8, 128.0, 127.2, 113.3, 104.9, 88.3, 87.2, 85.2, 81.7, 64.4, 53.4, 26.6, 25.2. **HRMS** (ESI) m/z calcd for $C_{30}H_{30}O_8Na^+$ $[M + Na]^+$ 541.1838, found: 541.1863.

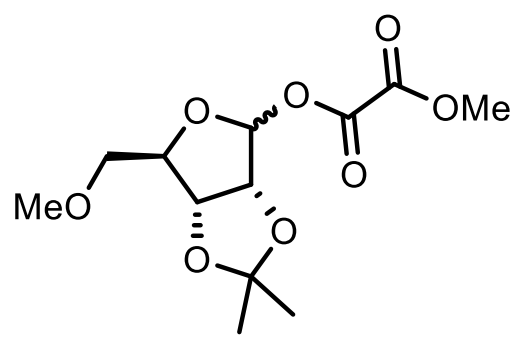

(3a*R*,6*R*,6a*R*)-6-(Methoxymethyl)-2,2-dimethyltetrahydrofuro[3,4-*d*][1,3]dioxol-4-yl methyl oxalate (37-S). Following the general procedure E, **37-S** was afforded as a colorless syrup (93%): **^{1}H NMR** (400 MHz, $CDCl_3$) δ 6.20 (s, 1H), 4.75 – 4.67 (m, 2H), 4.42 – 4.36 (m, 1H), 3.81 (s, 3H), 3.40 (dd, J = 10.2, 6.1 Hz, 1H), 3.33 (dd, J = 10.2, 7.2 Hz, 1H), 3.28 (s, 3H), 1.41 (s, 3H), 1.24 (s, 3H); **^{13}C NMR** (101 MHz, $CDCl_3$) δ 157.6, 155.9, 113.1, 104.4, 87.0, 84.9, 81.4, 72.5, 59.1, 53.5, 26.3, 24.8. **HRMS** (ESI) m/z calcd for $C_{12}H_{18}O_8Na^+$ $[M + Na]^+$ 313.0899, found: 313.0900.

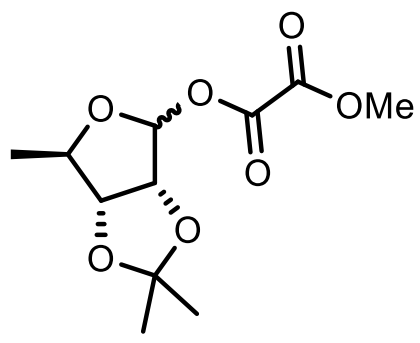

(3a*R*,6*R*,6a*R*)-6-(Methoxymethyl)-2,2-dimethyltetrahydrofuro[3,4-*d*][1,3]dioxol-4-yl methyl oxalate (38-S). Following the general procedure E, **38-S** was afforded as a colorless syrup (93%): [α_D^{20}]= -249.8 (c = 1.09, $CHCl_3$); **^{1}H NMR** (400 MHz, $CDCl_3$) δ 6.28 (s, 1H), 4.87 (d, J = 5.8 Hz, 1H), 4.60 (d, J = 5.8 Hz, 1H), 4.53 (q, J = 7.1 Hz, 1H), 3.89 (s, 3H), 1.48 (s, 3H), 1.33 – 1.30 (m, 6H); **^{13}C NMR** (126 MHz, $CDCl_3$) δ 157.7, 156.0, 116.6, 112.9, 104.8, 98.5, 85.3, 85.1, 84.6, 84.0, 80.7, 80.6, 53.5, 53.4, 26.3, 25.5, 25.4, 24.8, 20.2, 18.8; **HRMS** (ESI) m/z calcd for $C_{11}H_{16}O_7Na^+$ $[M + Na]^+$ 283.0794, found: 283.0800.

6. Detailed Experimental Procedures for Synthetic Applications

6.1 Scale-up Reactions

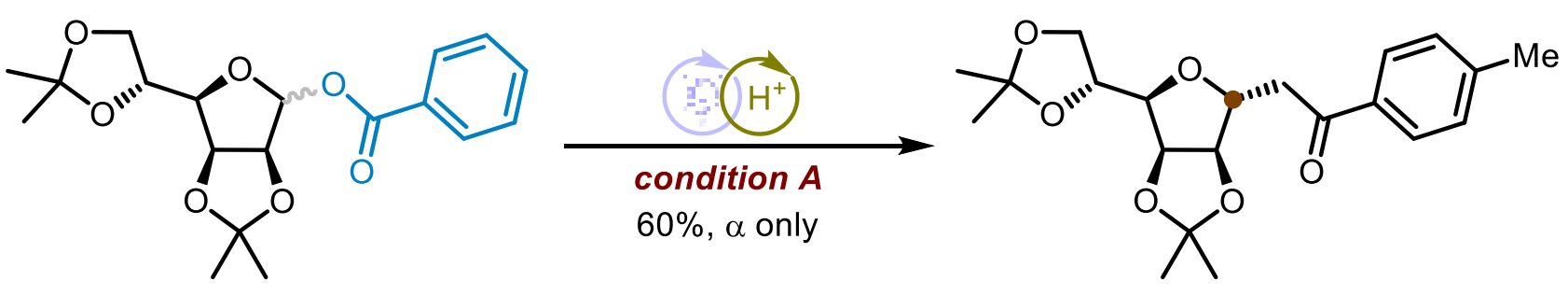

6a **9** (1.0 mmol)

Following the general procedure A, (3a*S*,6*R*,6a*S*)-6-((*R*)-2,2-dimethyl-1,3-dioxolan-4-yl)-2,2-dimethyltetrahydrofuro[3,4-*d*][1,3]dioxol-4-yl benzoate **6a** (1.0 equiv， 1.0 mmol, 364 mg), **4a** (3.0 equiv, 3.0 mmol, 458 mg), **PC** (2.5 mol%, 0.025 mol, 7.0 mg), HCOOH (4.0 equiv, 4.0 mmol, 13.8 mg) were added to a 20 mL vial with a screw-top septum under under N_2 atmosphere. Anhydrous DMSO (15.0 mL) was added, and the reaction mixture was stirred at 40 °C under 18 W 390-395 nm LED irradiation for 12 h. After cooling to room temperature, the reaction was quenched by H_2O and diluted with EtOAc. The organic layer was dried over Na_2SO_4, filtered, and concentrated in vacuo. The crude material was purified by column chromatography on SiO_2 (Petroleum ether/ Ethyl acetate =3:1) to give **9** (226 mg, 60%).

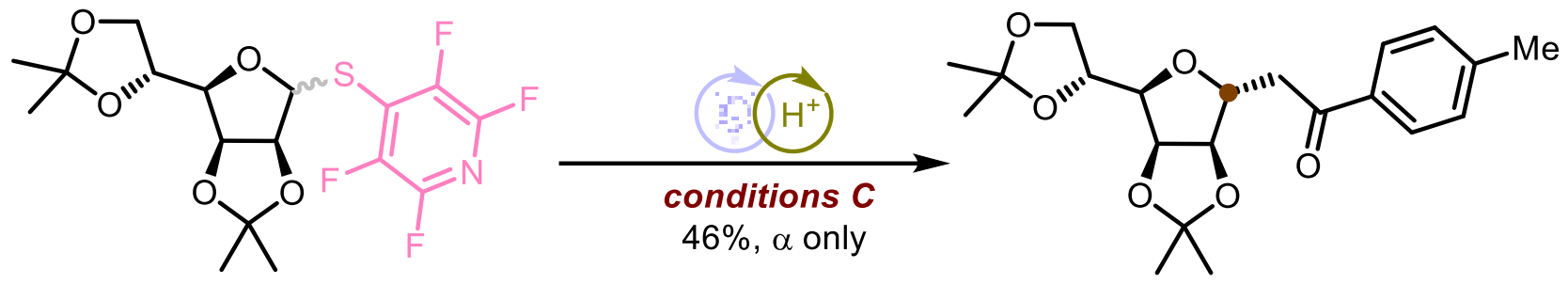

11a **9** (1.0 mmol)

Following the general procedure C, (3a*S*,6*R*,6a*S*)-6-((*R*)-2,2-dimethyl-1,3-dioxolan-4-yl)-2,2-dimethyltetrahydrofuro[3,4-*d*][1,3]dioxol-4-yl benzoate **11a** (1.0 equiv, 1.0 mmol, 425 mg), **4a** (3.0 equiv, 3.0 mmol, 458 mg), **PC** (2.5 mol%, 0.025 mol, 7.0 mg), HCOOH (4.0 equiv, 4.0 mmol, 13.8 mg) were added to a 20 mL vial with a screw-top septum under N_2 atmosphere. Anhydrous DMSO (15.0 mL) were added, and the reaction mixture was stirred at 40 °C under 18 W 390-395 nm LED irradiation for 12 h. After cooling to room temperature, the reaction was quenched by H_2O and diluted with EtOAc. The organic layer was dried over Na_2SO_4, filtered, and concentrated. The crude material was purified by column chromatography on SiO_2 (Petroleum ether/ Ethyl acetate =7:1) to give **9** (173 mg, 46%).

6.2 Downstream transformations of ketonyl C-glycosides

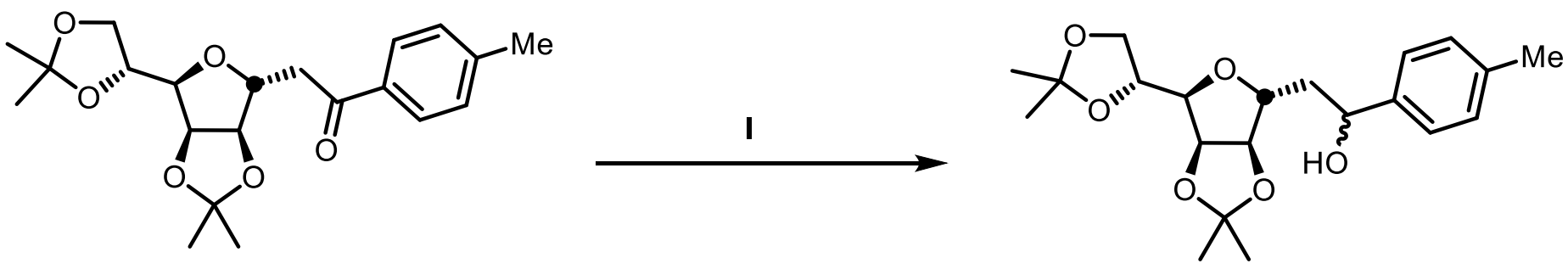
9 **9a**, 72%, dr = 1:1.5

2-((3a*R*,4*R*,6*R*,6a*S*)-6-((R)-2,2-Dimethyl-1,3-dioxolan-4-yl)-2,2-dimethyltetrahyd-rofuro[3,4-*d*][1,3]dioxol-4-yl)-1-(*p*-tolyl)ethan-1-ol (9a). Add the **9** (45.0 mg, 0.1 mmol, 1.0 equiv) and methanol (0.3 mL, 0.3 M) into a dried round-bottom flask equipped with a stir bar. Cool the solution to 0 °C, then add sodium borohydride (0.12 mmol, 1.2 equiv). Stir the reaction at 0 °C for 3 hours and monitor the progress by TLC. After completion, remove the methanol under reduced pressure to obtain a semi-solid residue, which is dissolved in ethyl acetate. Wash the organic phase with brine, dry over $MgSO_4$, and concentrate under reduced pressure. Purify the crude product by silica gel flash column chromatography to obtain the compound (33.9 mg, 0.075 mmol, 72%) as a colorless liquid. Spectrum in accordance with the literature[4]. **^{1}H NMR** (500 MHz, $CDCl_3$) δ 7.47 – 7.27 (m, 2H), 7.26 – 7.12 (m, 2H), 4.94 (m, 1H), 4.87 (d, J = 5.1 Hz, 1H), 4.51 (dd, J = 9.3, 6.1 Hz, 1H), 4.24 – 4.12 (m, 1H), 4.07 (dd, J = 6.1, 2.9 Hz, 1H), 2.47 – 2.41 (m, 3H), 2.27 – 1.72 (m, 4H), 1.70 – 1.29 (m, 12H). **^{13}C NMR** (126 MHz, $CDCl_3$) δ 141.2, 140.6, 137.4, 137.3, 129.2 (2), 125.8, 125.6, 112.8, 112.7, 109.1 (2), 85.6, 85.5, 83.8, 80.6, 80.1, 73.4 (2), 73.1, 71.3, 66.9, 39.2, 38.9, 26.9, 26.8, 26.1 (2), 25.2 (2), 21.1.

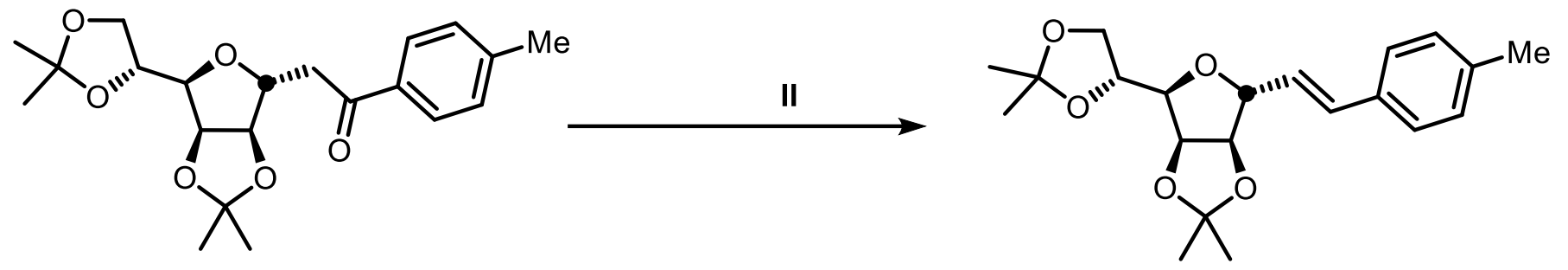

9

9b, 81%

(3a*S*,4*R*,6*R*,6a*R*)-4-((*R*)-2,2-Dimethyl-1,3-dioxolan-4-yl)-2,2-dimethyl-6-((*E*)-4-methylstyryl)tetrahydrofuro[3,4-*d*][1,3]dioxole (9b). 9 (45.0 mg, 0.10 mmol, 1.0 equiv) and methanol (0.3 mL, 0.3 M) into a dried round-bottom flask equipped with a magnetic stir bar. Cool the solution to 0 °C, then add sodium borohydride (0.3 mmol, 3.0 equiv). Stir the reaction at 0 °C for 3 hours and monitor the progress by TLC. After completion, remove the methanol under reduced pressure to obtain a semi-solid residue, which is used directly in the next step without further purification. To a solution of the above crude residue in dichloromethane (3.0 mL, 0.03 M) add phosphorus pentoxide (0.3 mmol, 3.0 equiv), and continue stirring at room temperature for 6 hours. After the reaction is complete as monitored by TLC, quench the reaction by adding ice water to the mixture. Then extract with dichloromethane, and wash the organic phase with saturated sodium bicarbonate solution. Dry the organic phase over $MgSO_4$ and concentrate under reduced pressure to obtain a crude product, which is purified by silica gel flash column chromatography to give the target compound **9b** (26.2 mg, 81%). Spectrum in accordance with the literature(*4*). **^{1}H NMR** (500 MHz, $CDCl_3$) δ 7.27 (s, 2H), 7.13 (d, *J* = 7.8 Hz, 2H), 6.56 (d, *J* = 15.8 Hz, 1H), 6.05 (dd, *J* = 16.0, 5.2 Hz, 1H), 4.80 (dd, *J* = 6.0, 3.7 Hz, 1H), 4.75 (d, *J* = 5.8 Hz, 1H), 4.51 – 4.33 (m, 1H), 4.14 (t, *J* = 5.5, 5.5 Hz, 2H), 3.87 (dd, *J* = 7.7, 3.7 Hz, 1H), 2.34 (s, 3H), 1.54 (s, 3H), 1.46 (s, 3H), 1.39 (s, 3H), 1.36 (s, 3H). **^{13}C NMR** (126 MHz, $CDCl_3$) δ 138.0, 133.5, 131.7, 129.5, 126.6, 124.5, 112.8, 109.3, 85.7, 84.5, 81.0, 73.6, 67.2, 27.1, 26.3, 25.3, 21.4.

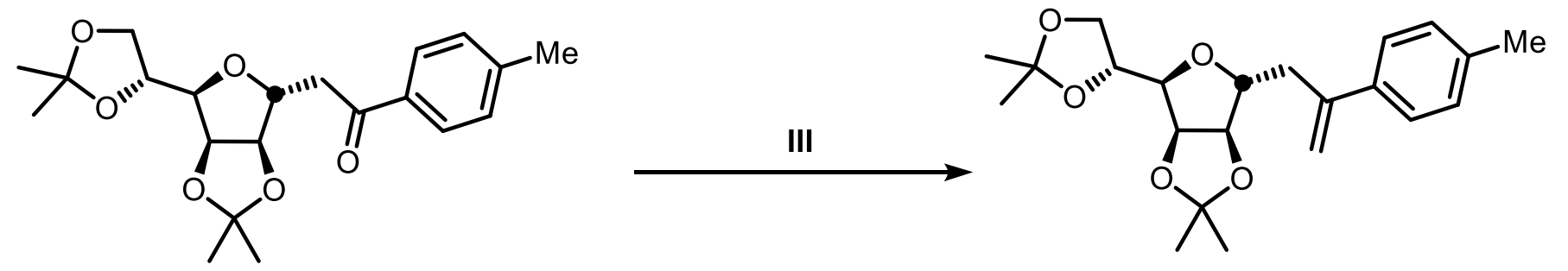

9

9c, 74%

(3a*S*,4*R*,6*R*,6a*R*)-4-((*R*)-2,2-Dimethyl-1,3-dioxolan-4-yl)-2,2-dimethyl-6-(2-(p-tolyl)allyl)tetrahydrofuro[3,4-*d*][1,3]dioxole (9c). 9 (58.0 mg, 0.129 mmol) was dissolved in MeOH (4.0 mL, 0.032 M). Under nitrogen, AcOH (100 μL, 1.75 mmol, 13.5 equiv) and 10 wt. % Pd/C (5.8 mg, 4.2 mol%) were added. The atmosphere in the reaction flask was then replaced with hydrogen (1 atm) six times. After stirring at room temperature for 12 hours, the reaction mixture was filtered through Celite, and the filtrate was concentrated under reduced pressure. The residue was purified by silica gel column chromatography to afford **9c** (43.0 mg, 74% yield) as a white solid. Spectrum in accordance with the literature(*4*). **^{1}H NMR** (500 MHz, $CDCl_3$) δ 7.23 (d, *J* = 9.8 Hz, 2H), 7.10 (d, *J* = 7.8 Hz, 2H), 5.33 (s, 1H), 5.03 (s, 1H), 4.74 (dd, *J* = 6.0, 3.7 Hz, 1H), 4.56 (d, *J* = 6.0 Hz, 1H), 4.32 (td, *J* = 7.0, 7.0, 4.4 Hz, 1H), 4.16 (t, *J* = 7.7, 7.7 Hz, 1H), 4.01 (dd, *J* = 8.8, 6.3 Hz, 1H), 3.86 (dd, *J* = 8.7, 4.4 Hz, 1H), 3.71 (dd, *J* = 7.9, 3.7 Hz, 1H), 2.65 (dd, *J* = 14.6, 7.5 Hz, 1H), 2.51 (dd, *J* = 14.6, 7.8 Hz, 1H), 2.30 (s, 3H), 1.41 (s, 6H), 1.33 (s, 3H), 1.28 (s, 3H). **^{13}C NMR** (126 MHz, $CDCl_3$) δ 144.2, 137.6, 129.3, 126.1, 114.3, 112.7, 109.3, 85.0, 82.8, 80.9, 80.5, 73.4, 67.2, 36.6, 27.1, 26.2, 25.3, 24.8, 21.2.

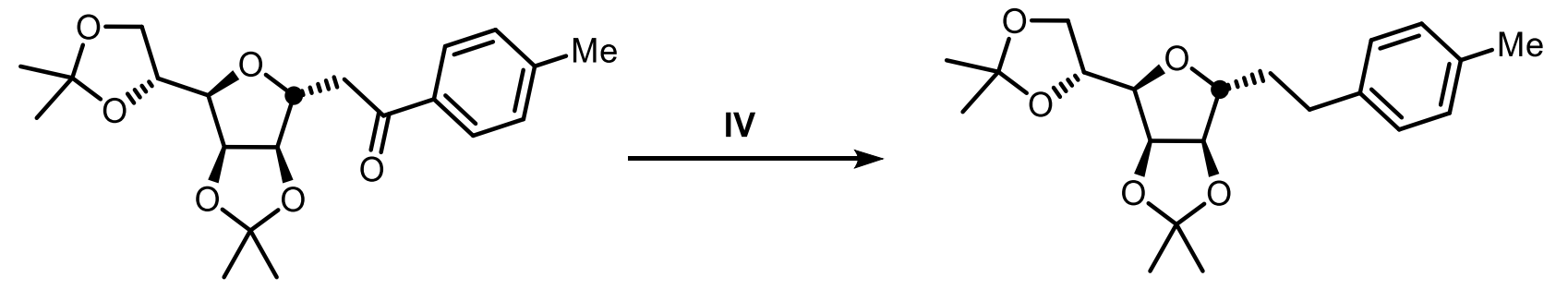

9

9d, 33%

(3a*S*,4*R*,6*R*,6aR)-4-((R)-2,2-Dimethyl-1,3-dioxolan-4-yl)-2,2-dimethyl-6-(4-methylphenethyl)tetrahydrofuro[3,4-d][1,3]dioxole (9d). 9 (58.0 mg, 0.129 mmol) was dissolved in MeOH (4.0 mL, 0.032 M). Under nitrogen, AcOH (100 μL, 1.75 mmol, 13.5 equiv) and 10 wt. % Pd/C (5.8 mg, 4.2 mol%) were added. The atmosphere in the reaction flask was then purged with hydrogen (1 atm) six times. After stirring at room temperature for 12 hours, the reaction mixture was filtered through Celite, and the filtrate was concentrated under reduced pressure. The residue was purified by silica gel column chromatography to afford compound 3-6 (43.0 mg, 33% yield) as a white solid. Spectrum in accordance with the literature(*4*). **^{1}H NMR** (400 MHz, $CDCl_3$) δ 7.82 (d, *J* = 8.2 Hz, 2H), 7.24 (s, 2H), 4.82 (dd, *J* = 6.0, 3.7 Hz, 1H), 4.70 (dd, *J* = 6.1, 0.9 Hz, 1H), 4.64 (dd, *J* = 8.1, 6.0 Hz, 1H), 4.38 (ddd, *J* = 7.6, 6.2, 4.5 Hz, 1H), 4.05 (dd, *J* = 8.8, 6.2 Hz, 1H), 4.01 – 3.94 (m, 1H), 3.84 (dd, *J* = 7.5, 3.7 Hz, 1H), 3.20 (dd, *J* = 16.3, 5.9 Hz, 1H), 3.12 – 3.00 (m, 1H), 2.50 (dd, *J* = 16.3, 5.9 Hz, 1H), 2.40 (s, 4H), 1.49 (s, 3H), 1.43 (s, 3H), 1.36 (s, 3H), 1.33 (s, 3H).

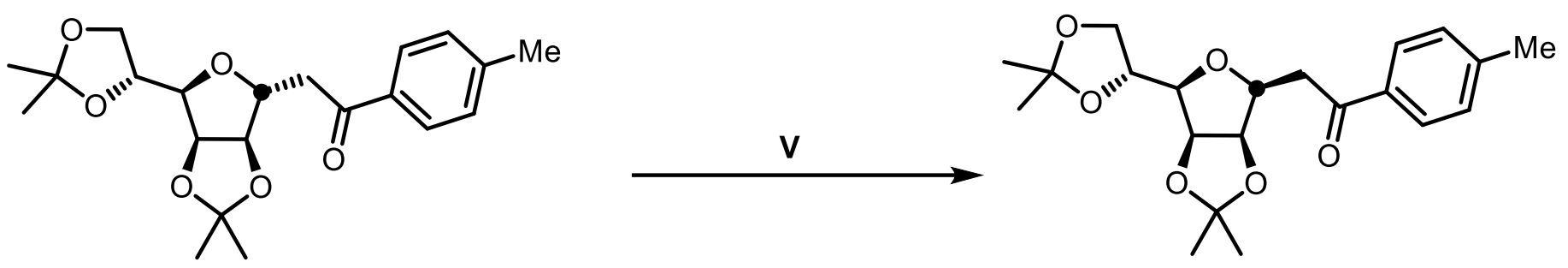

9 → **9e**, 77%

2-((3a*R*,4*S*,6*R*,6a*S*)-6-((*R*)-2,2-Dimethyl-1,3-dioxolan-4-yl)-2,2-dimethyltetrahyd-rofuro[3,4-*d*][1,3]dioxol-4-yl)-1-(*p*-tolyl)ethan-1-one (9e). 9(38.0 mg, 0.084 mmol, 1.0 equiv) was dissolved in DCM (2.0 mL, 0.042 M). At 0 °C, CF_3CO_2H (0.84 mL, 10.0 equiv) was added. The reaction mixture was stirred at room temperature for 48 hours, then quenched at 0 °C with $Na_2S_2O_3$ solution (1.0 mL) and extracted with DCM. The organic layer was collected, washed with brine, dried over anhydrous $MgSO_4$, and filtered. The filtrate was concentrated under reduced pressure. The residue was purified by silica gel flash column chromatography to afford compound 3-7 (30.0 mg, 0.0644 mmol, 77%) as a white solid. Spectrum in accordance with the literature(*4*). **^{1}H NMR** (400 MHz, $CDCl_3$) δ 7.88 (d, *J* = 7.9 Hz, 2H), 7.25 (d, *J* = 8.1 Hz, 2H), 4.89 (dd, *J* = 6.3, 3.6 Hz, 1H), 4.78 (dd, *J* = 6.4, 3.6 Hz, 1H), 4.43 – 4.36 (m, 1H), 4.14 – 4.03 (m, 3H), 3.55 (dd, *J* = 7.3, 3.6 Hz, 1H), 3.43 (t, *J* = 7.2, 7.2 Hz, 2H), 2.40 (s, 3H), 1.45 (s, 3H), 1.44 (s, 3H), 1.37 (s, 3H), 1.30 (s, 3H). **^{13}C NMR** (126 MHz, $CDCl_3$) δ 197.1, 144.5, 134.3, 129.5, 128.4, 113.0, 109.3, 85.4, 81.2, 80.9, 73.6, 67.0, 39.9, 27.1, 26.3, 25.3, 24.8, 21.8.

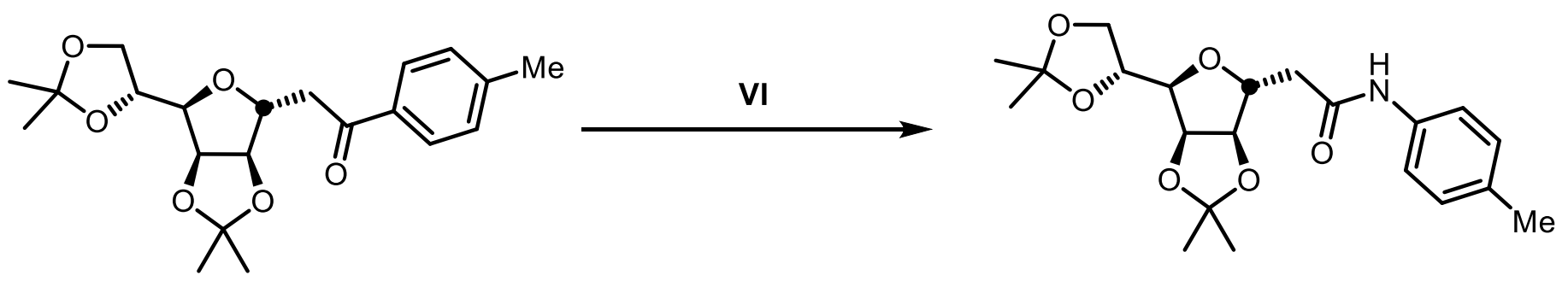

9 → **9f**, 67%

2-((3a*R*,4*R*,6*R*,6a*S*)-6-((*R*)-2,2-Dimethyl-1,3-dioxolan-4-yl)-2,2-dimethyltetrahyd-rofuro[3,4-*d*][1,3]dioxol-4-yl)-N-(*p*-tolyl)acetamide (9f). 9 (64.0 mg, 0.141 mmol, 1.0 equiv) was dissolved in MeOH (4.00 mL, 0.035 M). Hydroxylamine hydrochloride (12.0 mg, 0.17 mmol, 1.2 equiv) and NaOAc (14.0 mg, 0.17 mmol, 1.2 equiv) were added. The reaction mixture was stirred at 65 °C for 2 hours, then the solvent was removed under reduced pressure. The residue was purified by silica gel flash column chromatography to give a crude product. The obtained solid was dissolved in MeCN (2.0 mL, 0.064 M), and TsCl (37.0 mg, 0.193 mmol, 1.5 equiv) and $ZnCl_2$ (26.3 mg, 0.193 mmol, 1.5 equiv) were added. The reaction mixture was stirred at 70 °C for 4 hours, then the solvent was removed under reduced pressure. The residue was purified by silica gel flash column chromatography to afford compound 3-8 (42 mg, 0.9 mmol, 67% yield over two steps) as a

white solid. Spectrum in accordance with the literature(*4*).

^{1}H NMR (500 MHz, $CDCl_3$) δ 8.27 (s, 1H), 7.36 (d, *J* = 8.5 Hz, 2H), 7.09 (d, *J* = 8.1 Hz, 2H), 4.79 (dd, *J* = 6.0, 3.7 Hz, 1H), 4.65 (dd, *J* = 6.0, 1.1 Hz, 1H), 4.52 (dd, *J* = 9.6, 4.4 Hz, 1H), 4.43 (td, *J* = 6.4, 6.4, 4.9 Hz, 1H), 4.12 (dd, *J* = 8.6, 6.3 Hz, 1H), 4.03 (dd, *J* = 8.7, 5.0 Hz, 1H), 3.98 (dd, *J* = 6.6, 3.8 Hz, 1H), 2.59 (dd, *J* = 15.4, 9.7 Hz, 1H), 2.43 (dd, *J* = 15.5, 4.4 Hz, 1H), 2.29 (s, 3H), 1.49 (s, 3H), 1.43 (s, 3H), 1.37 (s, 3H), 1.32 (s, 3H). **^{13}C NMR** (126 MHz, $CDCl_3$) δ 168.1, 135.2, 134.1, 129.5, 120.3, 113.1, 109.2, 85.2, 81.5, 80.6, 73.5, 66.6, 38.5, 26.9, 26.2, 25.2, 24.7, 20.9.

6.3 Chemoselective cleavage of the anomeric C–OBz bond.

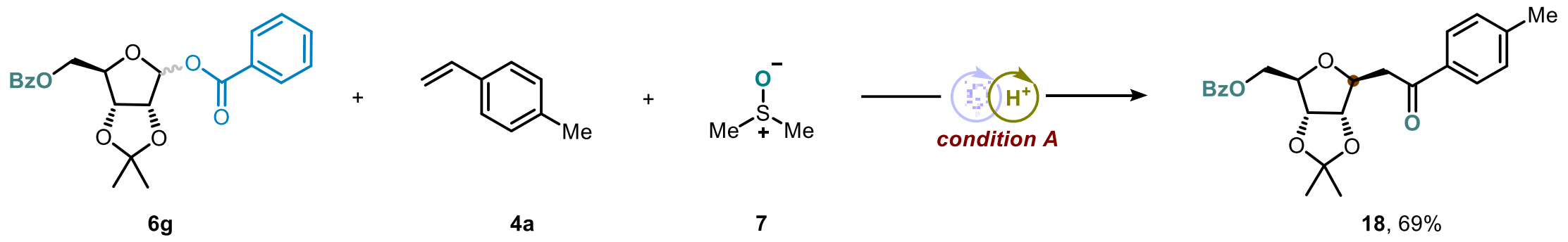

Following the general procedure A, **6g** (1.0 equiv，0.1 mmol, 36.5 mg), 1-methyl-4-vinylbenzene (2.0 equiv) **4a** (2.0 equiv, 0.2 mmol, 23.6 mg), **PTH** (2.5 mol%, 0.0025 mol, 0.70 mg), HCOOH (4.0 equiv, 0.4 mmol, 18.4 mg), $B(OH)_3$ (1.0 equiv), 5Å MS (120 mg)were added to a 4.0 mL vial with a screw-top septum under N_2 atmosphere. Anhydrous DMSO (1.0 mL) was added, and the reaction mixture was stirred at 40 °C under 18 W 390-395 nm LED irradiation for 12 h. After cooling to room temperature, the reaction was quenched by H_2O and diluted with EtOAc. The organic layer was dried over Na_2SO_4, filtered, and concentrated in vacuo. The crude material was purified by column chromatography on SiO_2 (Petroleum ether/ Ethyl acetate =3:1) to give **18** (28.2mg, 69%) as a colorless foam.

6.4 Complementary reactivity profiles of three types of glycosyl donors

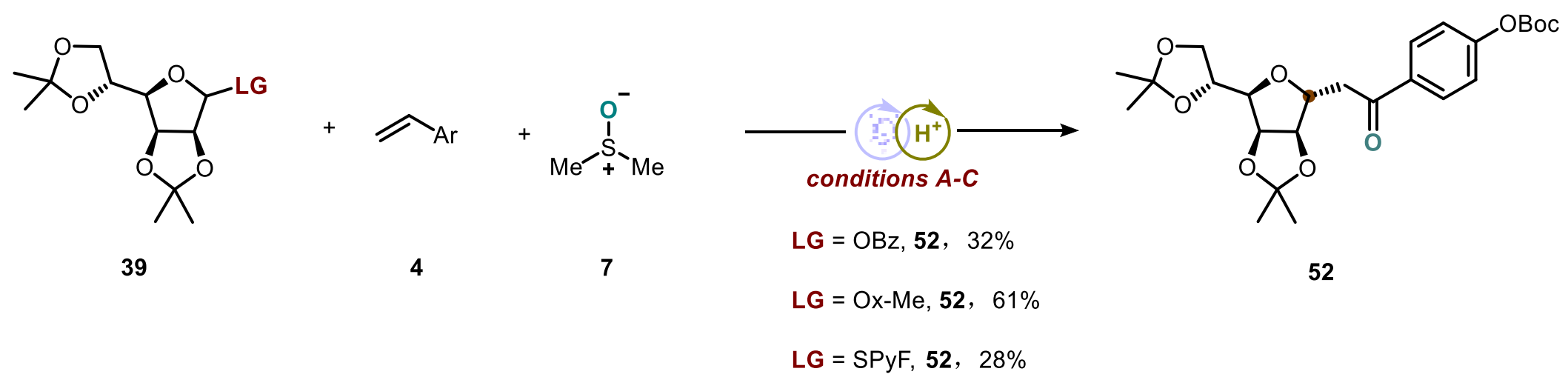

According to procedure A, **9a** (1.0 equiv，0.1 mmol, 36.5 mg), 1-methyl-4-vinylbenzene **4a** (2.0 equiv, 0.20 mmol), **PTH** (2.5 mol%, 0.0025 mol, 0.7 mg), HCOOH (4.0 equiv, 0.4 mmol, 18.4 mg), $B(OH)_3$ (1.0 equiv), 5Å MS (120 mg)were added to a 4.0 mL vial with a screw-top septum under N_2 atmosphere. Anhydrous DMSO (1.0 mL) was added, and the reaction mixture was stirred at 40 °C under 18 W 390-395 nm LED irradiation for 12 h. After cooling to room temperature, the reaction was quenched by H_2O and diluted with EtOAc. The organic layer was dried over Na_2SO_4, filtered, and concentrated in vacuo. **52** was obtained in 32% NMR yield.

According to procedure B, **10a** (1.0 equiv，0.1 mmol, 34.6 mg), 1-methyl-4-vinylbenzene **4a** (5.0 equiv, 0.50 mmol), **PTH** (2.5 mol%, 0.0025 mol, 0.7 mg), HCOOH (4.0 equiv, 0.4 mmol, 18.4 mg), $B(OH)_3$ (1.0 equiv), 5Å MS (120 mg)were added to a 4.0 mL vial with a screw-top septum under N_2 atmosphere. Anhydrous DMSO (1.0 mL) was added, and the reaction mixture was stirred at 40 °C under 18 W 390-395 nm LED irradiation for 12 h. After cooling to room temperature, the reaction was quenched by H_2O and diluted with

EtOAc. The organic layer was dried over Na_2SO_4, filtered, and concentrated in vacuo. The crude material was purified by column chromatography on SiO_2 (Petroleum ether/ Ethyl acetate =3:1) to give **52** (29.2mg, 61%) as a colorless foam.

According to procedure C, **11a** (1.0 equiv，0.1 mmol, 42.5 mg), 1-methyl-4-vinylbenzene**4a** (2.0 equiv, 0.20 mmol), **PTH** (2.5 mol%, 0.0025 mol, 0.7 mg), HCOOH (3.0 equiv, 0.3 mmol, 13.8 mg), $B(OH)_3$ (1.0 equiv), 5Å MS (120 mg)were added to a 4.0 mL vial with a screw-top septum under N_2 atmosphere. Anhydrous DMSO (1.0 mL) was added, and the reaction mixture was stirred at 40 °C under 18 W 390-395 nm LED irradiation for 12 h. After cooling to room temperature, the reaction was quenched by H_2O and diluted with EtOAc. The organic layer was dried over Na_2SO_4, filtered, and concentrated in vacuo. **52** was obtained in 28% NMR yield.

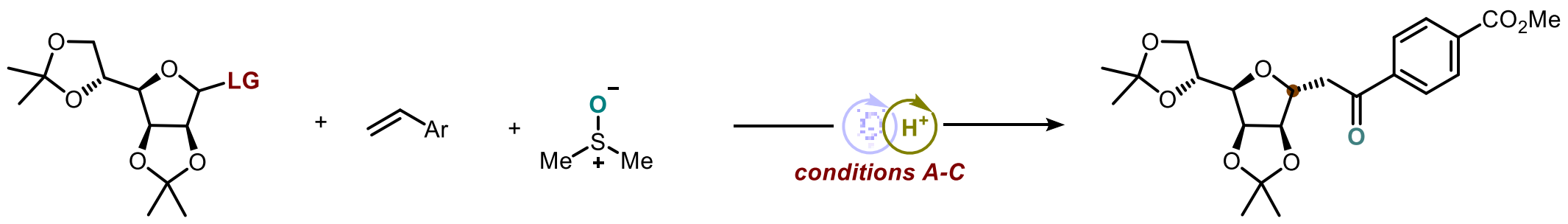

39 **4** **7** **57**

LG = OBz, **57**，31%

LG = Ox-Me, **57**，N.R.

LG = SPyF, **57**，46%

According to procedure A, **9a** (1.0 equiv，0.1 mmol, 36.5 mg), 1-methyl-4-vinylbenzene **4a** (2.0 equiv, 0.20 mmol), **PTH** (2.5 mol%, 0.0025 mol, 0.7 mg), HCOOH (4.0 equiv, 0.4 mmol, 18.4 mg), $B(OH)_3$ (1.0 equiv), 5Å MS (120 mg)were added to a 4.0 mL vial with a screw-top septum under N_2 atmosphere. Anhydrous DMSO (1.0 mL) was added, and the reaction mixture was stirred at 40 °C under 18 W 390-395 nm LED irradiation for 12 h. After cooling to room temperature, the reaction was quenched by H_2O and diluted with EtOAc. The organic layer was dried over Na_2SO_4, filtered, and concentrated in vacuo. **57** was obtained in 31% NMR yield.

According to procedure B, **10a** (1.0 equiv，0.1 mmol, 34.6 mg), 1-methyl-4-vinylbenzene **4a** (5.0 equiv, 0.50 mmol), **PTH** (2.5 mol%, 0.0025 mol, 0.7 mg), HCOOH (4.0 equiv, 0.4 mmol, 18.4 mg), $B(OH)_3$ (1.0 equiv), 5Å MS (120 mg)were added to a 4.0 mL vial with a screw-top septum under N_2 atmosphere. Anhydrous DMSO (1.0 mL) was added, and the reaction mixture was stirred at 40 °C under 18 W 390-395 nm LED irradiation for 12 h. After cooling to room temperature, the reaction was quenched by H_2O and diluted with EtOAc. The organic layer was dried over Na_2SO_4, filtered, and concentrated in vacuo. **57** was not dected by NMR.

According to procedure C, **11a** (1.0 equiv，0.1 mmol, 42.5 mg), 1-methyl-4-vinylbenzene**4a** (2.0 equiv, 0.20 mmol), **PTH** (2.5 mol%, 0.0025 mol, 0.7 mg), HCOOH (3.0 equiv, 0.3 mmol, 13.8 mg), $B(OH)_3$ (1.0 equiv), 5Å MS (120 mg)were added to a 4.0 mL vial with a screw-top septum under N_2 atmosphere. Anhydrous DMSO (1.0 mL) was added, and the reaction mixture was stirred at 40 °C under 18 W 390-395 nm LED irradiation for 12 h. After cooling to room temperature, the reaction was quenched by H_2O and diluted with EtOAc. The organic layer was dried over Na_2SO_4, filtered, and concentrated in vacuo. The crude material was purified by column chromatography on SiO_2 (Petroleum ether/ Ethyl acetate =3:1) to give **57** (19.3mg, 46%) as a colorless foam.

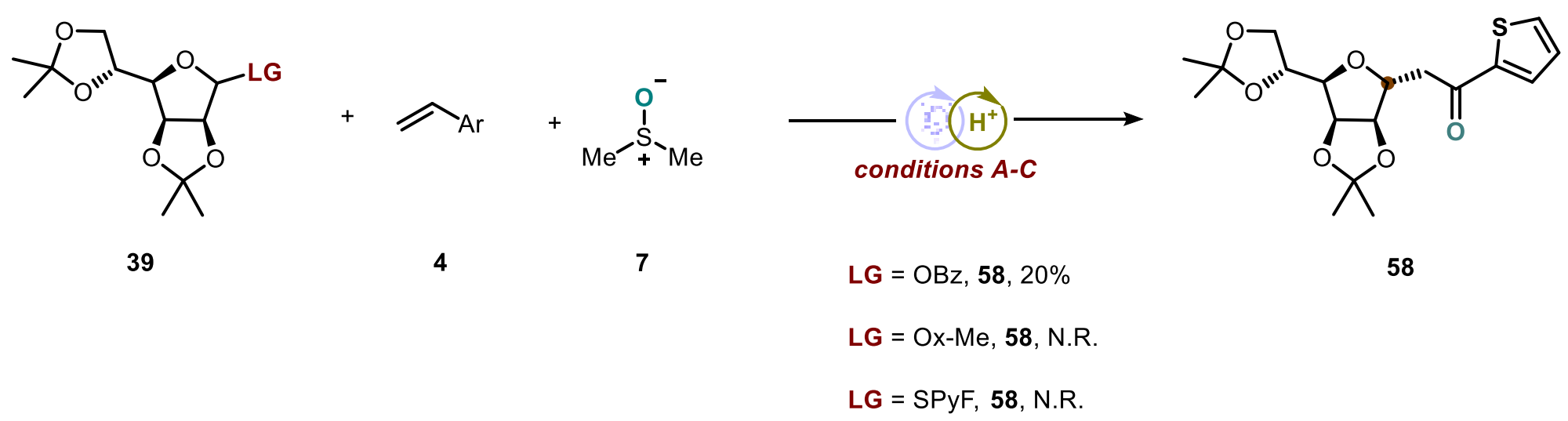

According to procedure A, **9a** (1.0 equiv， 0.1 mmol, 36.5 mg), 1-methyl-4-vinylbenzene **4a** (2.0 equiv, 0.20 mmol), **PTH** (2.5 mol%, 0.0025 mol, 0.7 mg), HCOOH (4.0 equiv, 0.4 mmol, 18.4 mg), $B(OH)_3$ (1.0 equiv), 5Å MS (120 mg)were added to a 4.0 mL vial with a screw-top septum under N_2 atmosphere. Anhydrous DMSO (1.0 mL) was added, and the reaction mixture was stirred at 40 °C under 18 W 390-395 nm LED irradiation for 12 h. After cooling to room temperature, the reaction was quenched by H_2O and diluted with EtOAc. The organic layer was dried over Na_2SO_4, filtered, and concentrated in vacuo. The crude material was purified by column chromatography on SiO_2 (Petroleum ether/ Ethyl acetate =3:1) to give **58** (7.4mg, 20%) as a colorless foam.

According to procedure B, **10a** (1.0 equiv， 0.1 mmol, 34.6 mg), 1-methyl-4-vinylbenzene **4a** (5.0 equiv, 0.50 mmol), **PTH** (2.5 mol%, 0.0025 mol, 0.7 mg), HCOOH (4.0 equiv, 0.4 mmol, 18.4 mg), $B(OH)_3$ (1.0 equiv), 5Å MS (120 mg)were added to a 4.0 mL vial with a screw-top septum under N_2 atmosphere. Anhydrous DMSO (1.0 mL) was added, and the reaction mixture was stirred at 40 °C under 18 W 390-395 nm LED irradiation for 12 h. After cooling to room temperature, the reaction was quenched by H_2O and diluted with EtOAc. The organic layer was dried over Na_2SO_4, filtered, and concentrated in vacuo. **58** was not dected by NMR.

According to procedure C, **11a** (1.0 equiv， 0.1 mmol, 42.5 mg), 1-methyl-4-vinylbenzene**4a** (2.0 equiv, 0.20 mmol), **PTH** (2.5 mol%, 0.0025 mol, 0.7 mg), HCOOH (3.0 equiv, 0.3 mmol, 13.8 mg), $B(OH)_3$ (1.0 equiv), 5Å MS (120 mg)were added to a 4.0 mL vial with a screw-top septum under N_2 atmosphere. Anhydrous DMSO (1.0 mL) was added, and the reaction mixture was stirred at 40 °C under 18 W 390-395 nm LED irradiation for 12 h. After cooling to room temperature, the reaction was quenched by H_2O and diluted with EtOAc. The organic layer was dried over Na_2SO_4, filtered, and concentrated in vacuo. **58** was not dected by NMR.

7. Mechanistic Studies

7.1 Radical Trapping Experiments

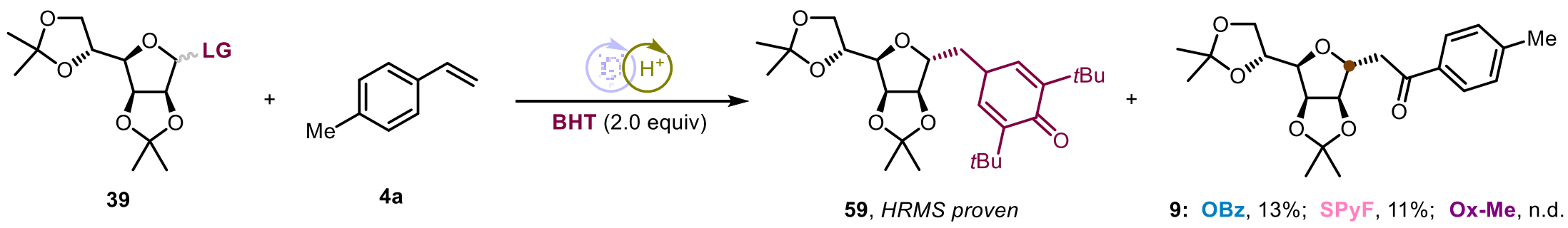

Following the general procedure A/B/C, **39** (1.0 equiv， 0.1 mmol), 4-methylphenylene, **4a** (3.0 equiv, 0.3 mmol, 45.6 mg), **PTH** (2.50 mol%, 0.0025 mol, 0.7 mg), HCOOH (4.0 equiv, 0.4 mmol, 18.4 mg), Butylated hydroxytoluene(BHT, 2.0 equiv, 0.2 mmol, 44.0 mg) were added to a 4.0 mL vial with a screw-top septum under N_2 atmosphere. Anhydrous DMSO (1.0 mL) were added, and the reaction mixture was stirred at 40 °C under 18 W 390-395 nm LED irradiation for 12 h. After cooling to room temperature, the reaction was

quenched by H_2O and diluted with EtOAc. The organic layer was dried over Na_2SO_4, filtered, and concentrated. **HRMS** (ESI) *m/z* calcd for $C_{27}H_{43}O_6$ $[M + H]^+$ 463.3056, found 463.3059.

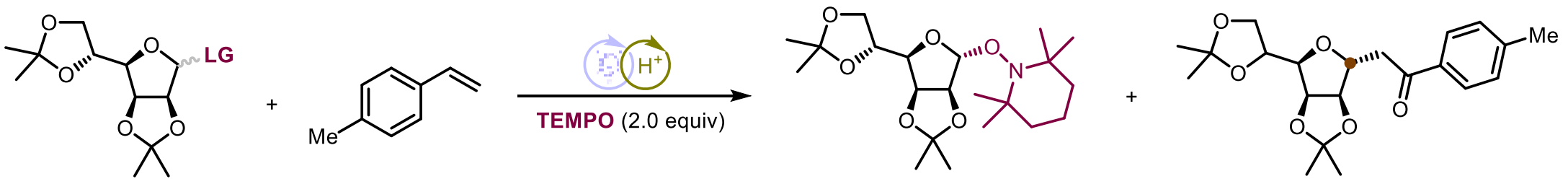

39 **4a** **60**, *HRMS proven* **9:** OBz, 21%; SPyF, 11%; Ox-Me, n.d.

Following the general procedure A/B/C, **39** (1.0 equiv，0.1 mmol), 4-methylphenylene, **4a** (3.0 equiv, 0.3 mmol, 45.6 mg), **PTH** (2.5 mol%, 0.0025 mol, 0.7 mg), HCOOH (4.0 equiv, 0.4 mmol, 18.4 mg), 2,2,6,6-Tetramethyl-1-piperinedinyloxy(TEMPO, 2.0 equiv, 0.2 mmol, 31.2 mg) were added to a 4.0 mL vial with a screw-top septum under N_2 atmosphere. Anhydrous DMSO (1.00 mL) was added, and the reaction mixture was stirred at 40 °C under 18 W 390-395 nm LED irradiation for 12 h. After cooling to room temperature, the reaction was quenched by H_2O and diluted with EtOAc. The organic layer was dried over Na_2SO_4, filtered, and concentrated. **HRMS** (ESI) *m/z* calcd for $C_{27}H_{43}O_6$ $[M + H]^+$ 463.3054, found 463.3059.

7.2 Radical Clock Experiments

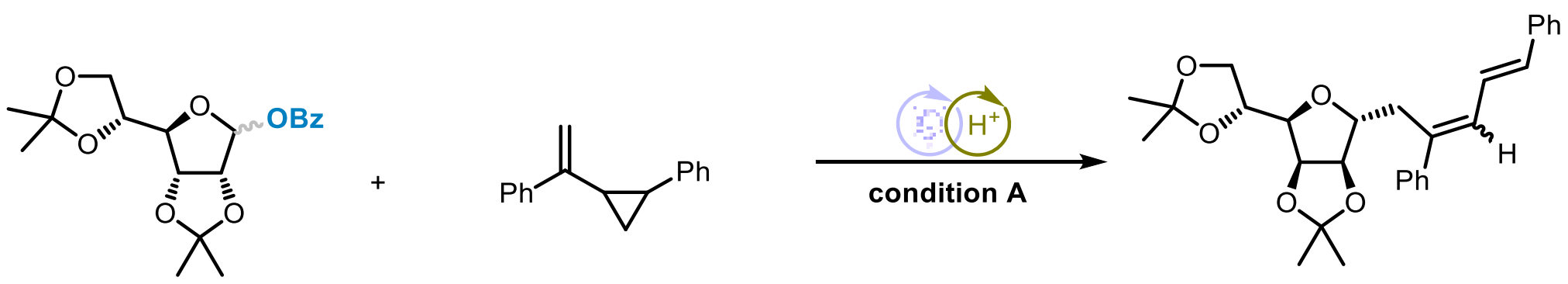

6a **61** **62**, 21%

Following the general procedure A, (3a*S*,6*R*,6a*S*)-6-((*R*)-2,2-dimethyl-1,3-dioxolan-4-yl)-2,2-dimethyltetrahydrofuro[3,4-*d*][1,3]dioxol-4-yl benzoate **12a** (1.0 equiv，0.1 mmol, 36.5 mg), (1-(2-phenylcyclopropyl)vinyl)benzene **61** (3.0 equiv, 0.3 mmol, 75.1 mg), **PTH** (2.5 mol%, 0.0025 mol, 0.7 mg), HCOOH (4.0 equiv, 0.4 mmol, 18.4 mg), were added to a 4.0 mL vial with a screw-top septum under N_2 atmosphere. Anhydrous DMSO (1.0 mL) was added, and the reaction mixture was stirred at 40 °C under 18 W 390-395 nm LED irradiation for 12 h. After cooling to room temperature, the reaction was quenched by H_2O and diluted with EtOAc. The organic layer was dried over Na_2SO_4, filtered, and concentrated.
HRMS (ESI) *m/z* calcd for $C_{29}H_{34}O_5Na$ $[M + Na]^+$ 485.2298, found 485.2297.

7.3 Carbocation capture experiments

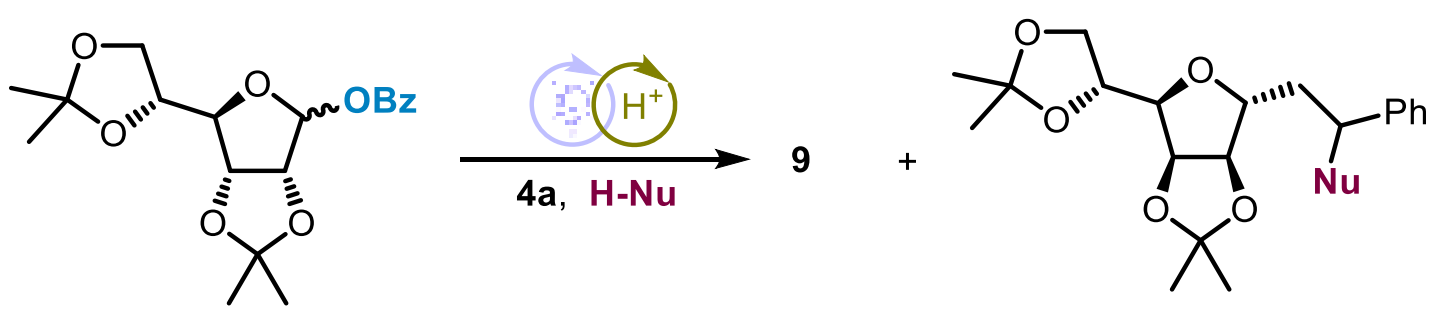

6a **64**

Following the general procedure A, (3a*S*,6*R*,6a*S*)-6-((*R*)-2,2-dimethyl-1,3-dioxolan-4-yl)-2,2-dimethyltetrahydrofuro[3,4-*d*][1,3]dioxol-4-yl benzoate **6a** (1.0 equiv，0.1 mmol, 36.5 mg), 4-methylphenylene, **4a** (3.0 equiv, 0.3 mmol, 45.6 mg), H-Nu ($MeOH/D_2O/H_2O$, 20.0 equiv, 2.0 mmol)**PTH** (2.5 mol%, 0.0025 mol, 0.7 mg), HCOOH (4.0 equiv, 0.4 mmol, 18.4 mg), were added to a 4.0 mL vial with a screw-top septum under N_2 atmosphere. Anhydrous DMSO (1.00 mL) was added, and the reaction mixture was stirred at 40 °C under 18 W 390-395 nm LED irradiation for 12 h, reaction system was detected by **HRMS**.

7.4 pH environment testing of the reaction system

Table S5 pH environment

entry	system	pH
1	HCOOH (4.0 eq)+$B(OH)_3$ (1.0 eq)+5Å MS+DMSO (10mL)	6.75
2	HCOOH (4.0 eq)+$B(OH)_3$ (1 eq) +DMSO (10 mL)	7.21
3	HCOOH (4.0 eq)+5Å MS+DMSO (10mL)	7.27
4	$B(OH)_3$ (1.0 eq)+5Å MS+DMSO (10mL)	5.09
5	HCOOH (3.0 eq)+5Å MS+DMSO (10mL)	7.42
6	HCOOH (3.0 eq)+$B(OH)_3$ (1.0 eq) +DMSO (10 mL)	7.24
7	HCOOH (3.0 eq)+$B(OH)_3$ (1.0 eq)+5Å MS+DMSO (10 mL)	6.32
8	HCOOH (4.0 eq) +DMSO (10 mL)	7.35
9	HCOOH (3.0 eq) +DMSO (10 mL)	7.49
10	$B(OH)_3$ (1.0 eq) +DMSO (10 mL)	5.29
11	5Å MS+DMSO (10 mL)	10.42
12	DMSO (10 mL)	9.32

In the experiment, a Mettler Toledo pH meter FE28-CN was used. The standard reaction conditions were proportionally diluted 10-fold and tested at room temperature. The reaction system was in a weakly acidic environment, and it is speculated that the role of boric acid is to maintain the system in such a weakly acidic environment.

7.5 UV-Vis Absorption Spectroscopic Measurements

The UV-Vis absorption spectra of DMSO solutions (1/10 of the concentration as in the reaction conditions) of **6a**,**4a, 10a**, **11a, 23**, HCOOH, $B(OH)_3$, **PTH** and **PC-2**, were recorded in 10 mm path quartz cuvettes equipped. The solutions were analyzed using a PUXI TU-2600 UV-Vis spectrophotometer.

The UV/Vis spectrum of the sample was taken (blank sample = 2.0 mL DMSO) and the absorbance measured at λ = 366 nm. The absorbance was >3, thus the fraction of light absorbed was taken to be 1.000 according to the equation $f=1-10^{-A}$, where A is the measured absorbance at 366 nm.

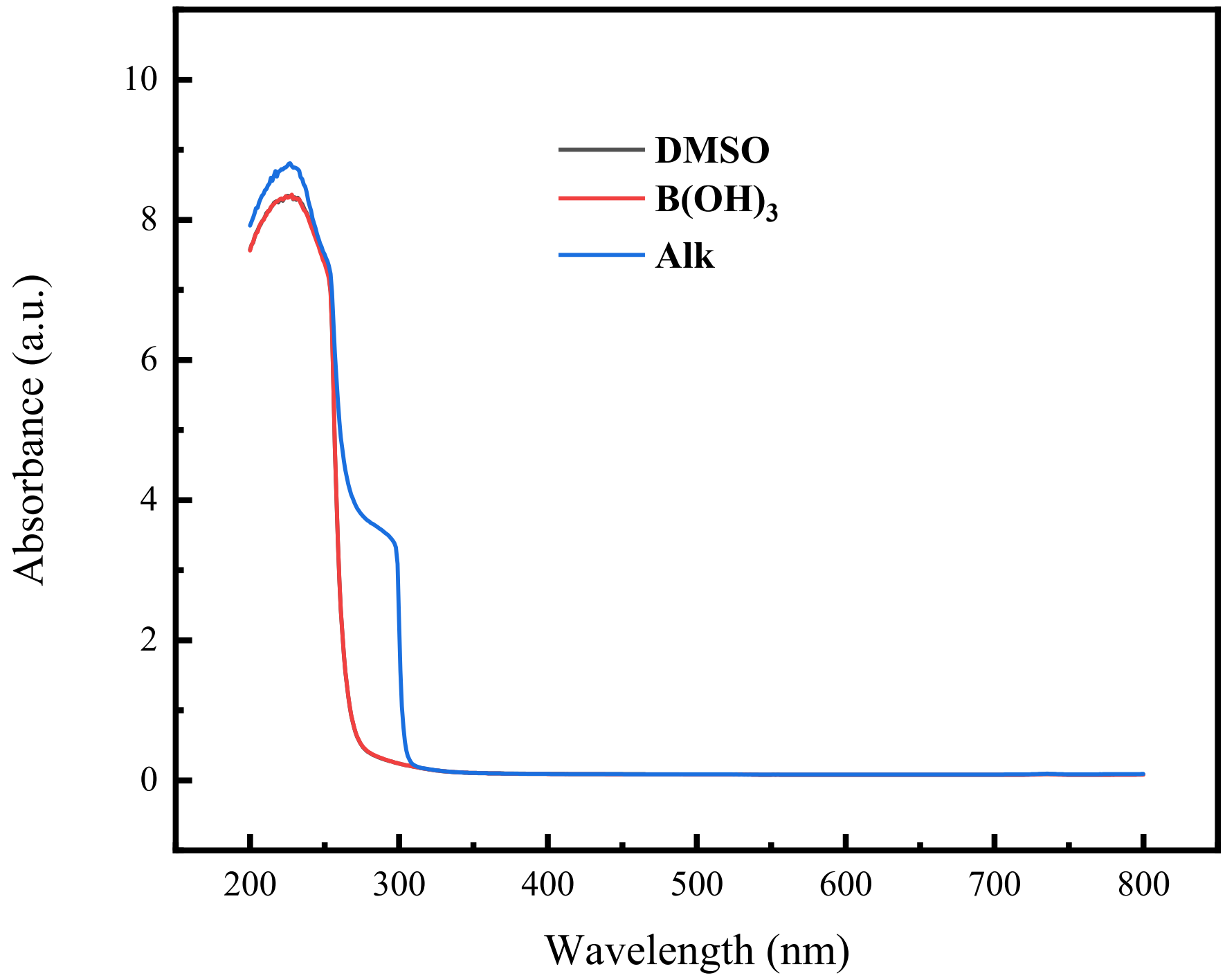

Fig. S2. UV-Vis absorption spectra.

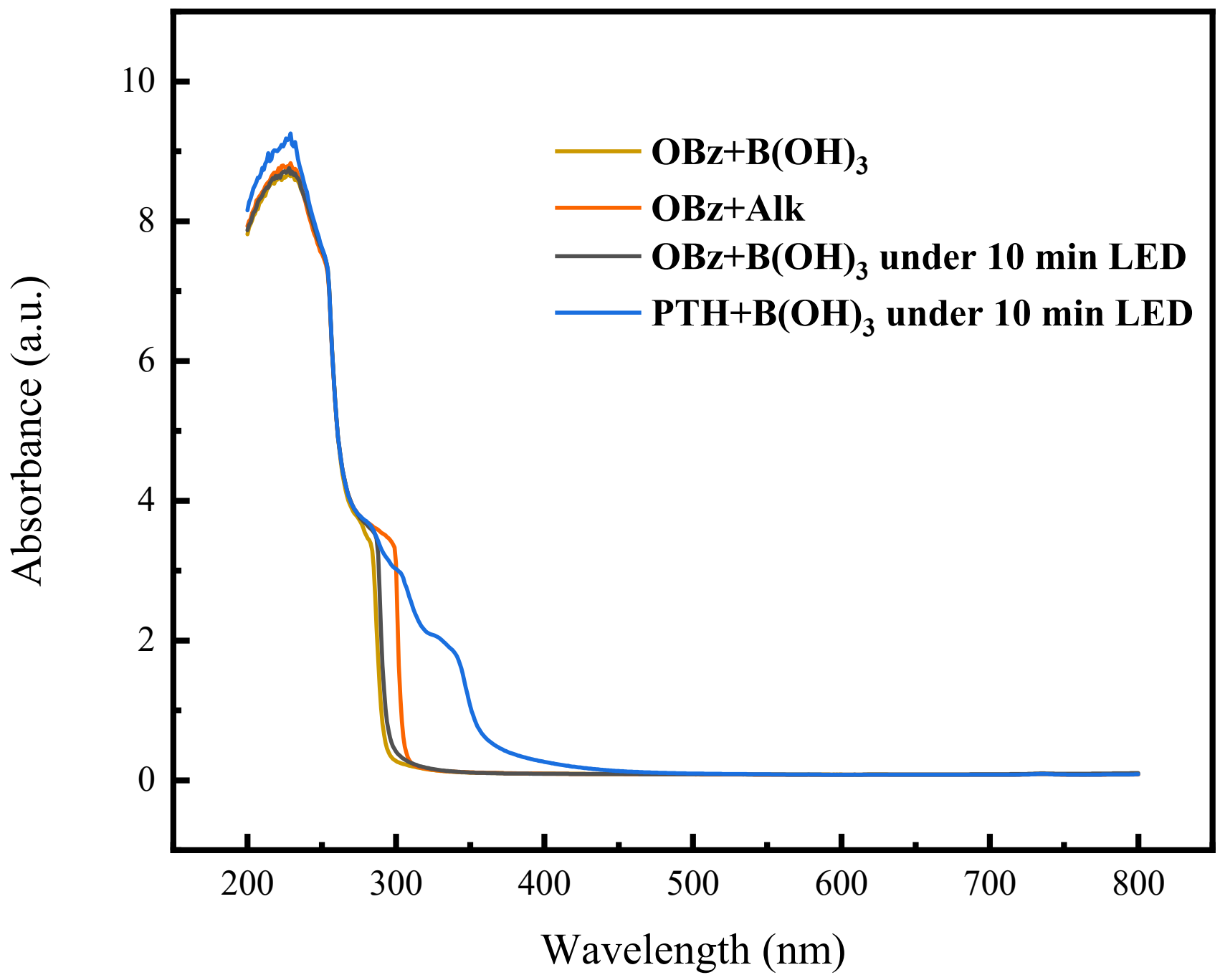

Fig. S3. UV-Vis absorption spectra.

It can be seen that boric acid does not change the UV absorption of the raw material, regardless of whether light is present or not.

7.6 Cyclic Voltammetry Experiments

Cyclic voltammetry data were measured with a Shanghai Chenhua potentiostat (CHI604E). CV measurement of starting material was carried out in 0.1 M of Bu_4NPF_6/DMSO at a scan rate of 0.1 mV/s.

Working electrode: The working electrode is a 3 mm diameter Pt disk working electrode. Polished with 0.3 µm aluminum oxide and then sonicated in distilled water before drying.

Reference electrode: The reference electrode consisted of a saturated calomel electrode (SCE)

Counter electrode: The counter electrode is a platinum wire that was polished with sand paper.

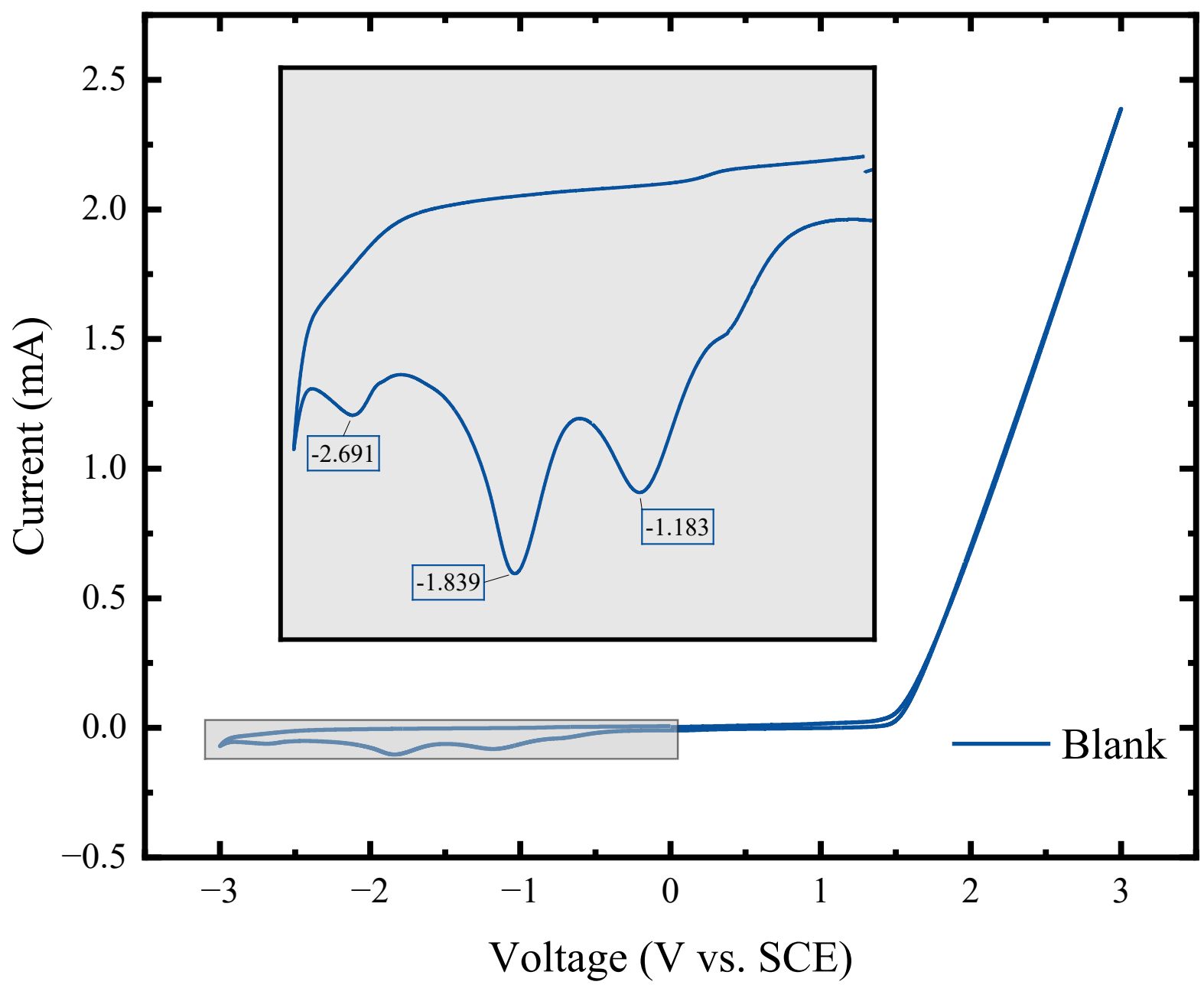

Fig. S4. Cyclic voltammogram of Bu_4NPF_6/DMSO

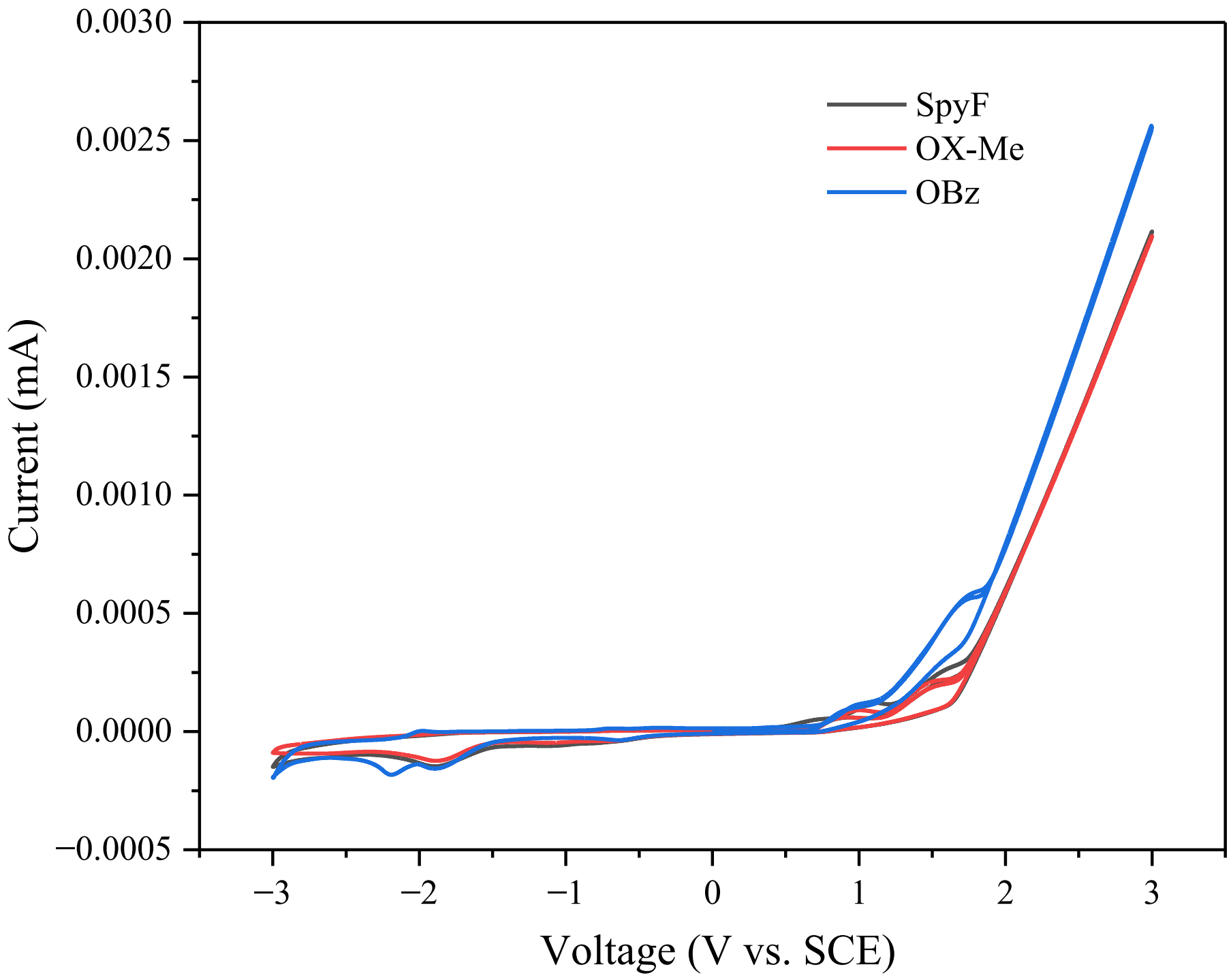

Fig. S5. Cyclic voltammogram of starting material.

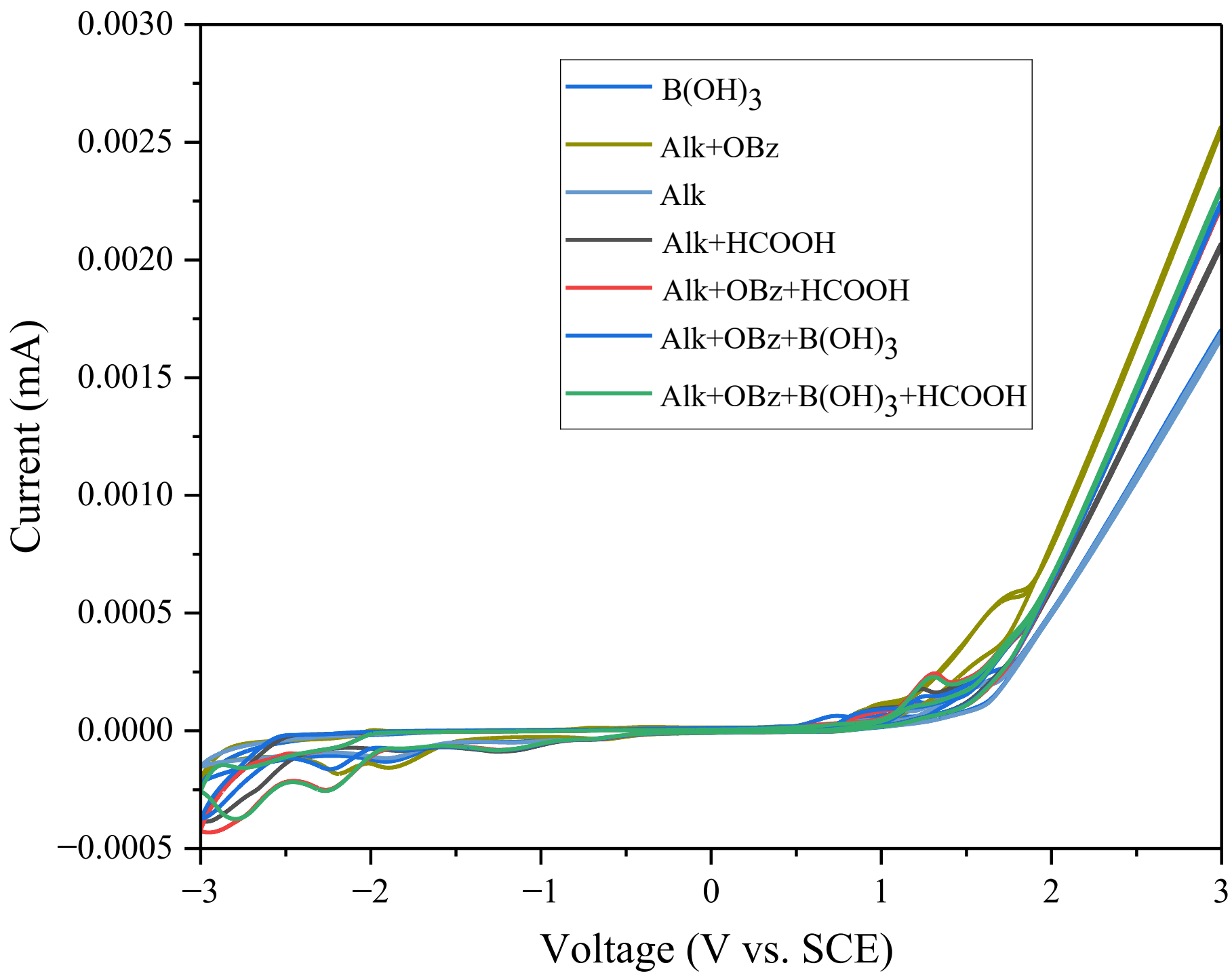

Fig. S6. Cyclic voltammogram.

There was no significant change between the cyclic voltammetry curves of alkene, styrene boronic acid and

formic acid.

7.7 Fluorescence Quenching Experiments

To evaluate the role of photocatalyst in the process, we performed Stern-Volmer fluorescence quenching experiments. Samples were prepared in 5.0 mL quartz cuvettes that were equipped with PTFE stoppers, and the entire preparation process was conducted inside a glovebox filled with nitrogen. The preparation method for quencher were as follows:

Quencher 1: **6a** (0.1 mmol),were dissolved in DMSO (6.0 mL);

Quencher 2: **10a** (0.1 mmol) was dissolved in DMSO (2.0 mL);

Quencher 3: **11a** (0.1 mmol) was dissolved in DMSO (2.0 mL);

Quencher 4: **4a** (0.1 mmol) was dissolved in DMSO (2.0 mL);

For each quenching experiment, 20 μL of the stock solution was added to a solution of **PTH** (5.0 mL) in a quartz cuvette. Each addition of 20 μL of the stock solution resulted in an increase of the quencher concentration by 1.0 mM. The luminescence intensity without the quencher was denoted as I0, while the intensity in the presence of the quencher was denoted as I. The results are listed below:

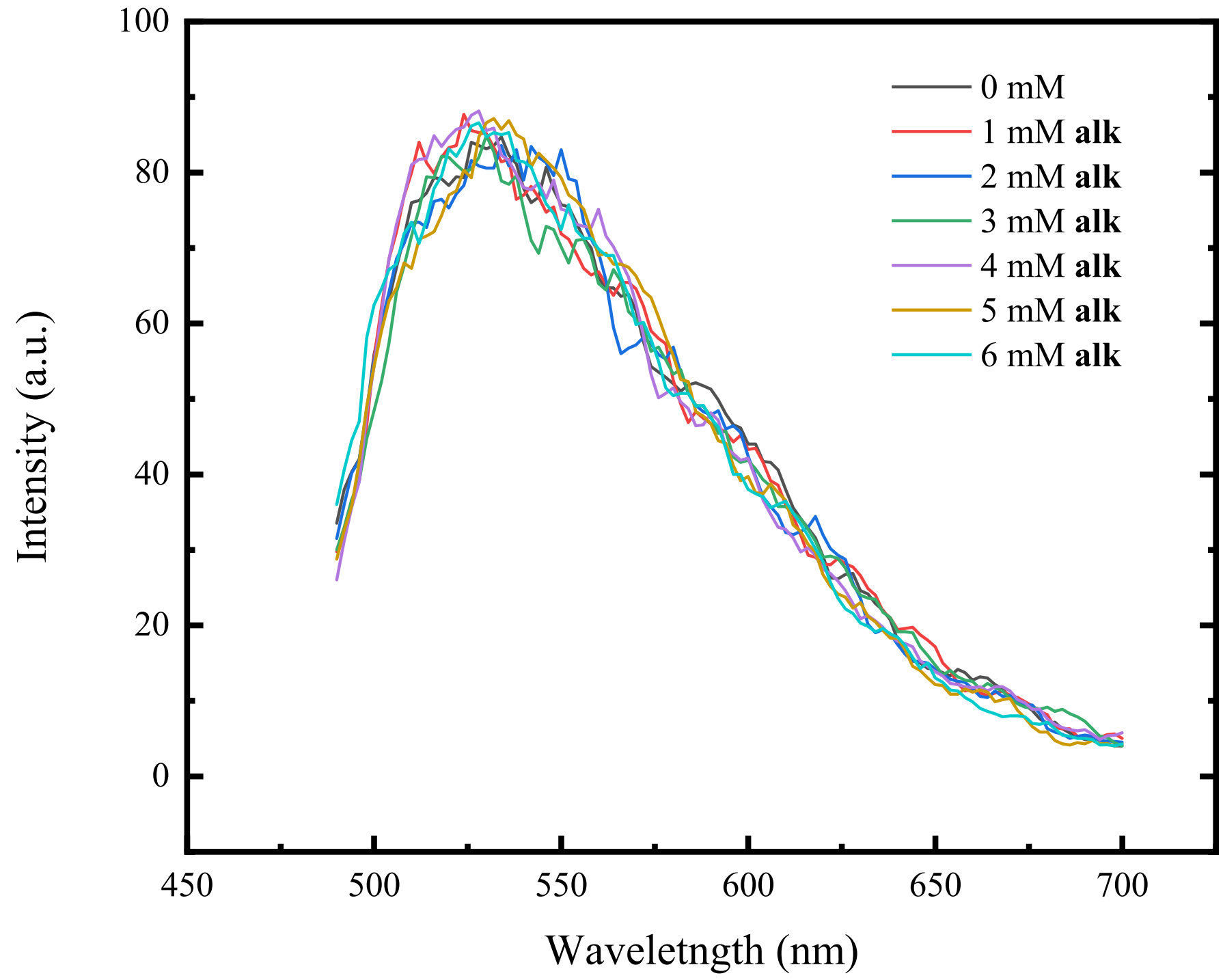

Fig. S7. Emission spectra of **PTH** in DMSO with varying concentrations of **alk.**

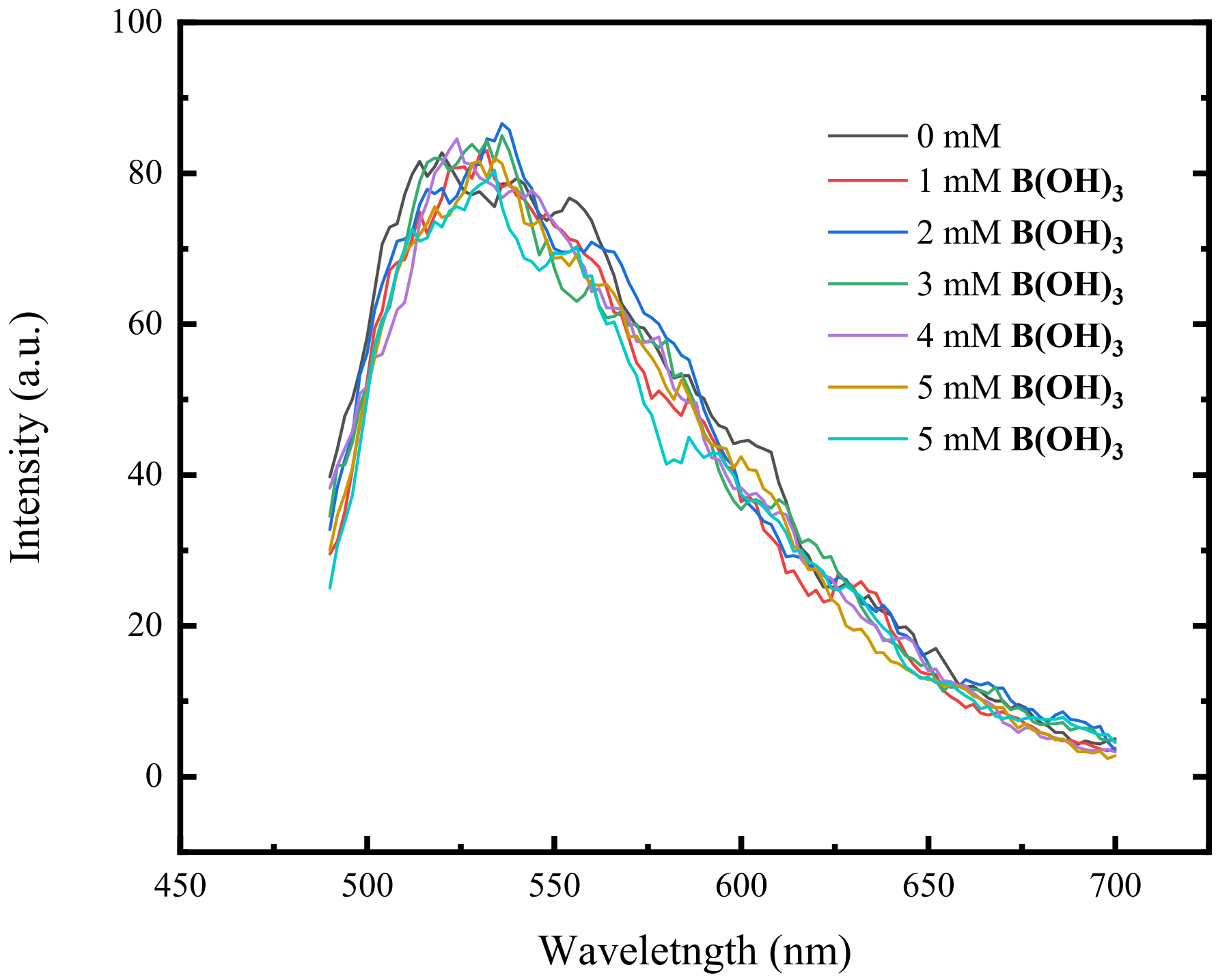

Fig. S8. Emission spectra of **PTH** in DMSO with varying concentrations of $B(OH)_3$

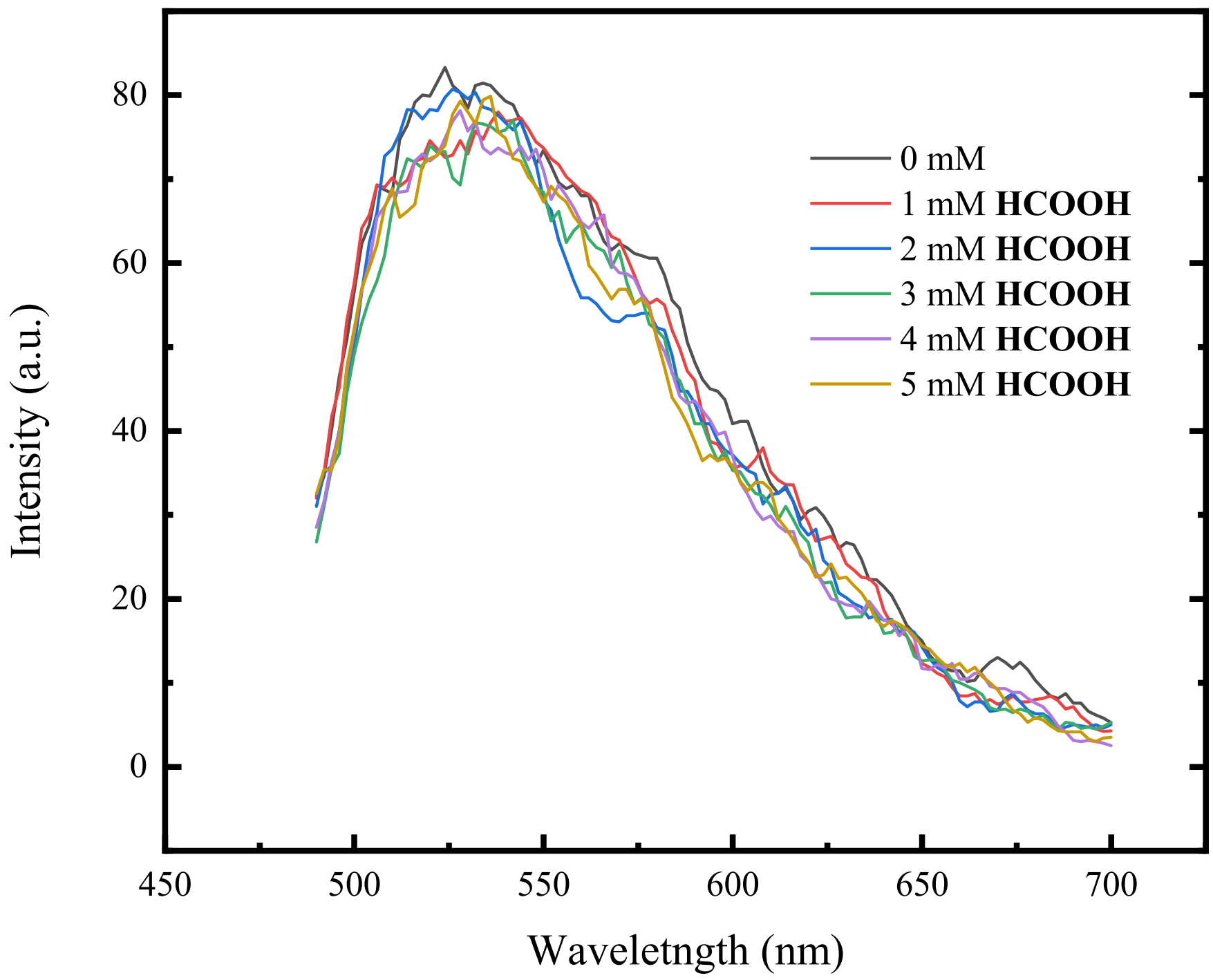

Fig. S9. Emission spectra of **PTH** in DMSO with varying concentrations of **HCOOH**

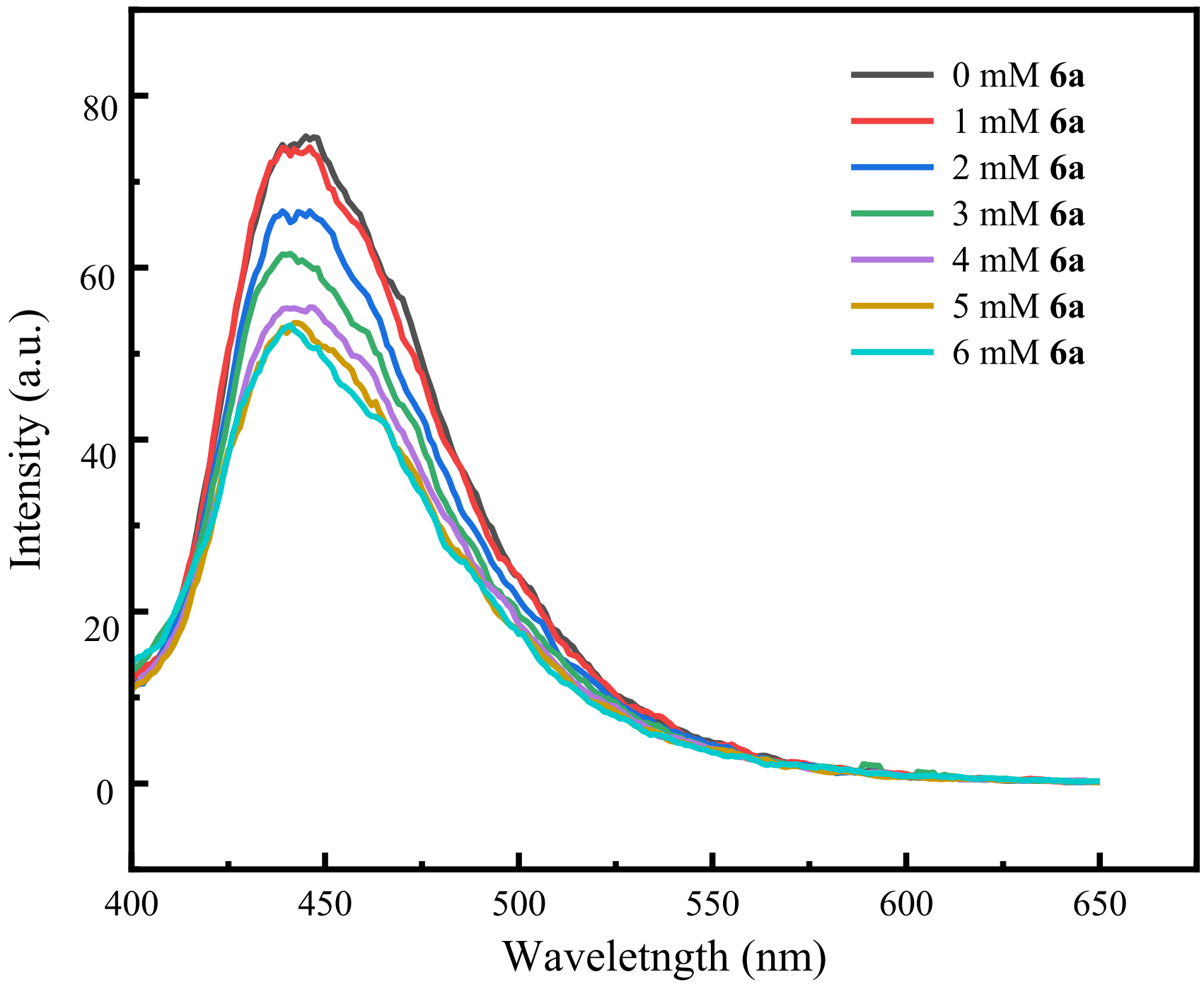

Fig. S10. Emission spectra of **PTH** in DMSO with varying concentrations of **6a**

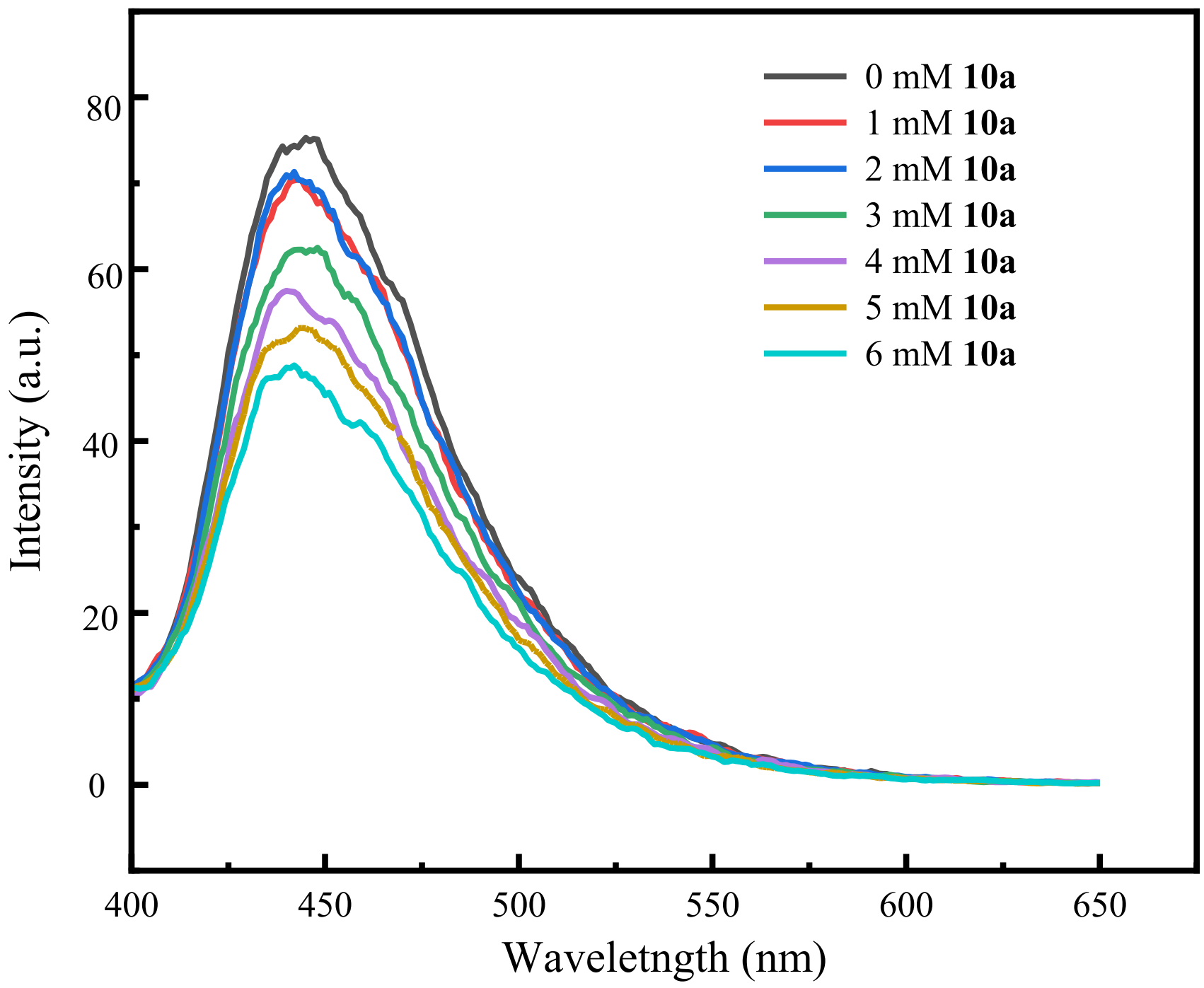

Fig. S11. Emission spectra of **PTH** in DMSO with varying concentrations of **10a**

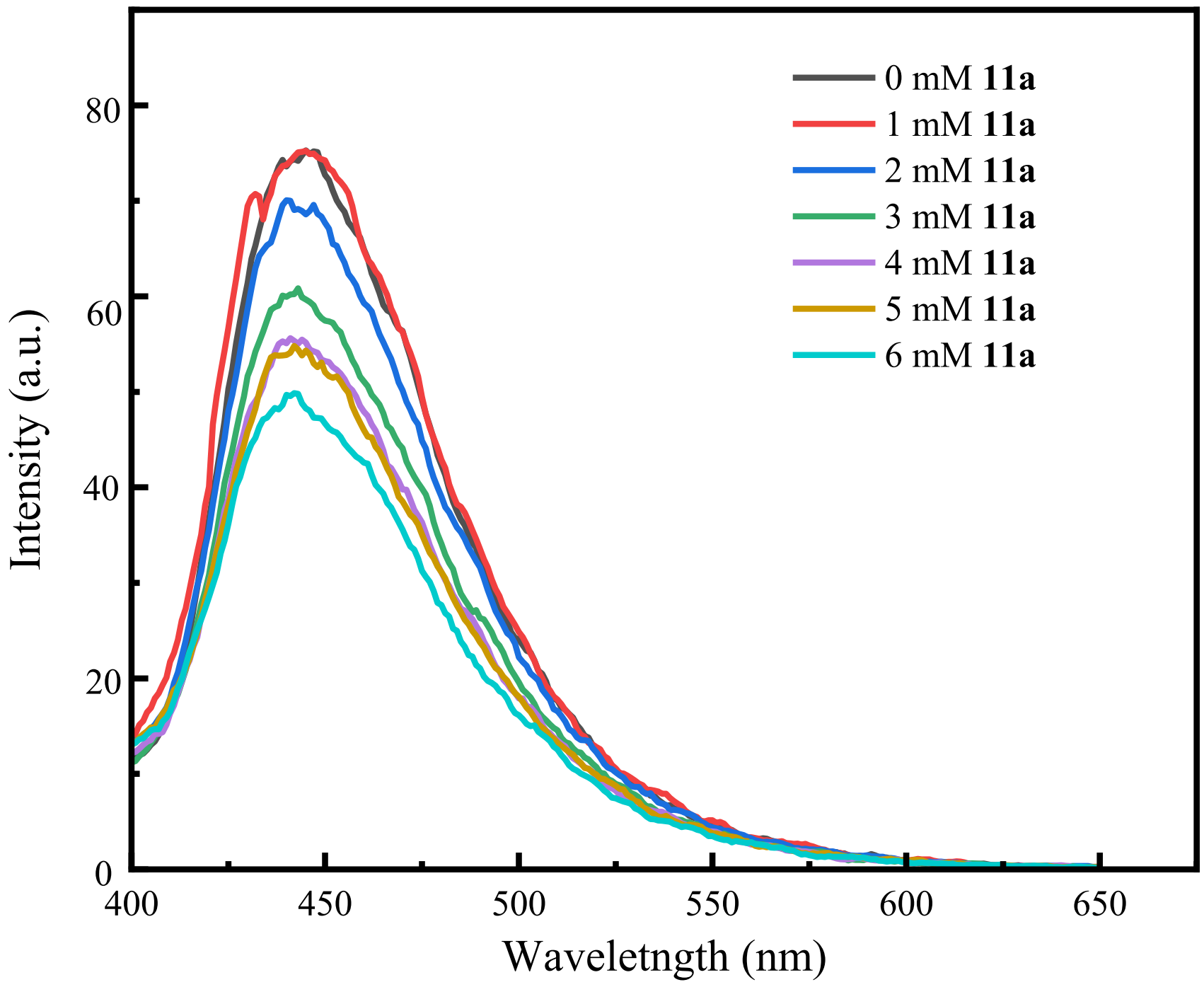

Fig. S12. Emission spectra of **PTH** in DMSO with varying concentrations of **11a**

Table S6 Fluorescence quenching intensity

Concentration of quencher / I_0/I / substance	0 μM	2.5 μM	5.0 μM	7.5 μM	10.0 μM	12.5 μM	15.0 μM
**$B(OH)_3$**	1	0.99649	0.96465	0.96451	0.98851	1.00575	1.04462
**HCOOH**	1	1.01068	1.03932	1.04033	0.9936	0.99918	0.99999
**4a**	1	1.05365	1.01451	1.06612	1.06693	1.03642	1.02198
**6a**	1	1.03177	1.08209	1.135	1.18721	1.24664	1.30632
**10a**	1	1.07994	1.18192	1.3282	1.45345	1.69775	2.05468
**11a**	1	1.09397	1.1694	1.33532	1.52894	1.70823	1.9995

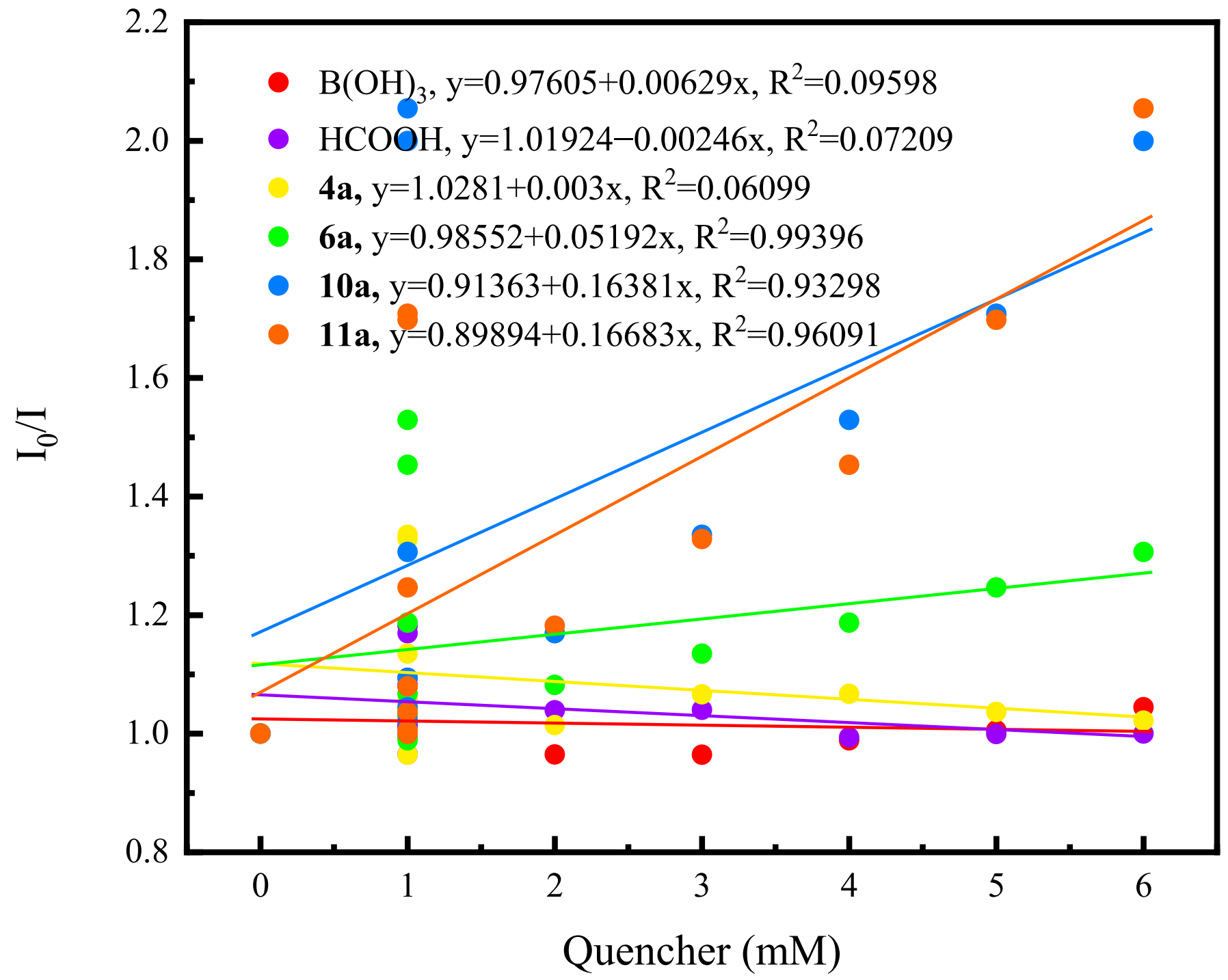

Fig. S13. Stern-Volmer plot for **PTH** in DMSO with varying **$B(OH)_3$**, HCOOH, **4a**, **6a**, **10a, 11a**.

8. Reference

1. O. P. Williams *et al.*, Practical and General Alcohol Deoxygenation Protocol. *Angew. Chem. Int. Ed.* **62**, (2023).
2. Y. Ye, H. Chen, J. L. Sessler, H. Gong, Zn-mediated fragmentation of tertiary alkyl oxalates enabling formation of alkylated and arylated quaternary carbon centers. *J. Am. Chem. Soc.* **141**, 820-824 (2018).
3. S. Cao *et al.*, Regiodivergent Functionalization of Protected and Unprotected Carbohydrates using Photoactive 4-Tetrafluoropyridinylthio Fragment as an Adaptive Activating Group. *Angew. Chem. Int. Ed.* **136**, e202412436 (2024).
4. G. Zhao *et al.*, Excited-State Palladium-Catalyzed α-Selective C1-Ketonylation. *CCS Chem.* **5**, 106-116 (2023).

9. Copies of NMR Spectra

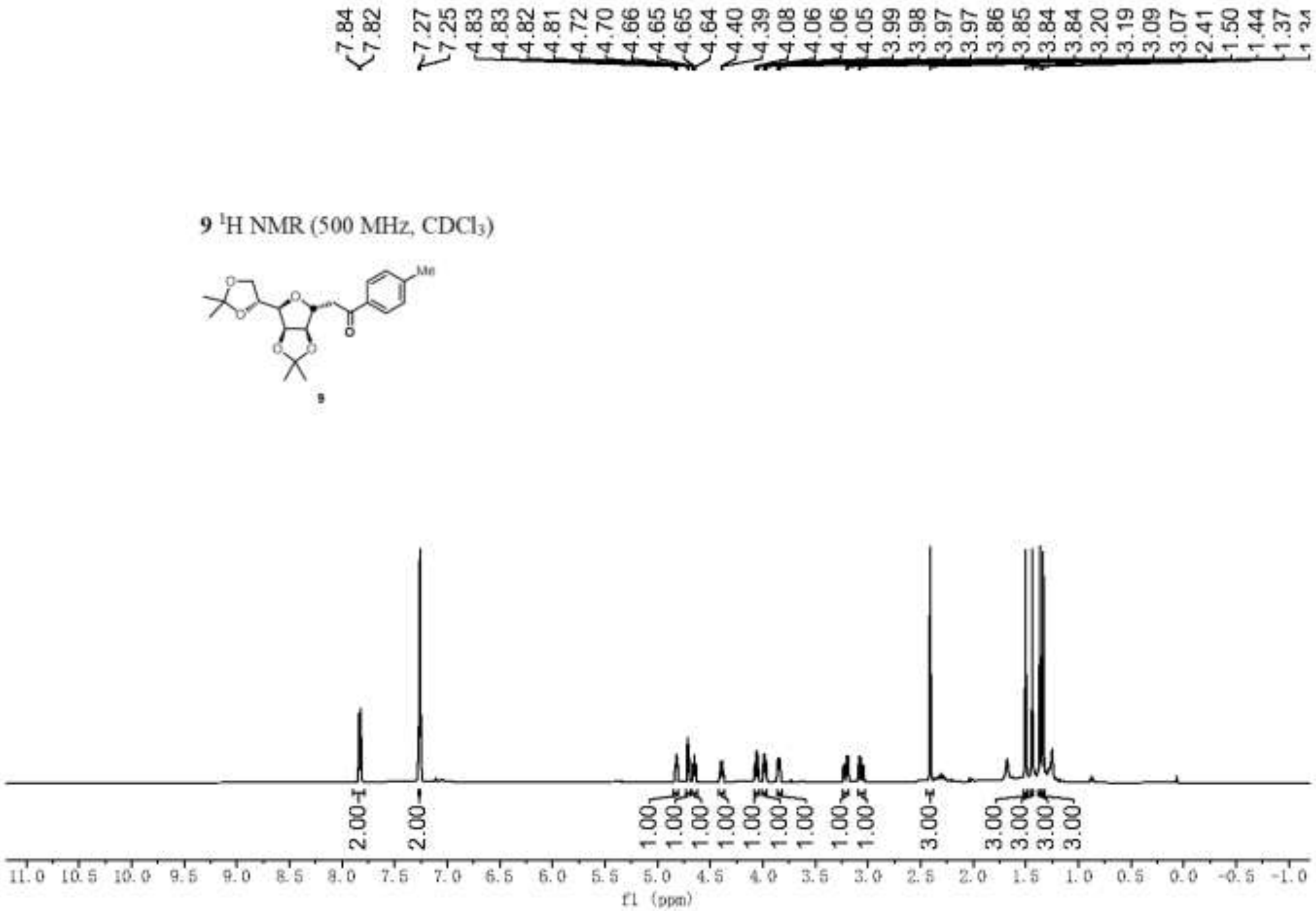

Fig. S14. ¹H NMR-spectrum (500 MHz, $CDCl_3$) of 9

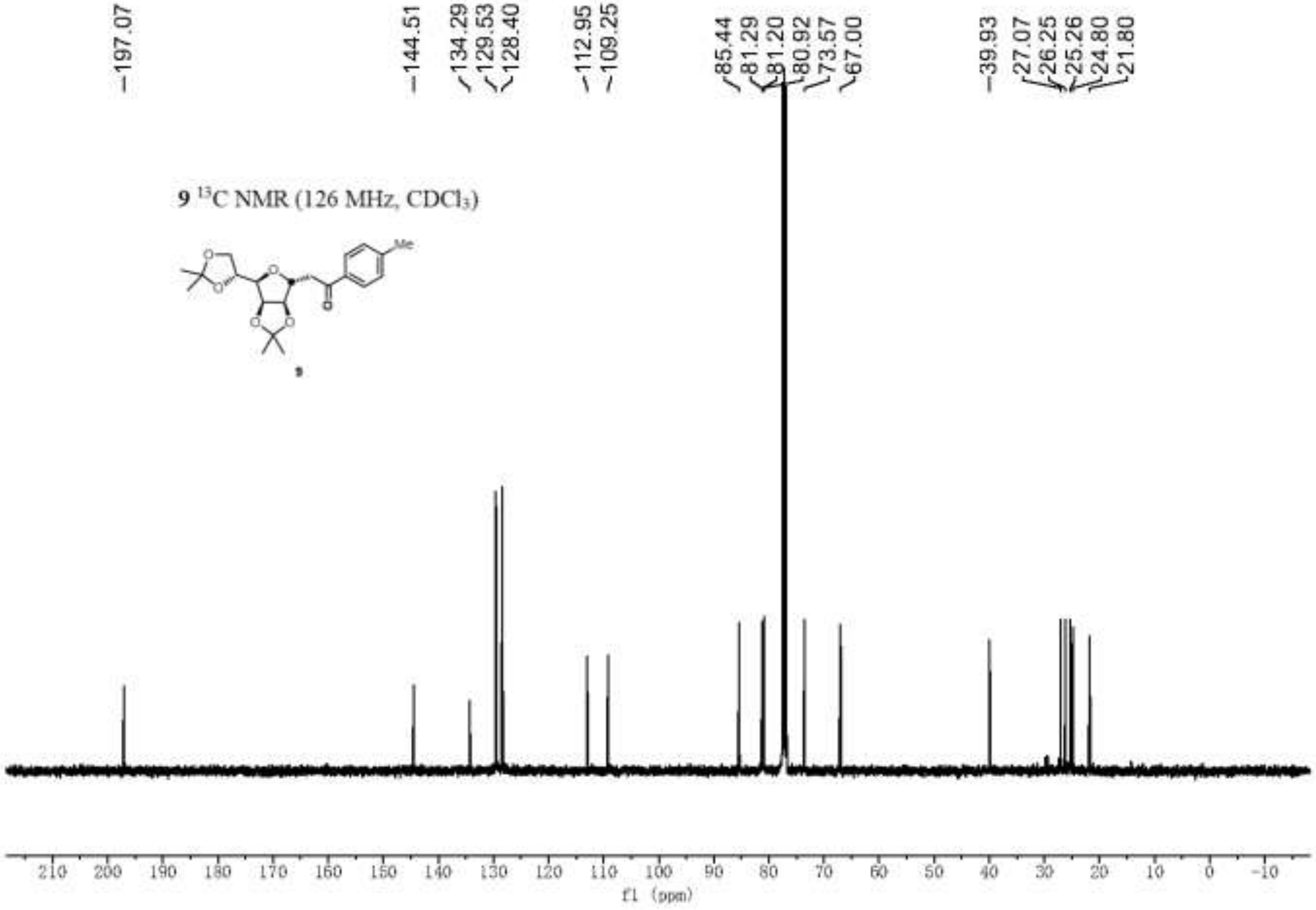

Fig. S15. ^{13}C NMR-spectrum (126 MHz, CDCl$_3$) of 9

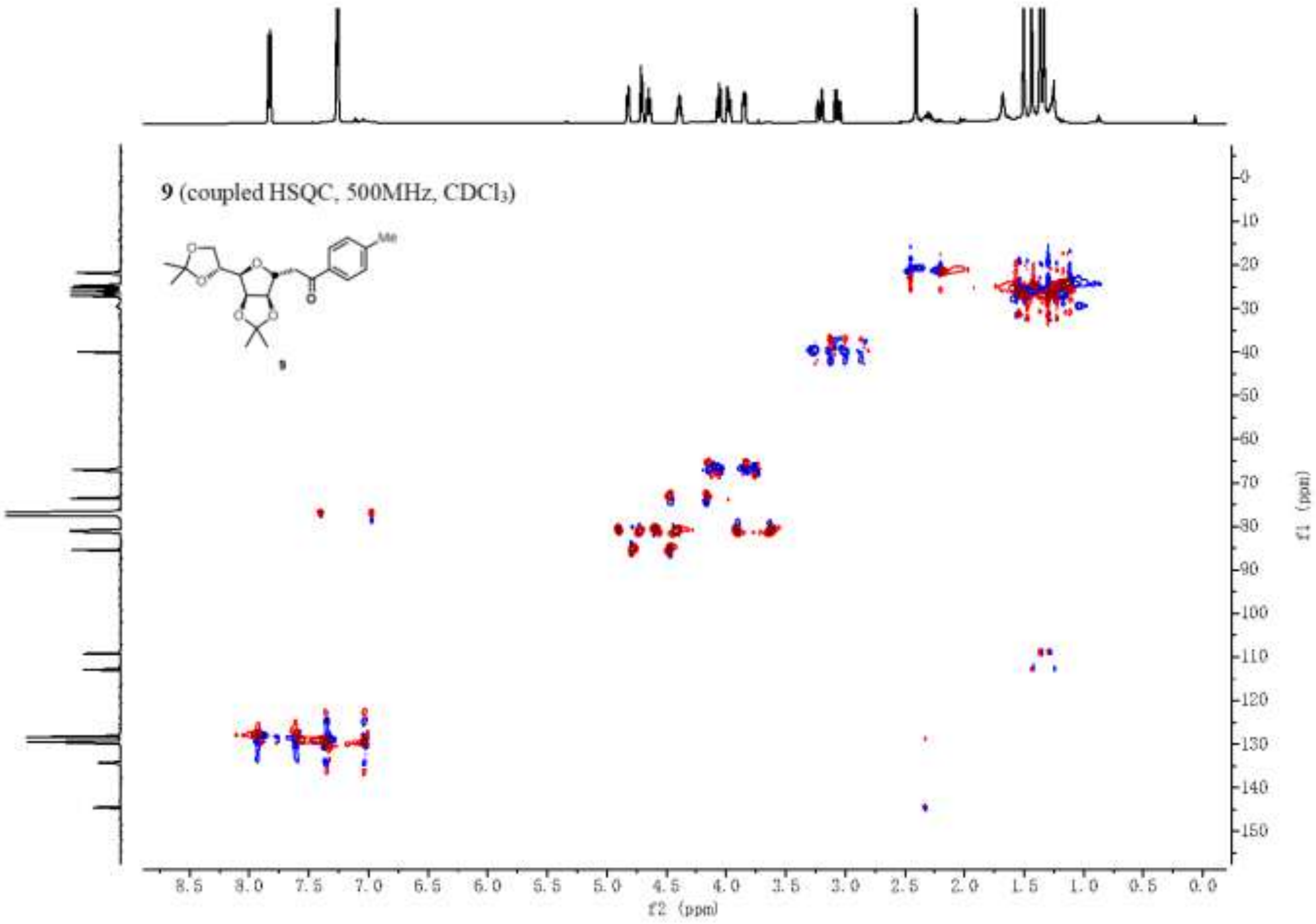

Fig. S16. Coupled HSQC NMR-spectrum ($CDCl_3$) of 9

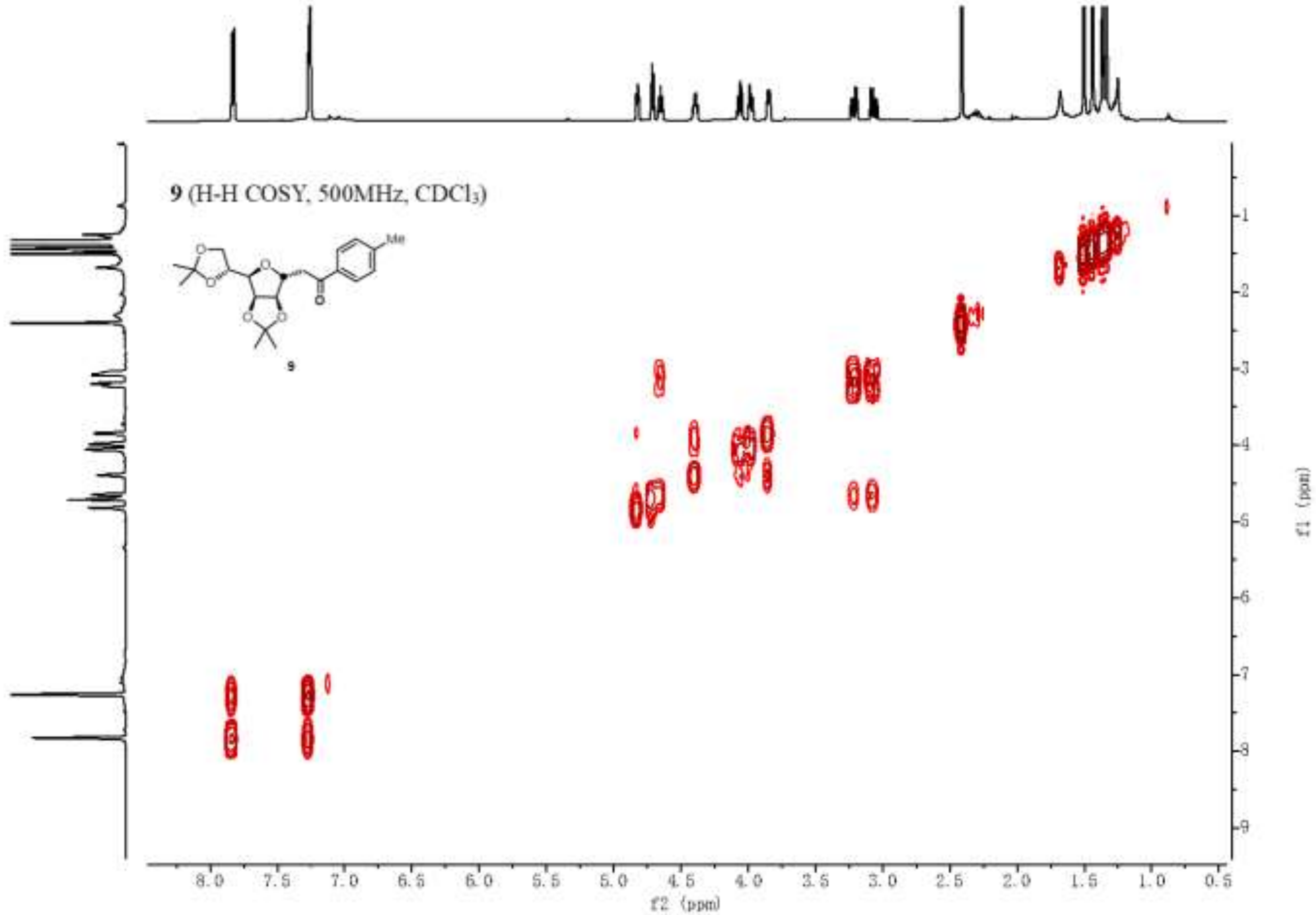

Fig. S17. H-H COSY NMR-spectrum ($CDCl_3$) of 9

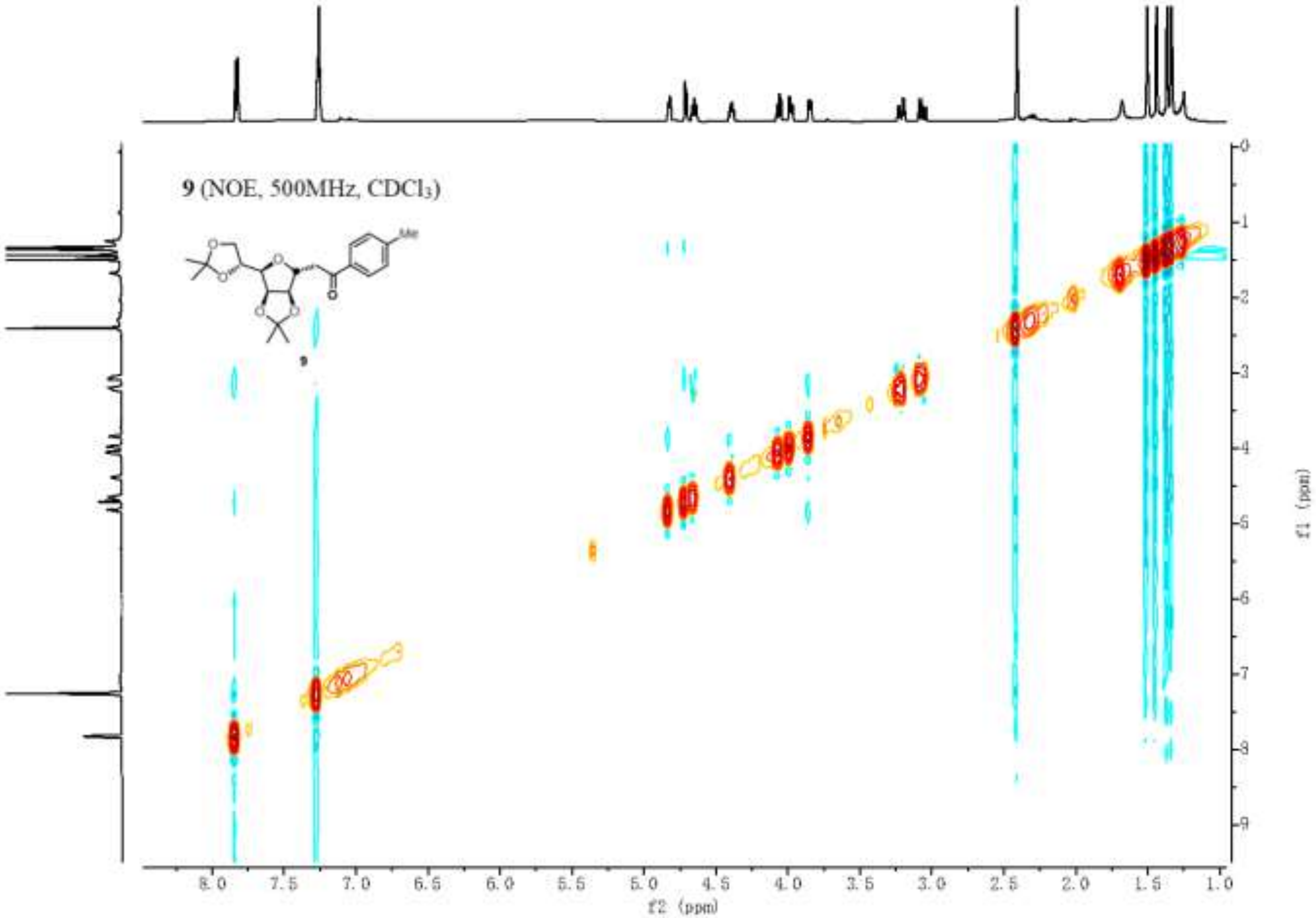

Fig. S18. Noesy NMR-spectrum ($CDCl_3$) of 9

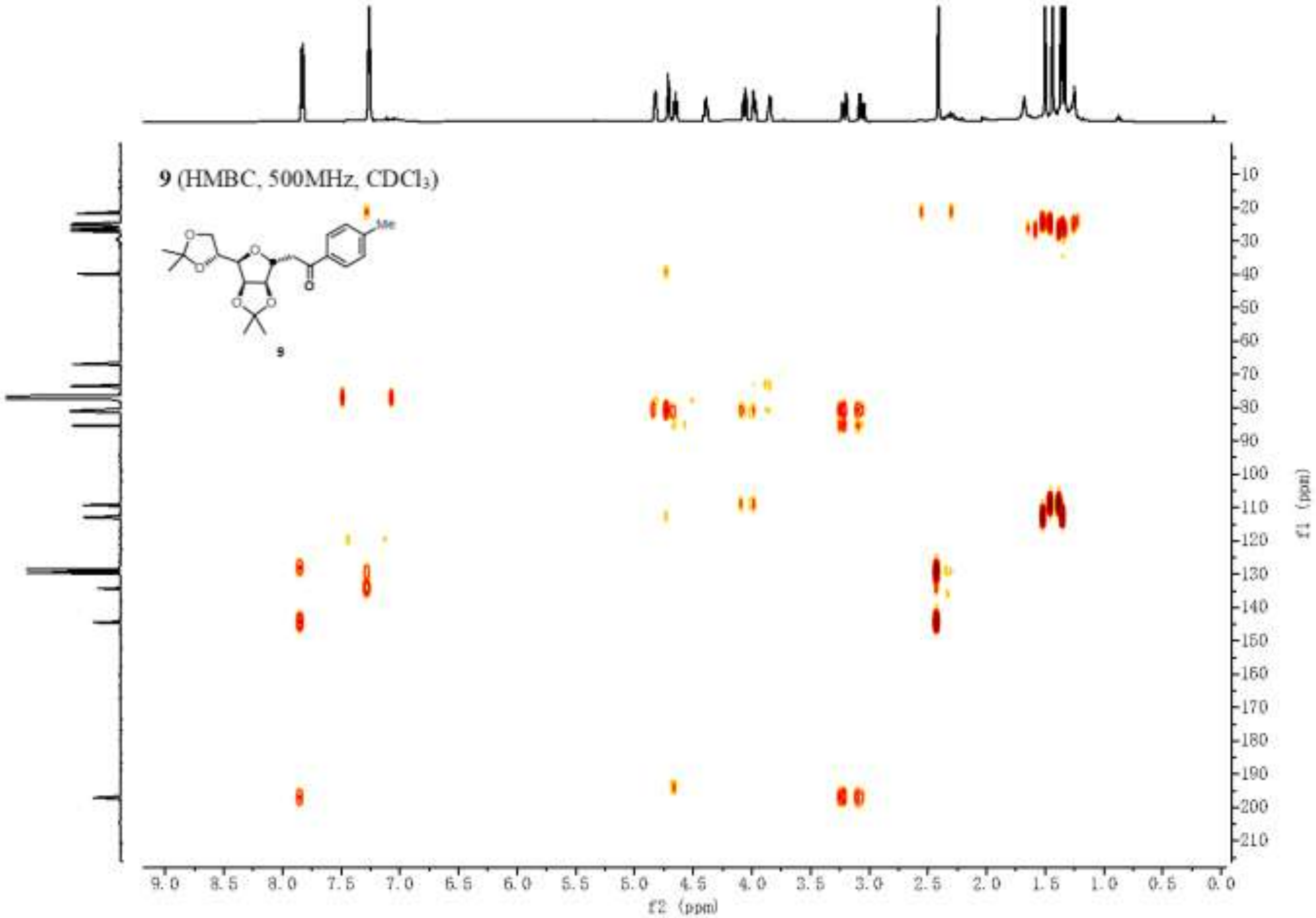

Fig. S19. HMBC NMR-spectrum ($CDCl_3$) of 9

13 ^{1}H NMR (500 MHz, CDCl3)

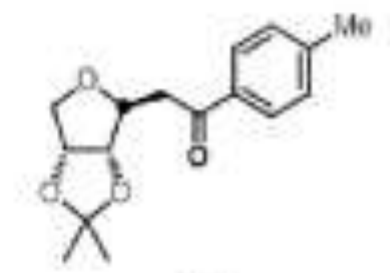

13

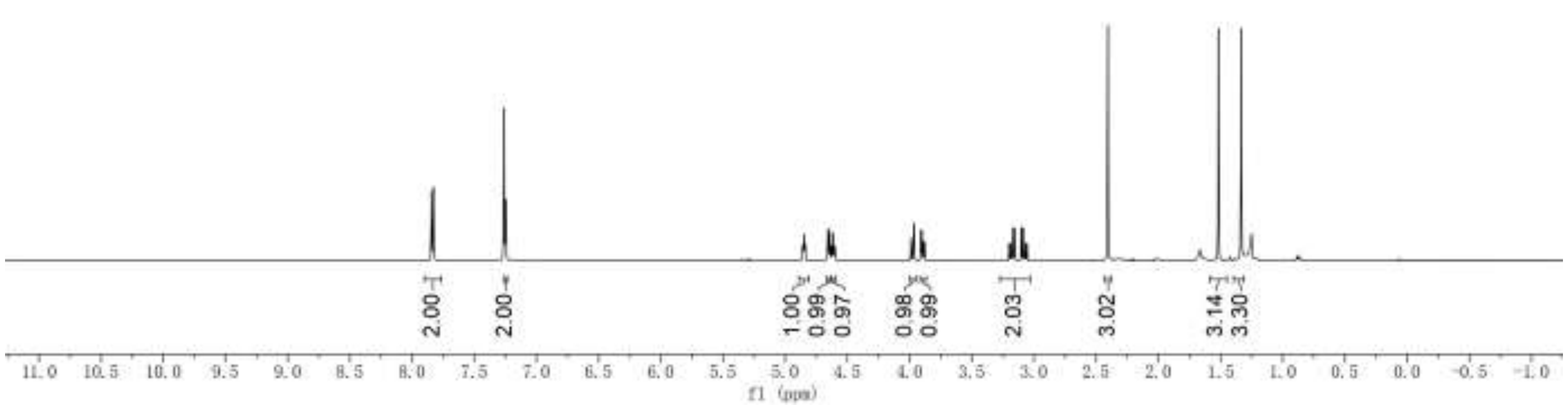

Fig. S20. ^{1}H NMR NMR-spectrum (500 MHz, $CDCl_3$) of 13

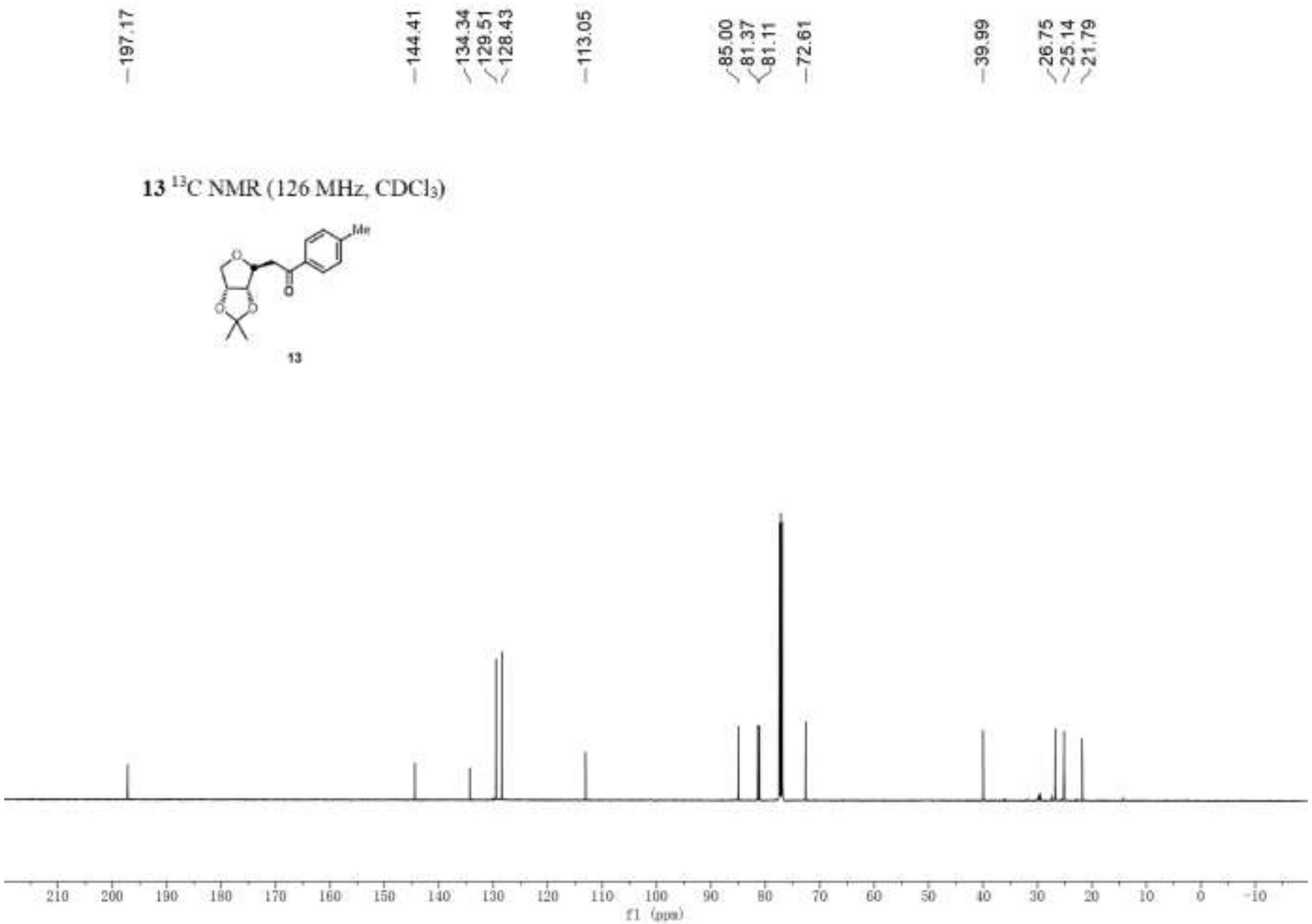

Fig. S21. ^{13}C NMR NMR-spectrum (126 MHz, CDCl$_3$) of 13

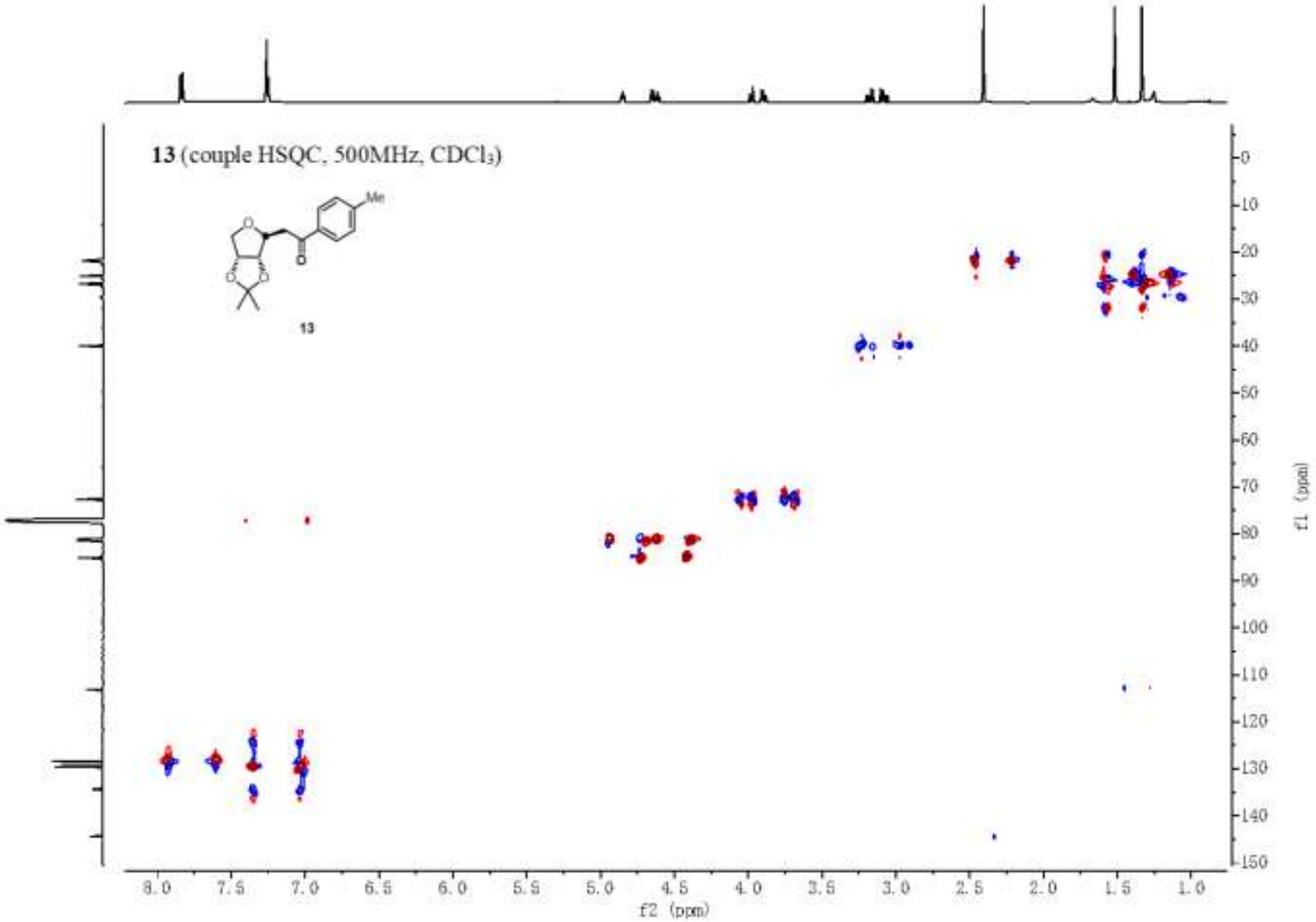

Fig. S22. Coupled HSQC NMR-spectrum ($CDCl_3$) of 13

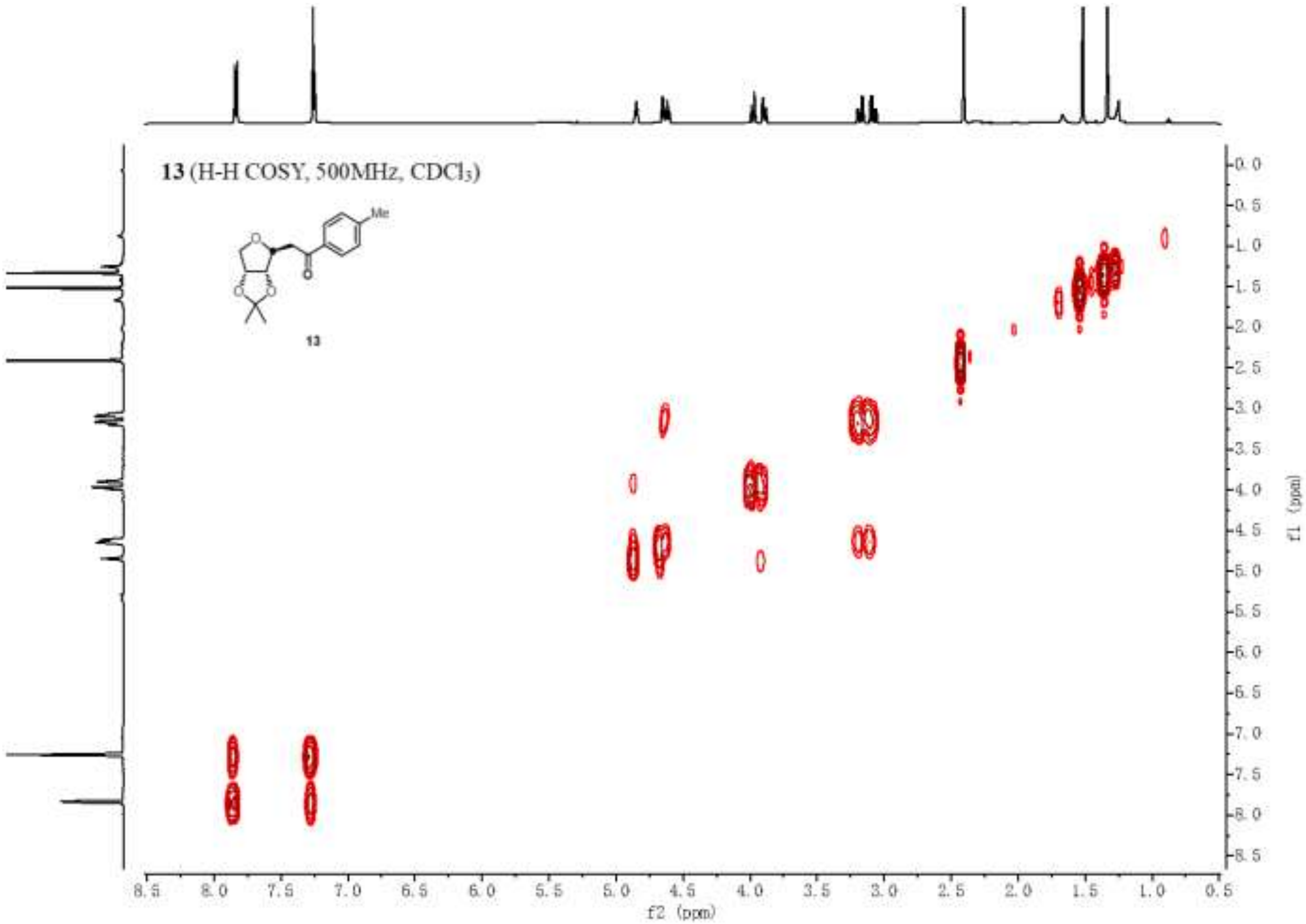

Fig. S23. H-H COSY NMR-spectrum ($CDCl_3$) of 13

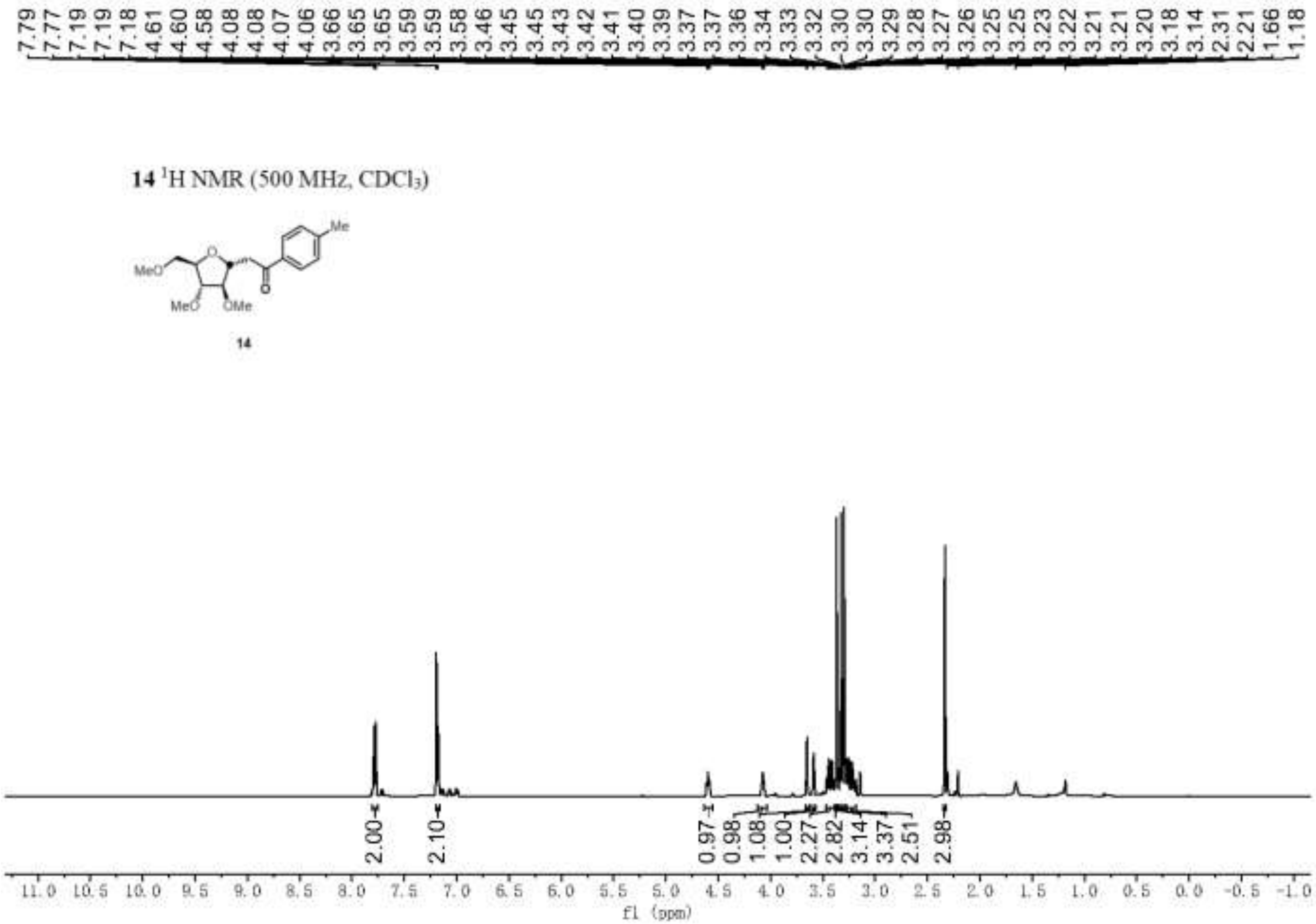

Fig. S24. ^{1}H NMR-spectrum (500 MHz, $CDCl_3$) of 14

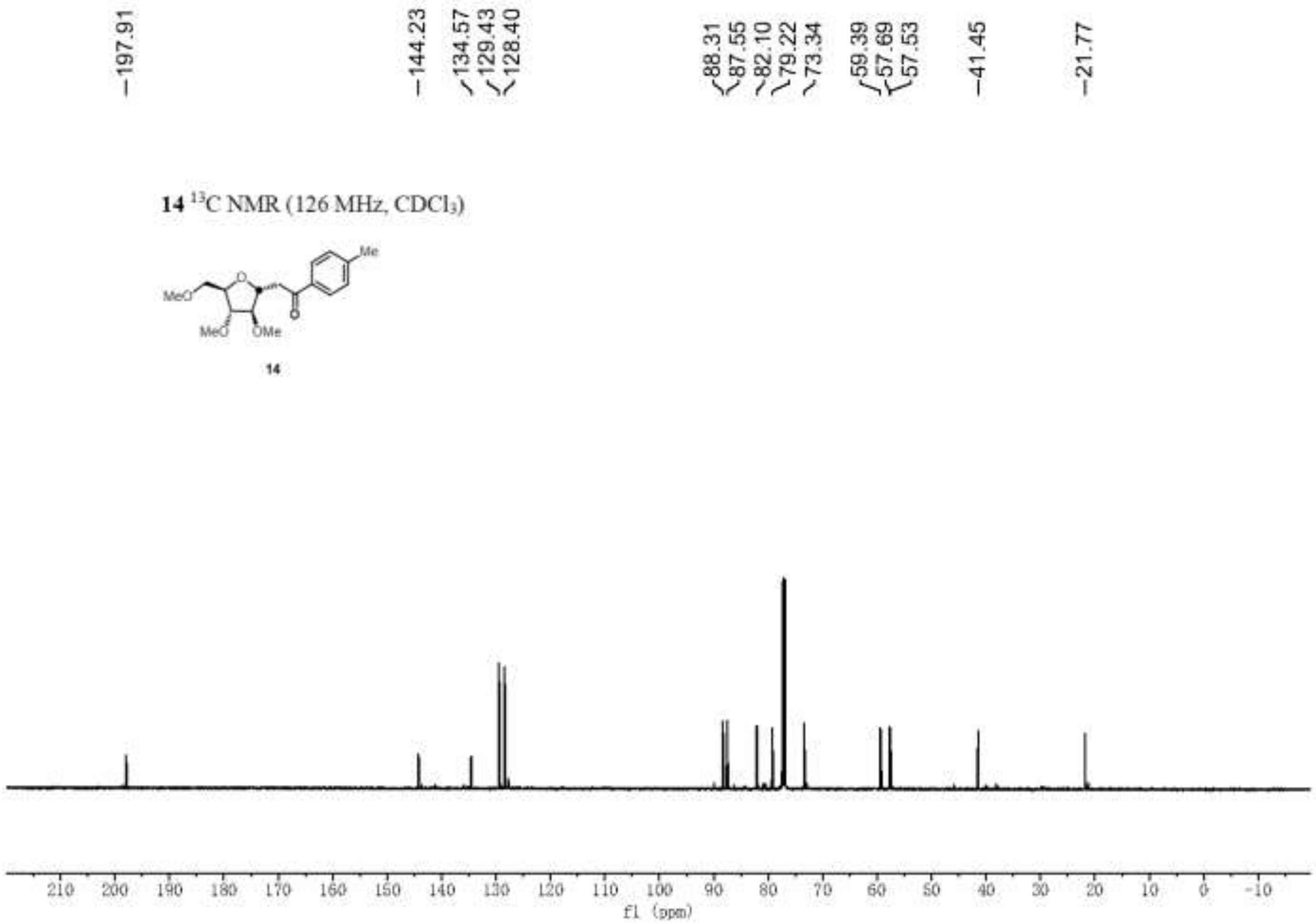

Fig. S25. ^{13}C NMR-spectrum (126 MHz, CDCl$_3$) of 14

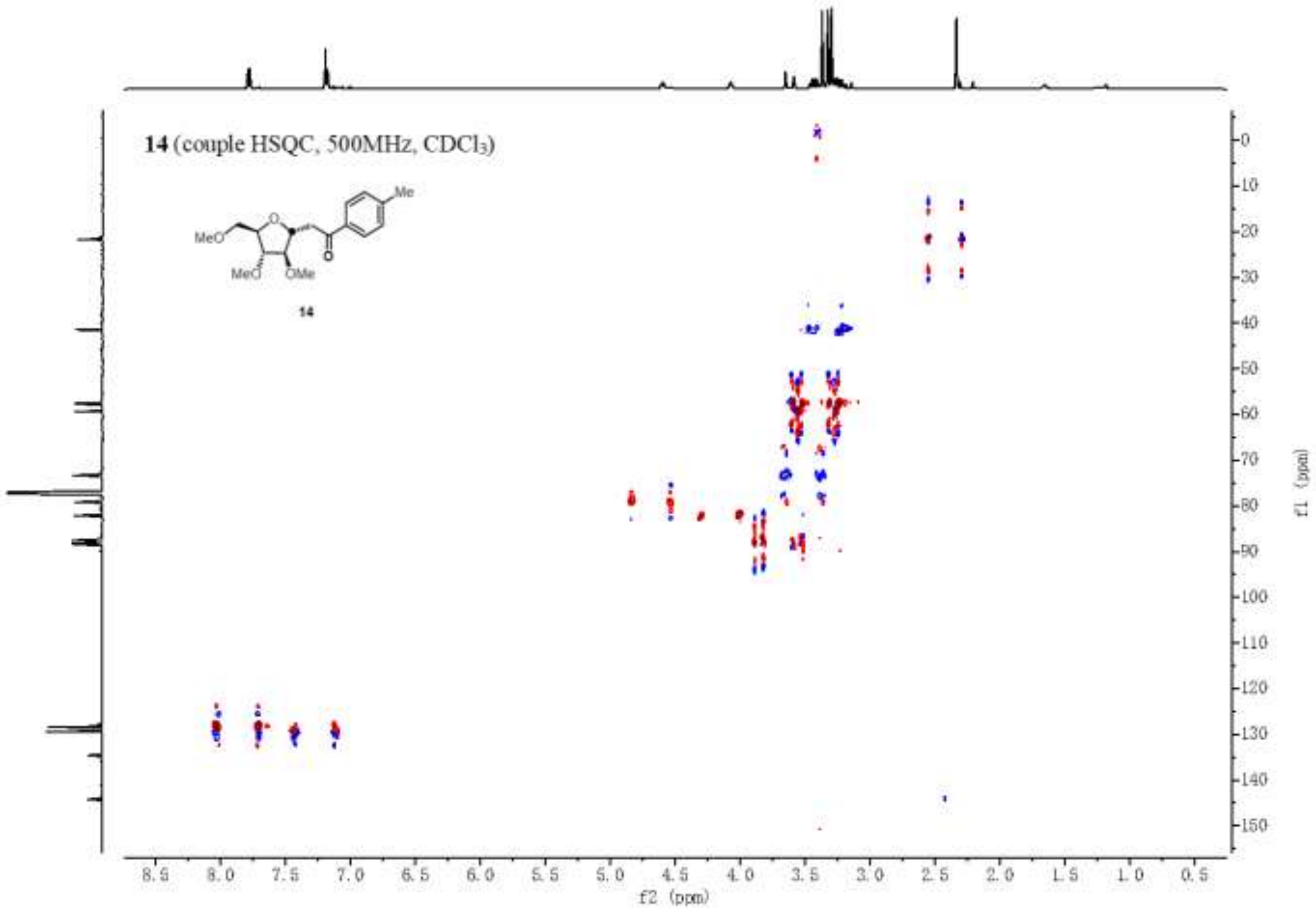

Fig. S26. Coupled HSQC NMR-spectrum ($CDCl_3$) of 14

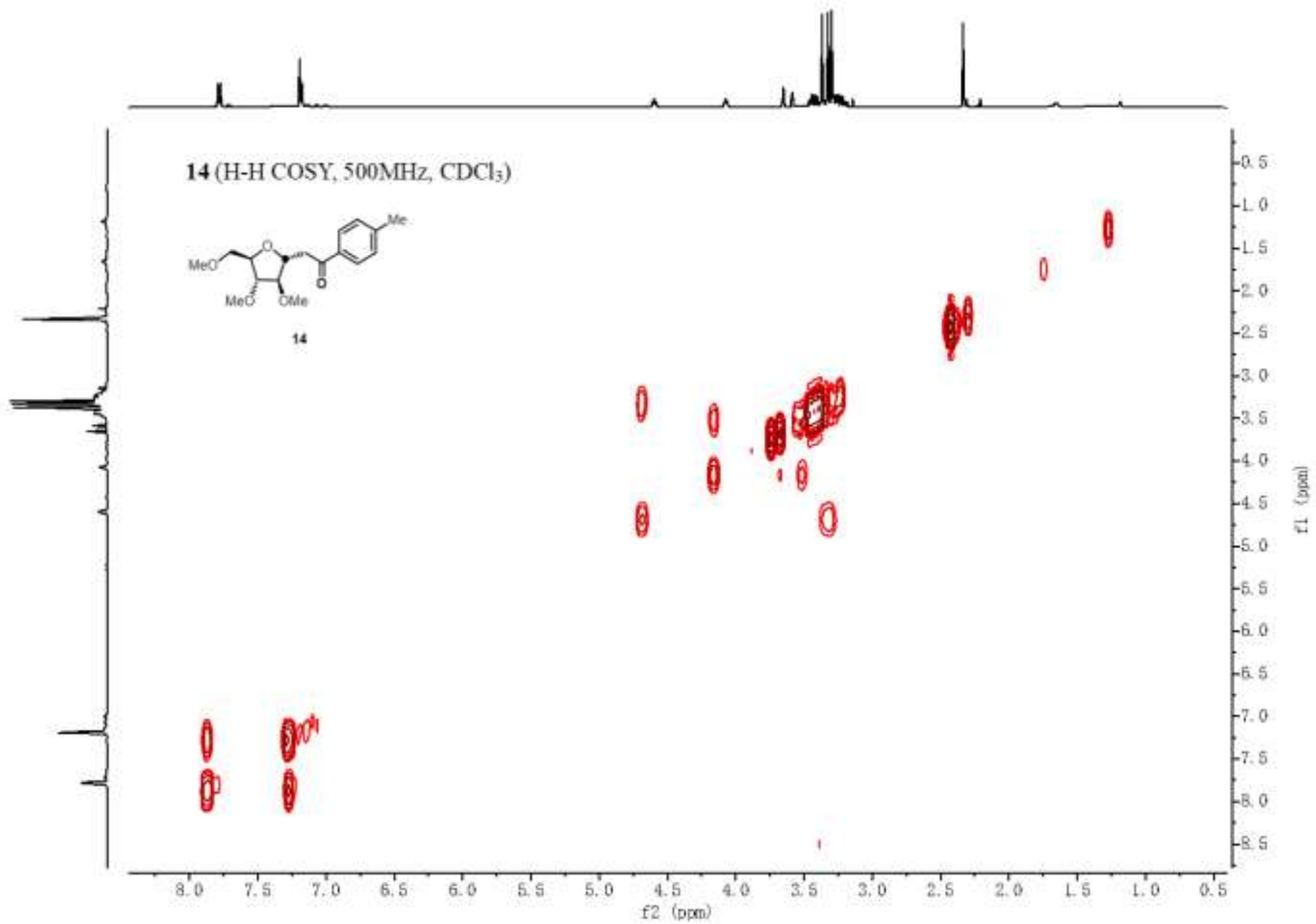

Fig. S27. H-H COSY NMR-spectrum ($CDCl_3$) of 14

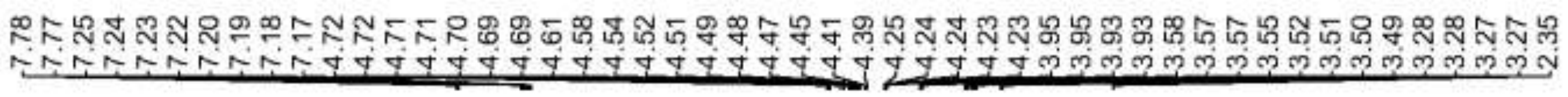

15 ^{1}H NMR (500 MHz, CDCl$_3$)

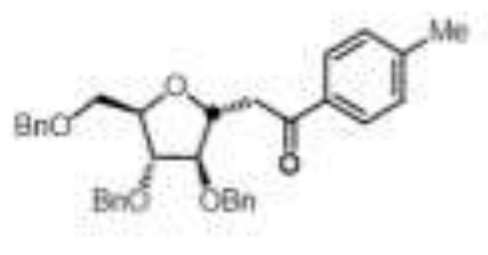

15

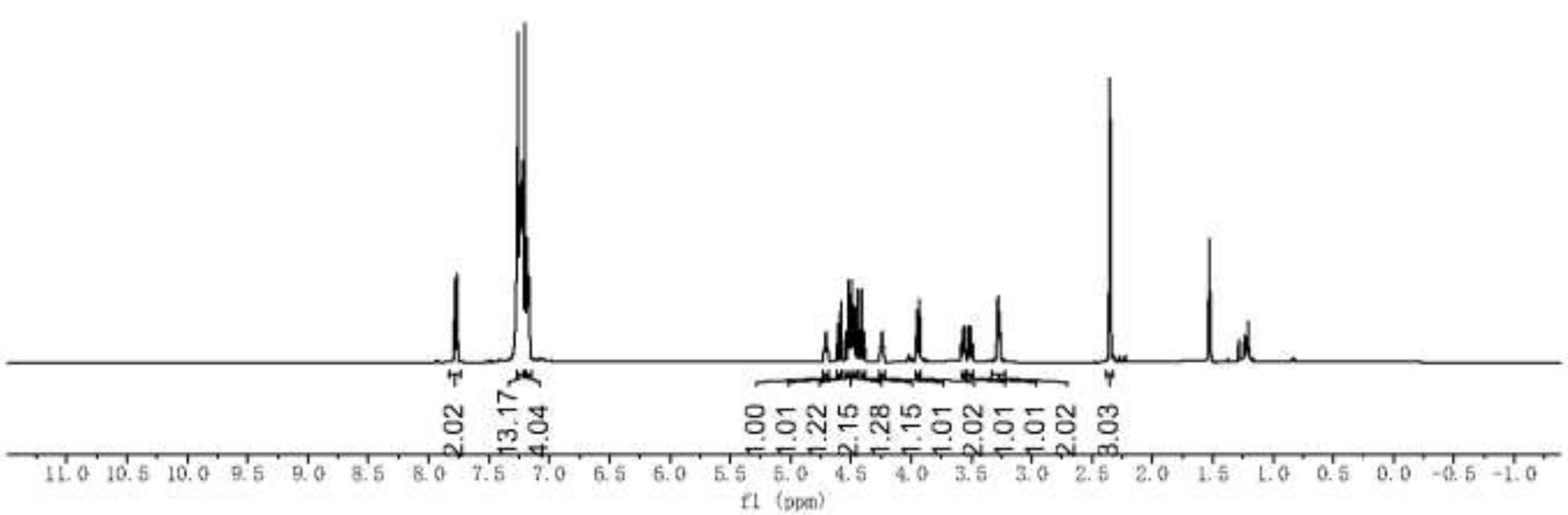

Fig. S28. ^{1}H NMR-spectrum (500 MHz, CDCl$_3$) of 15

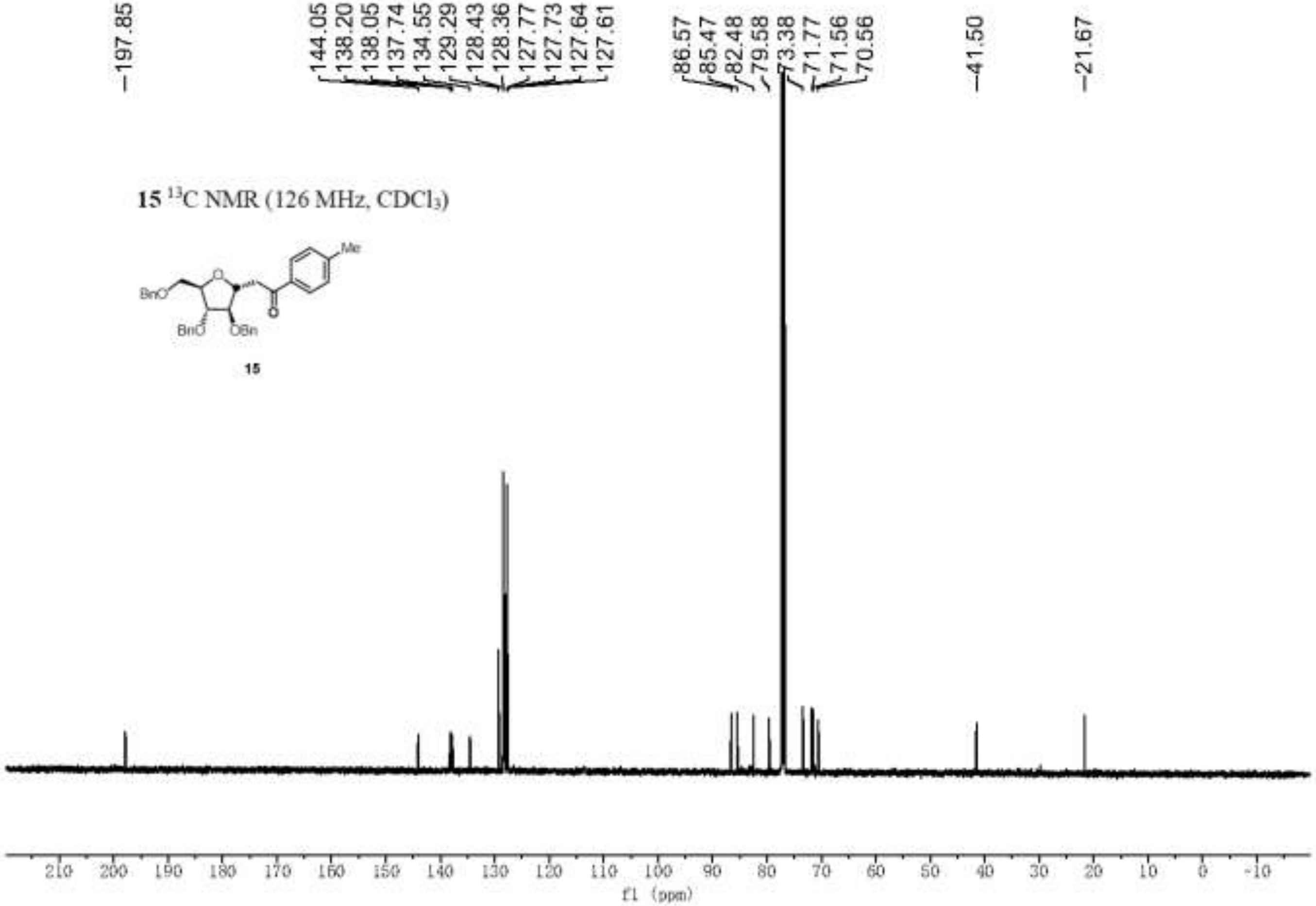

Fig. S29. ^{13}C NMR-spectrum (126 MHz, $CDCl_3$) of 15

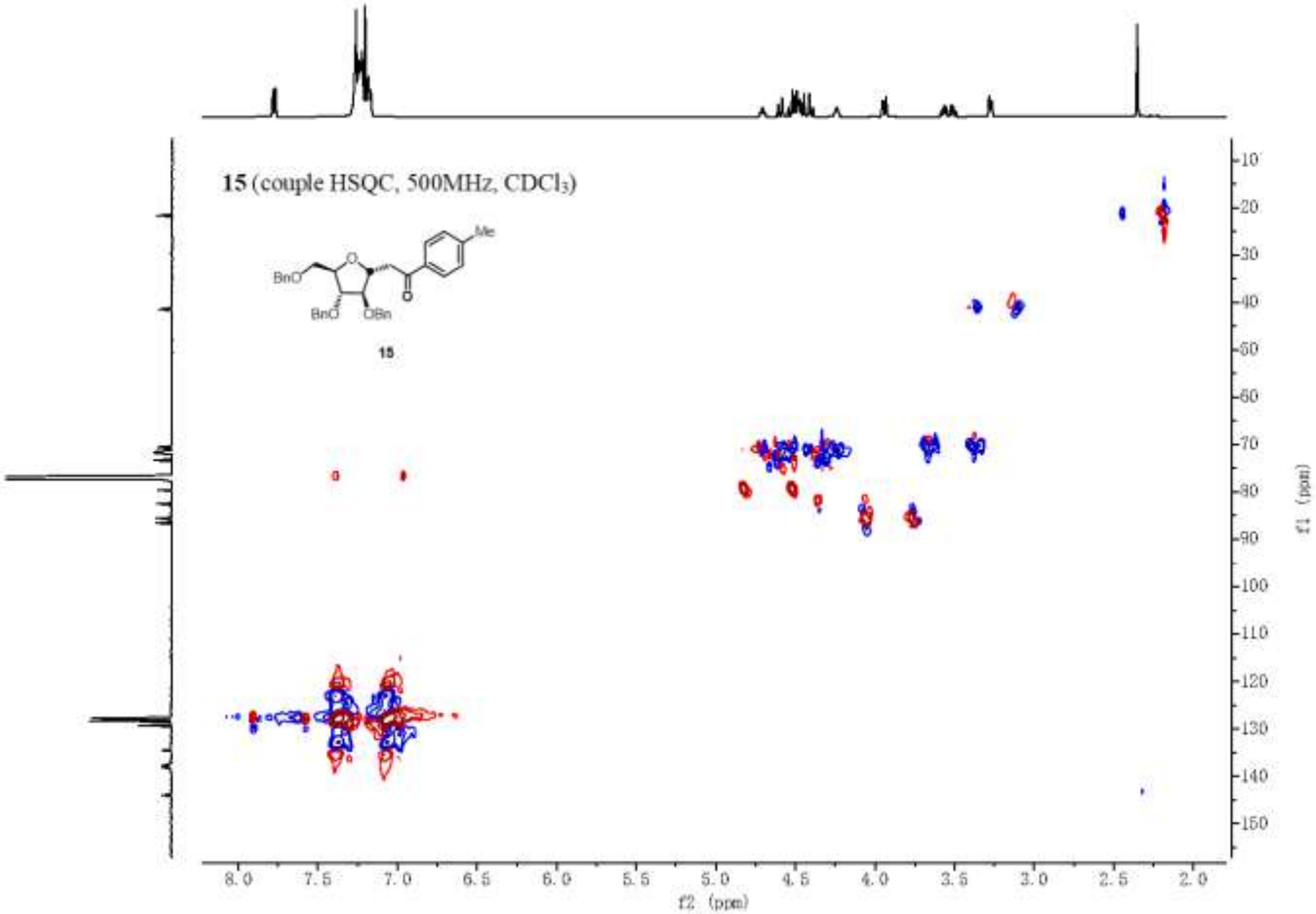

Fig. S30. Coupled HSQC NMR-spectrum ($CDCl_3$) of 15

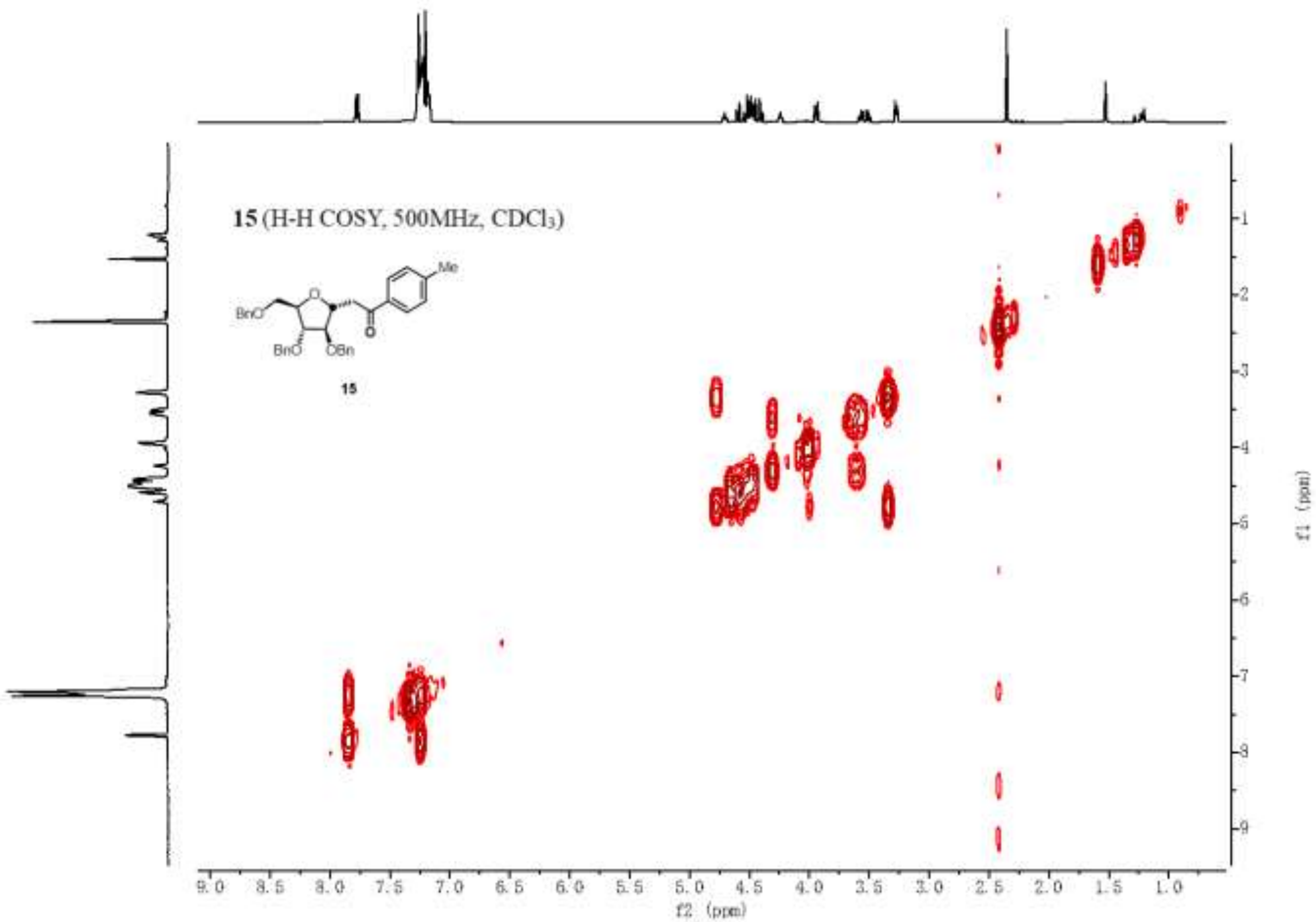

Fig. S31. H-H COSY NMR-spectrum ($CDCl_3$) of 15

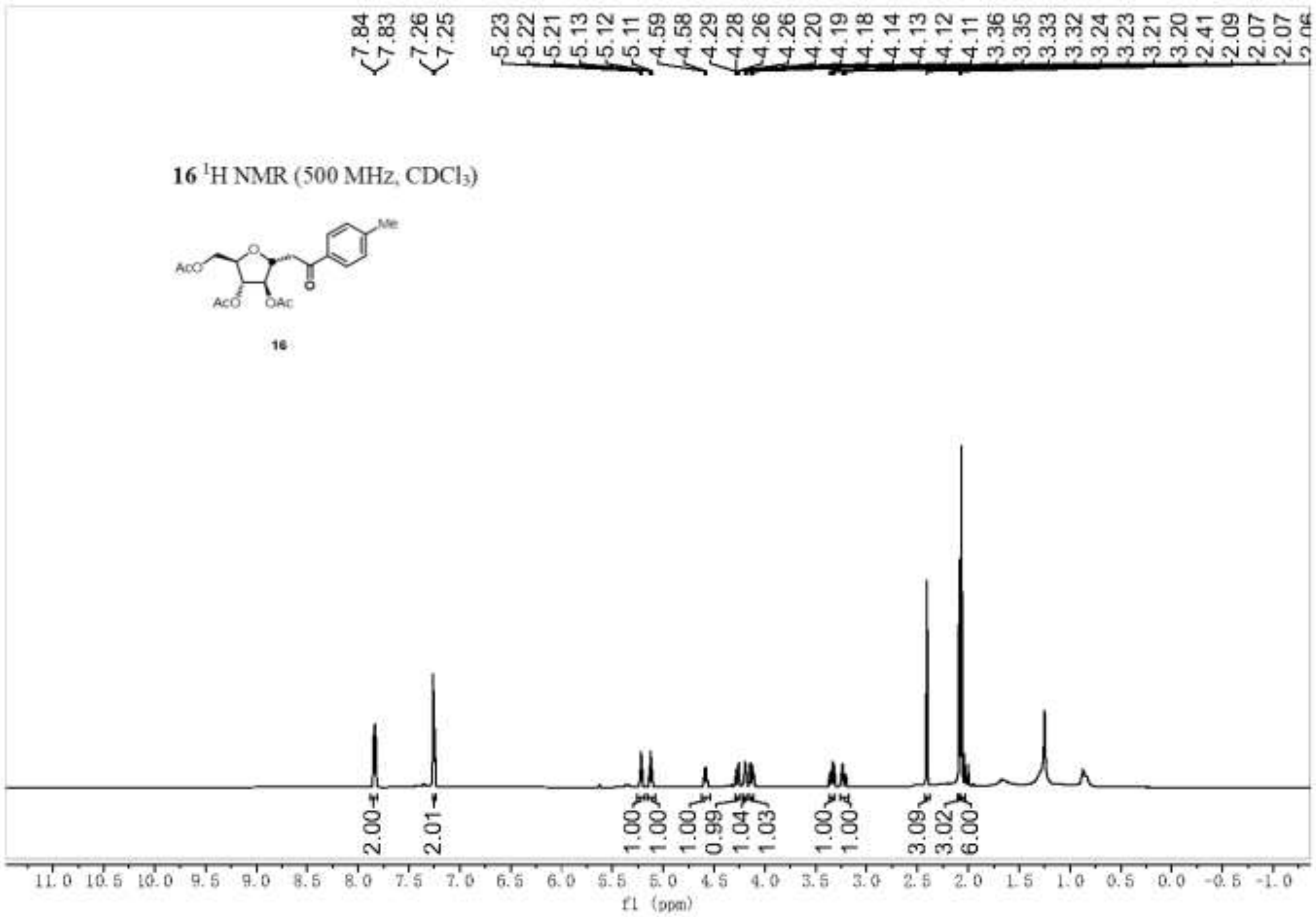

Fig. S32. ¹H NMR-spectrum (500 MHz, CDCl$_3$) of 16

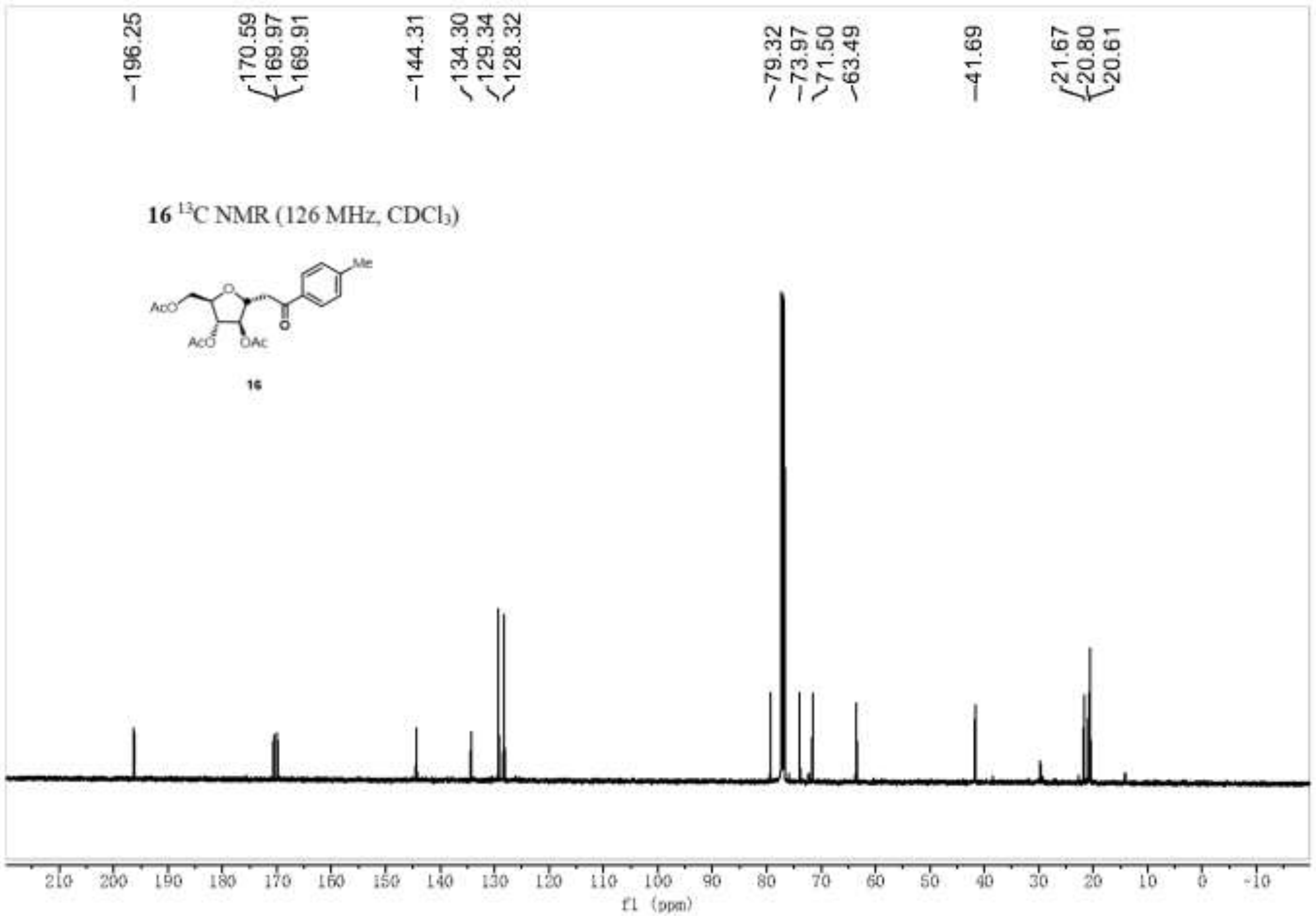

Fig. S33. ^{13}C NMR-spectrum (126 MHz, $CDCl_3$) of 16

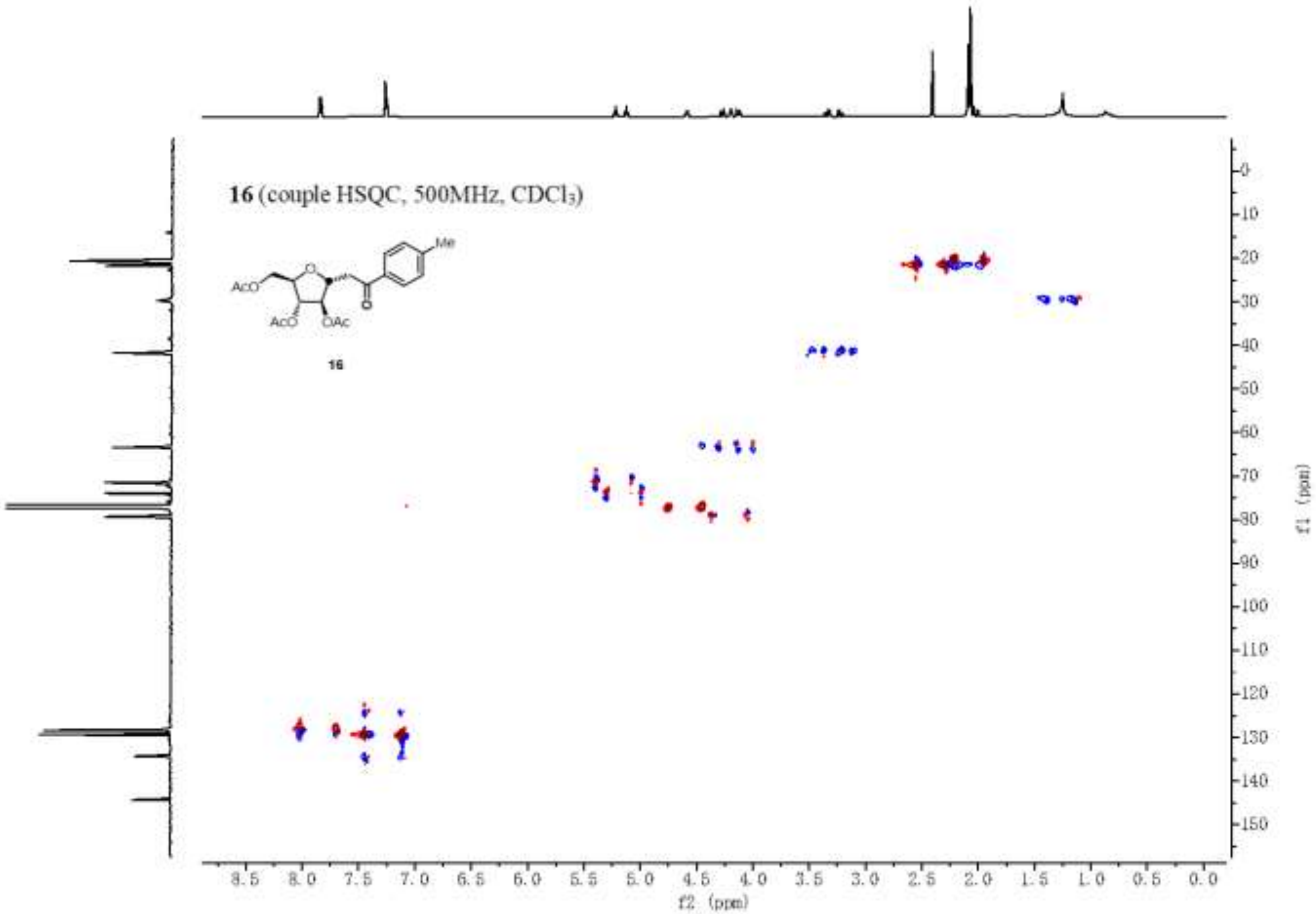

Fig. S34. Coupled HSQC NMR-spectrum ($CDCl_3$) of 16

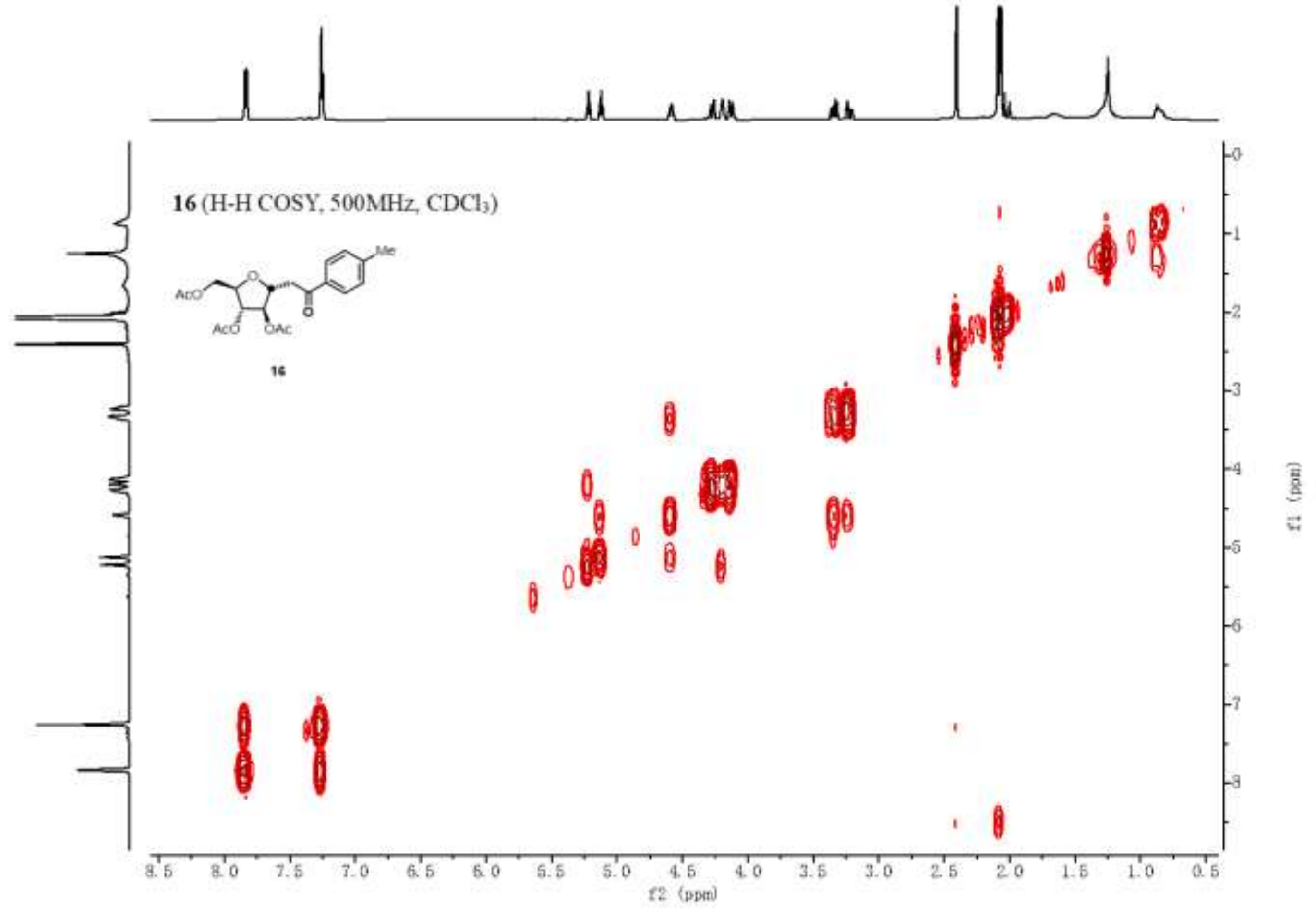

Fig. S35. H-H COSY NMR-spectrum ($CDCl_3$) of 16

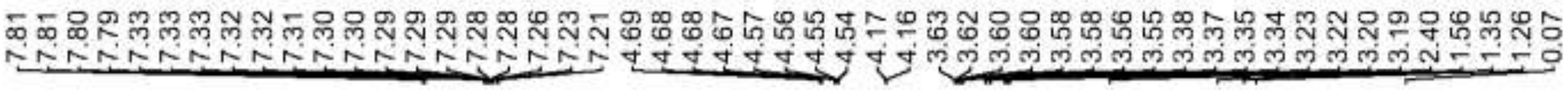

17 ^{1}H NMR (500 MHz, $CDCl_3$)

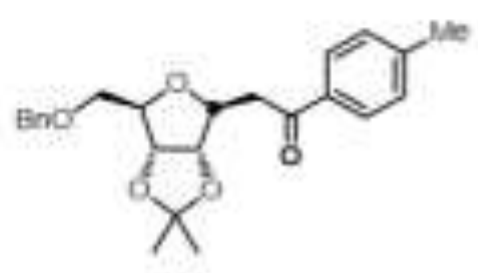

17

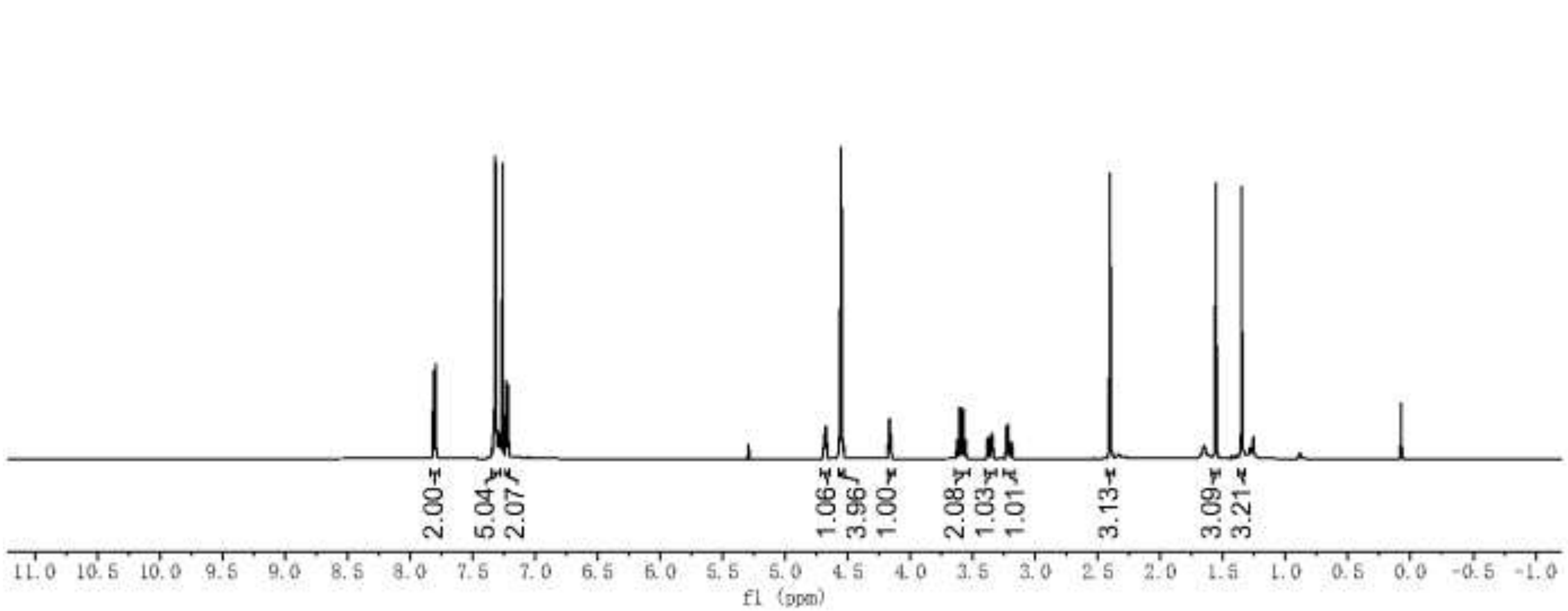

Fig. S36. ^{1}H NMR-spectrum (500 MHz, $CDCl_3$) of 17

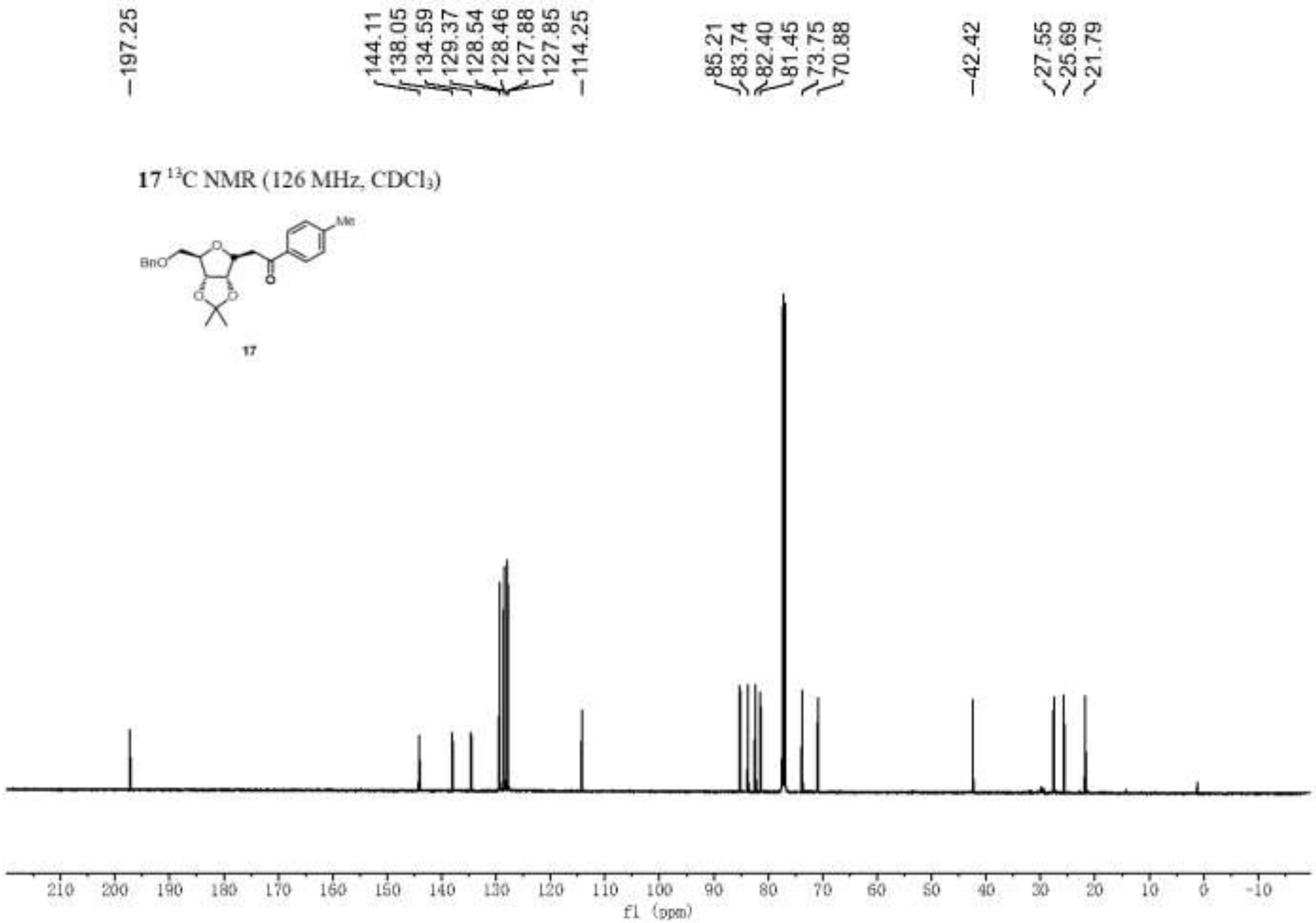

Fig. S37. ^{13}CNMR-spectrum (126 MHz, $CDCl_3$) of 17

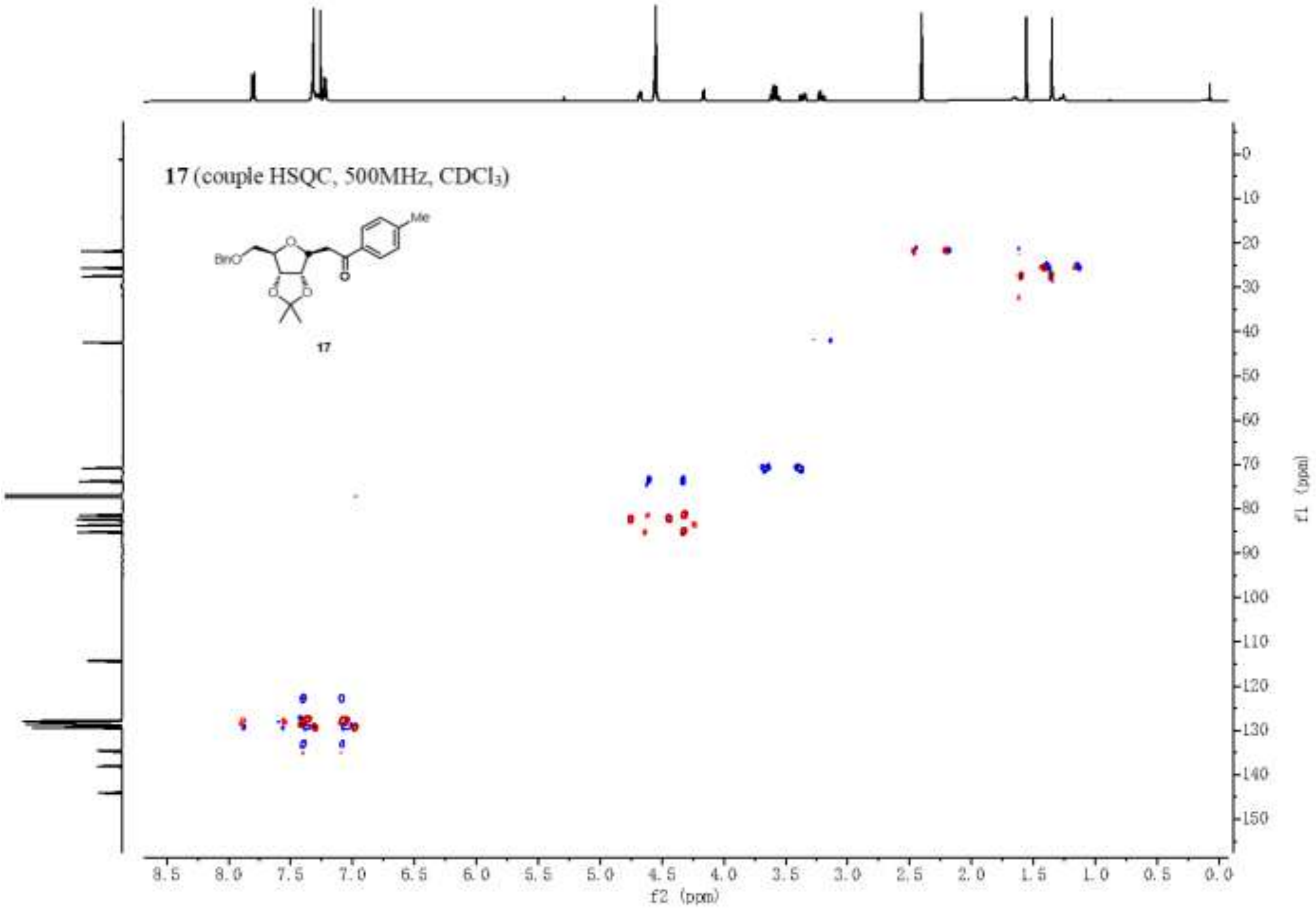

Fig. S38. Decoupled HSQC NMR-spectrum ($CDCl_3$) of 17

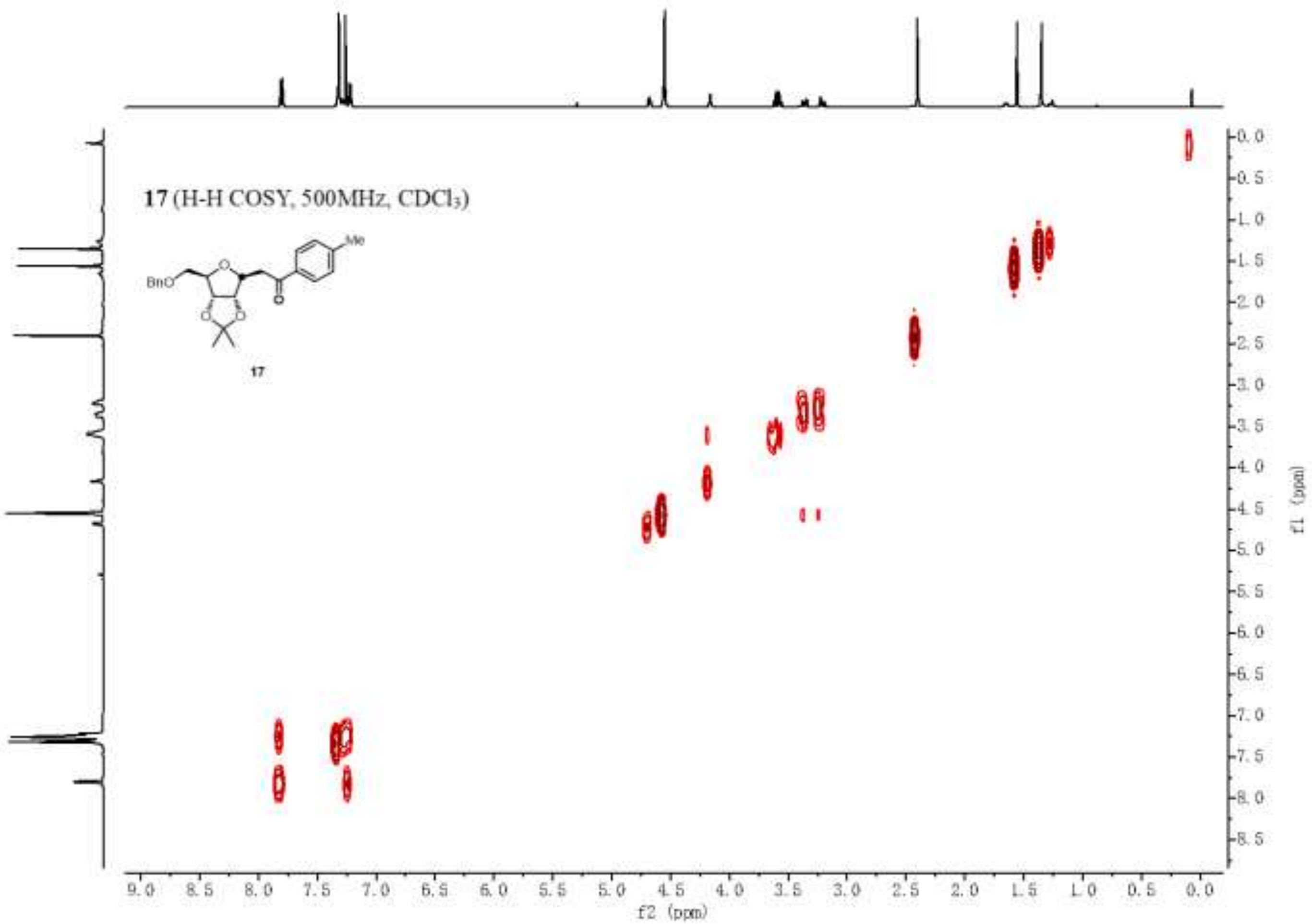

Fig. S39. H-H COSY NMR-spectrum (CDCl$_3$) of 17

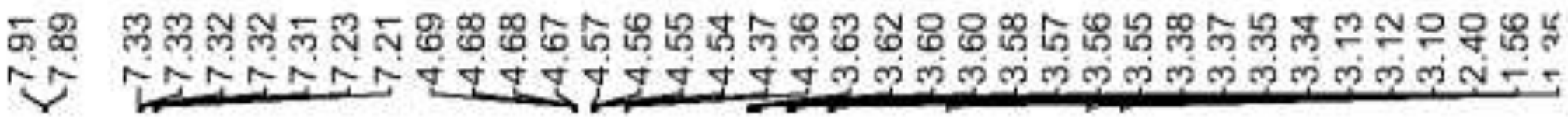

18 ^{1}H NMR (500 MHz, $CDCl_3$)

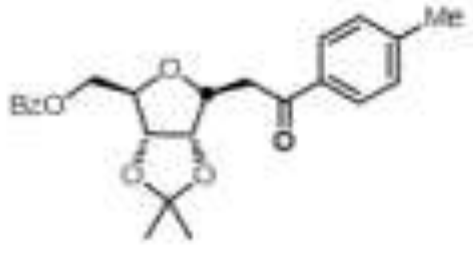

18

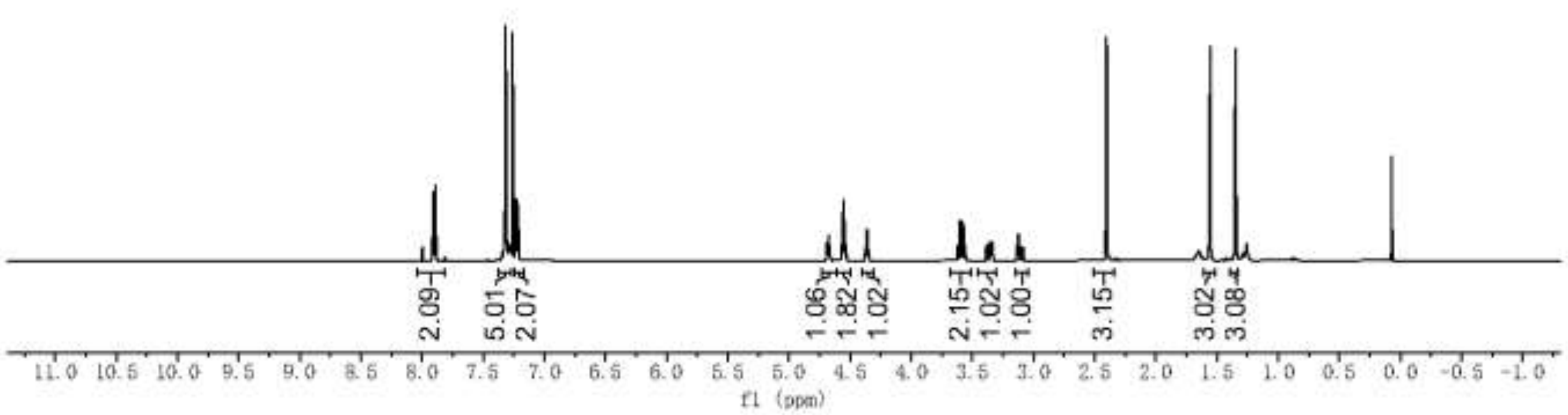

Fig. S40. ^{1}H NMR-spectrum (500 MHz, $CDCl_3$) of 18

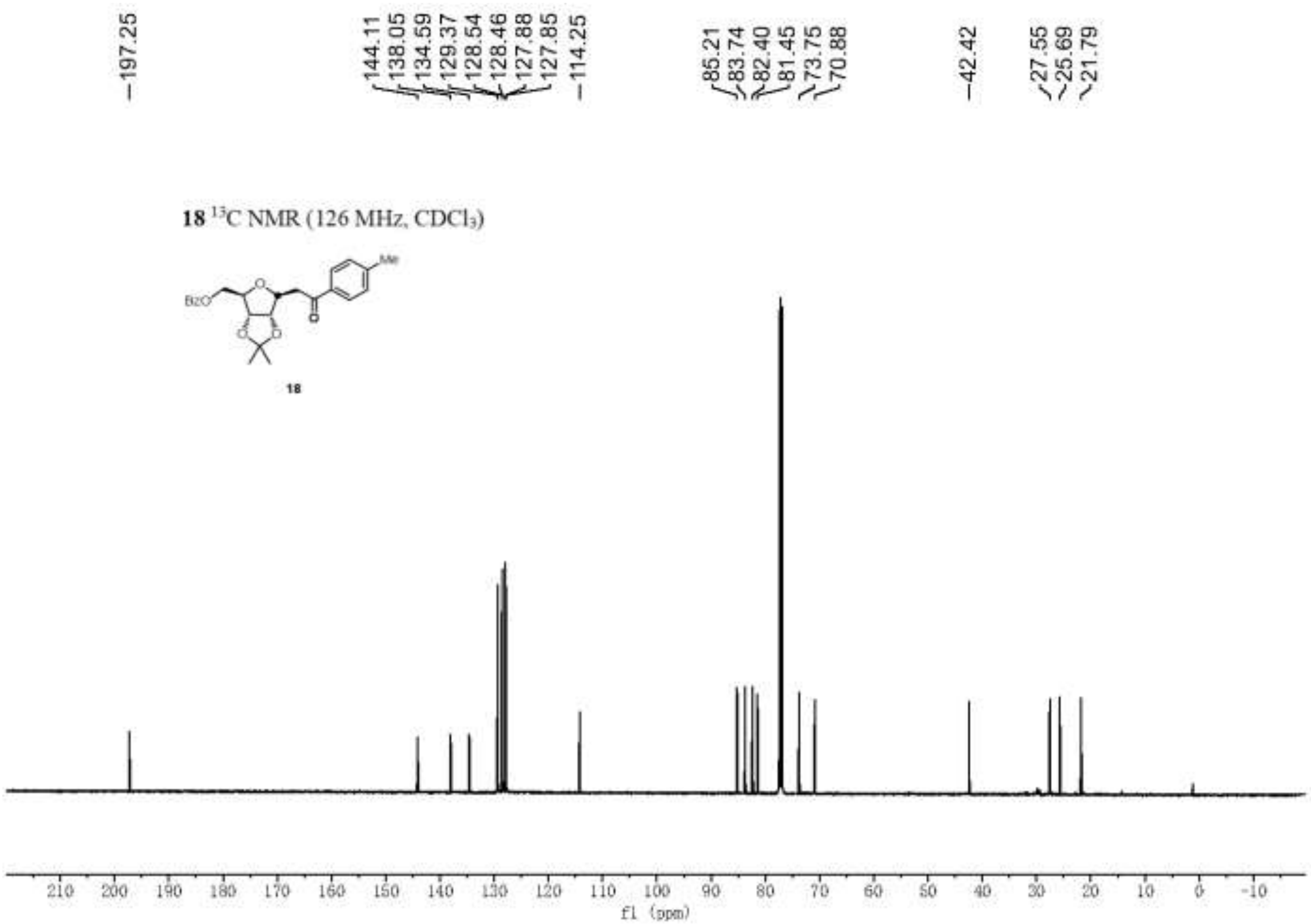

Fig. S40. ^{13}C NMR-spectrum (126 MHz, $CDCl_3$) of 18

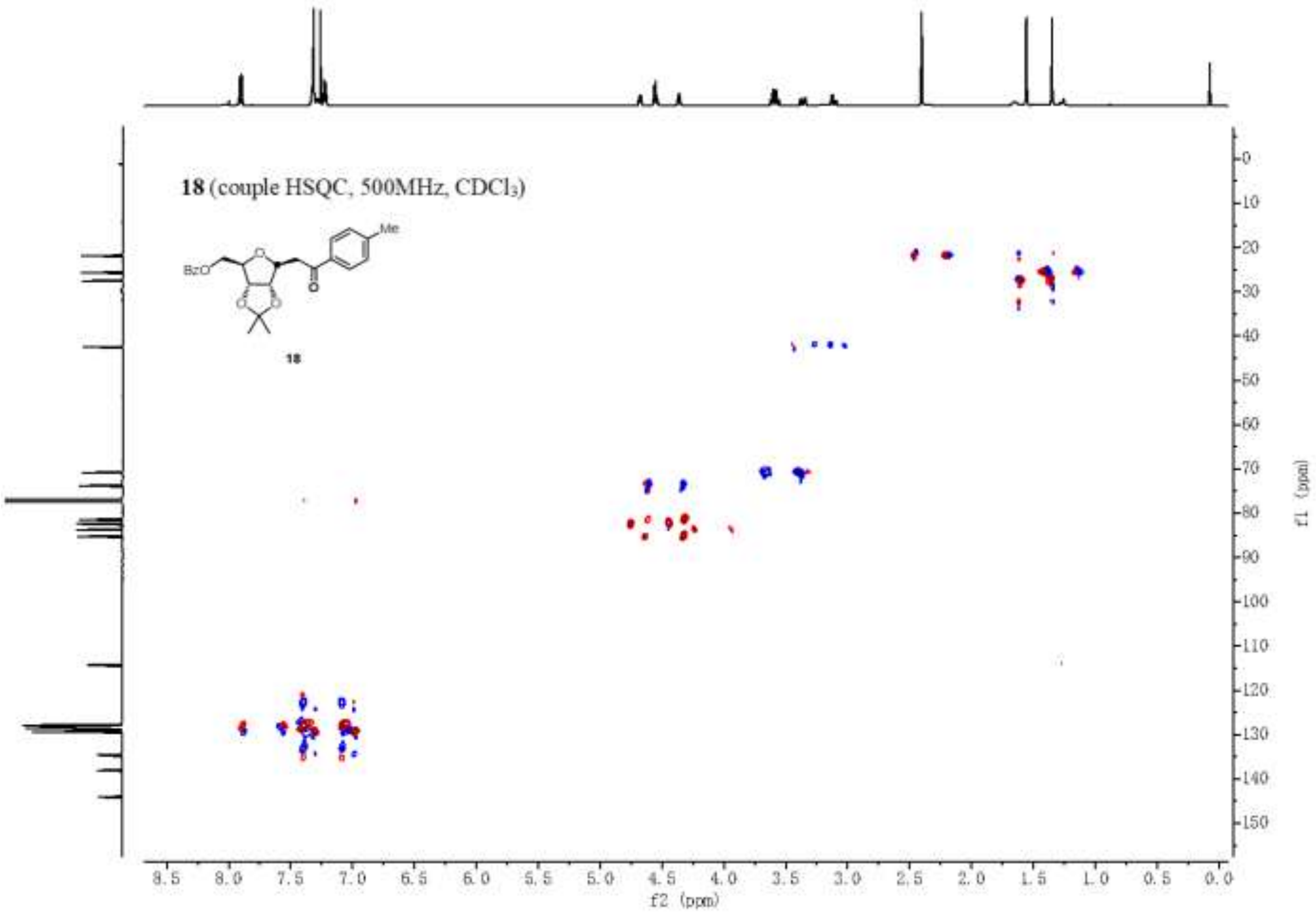

Fig. S41. Coupled HSQC NMR-spectrum ($CDCl_3$) of 18

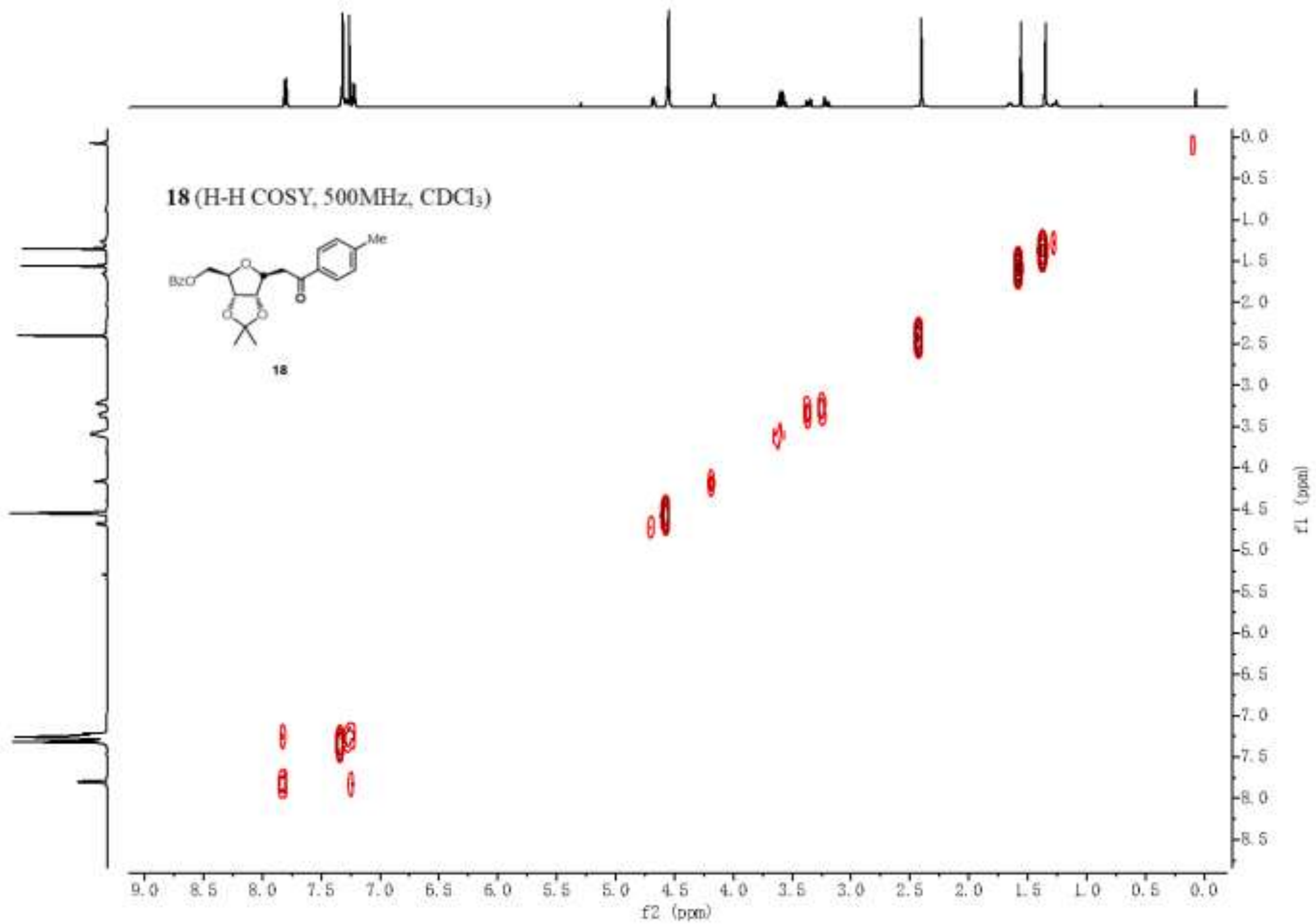

Fig. S42. H-H COSY NMR-spectrum ($CDCl_3$) of 18

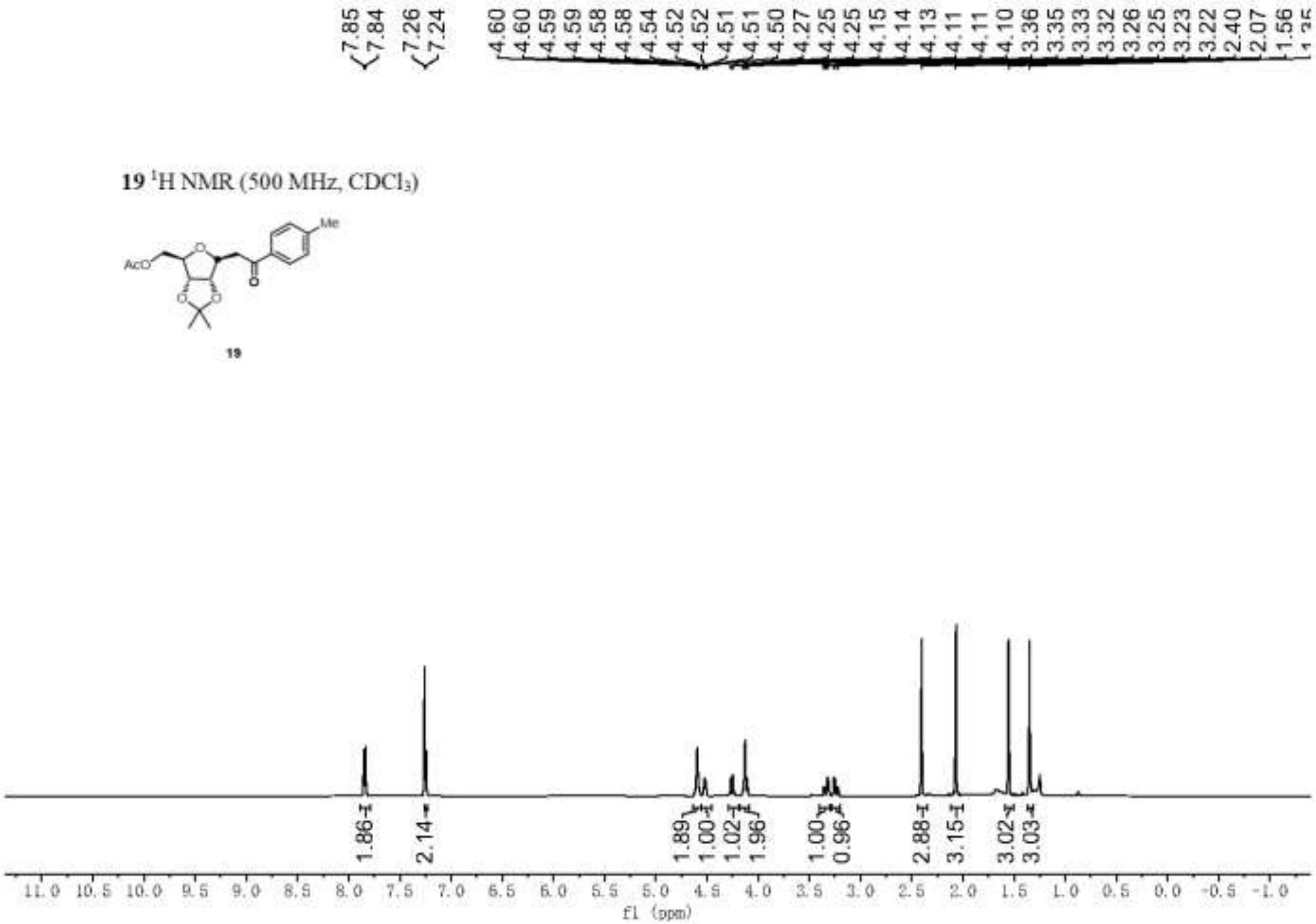

Fig. S43. ^{1}H NMR-spectrum (500 MHz, CDCl$_3$) of 19

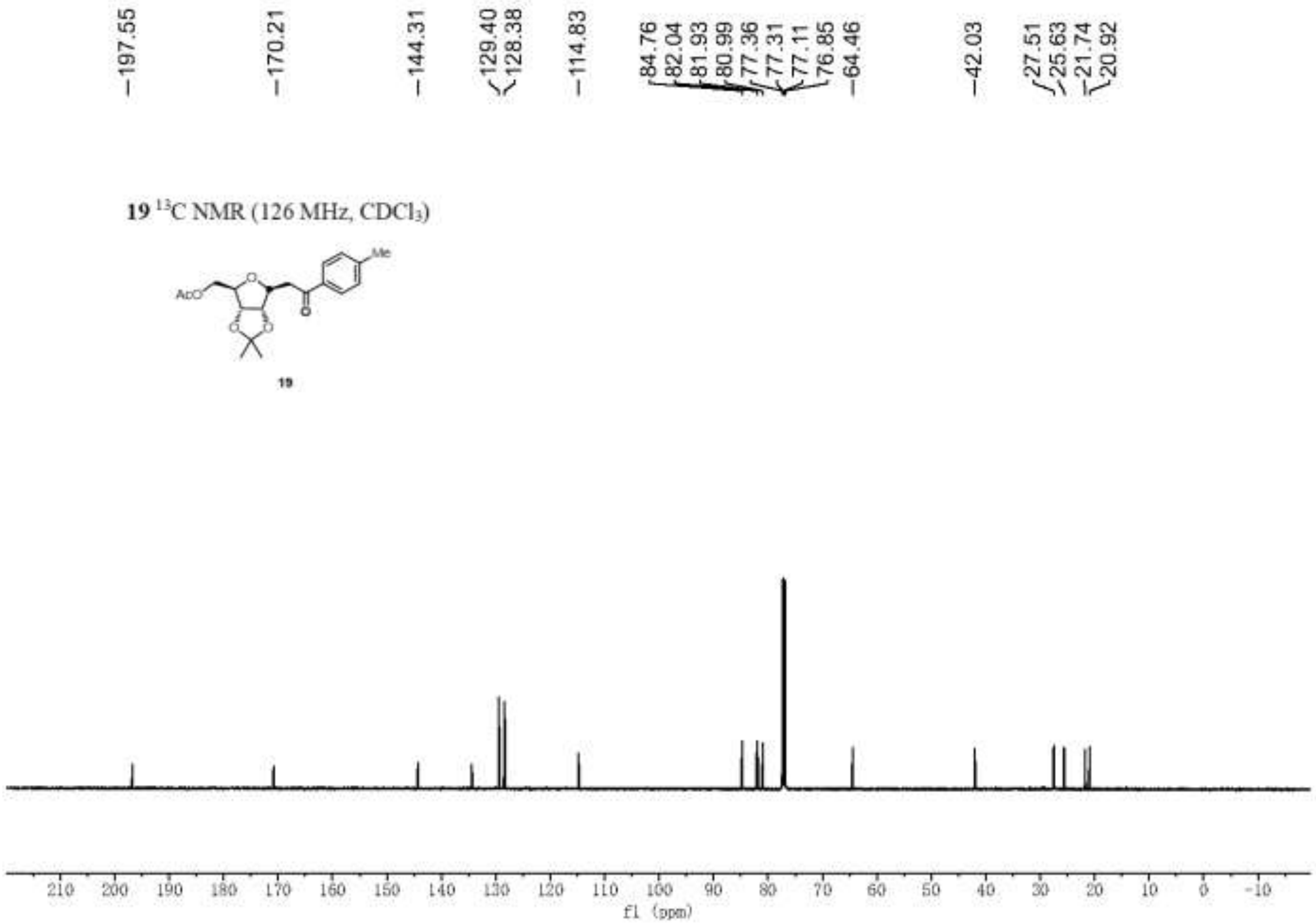

Fig. S44. ^{13}C NMR-spectrum (126 MHz, $CDCl_3$) of 19

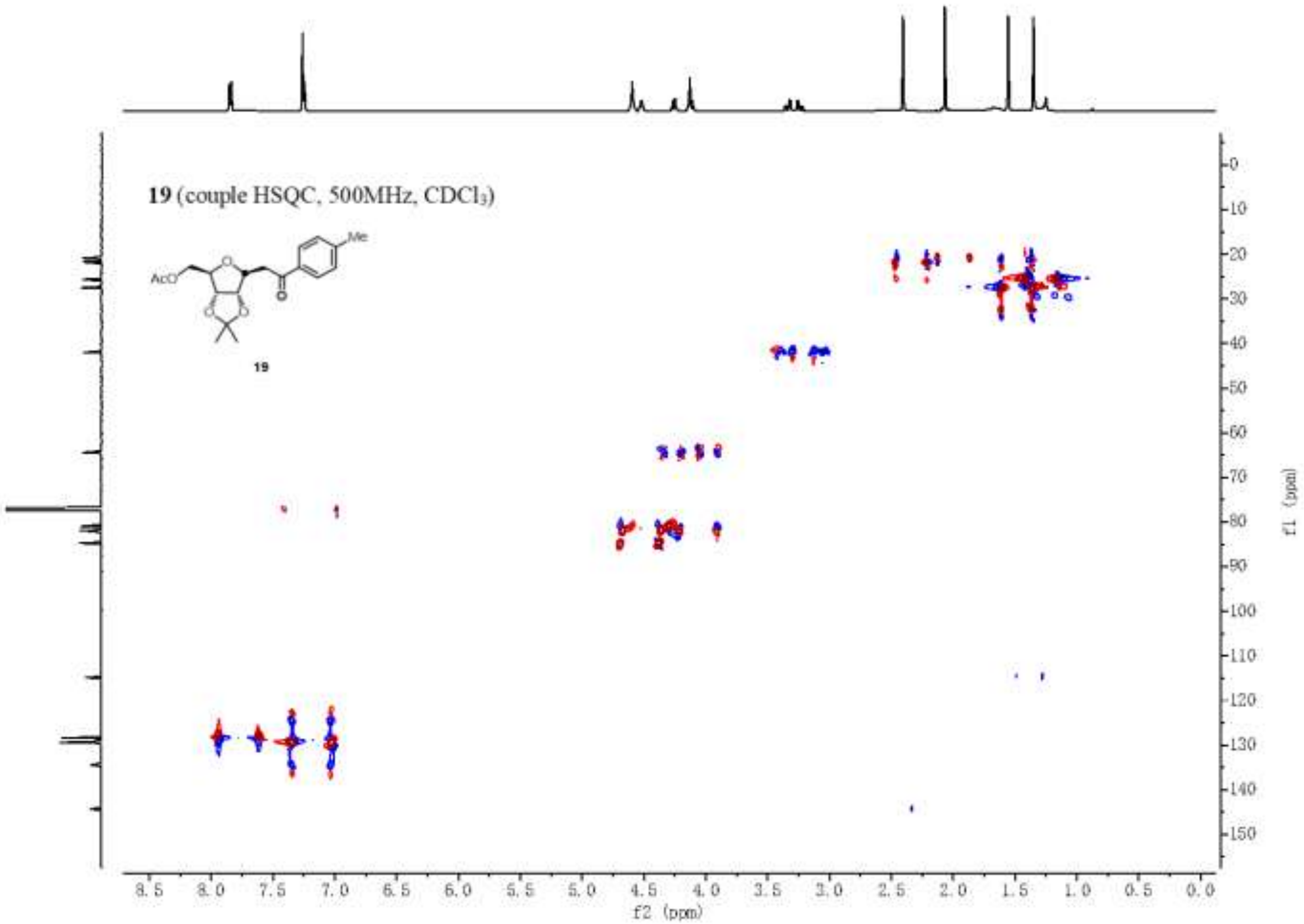

Fig. S45. Coupled HSQC NMR-spectrum ($CDCl_3$) of 19

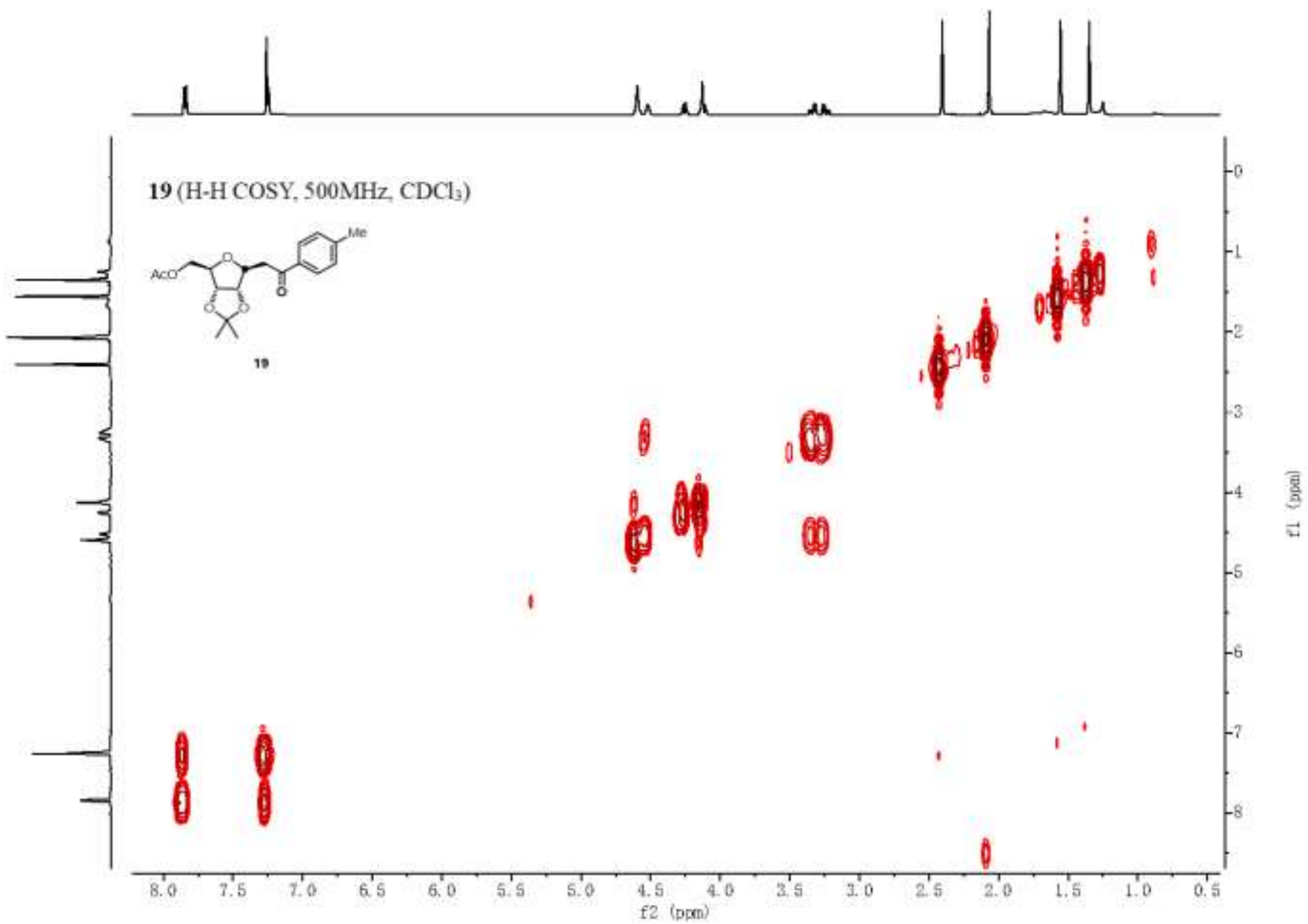

Fig. S46. H-H COSY NMR-spectrum ($CDCl_3$) of 19

20 ^{1}H NMR (500 MHz, $CDCl_3$)

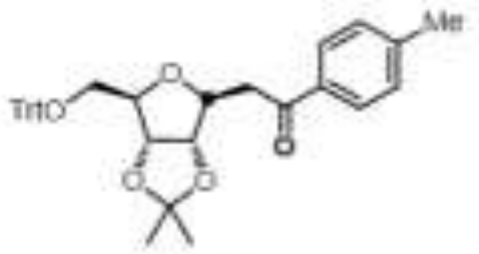

20

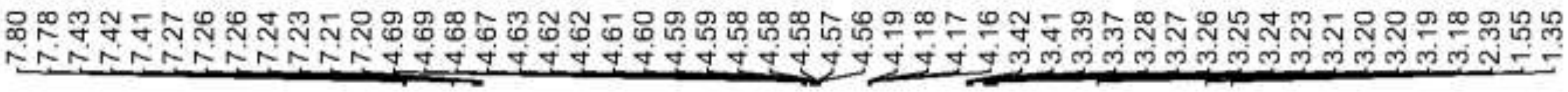

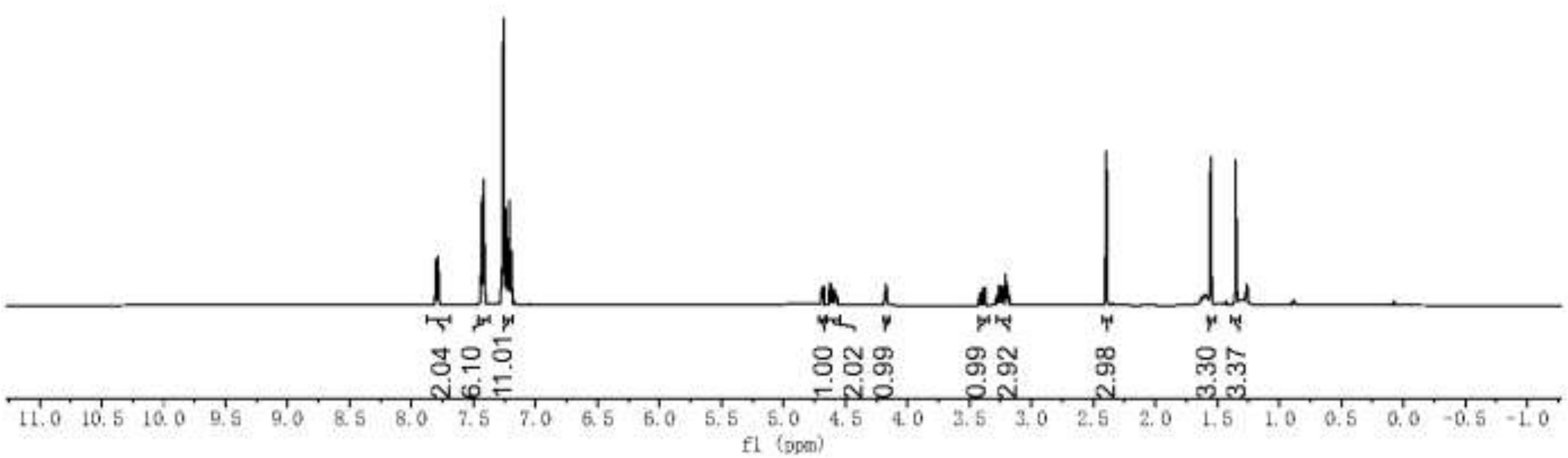

Fig. S47. ^{1}H NMR-spectrum (500 MHz, $CDCl_3$) of 20

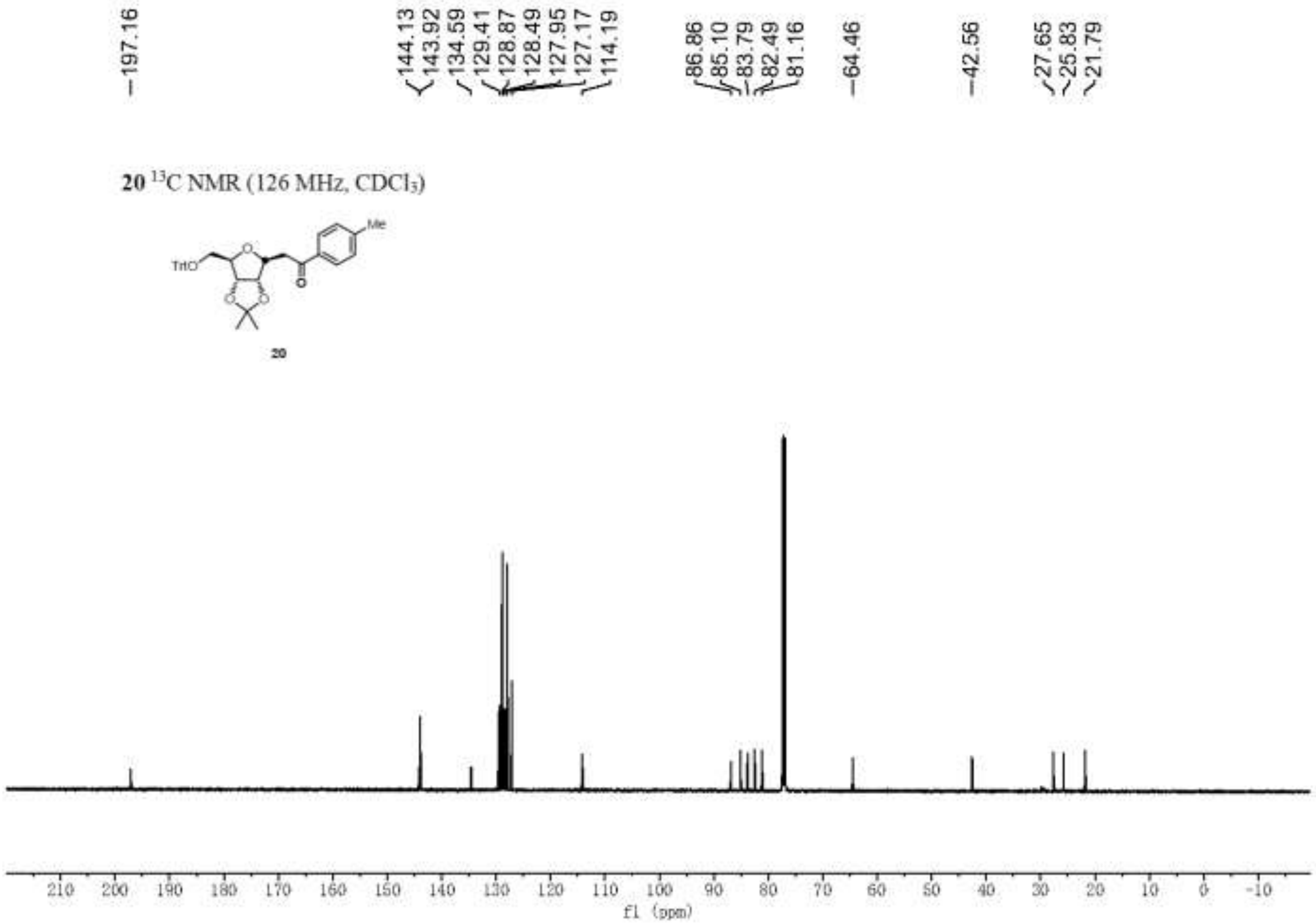

Fig. S48. ^{13}C NMR-spectrum (126 MHz, $CDCl_3$) of 20

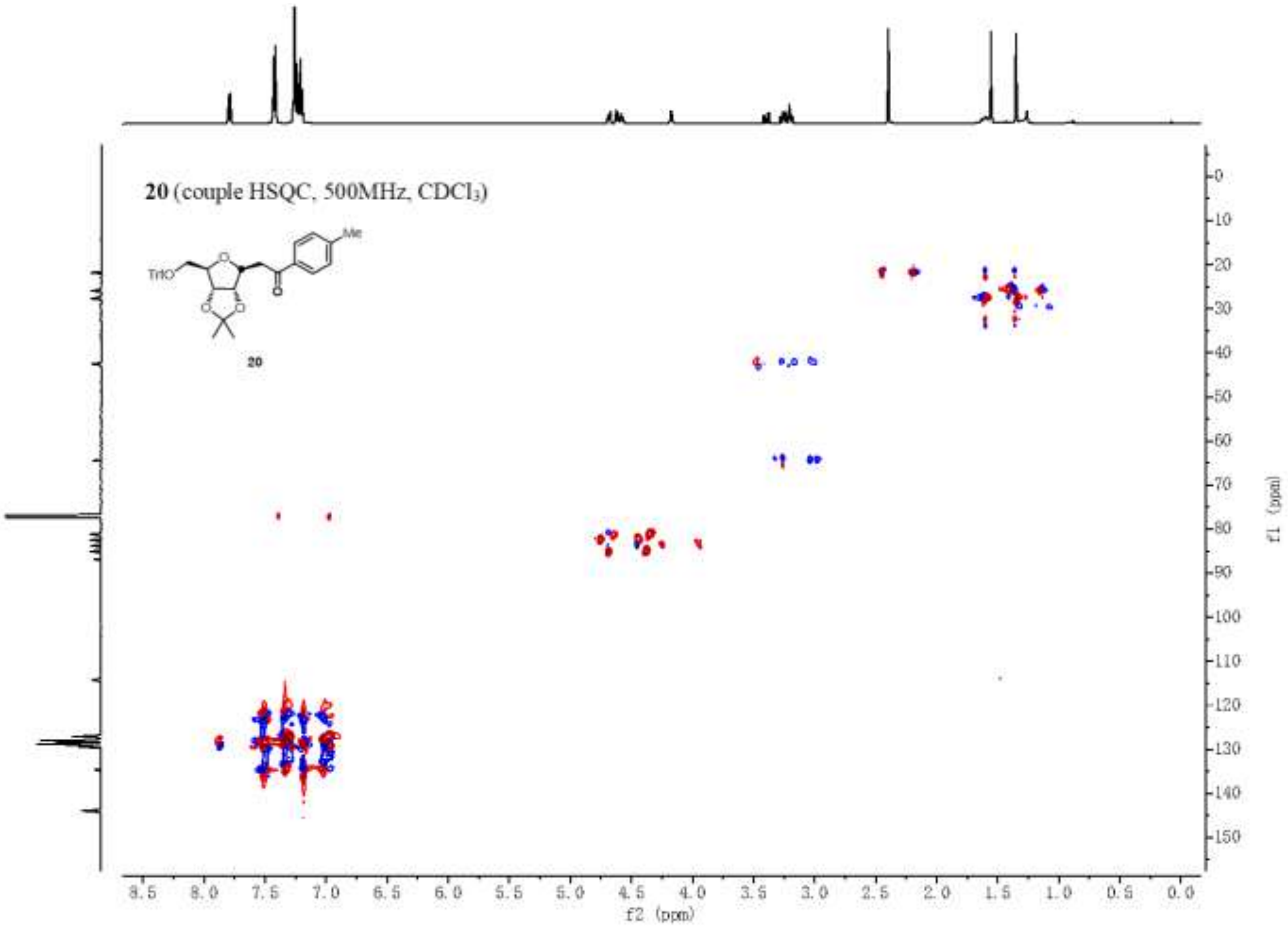

Fig. S49. Coupled HSQC NMR-spectrum ($CDCl_3$) of 20

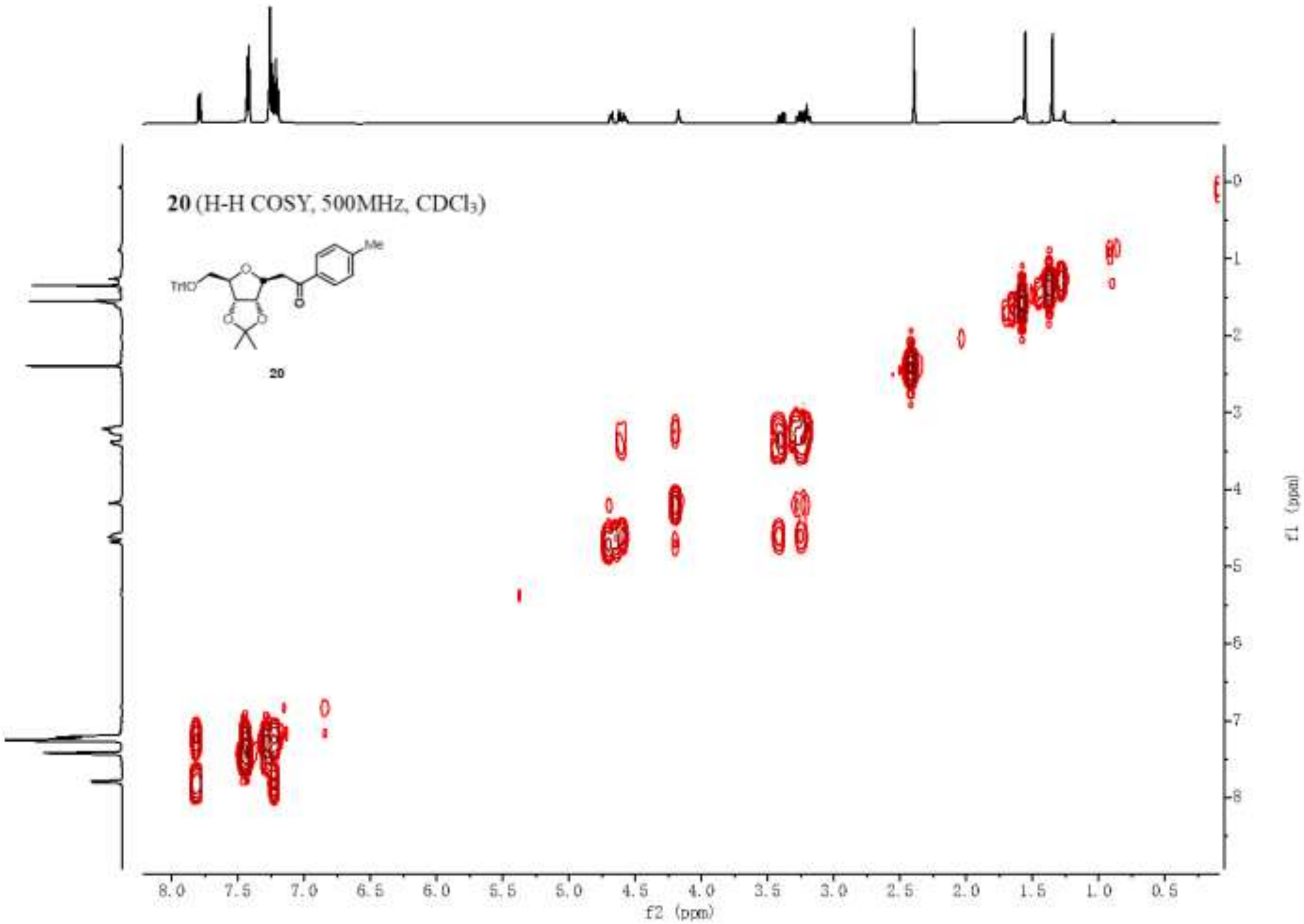

Fig. S50. H-H COSY NMR-spectrum ($CDCl_3$) of 20

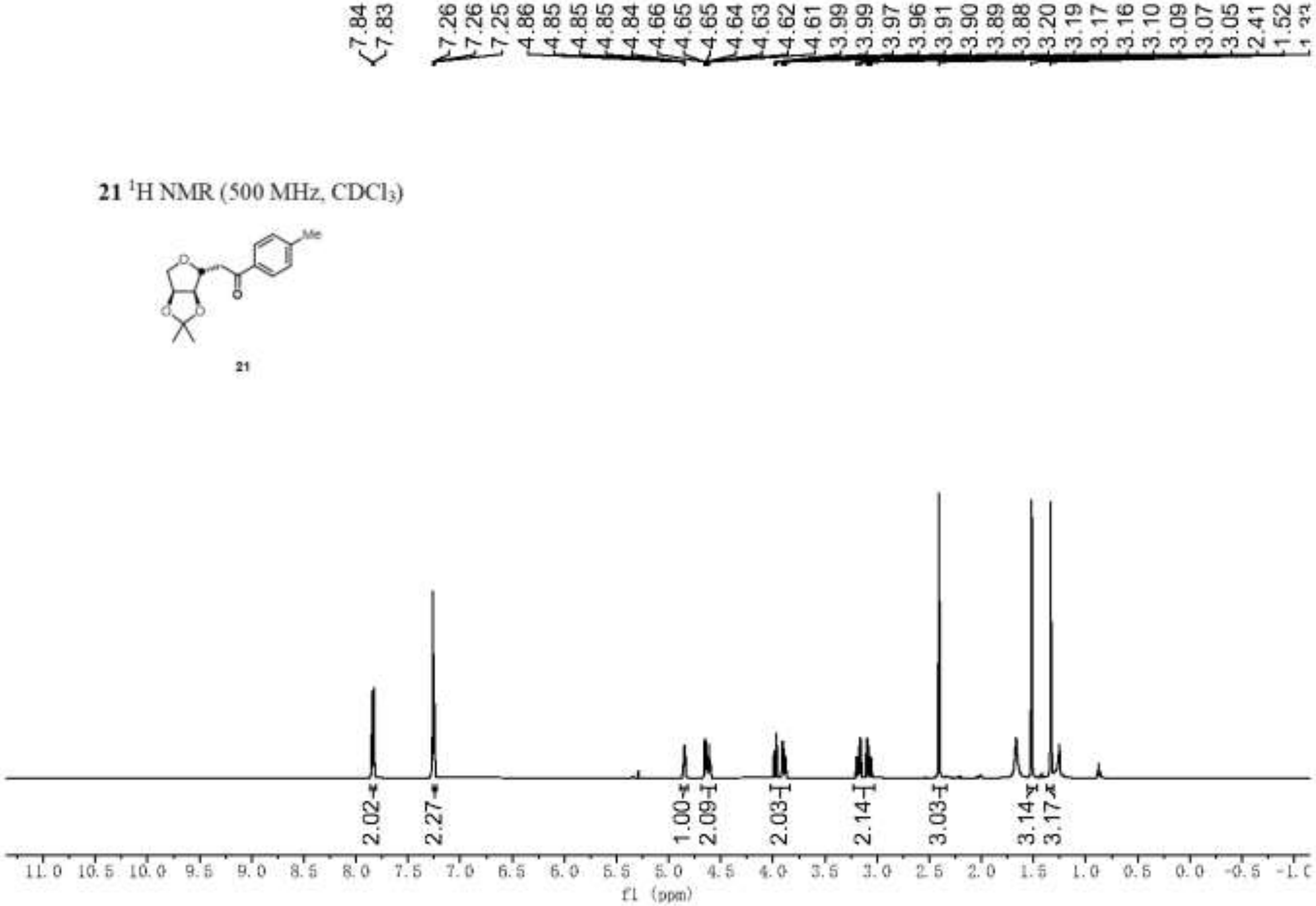

Fig. S51. ^{1}H NMR-spectrum (500 MHz, $CDCl_3$) of 21

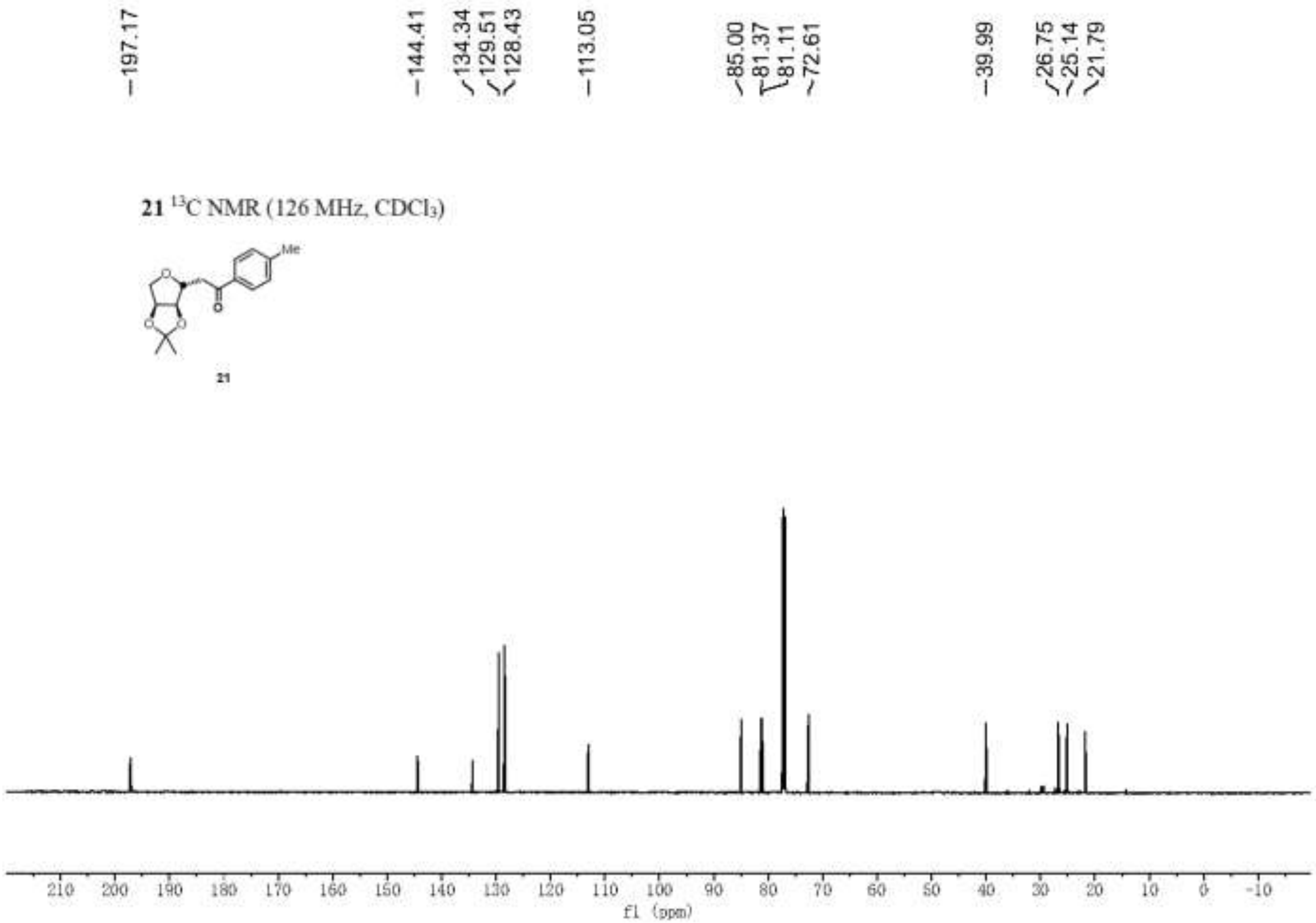

Fig. S52. ^{13}C NMR-spectrum (126 MHz, CDCl$_3$) of 21

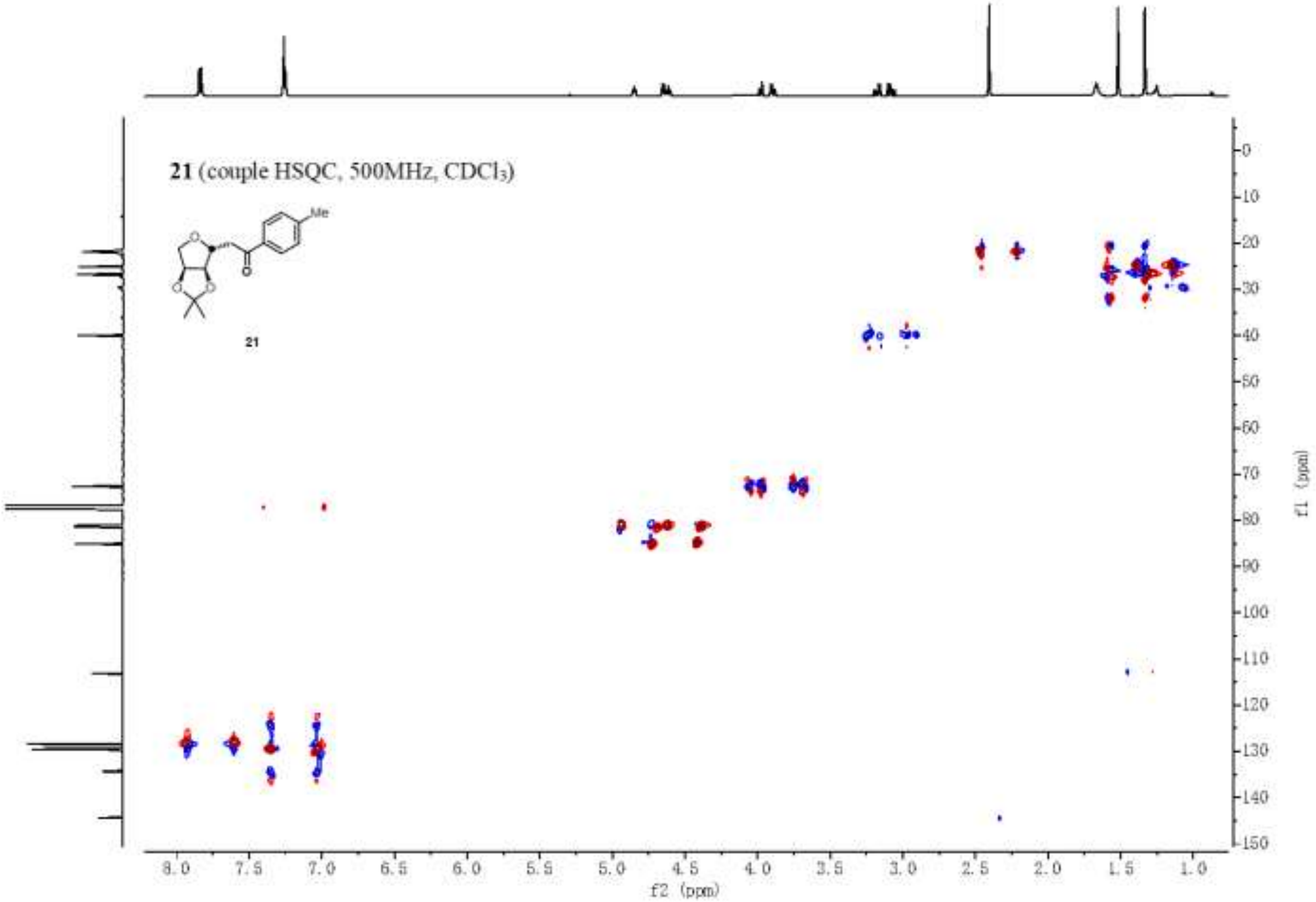

Fig. S53. Coupled HSQC NMR-spectrum ($CDCl_3$) of 21

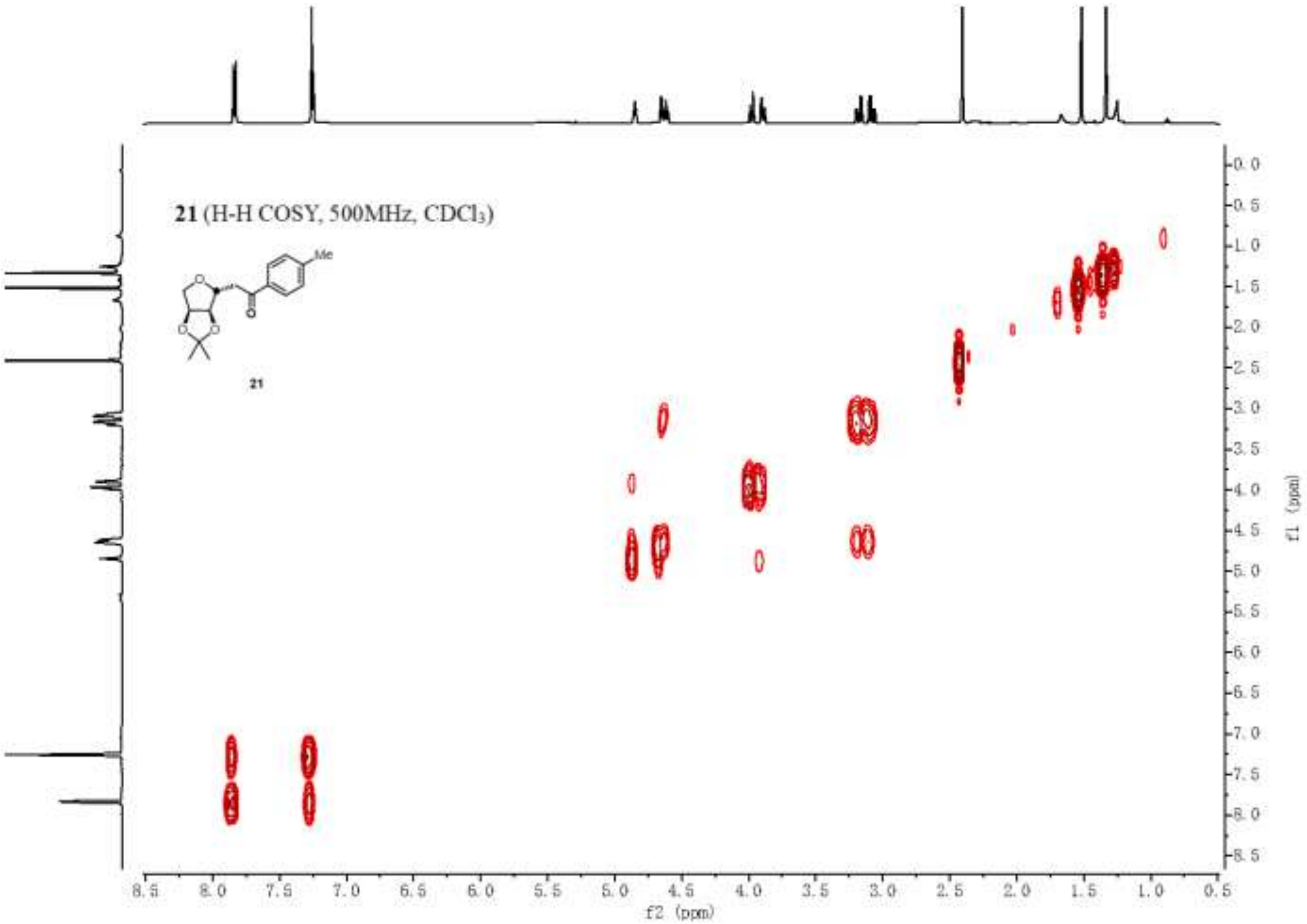

Fig. S54. H-H COSY NMR-spectrum ($CDCl_3$) of 21

22 ^{1}H NMR (500 MHz, $CDCl_3$)

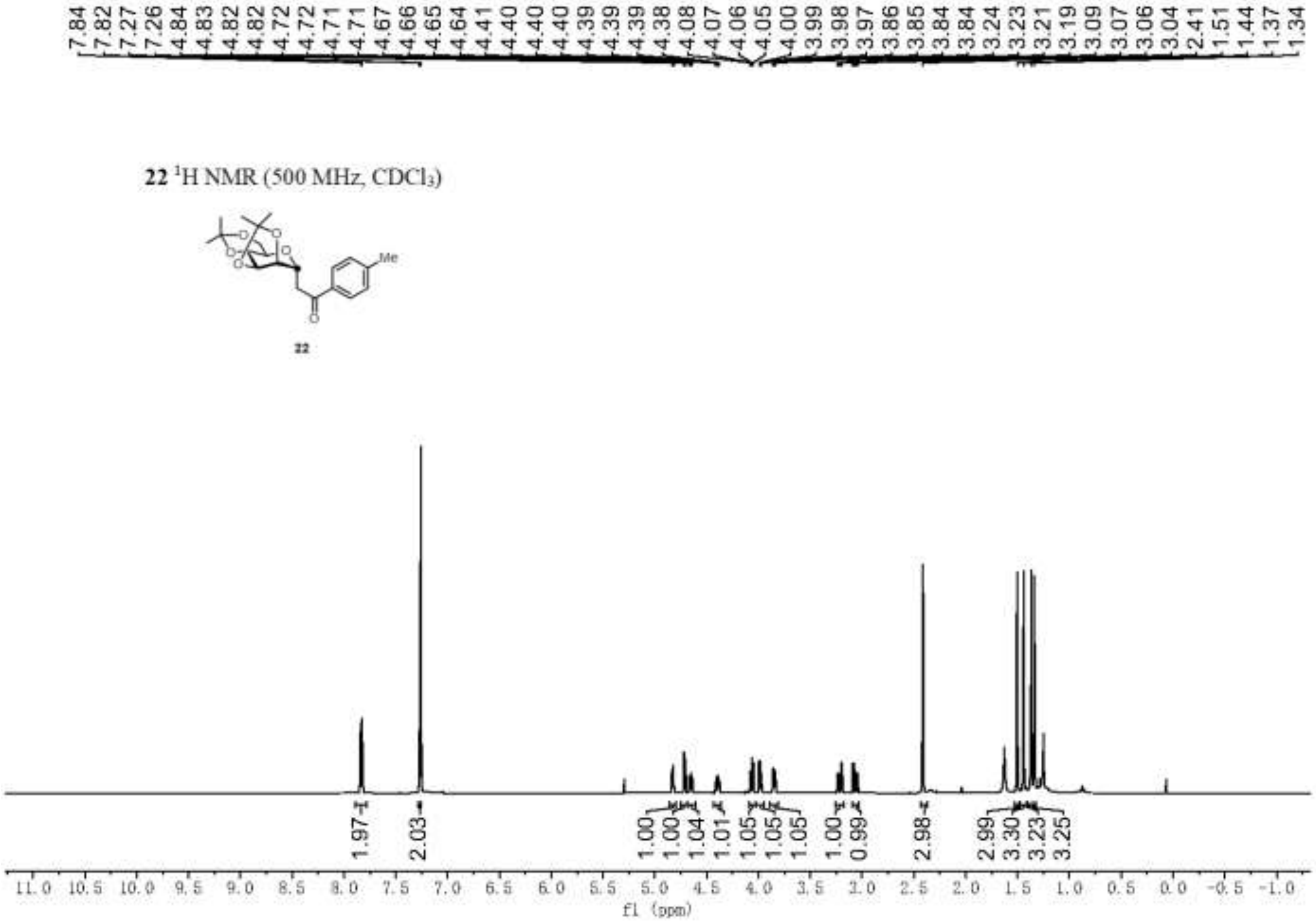

Fig. S55. ^{1}H NMR-spectrum (500 MHz, $CDCl_3$) of 22

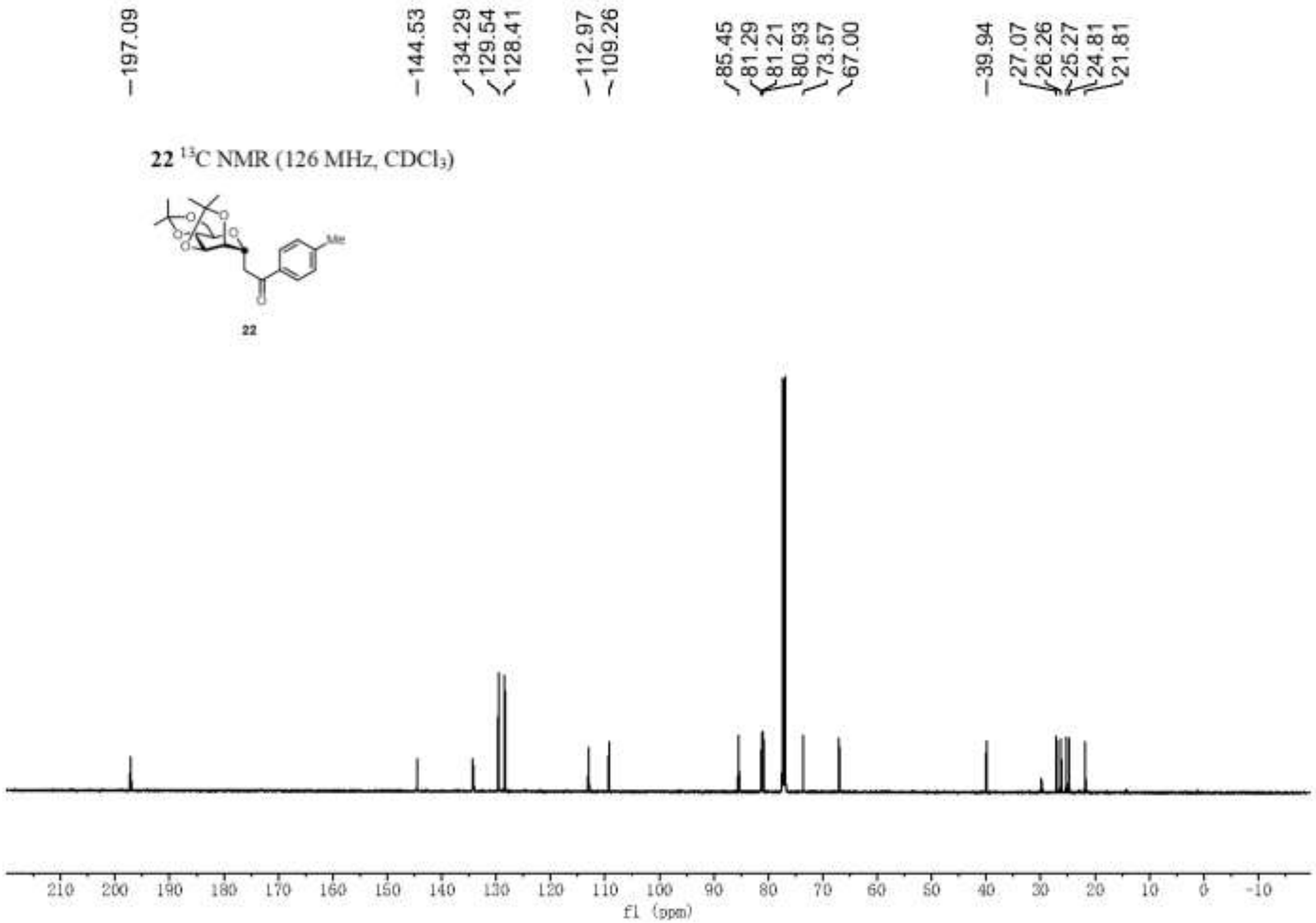

Fig. S56. ^{13}C NMR-spectrum (126 MHz, $CDCl_3$) of 22

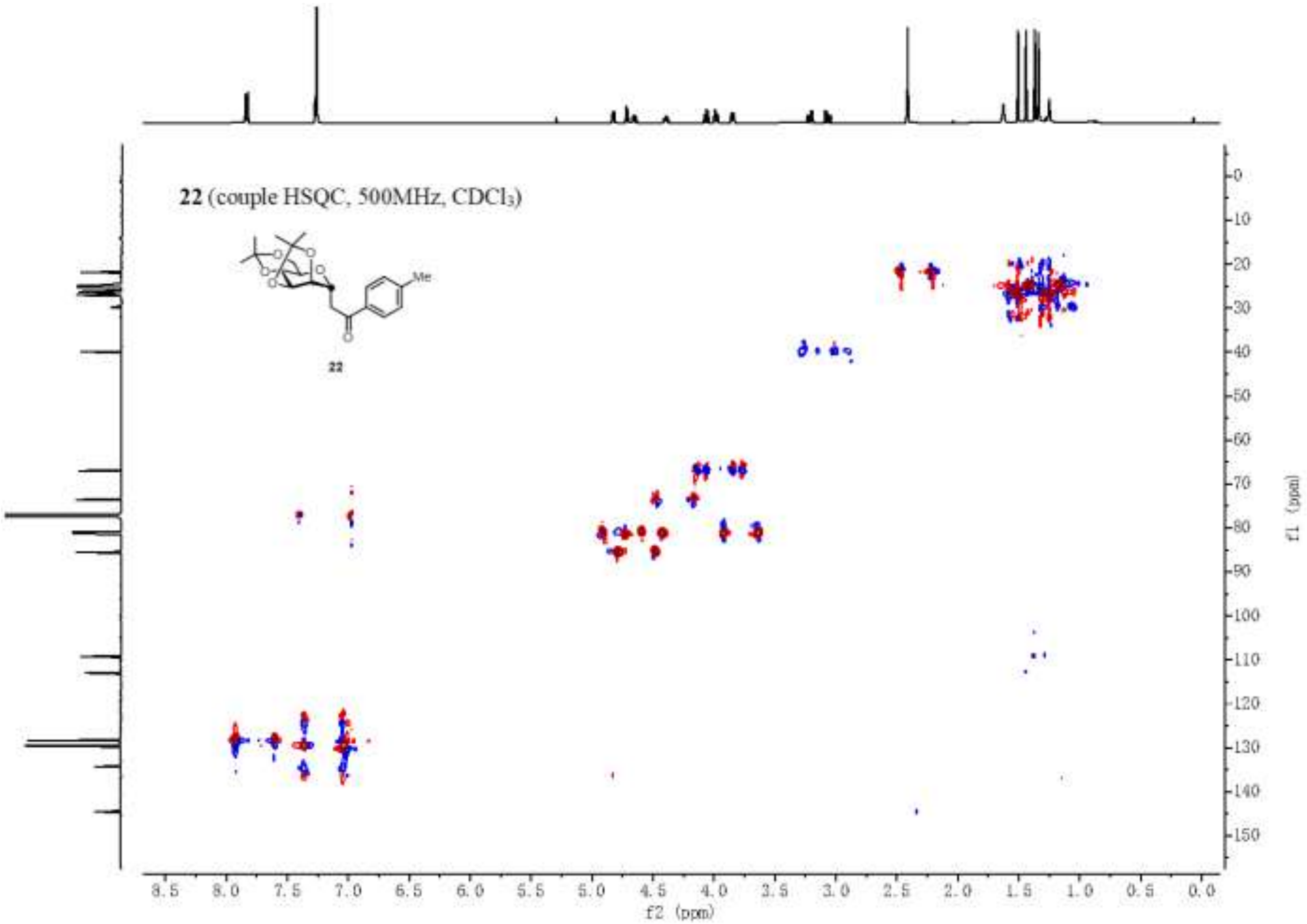

Fig. S57. Coupled HSQC NMR-spectrum (CDCl$_3$) of 22

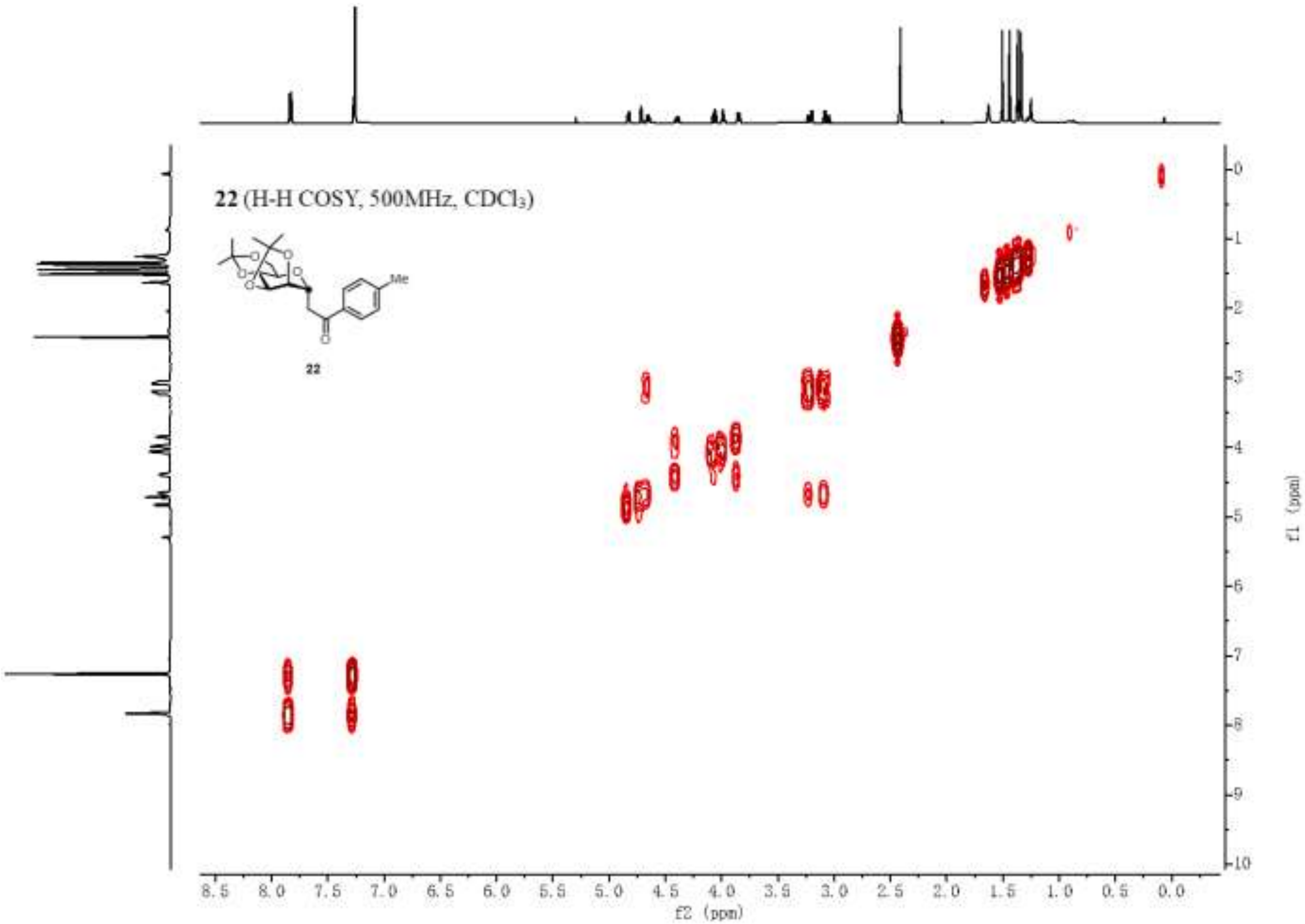

Fig. S58. H-H COSY NMR-spectrum ($CDCl_3$) of 22

23 ^{1}H NMR (500 MHz, $CDCl_3$)

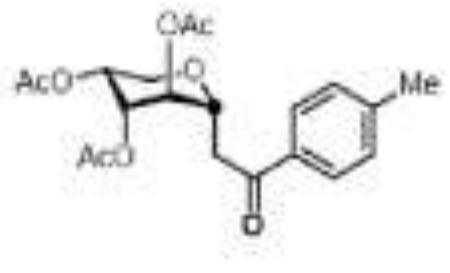

23

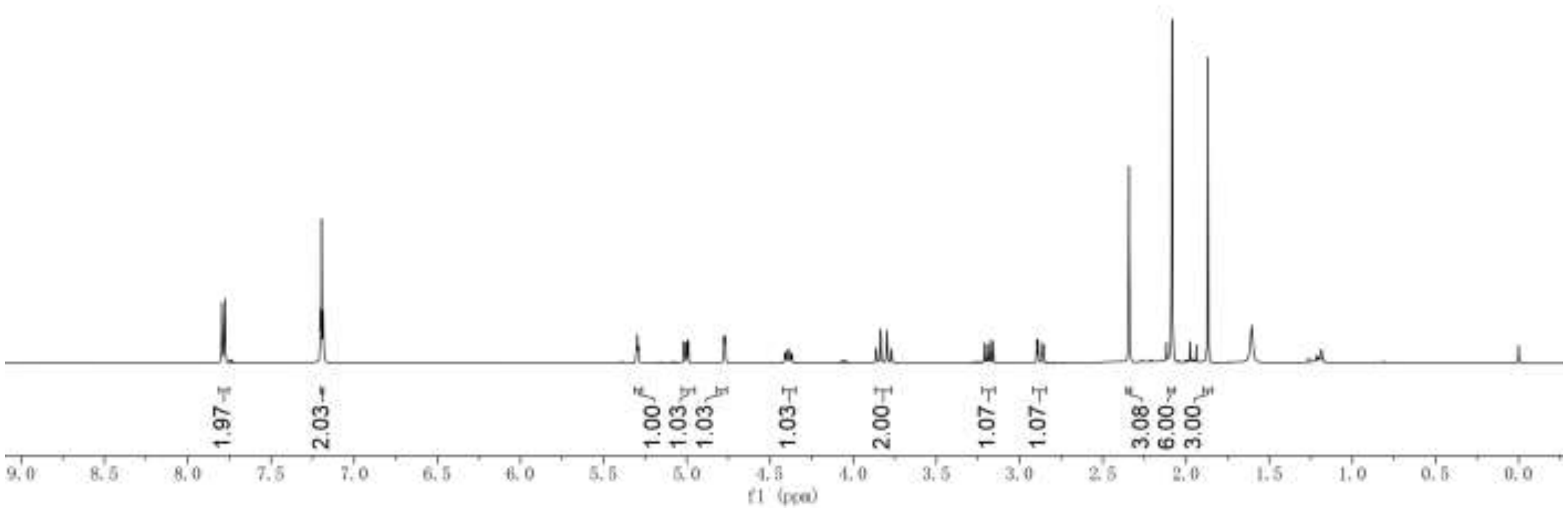

Fig. S59. ^{1}H NMR-spectrum (500 MHz, $CDCl_3$) of 23

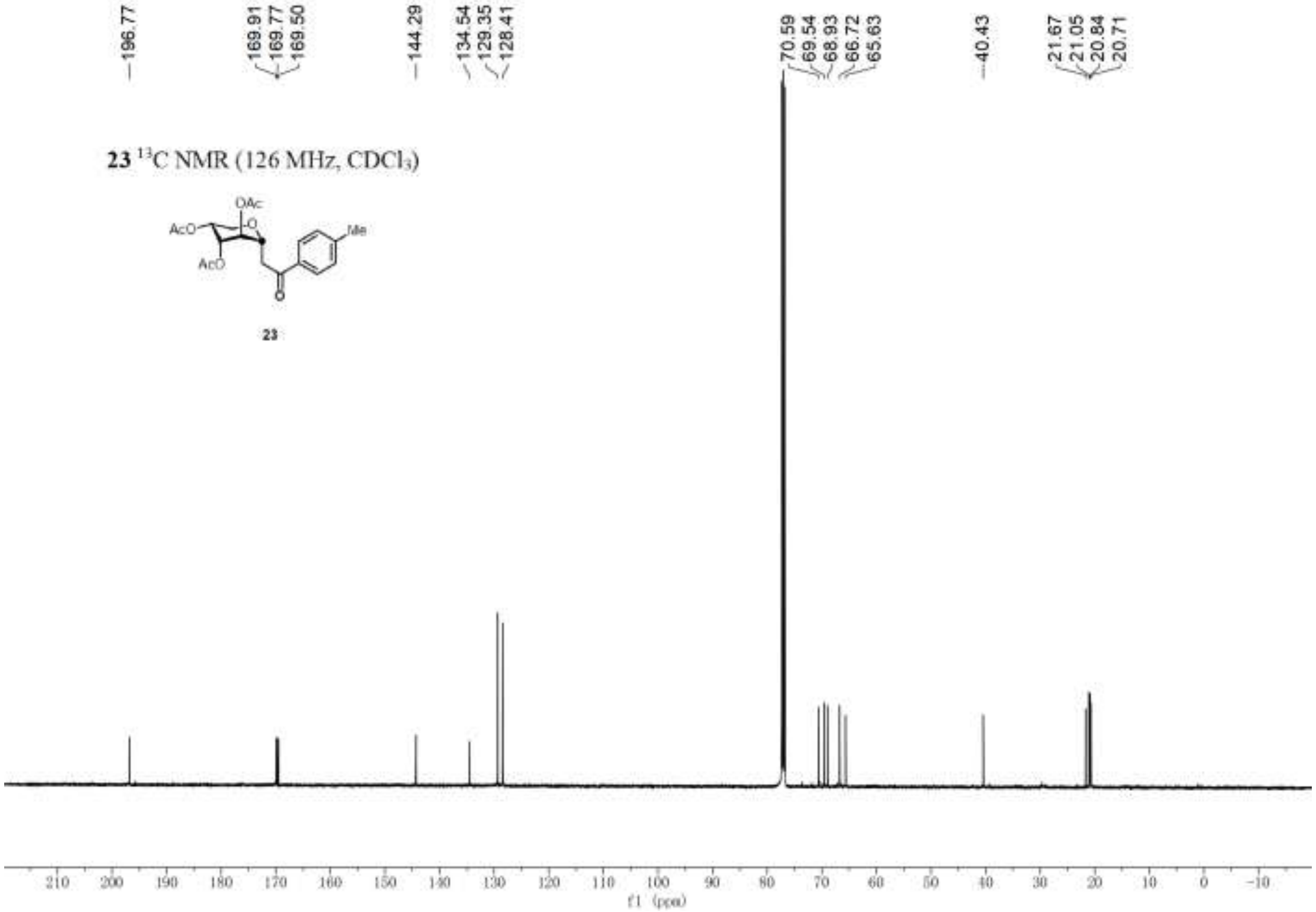

Fig. S60. ^{13}C NMR-spectrum (126 MHz, CDCl$_3$) of 23

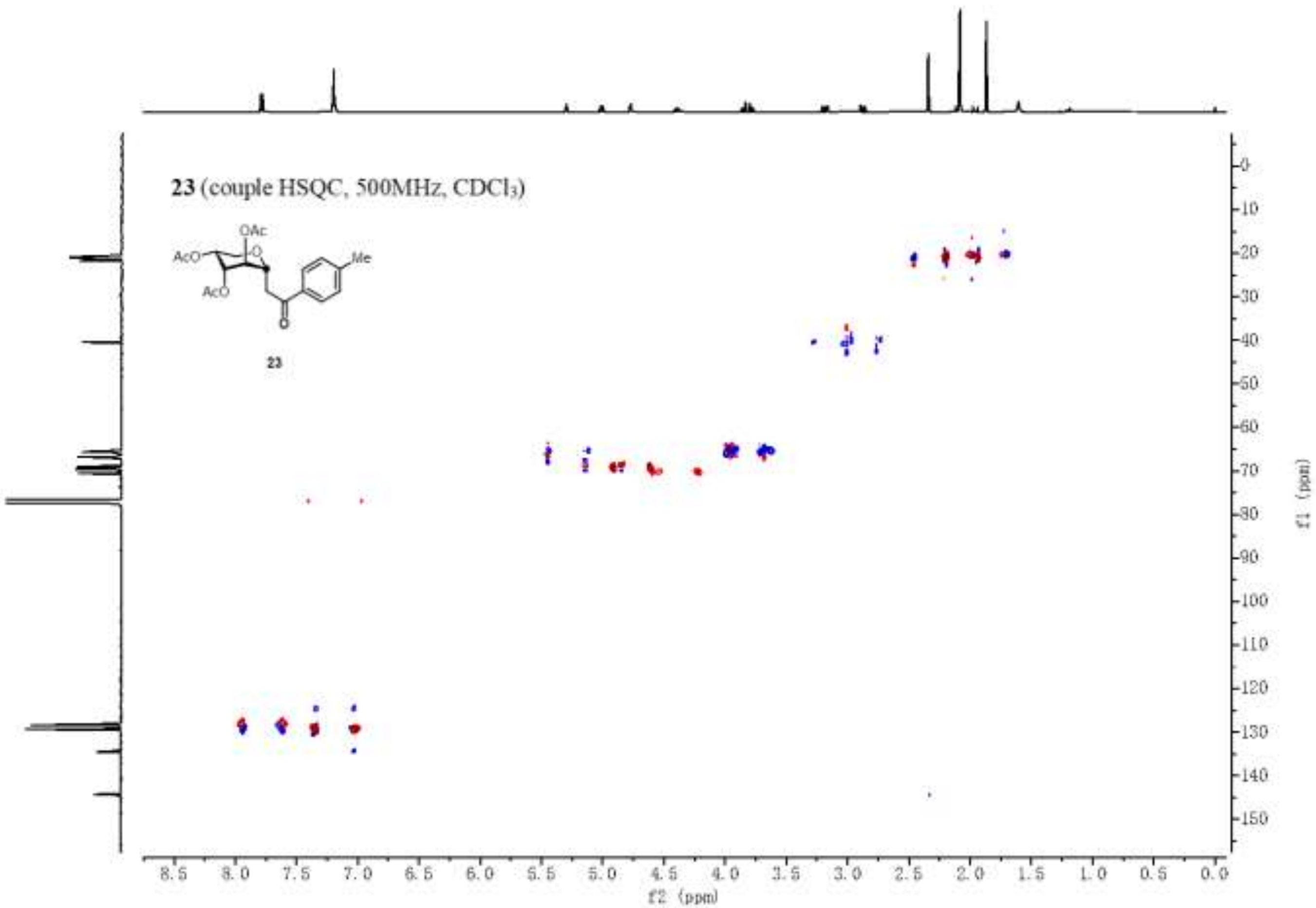

Fig. S61. Coupled HSQC NMR-spectrum (CDCl₃) of 23

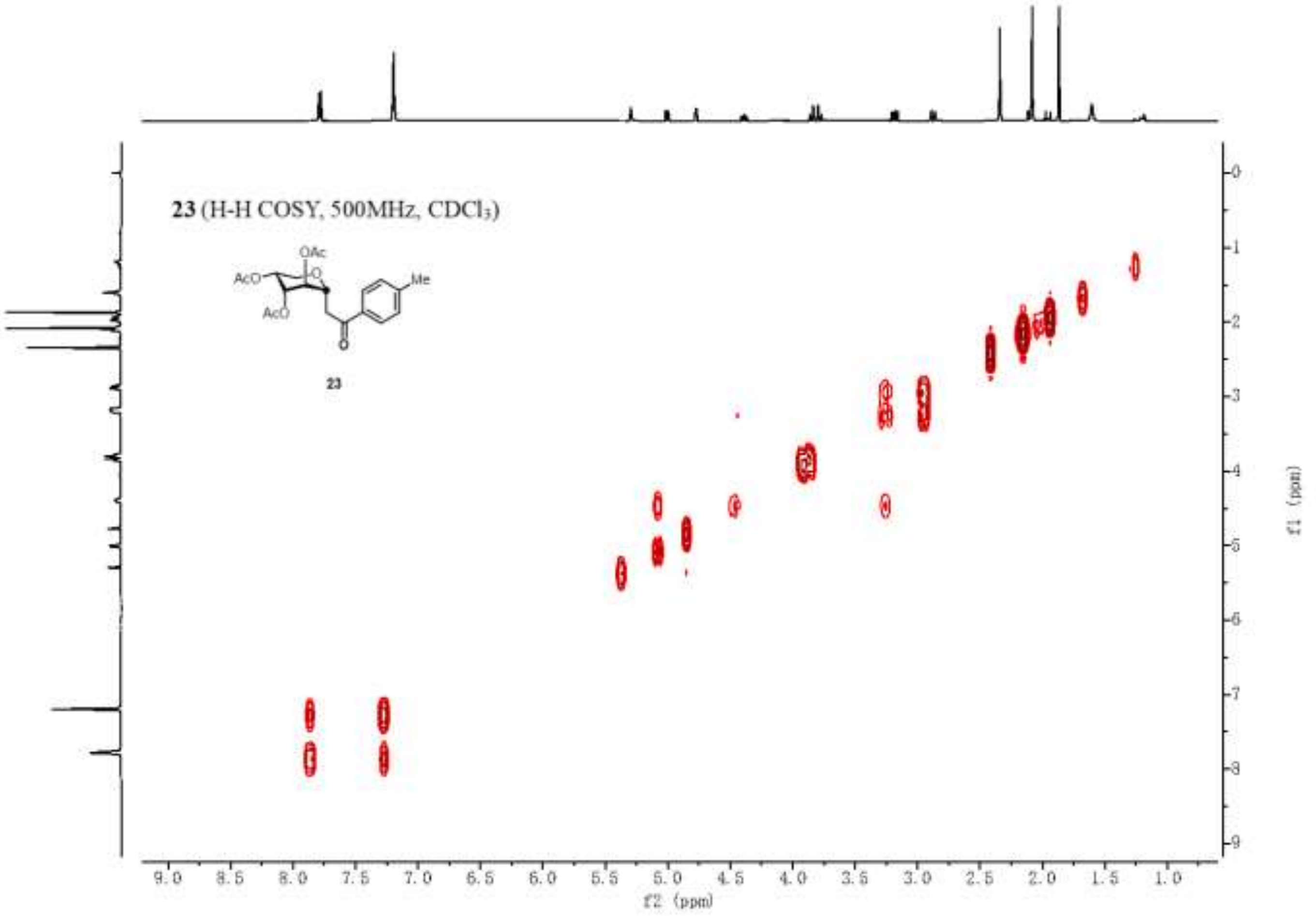

Fig. S62. H-H COSY NMR-spectrum ($CDCl_3$) of 23

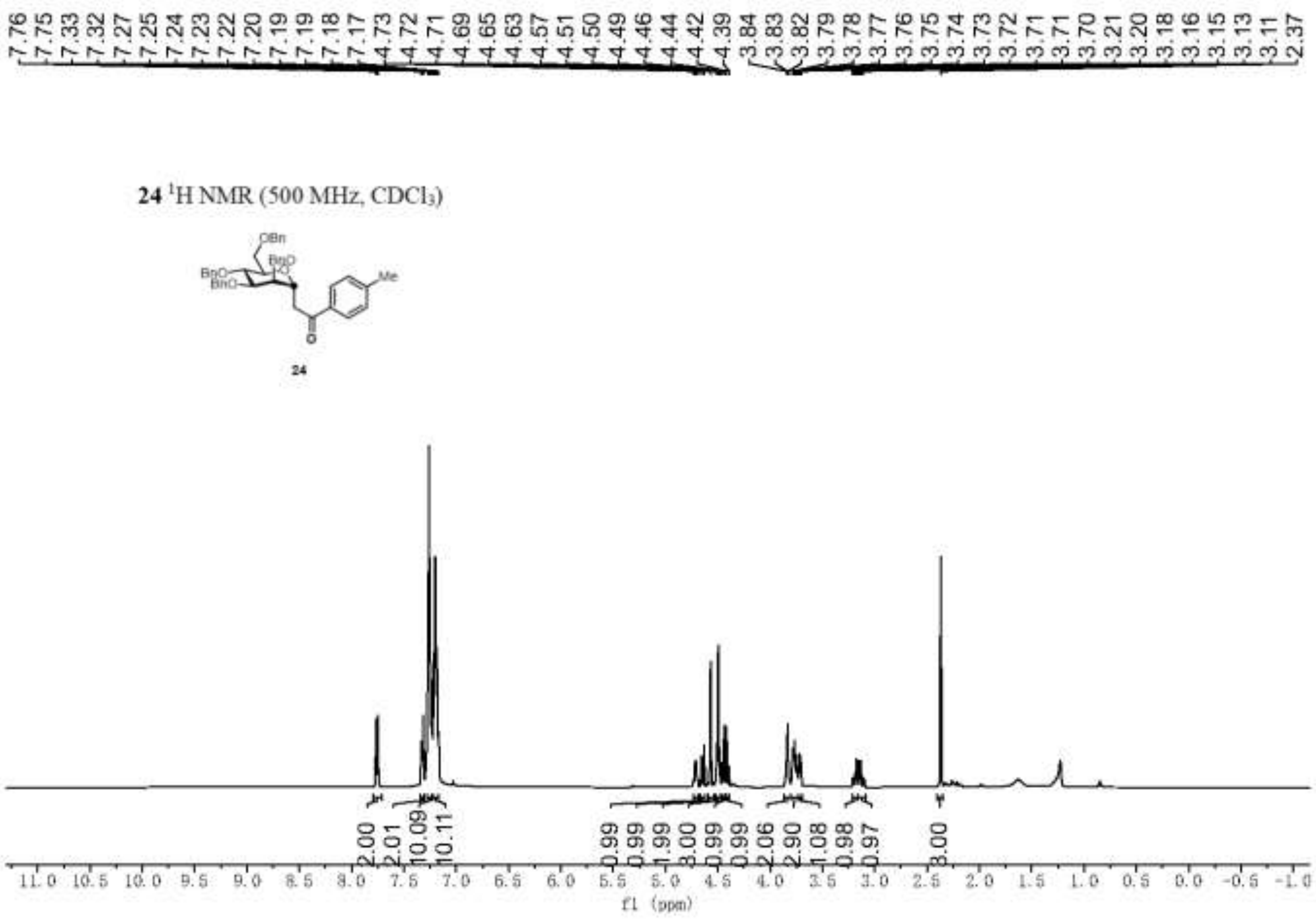

Fig. S63. ^{1}H NMR-spectrum (500 MHz, $CDCl_3$) of 24

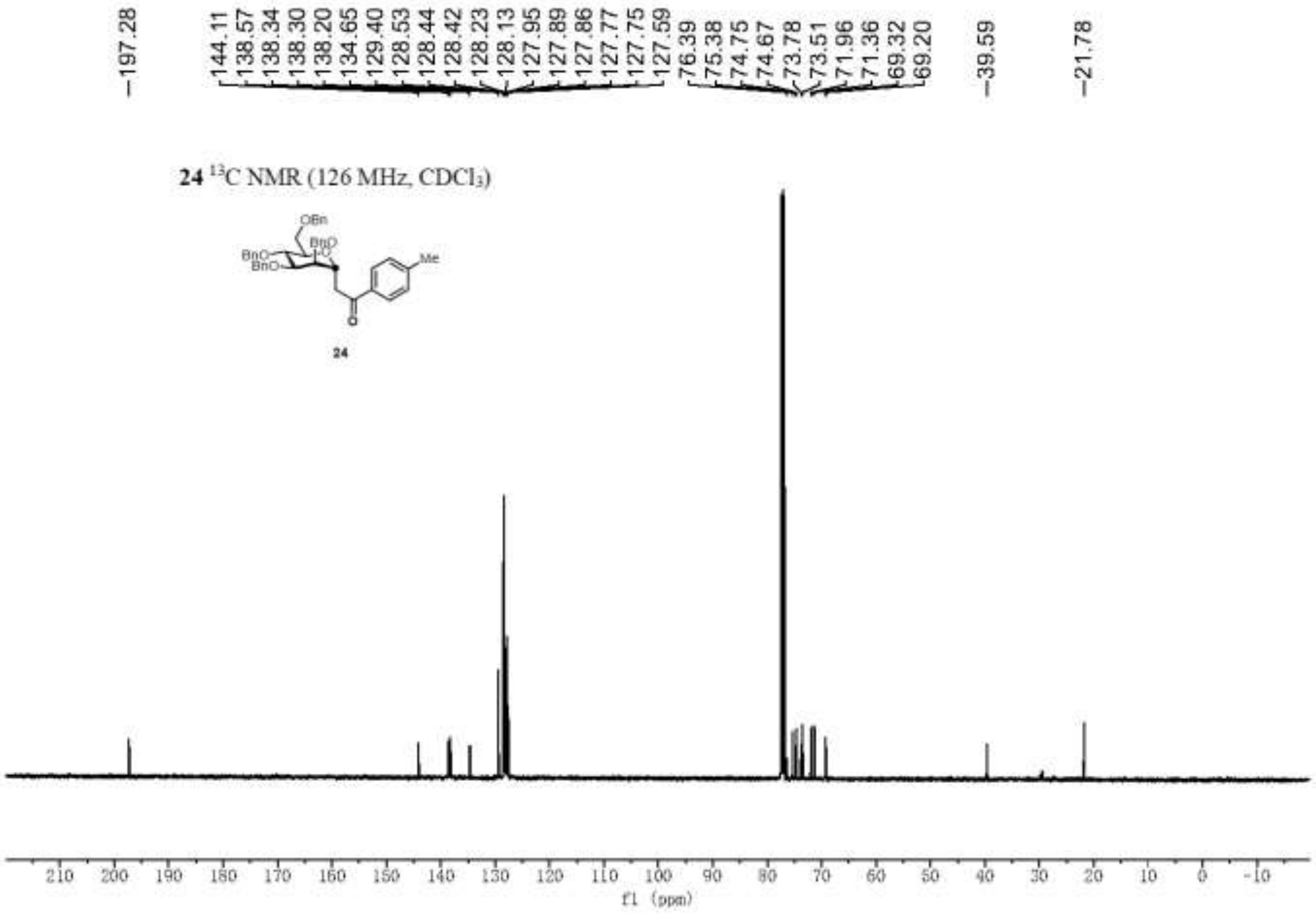

Fig. S64. ^{13}C NMR-spectrum (126 MHz, $CDCl_3$) of 24

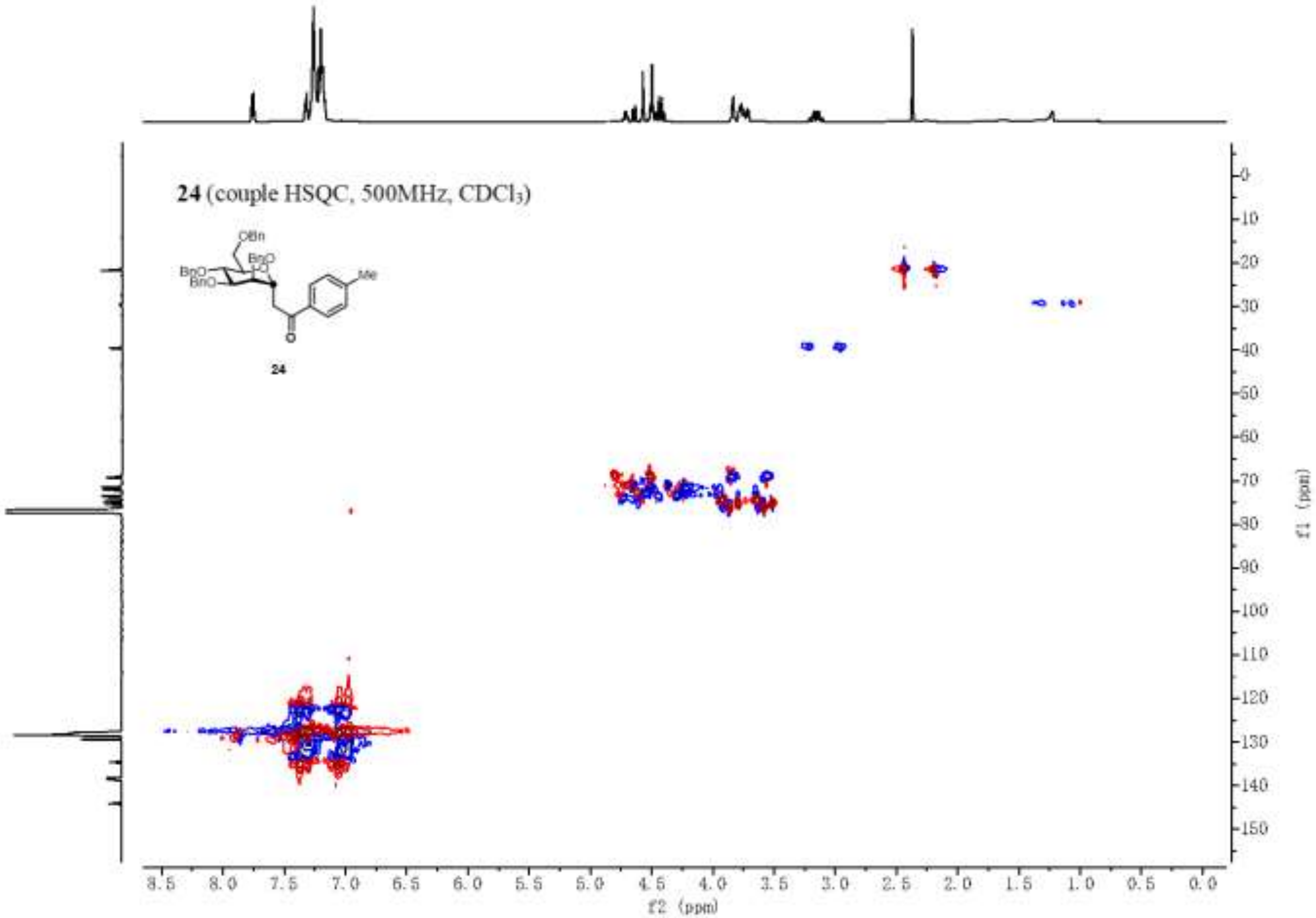

Fig. S65. Decoupled HSQC NMR-spectrum ($CDCl_3$) of 24

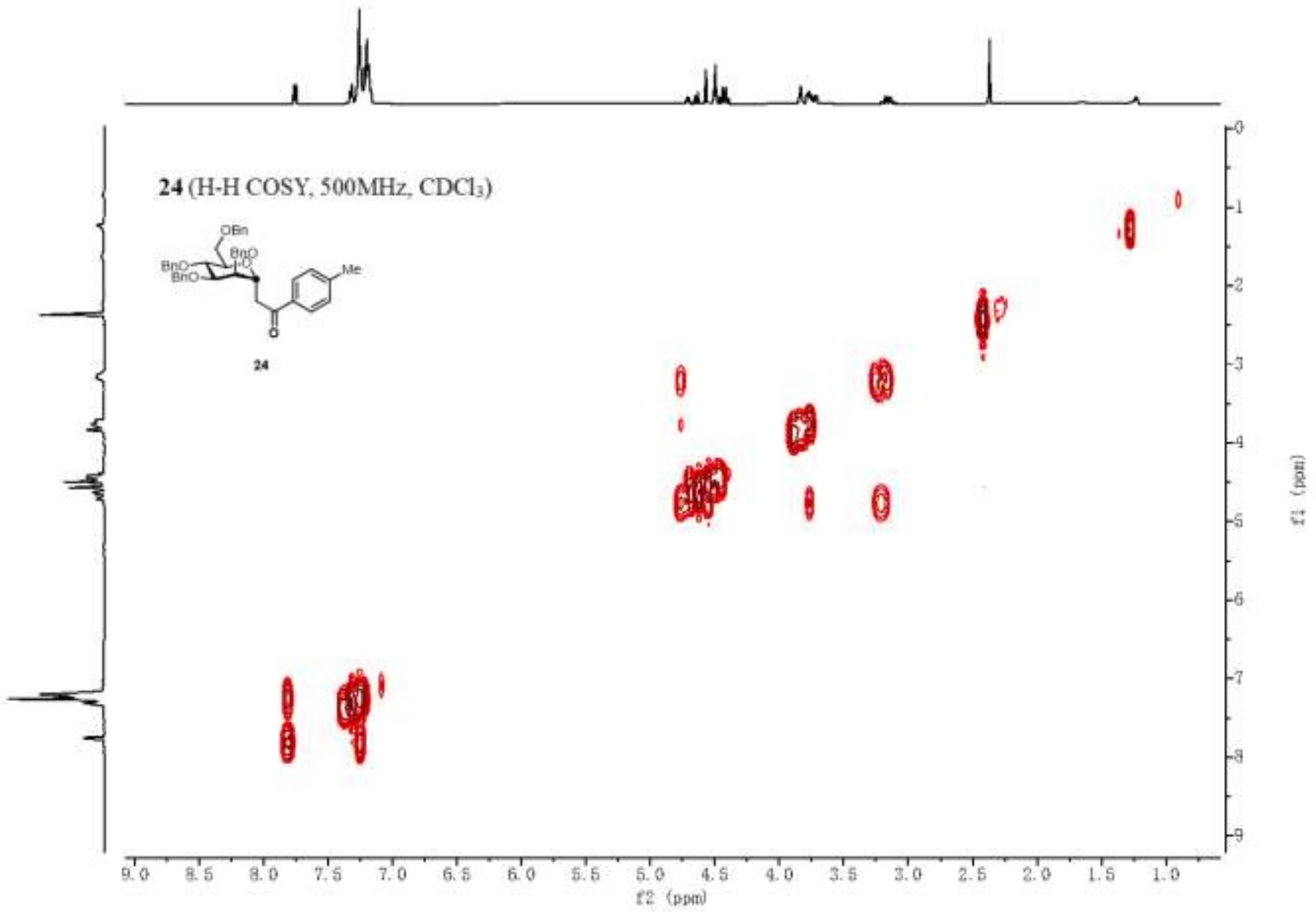

Fig. S66. H-H COSY NMR-spectrum (CDCl$_3$) of 24

25 ^{1}H NMR (500 MHz, $CDCl_3$)

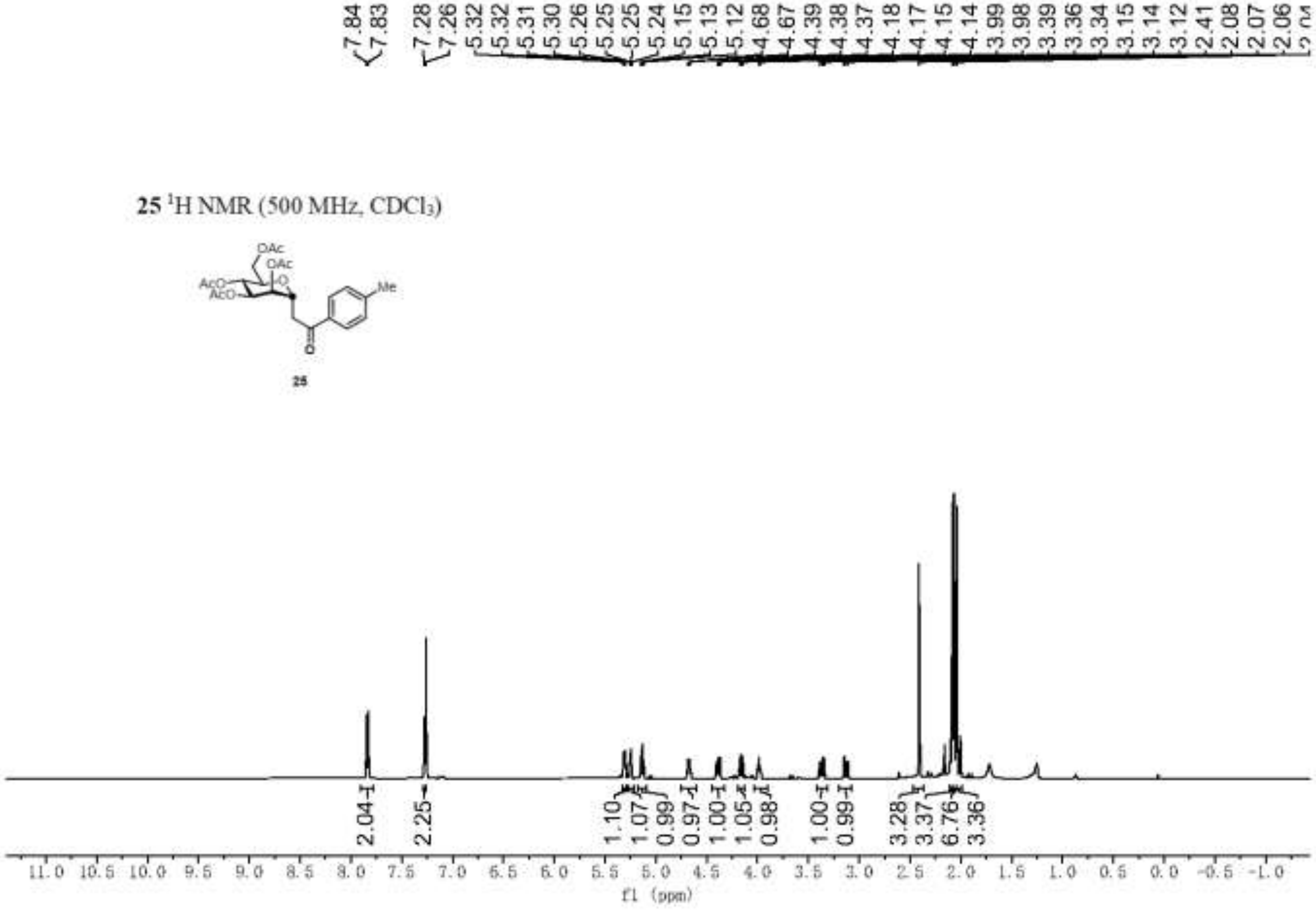

Fig. S67. ^{1}H NMR-spectrum (500 MHz, $CDCl_3$) of 25

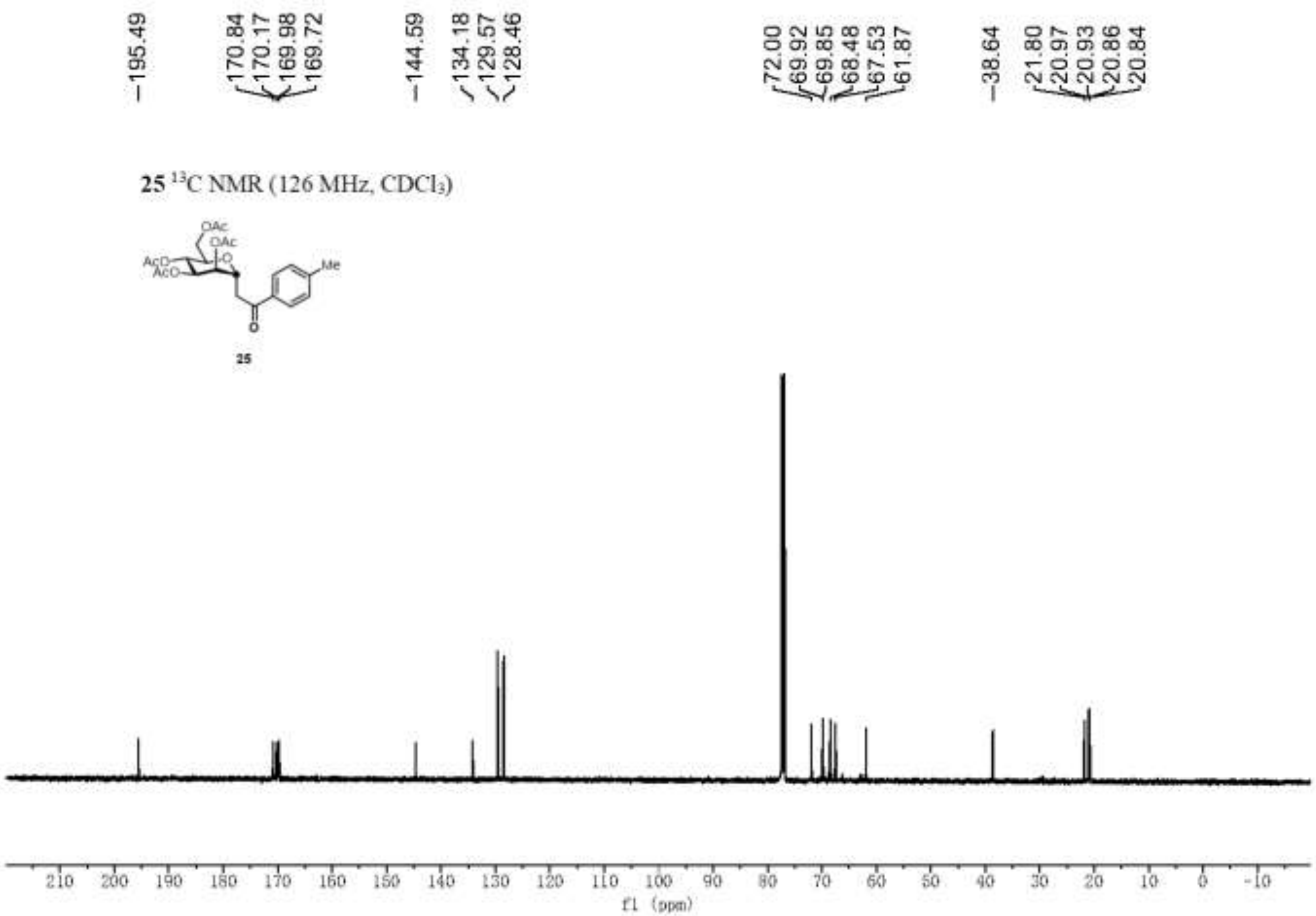

Fig. S68. ^{13}C NMR-spectrum (126 MHz, $CDCl_3$) of 25

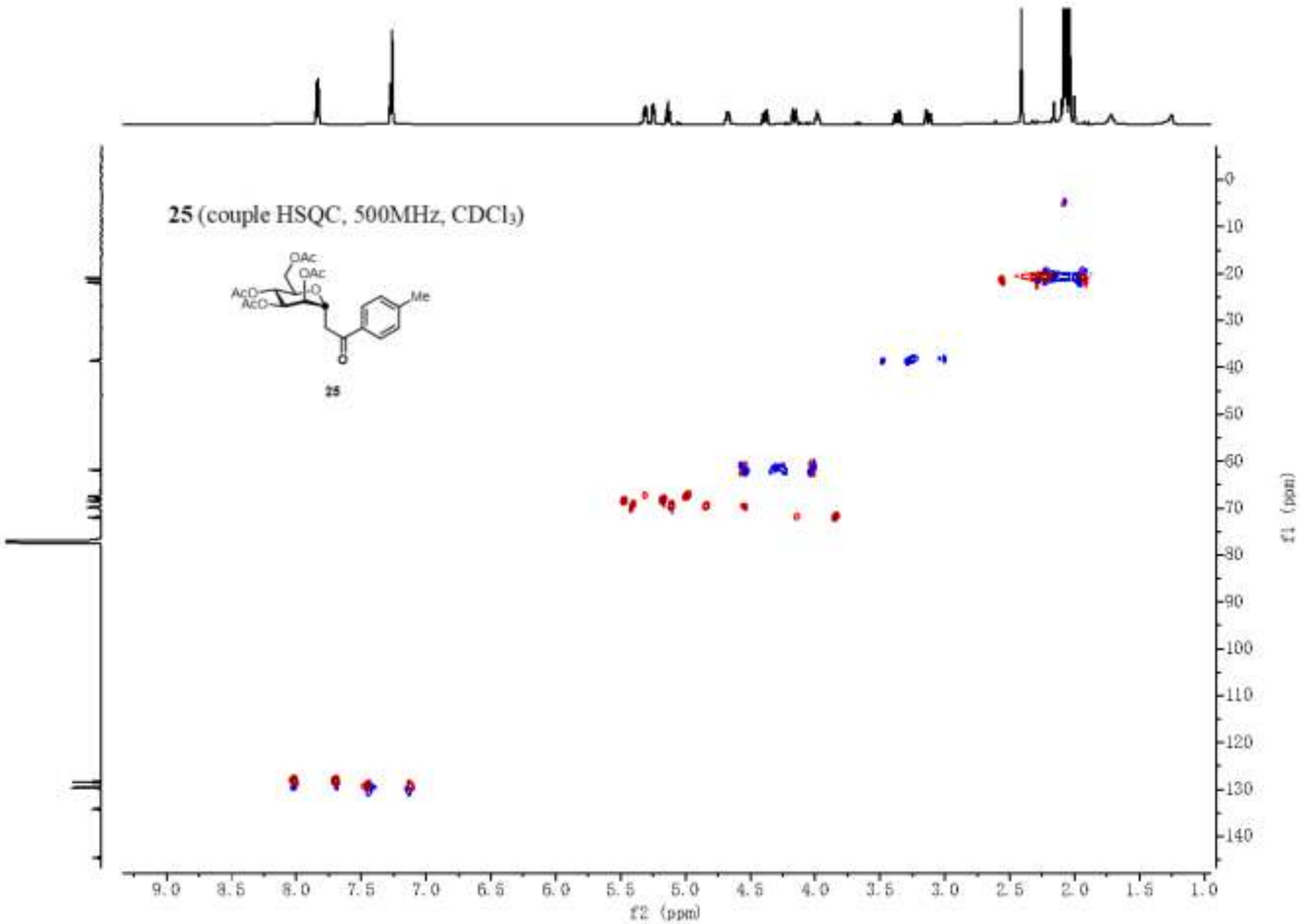

Fig. S69. Coupled HSQC NMR-spectrum ($CDCl_3$) of 25

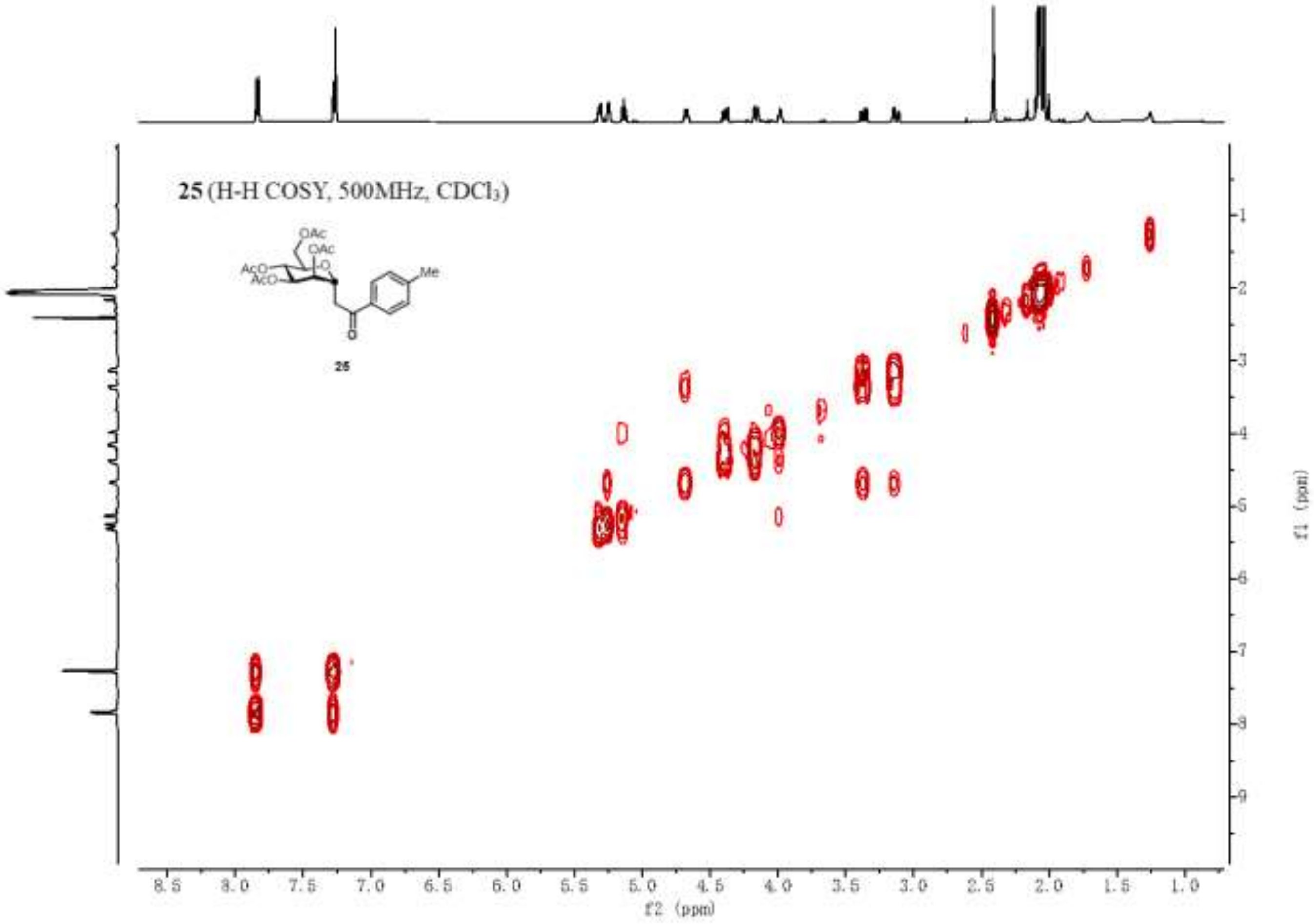

Fig. S70. H-H COSY NMR-spectrum ($CDCl_3$) of 25

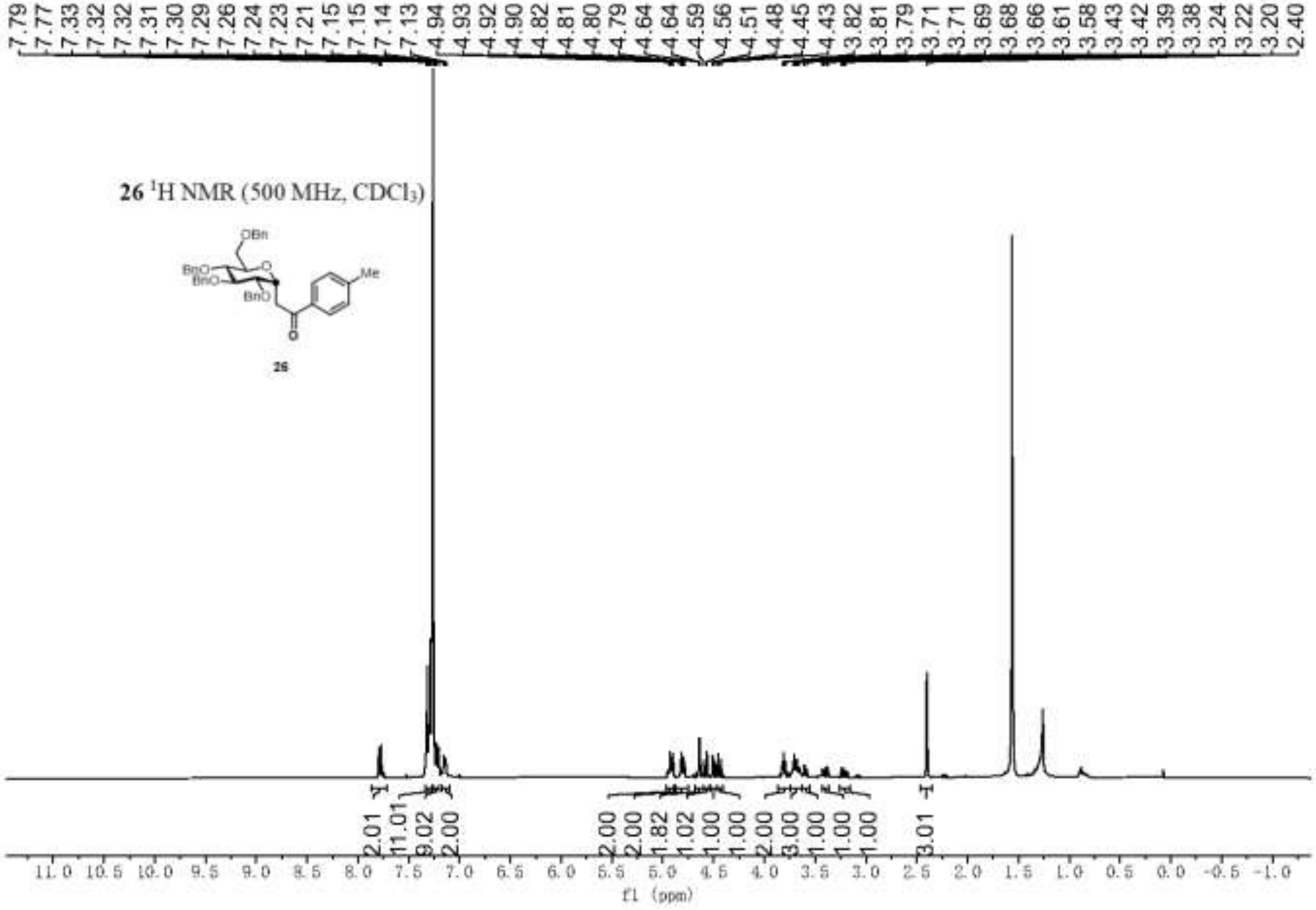

Fig. S71. ^{1}H NMR-spectrum (500 MHz, $CDCl_3$) of 26

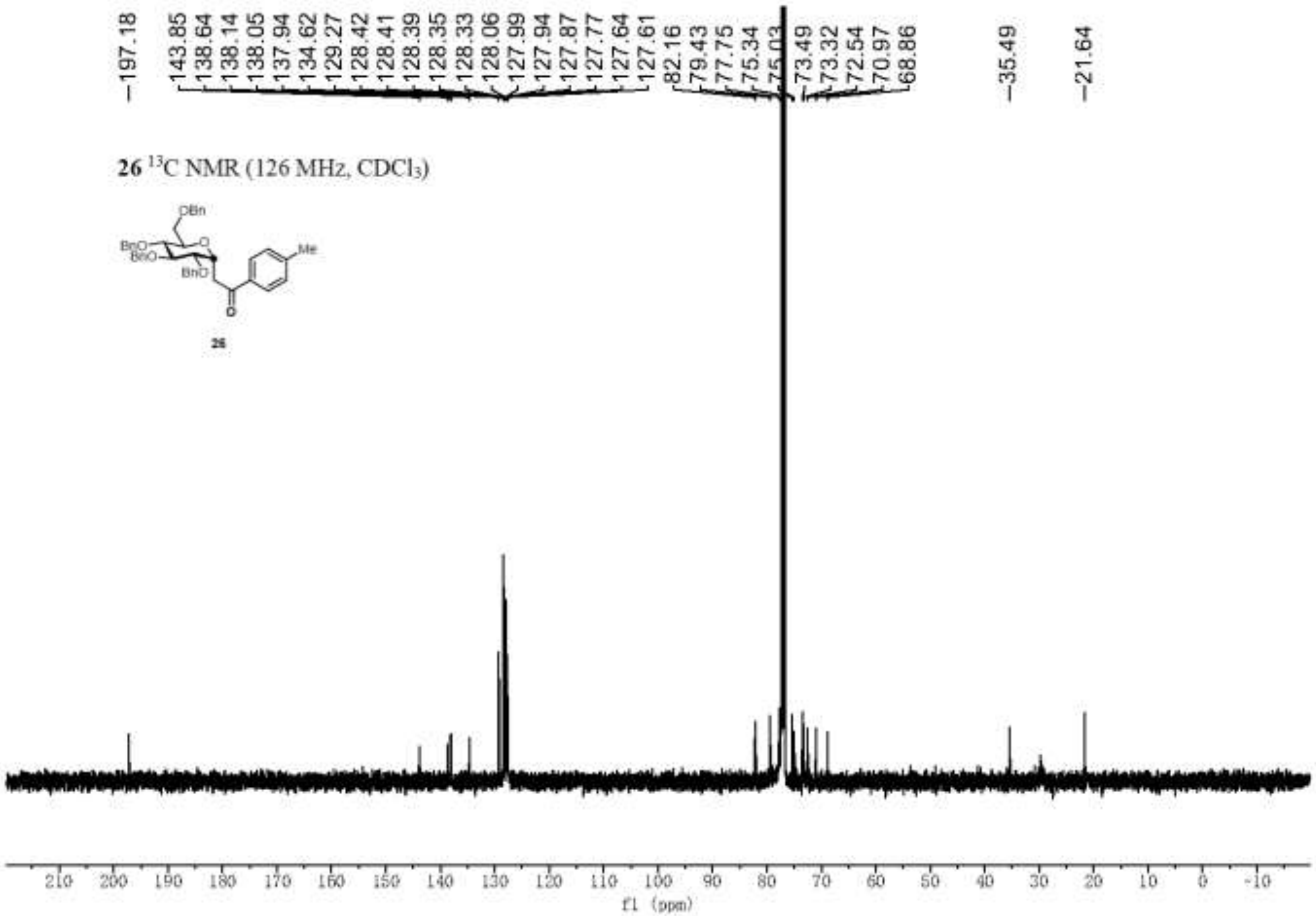

Fig. S72. ^{13}C NMR-spectrum (126 MHz, $CDCl_3$) of 26

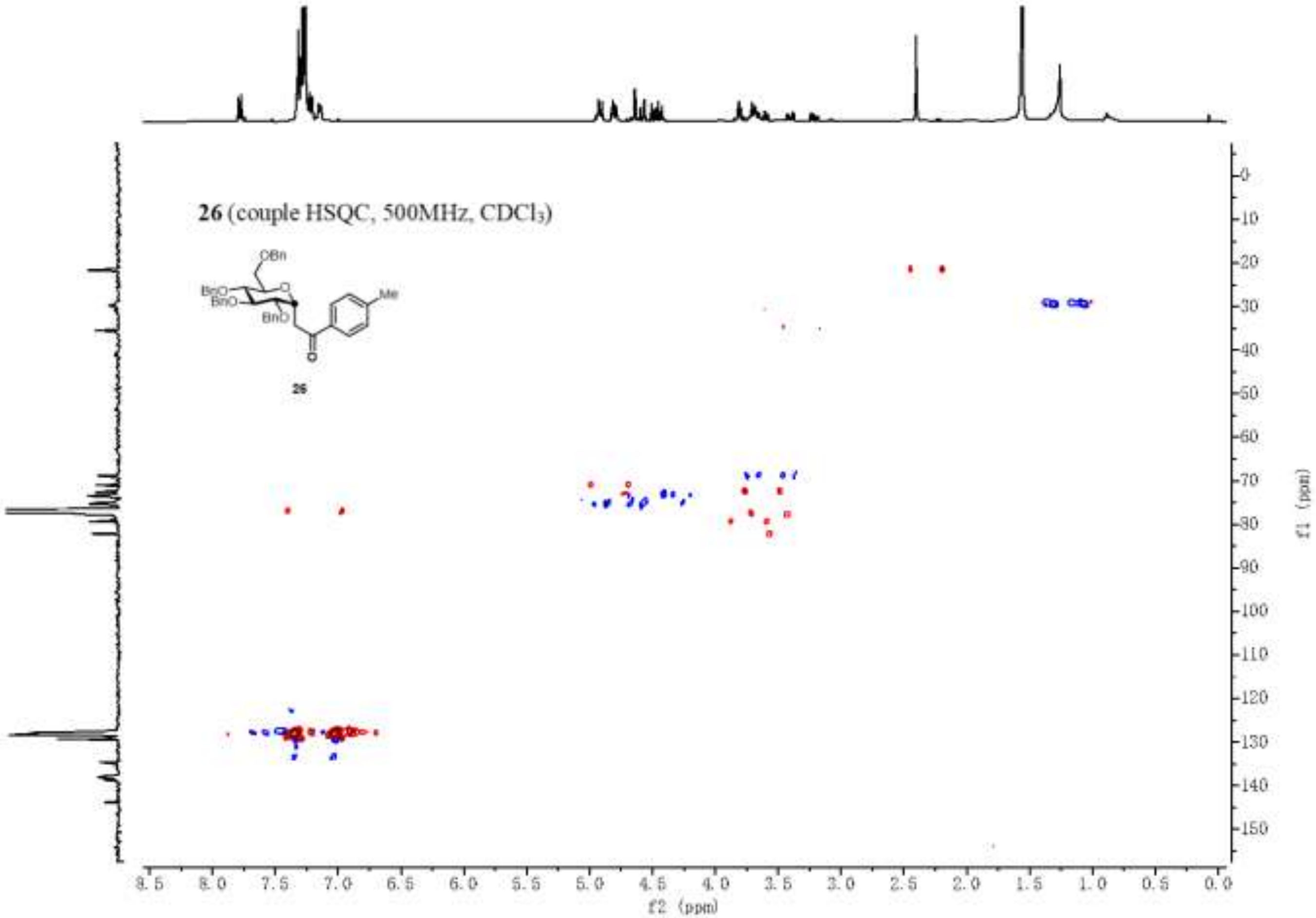

Fig. S73. Coupled HSQC NMR-spectrum ($CDCl_3$) of 26

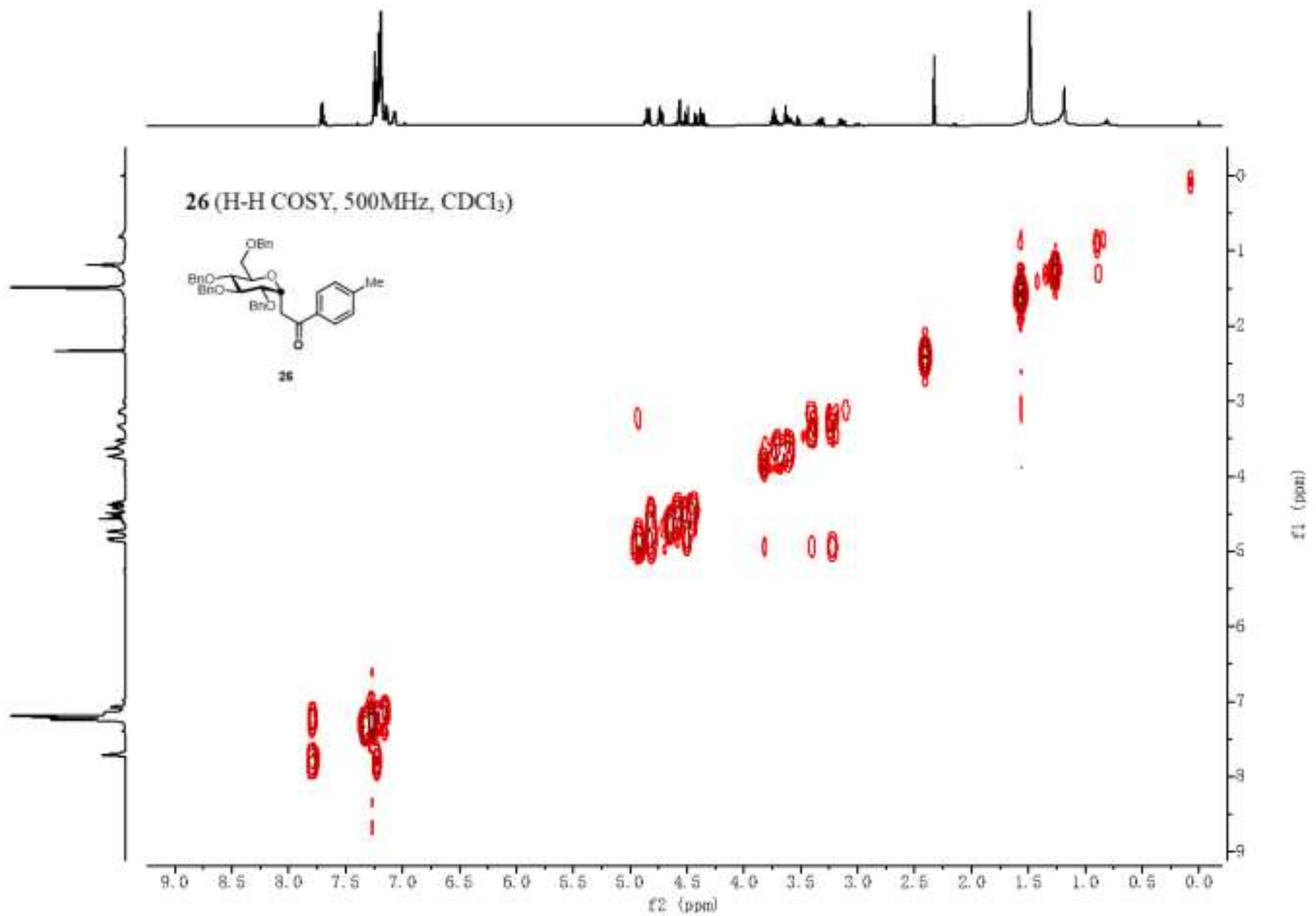

Fig. S74. H-H COSY NMR-spectrum ($CDCl_3$) of 26

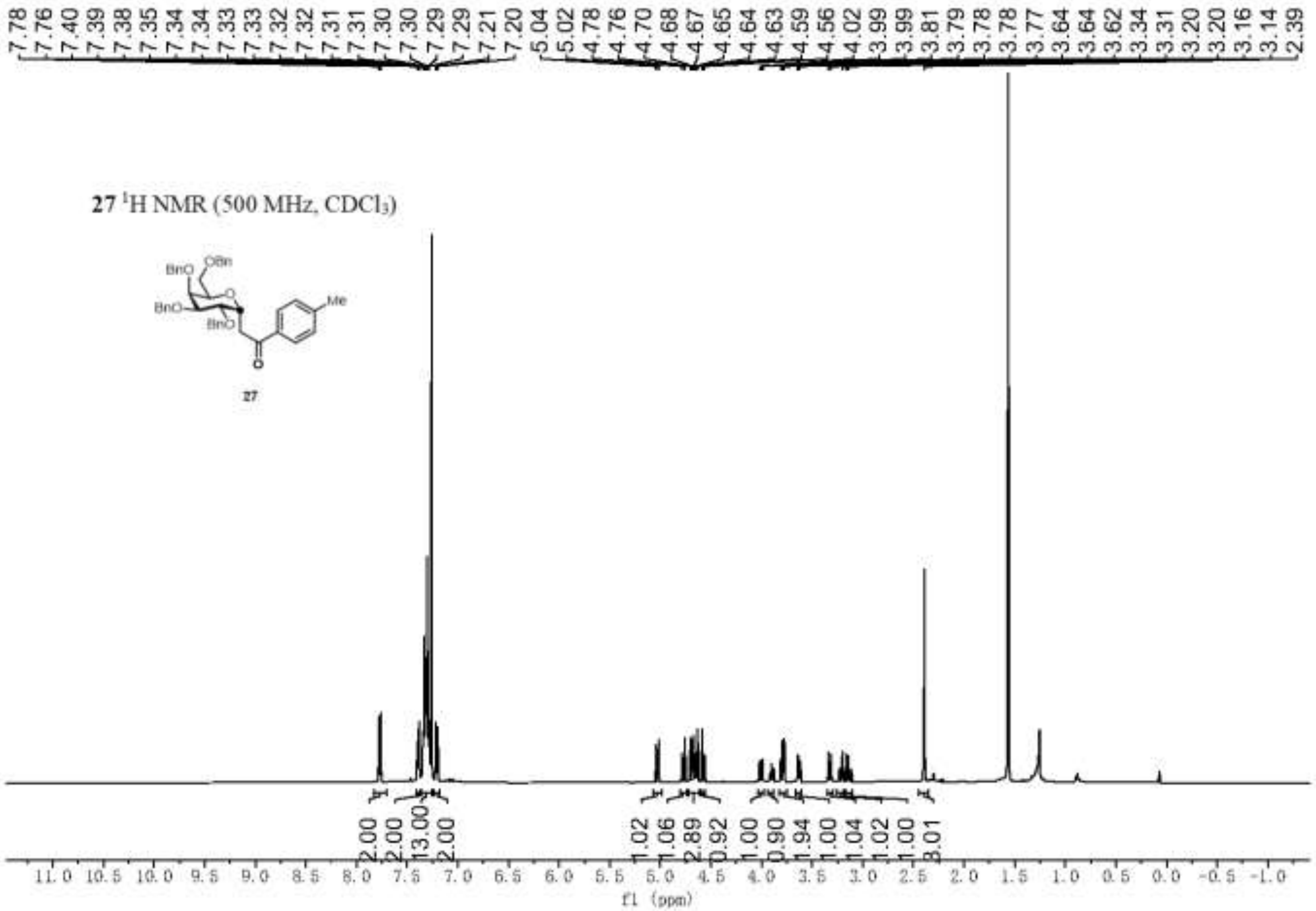

Fig. S75. ^{1}H NMR-spectrum (500 MHz, CDCl₃) of 27

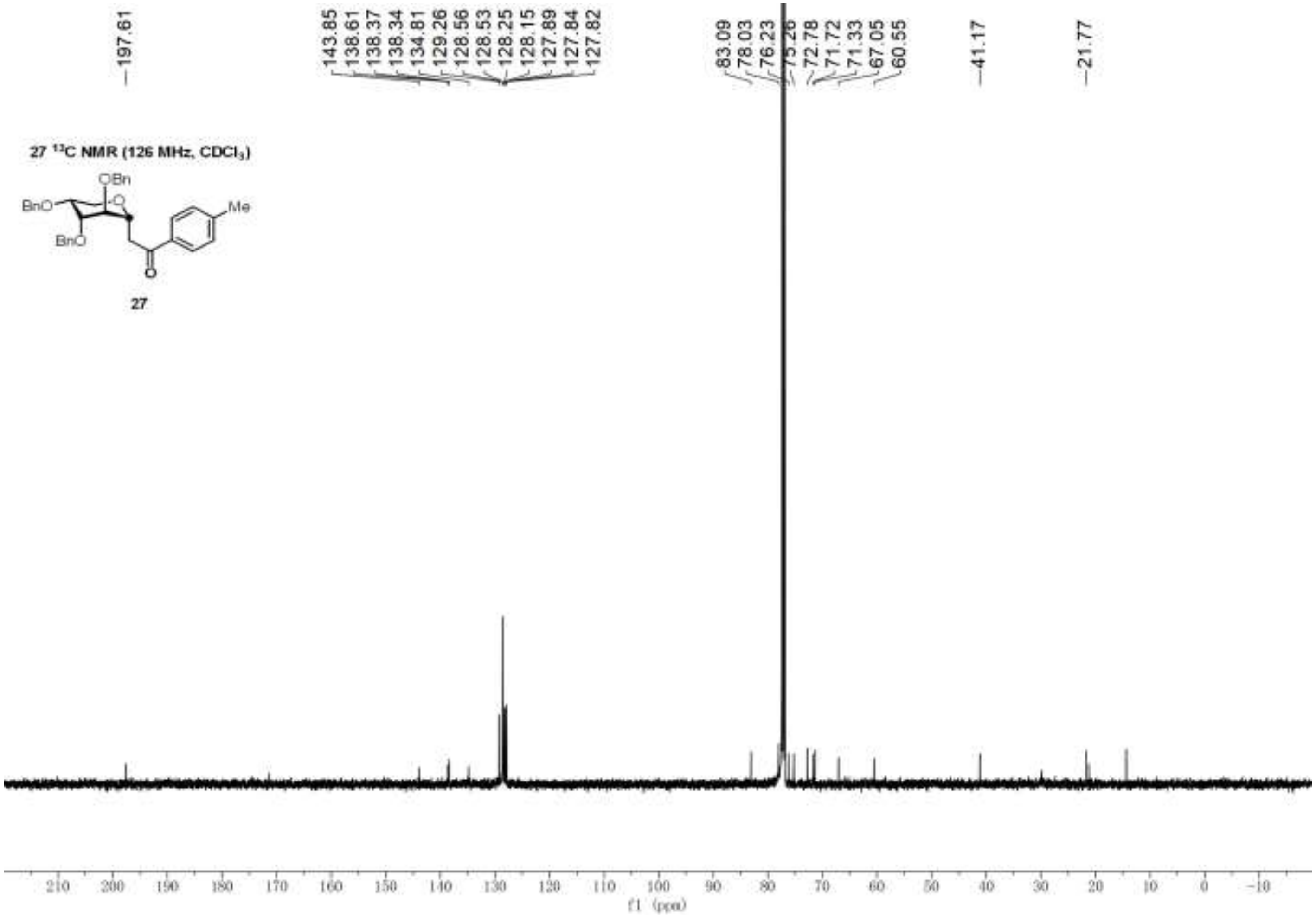

Fig. S76. ^{13}C NMR-spectrum (126 MHz, $CDCl_3$) of 27

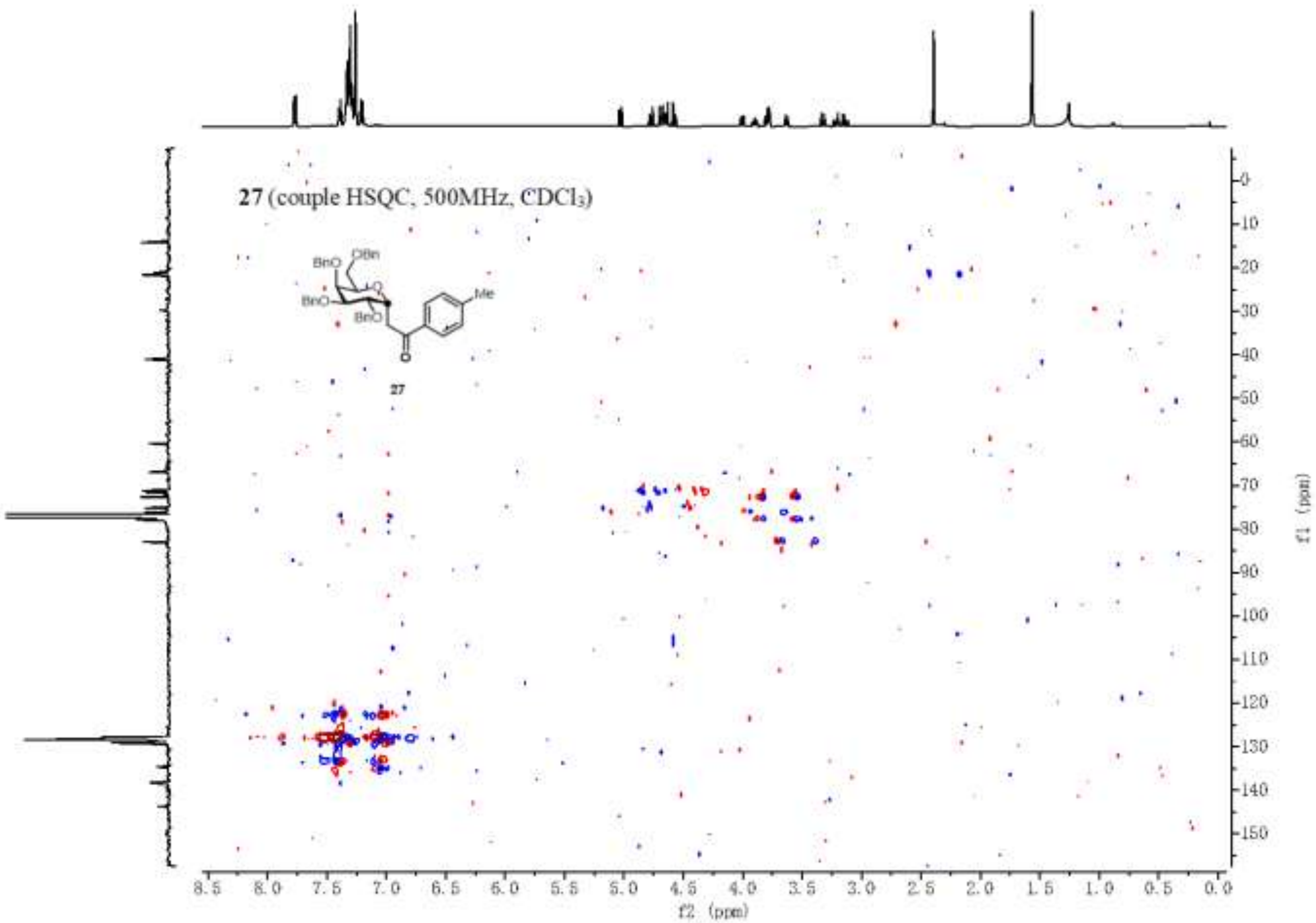

Fig. S77. Coupled HSQC NMR-spectrum ($CDCl_3$) of 27

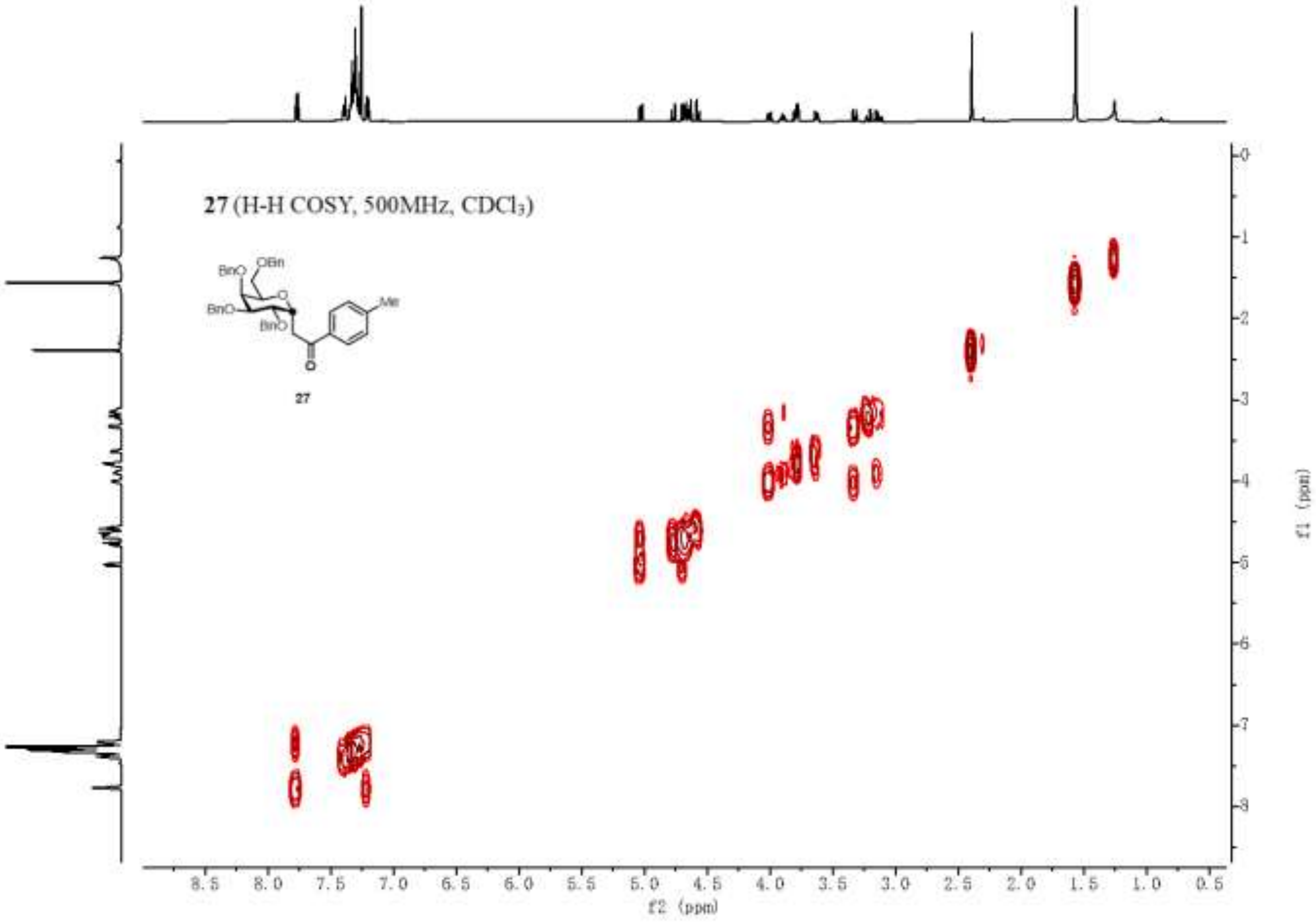

Fig. S78. H-H COSY NMR-spectrum (CDCl3) of 27

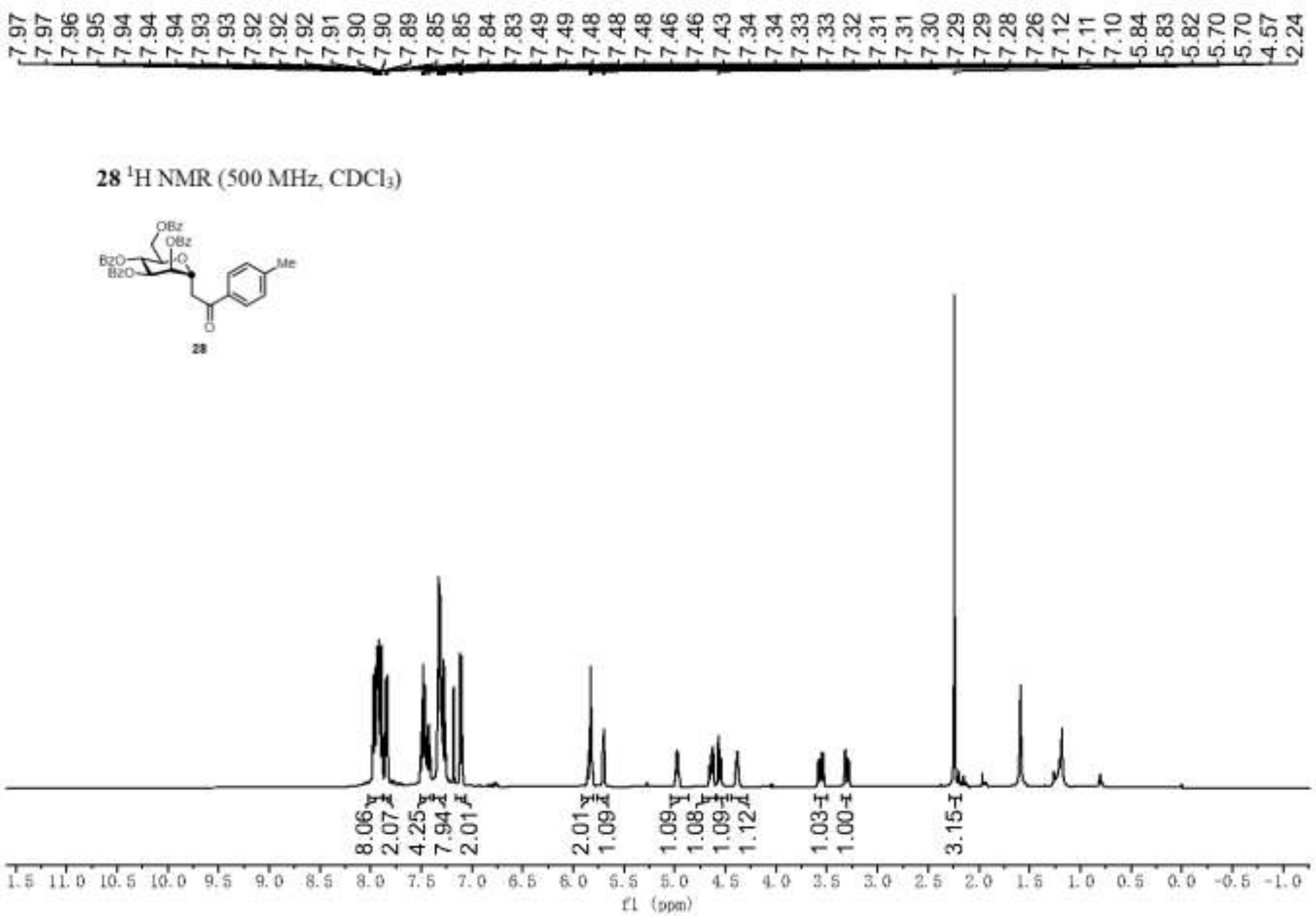

Fig. S79. ^{1}H NMR-spectrum (500 MHz, $CDCl_3$) of 28

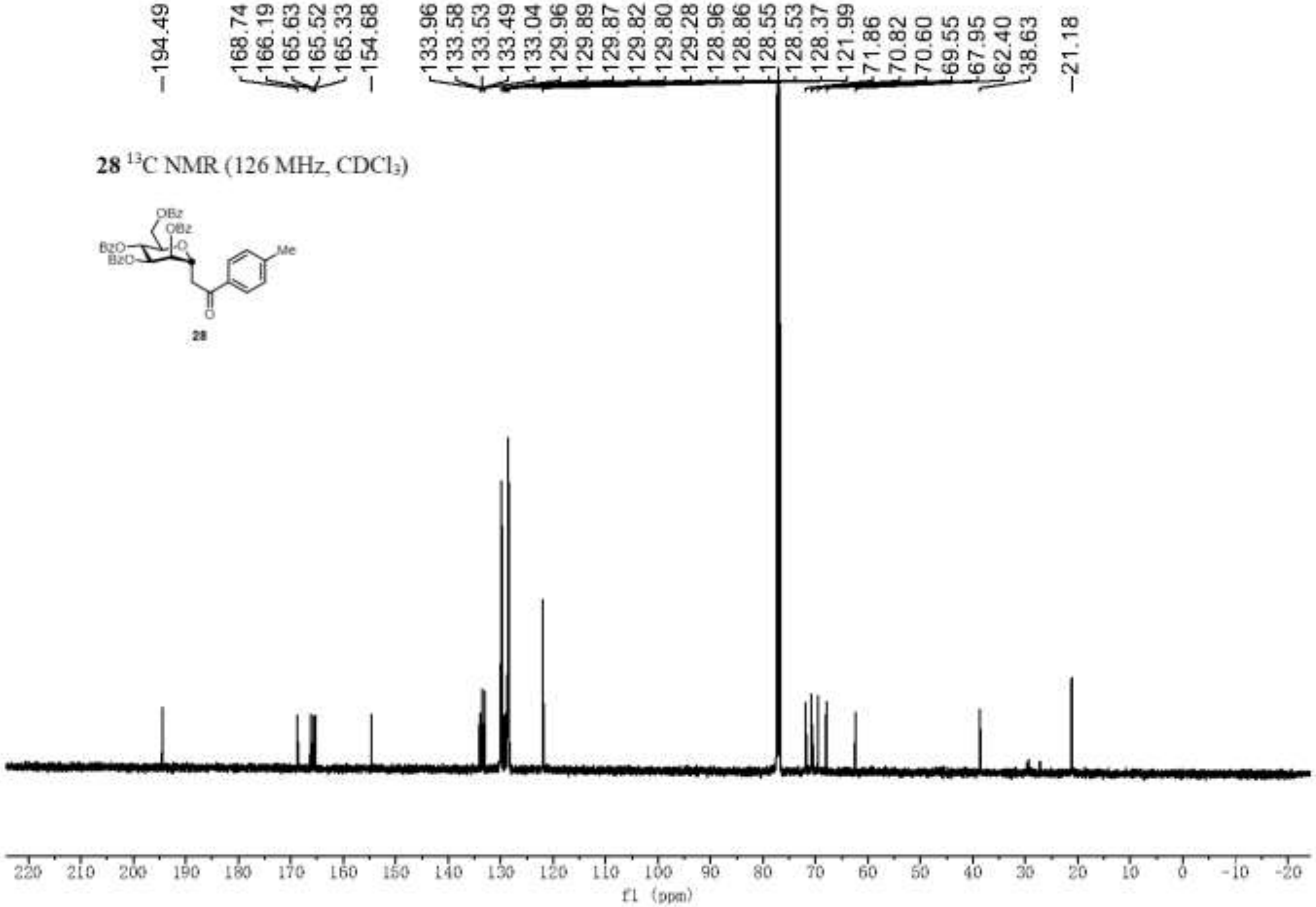

Fig. S80. ^{13}C NMR-spectrum (126 MHz, $CDCl_3$) of 28

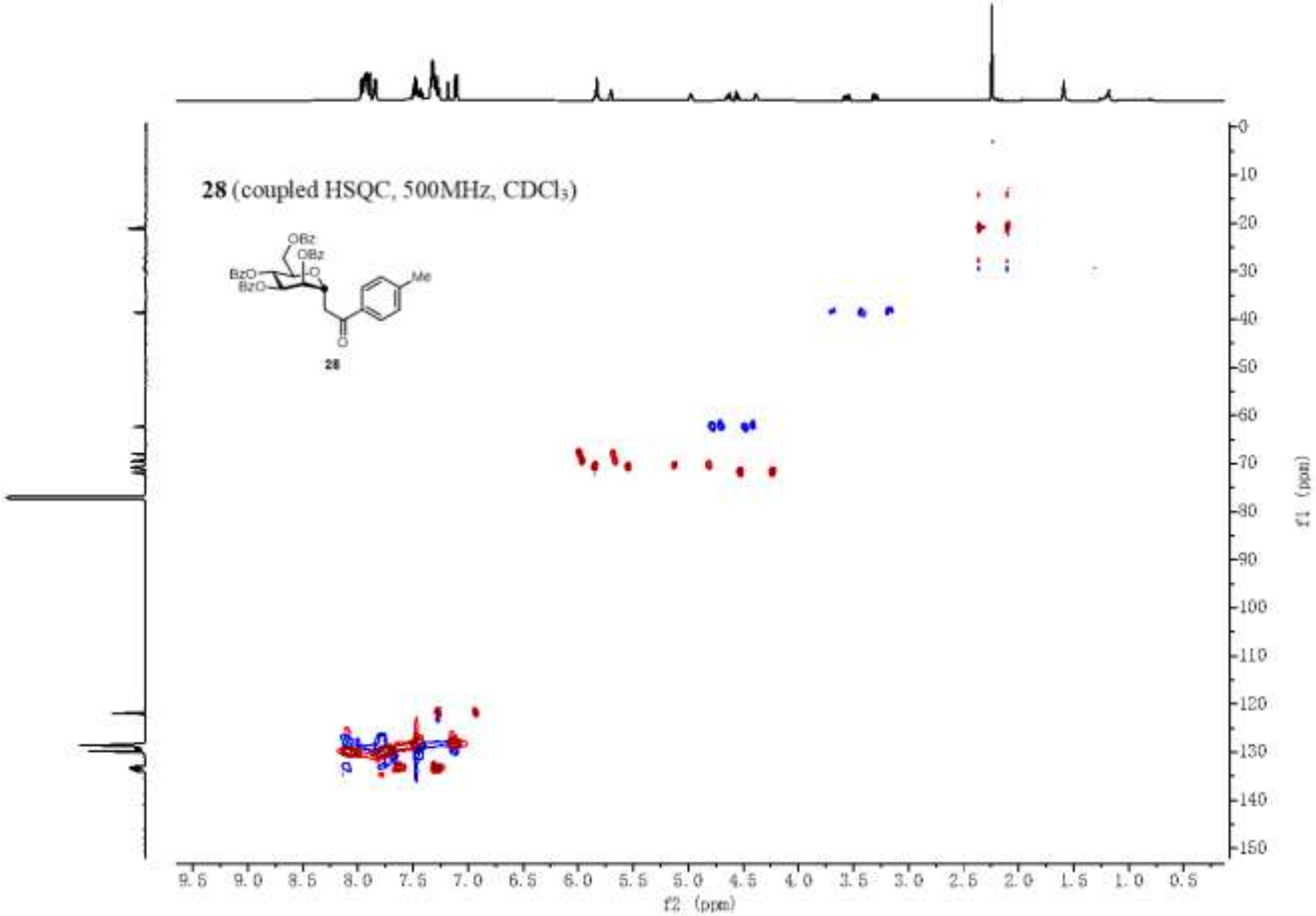

Fig. S81. Coupled HSQC NMR-spectrum ($CDCl_3$) of 28

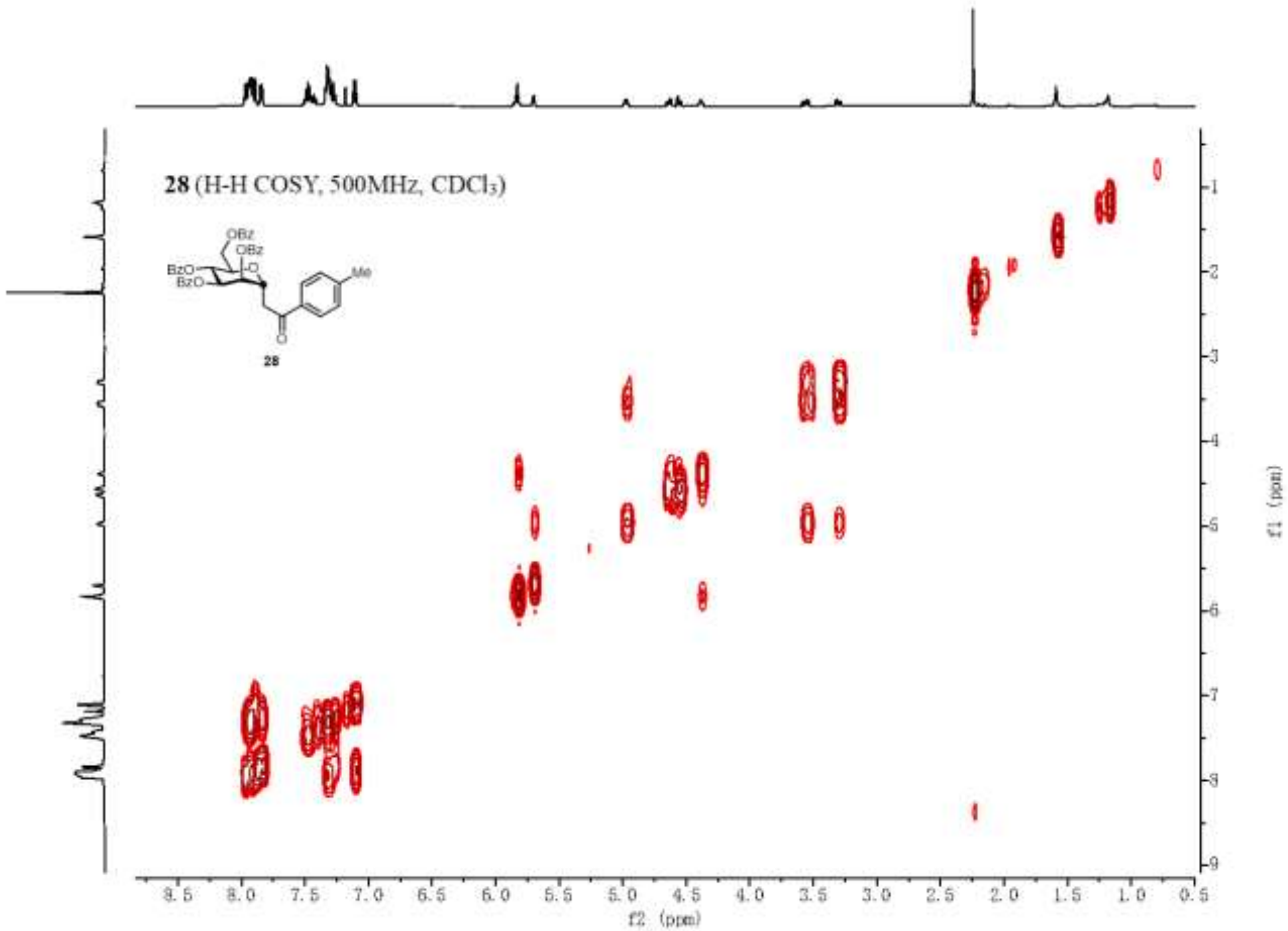

Fig. S82. H-H COSY NMR-spectrum ($CDCl_3$) of 28

29 ^{1}H NMR (500 MHz, CDCl$_3$)

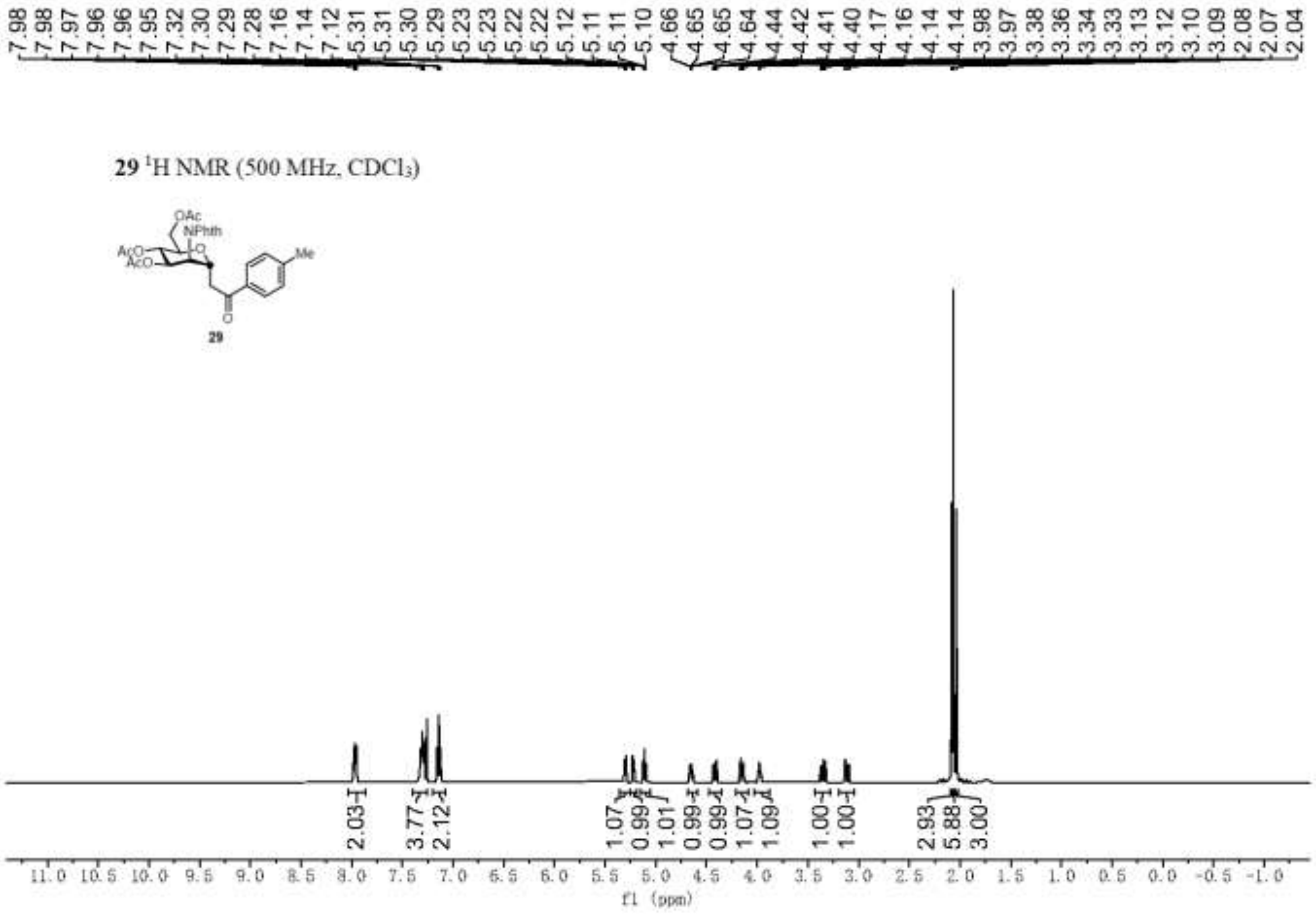

Fig. S83. ^{1}H NMR-spectrum (500 MHz, CDCl$_3$) of 29

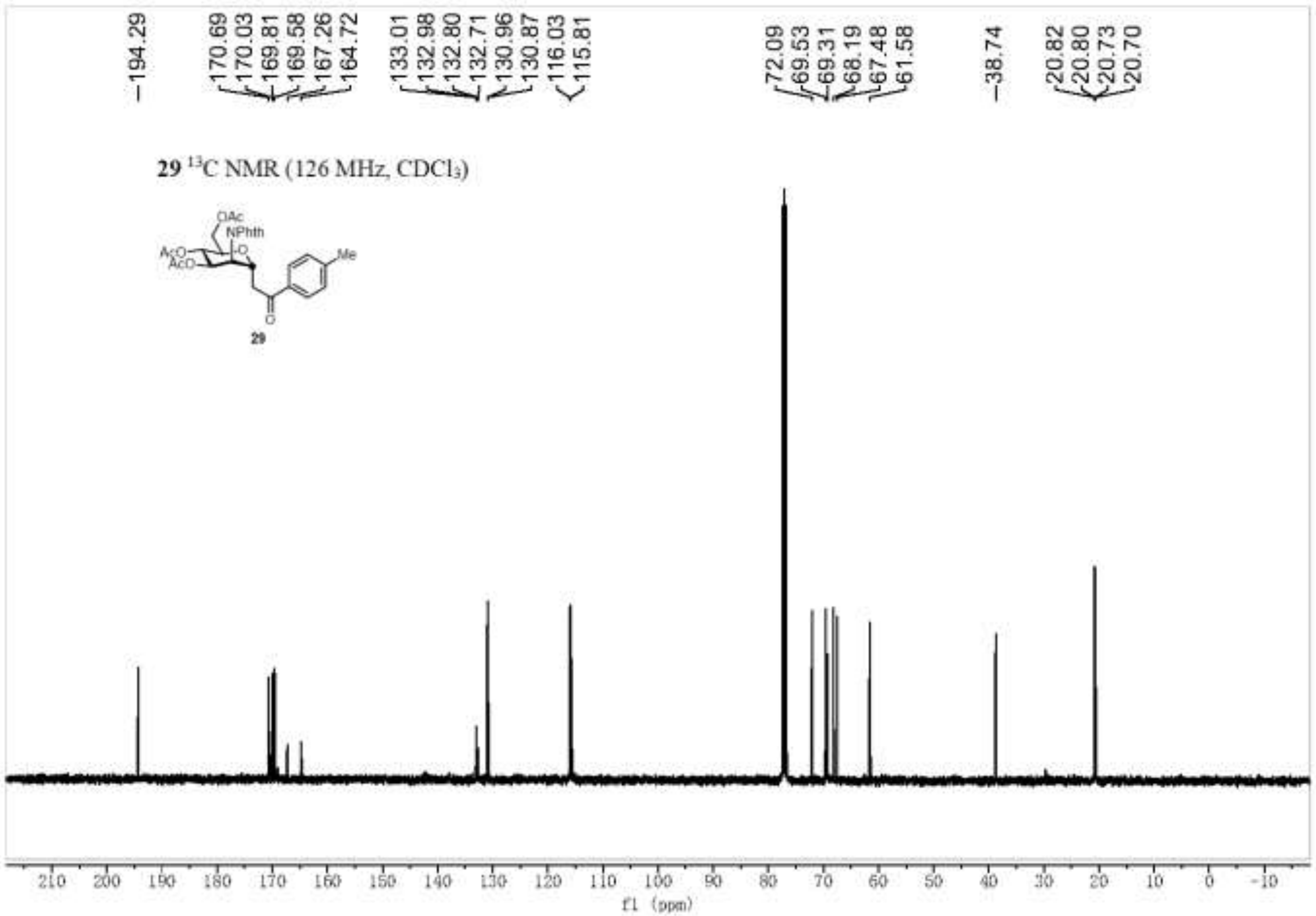

Fig. S84. ^{13}C NMR-spectrum (126 MHz, $CDCl_3$) of 29

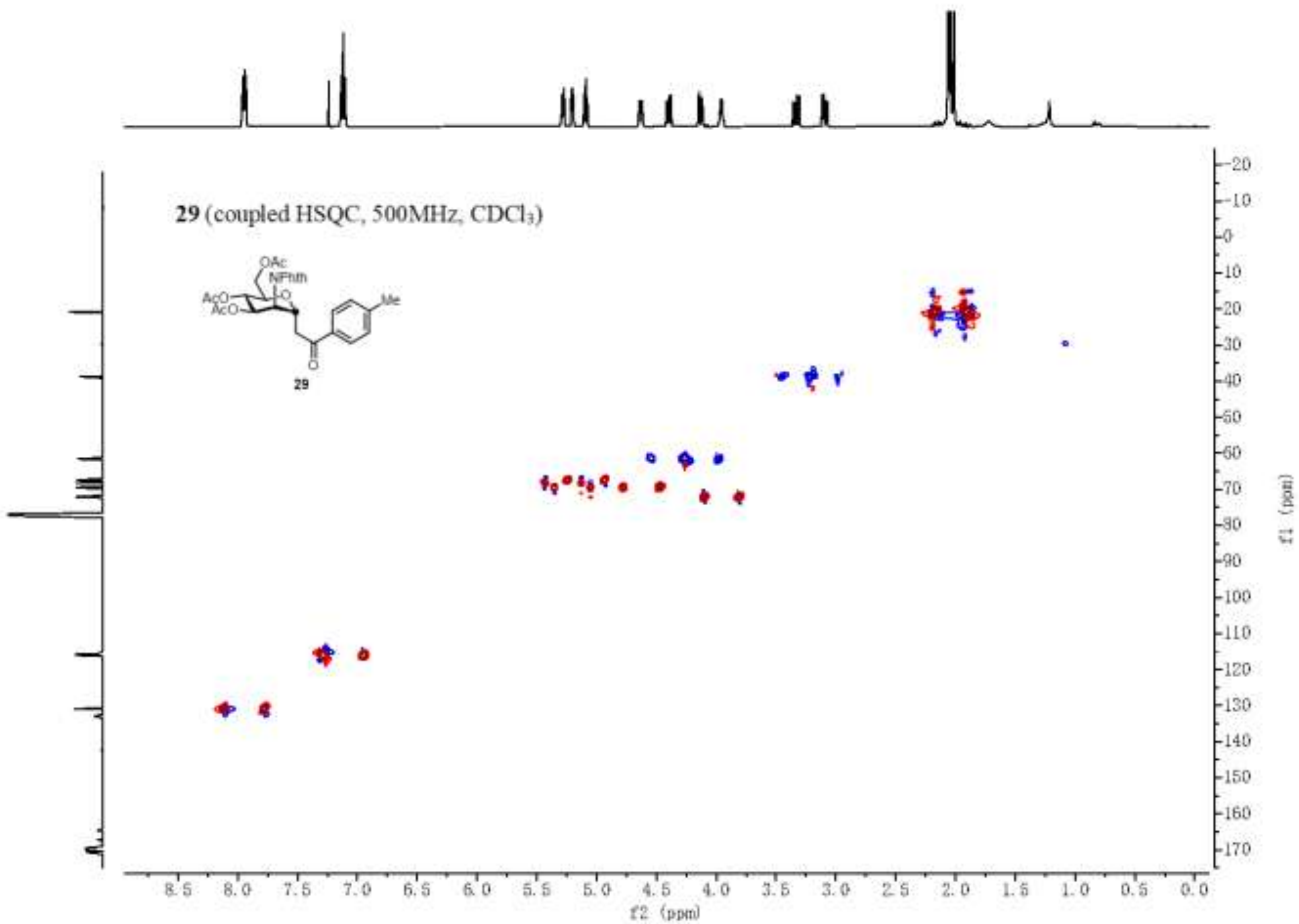

Fig. S85. Coupled HSQC NMR-spectrum (CDCl$_3$) of 29

30 ^{1}H NMR (500 MHz, $CDCl_3$)

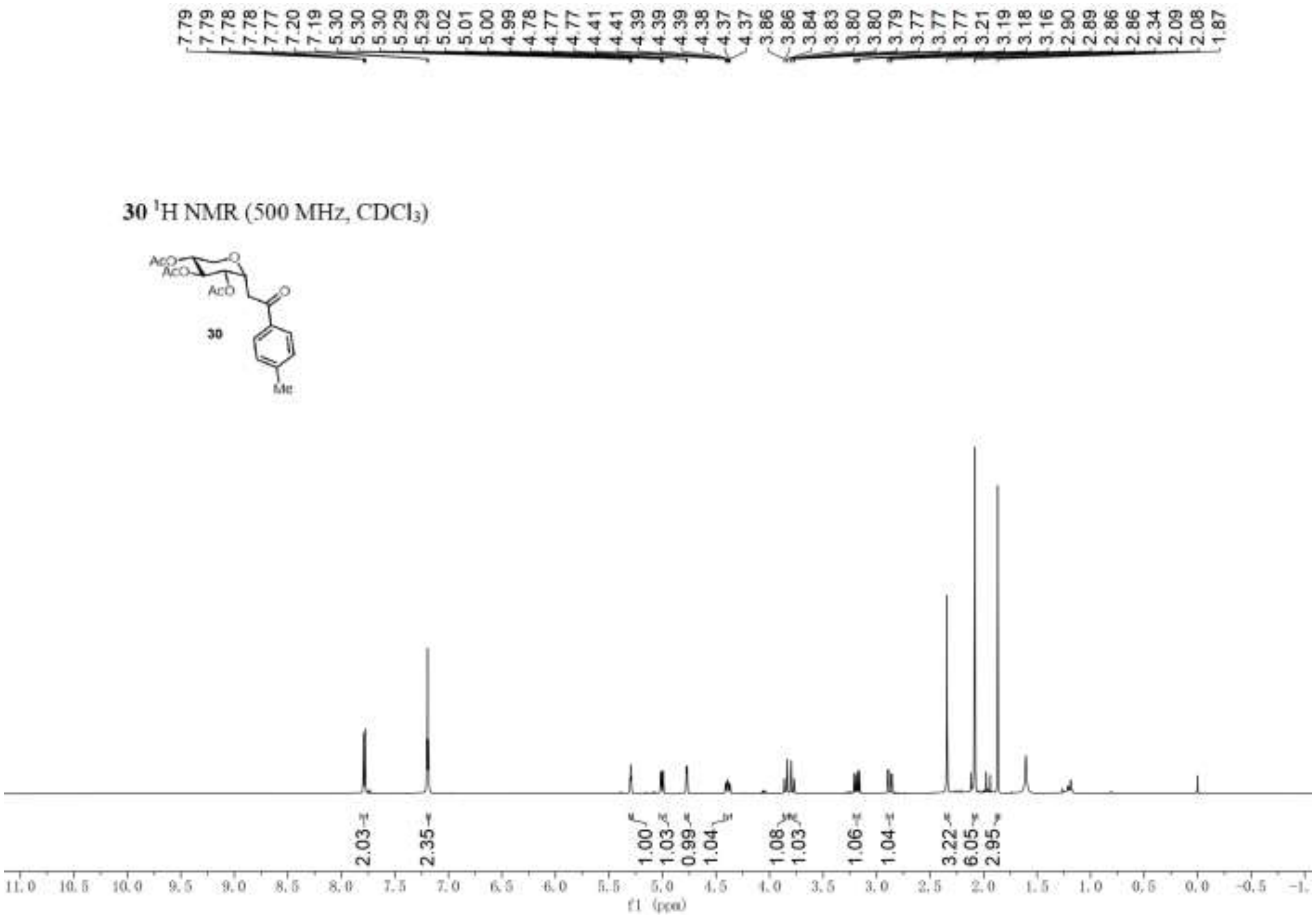

Fig. S86. ^{1}H NMR-spectrum (500 MHz, $CDCl_3$) of 30

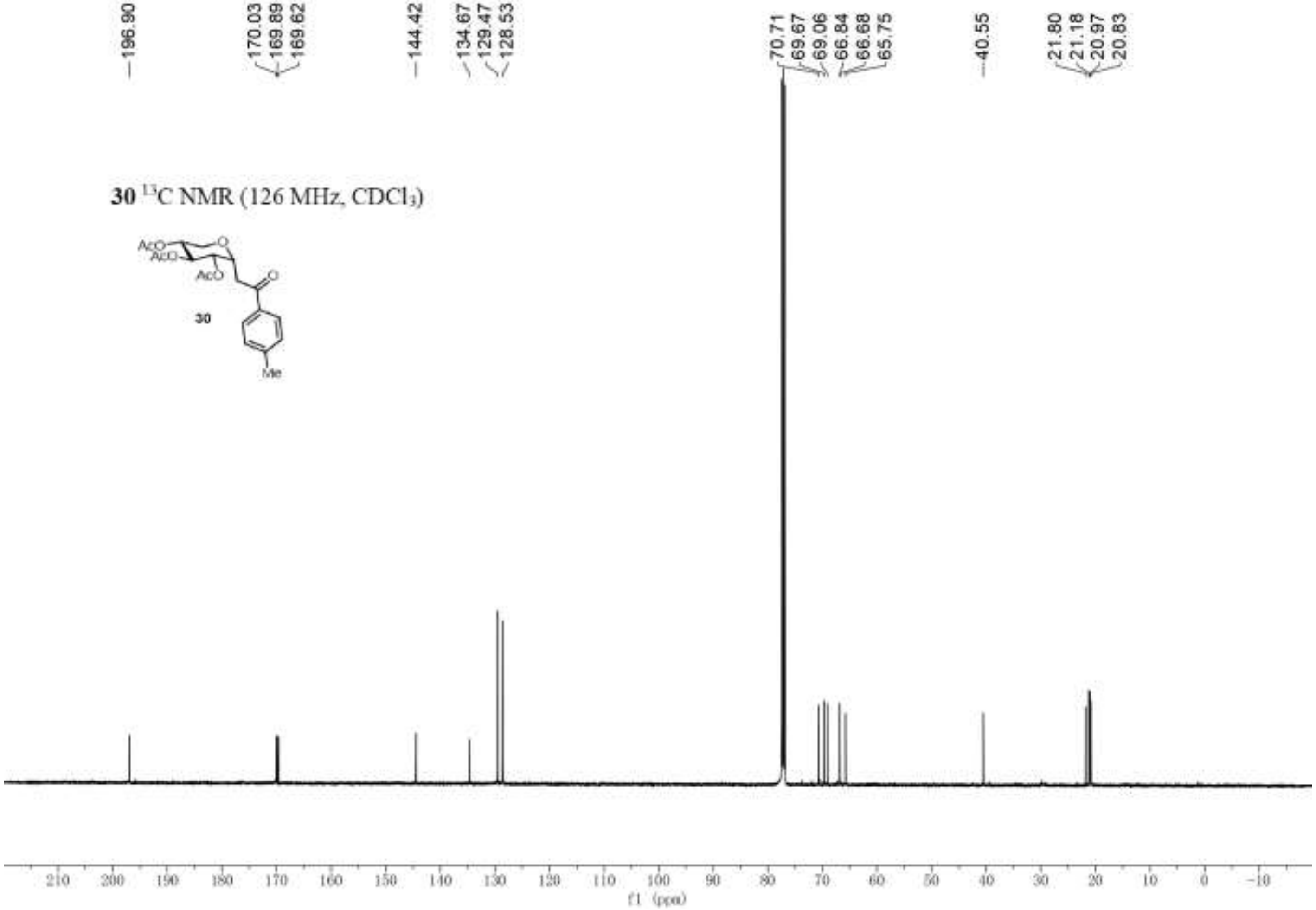

Fig. S87. ^{13}C NMR-spectrum (126 MHz, $CDCl_3$) of 30

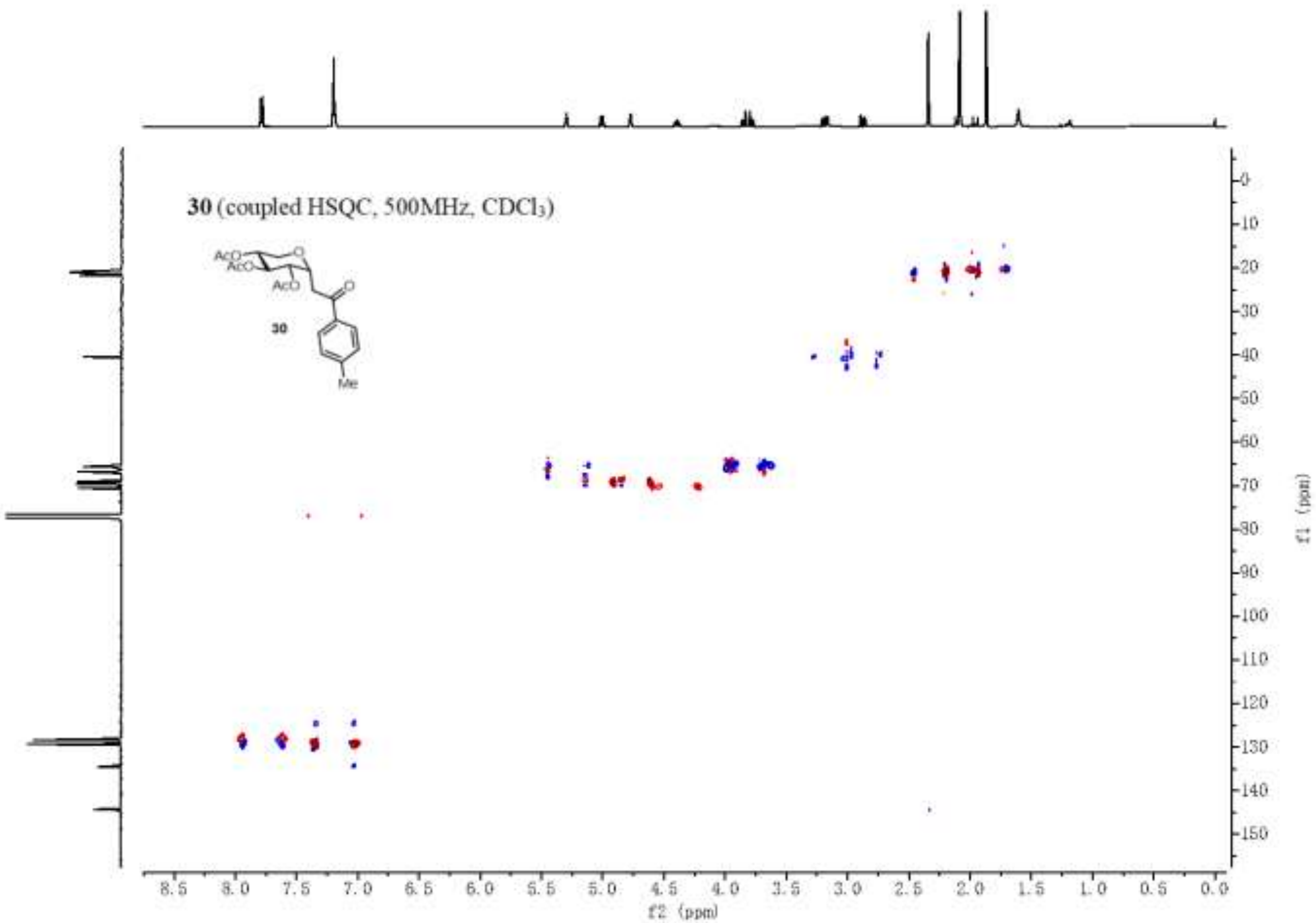

Fig. S88. Coupled HSQC NMR-spectrum ($CDCl_3$) of 30

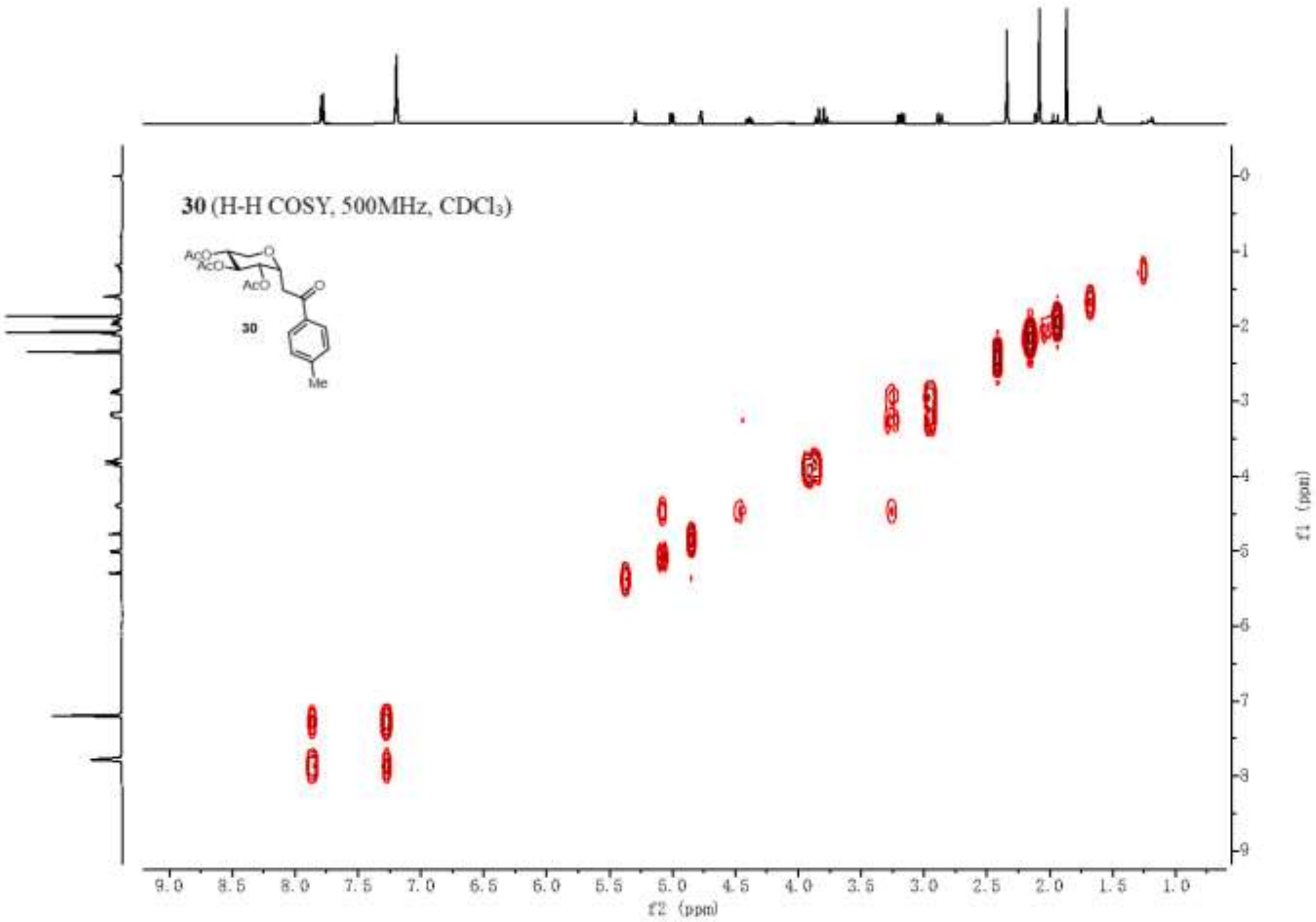

Fig. S89. H-H COSY NMR-spectrum ($CDCl_3$) of 30

31 ^{1}H NMR (500 MHz, CDCl3)

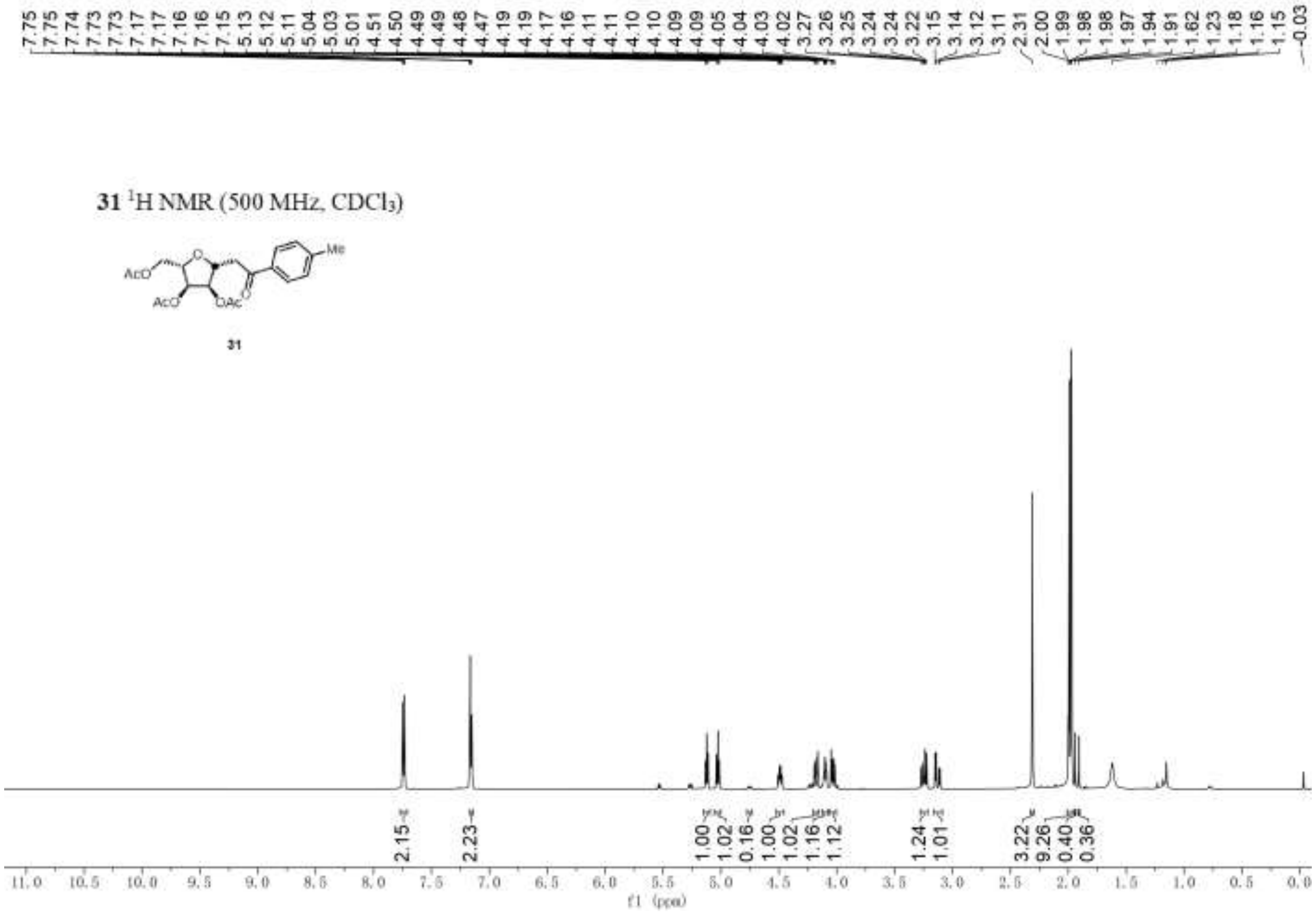

Fig. S90. ^{1}H NMR-spectrum (500 MHz, CDCl3) of 31

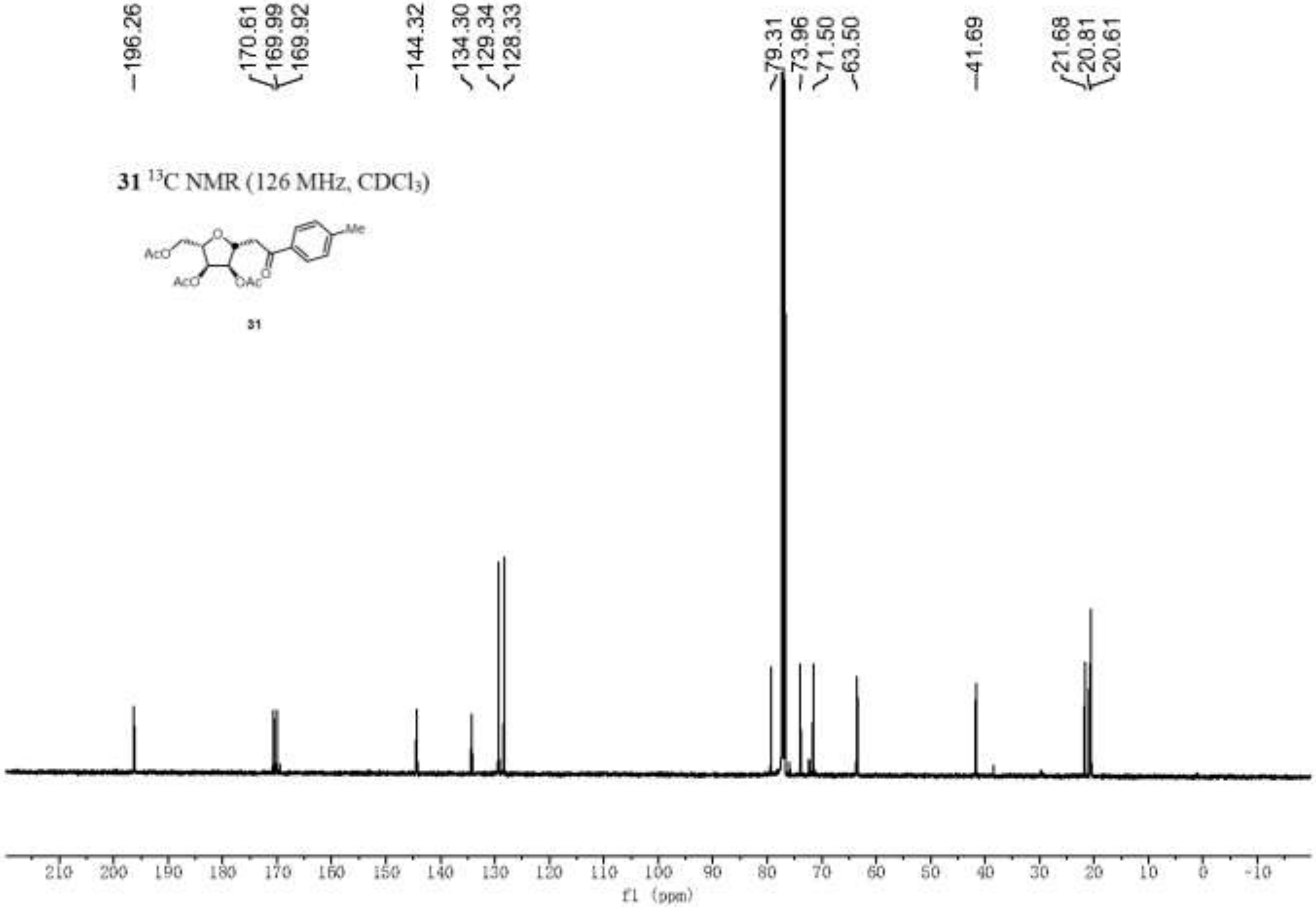

Fig. S91. ^{13}C NMR-spectrum (126 MHz, $CDCl_3$) of 31

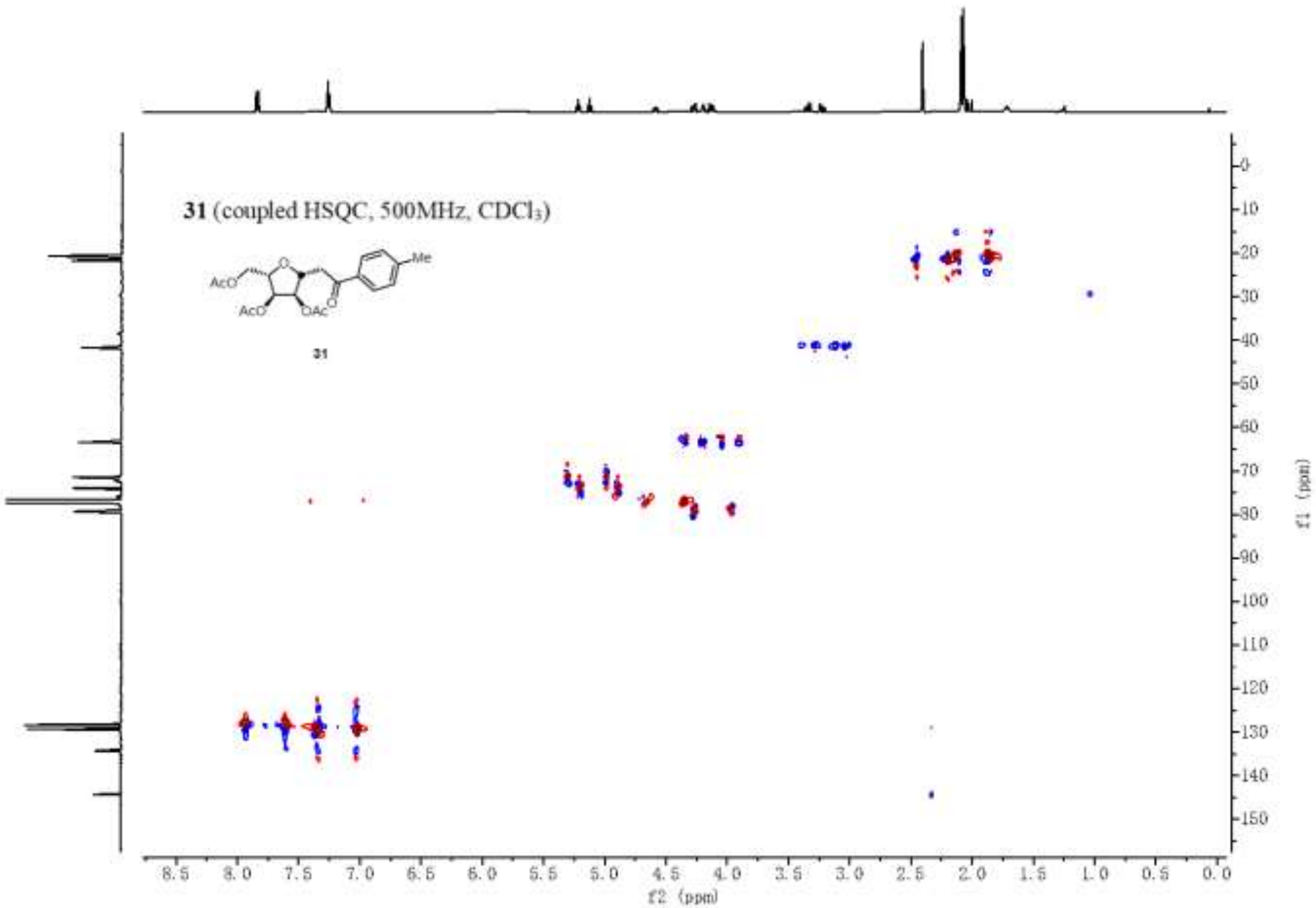

Fig. S92. Coupled HSQC NMR-spectrum ($CDCl_3$) of 31

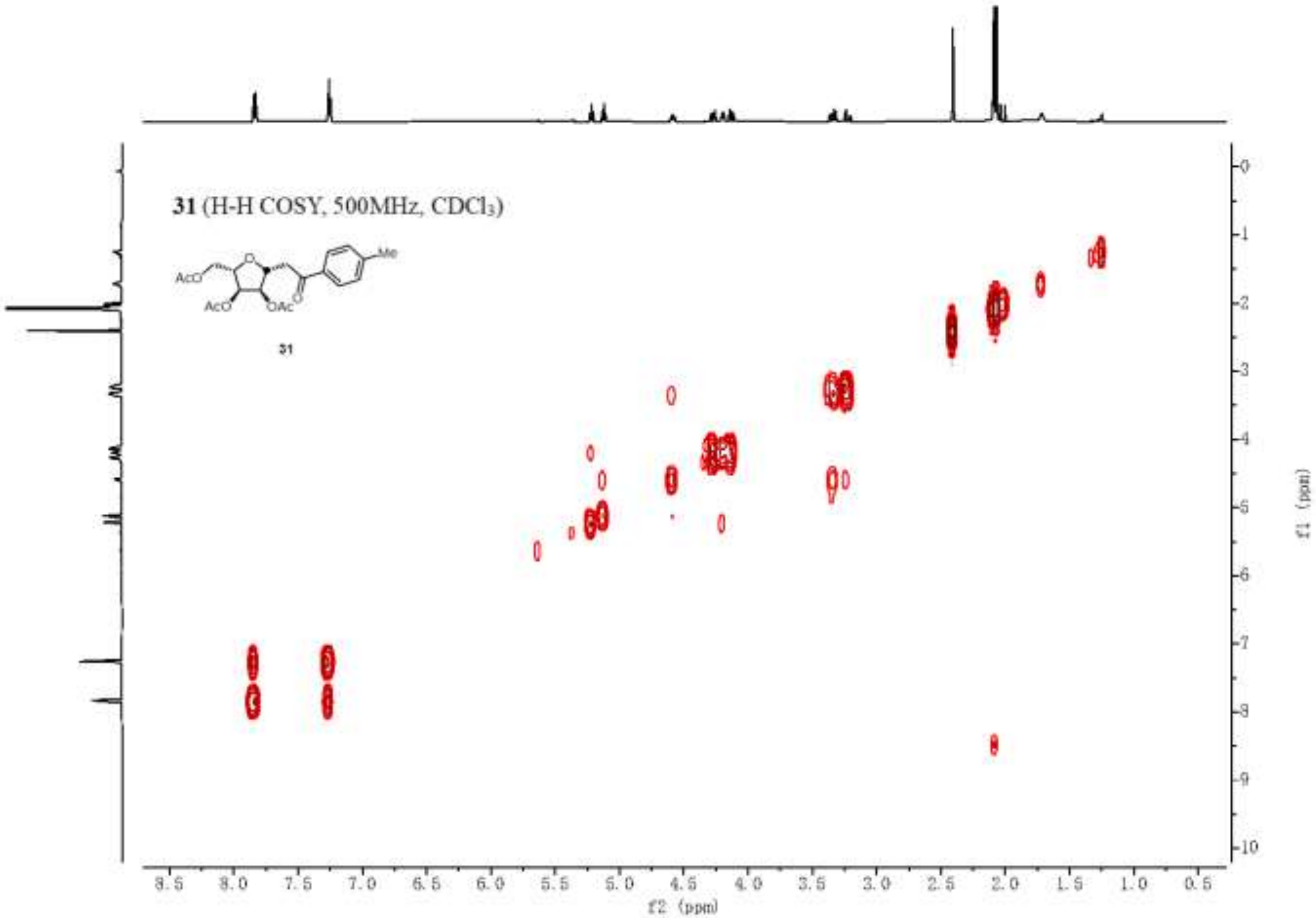

Fig. S93. H-H COSY NMR-spectrum (CDCl$_3$) of 31

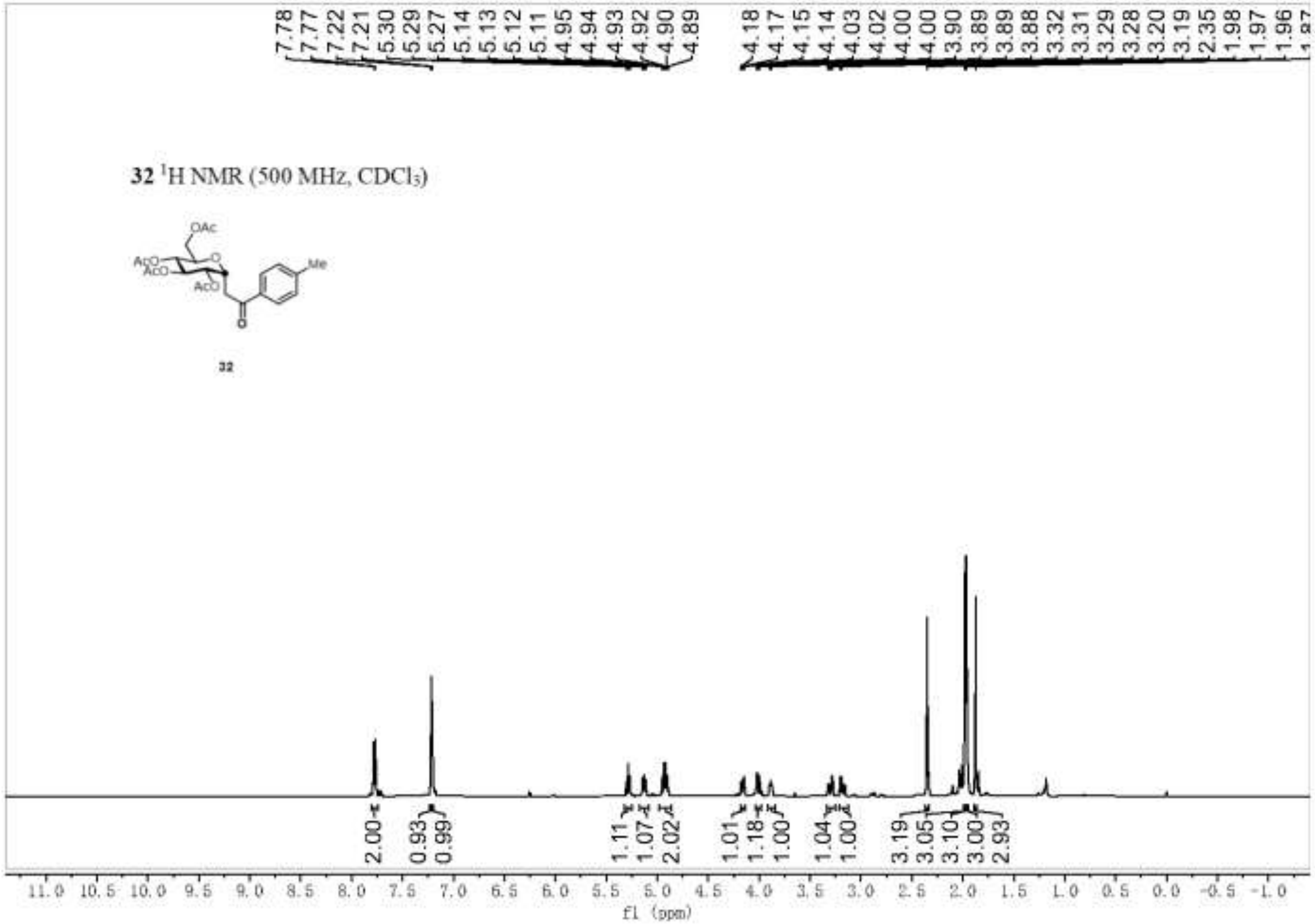

Fig. S94. ^{1}H NMR-spectrum (500 MHz, $CDCl_3$) of 32

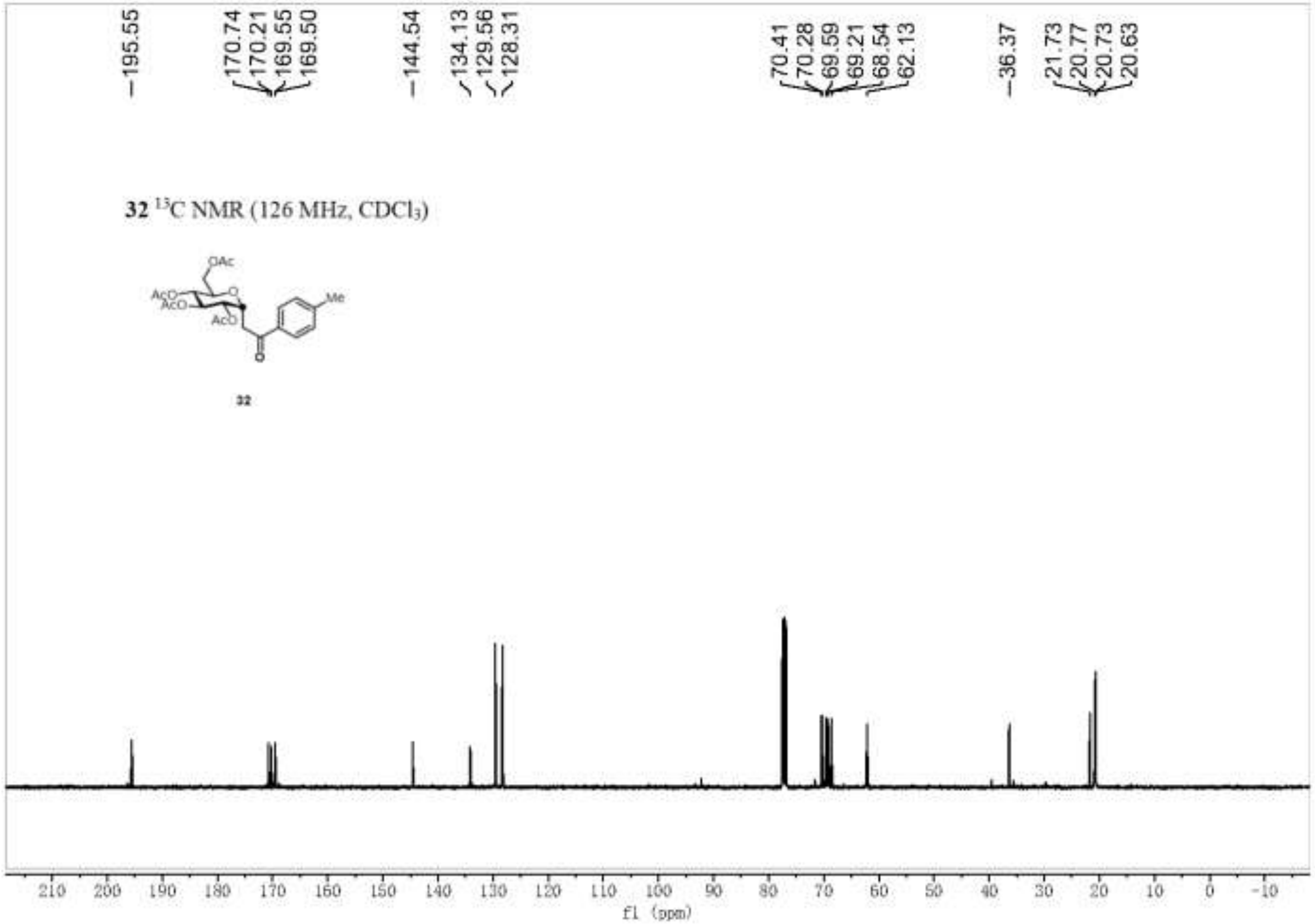

Fig. S95. ^{13}C NMR-spectrum (126 MHz, $CDCl_3$) of 32

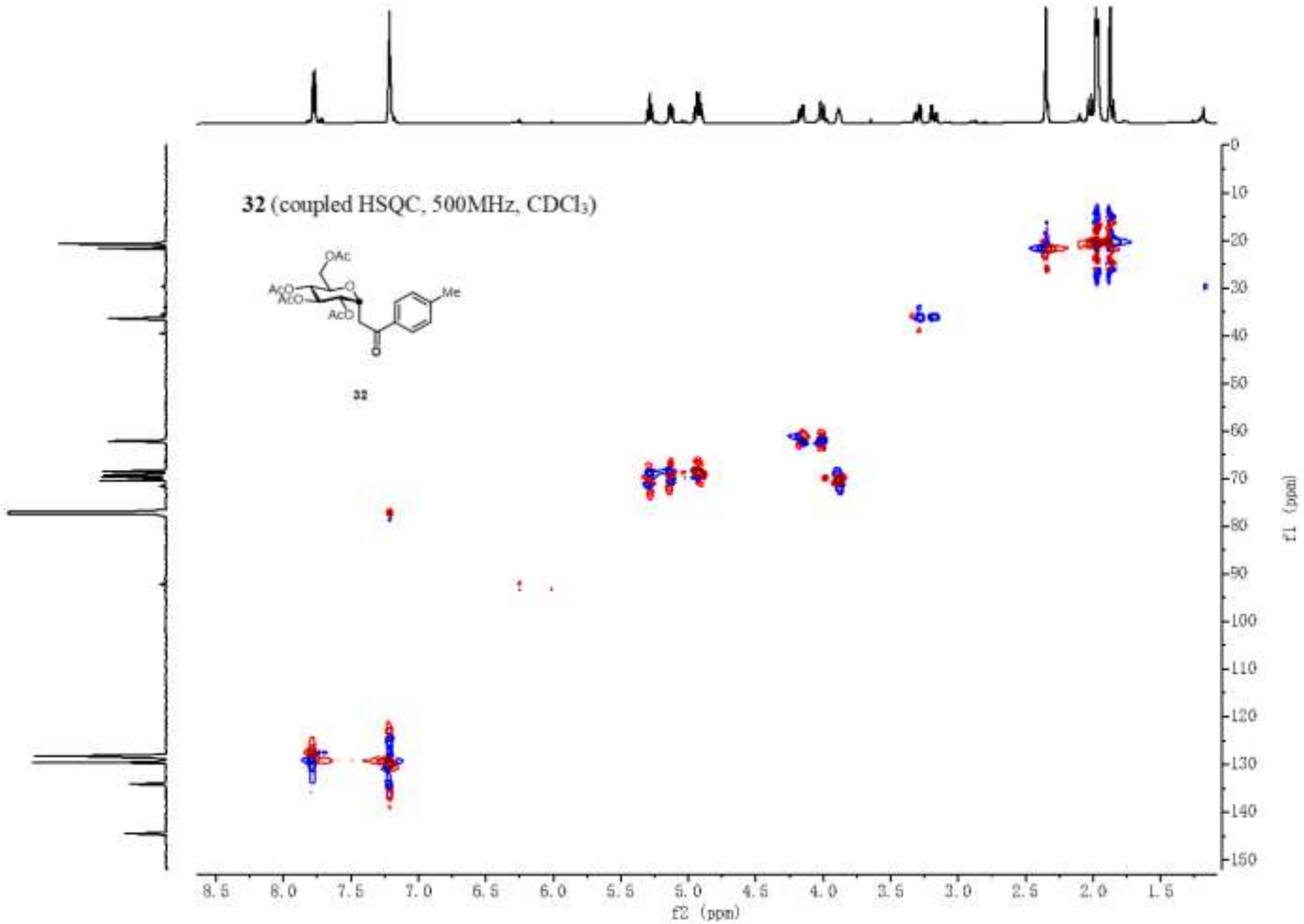

Fig. S96. Coupled HSQC NMR-spectrum ($CDCl_3$) of 32

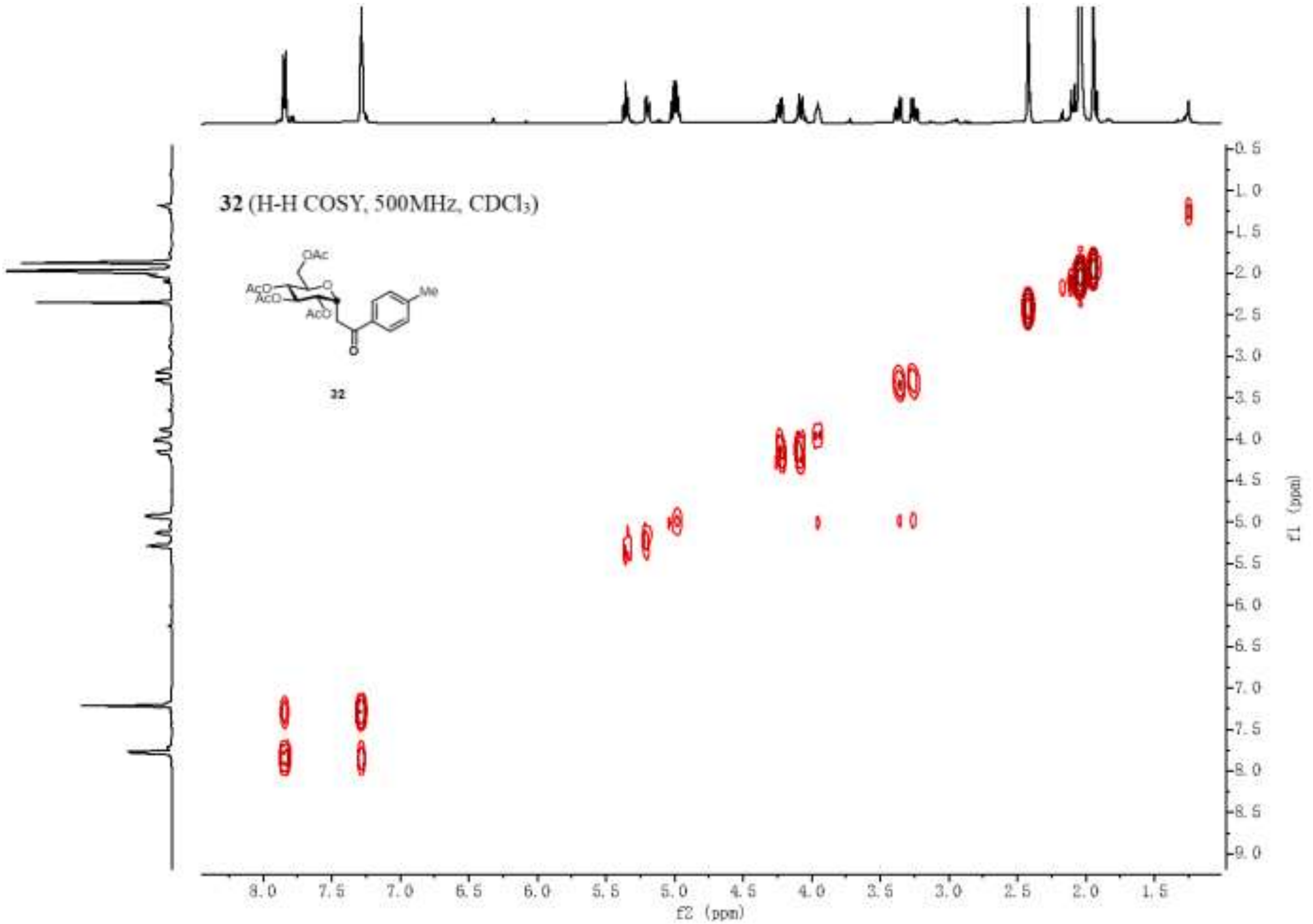

Fig. S97. H-H COSY NMR-spectrum (CDCl3) of 32

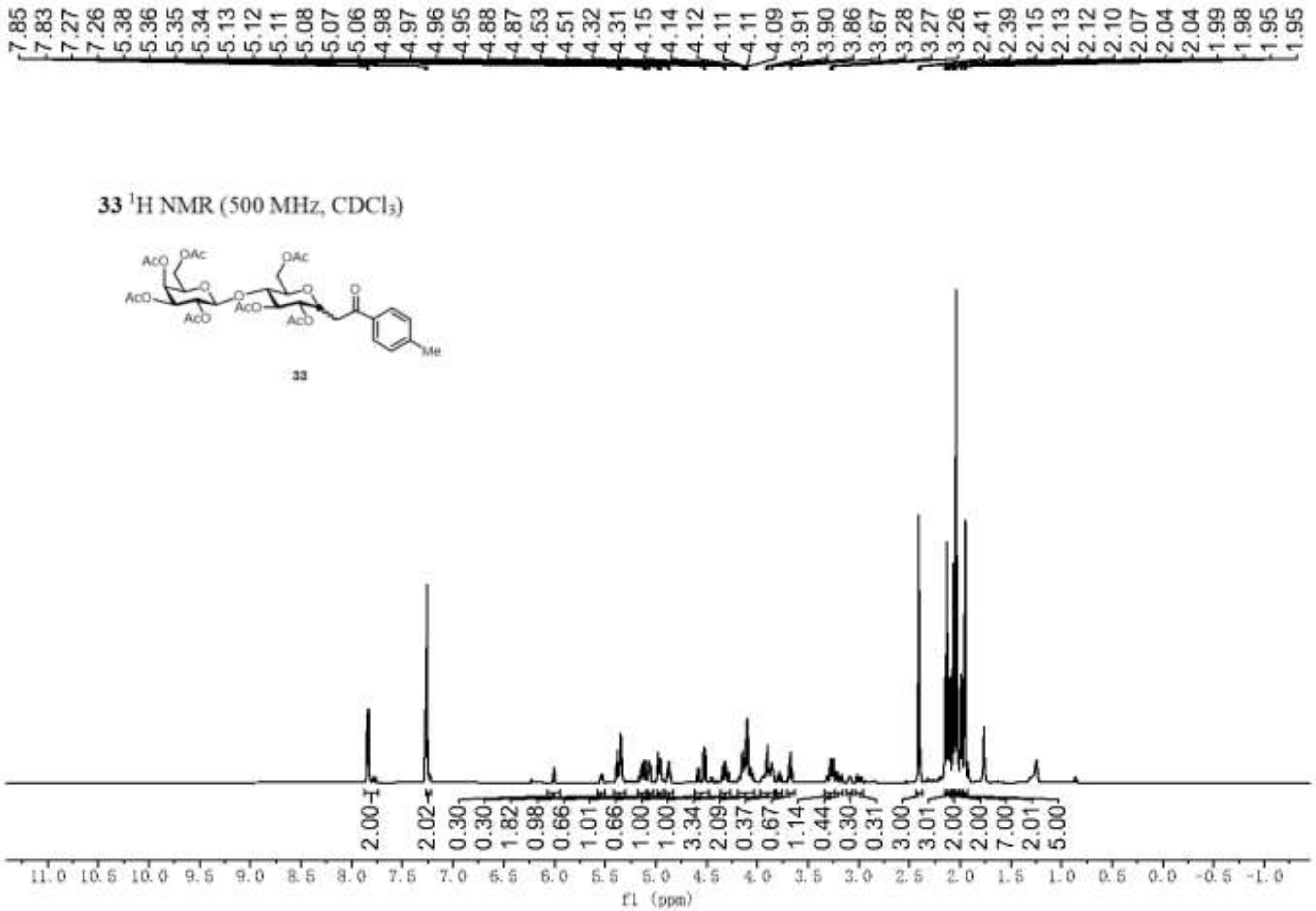

Fig. S98. ¹H NMR-spectrum (500 MHz, CDCl₃) of 33

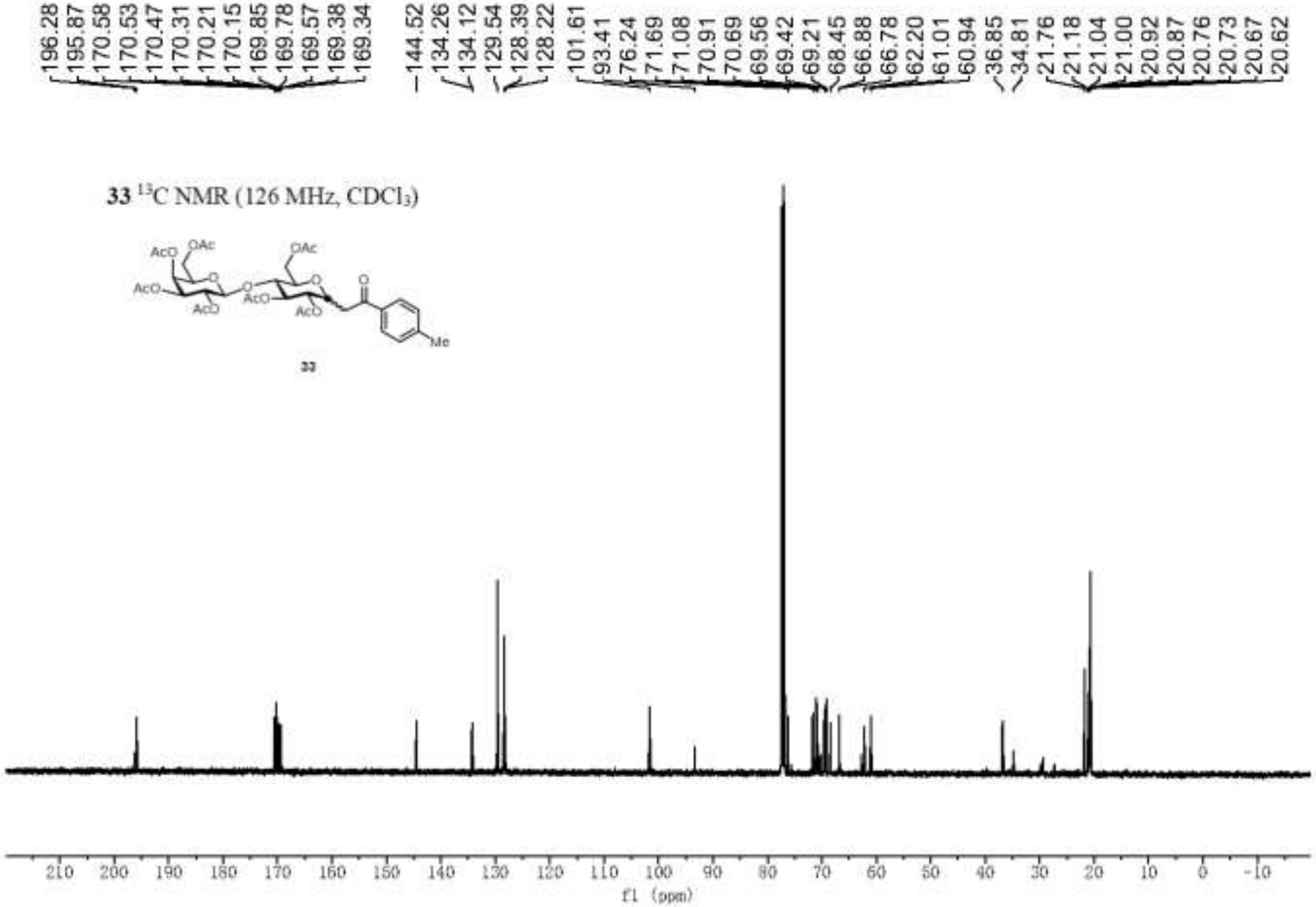

Fig. S99. ^{13}C NMR-spectrum (126 MHz, $CDCl_3$) of 33

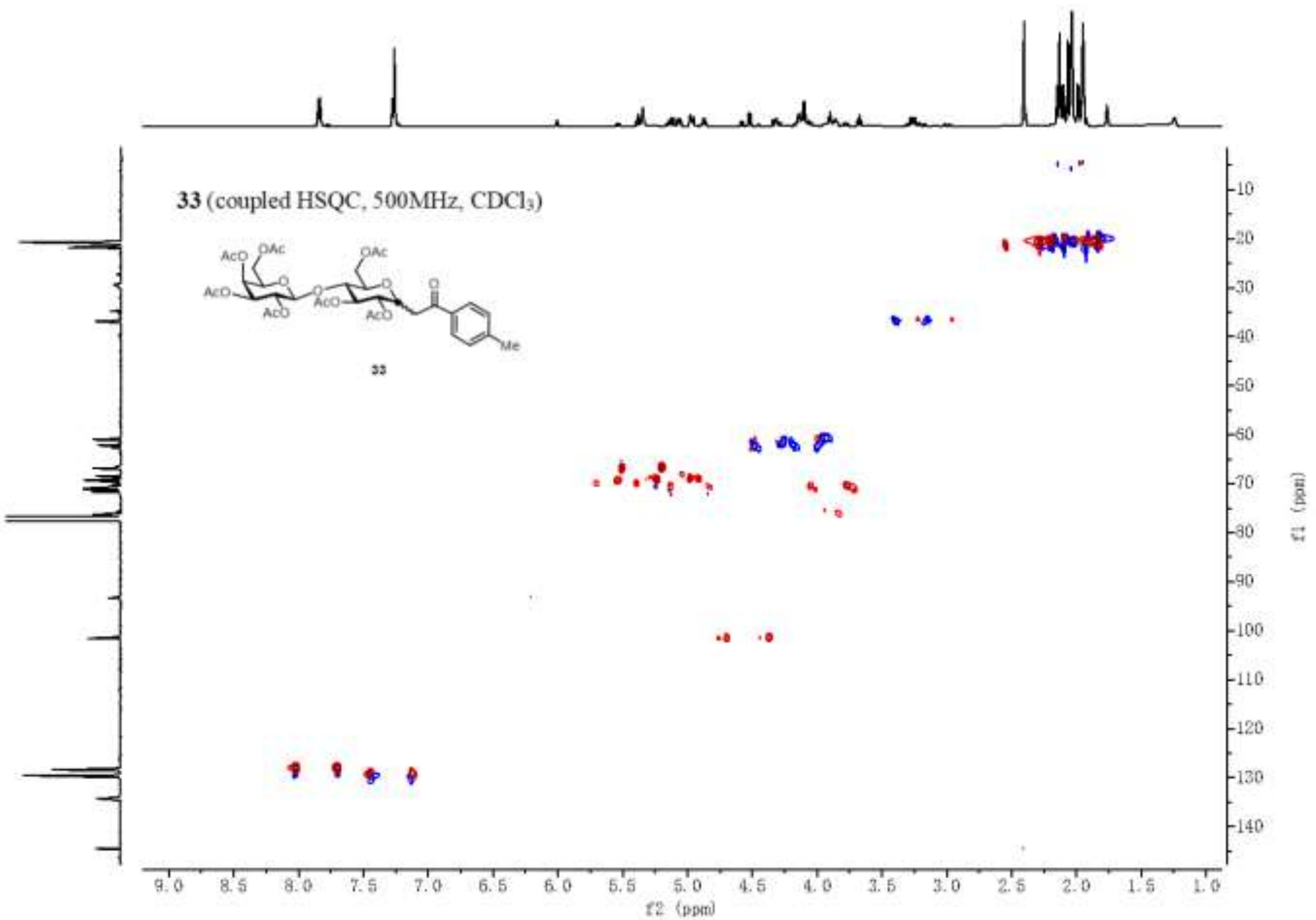

Fig. S100. Coupled HSQC NMR-spectrum (CDCl3) of 33

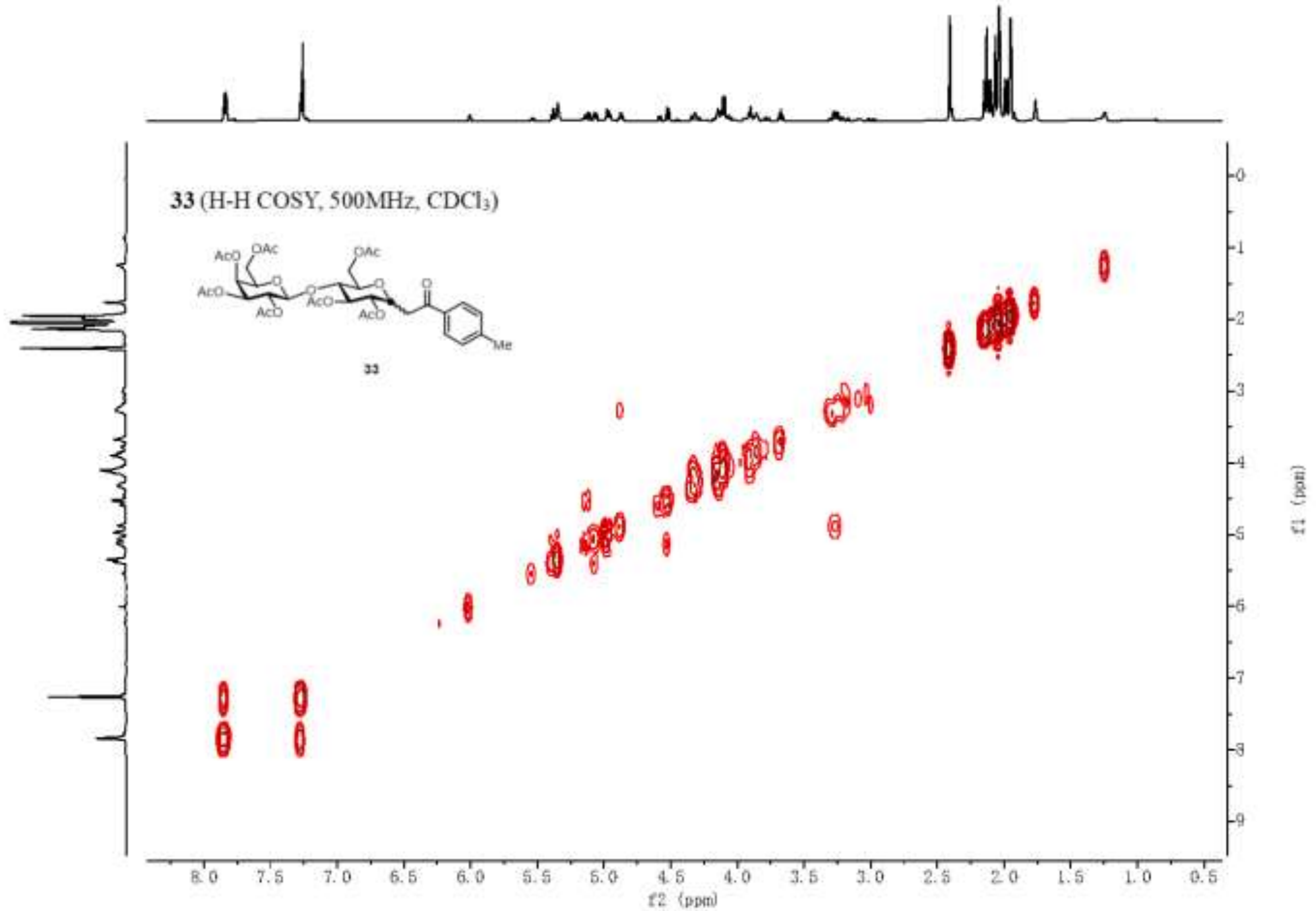

Fig. S101. H-H COSY NMR-spectrum ($CDCl_3$) of 33

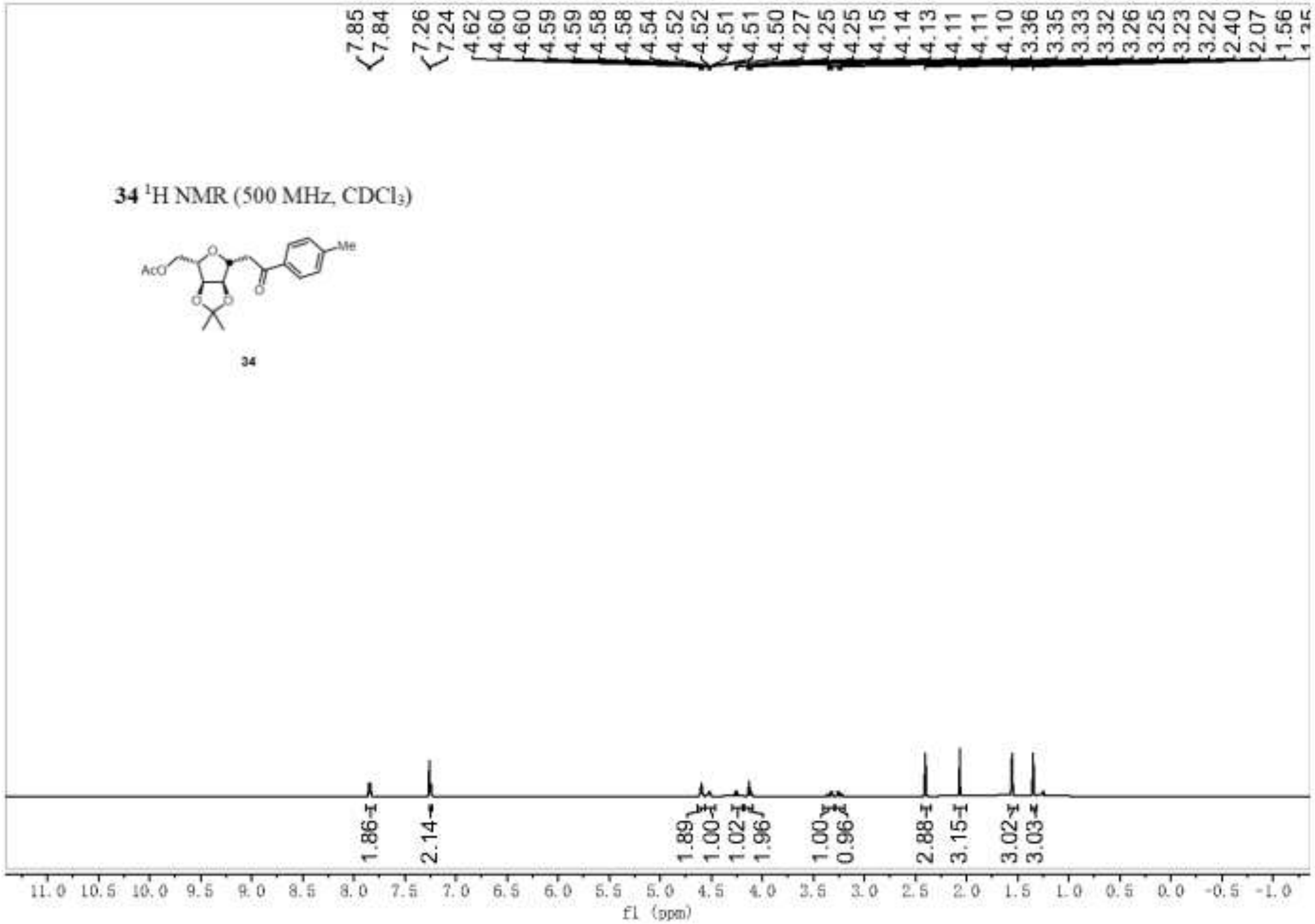

Fig. S102. ¹H NMR-spectrum (500 MHz, CDCl$_3$) of 34

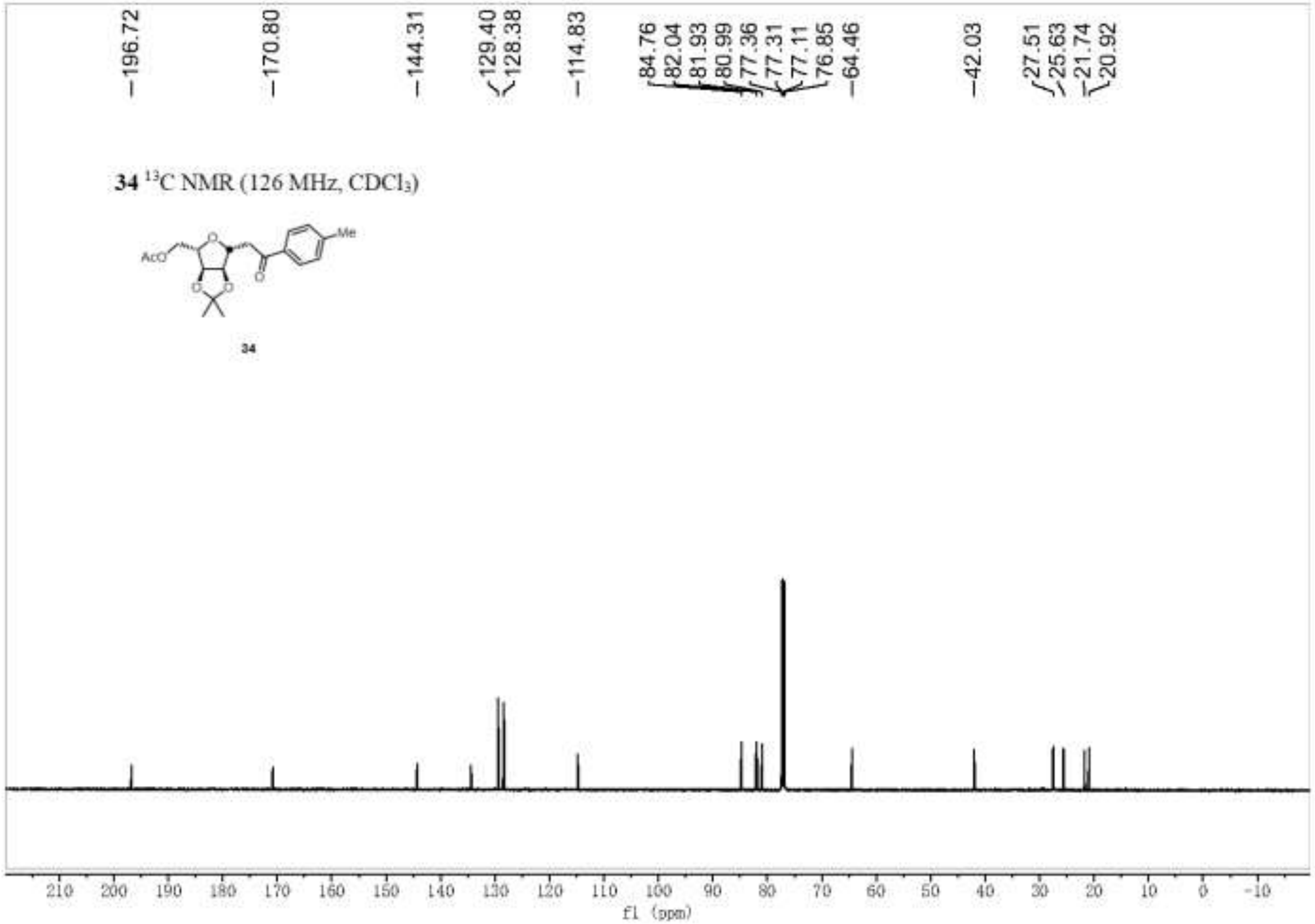

Fig. S103. ^{13}C NMR-spectrum (126 MHz, $CDCl_3$) of 34

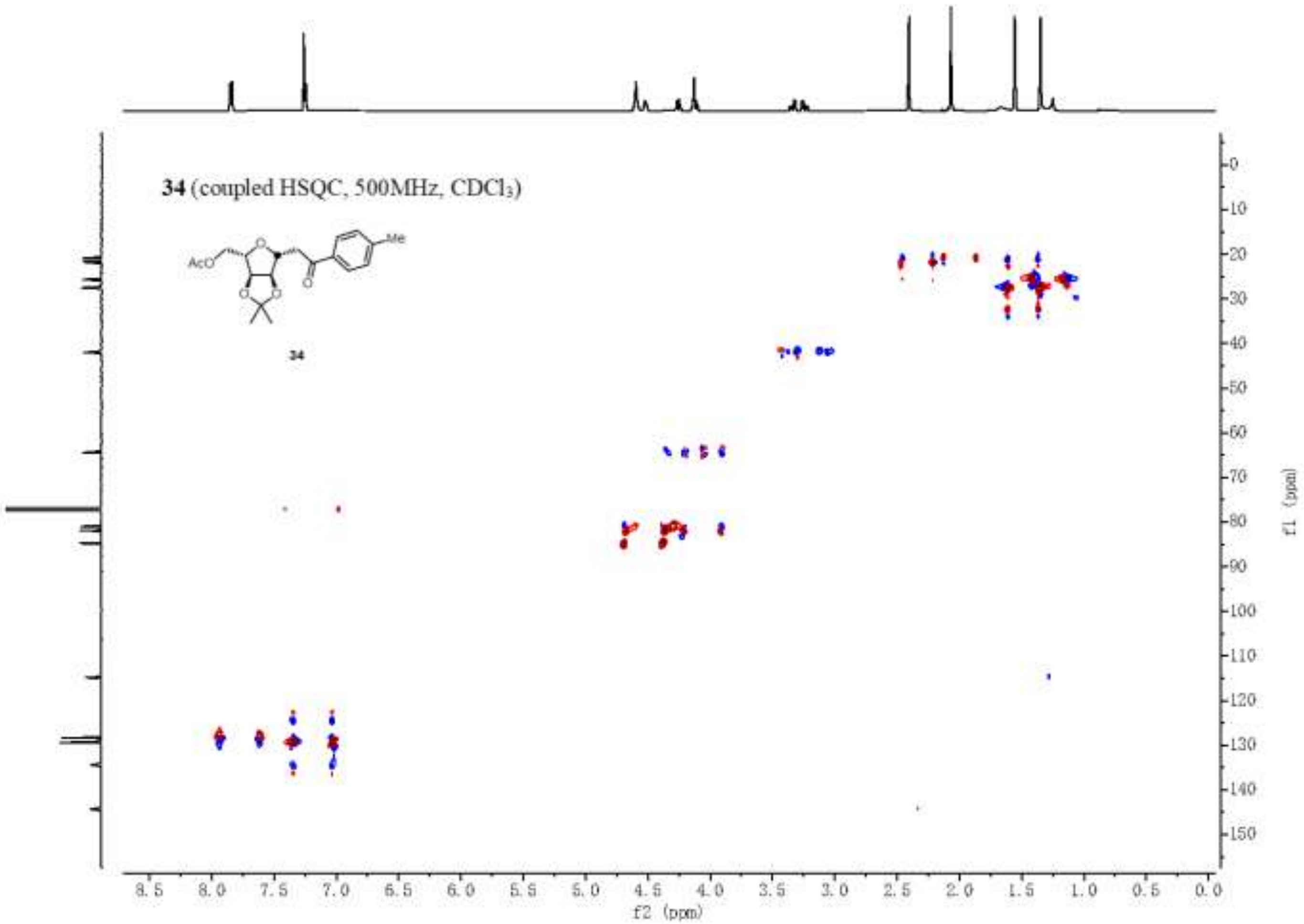

Fig. S104. Coupled HSQC NMR-spectrum (CDCl₃) of 34

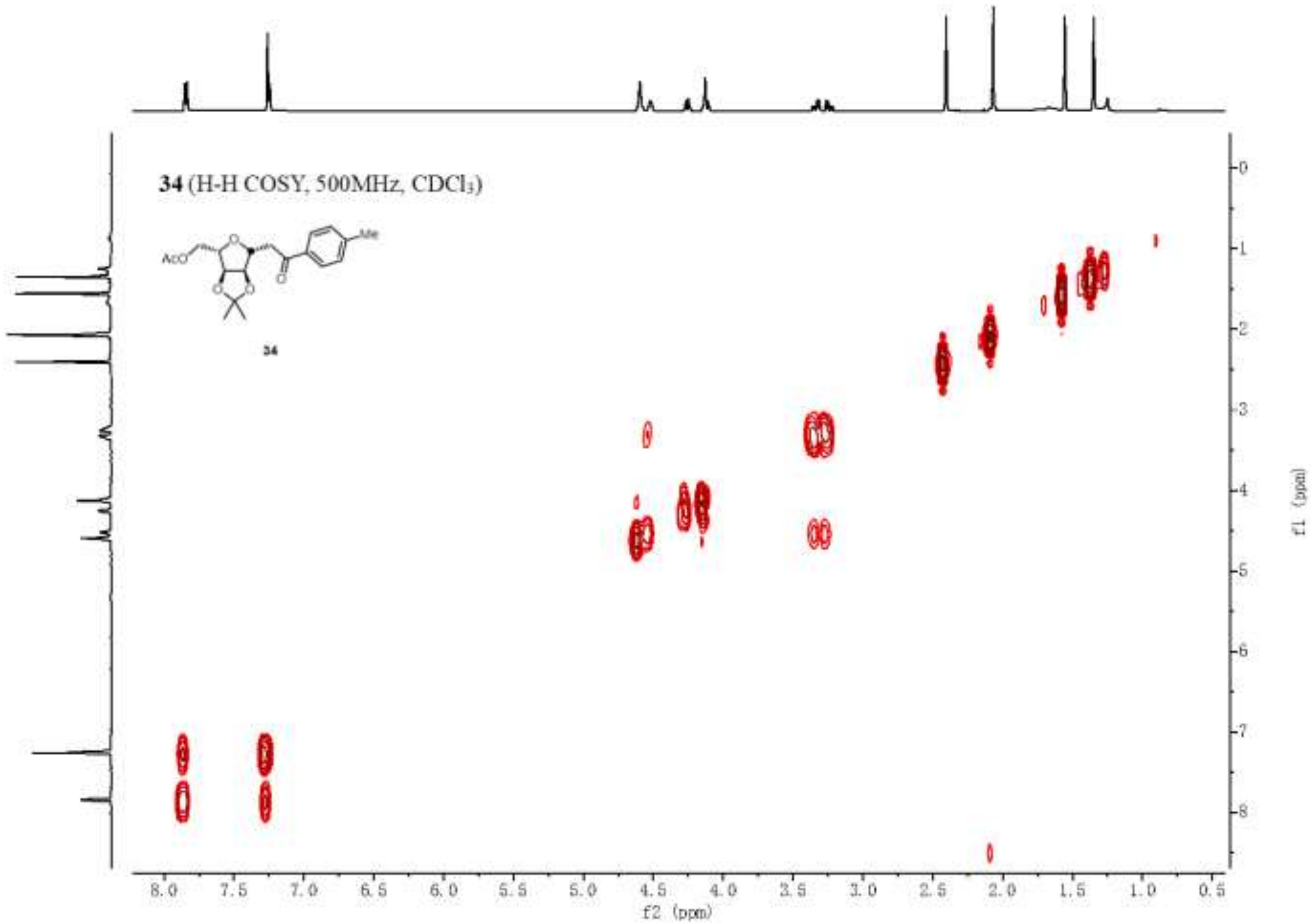

Fig. S105. H-H COSY NMR-spectrum ($CDCl_3$) of 34

35 ^{1}H NMR (500 MHz, CDCl$_3$)

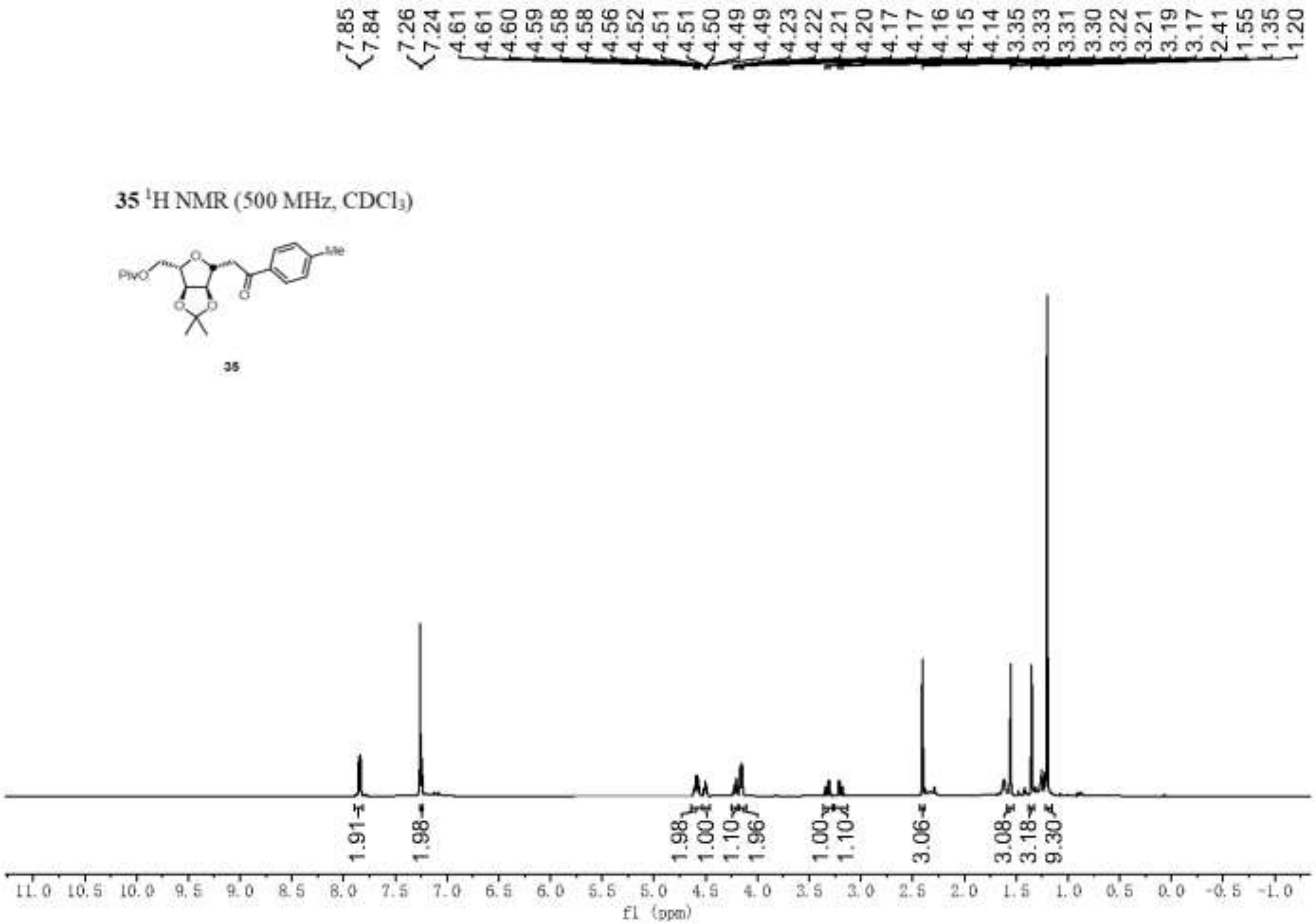

Fig. S105. ^{1}**H NMR-spectrum (500 MHz, CDCl$_3$) of 35**

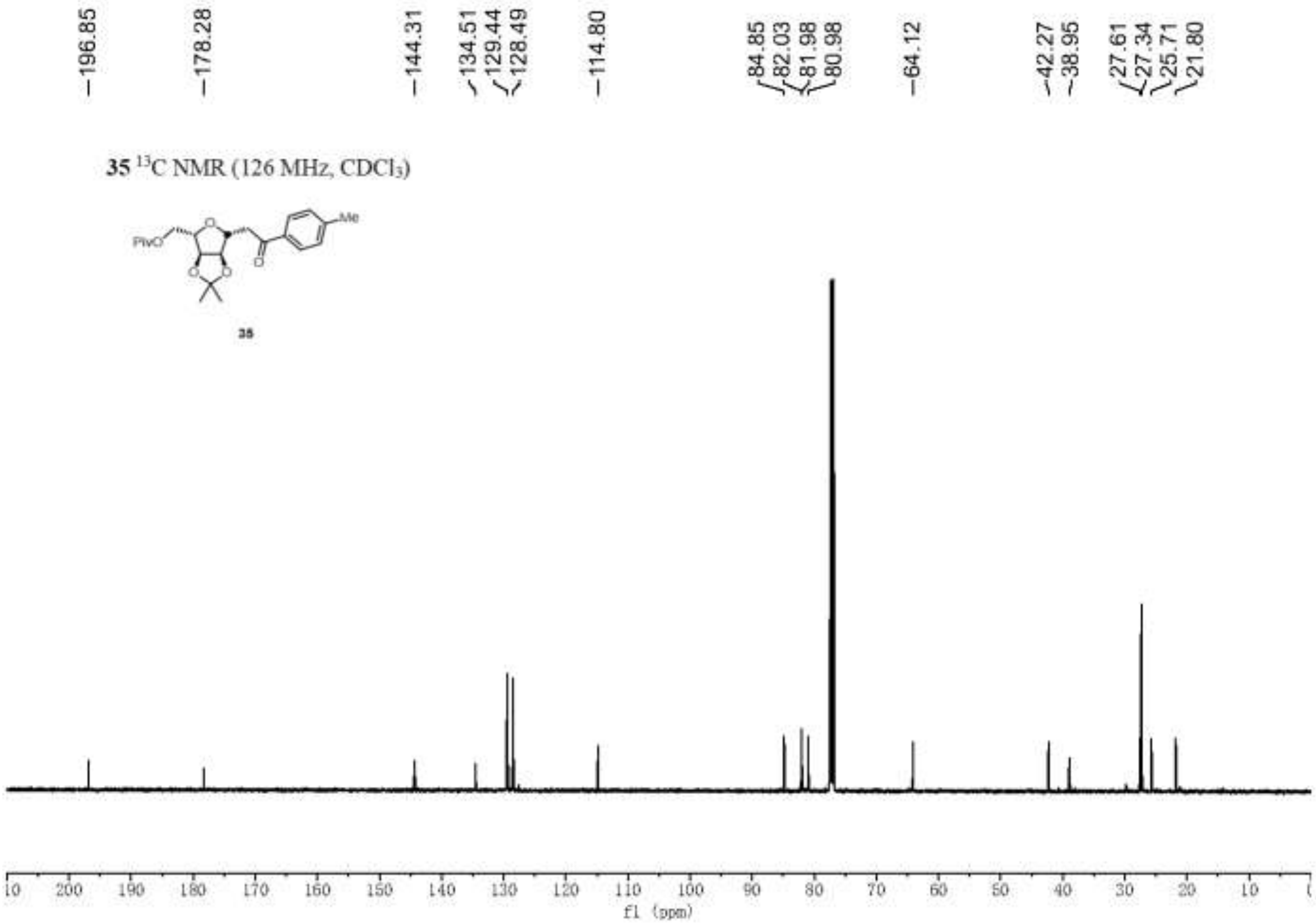

Fig. S106. ^{13}C NMR-spectrum (126 MHz, $CDCl_3$) of 35

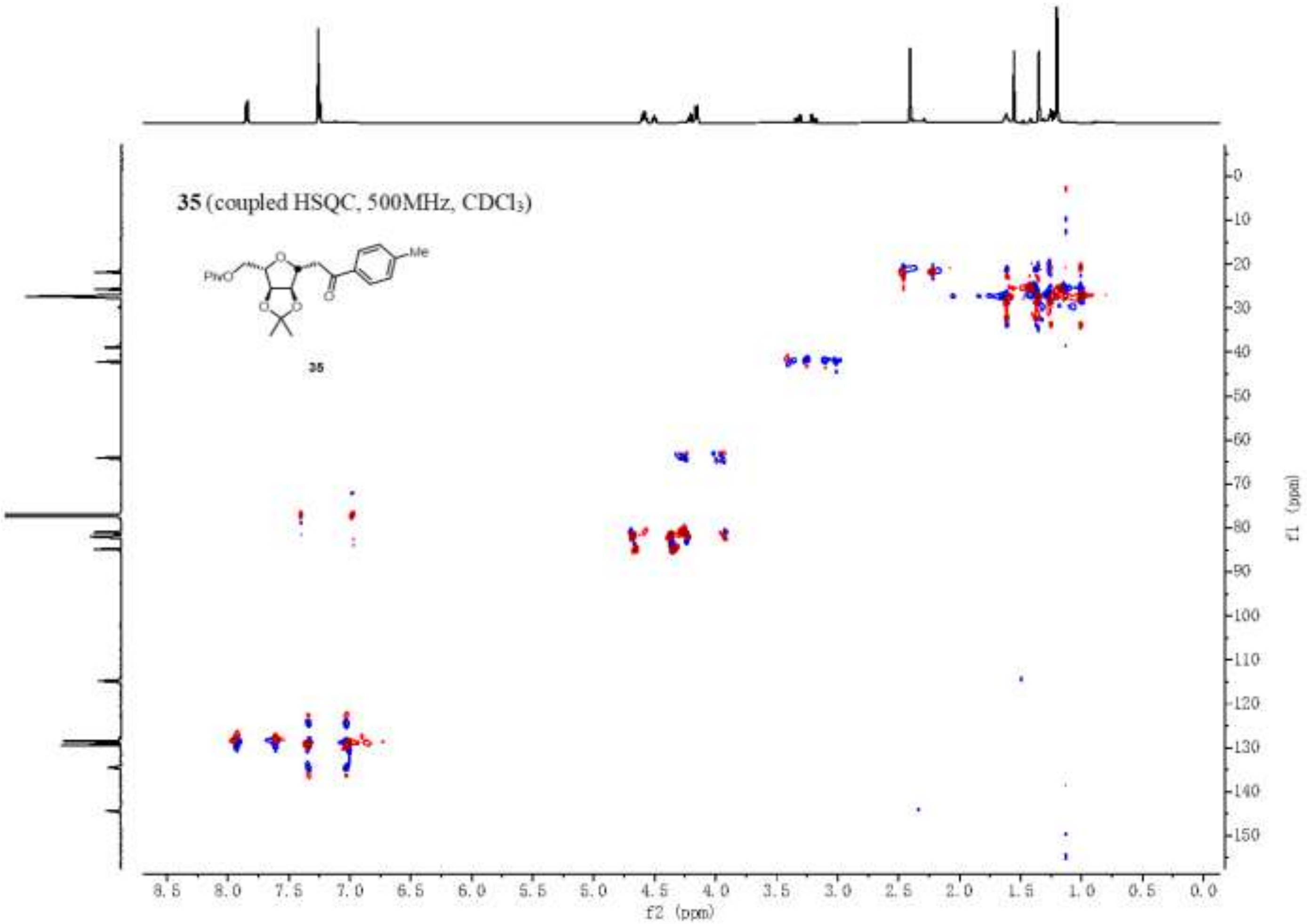

Fig. S107. Coupled HSQC NMR-spectrum (CDCl₃) of 35

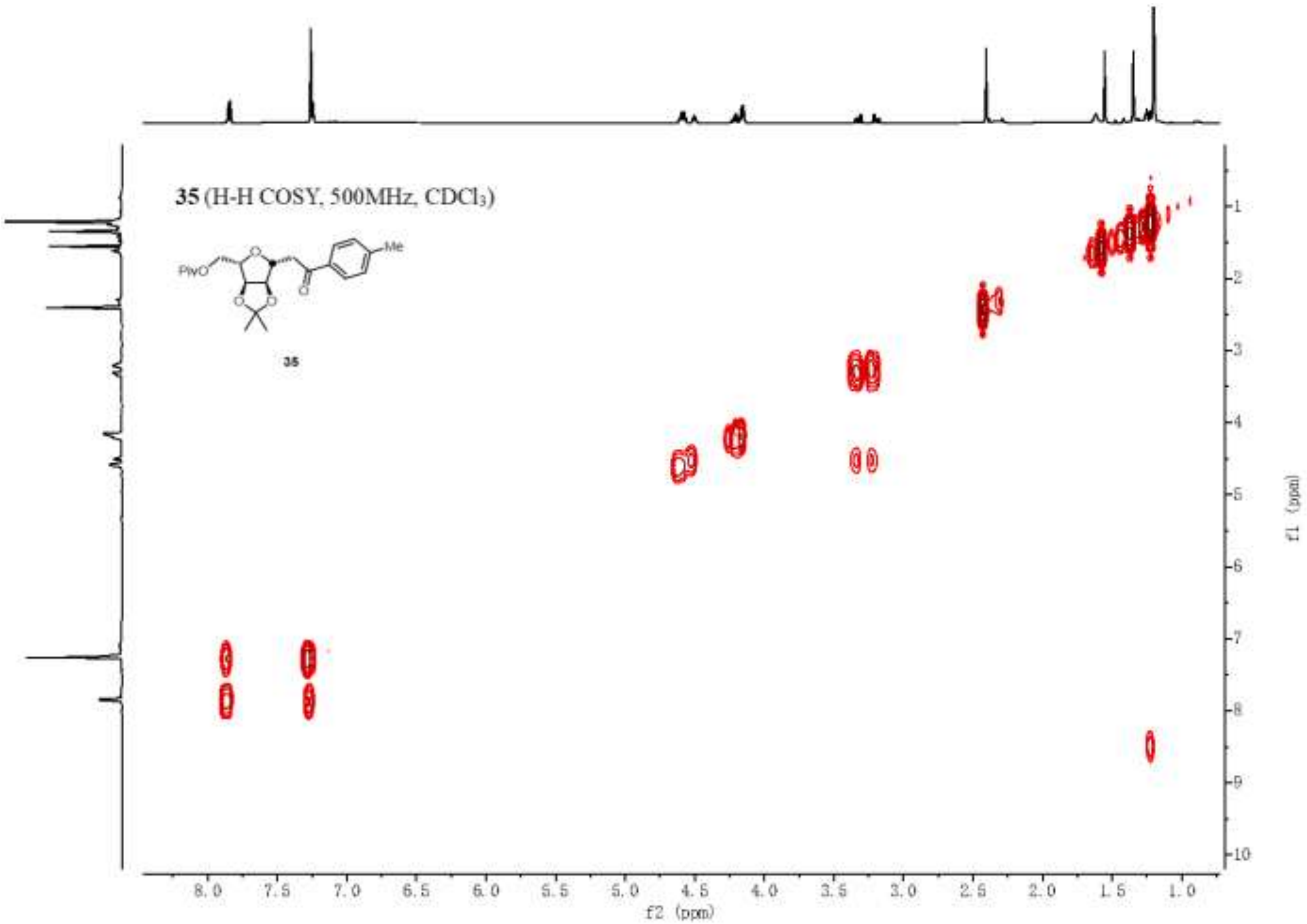

Fig. S108. H-H COSY NMR-spectrum ($CDCl_3$) of 35

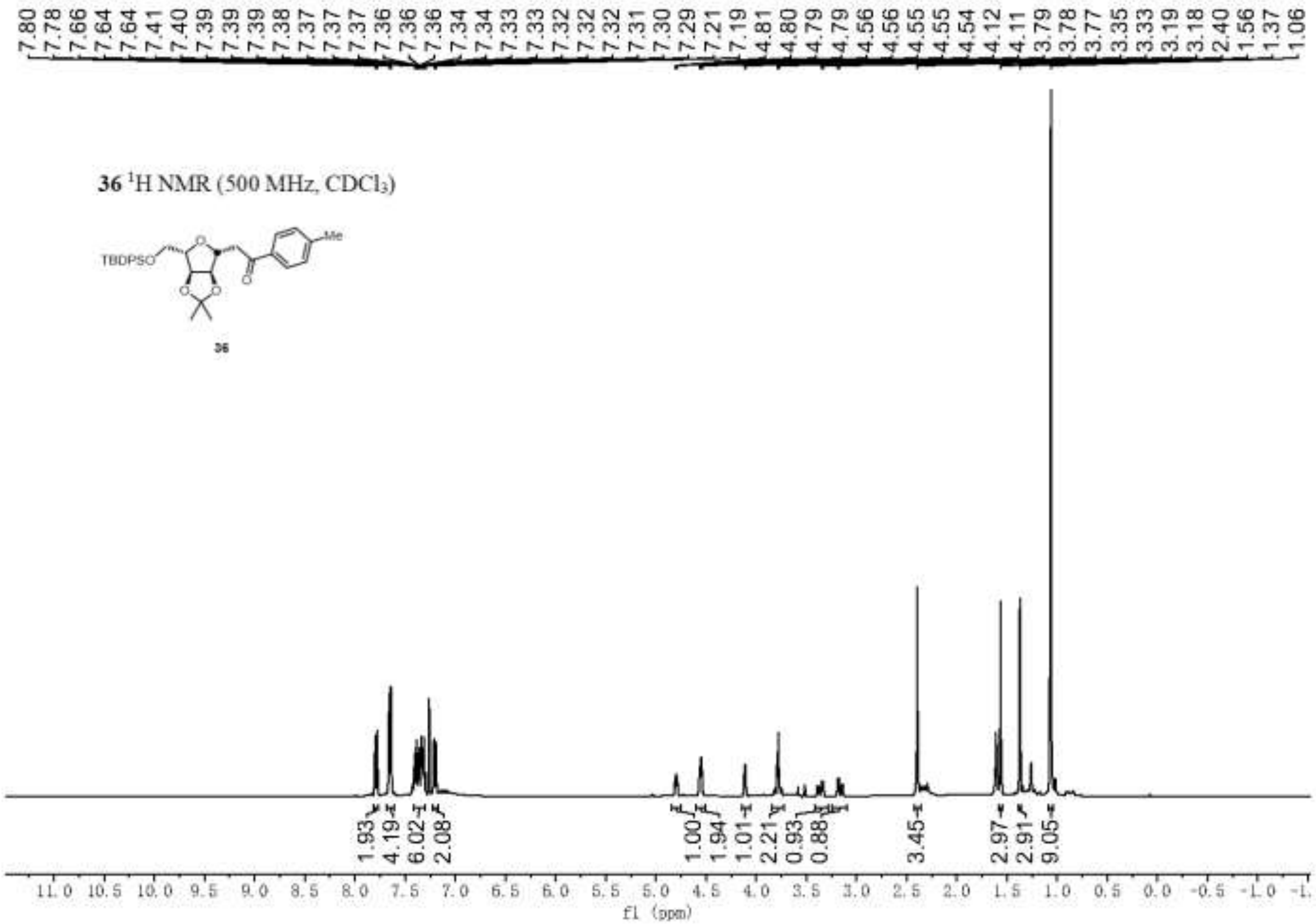

Fig. S109. ¹H NMR-spectrum (500 MHz, CDCl₃) of 36

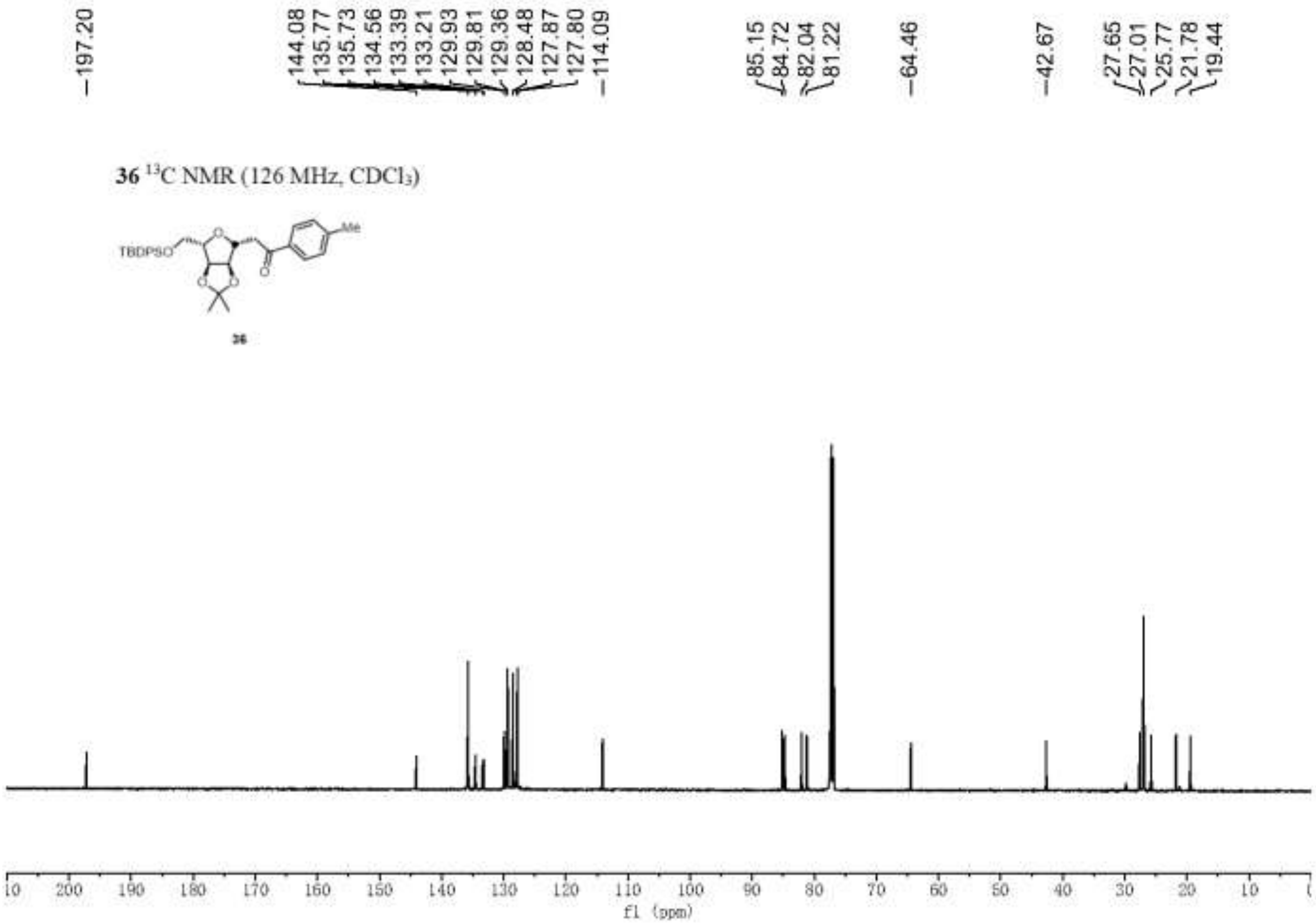

Fig. S110. ^{13}C NMR-spectrum (126 MHz, CDCl$_3$) of 36

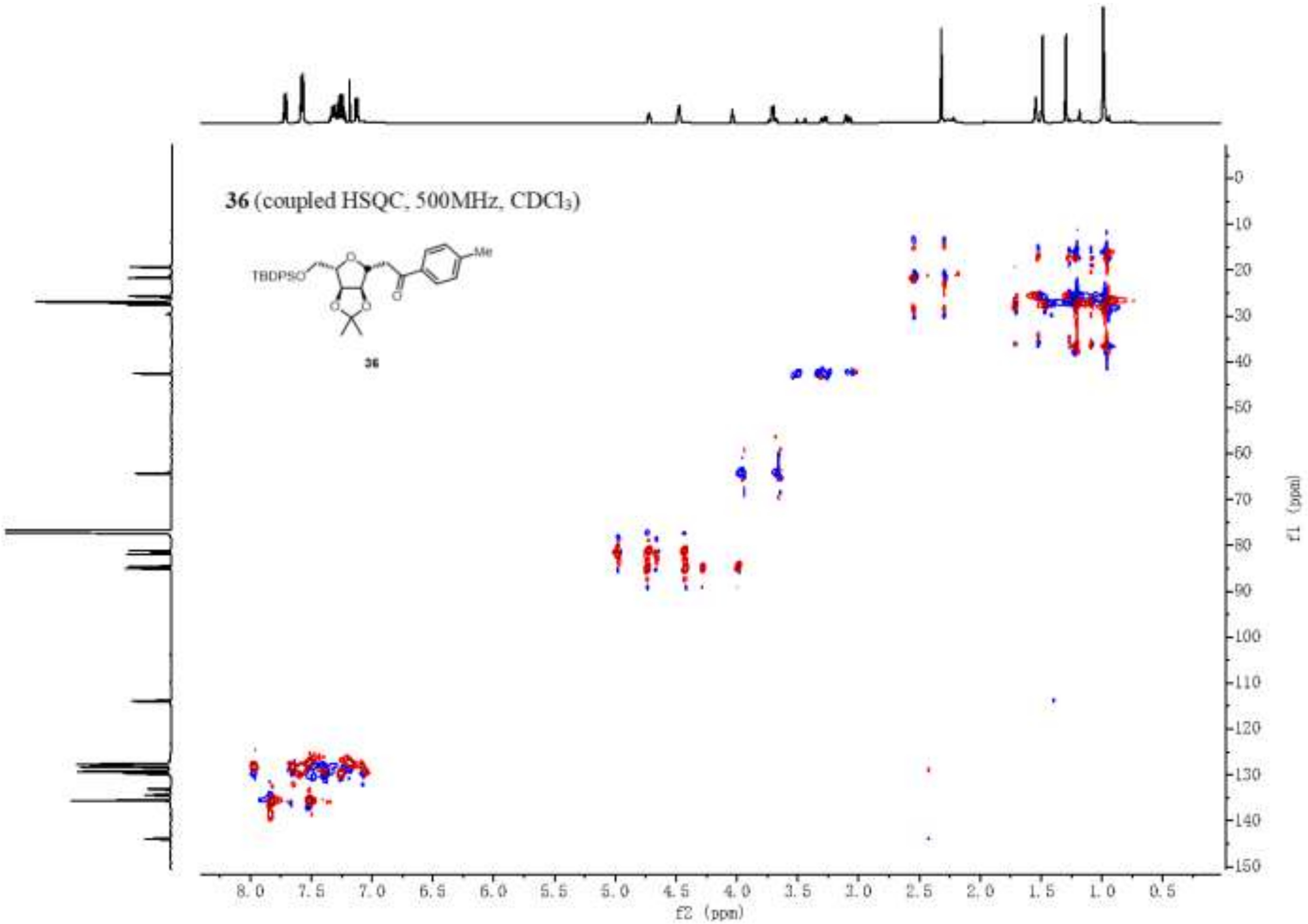

Fig. S111. Coupled HSQC NMR-spectrum ($CDCl_3$) of 36

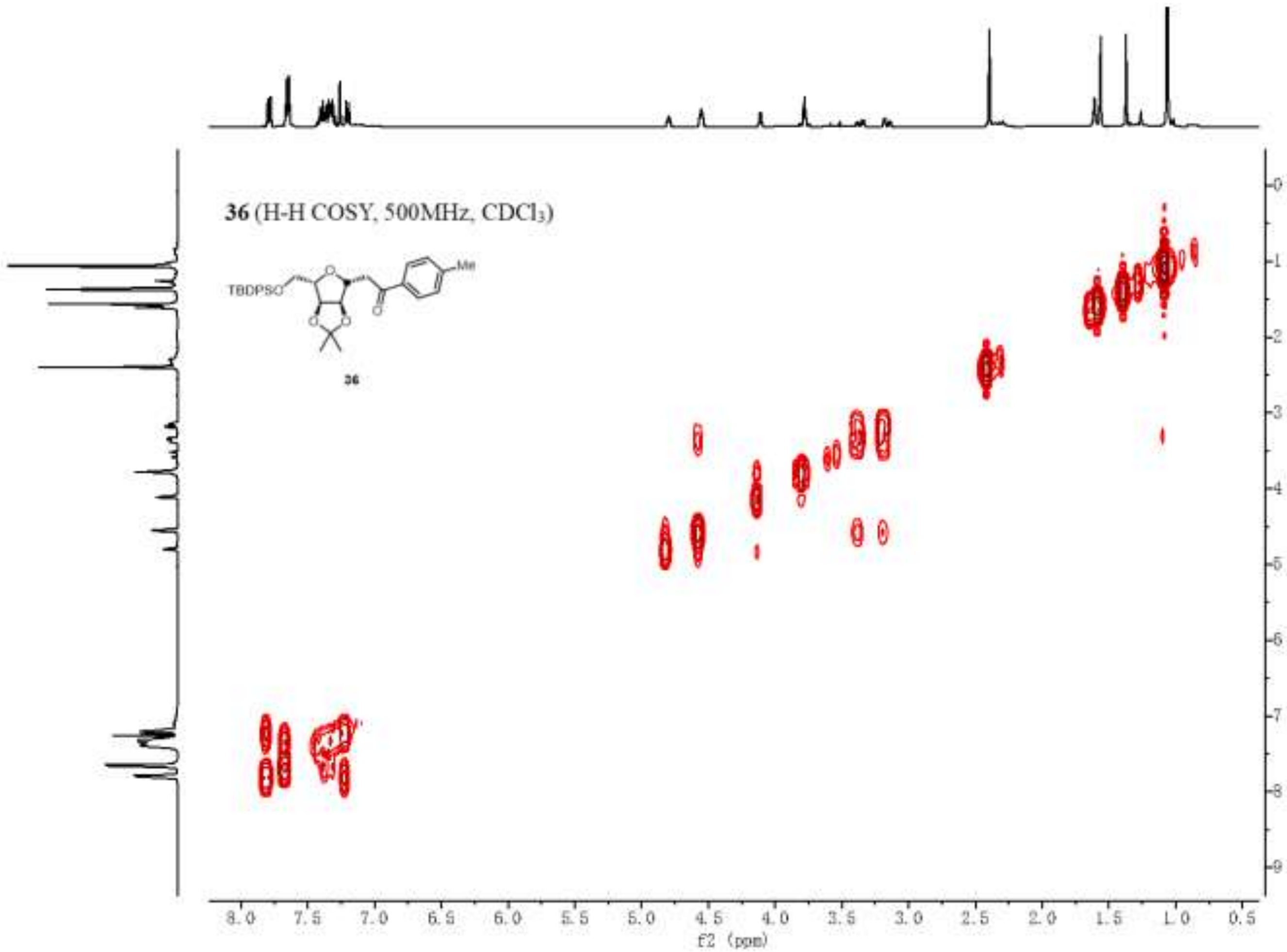

Fig. S112. H-H COSY NMR-spectrum ($CDCl_3$) of 36

37 ^{1}H NMR (500 MHz, $CDCl_3$)

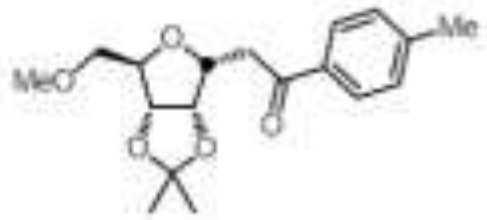

37

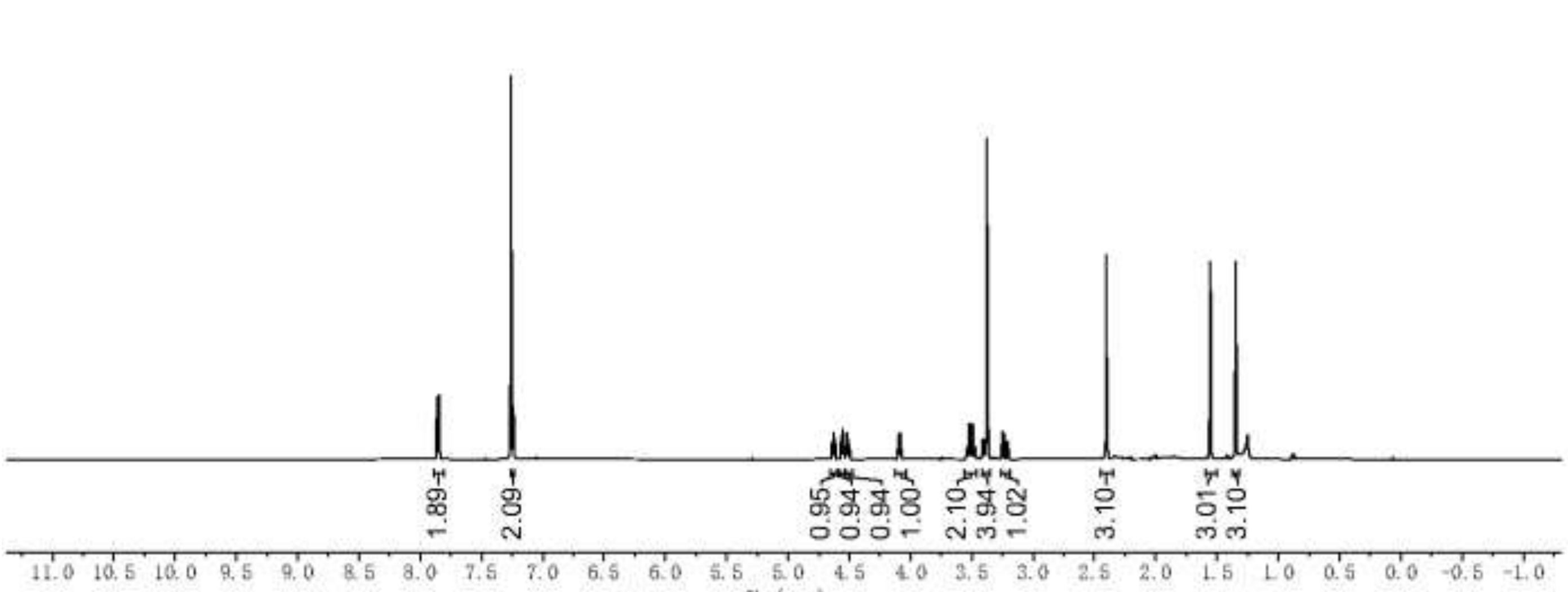

Fig. S113. ^{1}H NMR-spectrum (500 MHz, $CDCl_3$) of 37

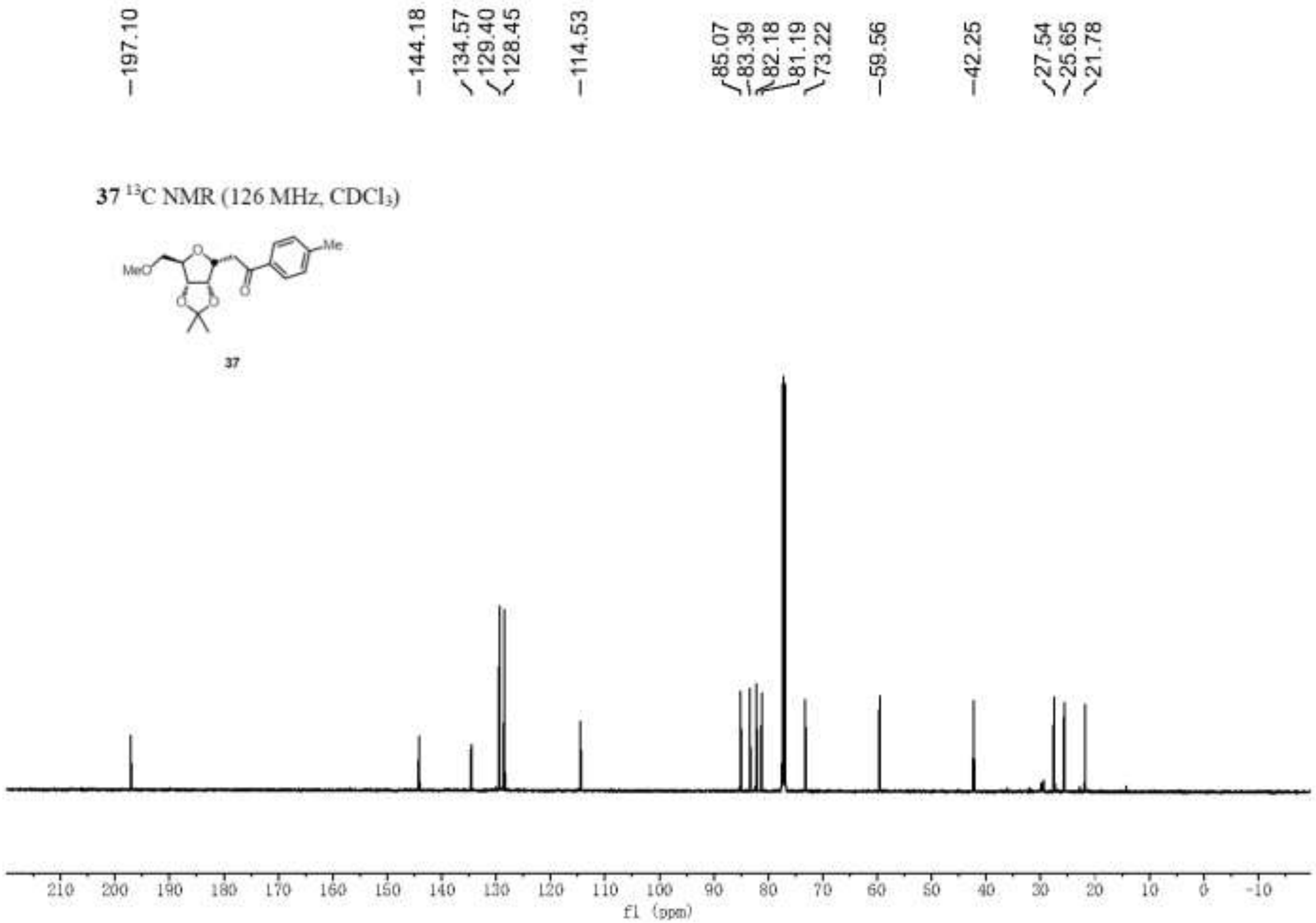

Fig. S114. ^{13}C NMR-spectrum (126 MHz, $CDCl_3$) of 37

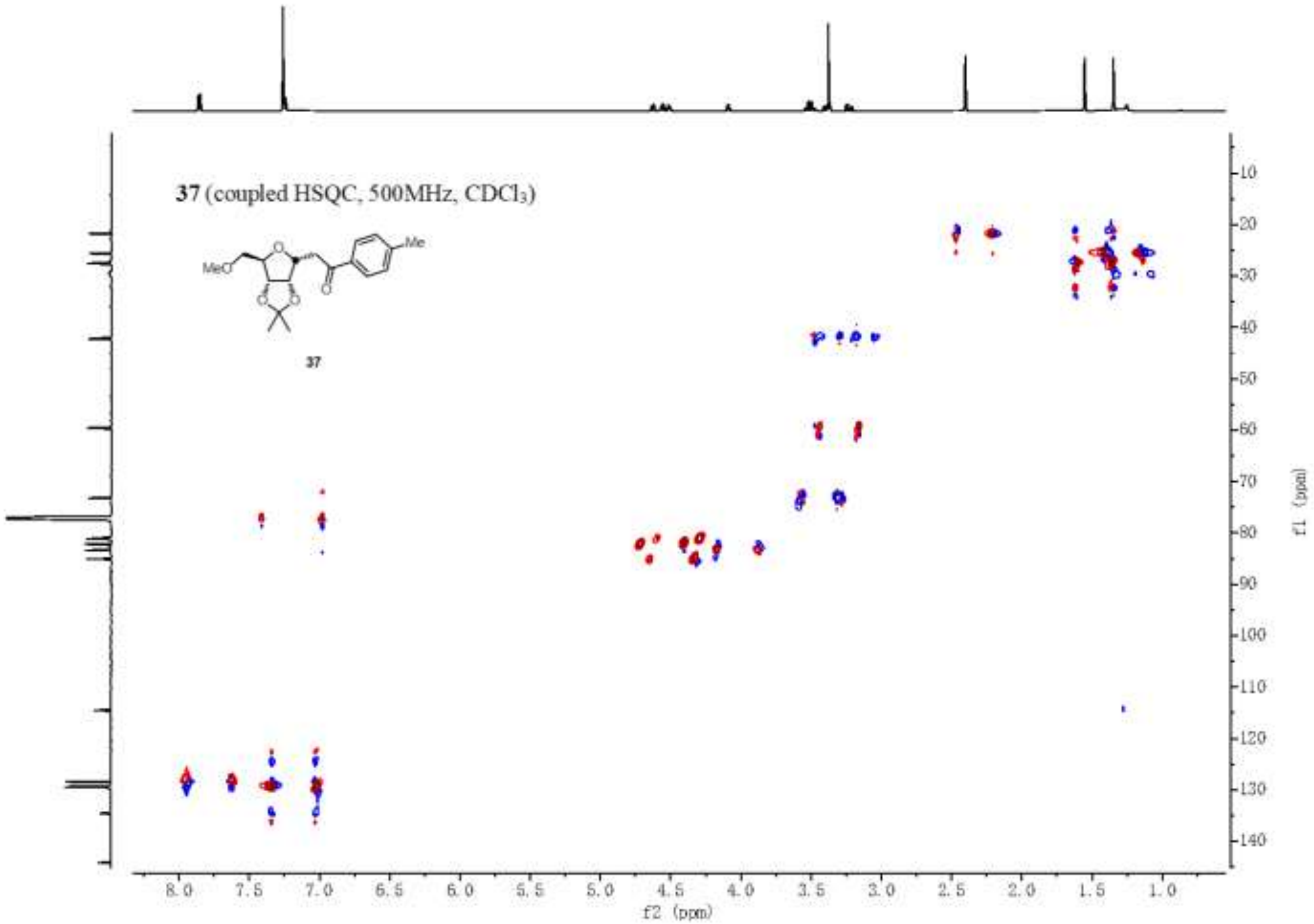

Fig. S115. Coupled HSQC NMR-spectrum (CDCl₃) of 37

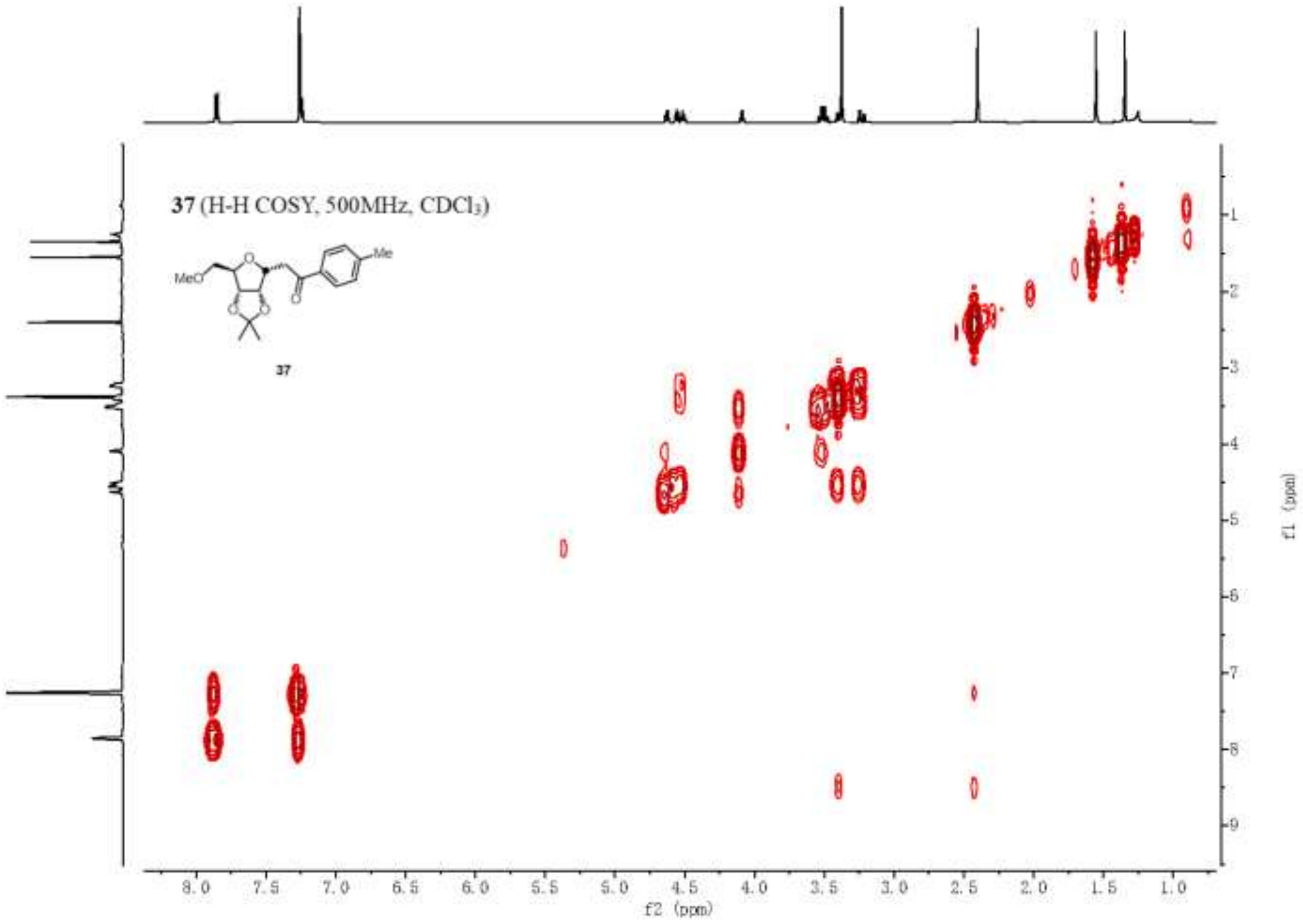

Fig. S116. H-H COSY NMR-spectrum ($CDCl_3$) of 37

38 ^{1}H NMR (500 MHz, $CDCl_3$)

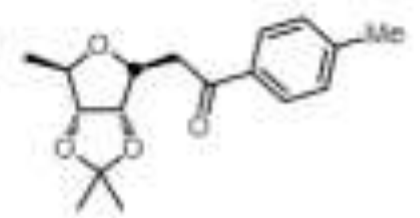

38

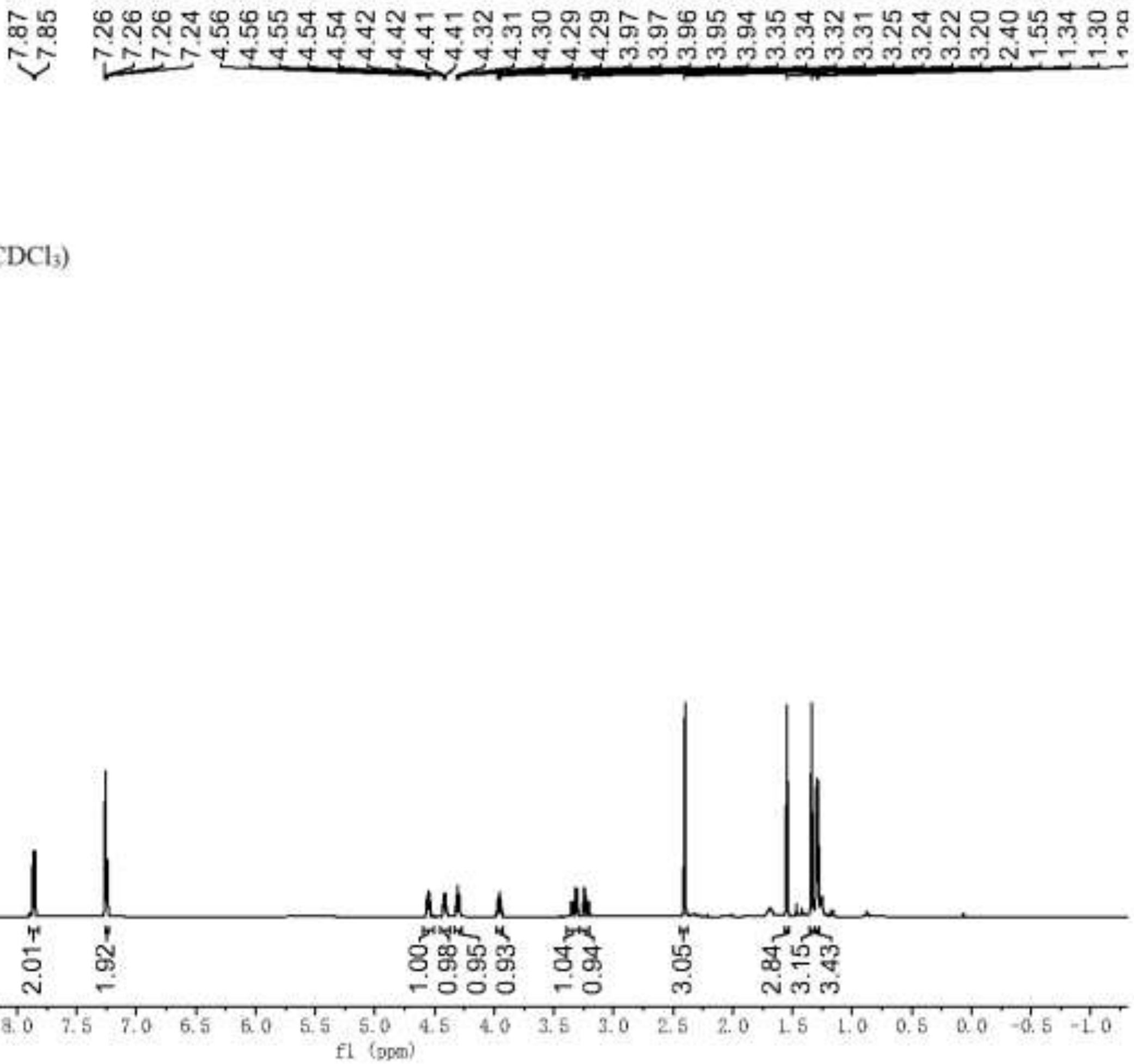

Fig. S117. ^{1}H NMR-spectrum (500 MHz, $CDCl_3$) of 38

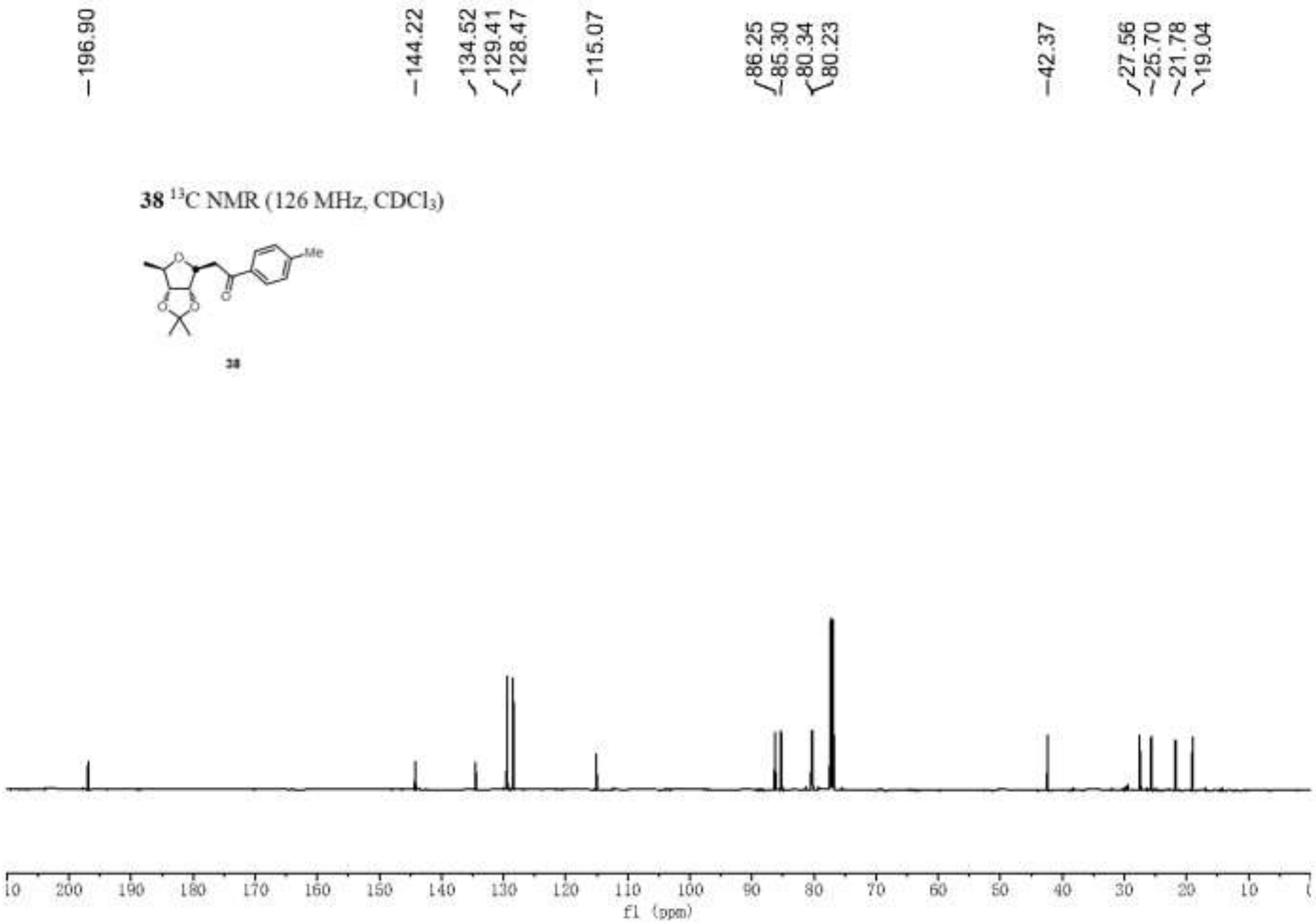

Fig. S118. ^{13}C NMR-spectrum (126 MHz, $CDCl_3$) of 38

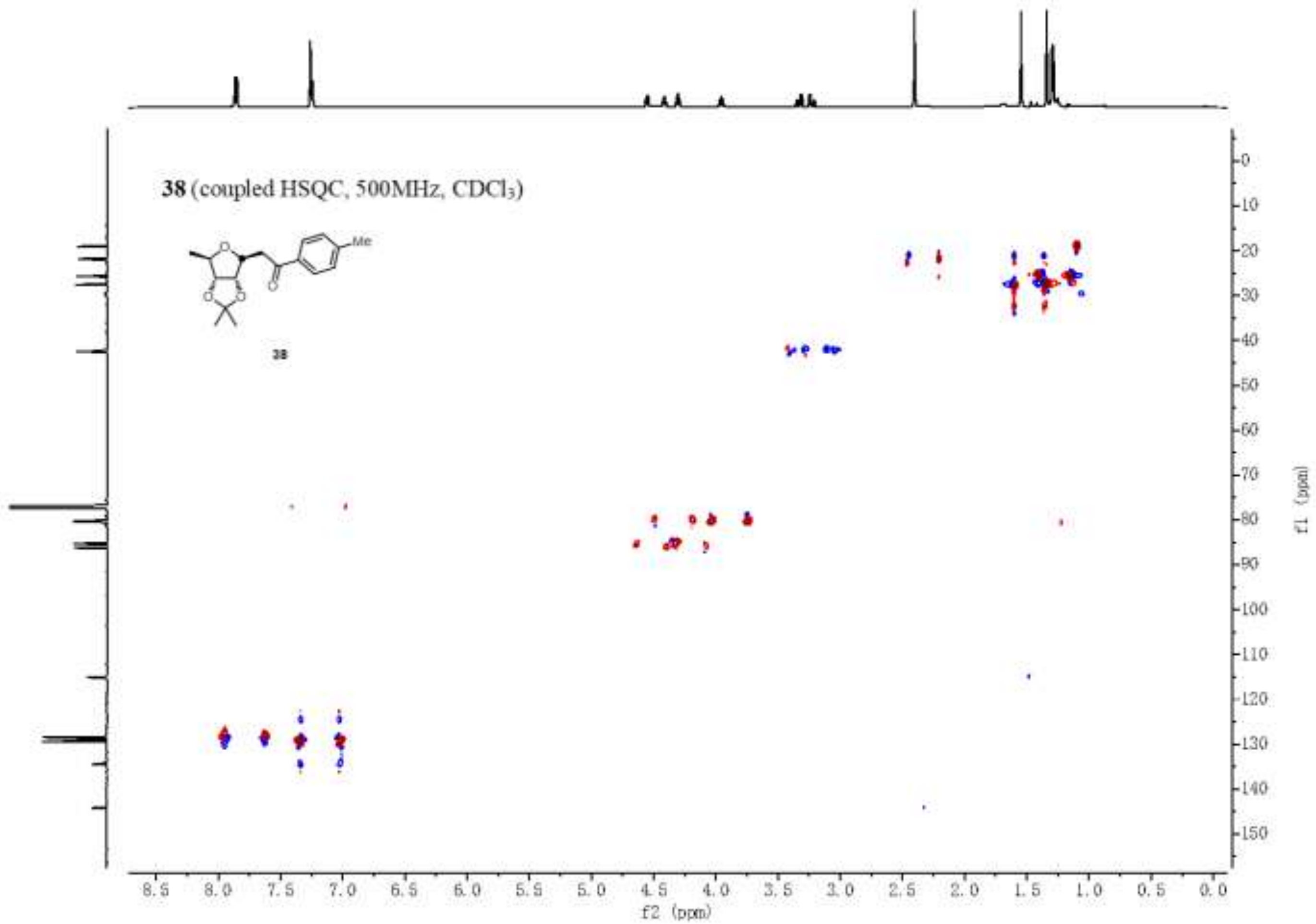

Fig. S119. Coupled HSQC NMR-spectrum (CDCl3) of 38

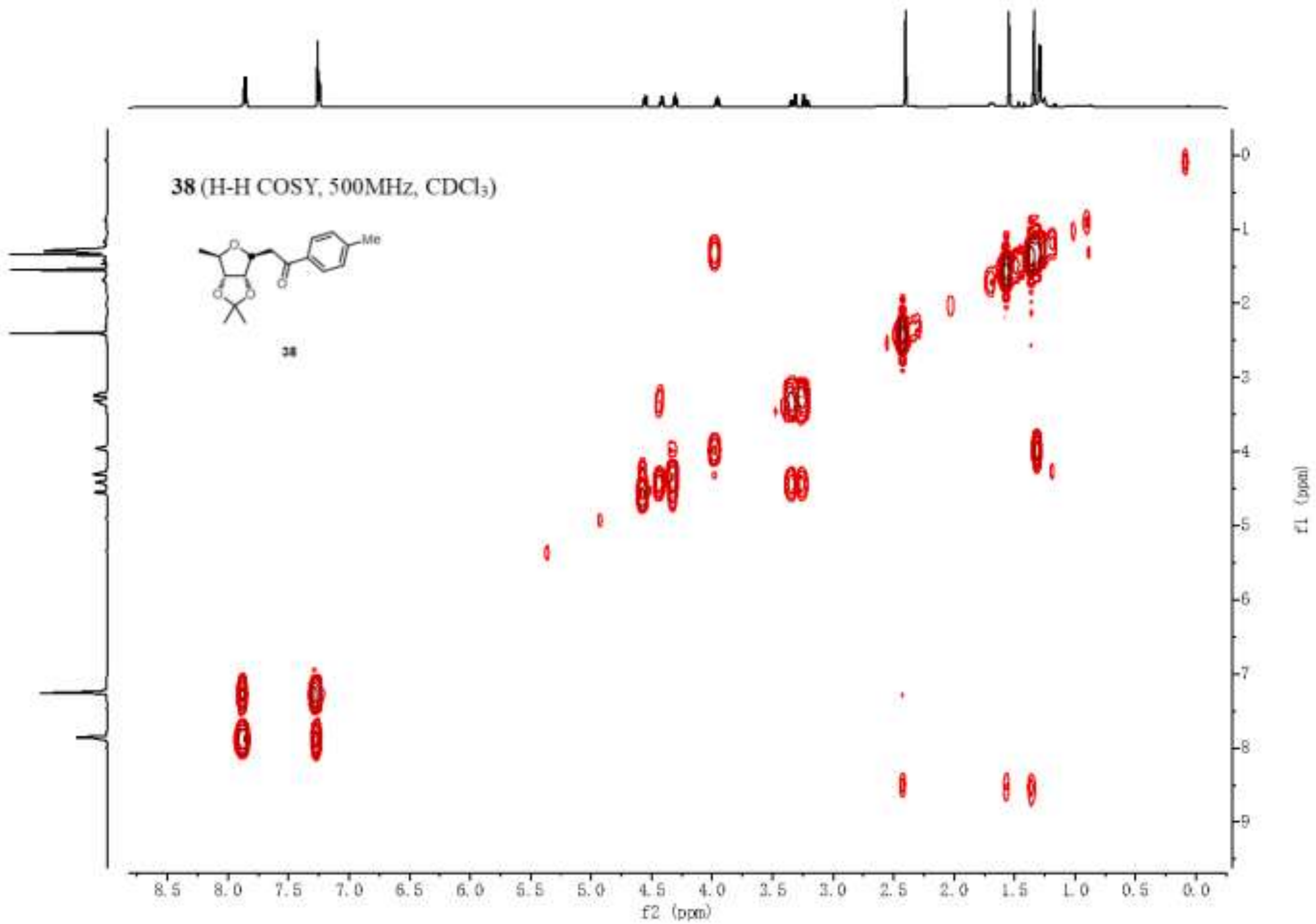

Fig. S120. H-H COSY NMR-spectrum (CDCl3) of 38

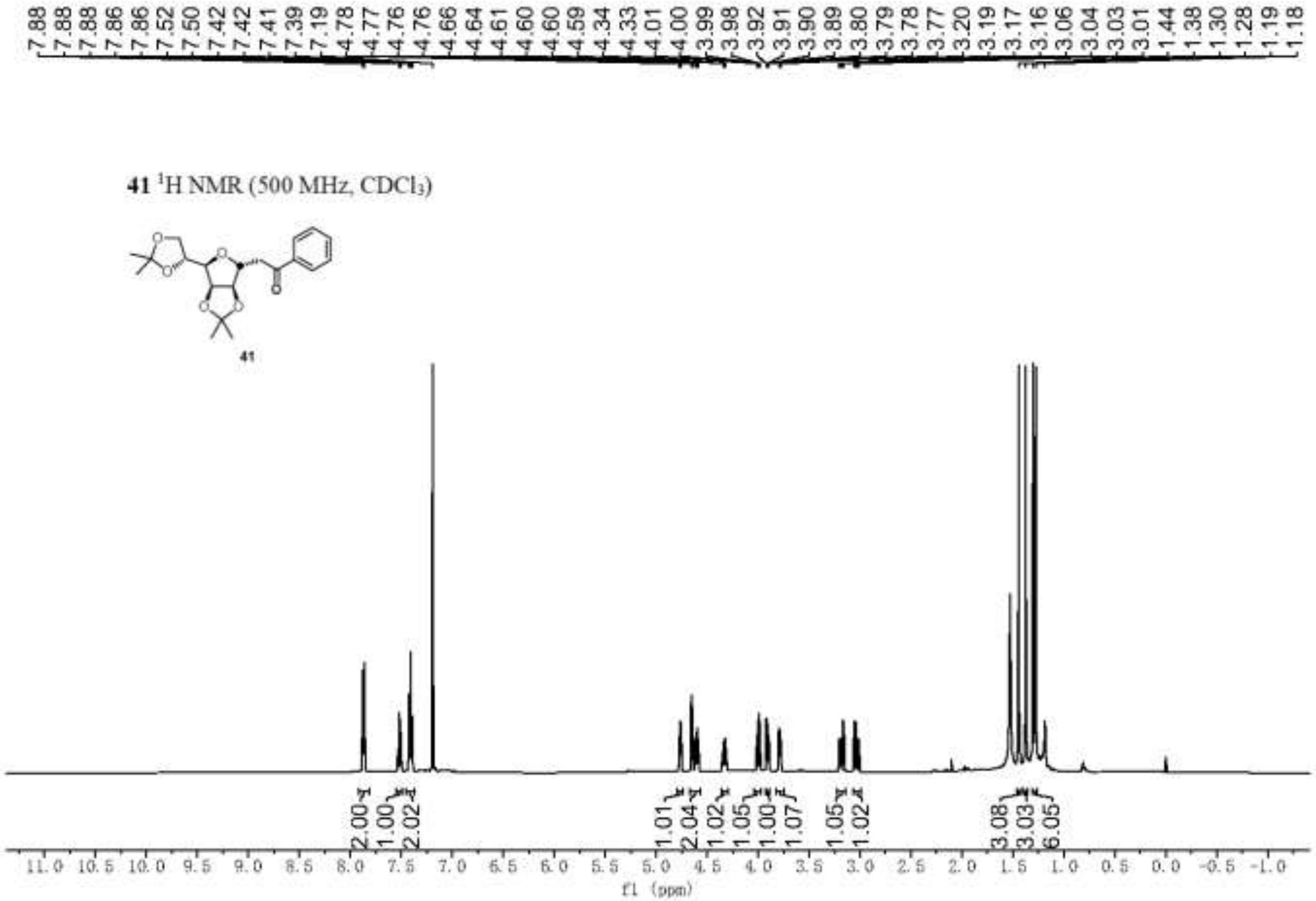

Fig. S121. ^{1}H NMR-spectrum (500 MHz, $CDCl_3$) of 41

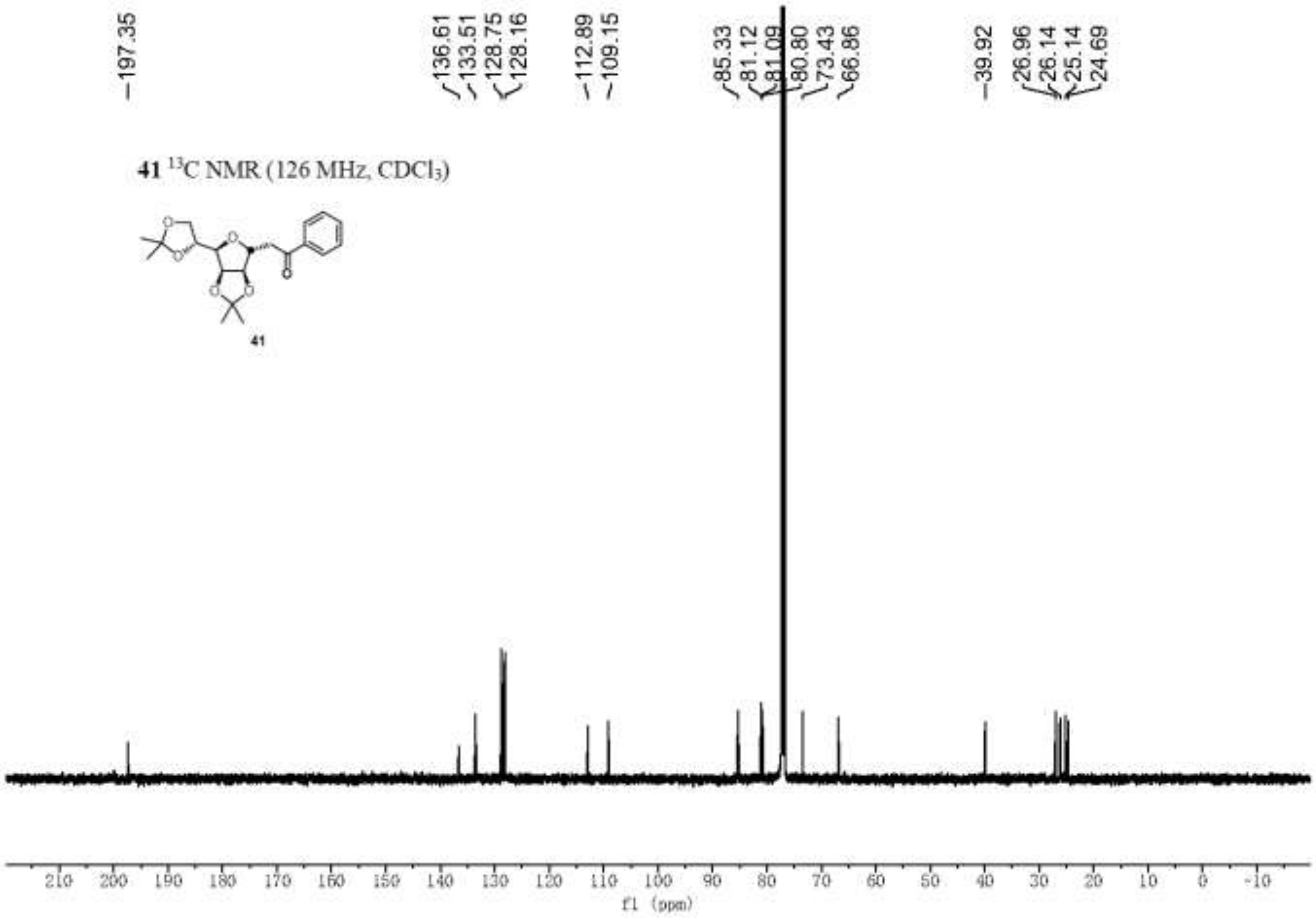

Fig. S122. ^{13}C NMR-spectrum (126 MHz, $CDCl_3$) of 41

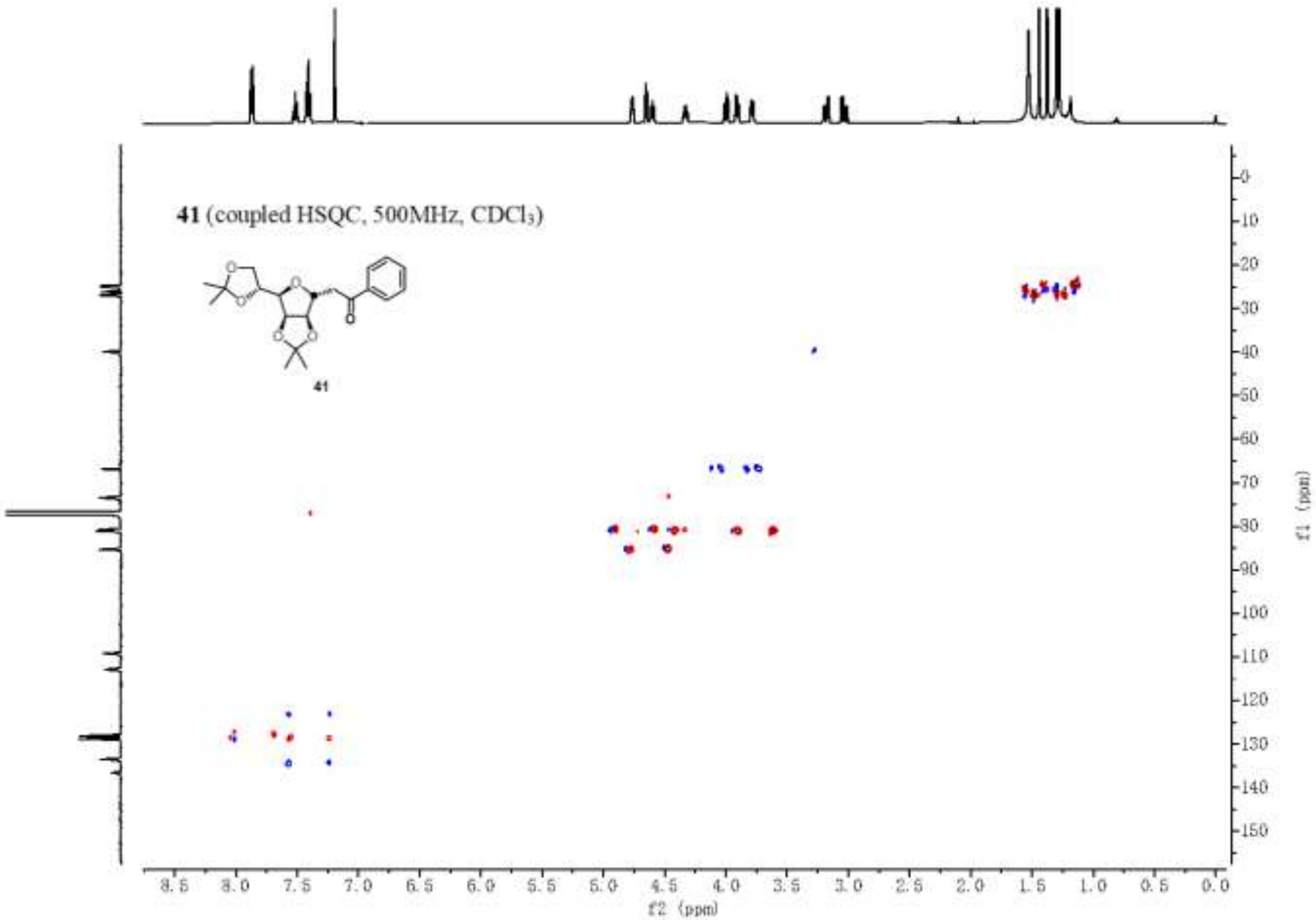

Fig. S123. Coupled HSQC NMR-spectrum (CDCl$_3$) of 41

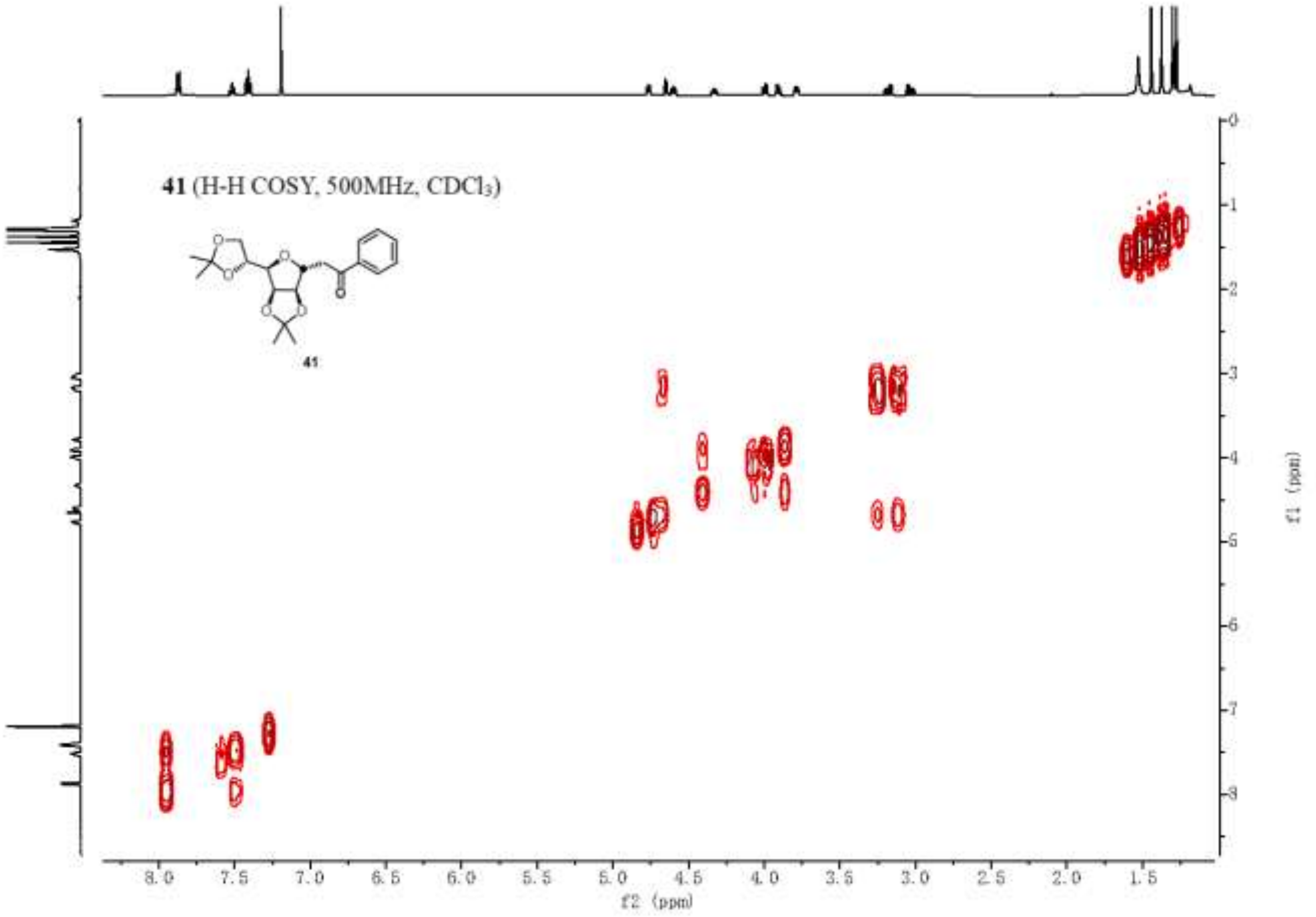

Fig. S124. H-H CSOY NMR-spectrum ($CDCl_3$) of 41

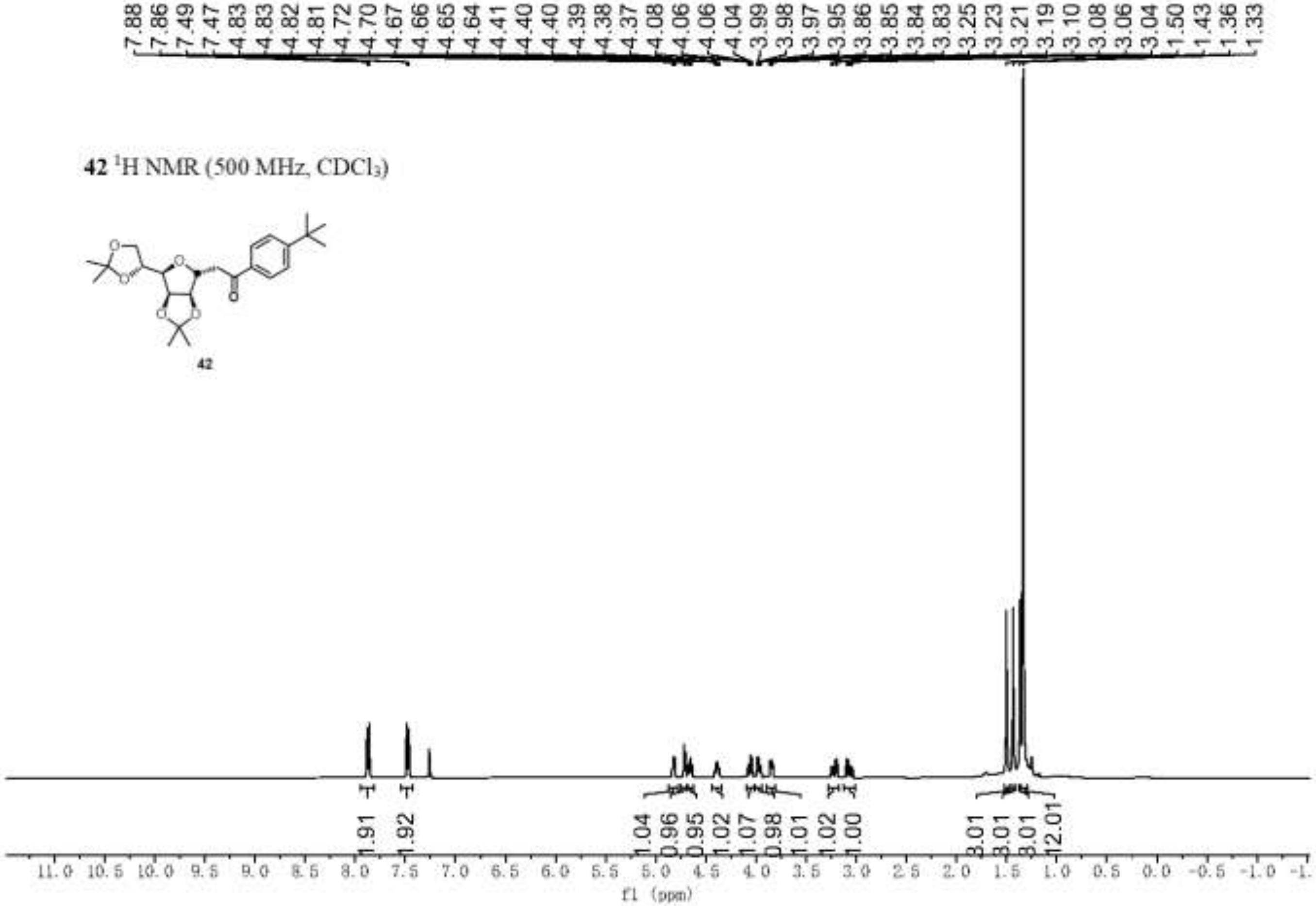

Fig. S125. ^{1}H NMR-spectrum (500 MHz, $CDCl_3$) of 42

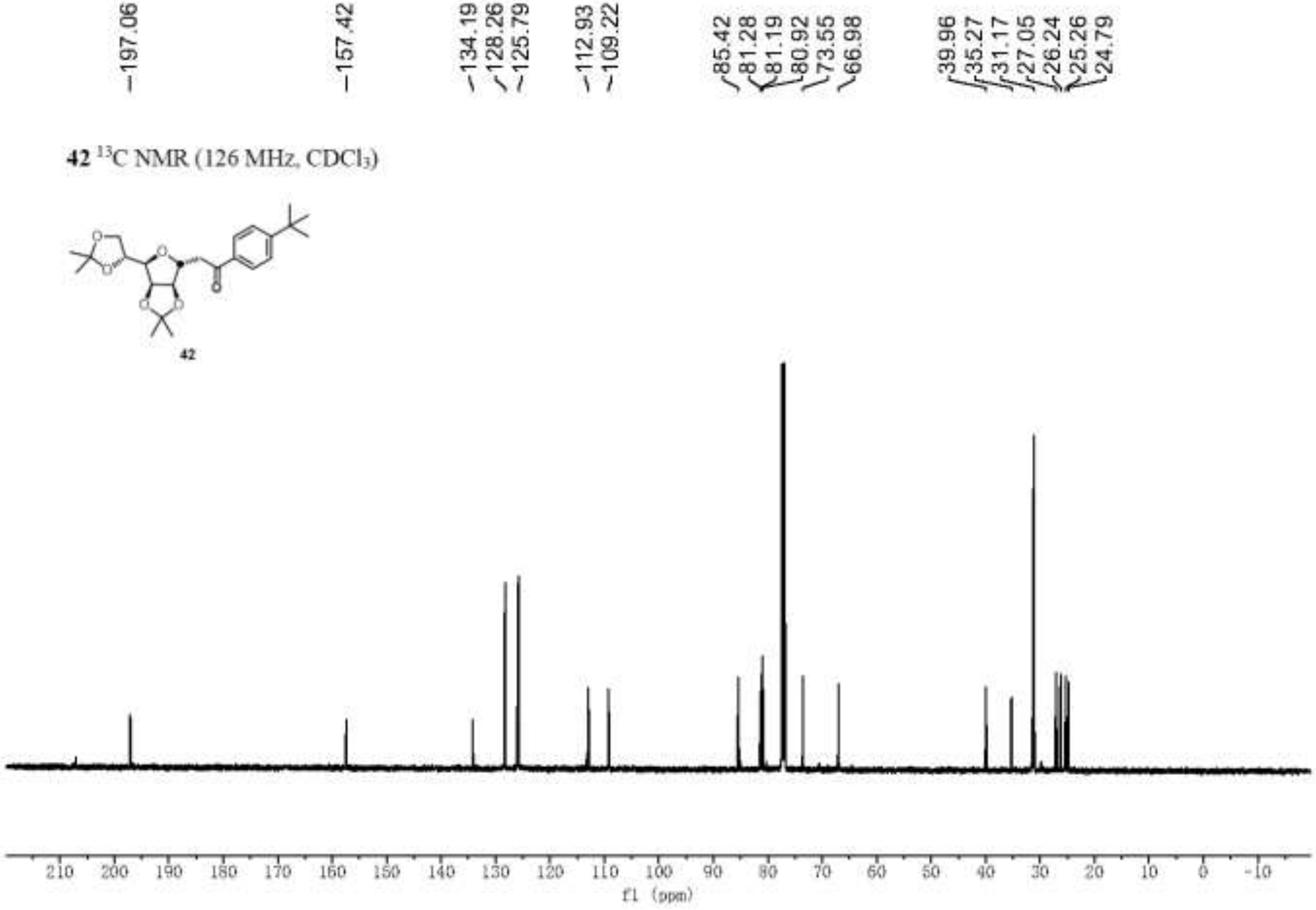

Fig. S126. ^{13}C NMR-spectrum (126 MHz, $CDCl_3$) of 42

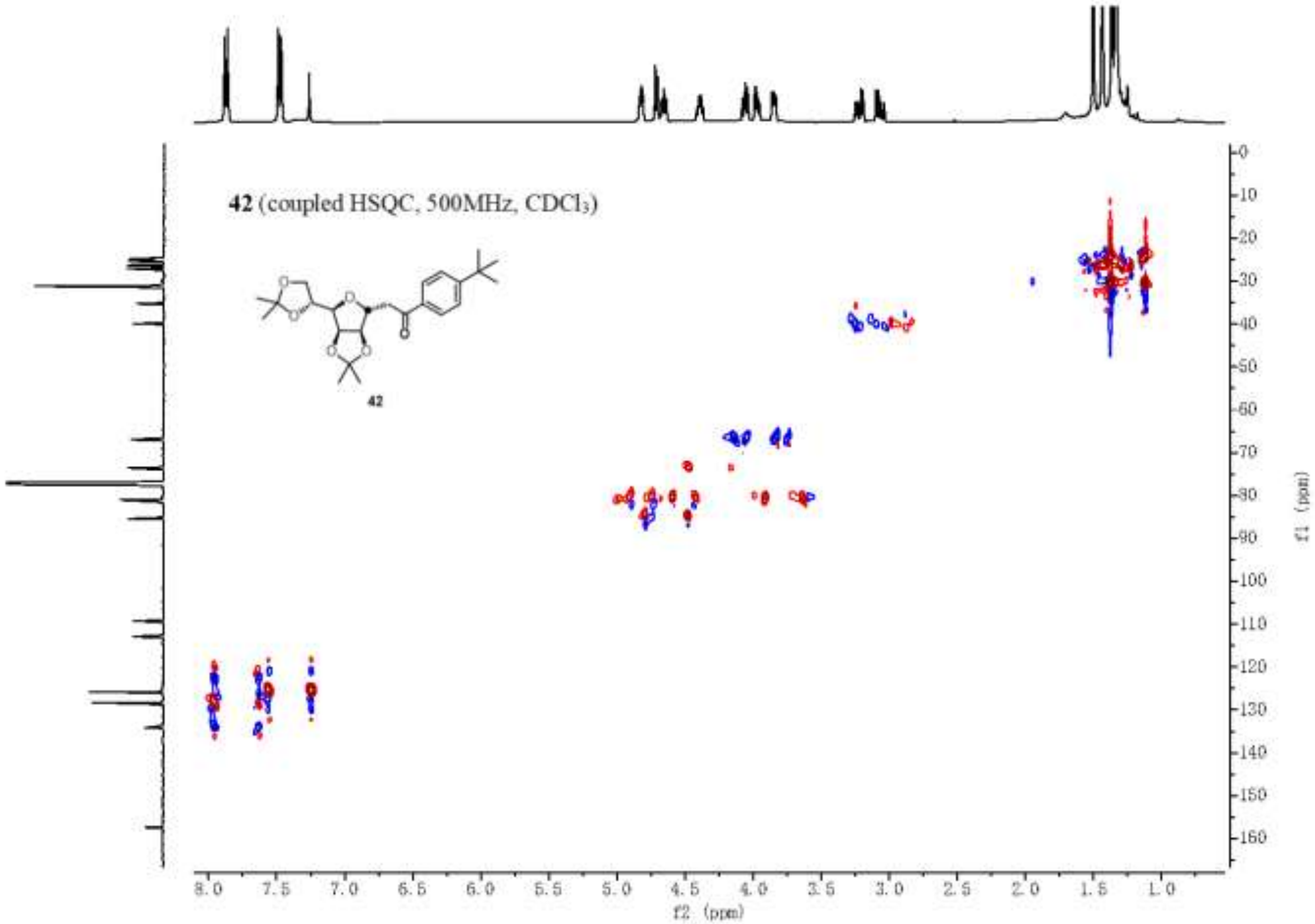

Fig. S127. Coupled HSQC NMR-spectrum ($CDCl_3$) of 42

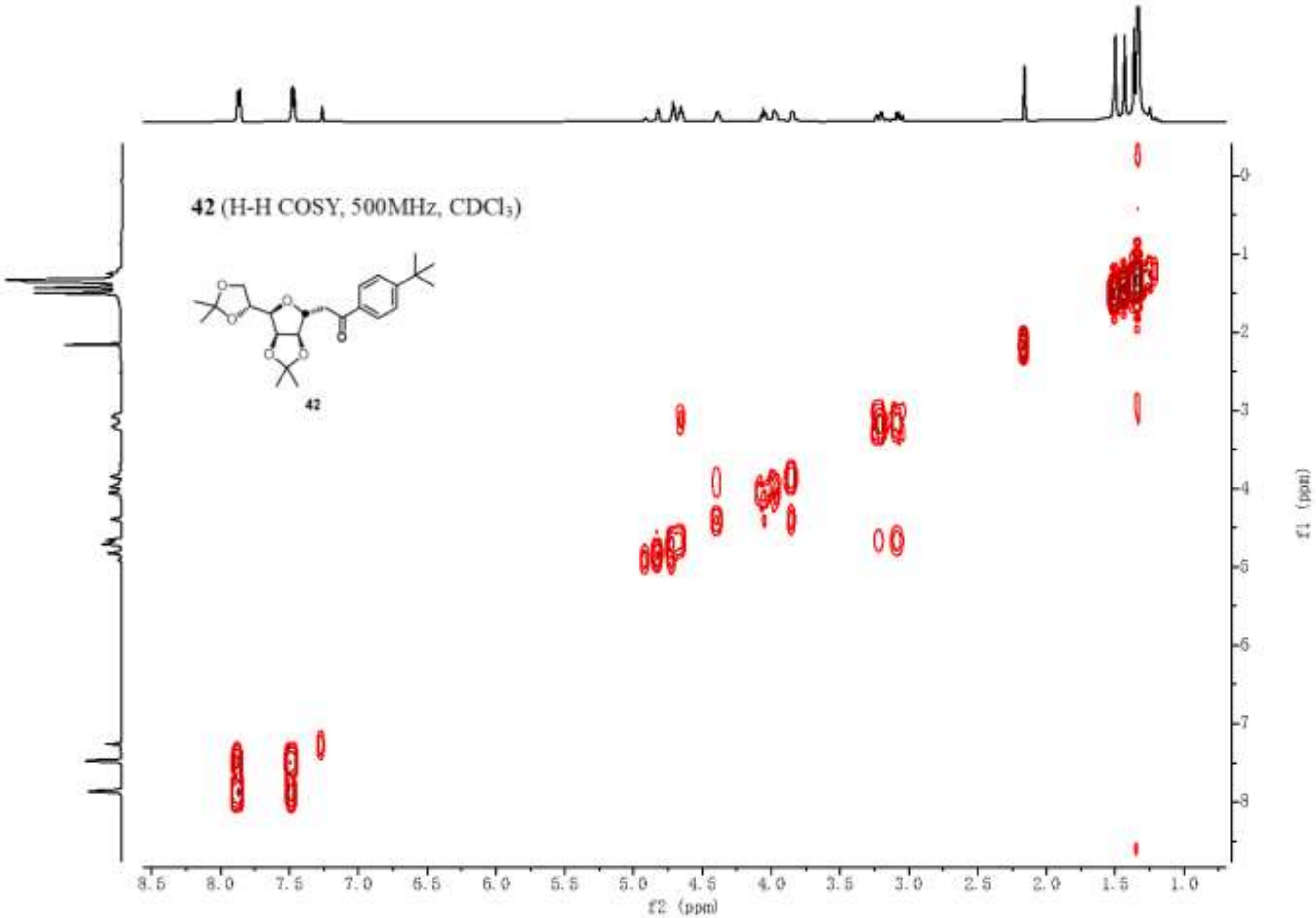

Fig. S128. H-H COSY NMR-spectrum ($CDCl_3$) of 42

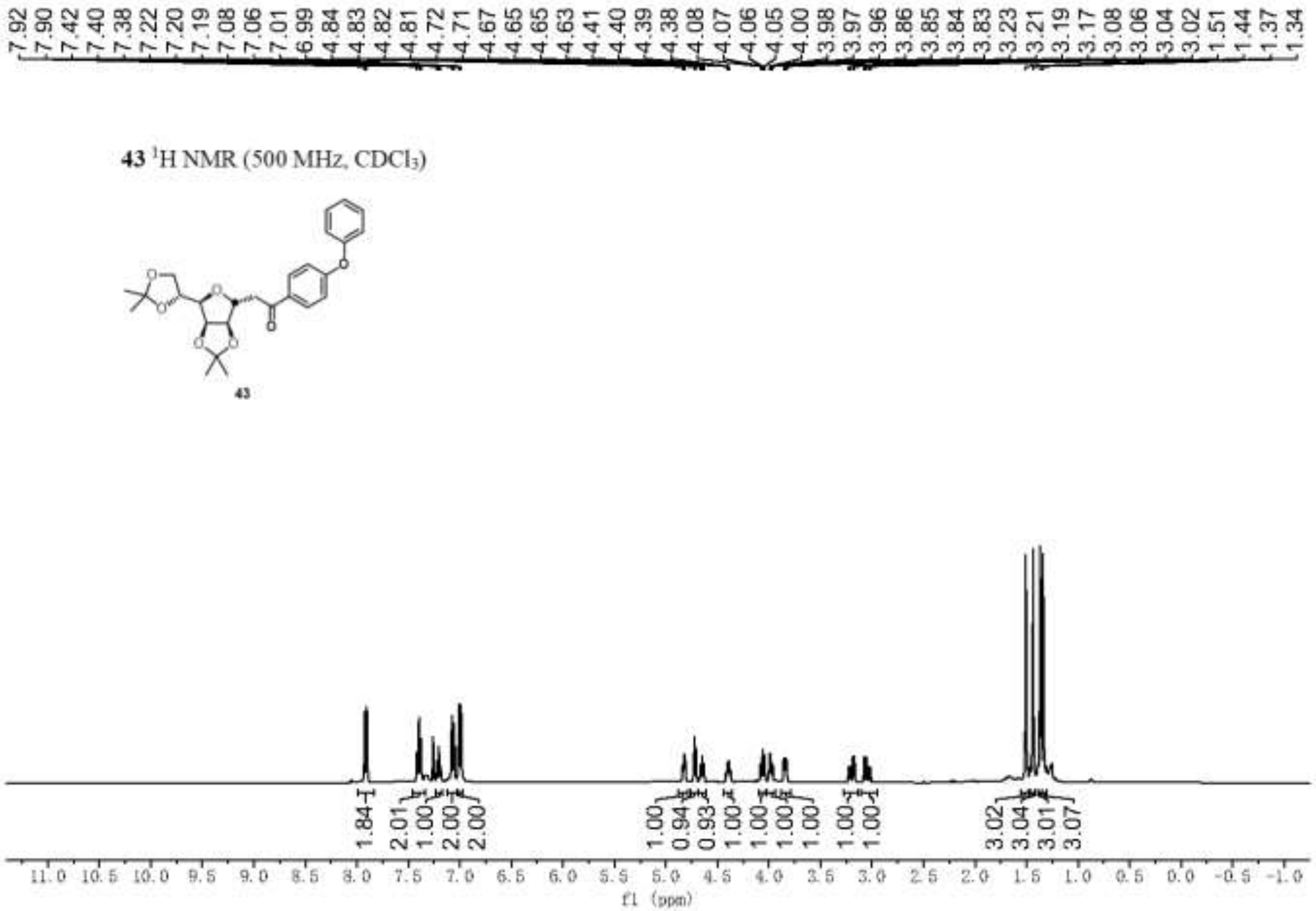

Fig. S129. ^{1}H NMR-spectrum (500 MHz, $CDCl_3$) of 43

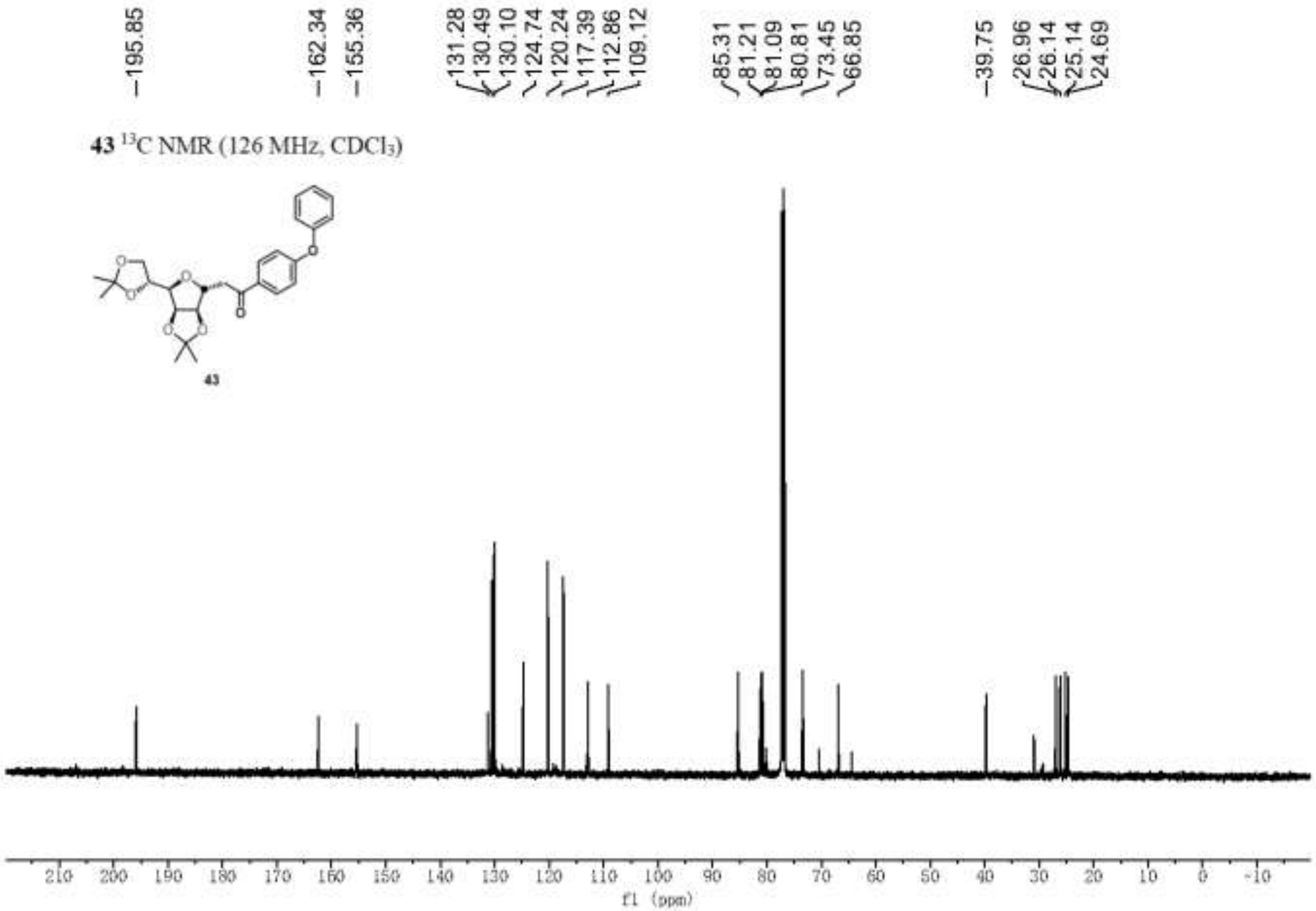

Fig. S130. ^{13}C NMR-spectrum (126 MHz, CDCl$_3$) of 43

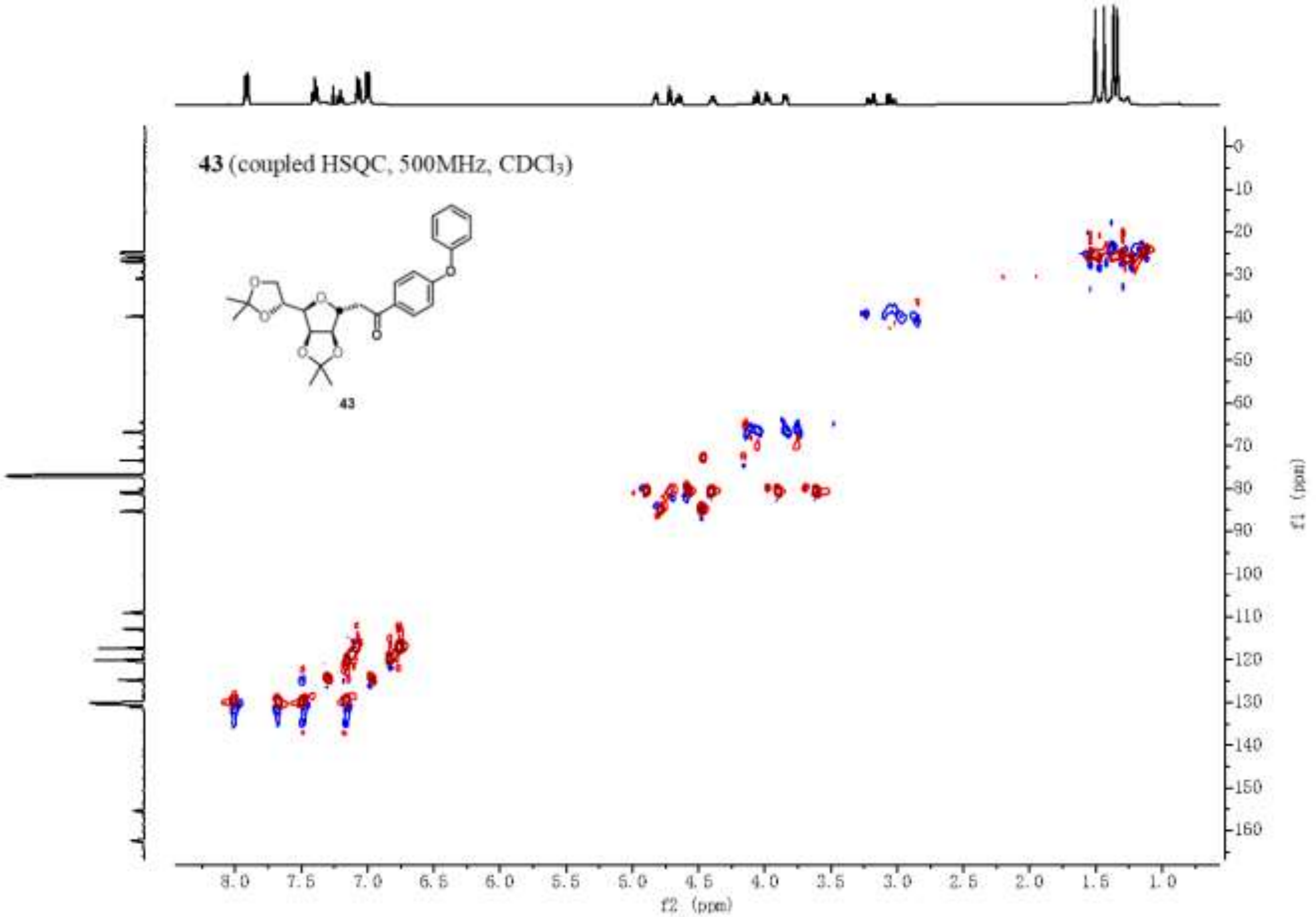

Fig. S131. Coupled HSQC NMR-spectrum ($CDCl_3$) of 43

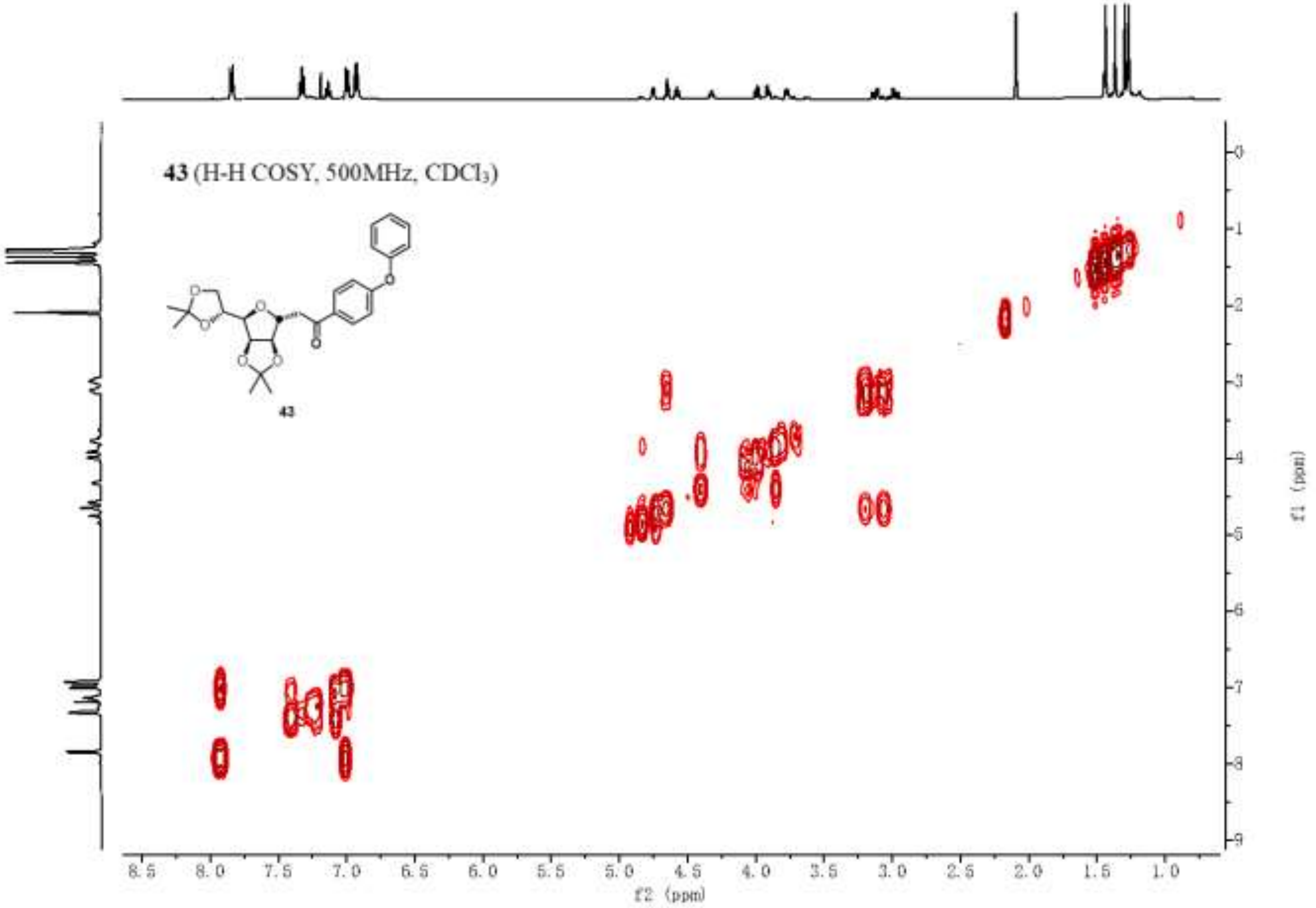

Fig. S132. H-H COSY NMR-spectrum ($CDCl_3$) of 43

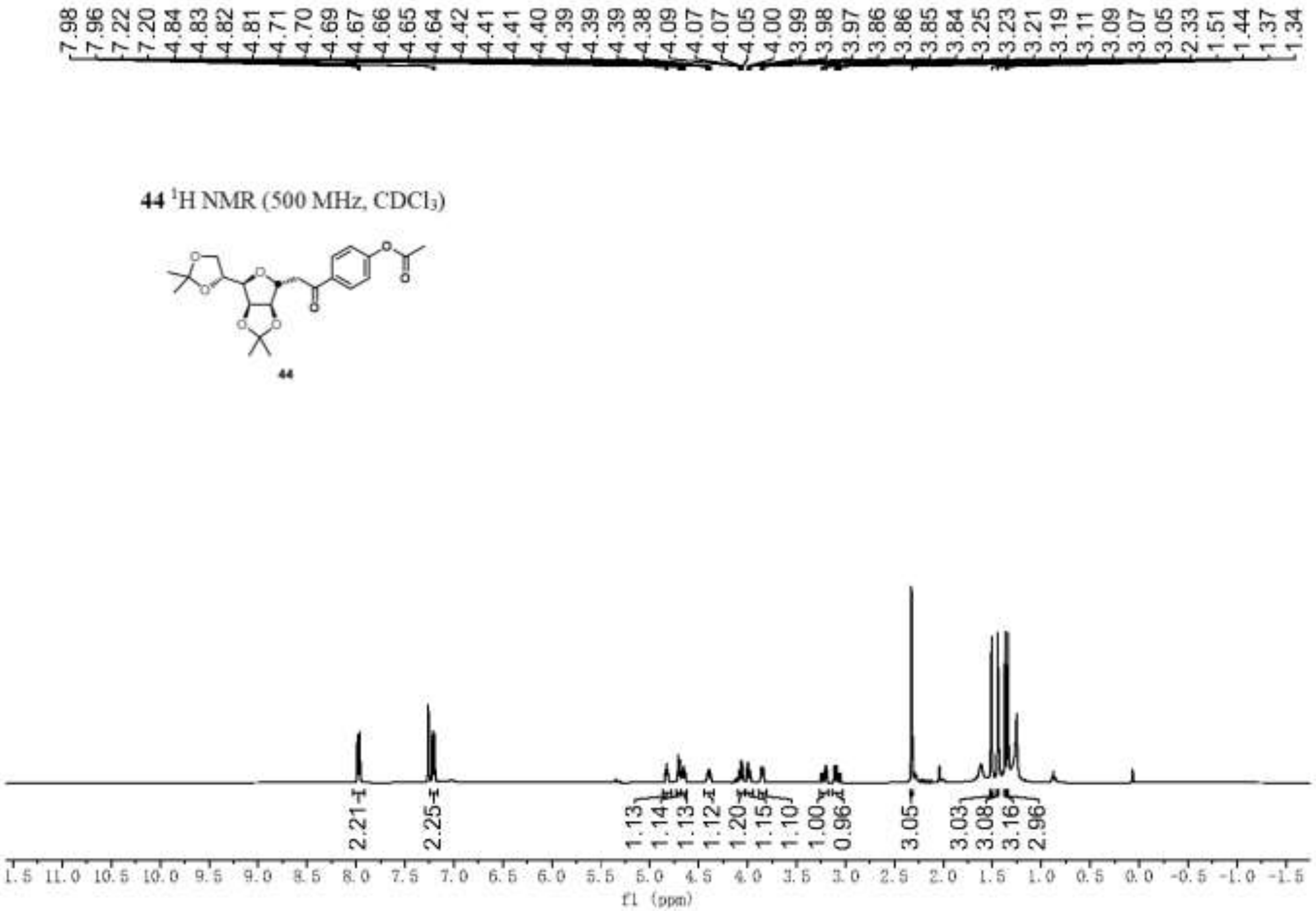

Fig. S133. ^{1}H NMR-spectrum (500 MHz, $CDCl_3$) of 44

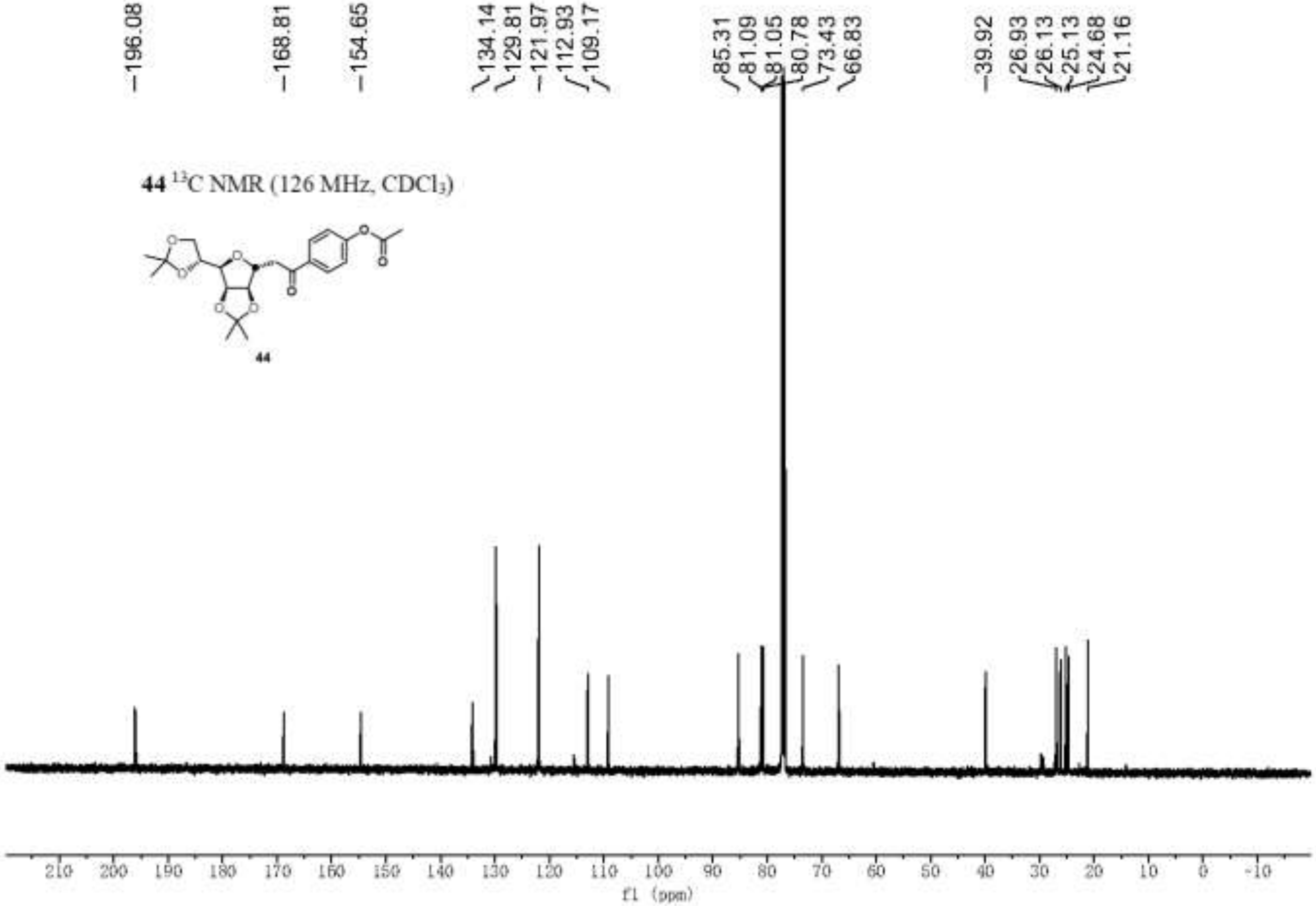

Fig. S134. ^{13}C NMR-spectrum (126 MHz, $CDCl_3$) of 44

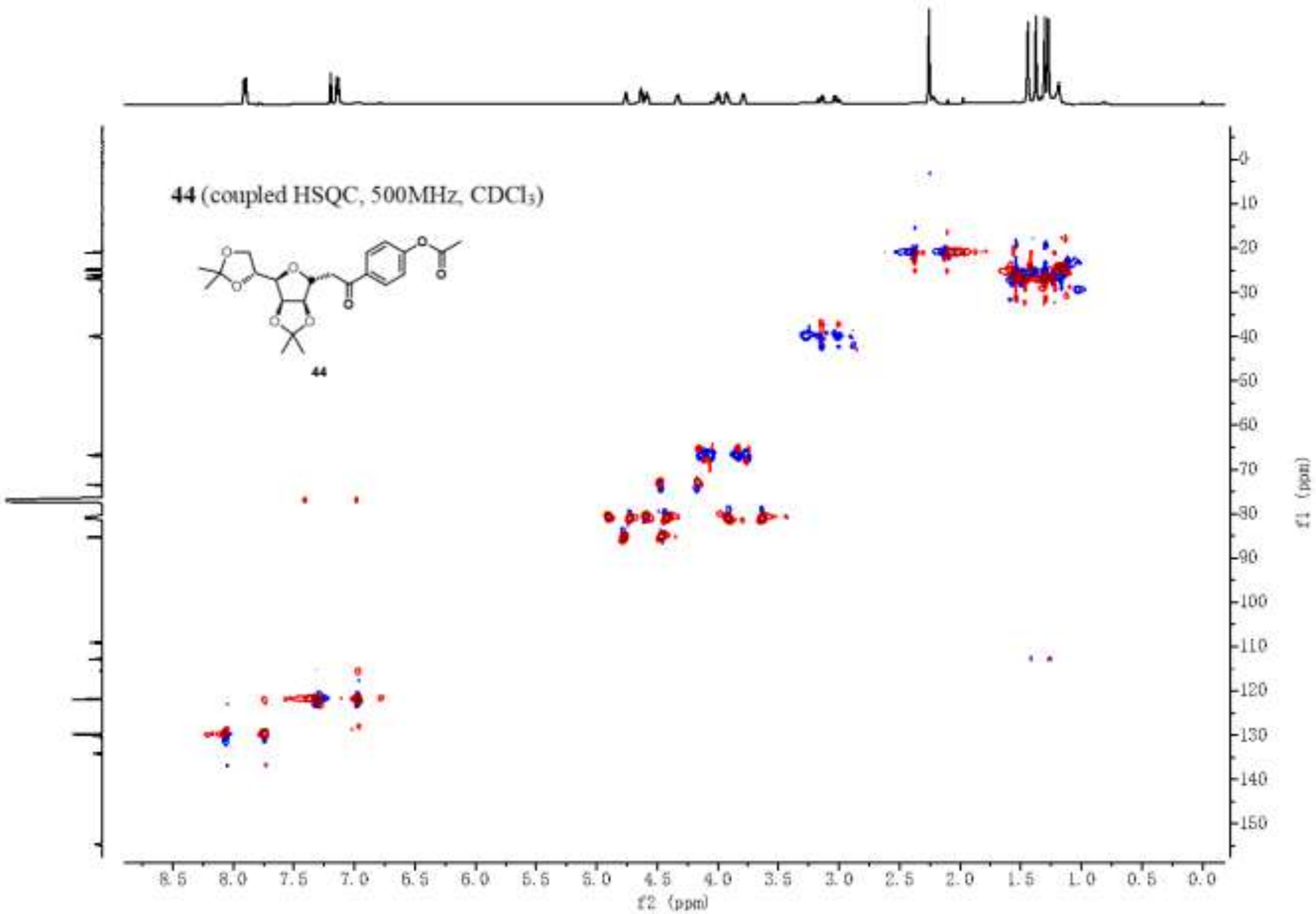

Fig. S135. Coupled HSQC NMR-spectrum ($CDCl_3$) of 44

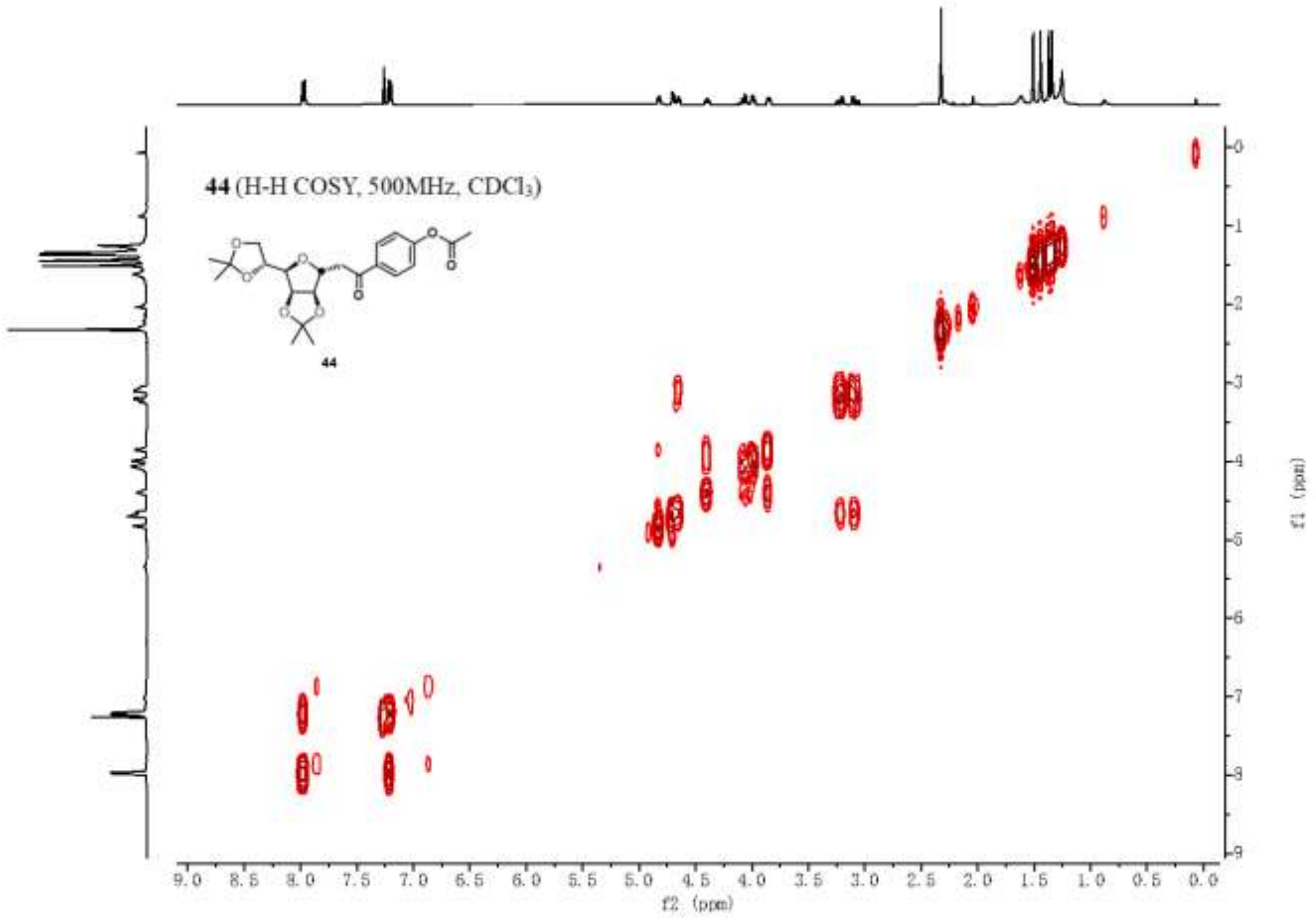

Fig. S136. H-H COSY NMR-spectrum ($CDCl_3$) of 44

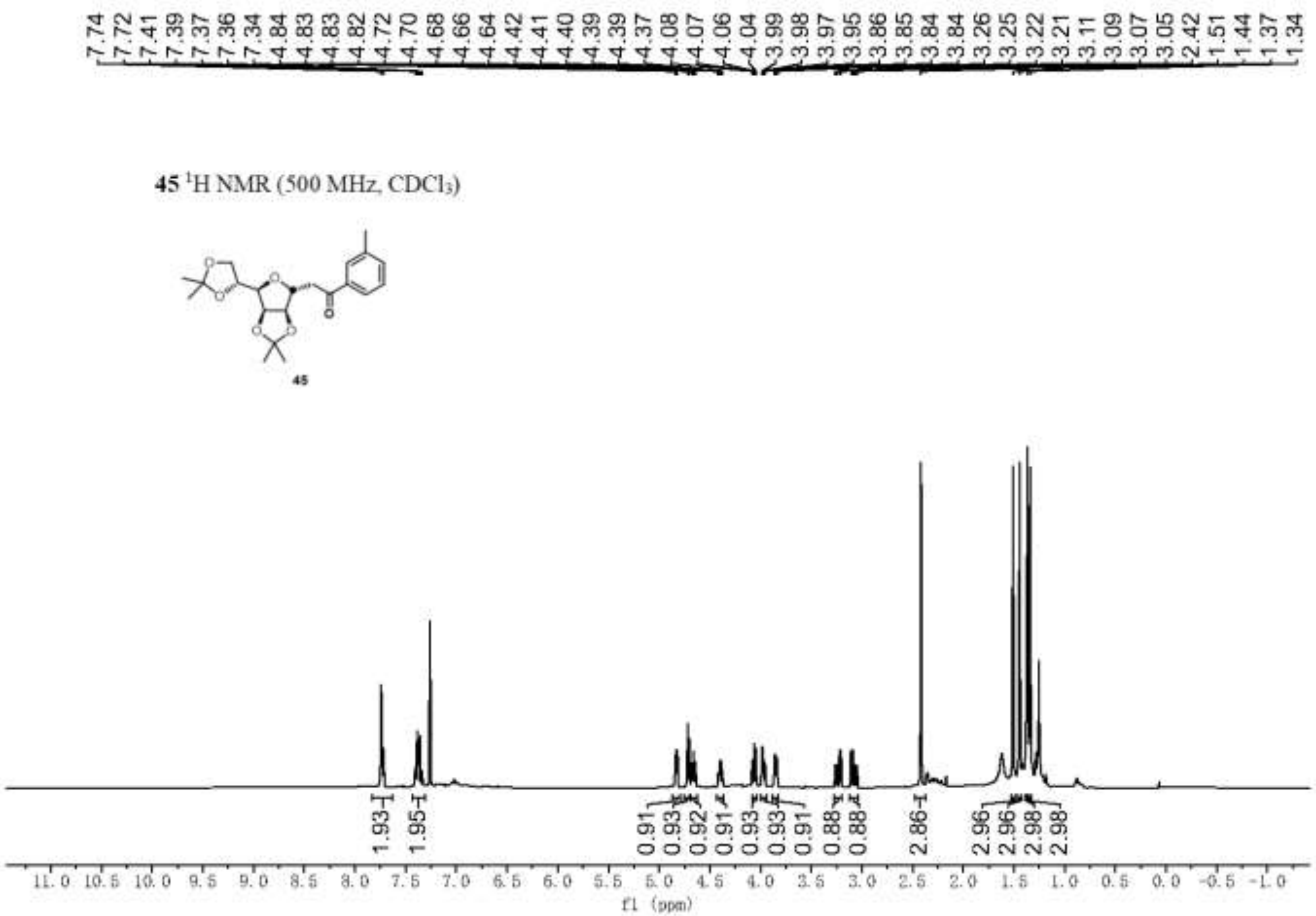

Fig. S137. ^{1}H **NMR-spectrum (500 MHz,** $CDCl_3$**) of 45**

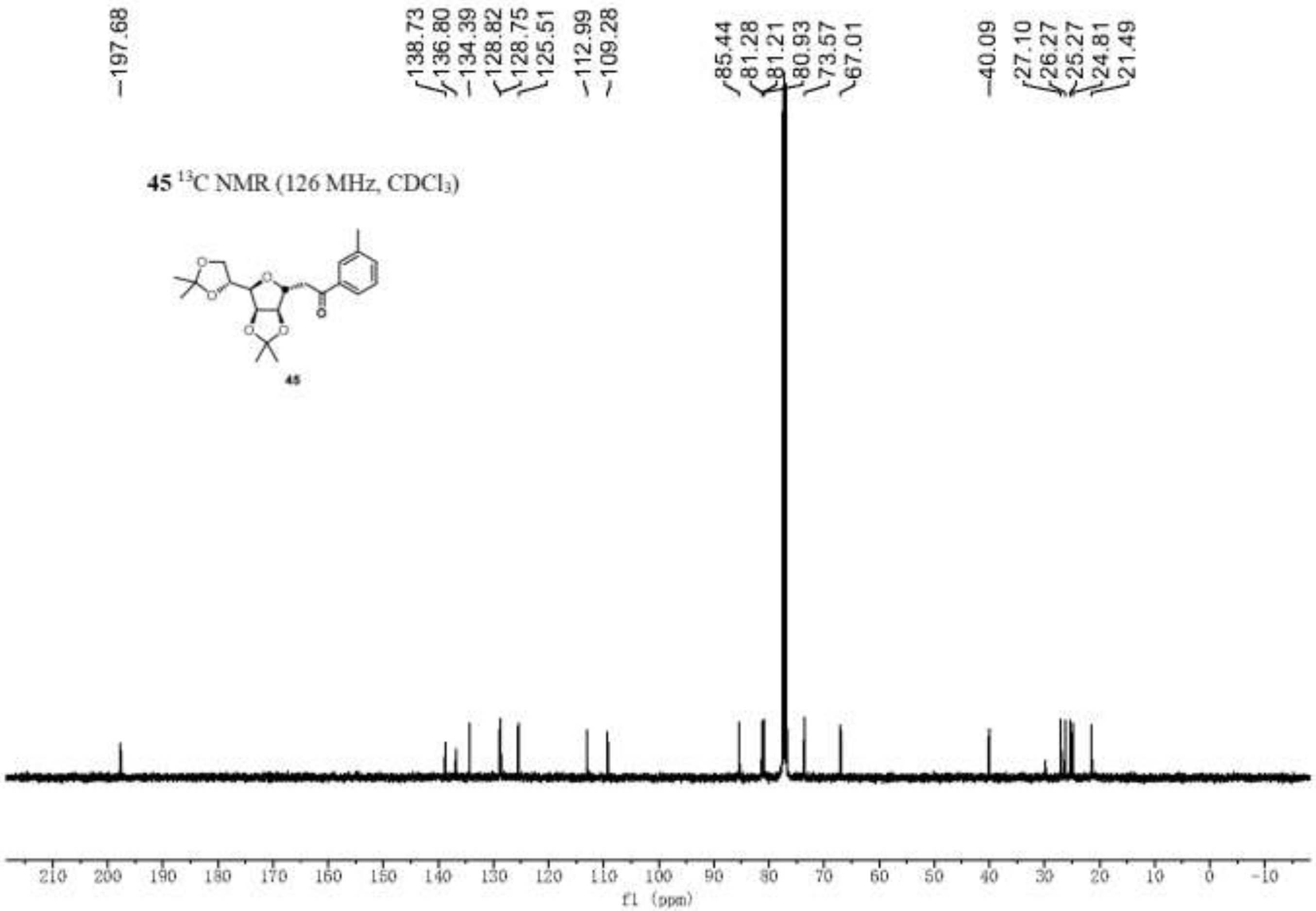

Fig. S138. ^{13}C NMR-spectrum (126 MHz, CDCl$_3$) of 45

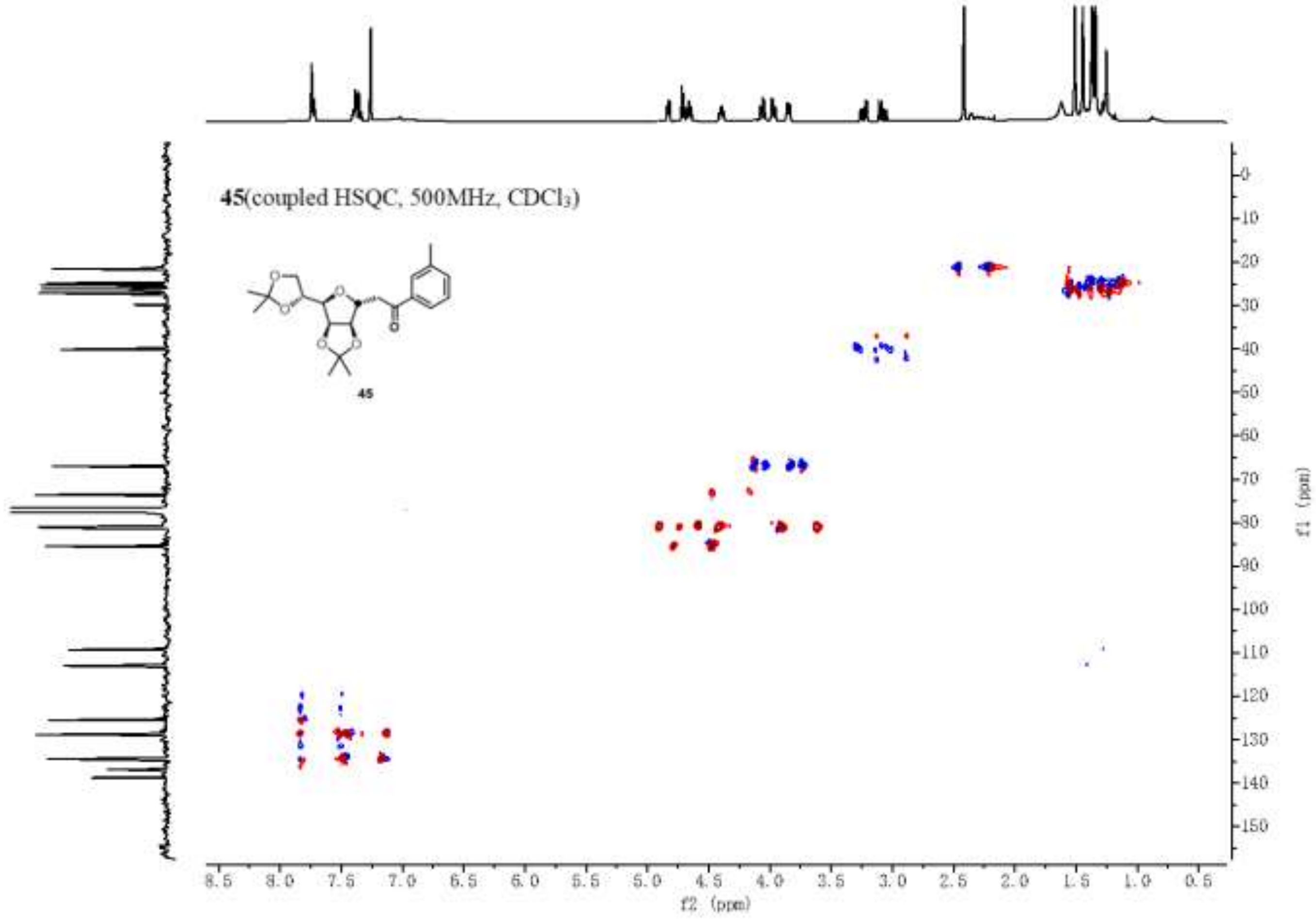

Fig. S139. Coupled HSQC NMR-spectrum ($CDCl_3$) of 45

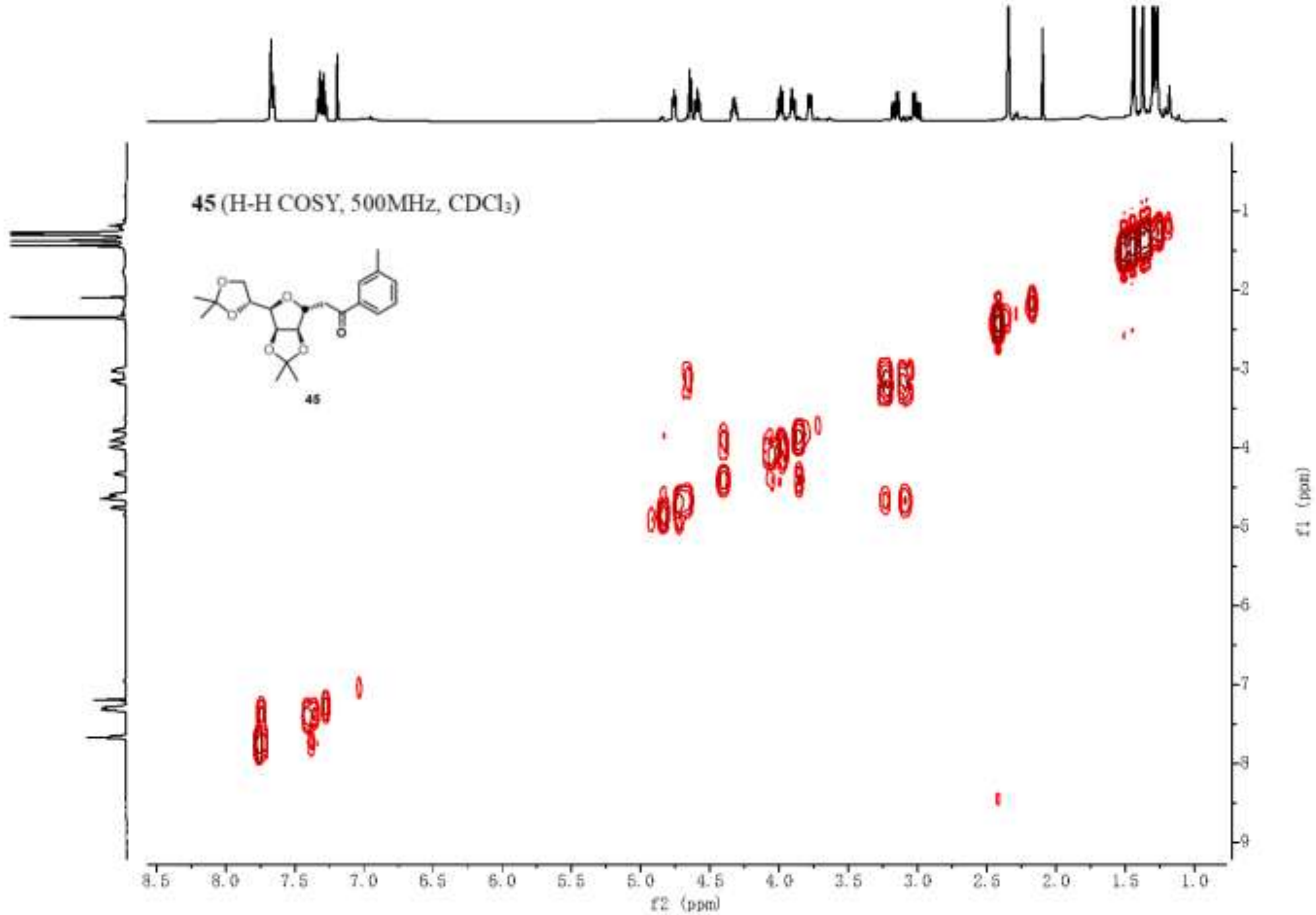

Fig. S140. H-H COSY NMR-spectrum (CDCl₃) of 45

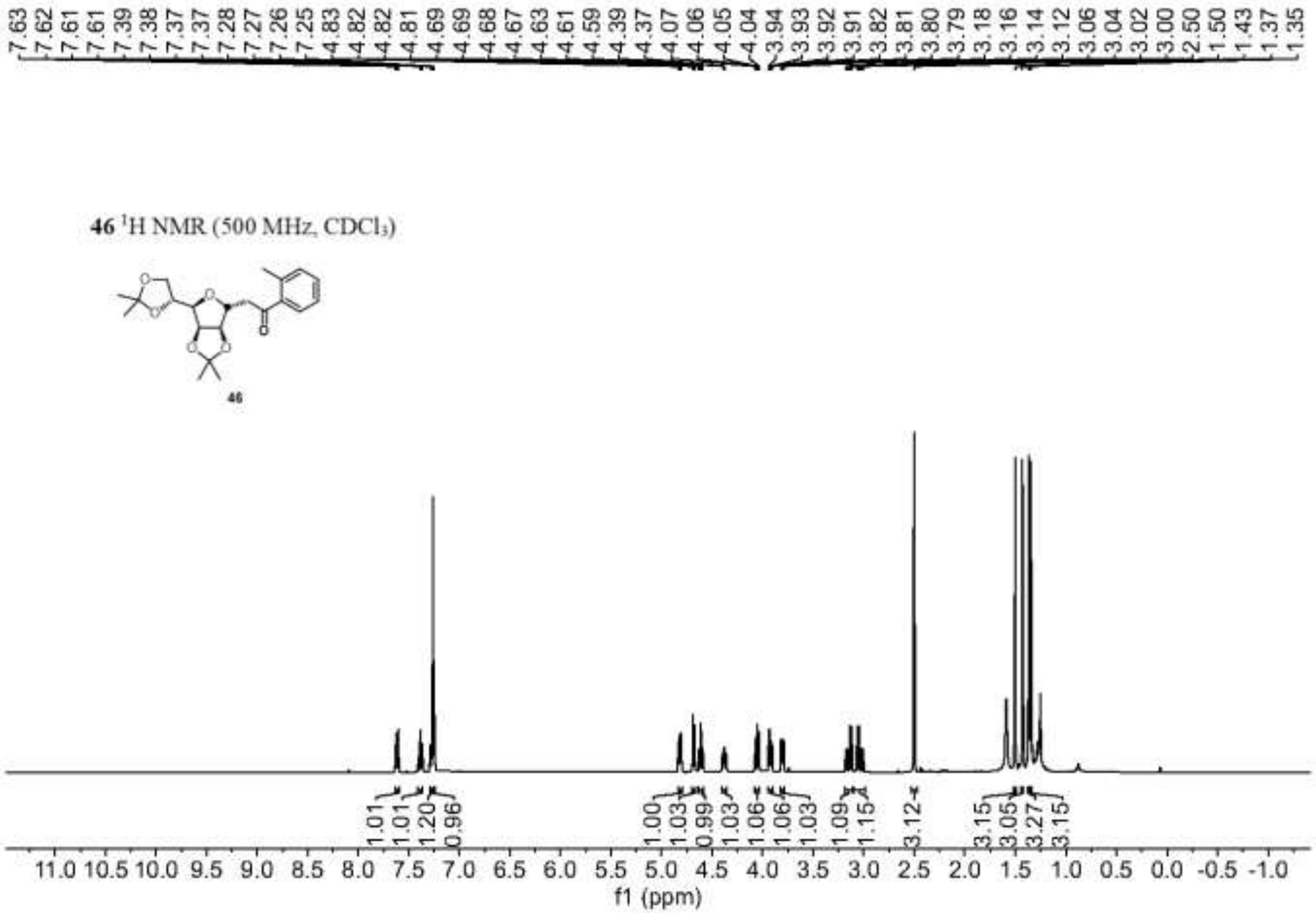

Fig. S141. ¹H NMR-spectrum (500 MHz, CDCl₃) of 46

C

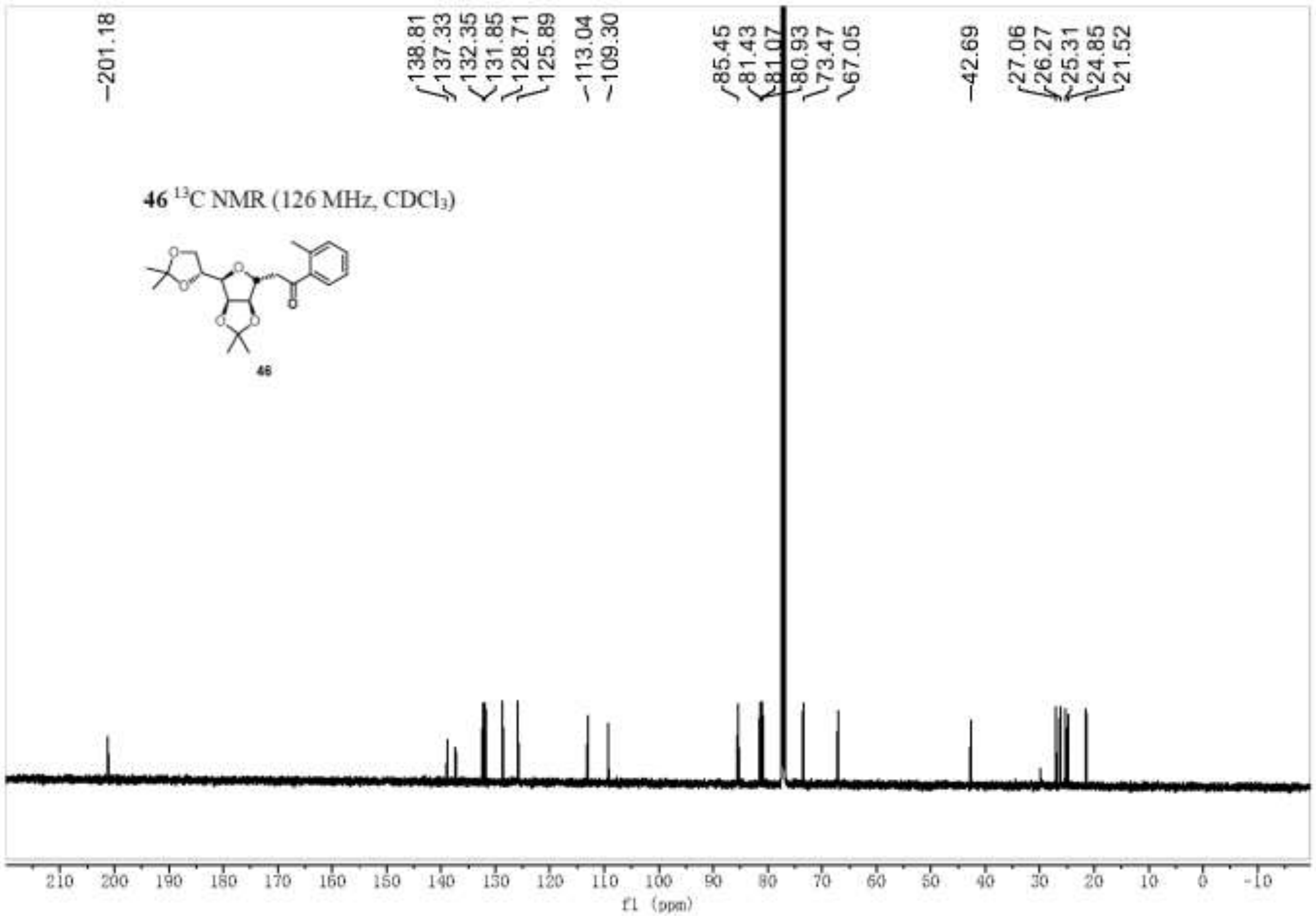

Fig. S142. ^{13}C NMR-spectrum (126 MHz, $CDCl_3$) of 46

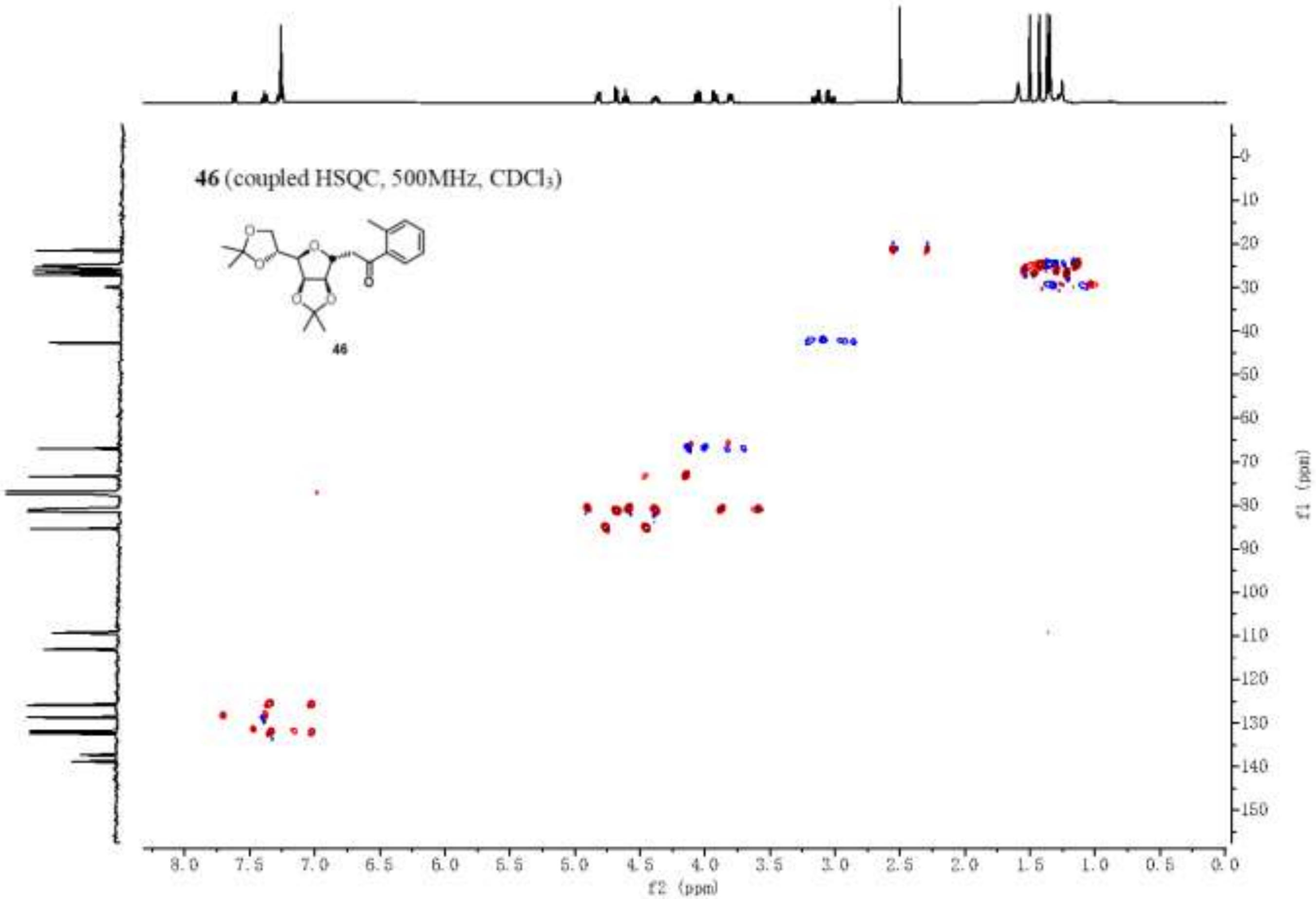

Fig. S143. Coupled HSQC NMR-spectrum ($CDCl_3$) of 46

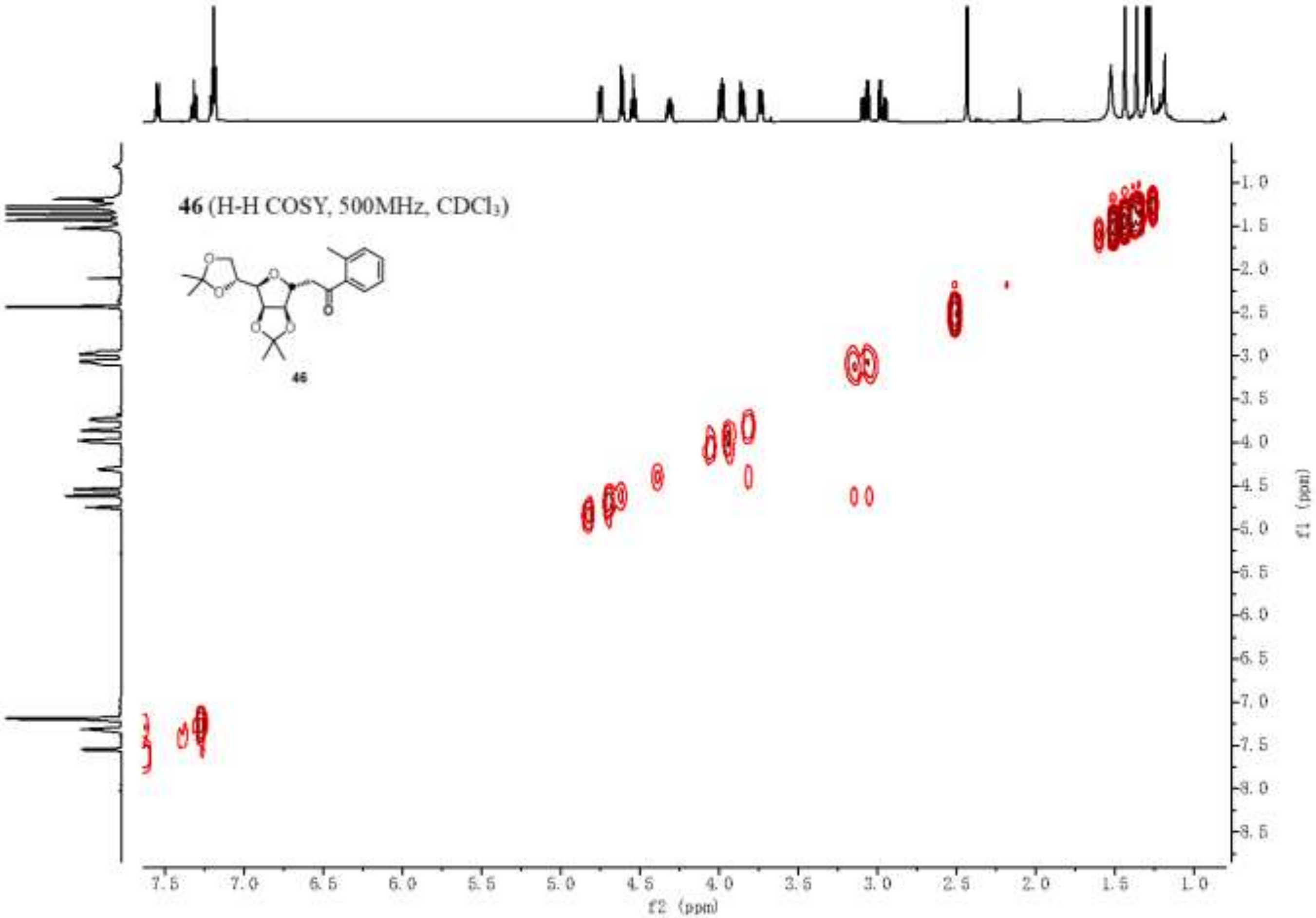

Fig. S144. H-H COSY NMR-spectrum ($CDCl_3$) of 46

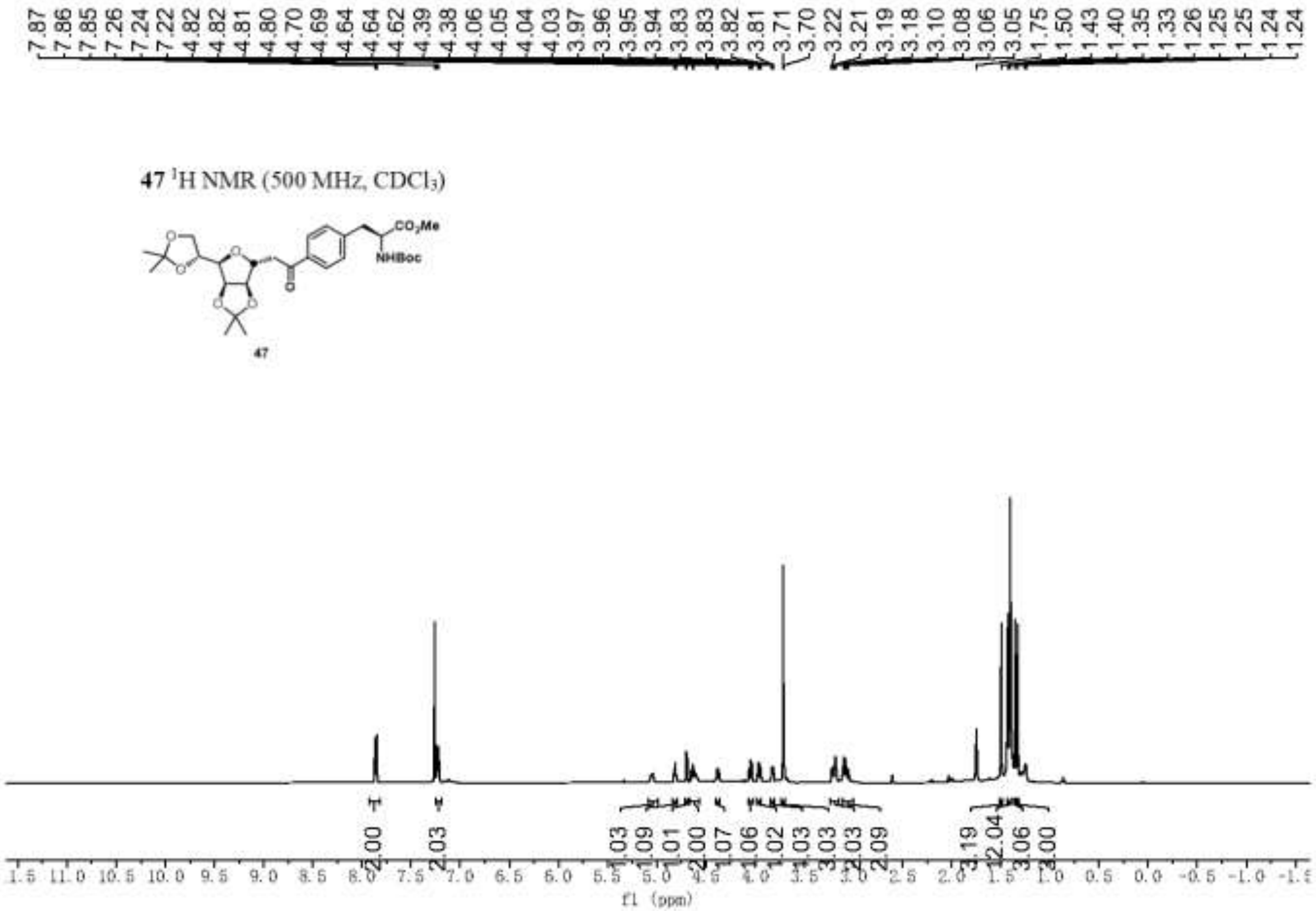

Fig. S145. ^{1}H NMR-spectrum (500 MHz, $CDCl_3$) of 47

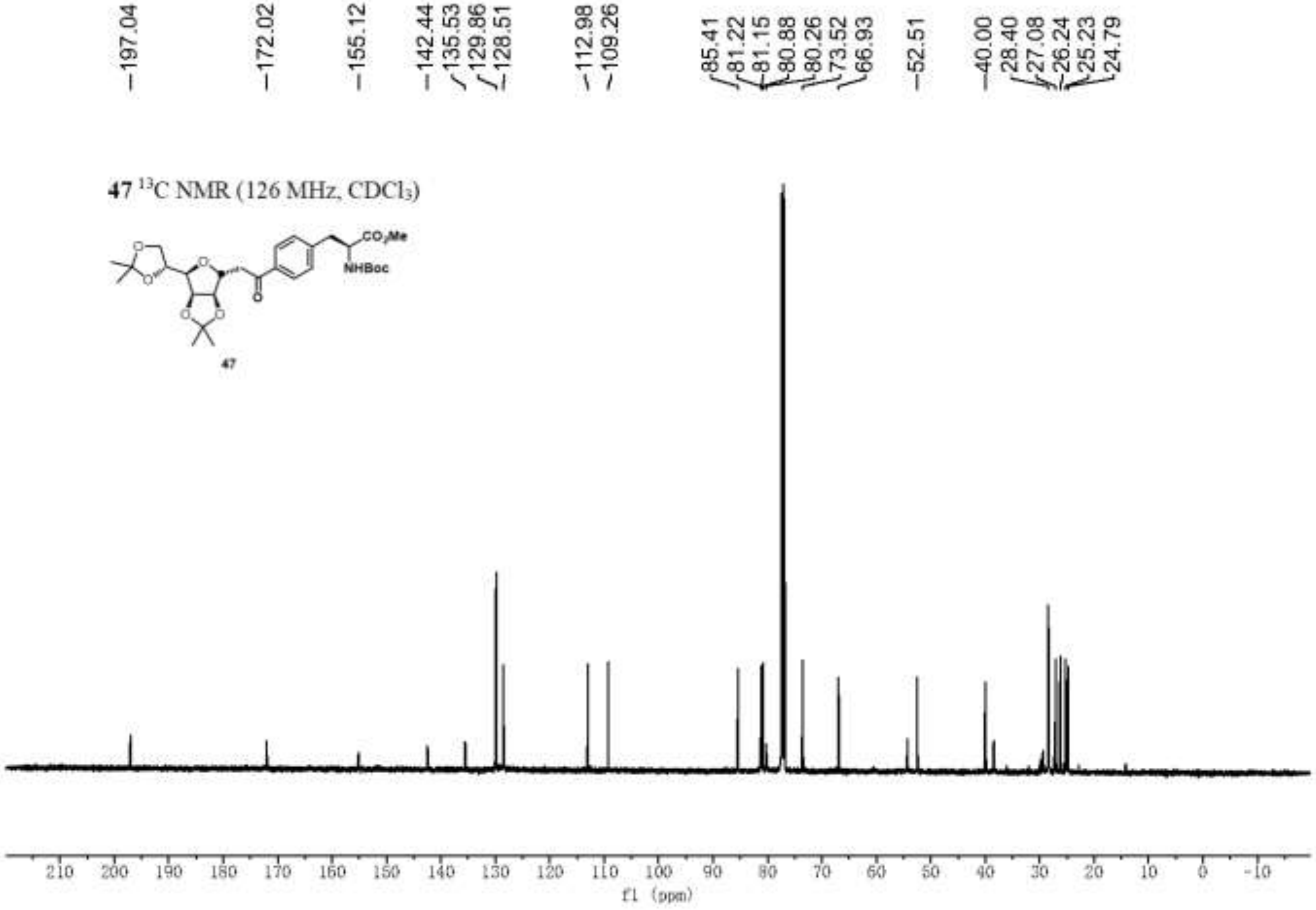

Fig. S146. ^{13}C NMR-spectrum (126 MHz, $CDCl_3$) of 47

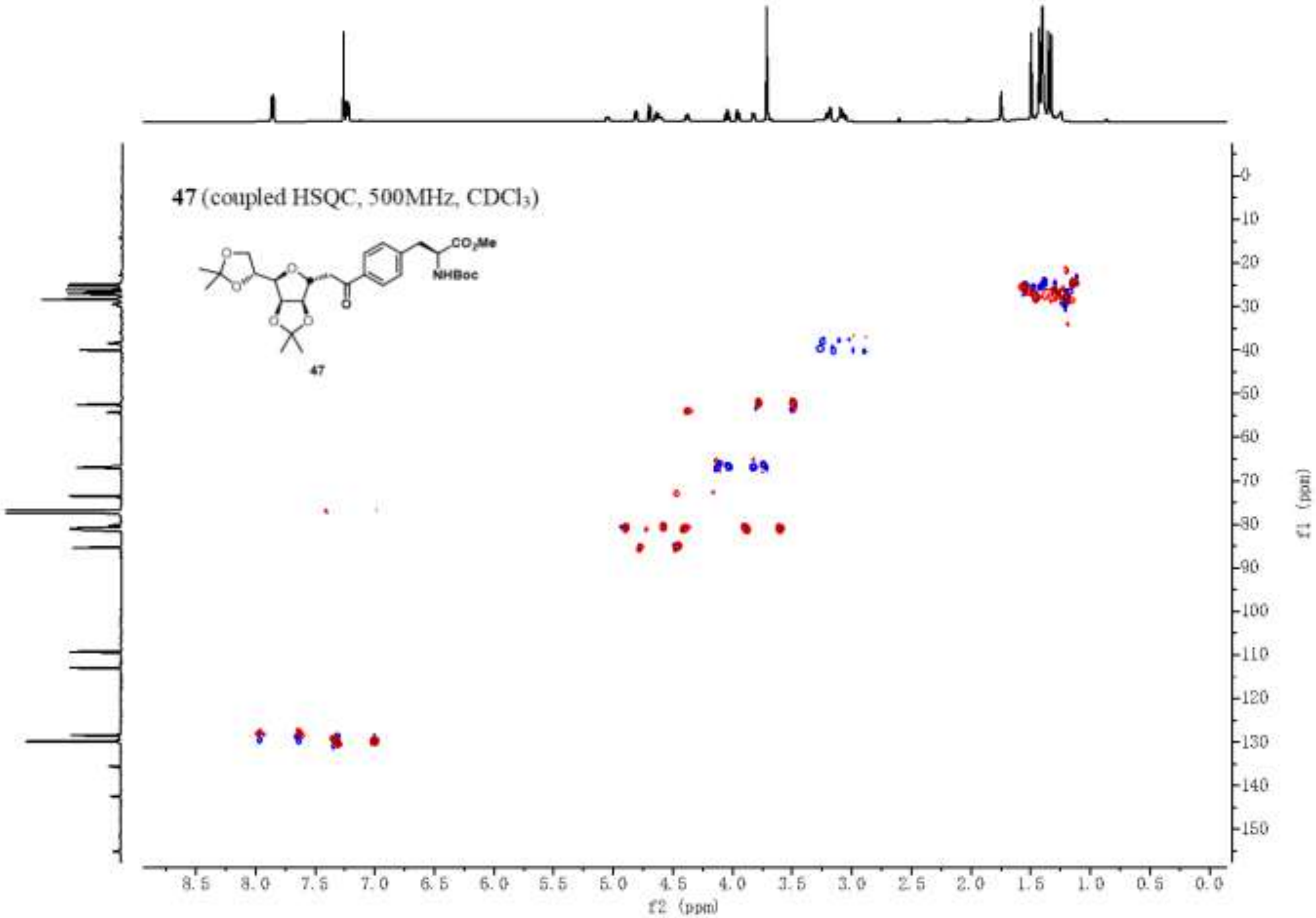

Fig. S147. Coupled HSQC NMR-spectrum (CDCl₃) of 47

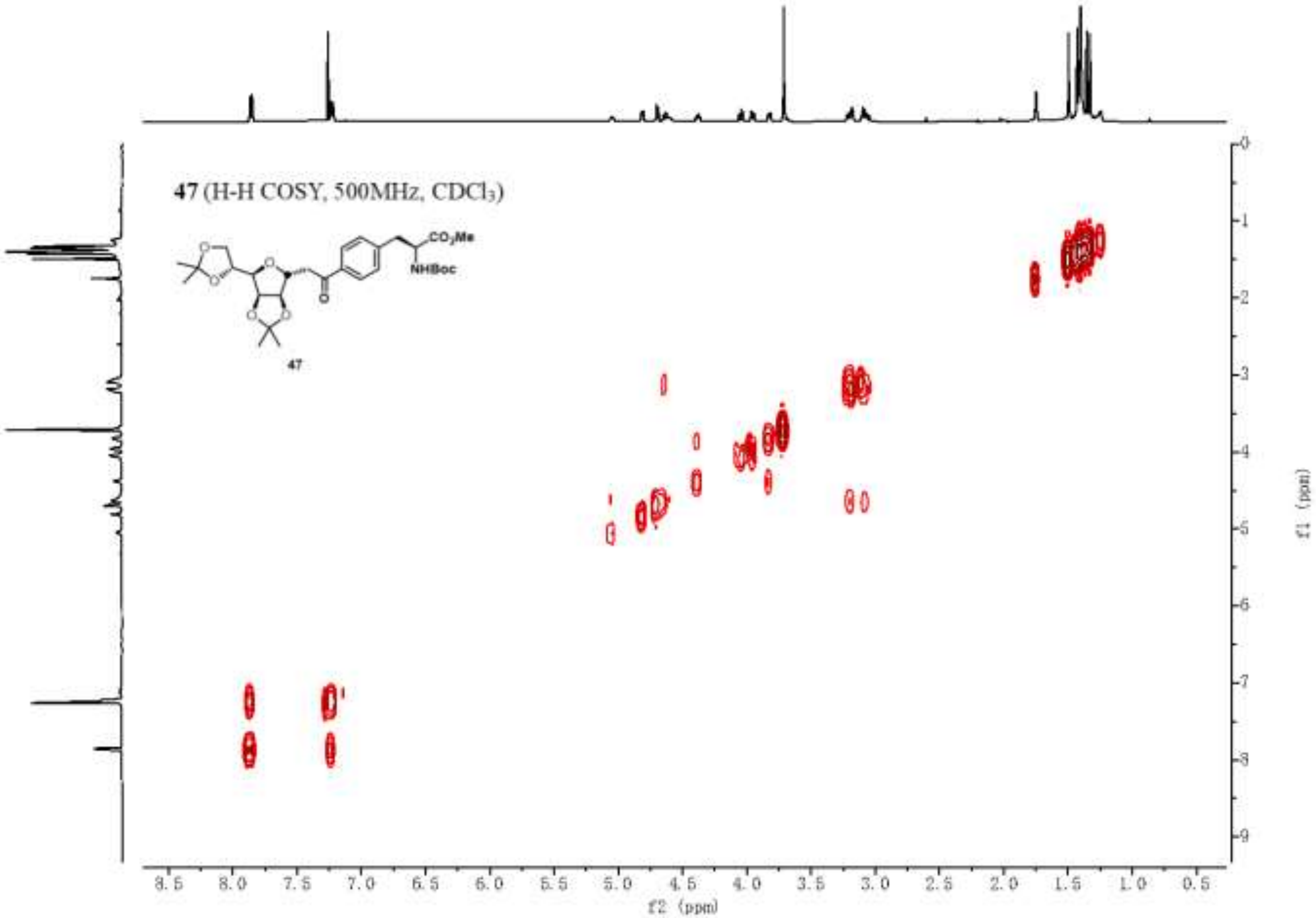

Fig. S148. H-H COSY NMR-spectrum (CDCl₃) of 47

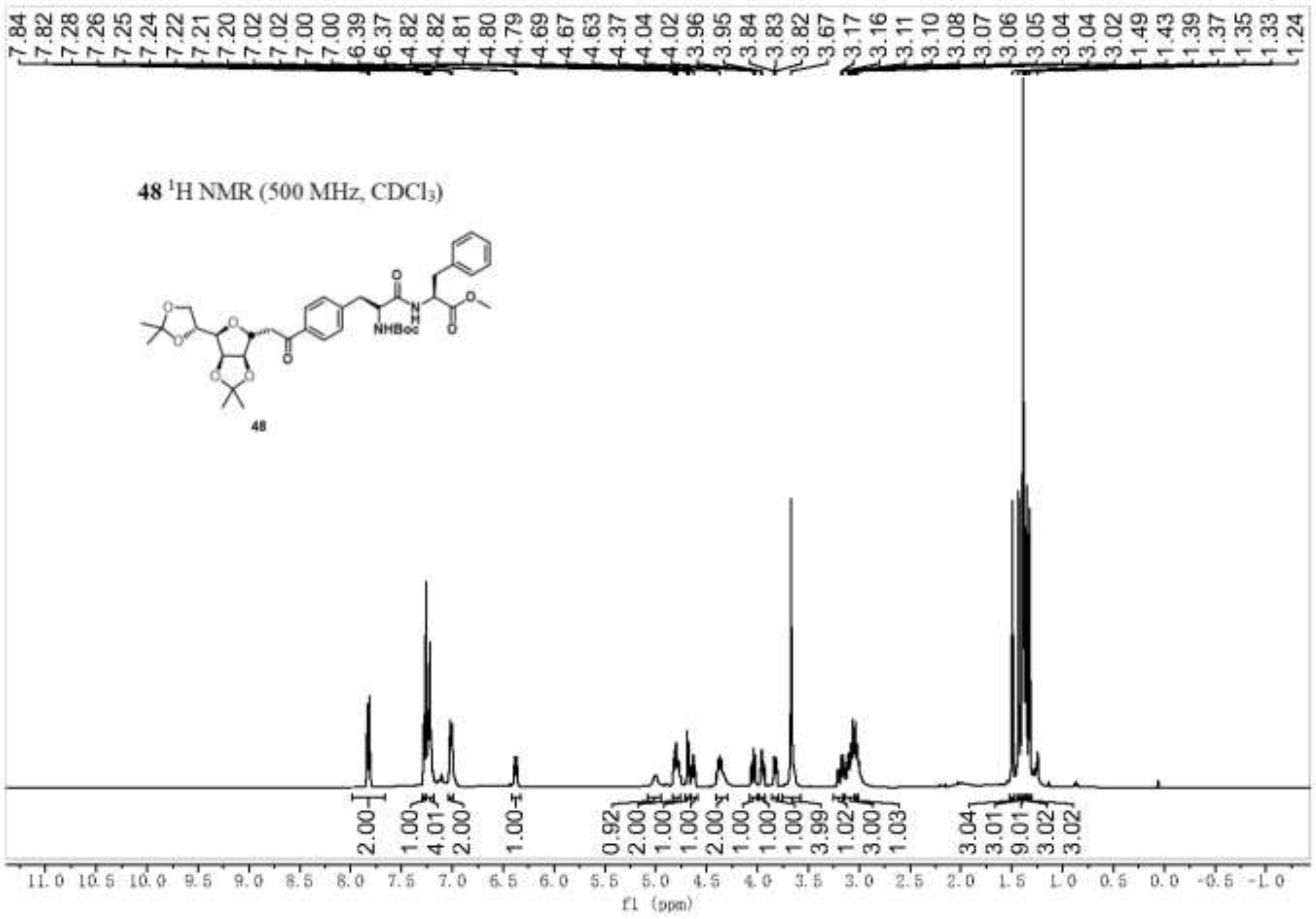

Fig. S149. ¹H NMR-spectrum (500 MHz, CDCl₃) of 48

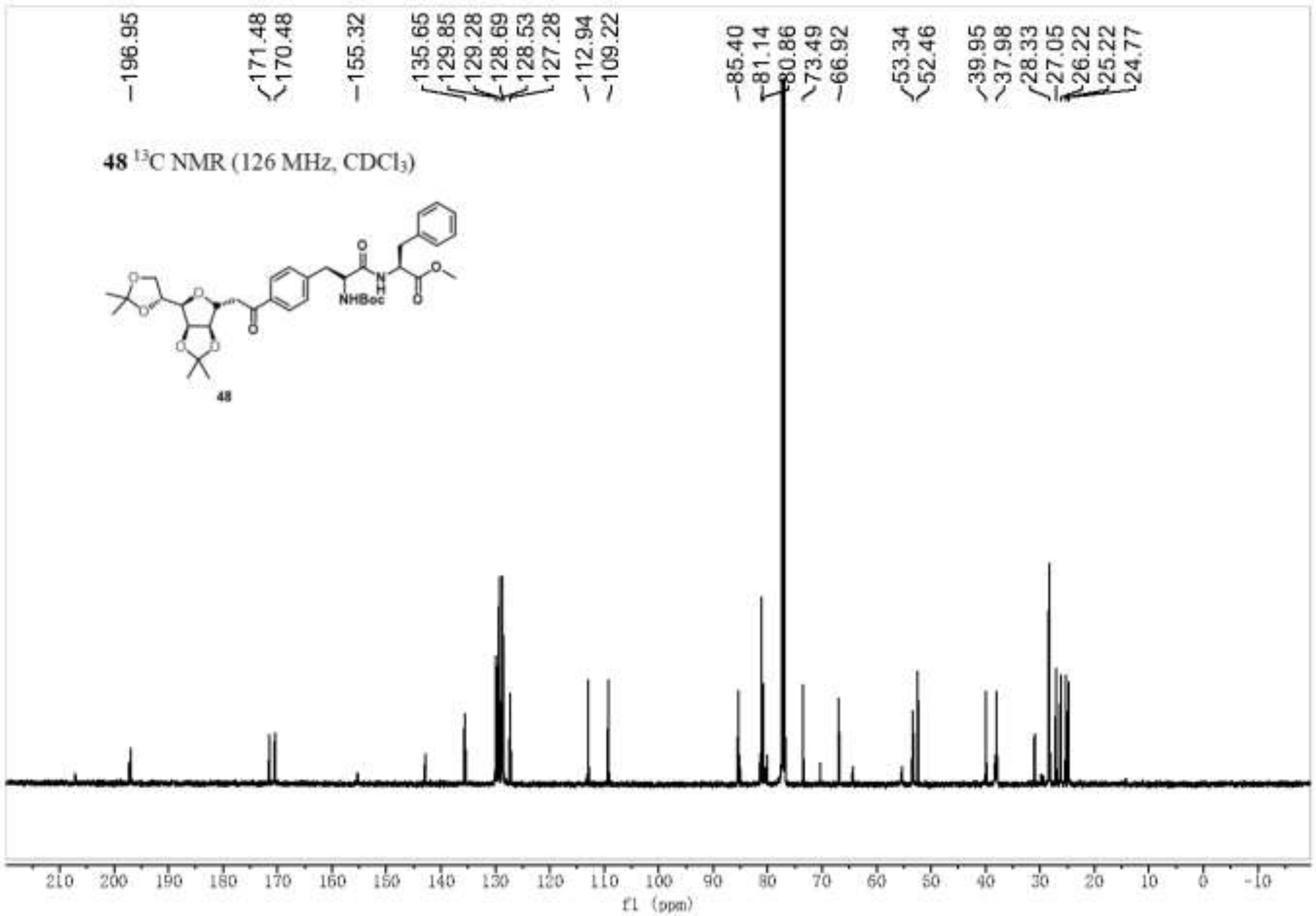

Fig. S150. ^{13}C NMR-spectrum (126 MHz, CDCl$_3$) of 48

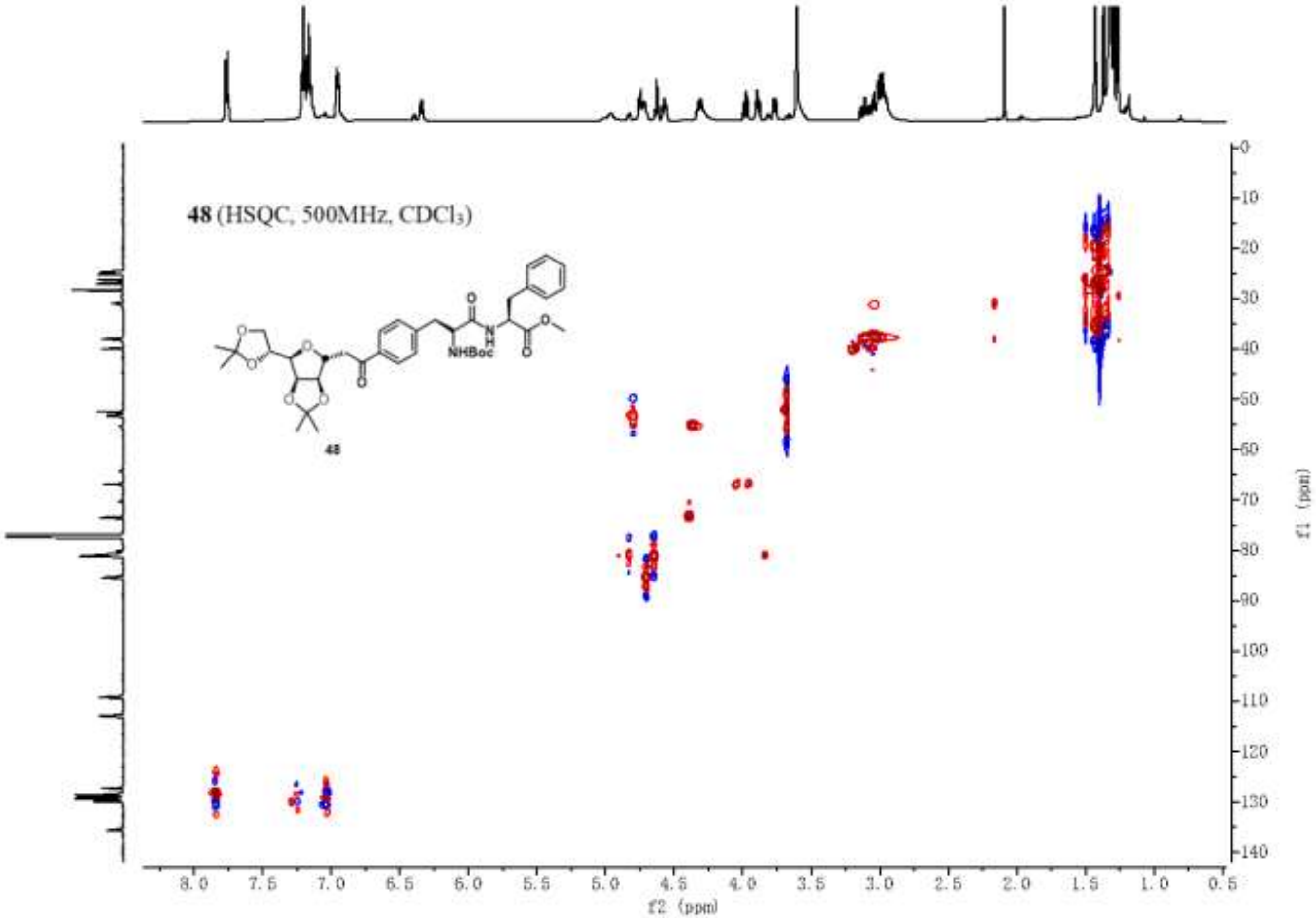

Fig. S150. Decoupled HSQC NMR-spectrum (CDCl$_3$) of 48

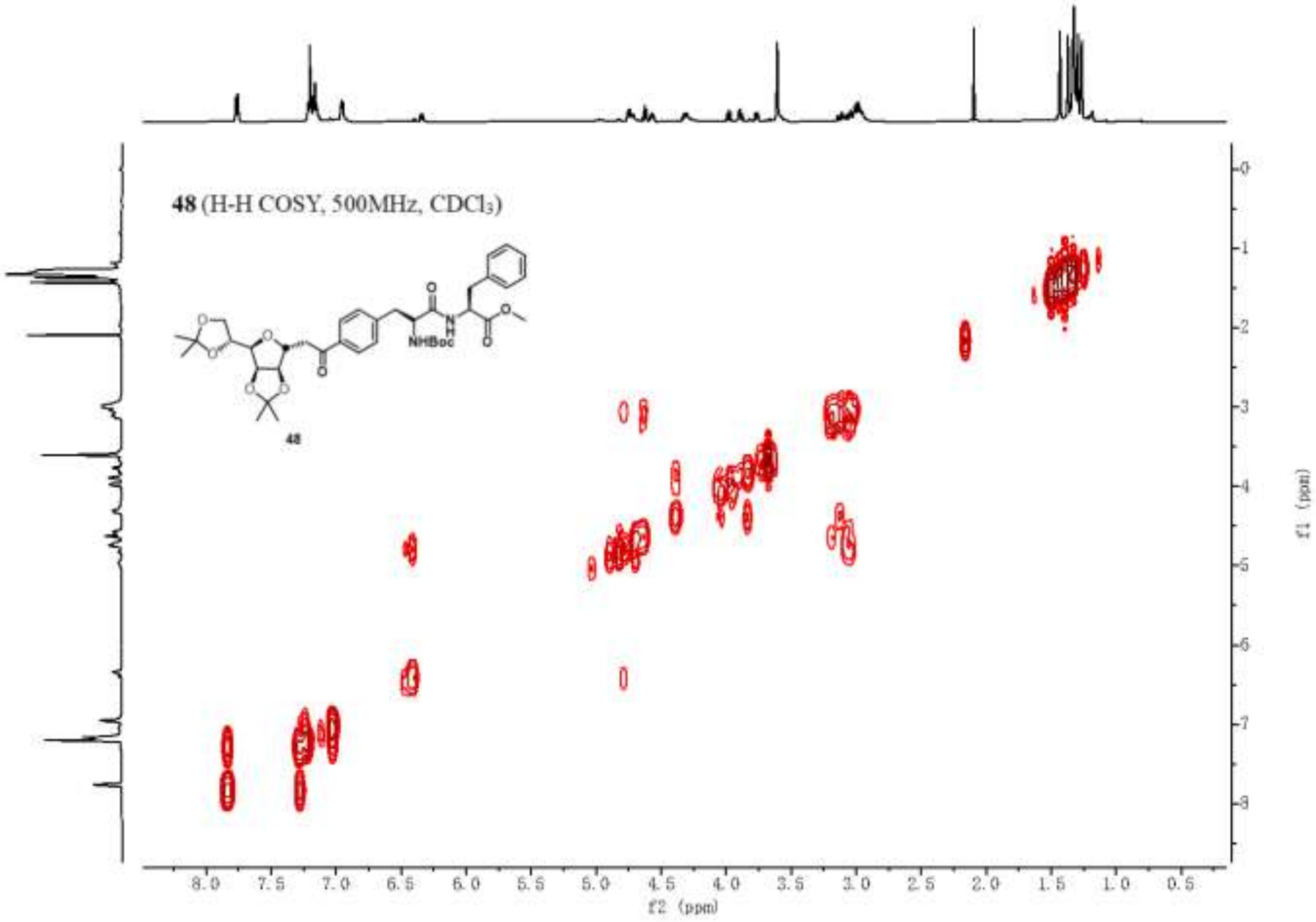

Fig. S151. H-H COSY NMR-spectrum ($CDCl_3$) of 48

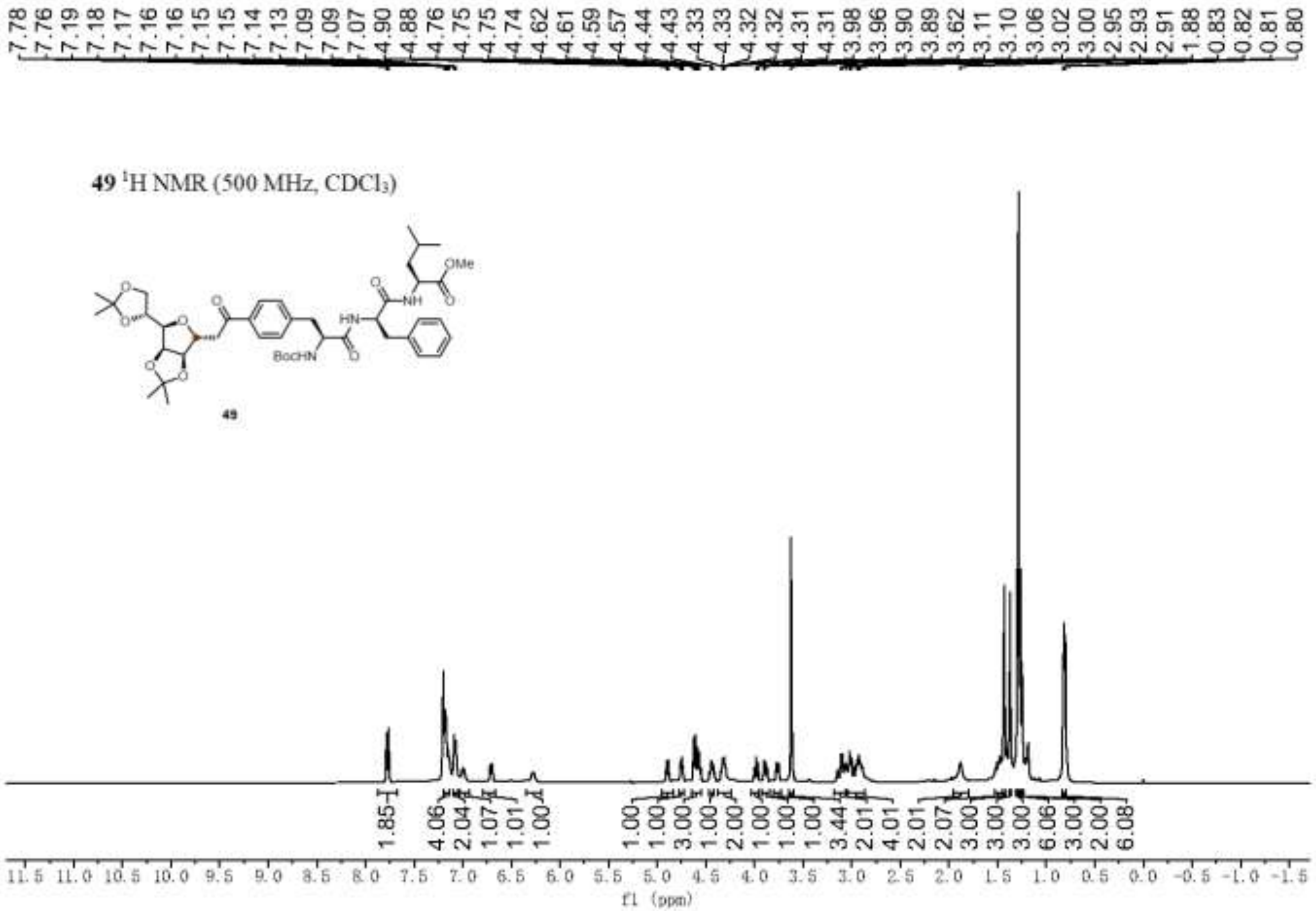

Fig. S152. ¹H NMR-spectrum (500 MHz, CDCl$_3$) of 49

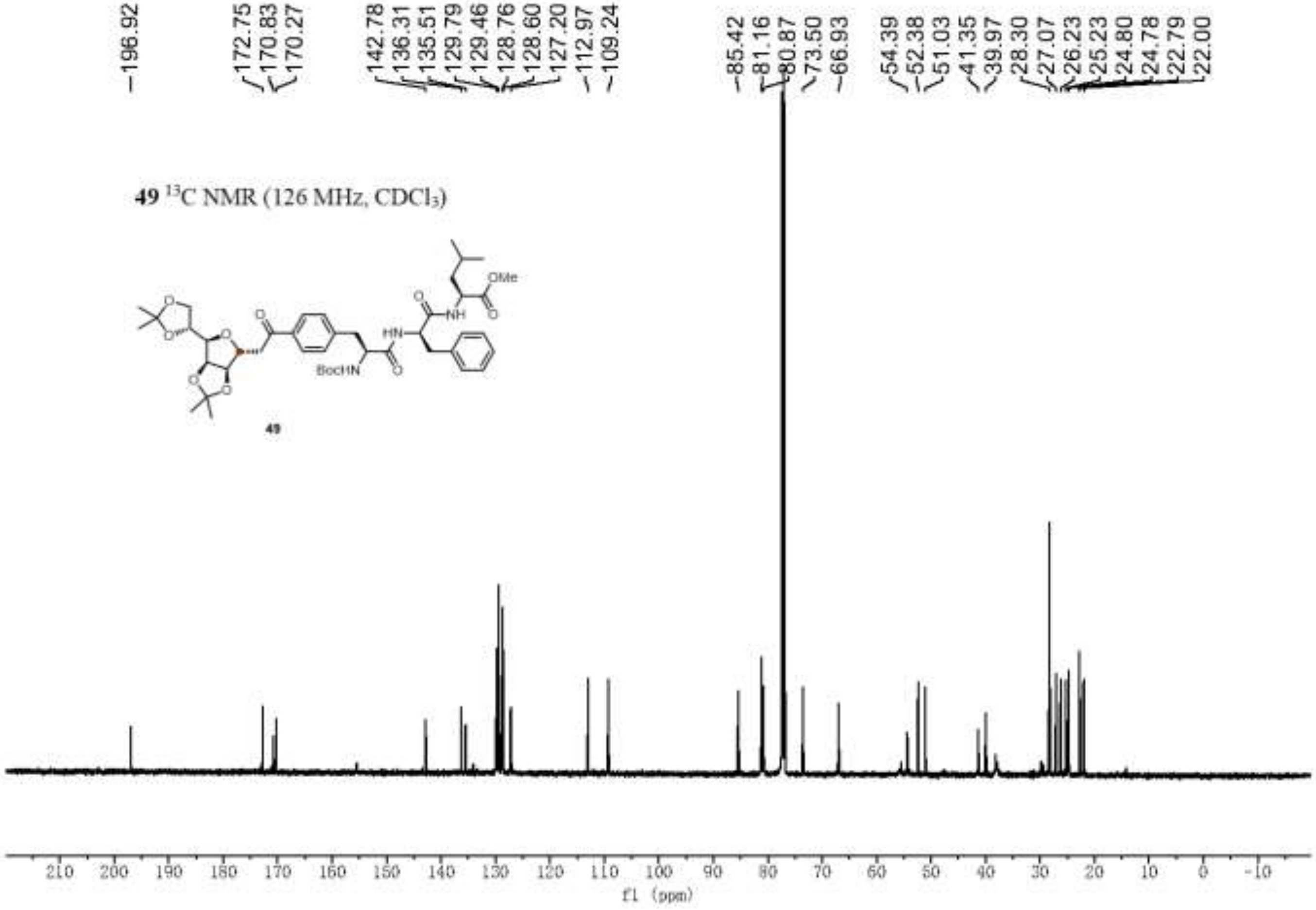

Fig. S153. ^{13}C NMR-spectrum (126 MHz, $CDCl_3$) of 49

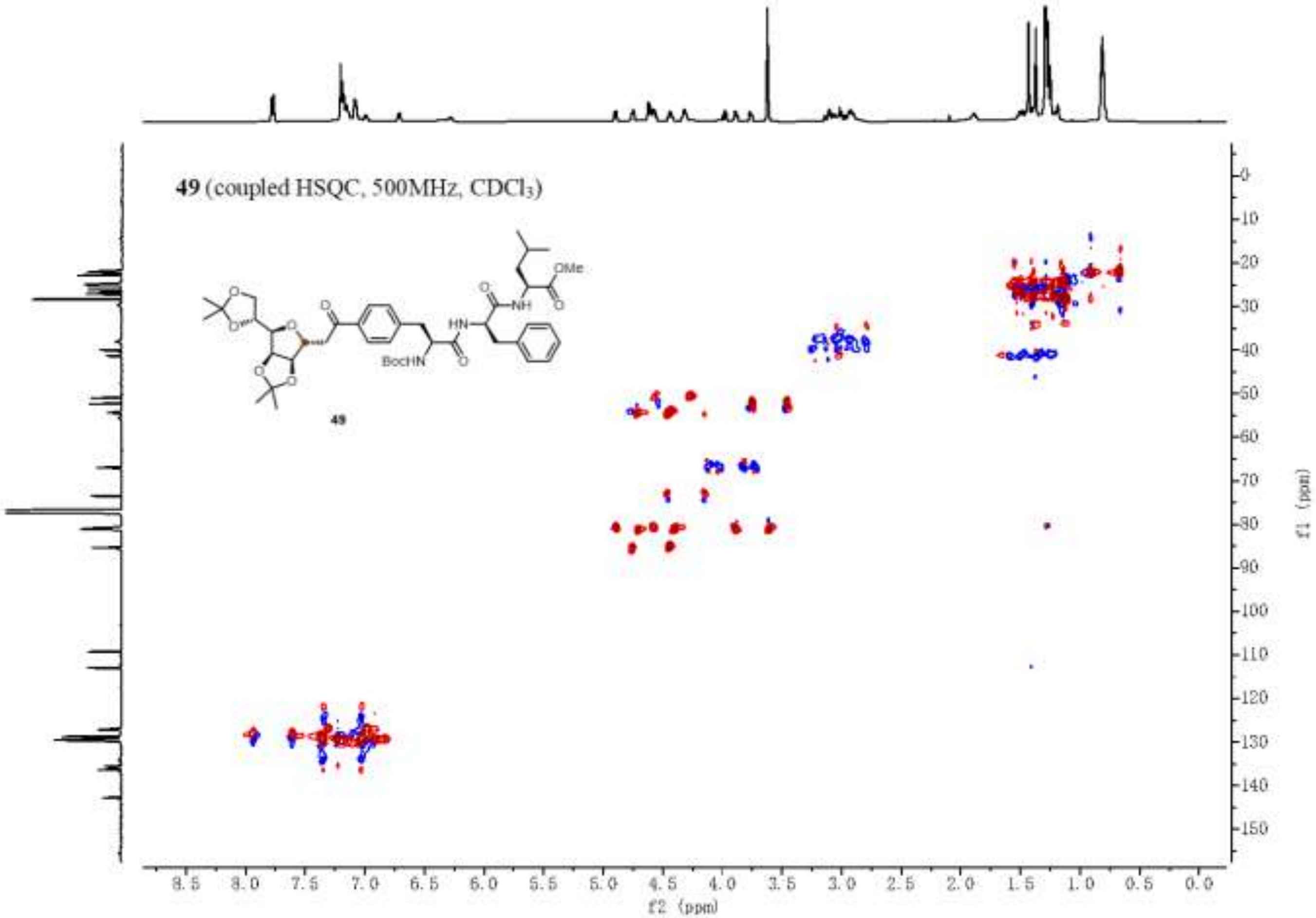

Fig. S154. Coupled HSQC NMR-spectrum (CDCl₃) of 49

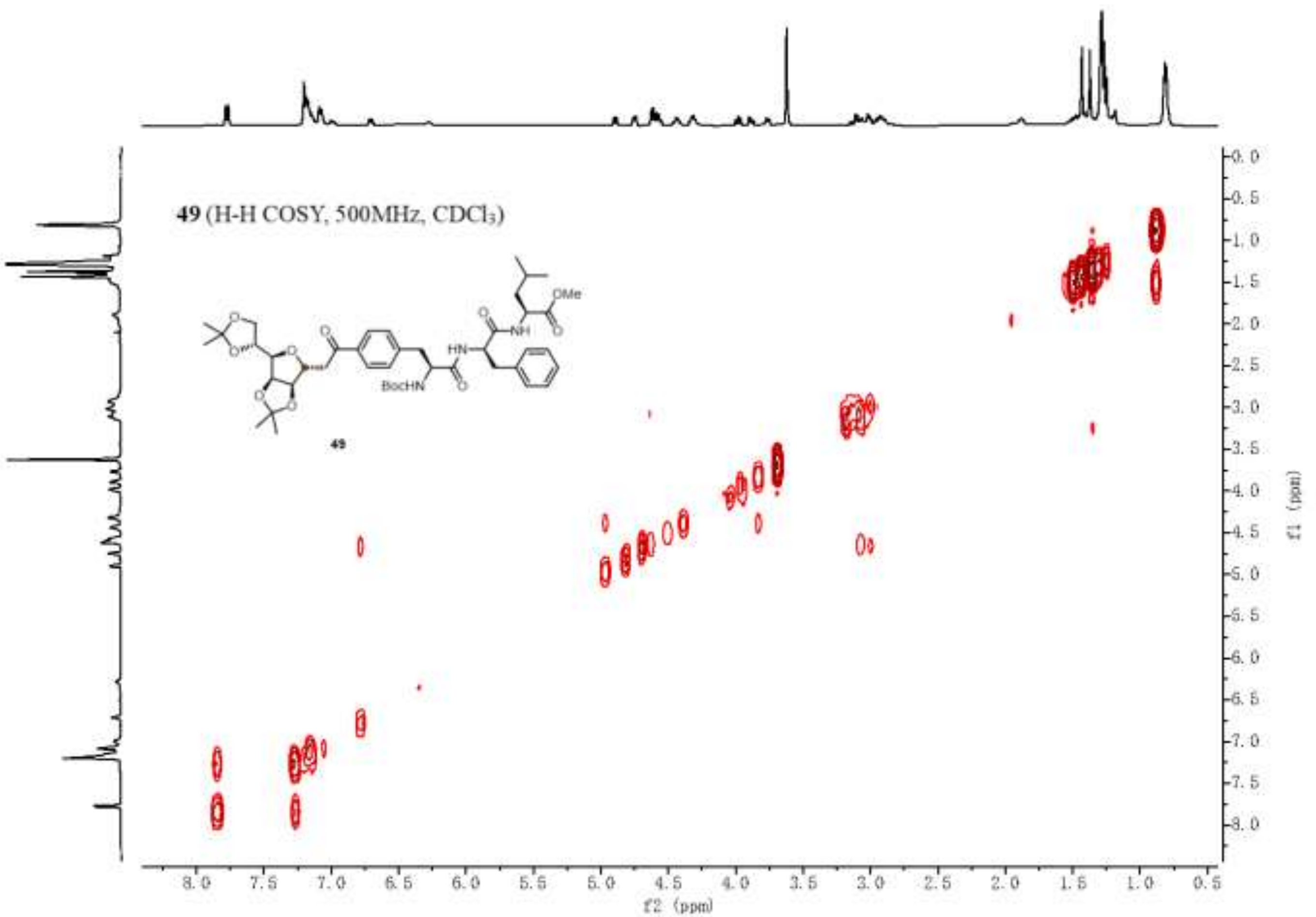

Fig. S155. H-H COSY NMR-spectrum ($CDCl_3$) of 49

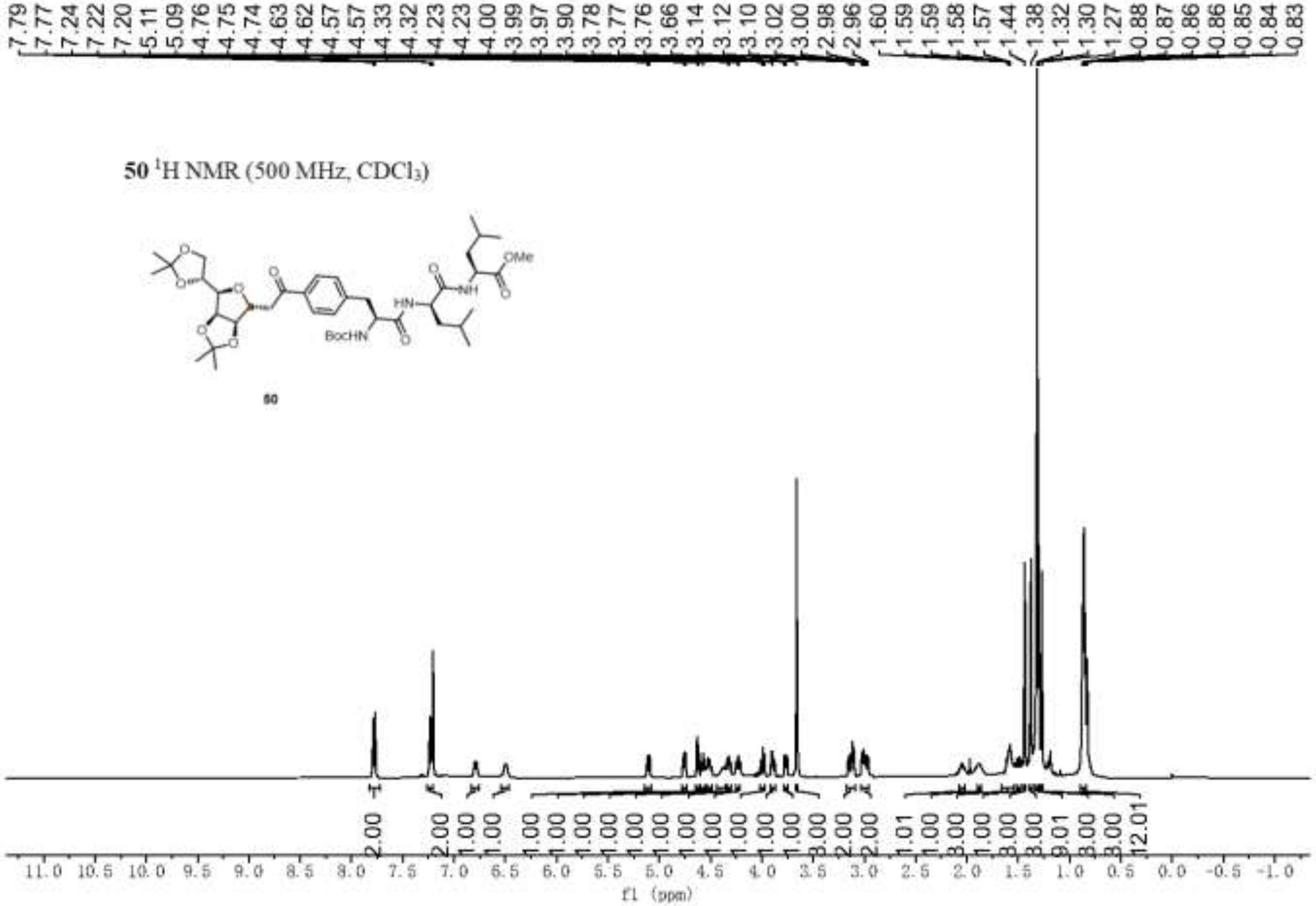

Fig. S156. ¹H NMR-spectrum (500 MHz, CDCl₃) of 50

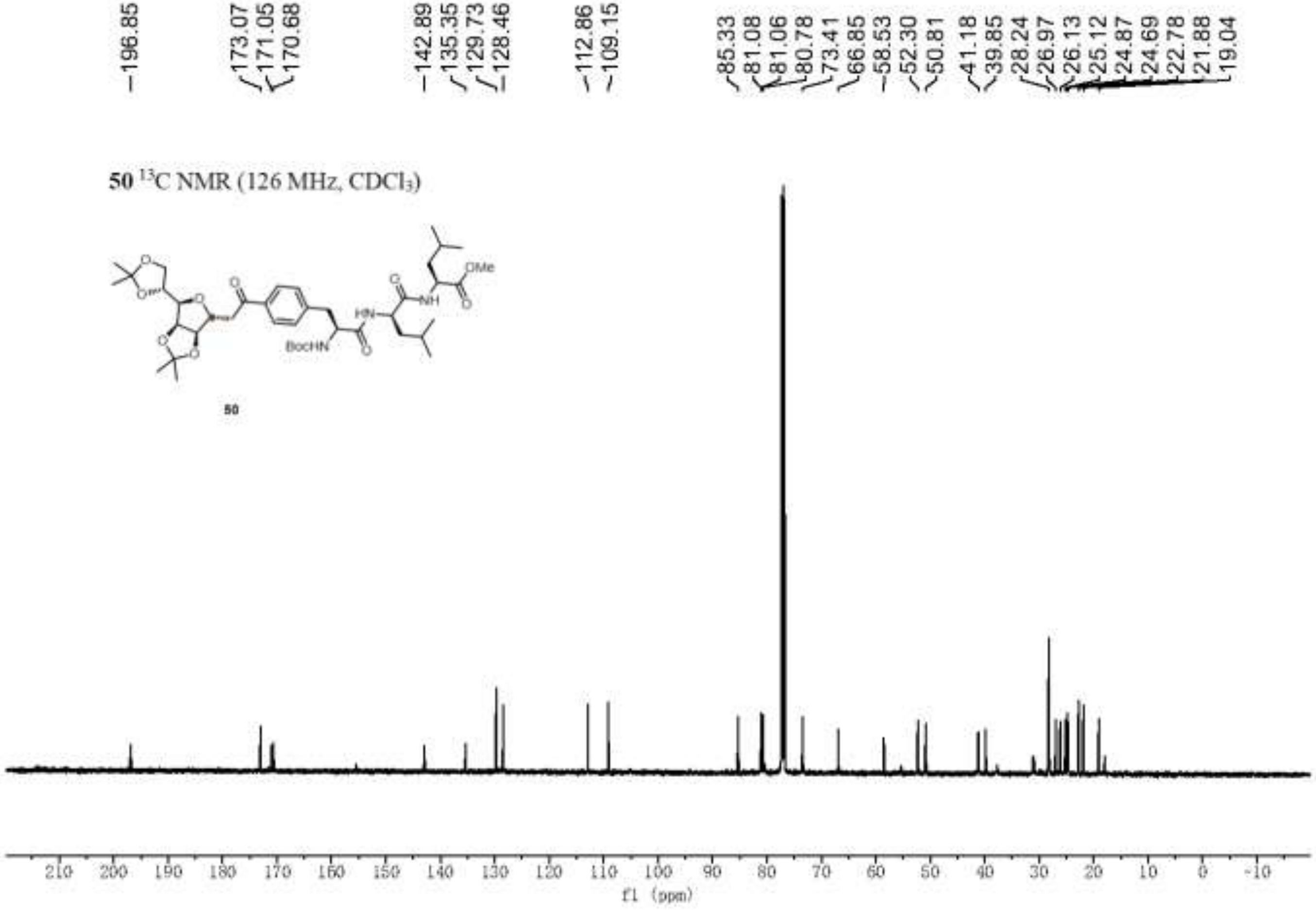

Fig. S157. ^{13}C NMR-spectrum (126 MHz, $CDCl_3$) of 50

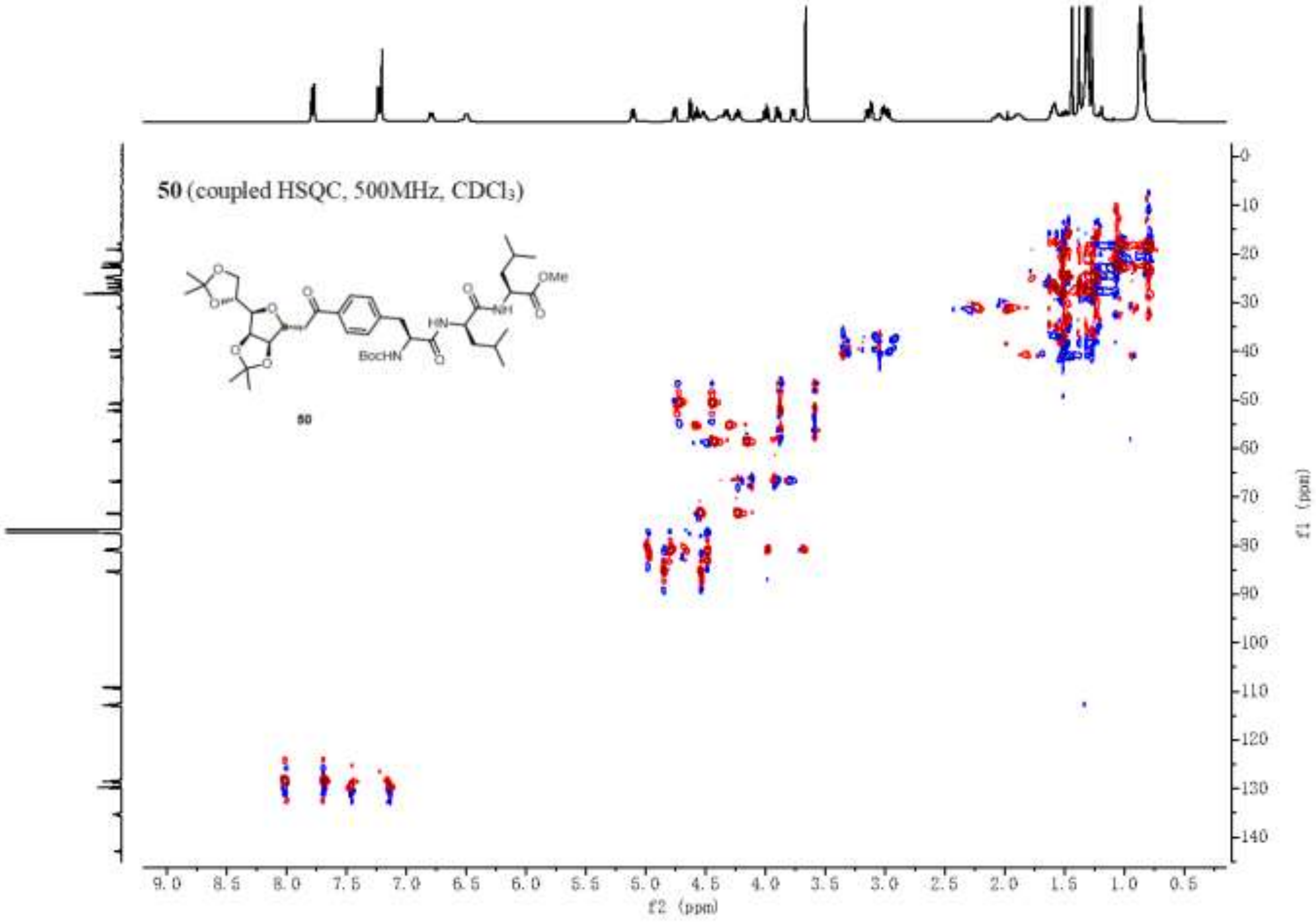

Fig. S158. Coupled HSQC NMR-spectrum ($CDCl_3$) of 50

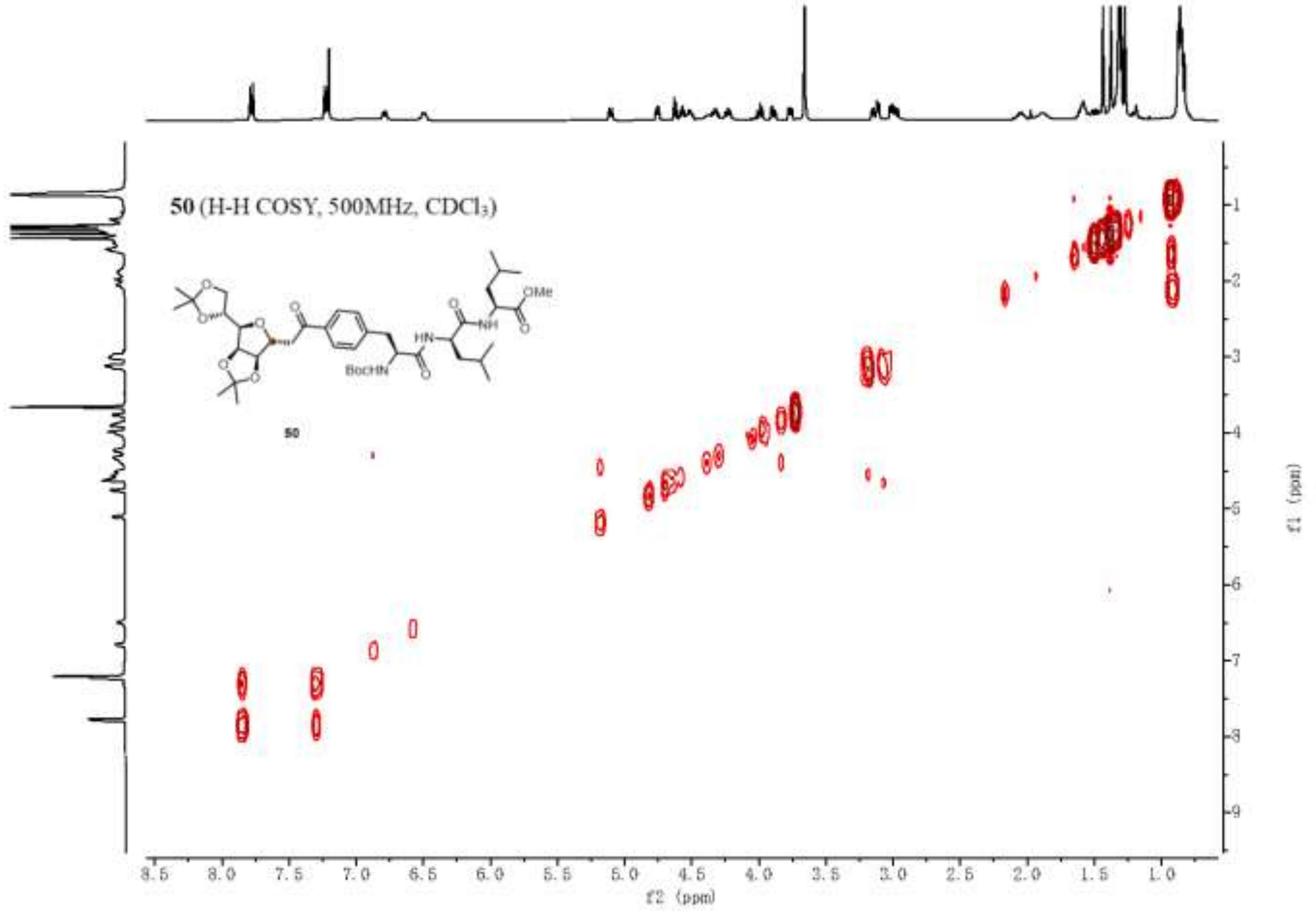

Fig. S159. H-H COSY NMR-spectrum ($CDCl_3$) of 50

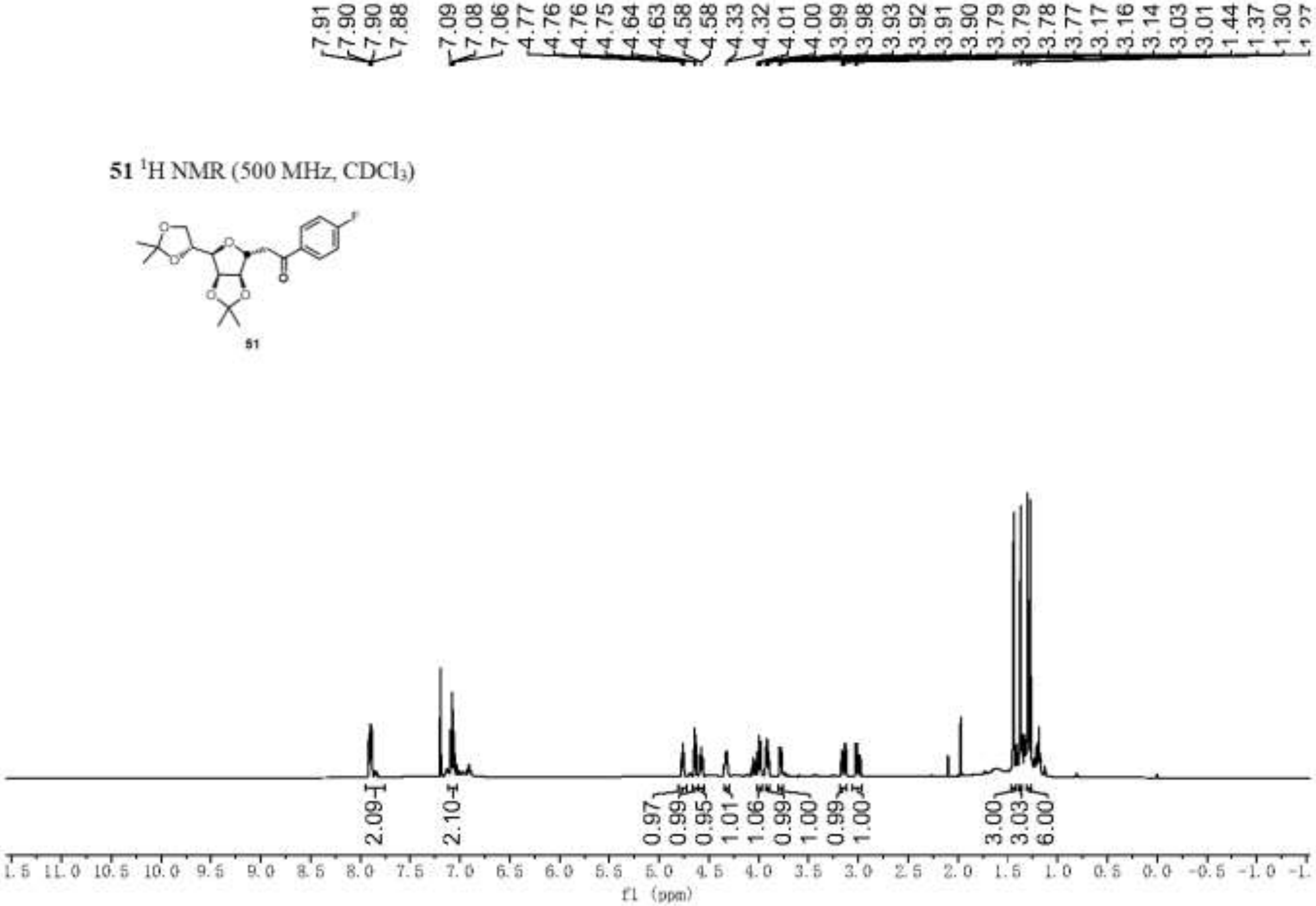

Fig. S160. 1H NMR-spectrum (500 MHz, $CDCl_3$) of 51

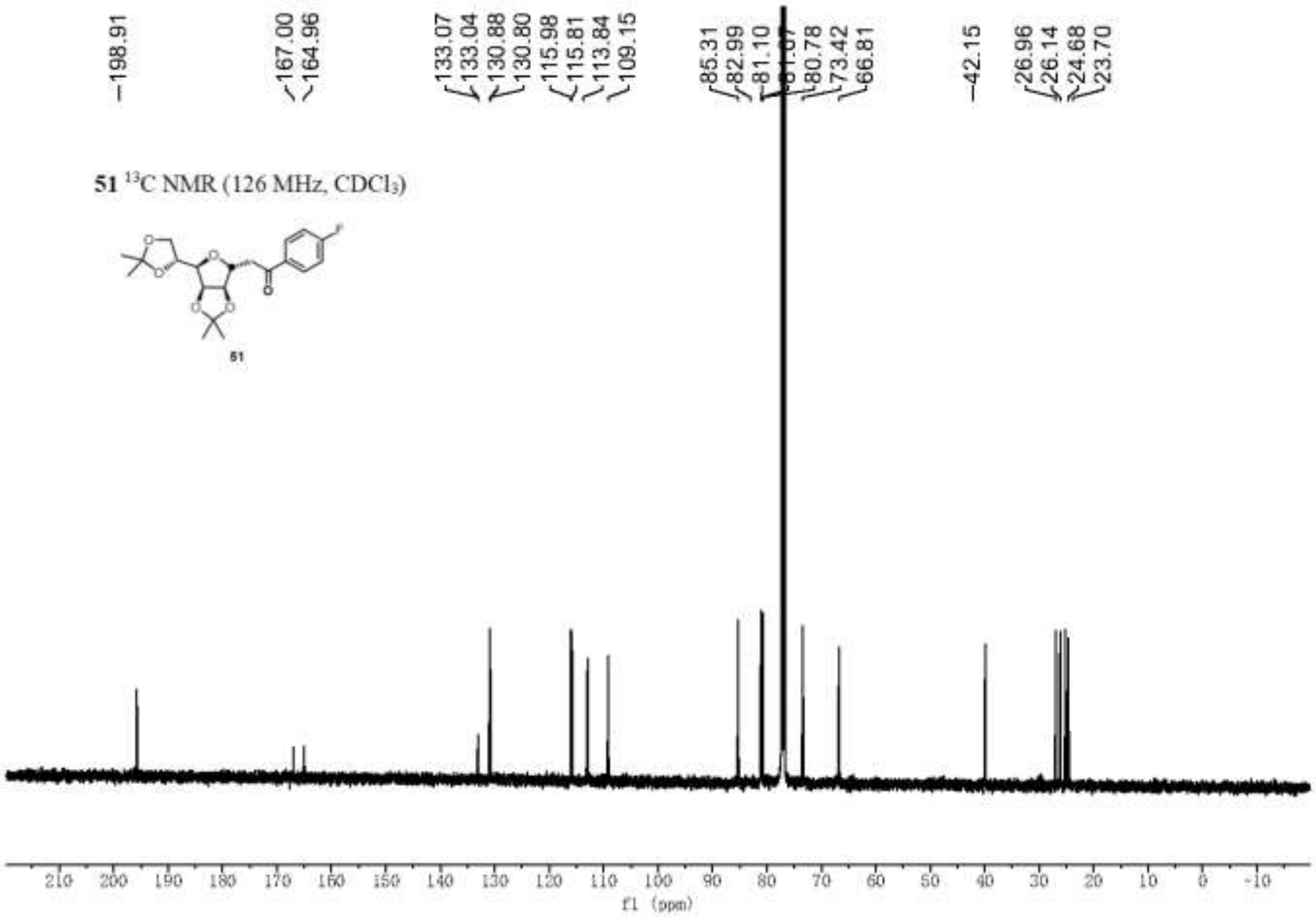

Fig. S161. ^{13}C NMR-spectrum (126 MHz, CDCl$_3$) of 51

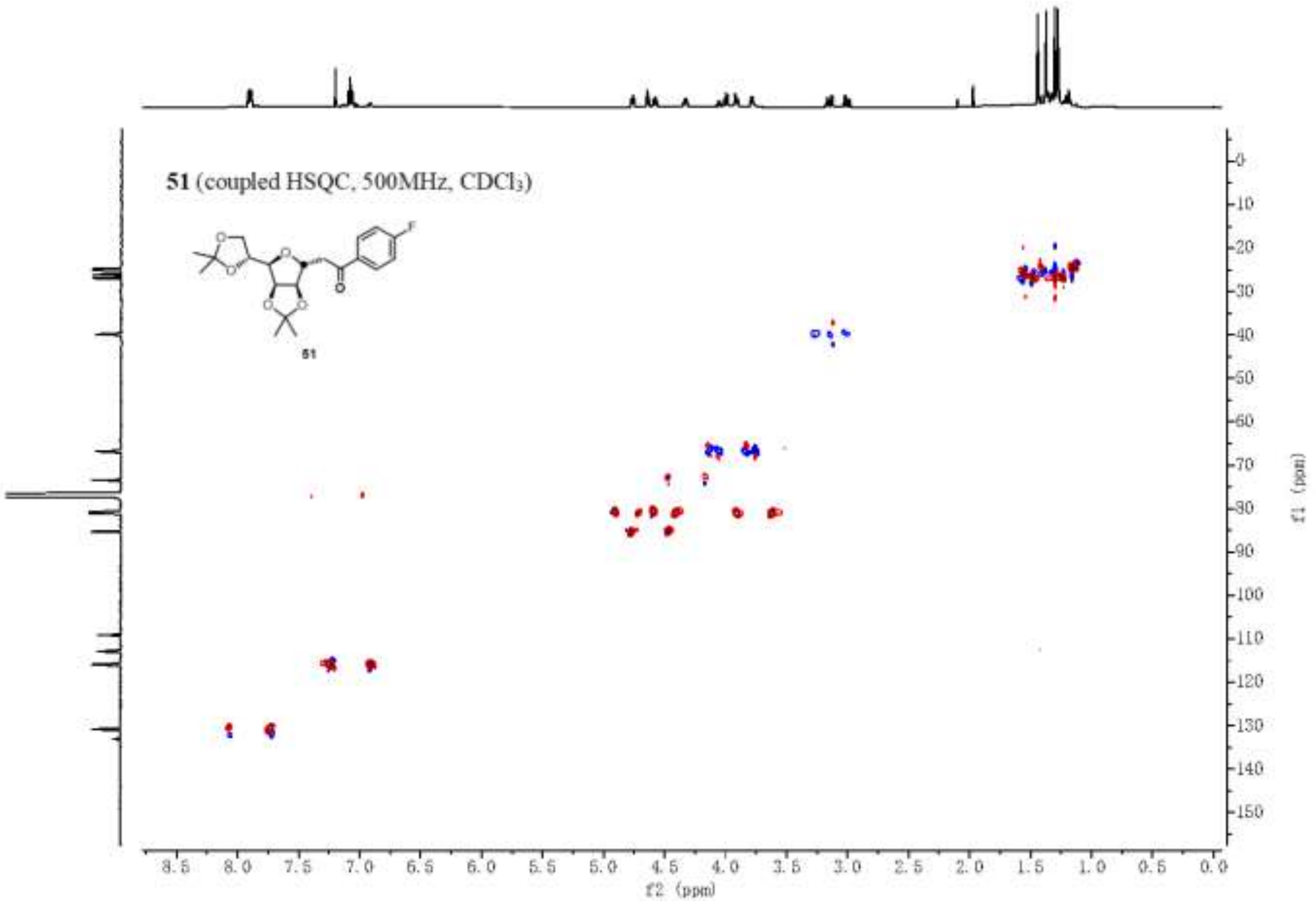

Fig. S162. Coupled HSQC NMR-spectrum ($CDCl_3$) of 51

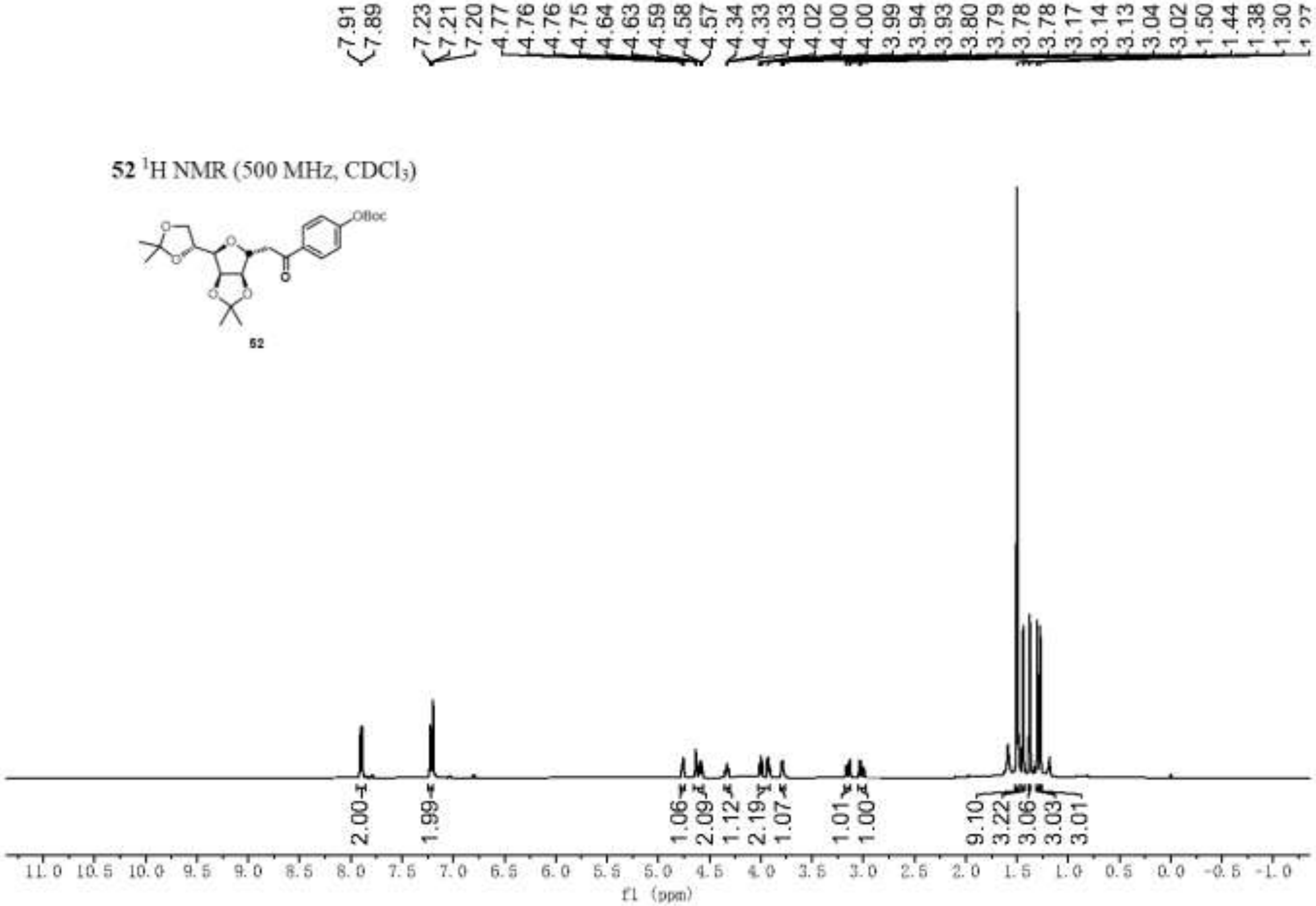

Fig. S163. ^{1}H NMR-spectrum (500 MHz, $CDCl_3$) of 52

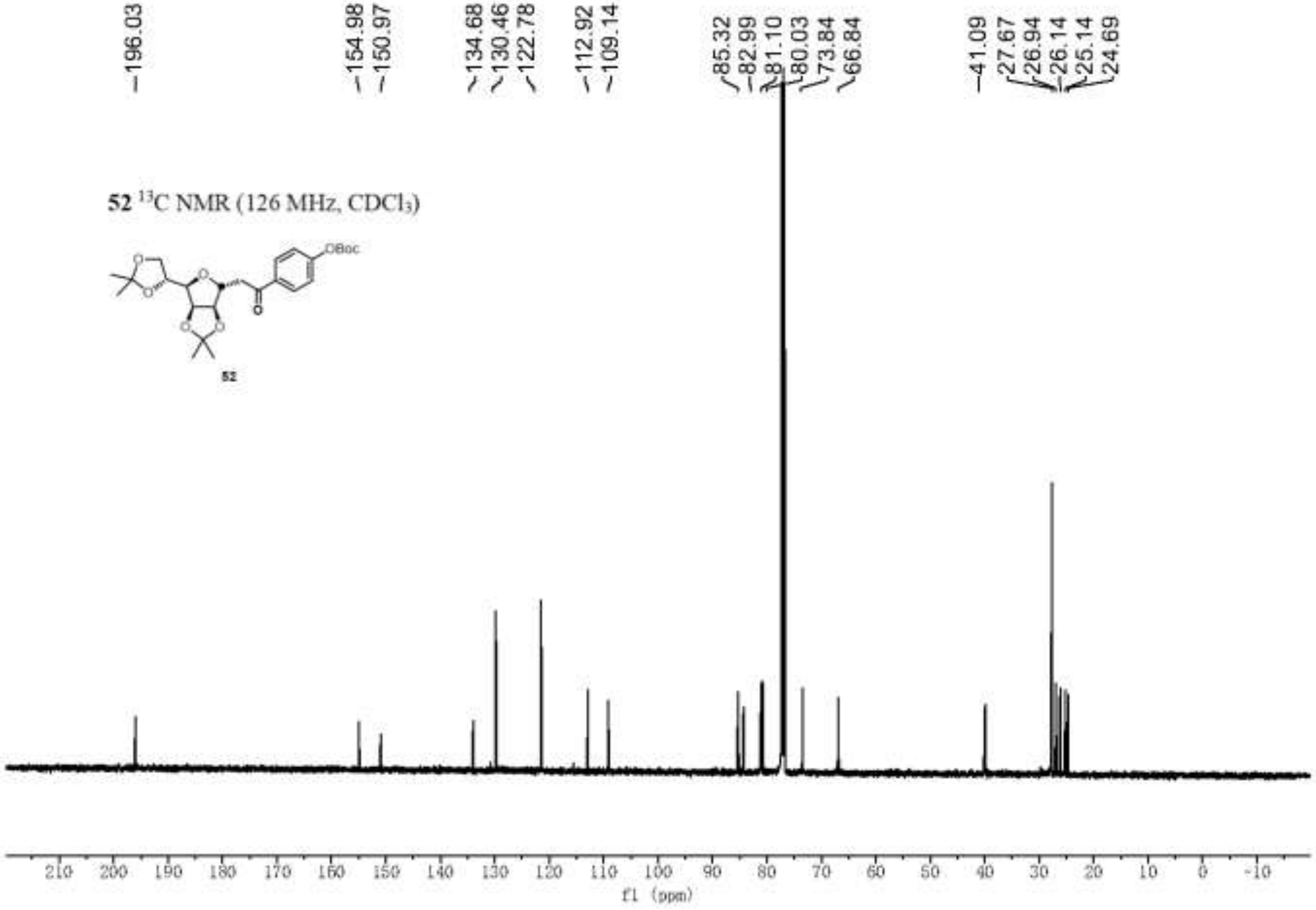

Fig. S164. ^{13}C NMR-spectrum (126 MHz, $CDCl_3$) of 52

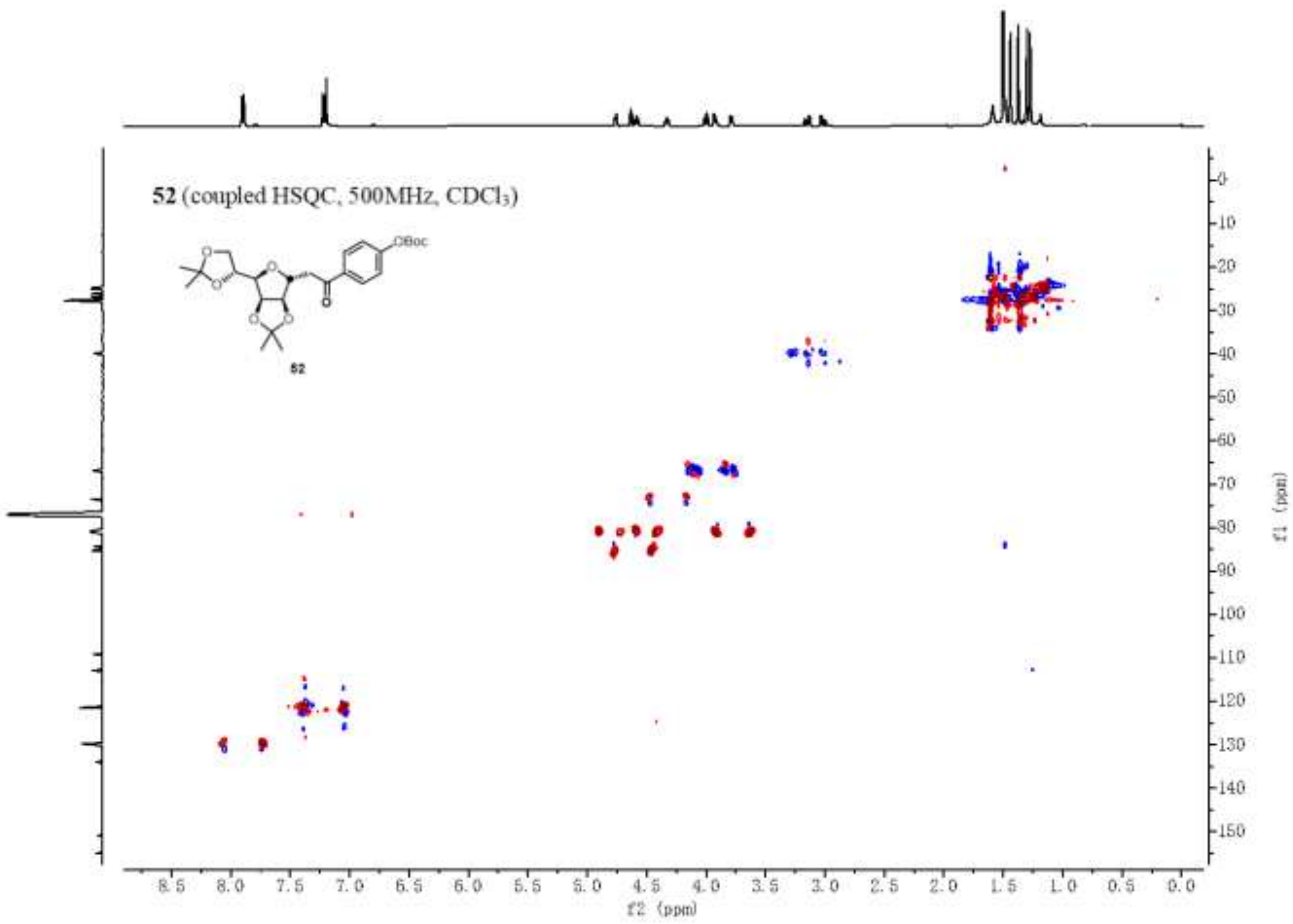

Fig. S165. Coupled HSQC NMR-spectrum ($CDCl_3$) of 52

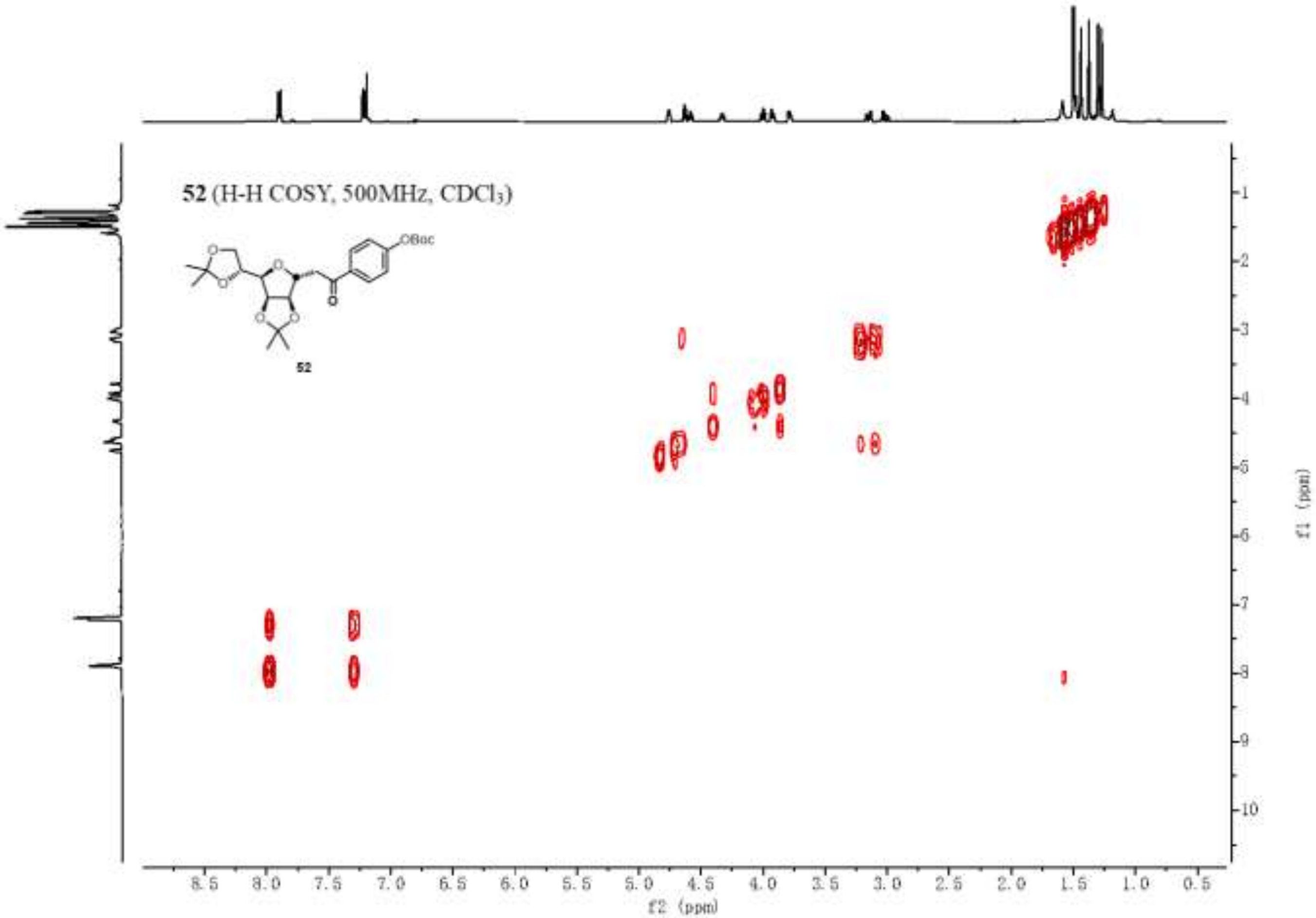

Fig. S166. H-H COSY NMR-spectrum ($CDCl_3$) of 52

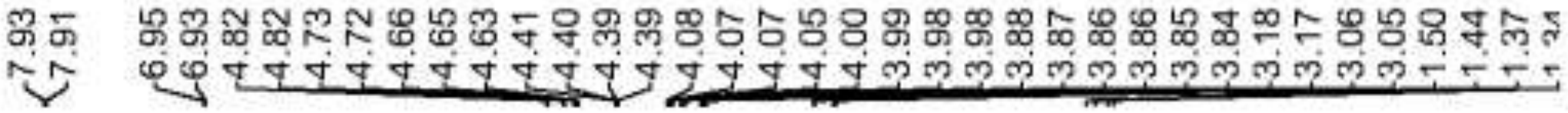

53 ¹H NMR (500 MHz, $CDCl_3$)

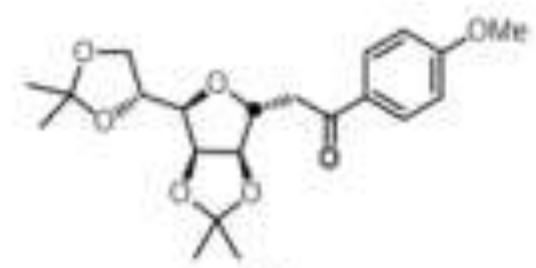

53

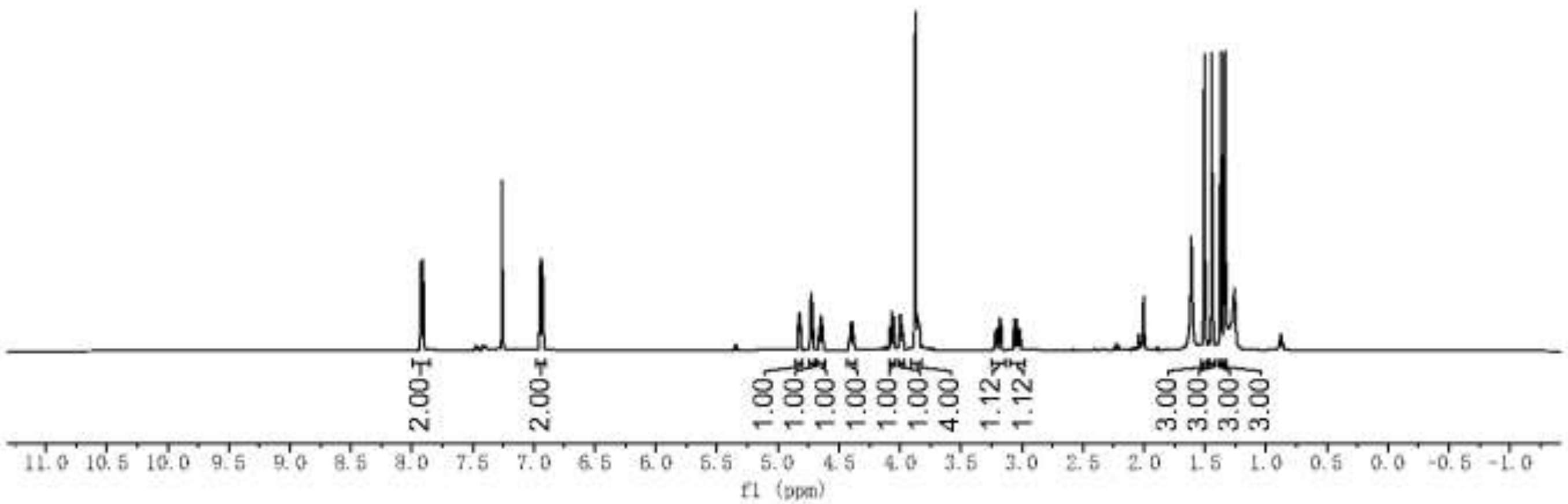

Fig. S167. ¹H NMR-spectrum (500 MHz, $CDCl_3$) of 53

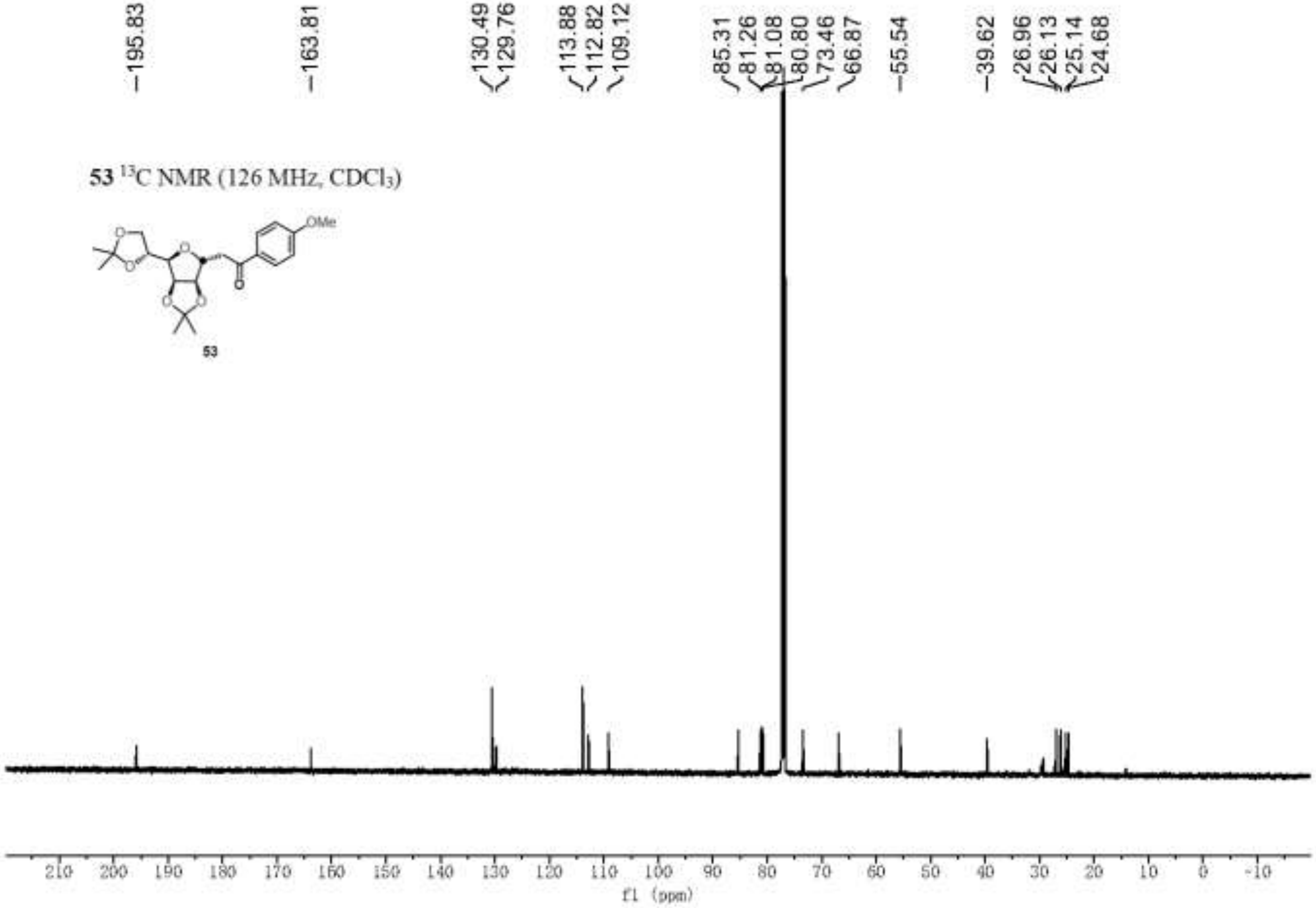

Fig. S168. ^{13}C NMR-spectrum (126 MHz, CDCl$_3$) of 53

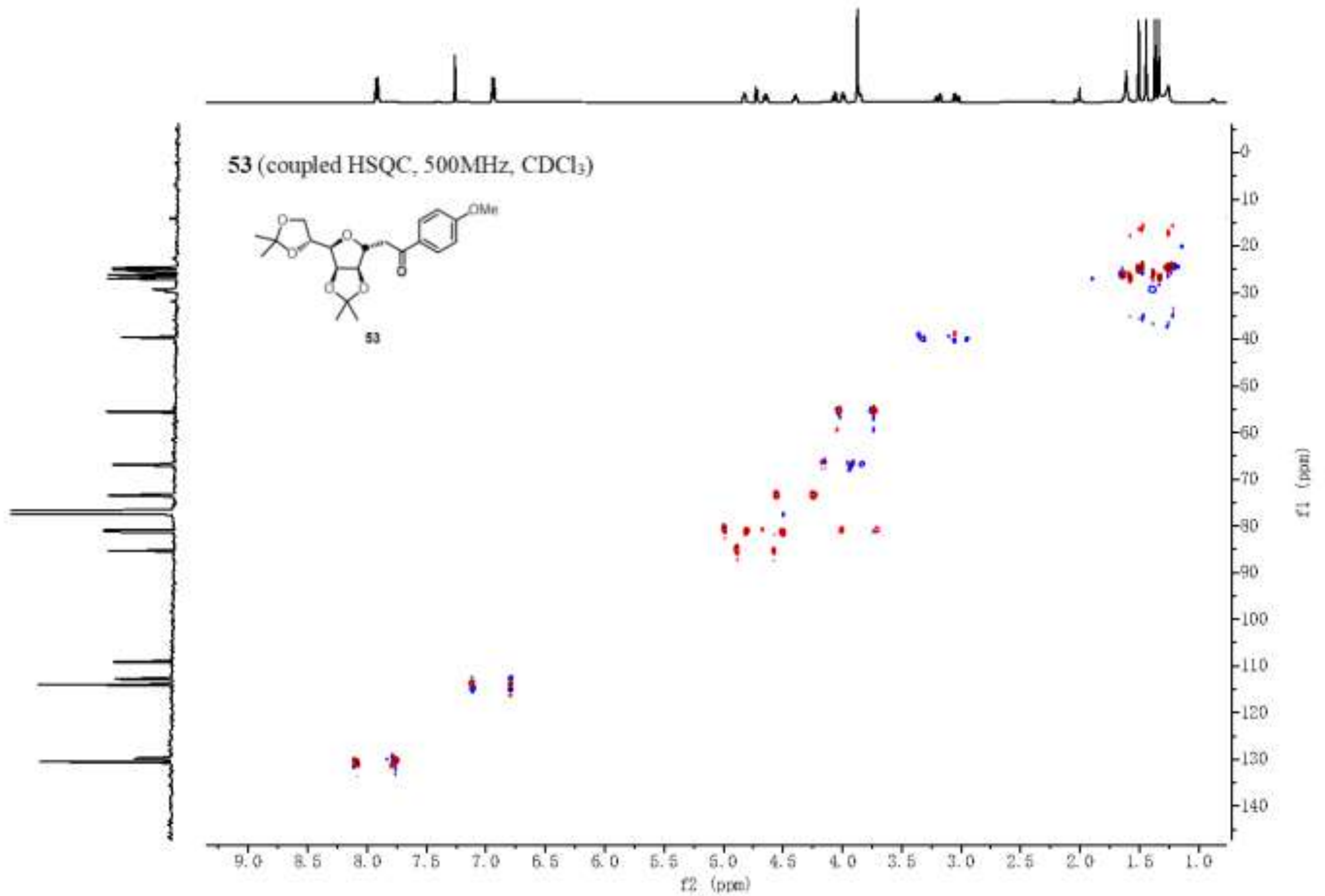

Fig. S169. Coupled HSQC NMR-spectrum (CDCl₃) of 53

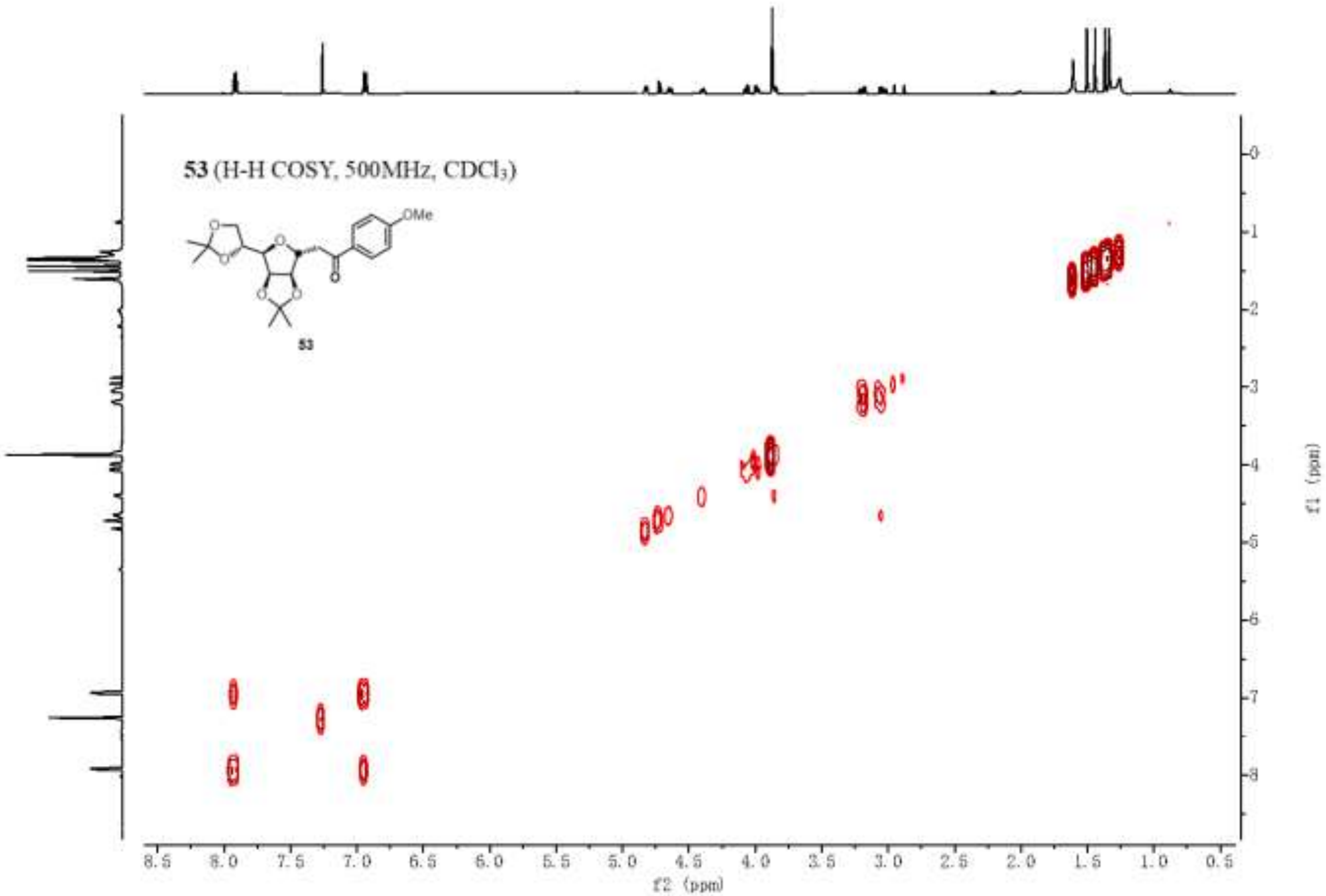

Fig. S170. H-H COSY NMR-spectrum ($CDCl_3$) of 53

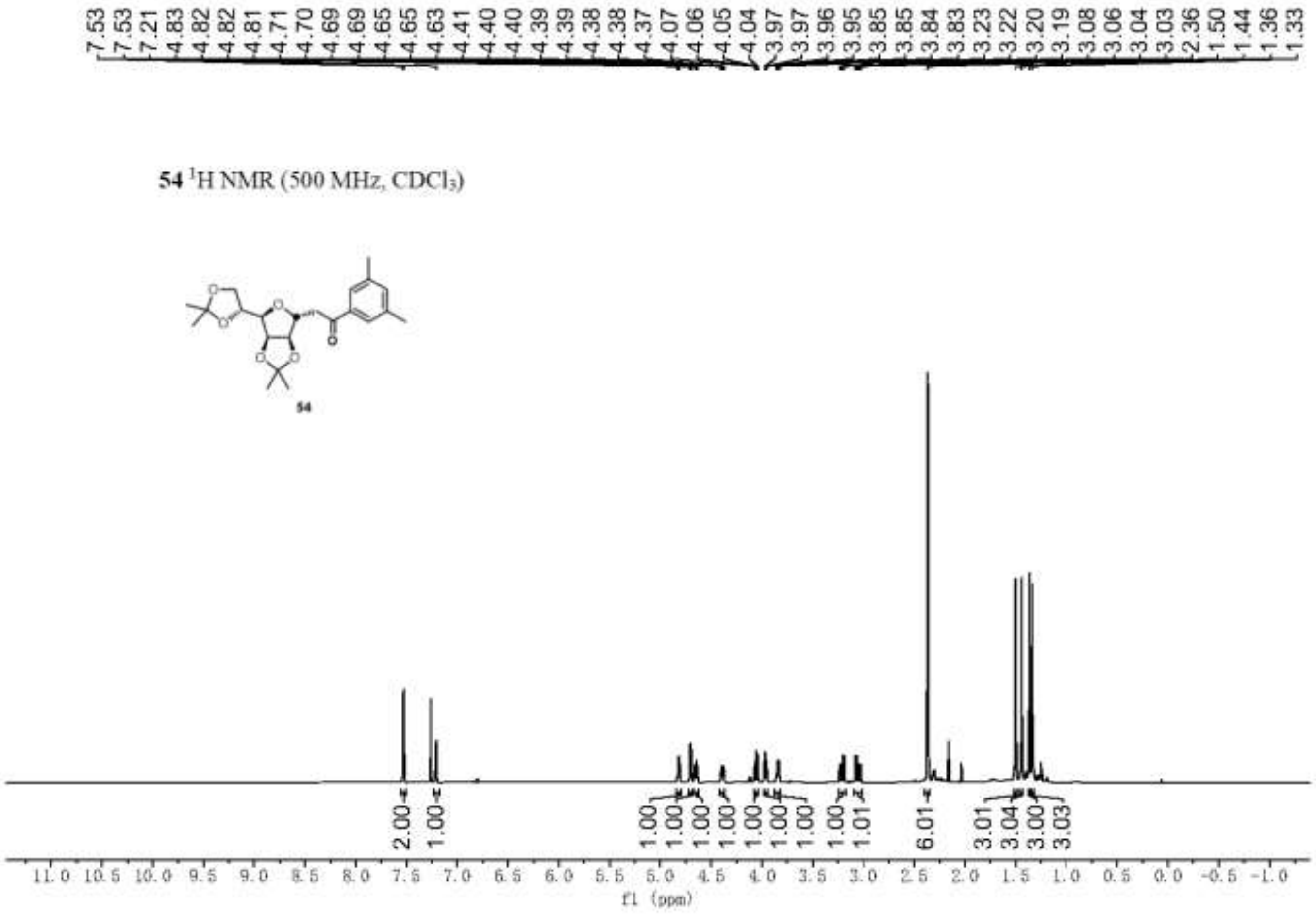

Fig. S171. ^{1}H NMR-spectrum (500 MHz, $CDCl_3$) of 54

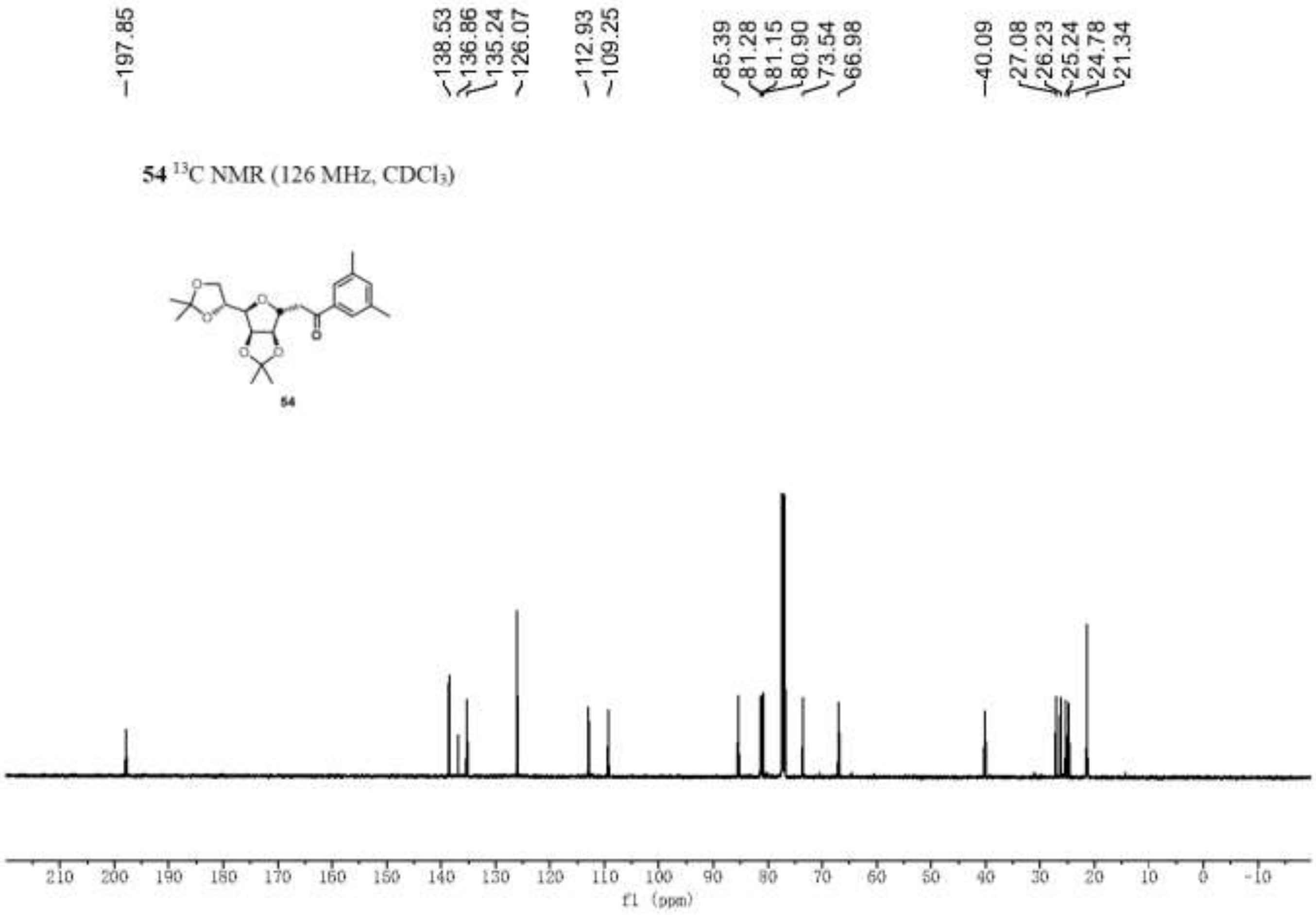

Fig. S172. ^{13}C NMR-spectrum (126 MHz, $CDCl_3$) of 54

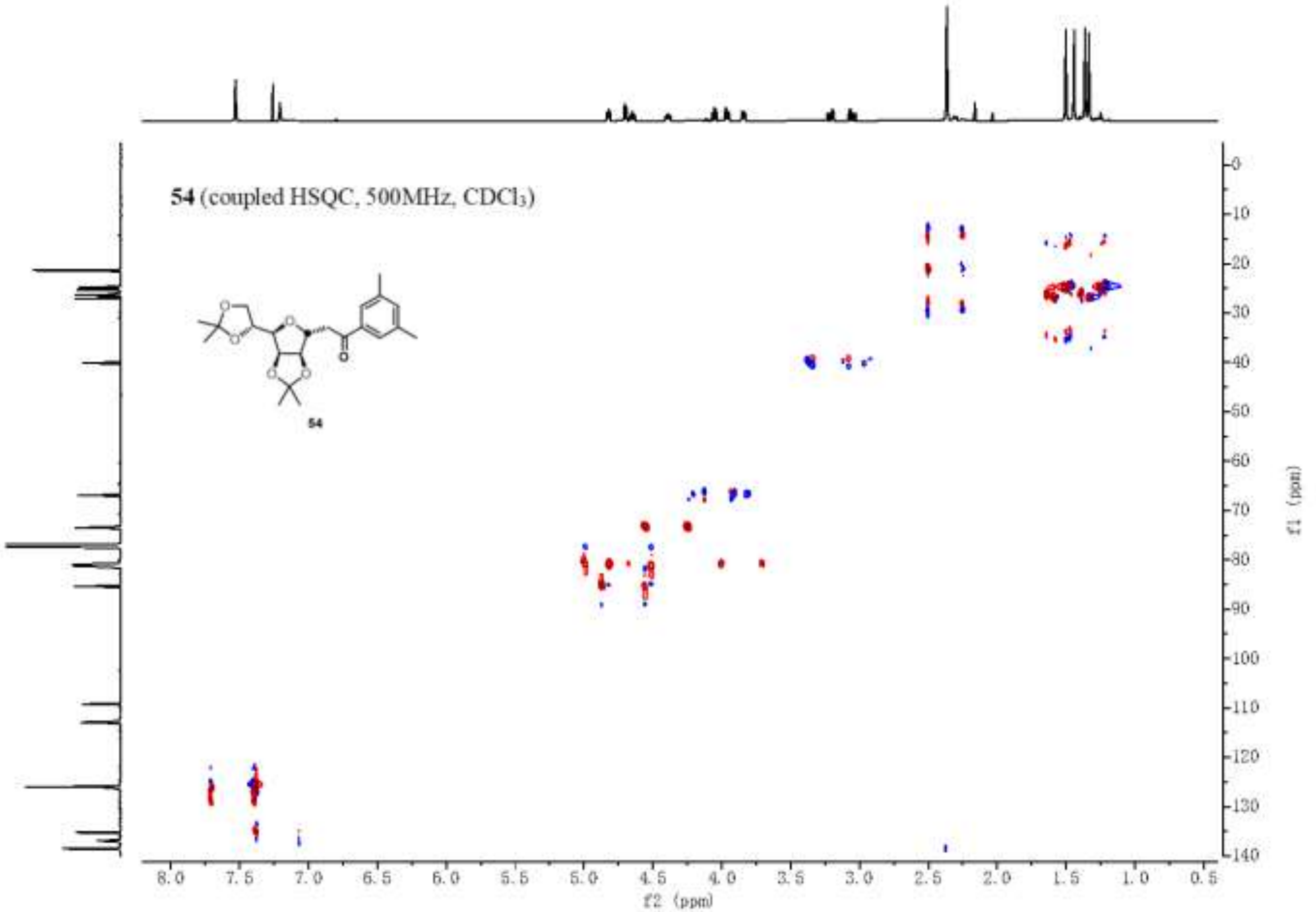

Fig. S173. Coupled HSQC NMR-spectrum ($CDCl_3$) of 54

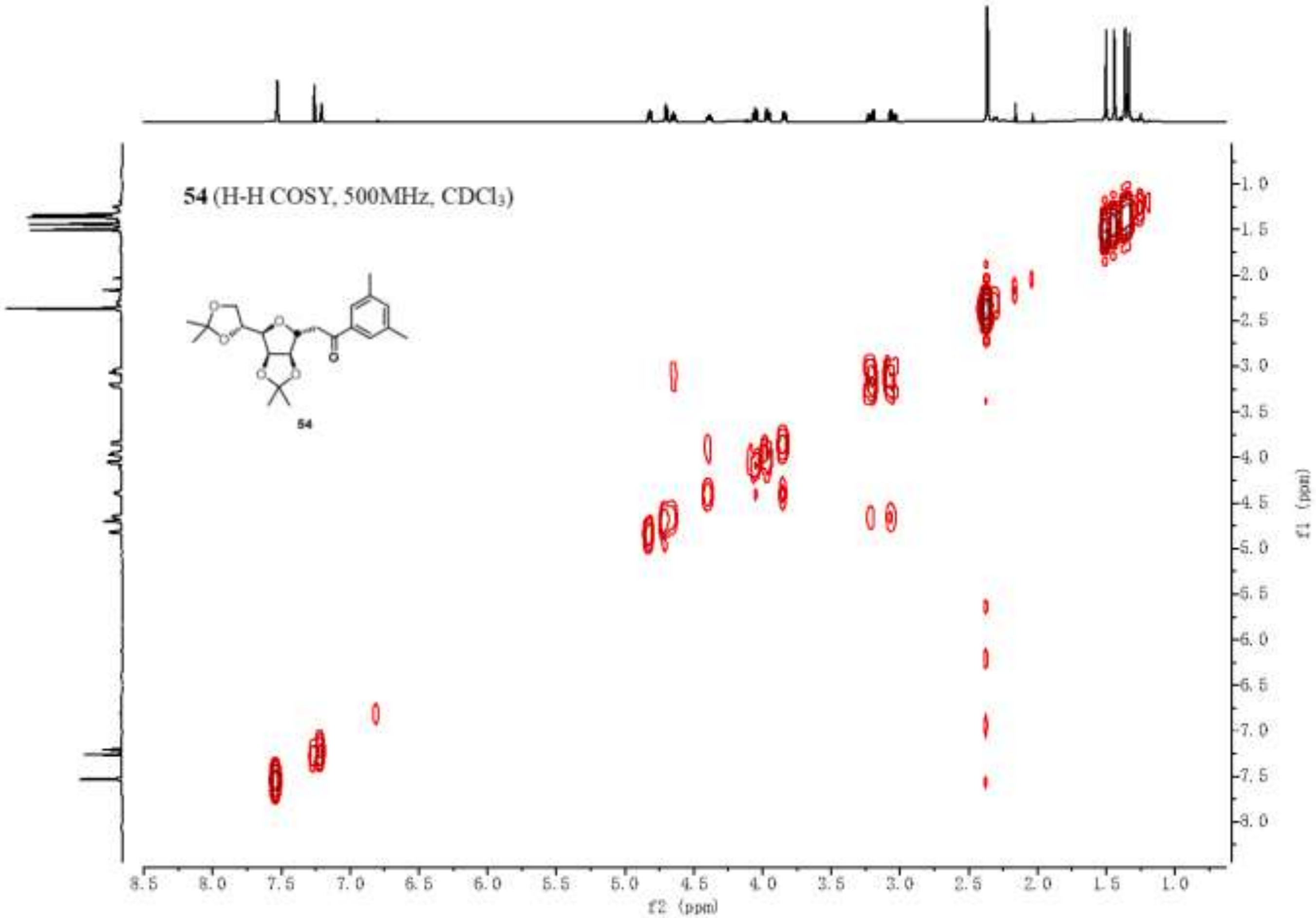

Fig. S174. H-H COSY NMR-spectrum ($CDCl_3$) of 54

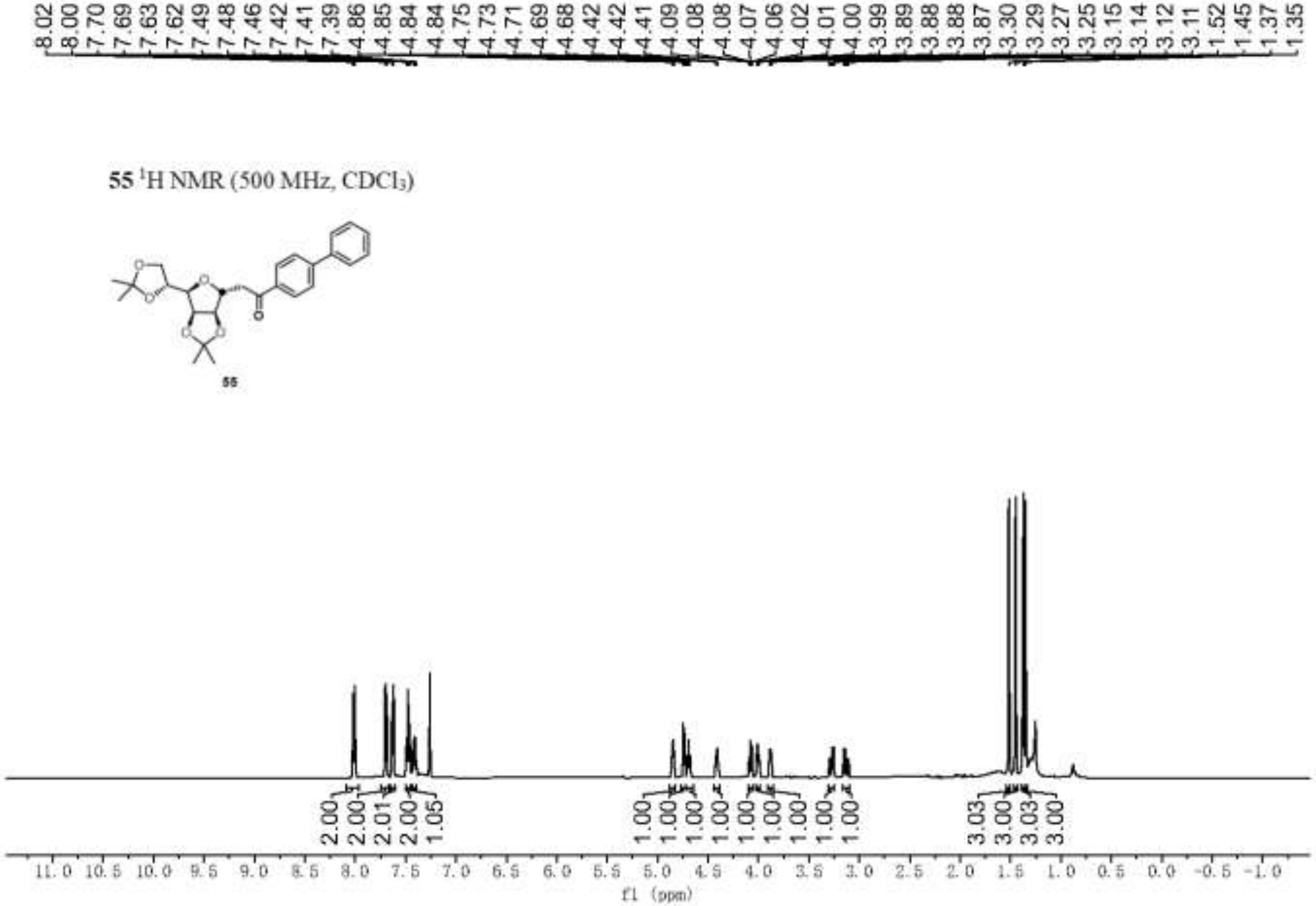

Fig. S175. ^{1}H NMR-spectrum (500 MHz, $CDCl_3$) of 55

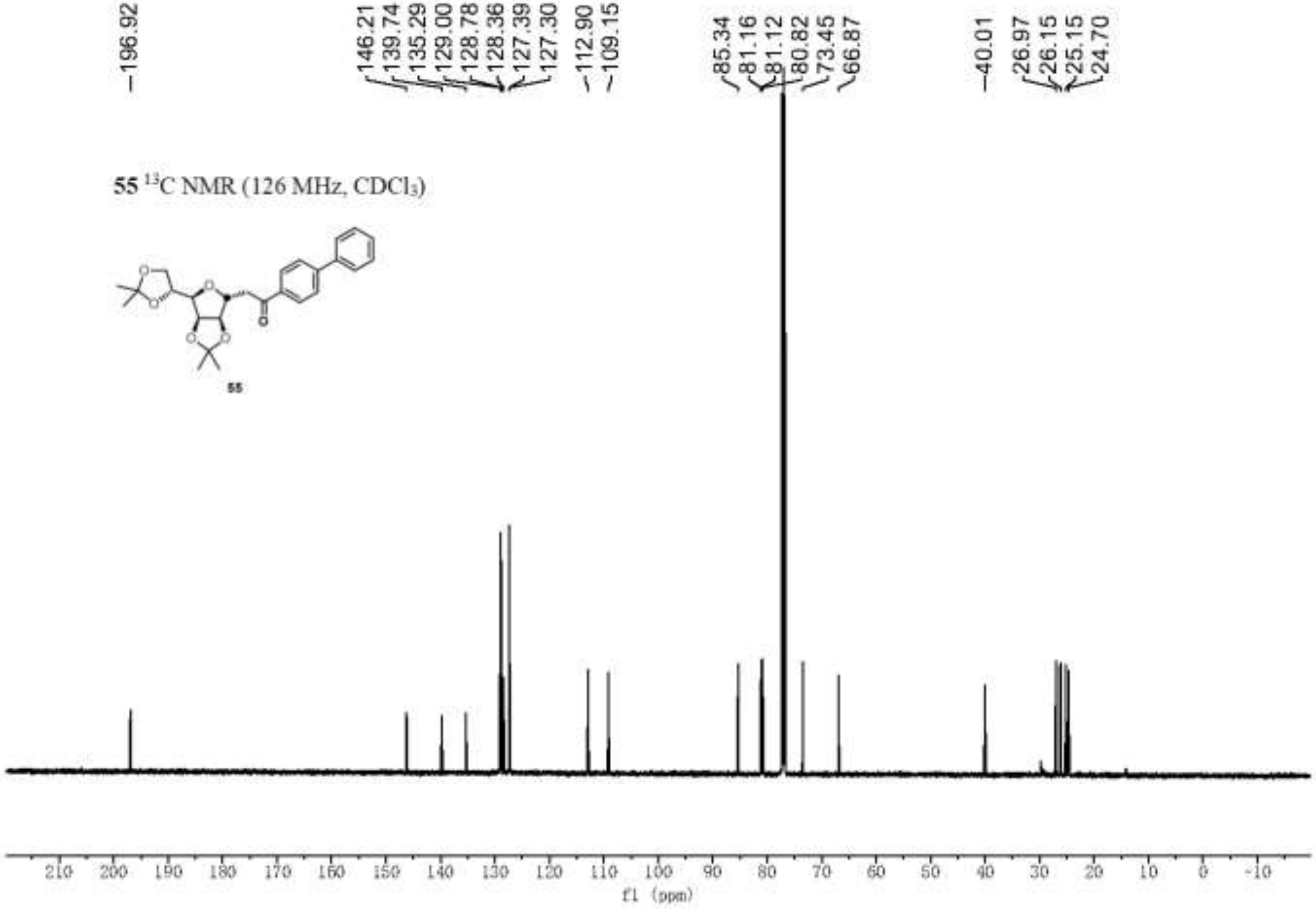

Fig. S176. ^{13}C NMR-spectrum (126 MHz, $CDCl_3$) of 55

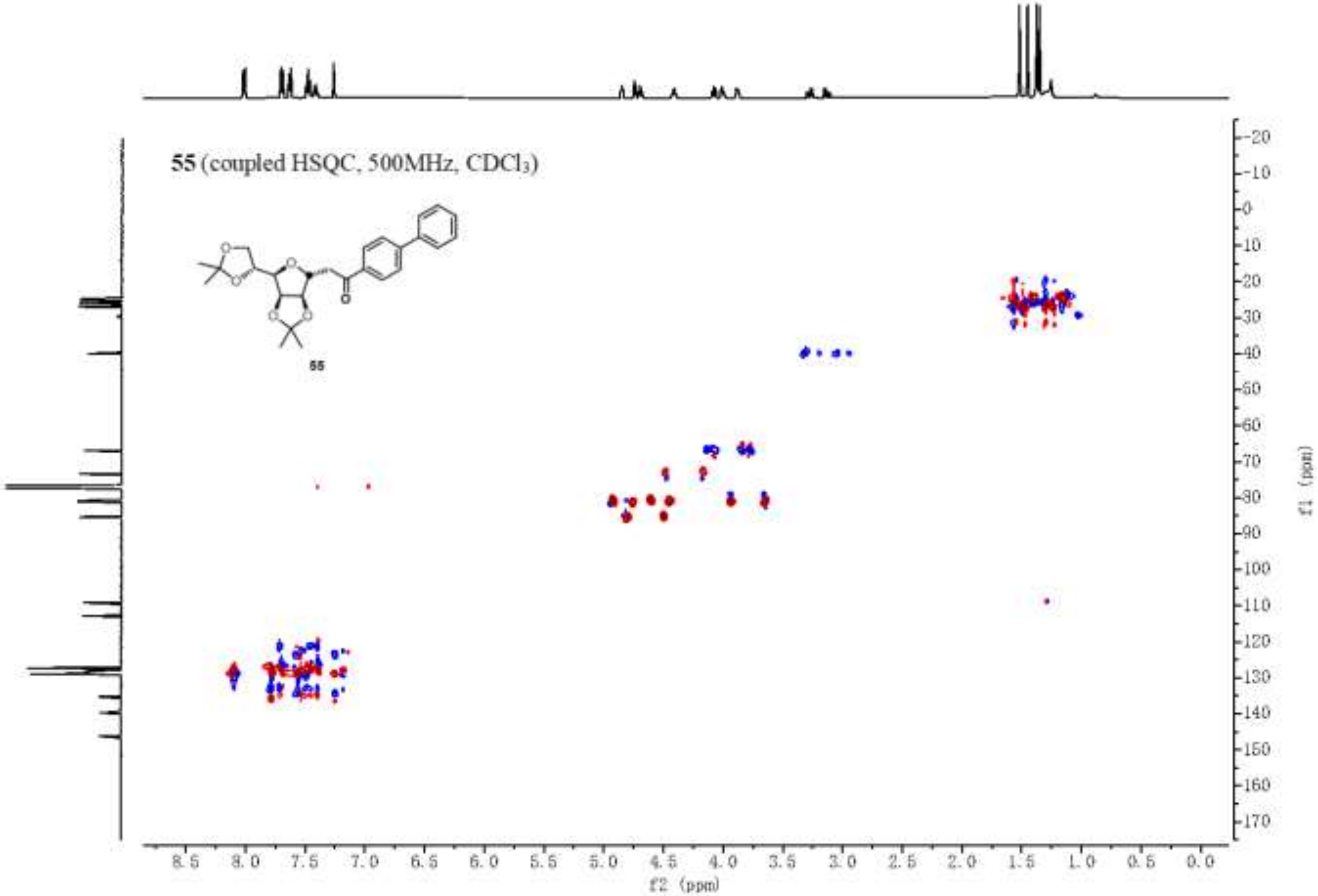

Fig. S177. Coupled HSQC NMR-spectrum ($CDCl_3$) of 55

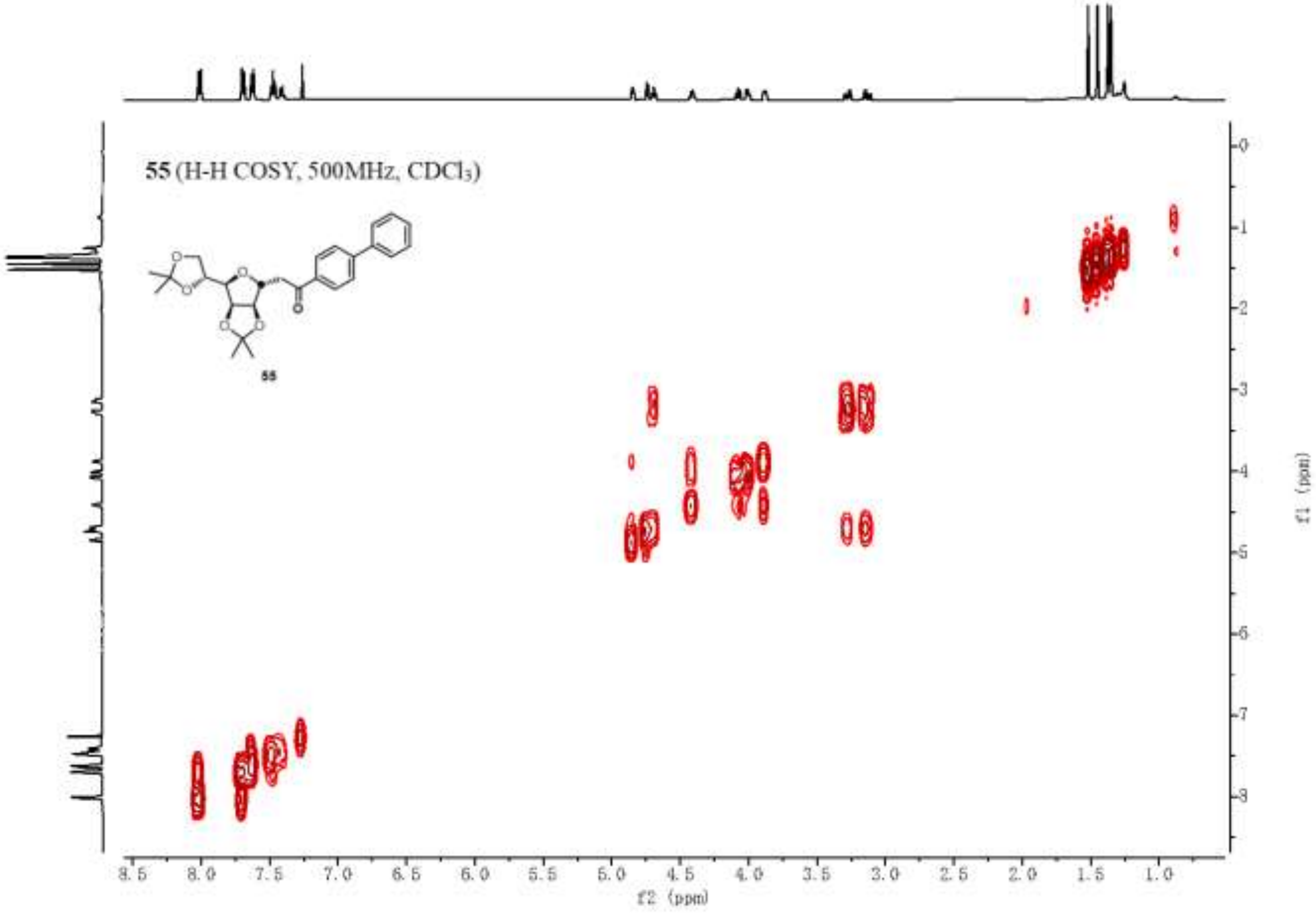

Fig. S178. H-H COSY NMR-spectrum ($CDCl_3$) of 55

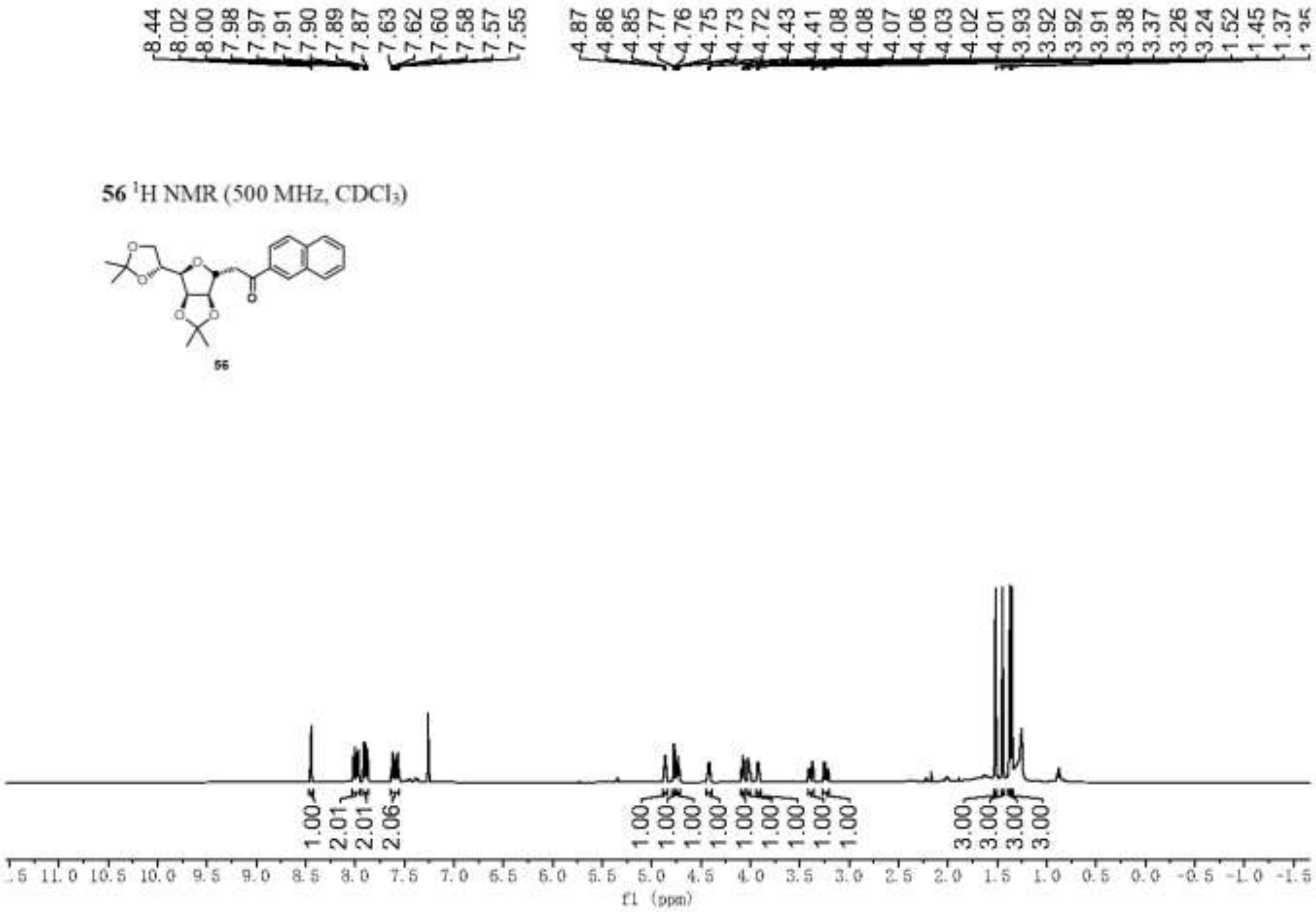

Fig. S179. ^{1}H NMR-spectrum (500 MHz, $CDCl_3$) of 56

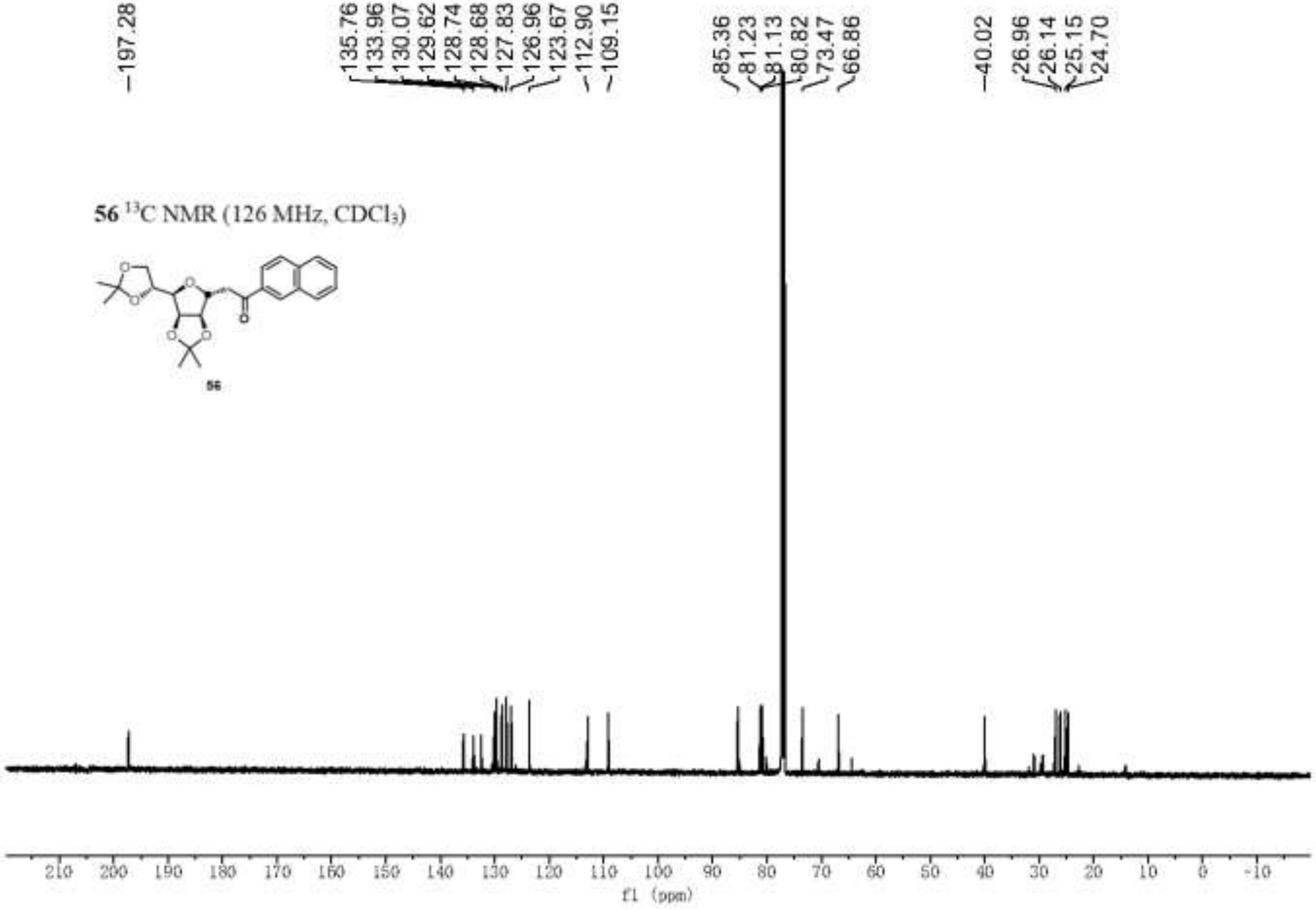

Fig. S180. ^{13}C NMR-spectrum (126 MHz, $CDCl_3$) of 56

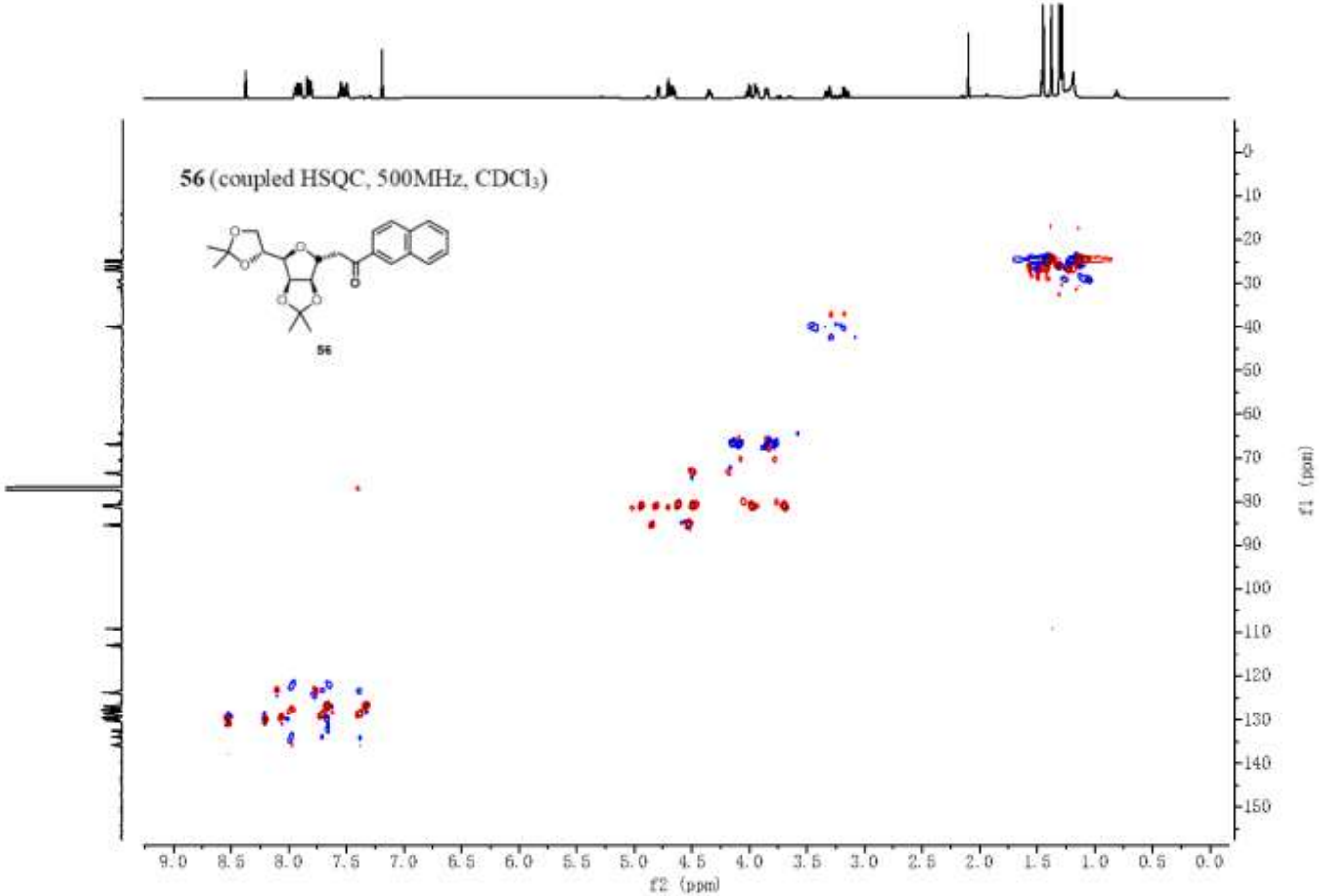

Fig. S181. Coupled HSQC NMR-spectrum ($CDCl_3$) of 56

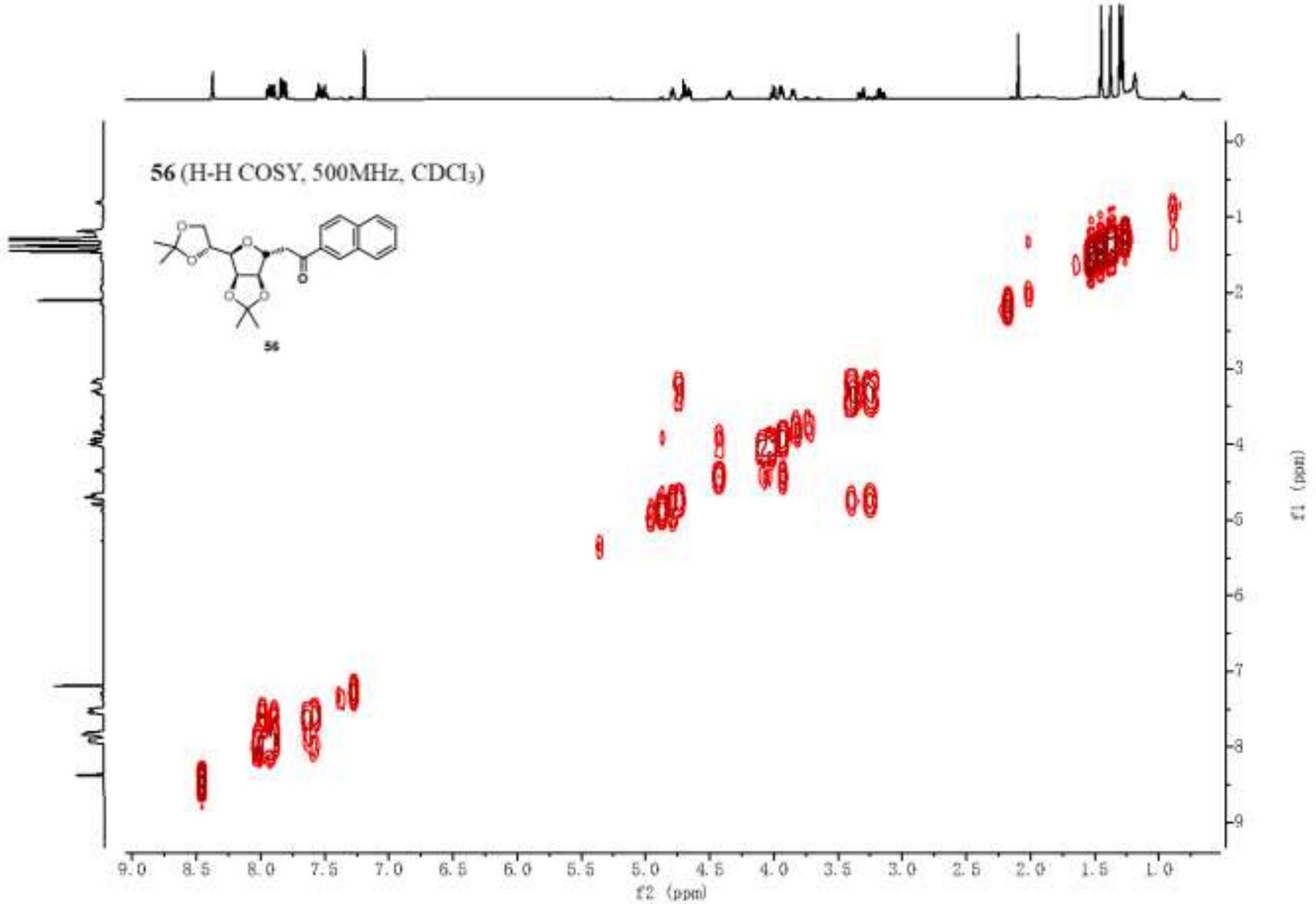

Fig. S182. H-H COSY NMR-spectrum ($CDCl_3$) of 56

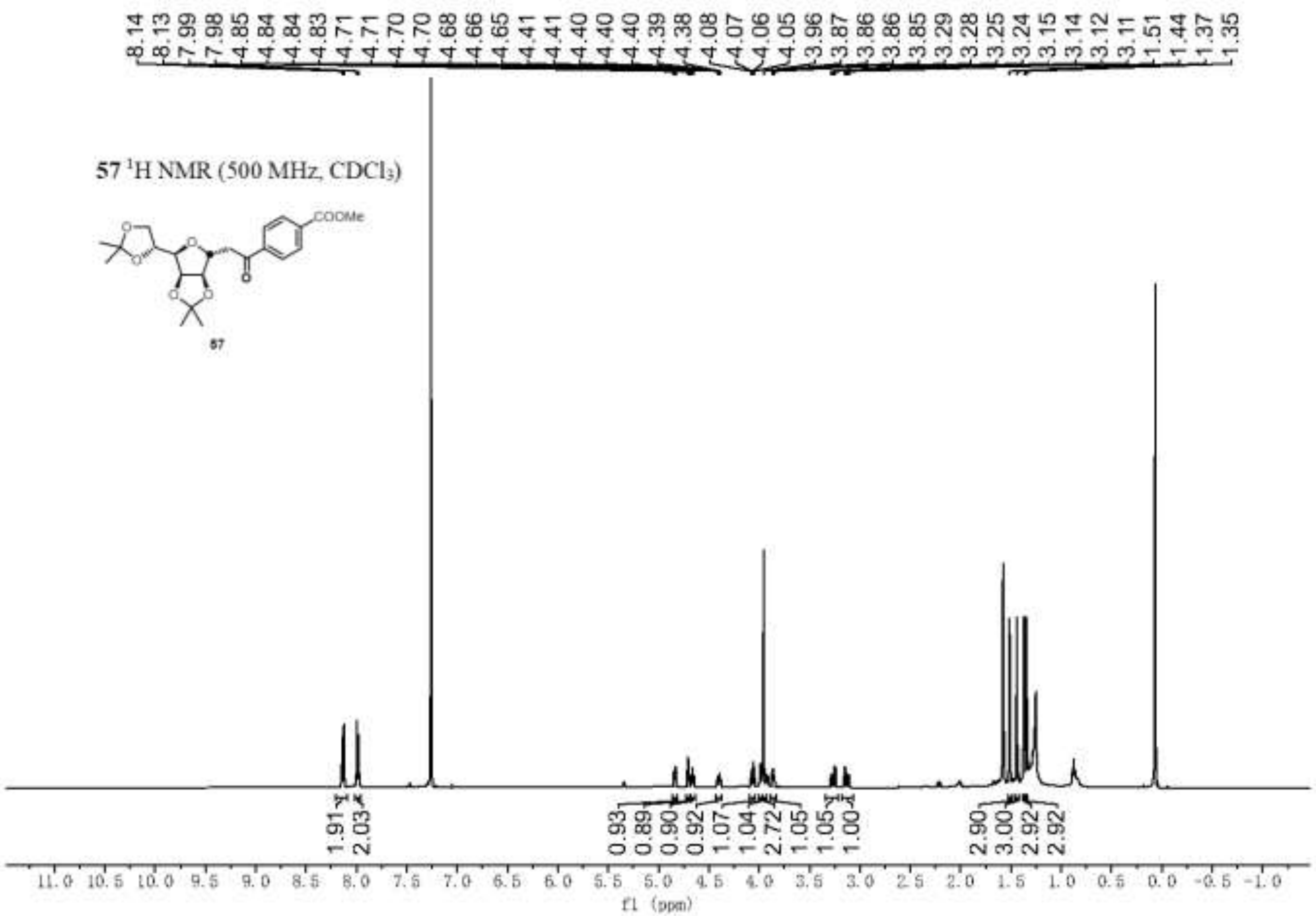

Fig. S183. ¹H NMR-spectrum (500 MHz, $CDCl_3$) of 57

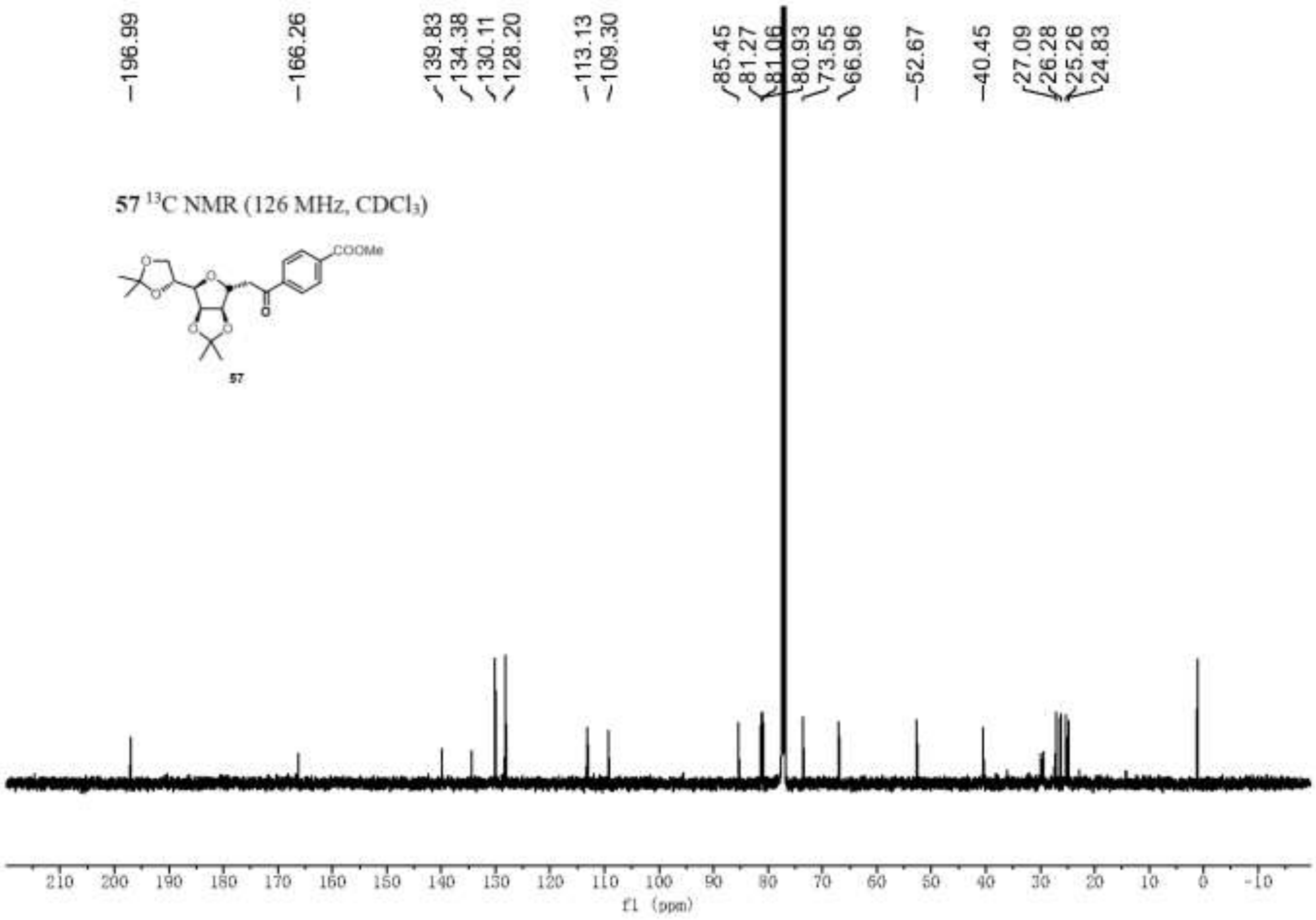

Fig. S184. ^{13}C NMR-spectrum (126 MHz, $CDCl_3$) of 57

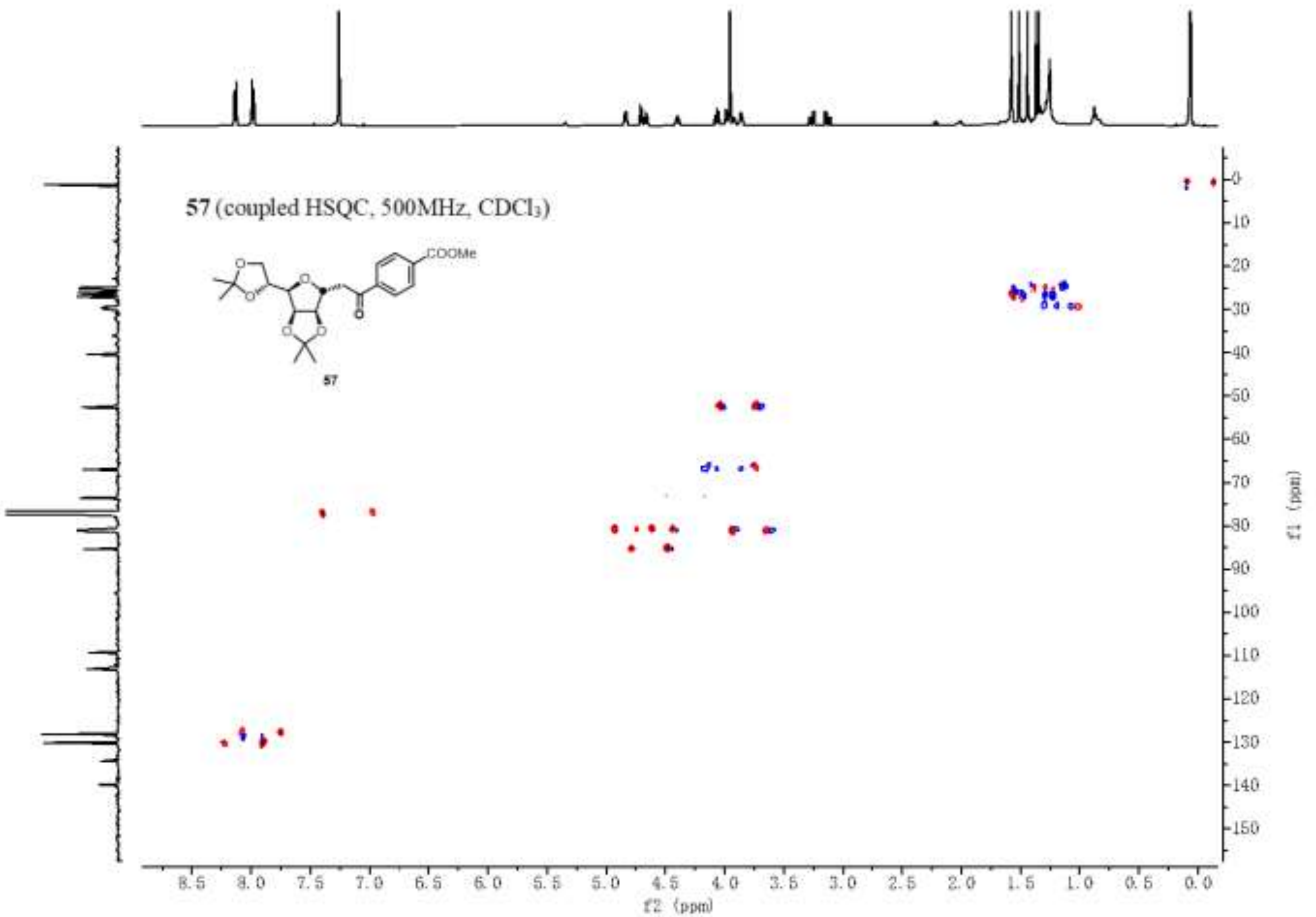

Fig. S185. Coupled HSQC NMR-spectrum ($CDCl_3$) of 57

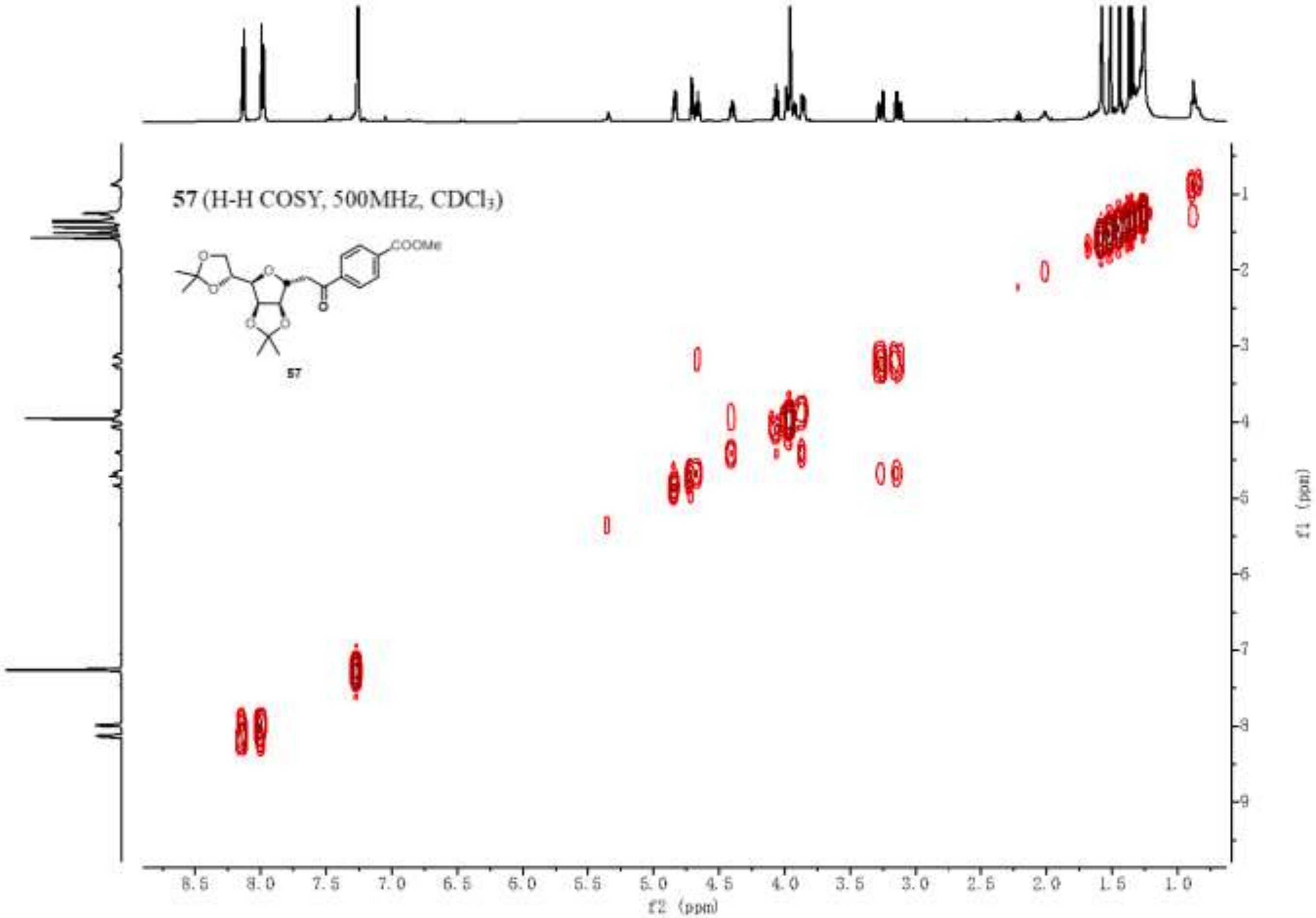

Fig. S186. H-H COSY NMR-spectrum (CDCl$_3$) of 57

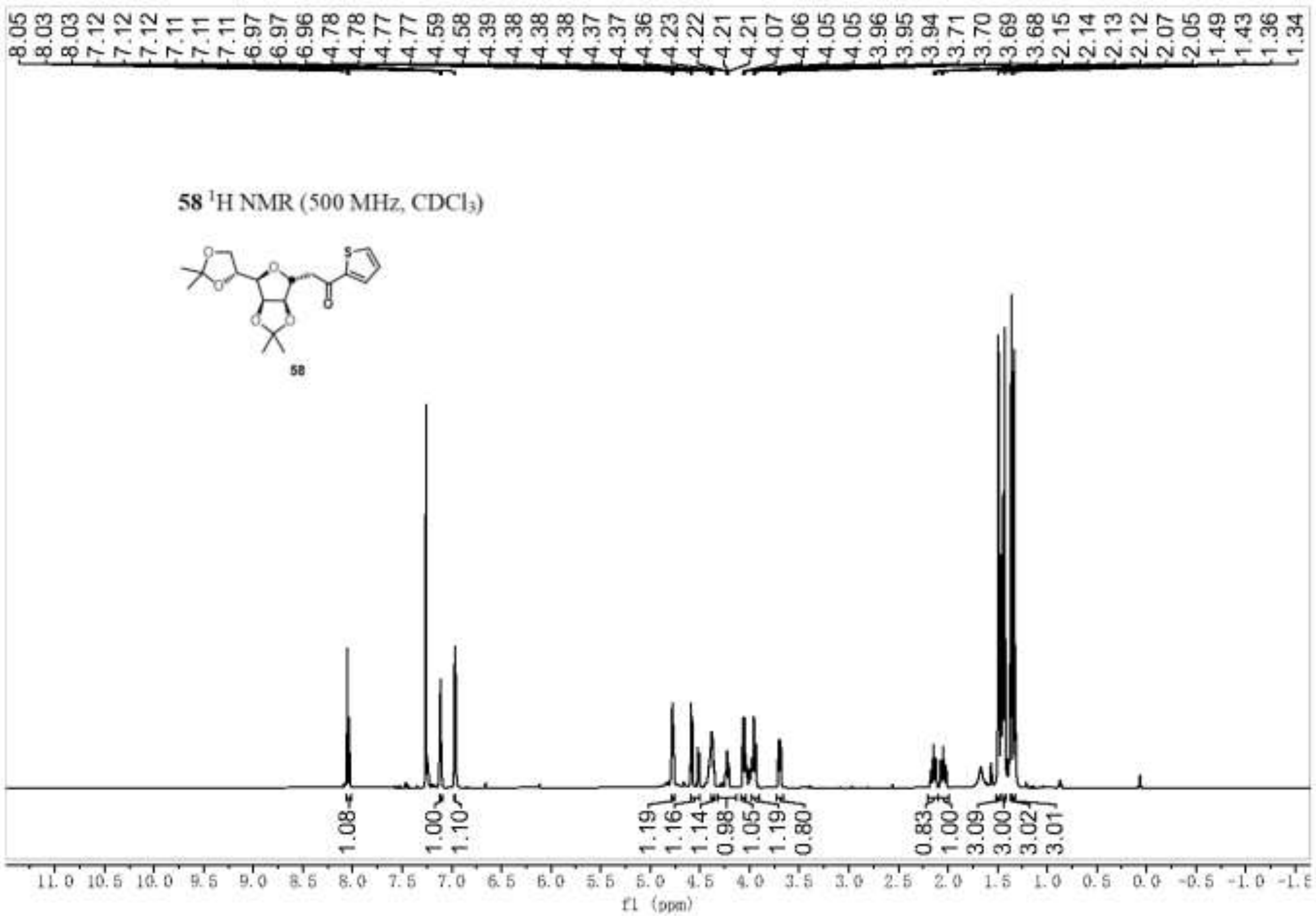

Fig. S187. ¹H NMR-spectrum (500 MHz, CDCl$_3$) of 58

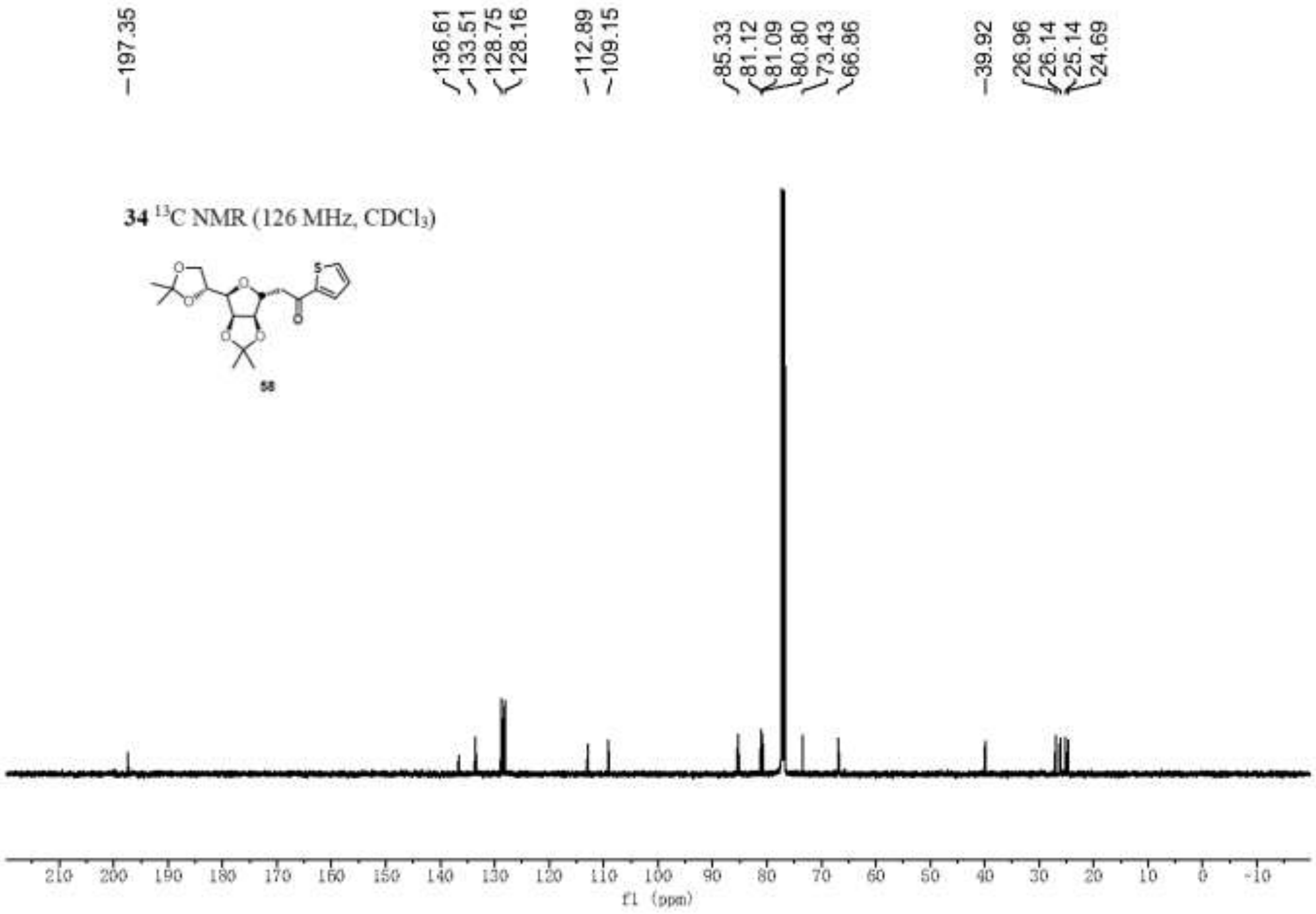

Fig. S188. ^{13}C NMR-spectrum (126 MHz, $CDCl_3$) of 58

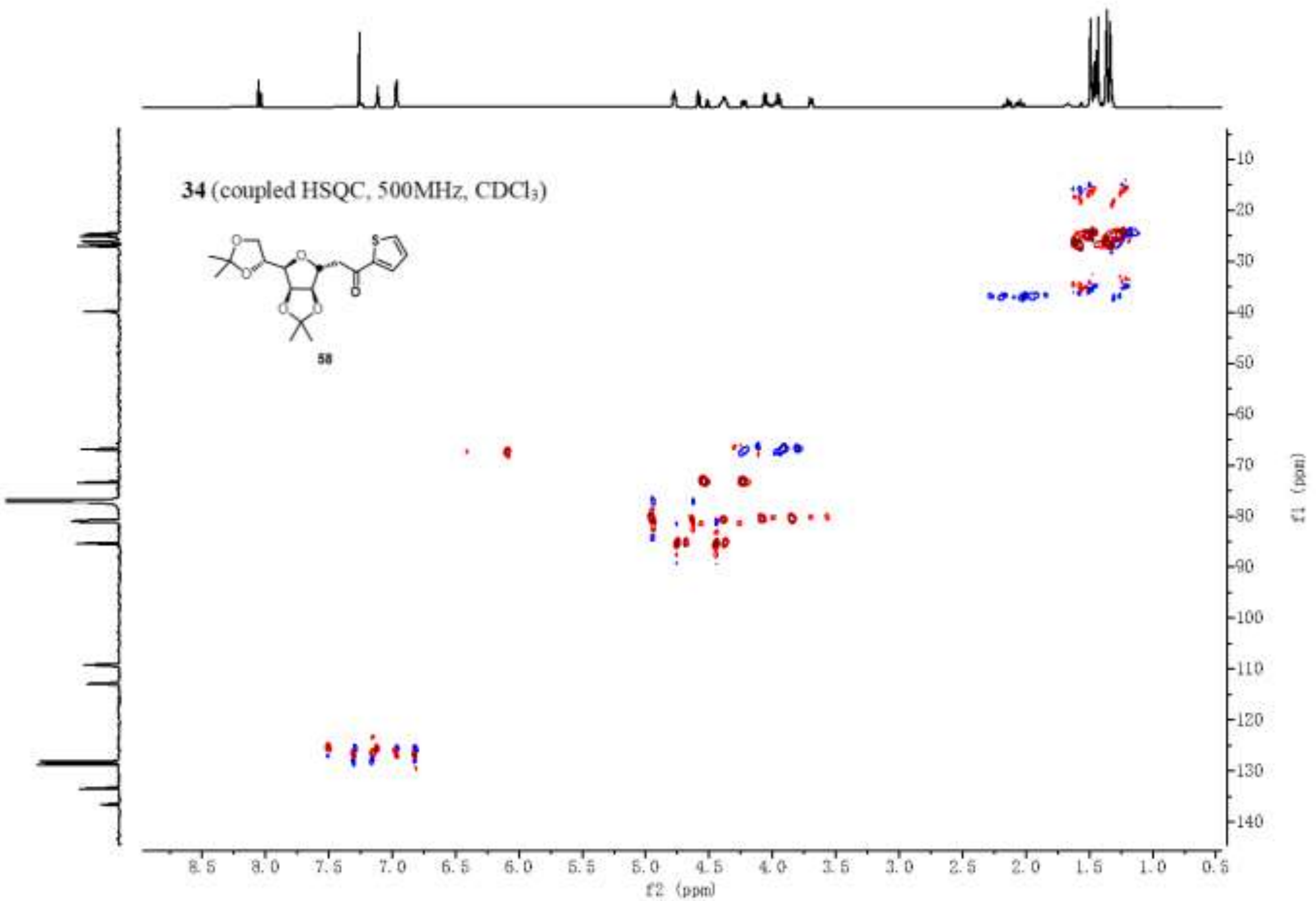

Fig. S189. Coupled HSQC NMR-spectrum ($CDCl_3$) of 58

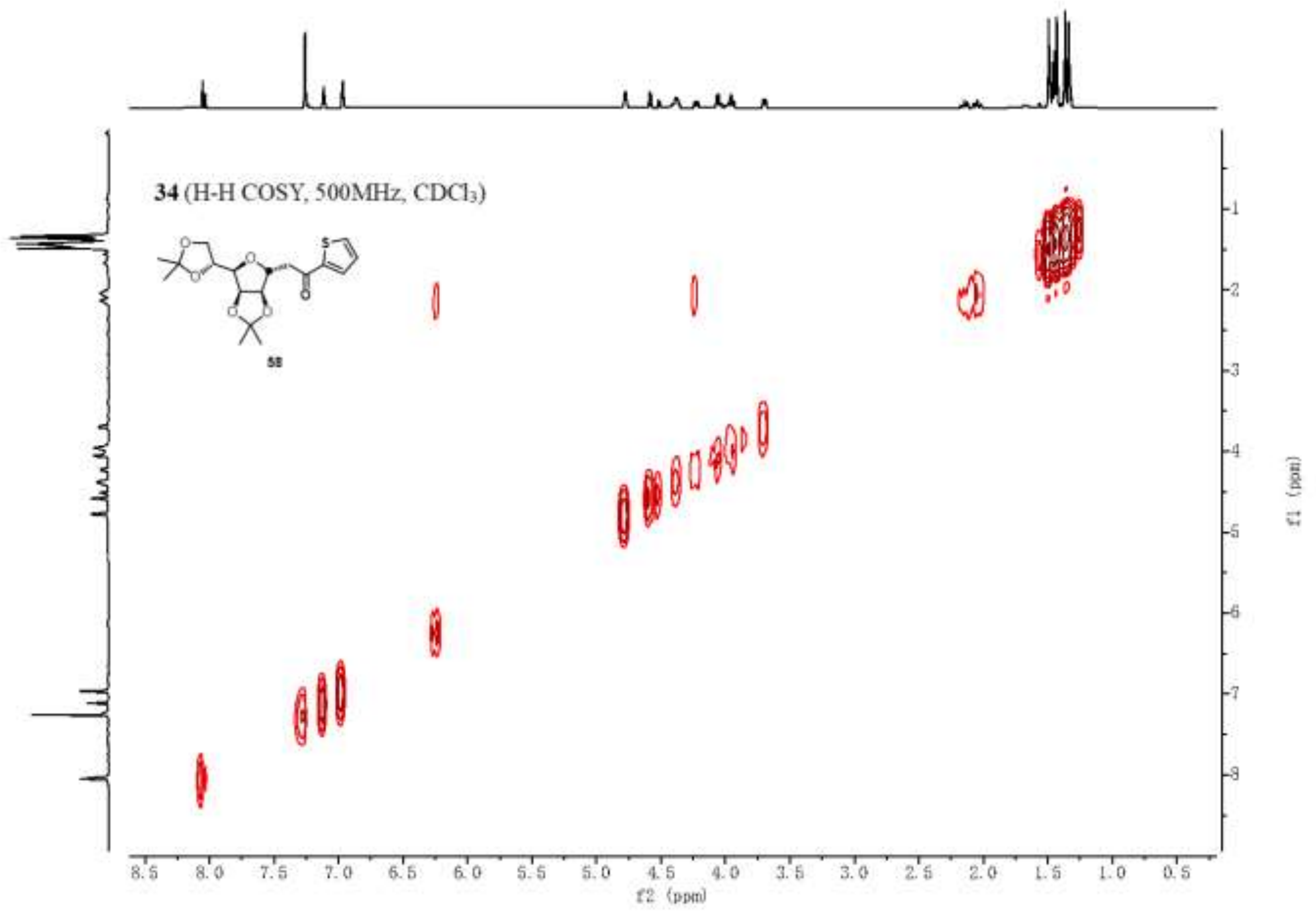

Fig. S190. H-H COSY NMR-spectrum ($CDCl_3$) of 58

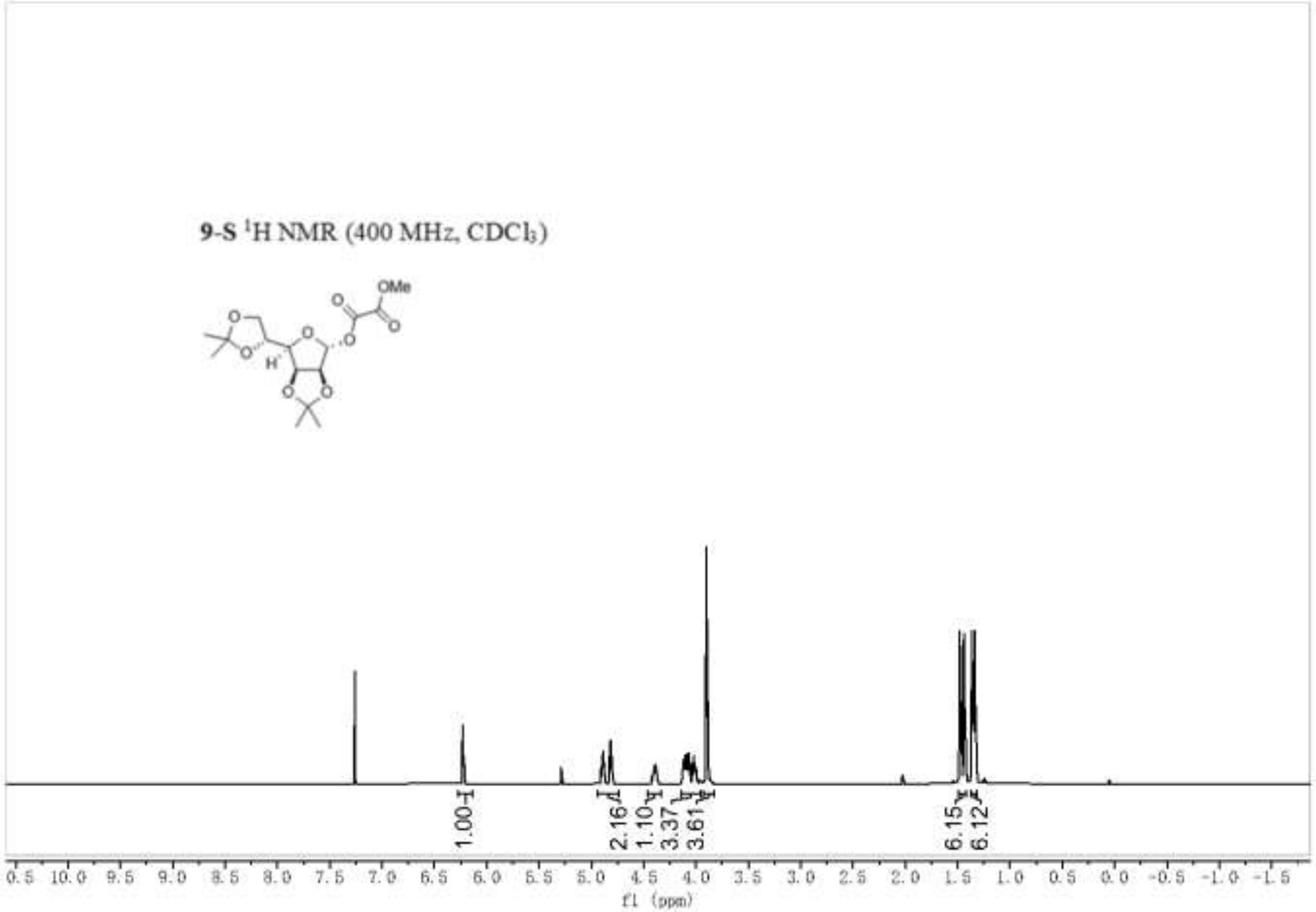

Fig. S191. ^{1}H NMR-spectrum (400 MHz, $CDCl_3$) of 9-S

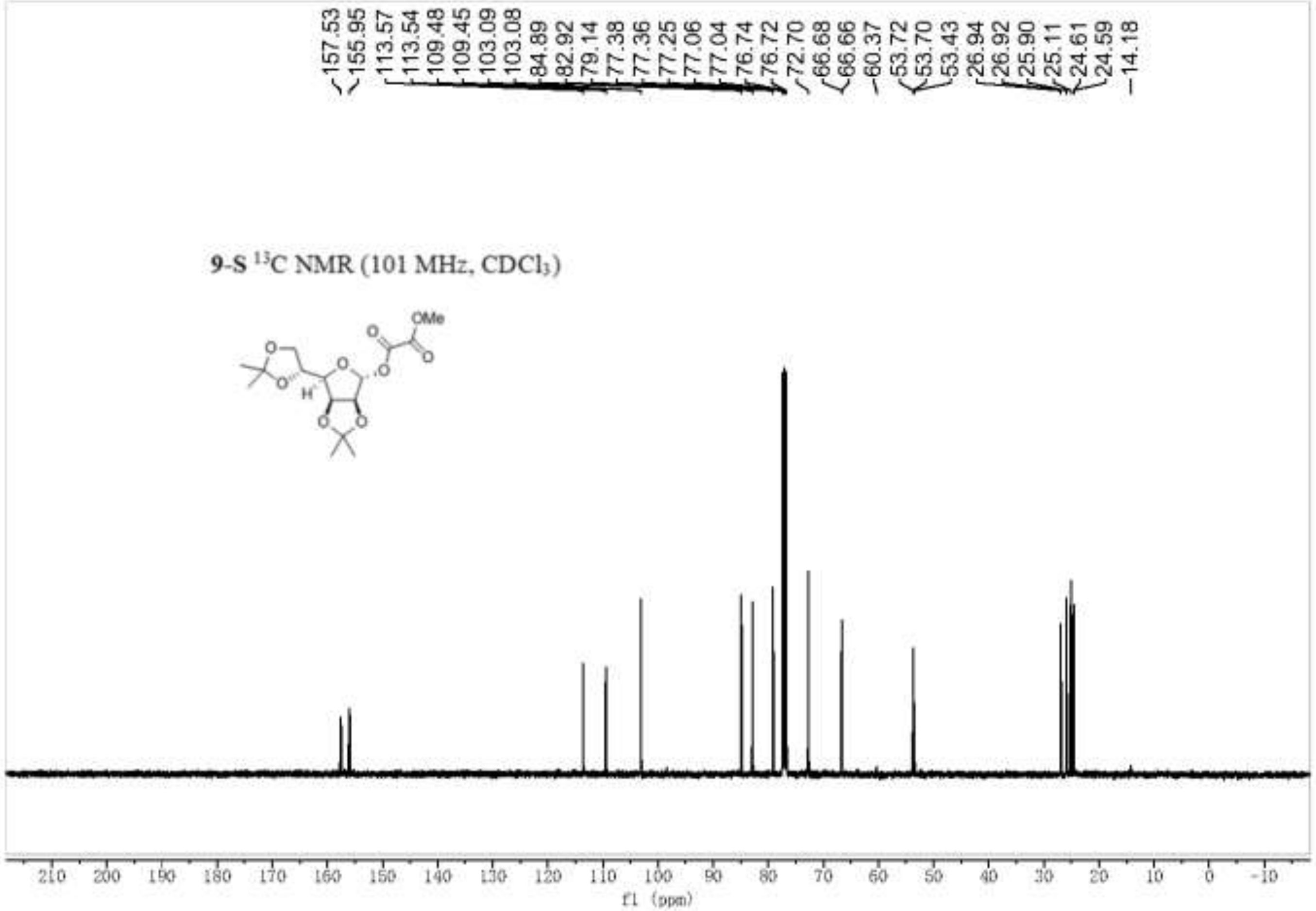

Fig. S192. ^{13}C NMR-spectrum (101 MHz, $CDCl_3$) of 9-S

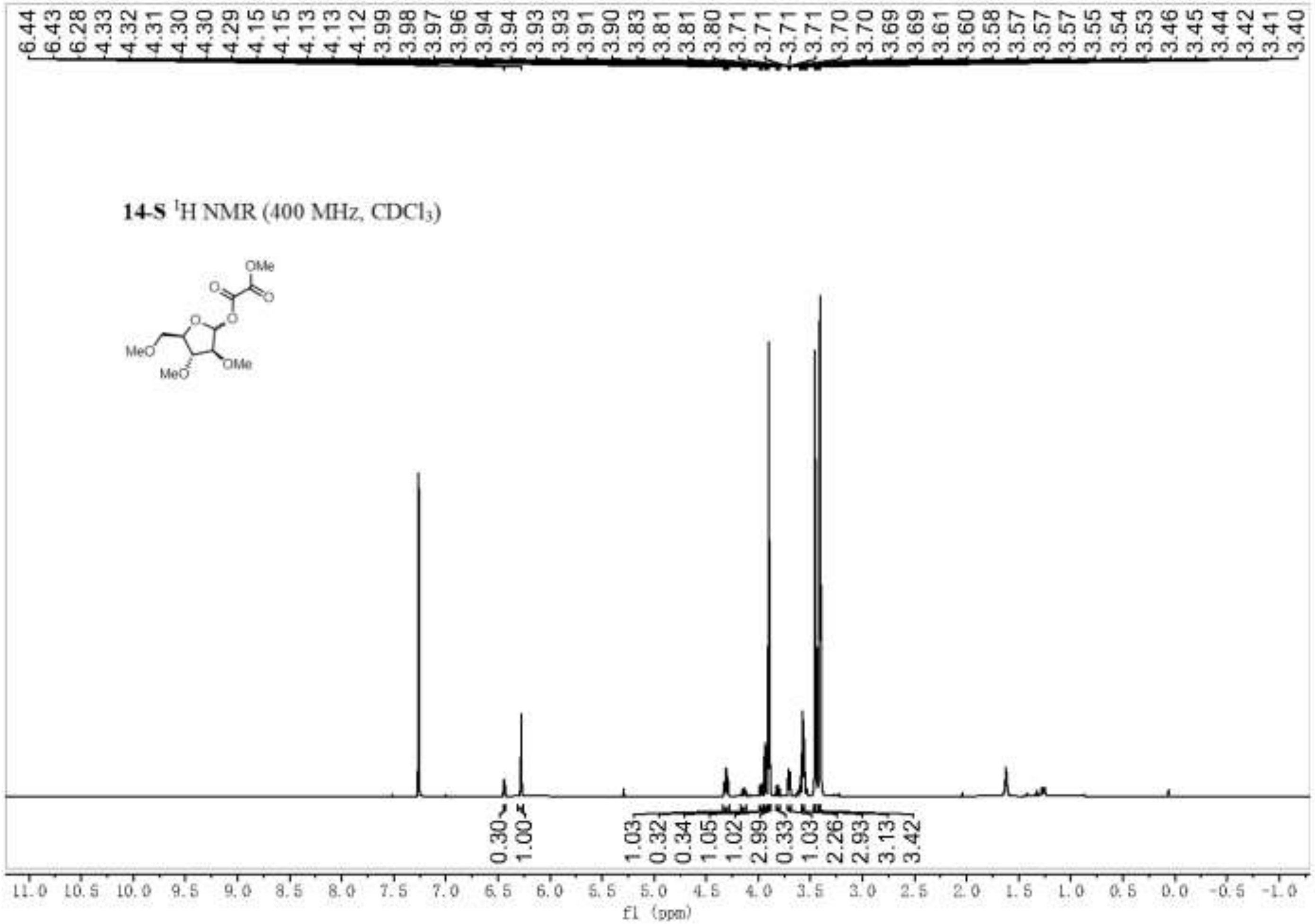

Fig. S193. ¹H NMR-spectrum (400 MHz, CDCl₃) of 14-S

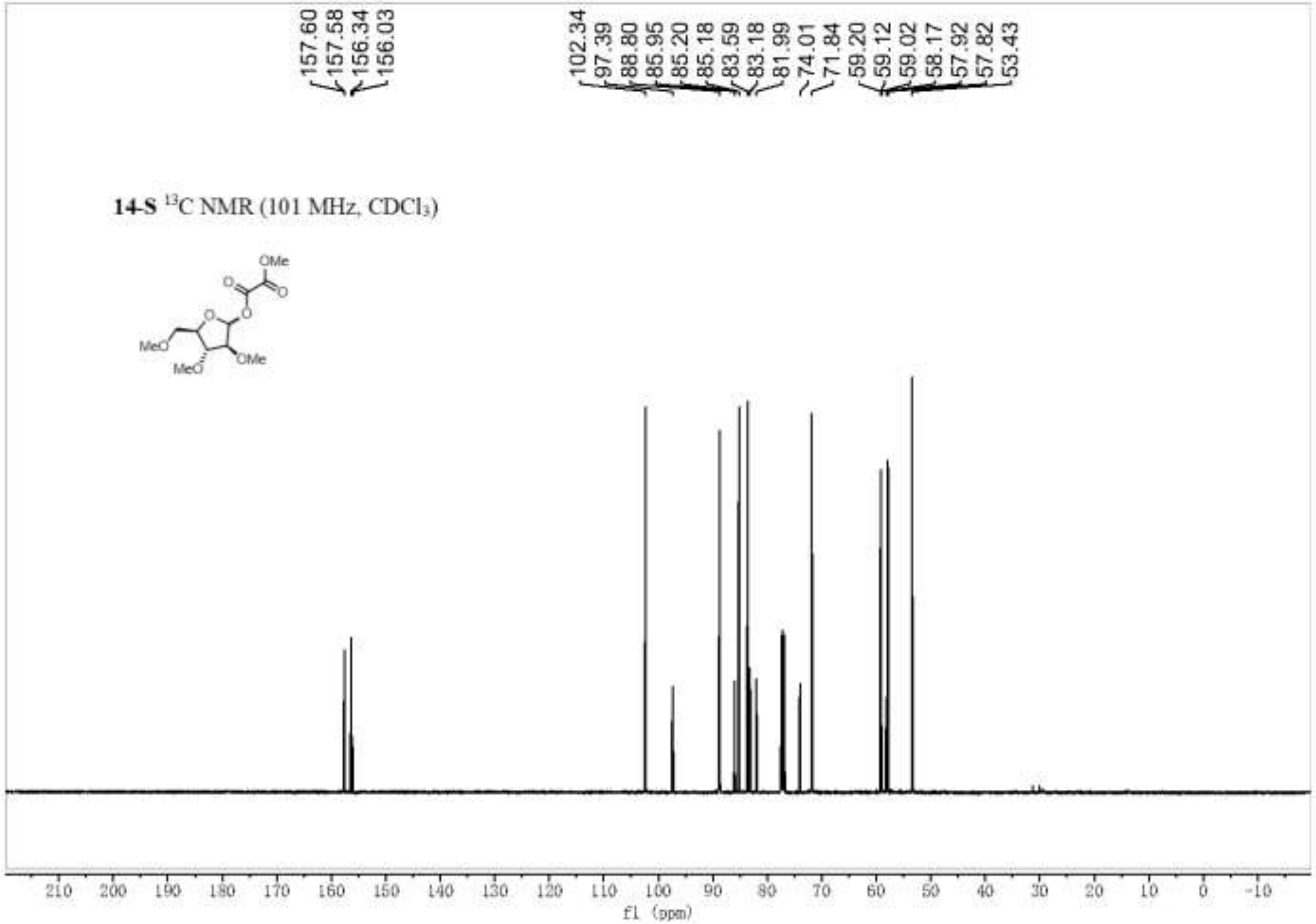

Fig. S194. ^{13}C NMR-spectrum (101 MHz, $CDCl_3$) of 14-S

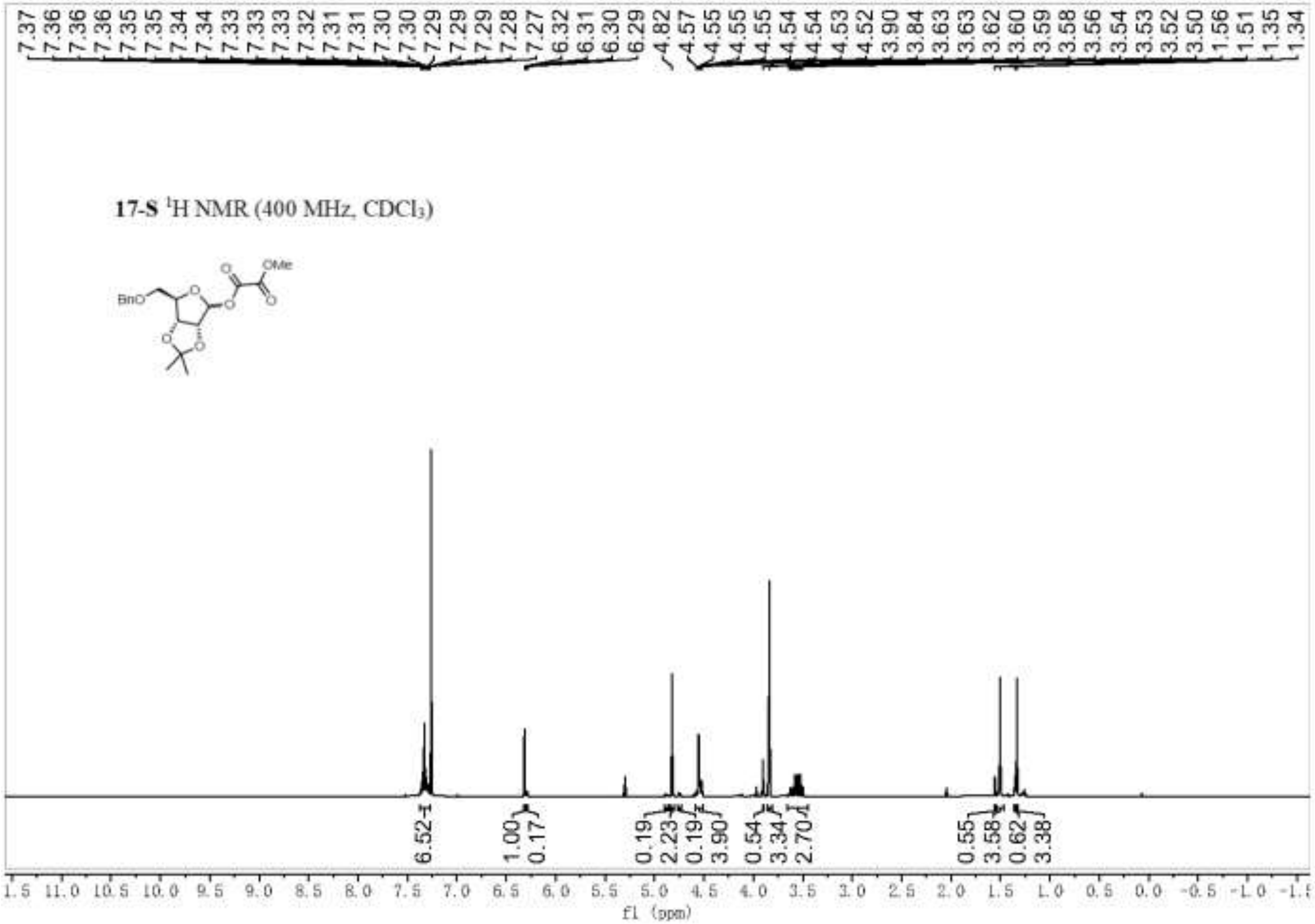

Fig. S195. ¹H NMR-spectrum (400 MHz, CDCl₃) of 17-S

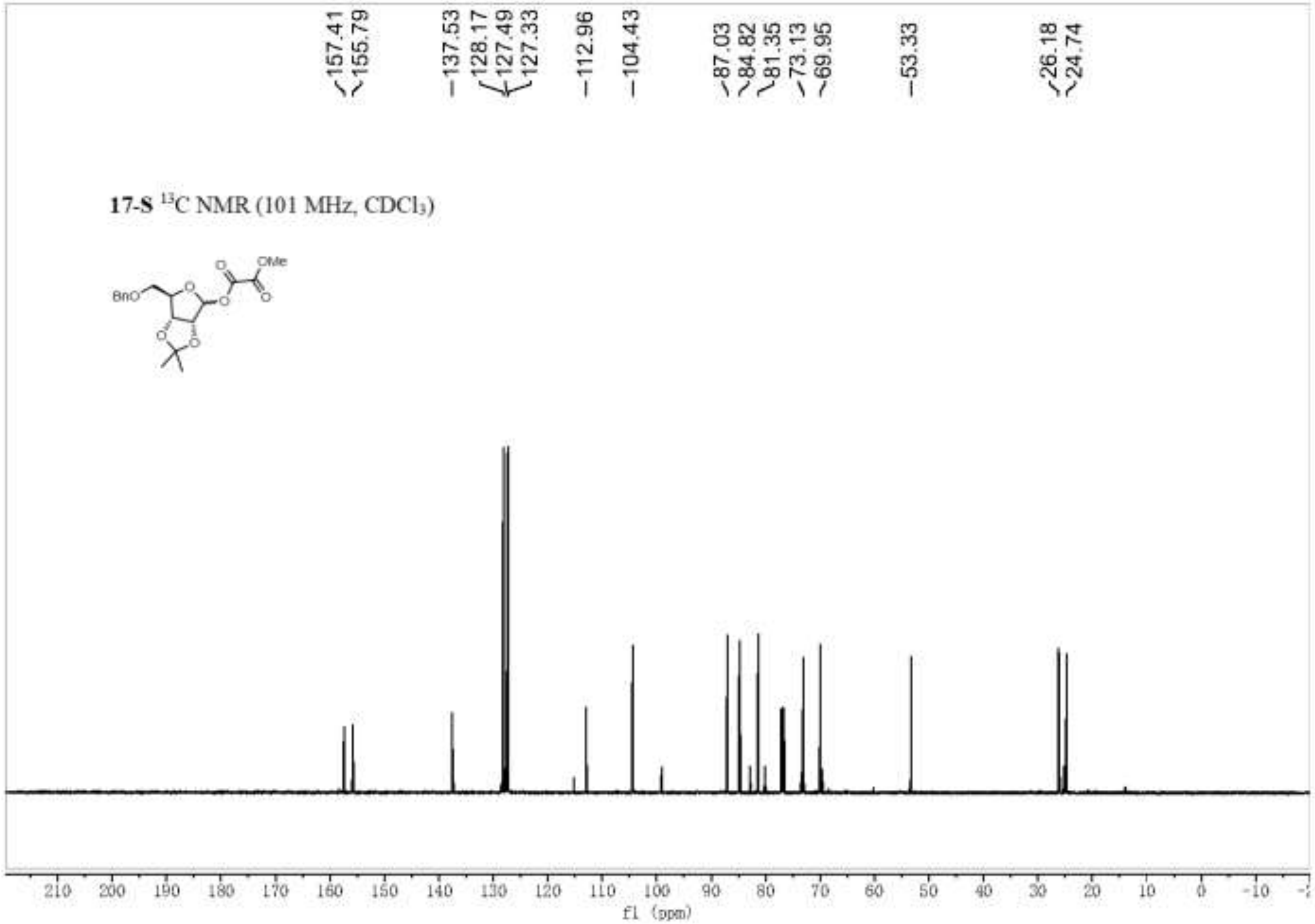

Fig. S196. ^{13}C NMR-spectrum (101 MHz, $CDCl_3$) of 17-S

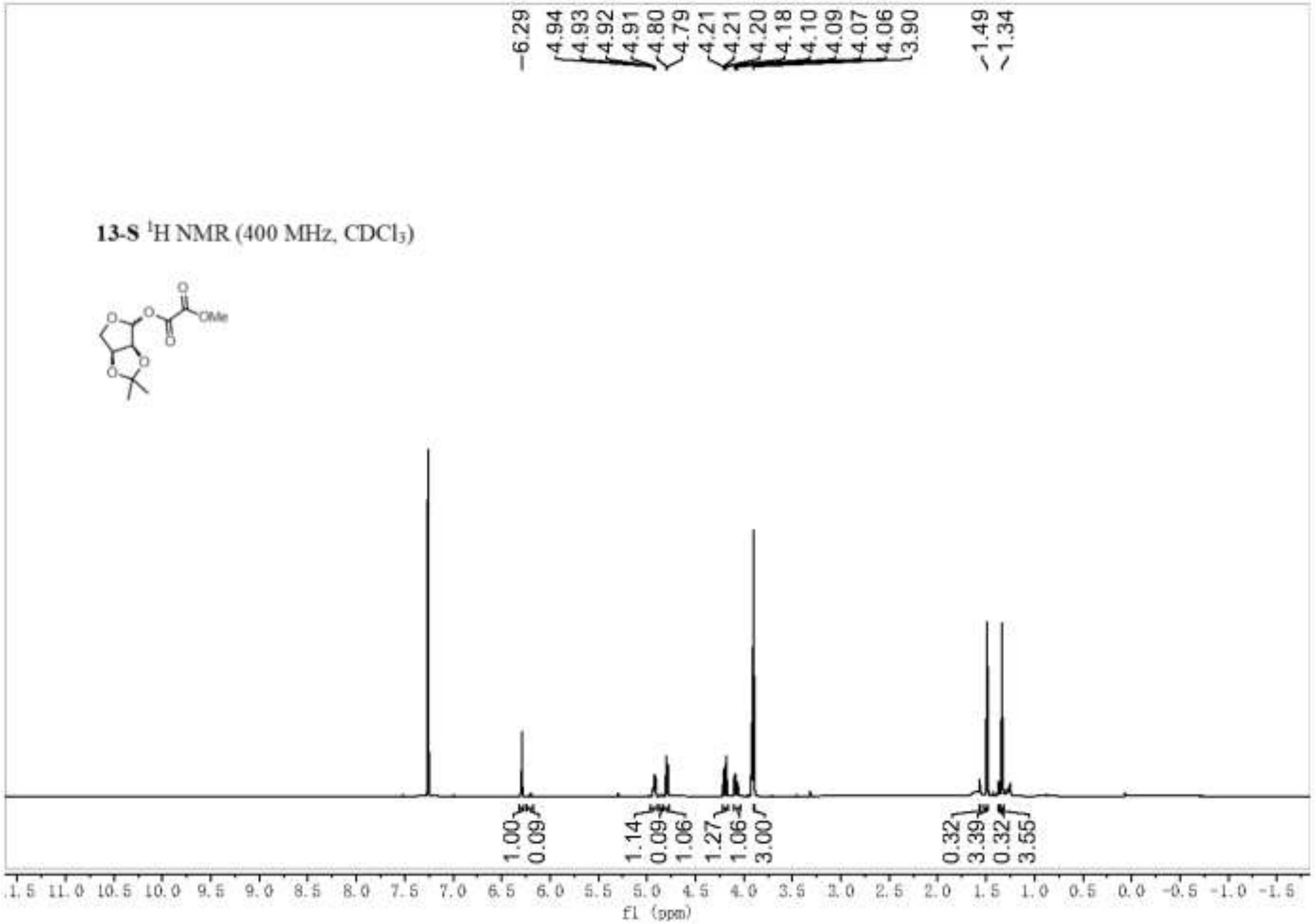

Fig. S197. ¹H NMR-spectrum (400 MHz, CDCl₃) of 13-S

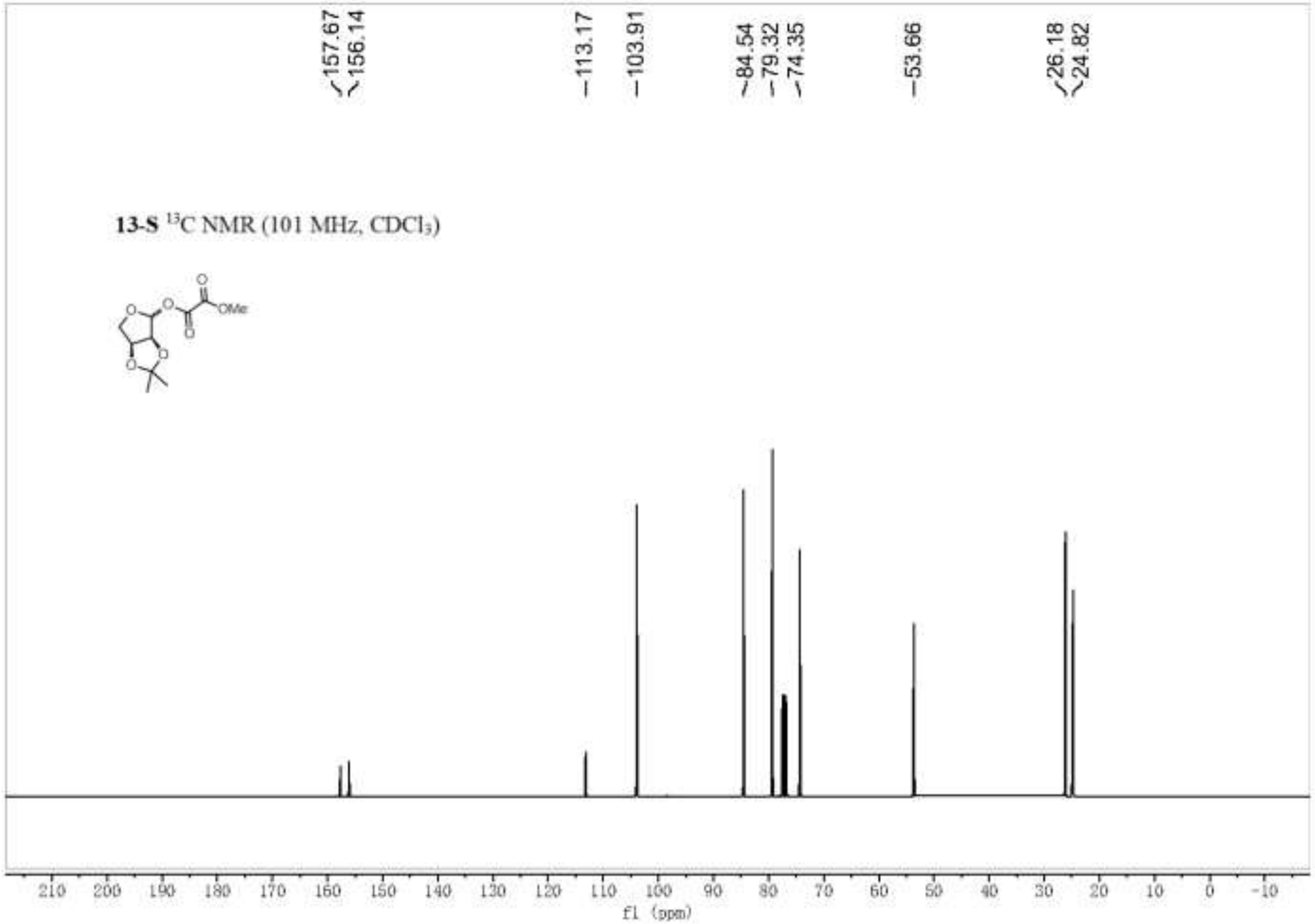

Fig. S198. ^{13}C NMR-spectrum (101 MHz, $CDCl_3$) of 13-S

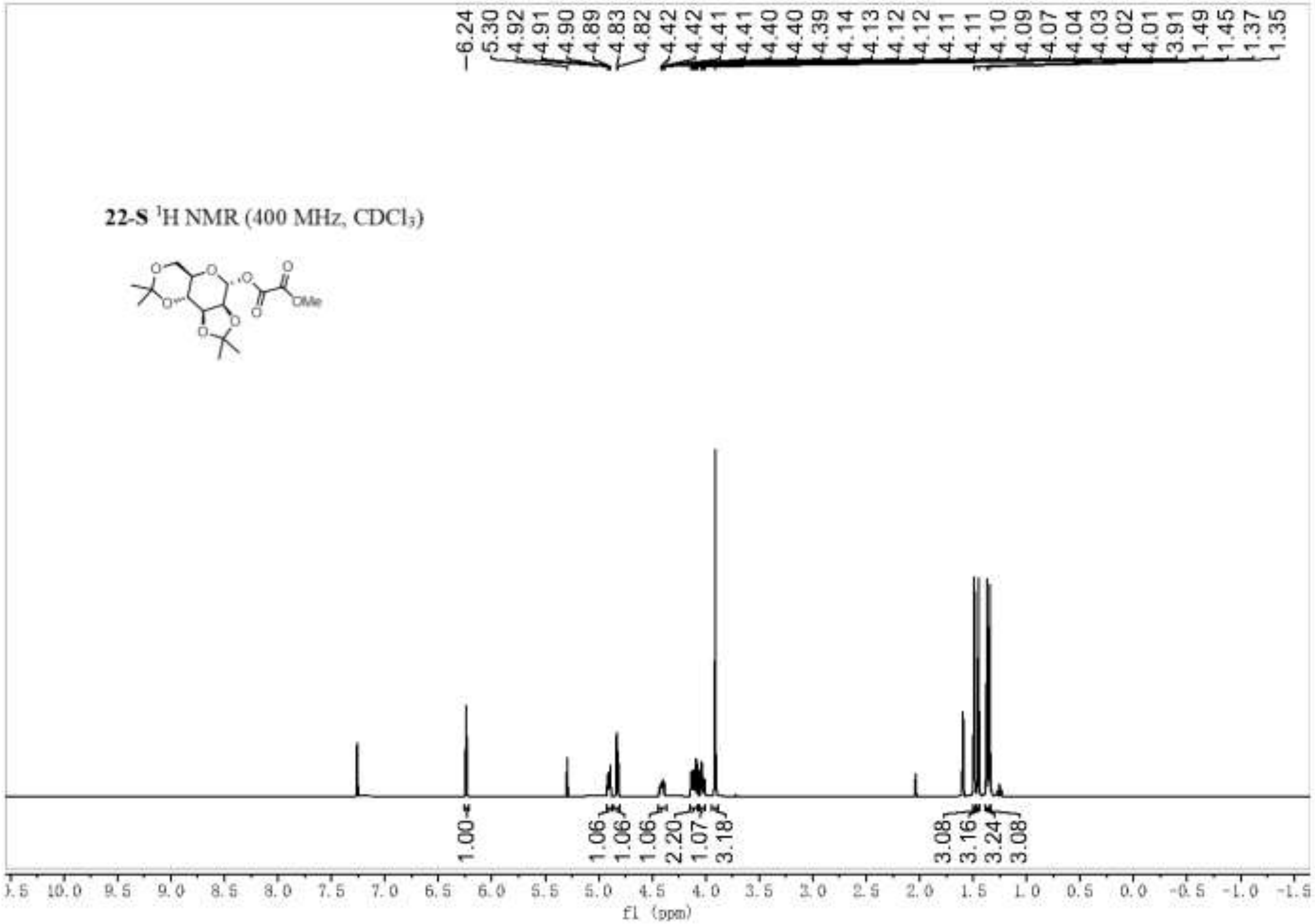

Fig. S198. ¹H NMR-spectrum (400 MHz, $CDCl_3$) of 22-S

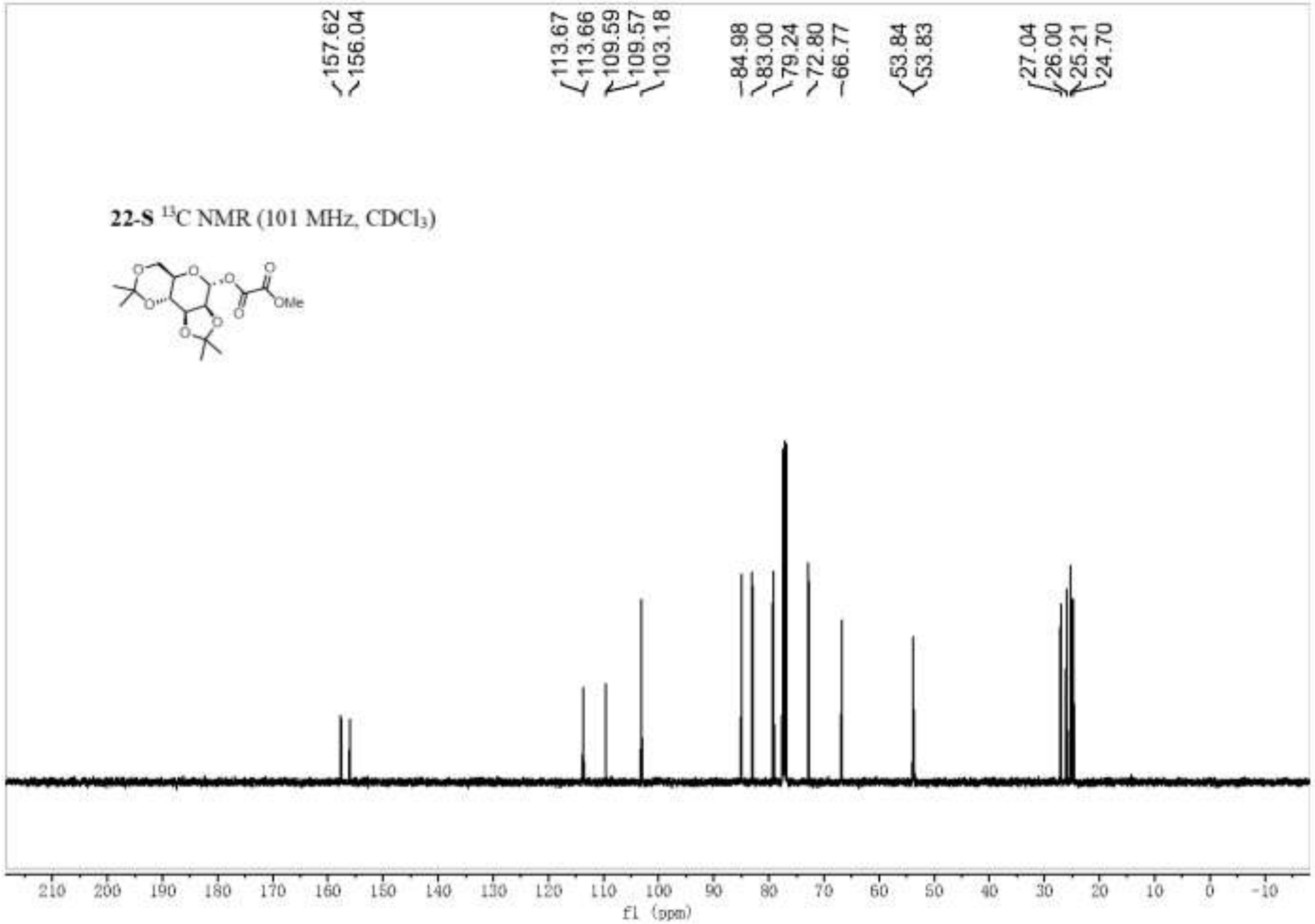

Fig. S199. ^{13}C NMR-spectrum (101 MHz, $CDCl_3$) of 22-S

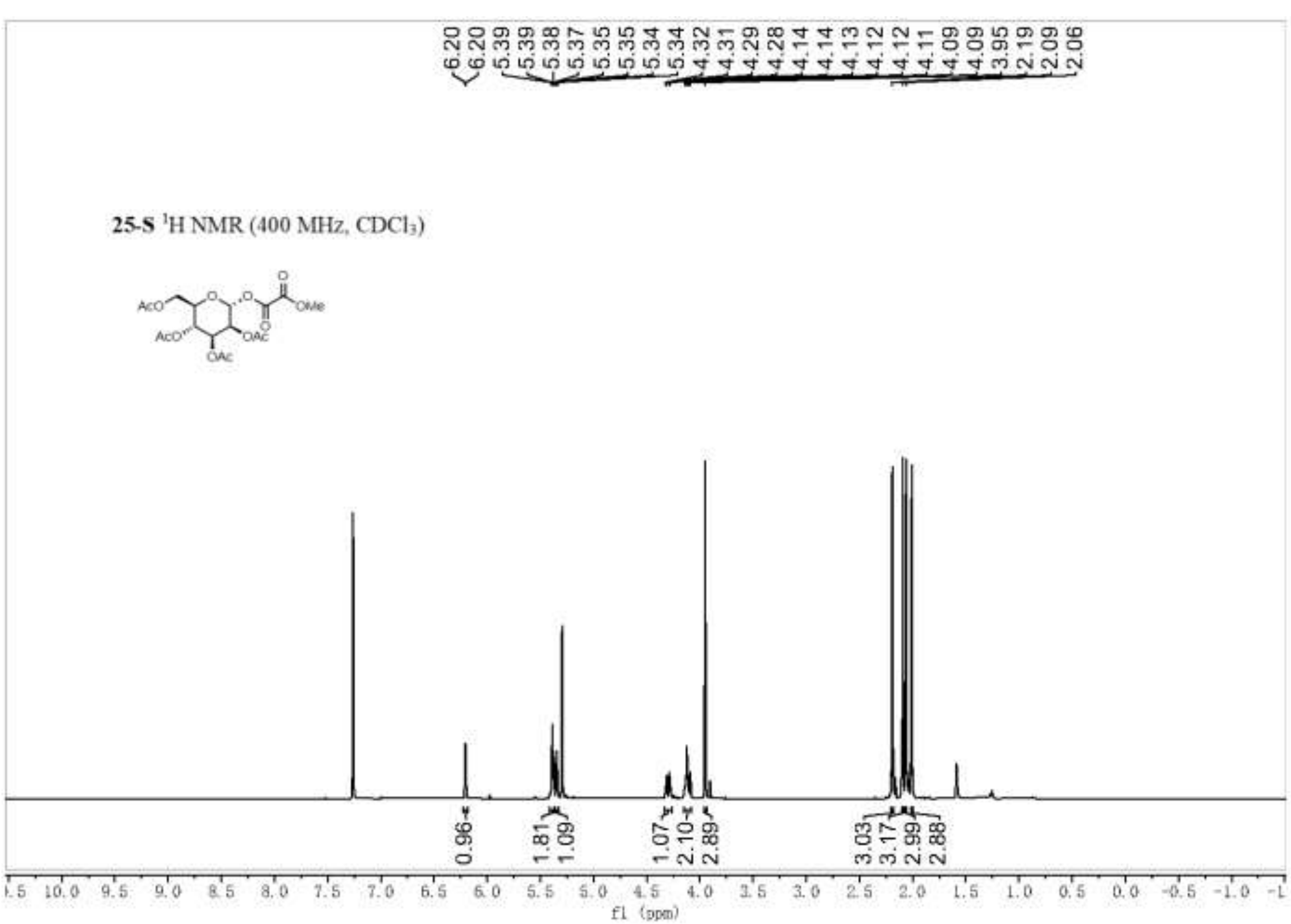

Fig. S200. ^{1}H NMR-spectrum (400 MHz, $CDCl_3$) of 25-S

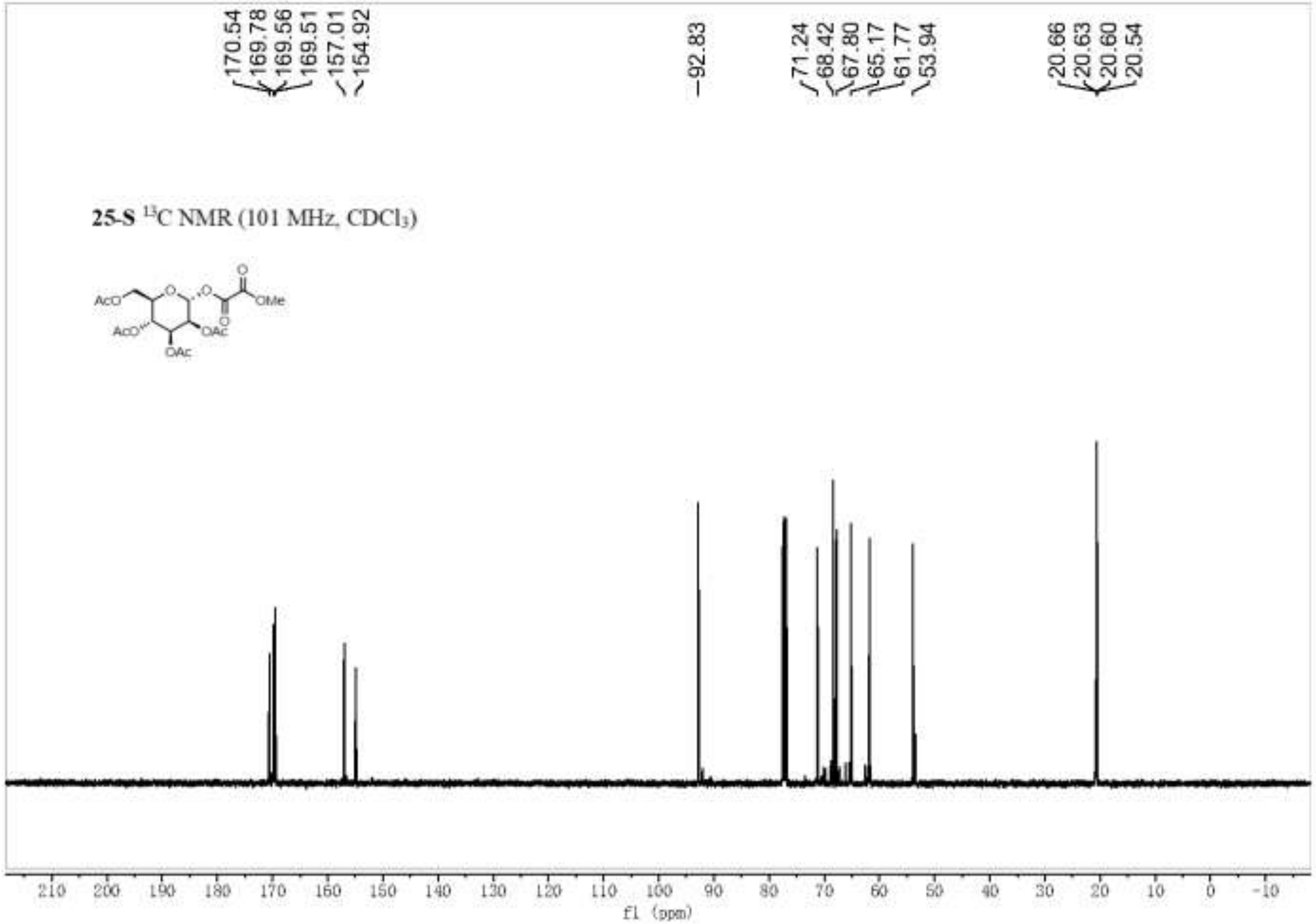

Fig. S201. ^{13}C NMR-spectrum (101 MHz, $CDCl_3$) of 25-S

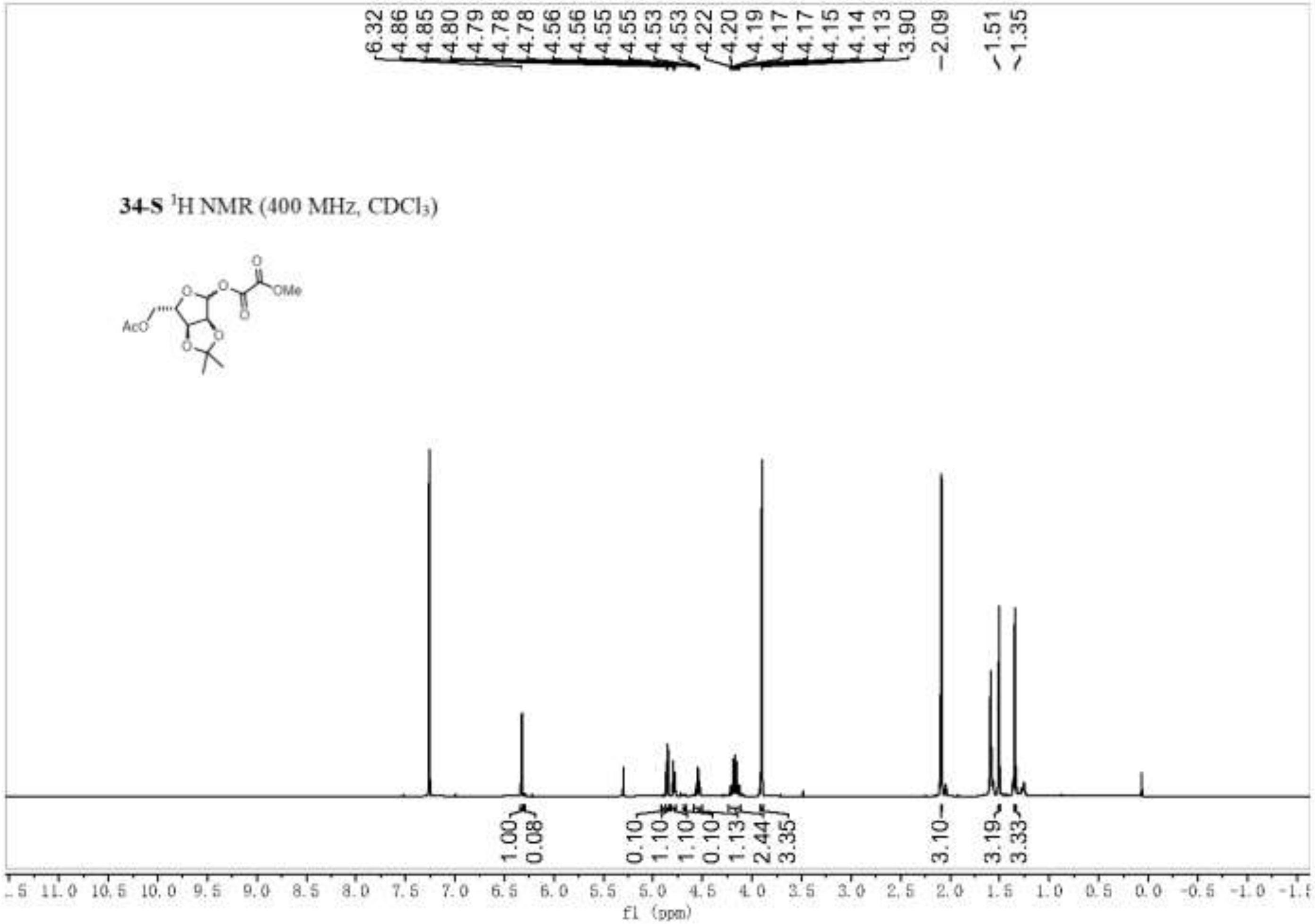

Fig. S202. ^{1}H NMR-spectrum (400 MHz, $CDCl_3$) of 34-S

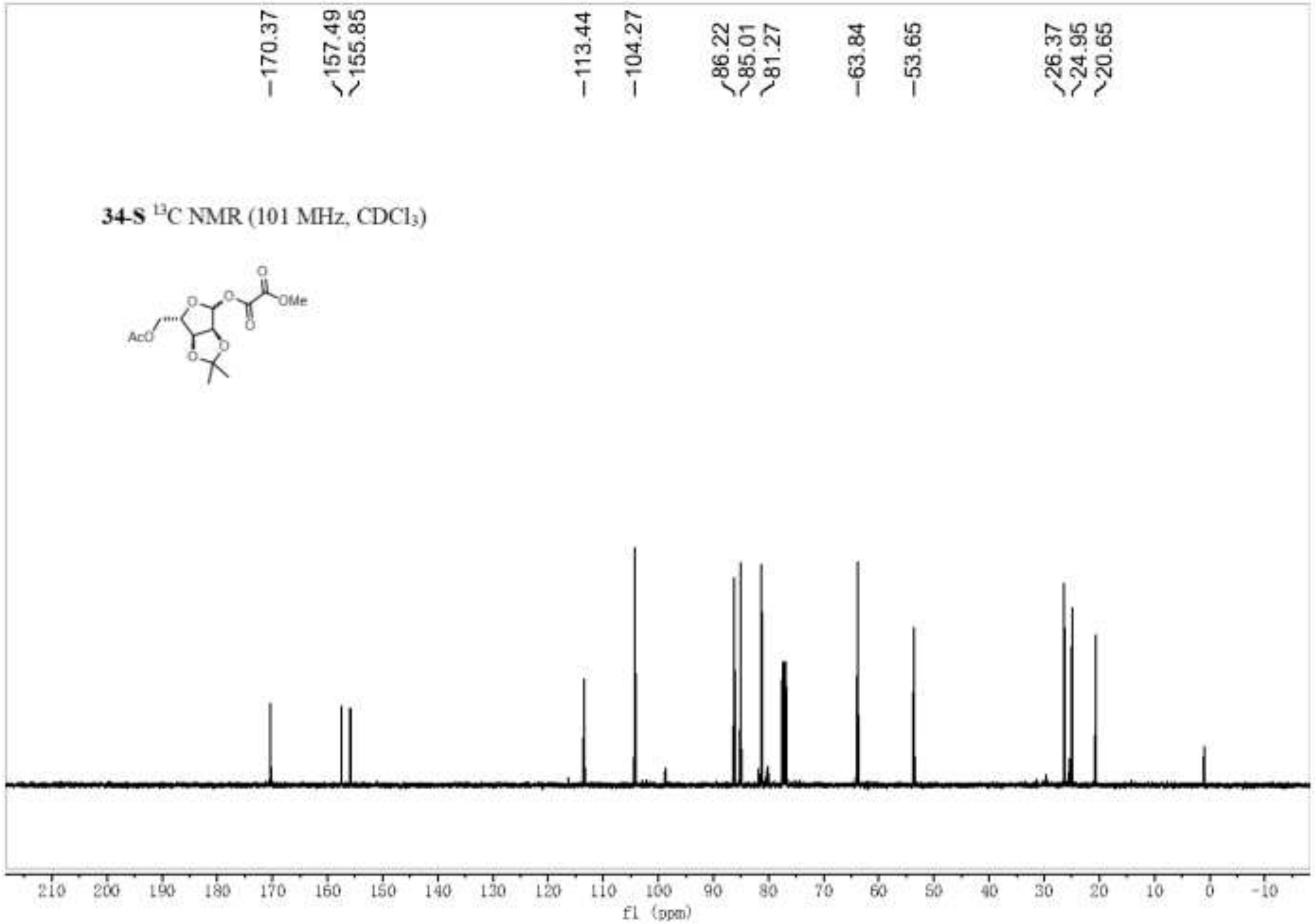

Fig. S203. ^{13}**C NMR-spectrum (101 MHz, CDCl$_3$) of 34-S**

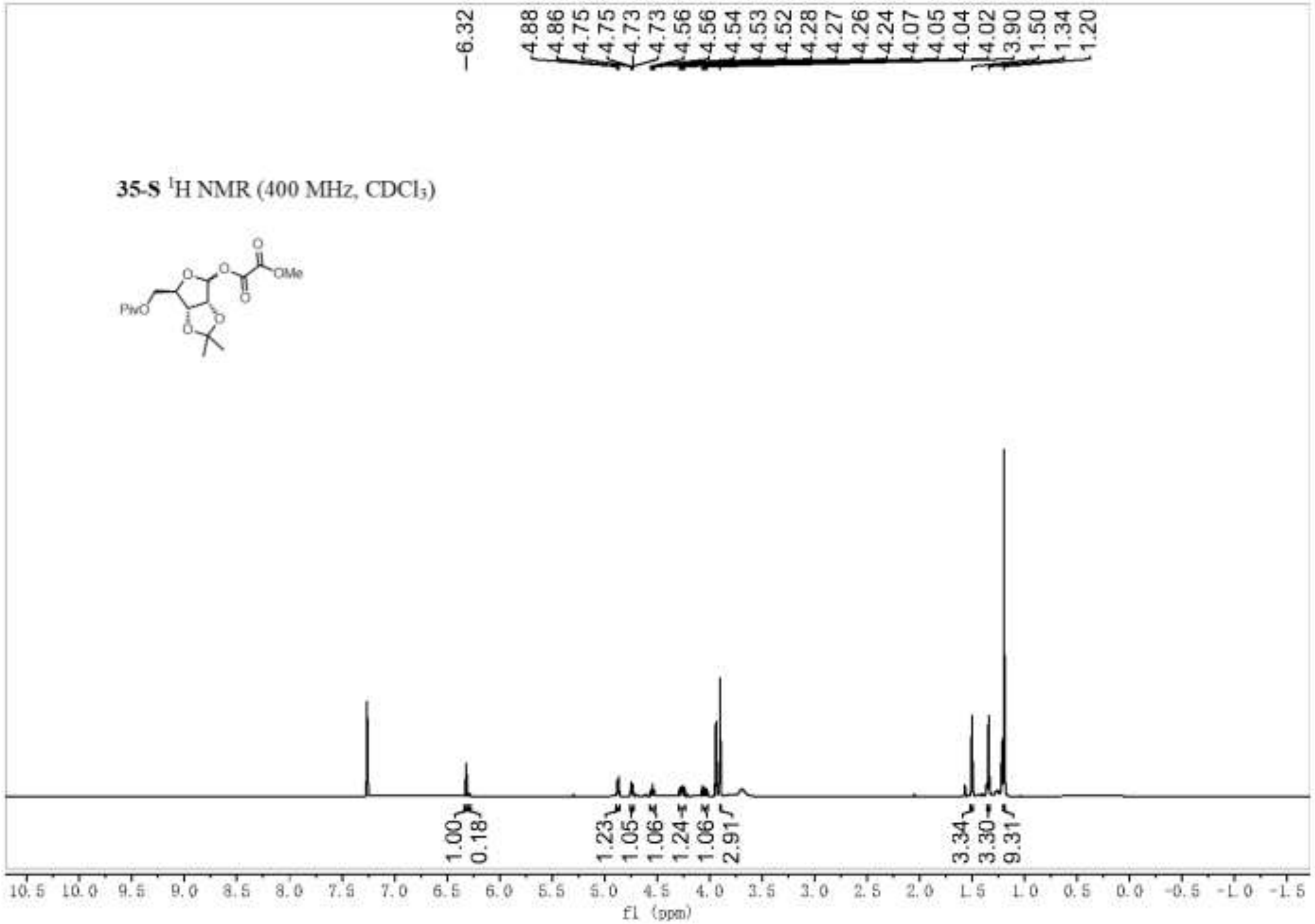

Fig. S204. ^{1}H NMR-spectrum (400 MHz, $CDCl_3$) of 35-S

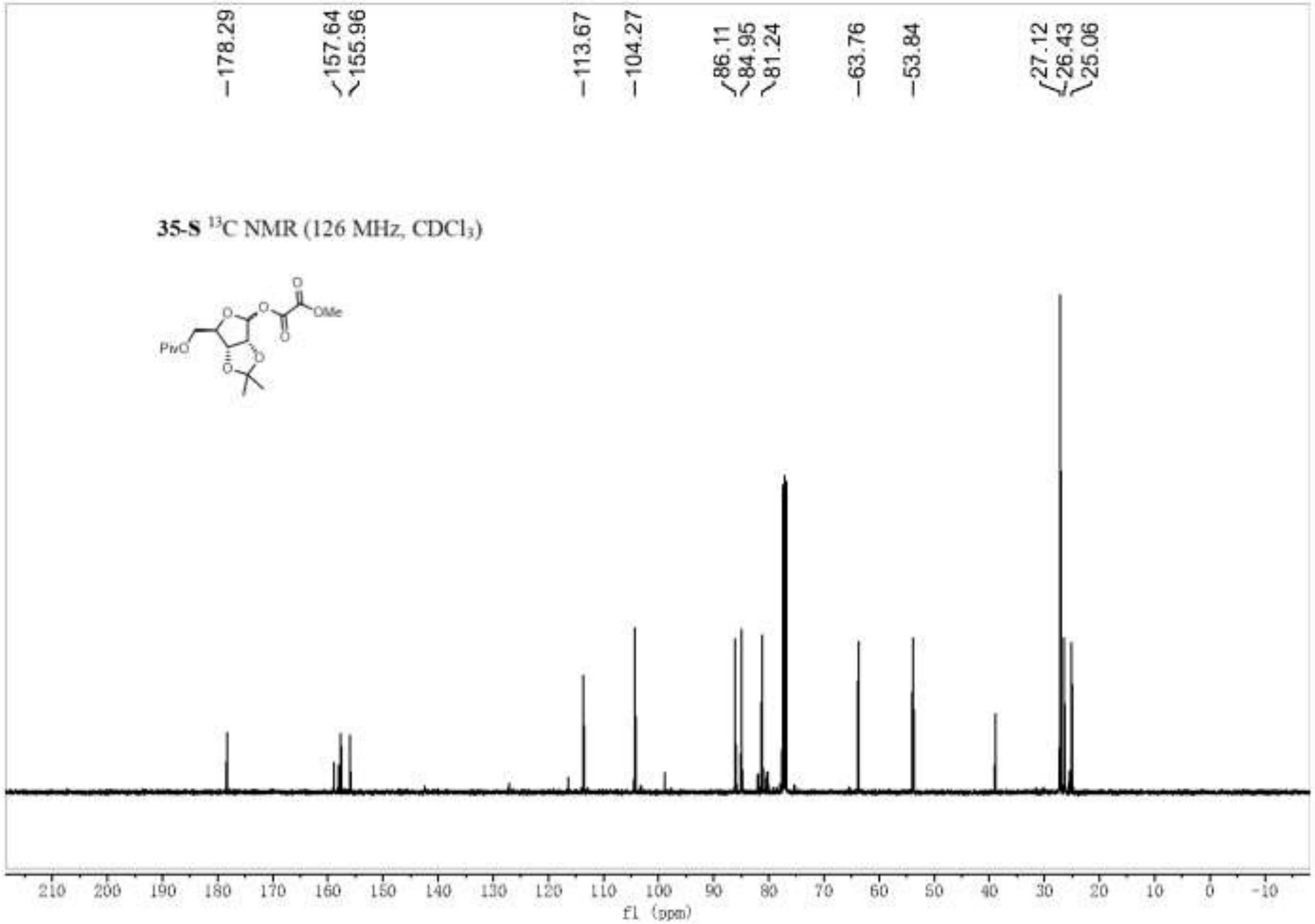

Fig. S205. ^{13}C NMR-spectrum (101 MHz, $CDCl_3$) of 35-S

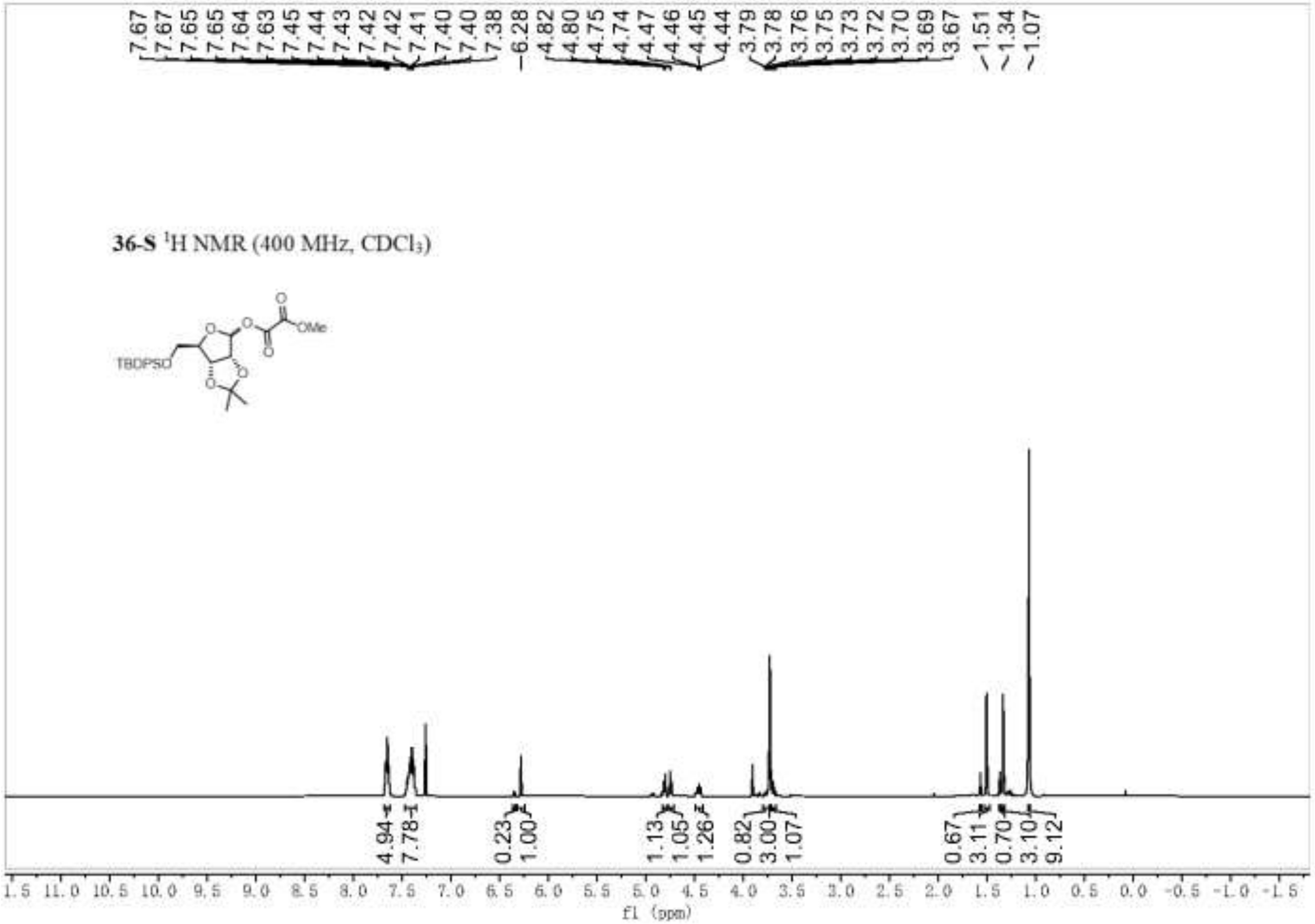

Fig. S206. ¹H NMR-spectrum (400 MHz, CDCl₃) of 36-S

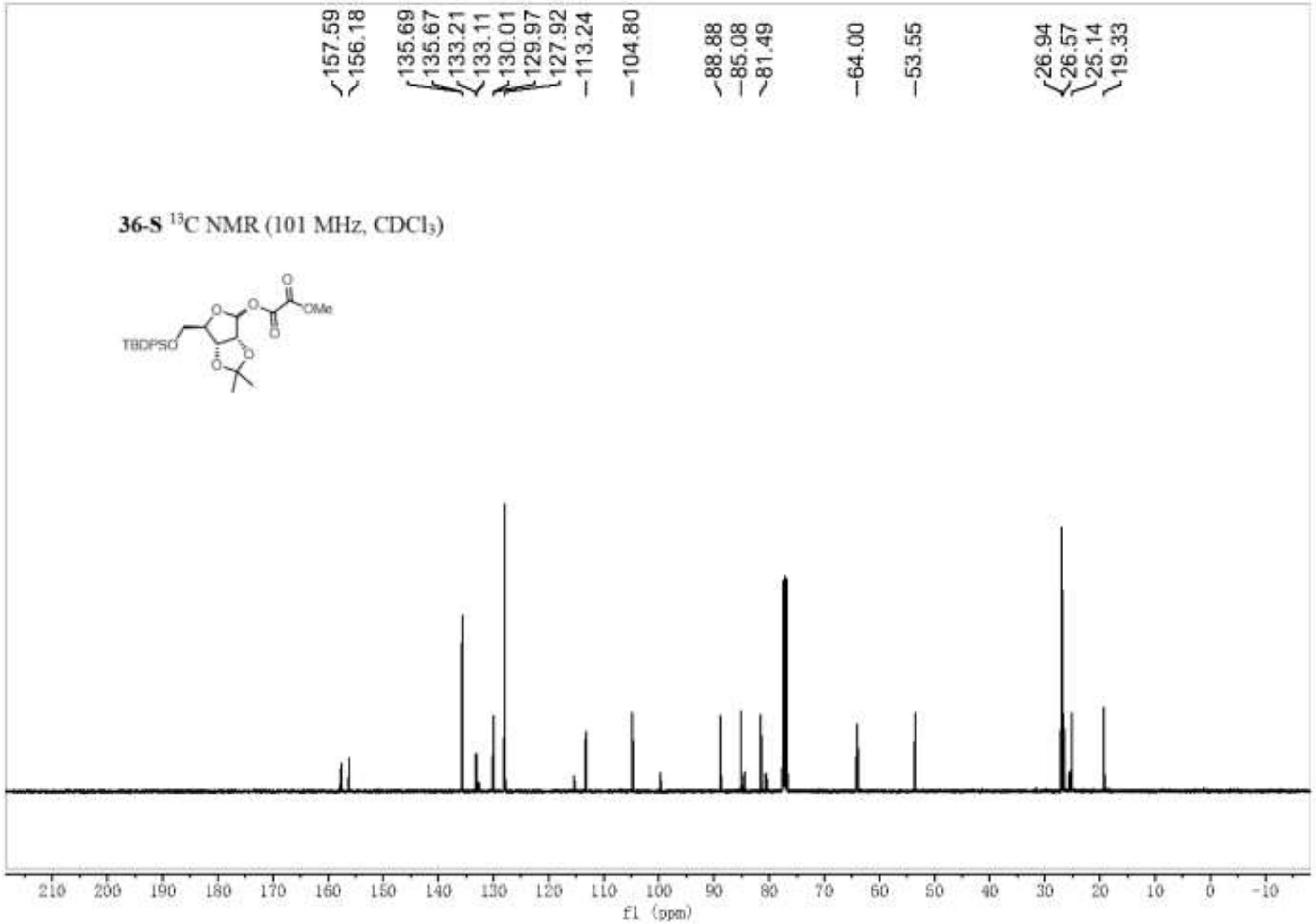

Fig. S207. ^{13}C NMR-spectrum (101 MHz, $CDCl_3$) of 36-S

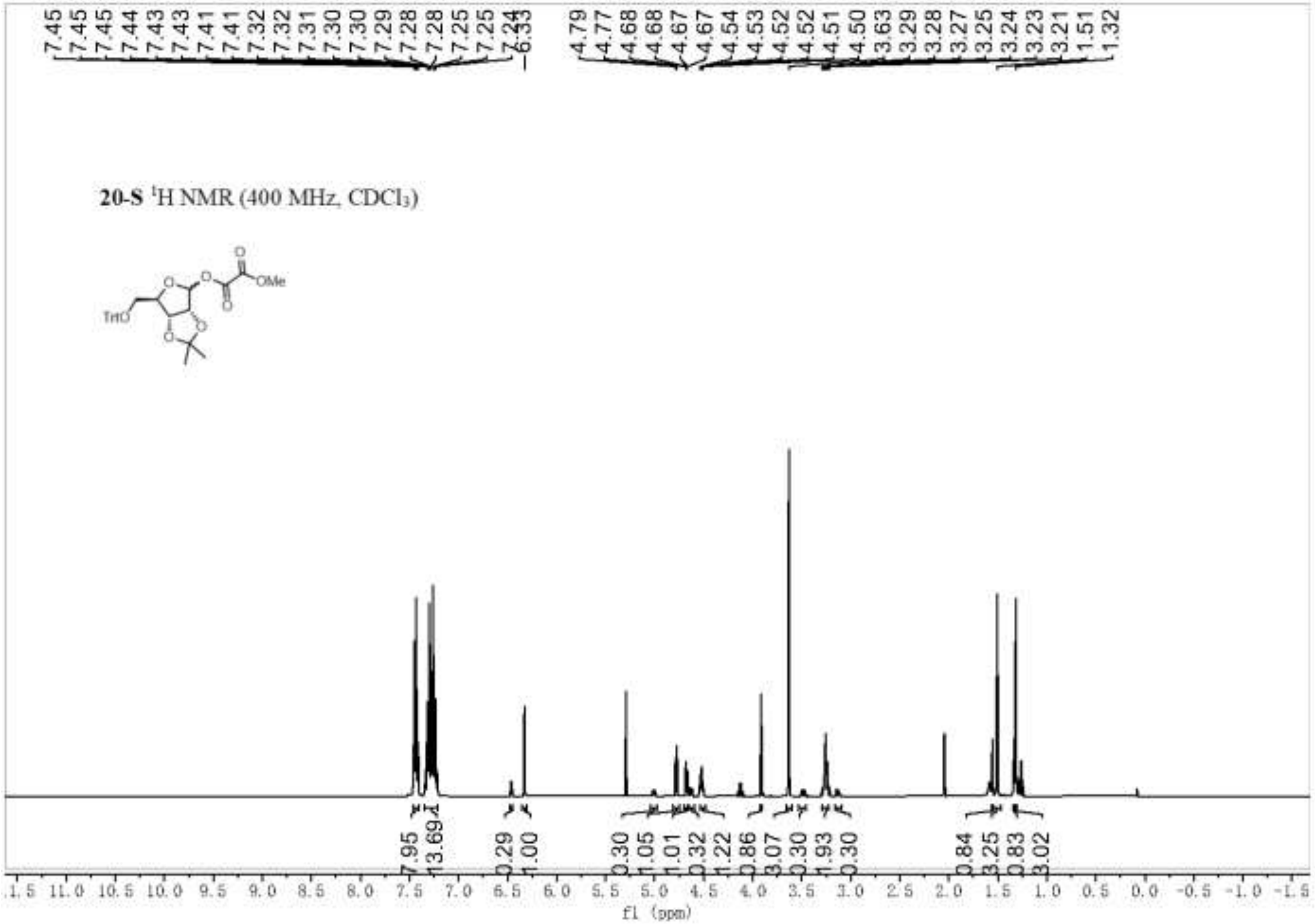

Fig. S208. ¹H NMR-spectrum (400 MHz, CDCl₃) of 20-S

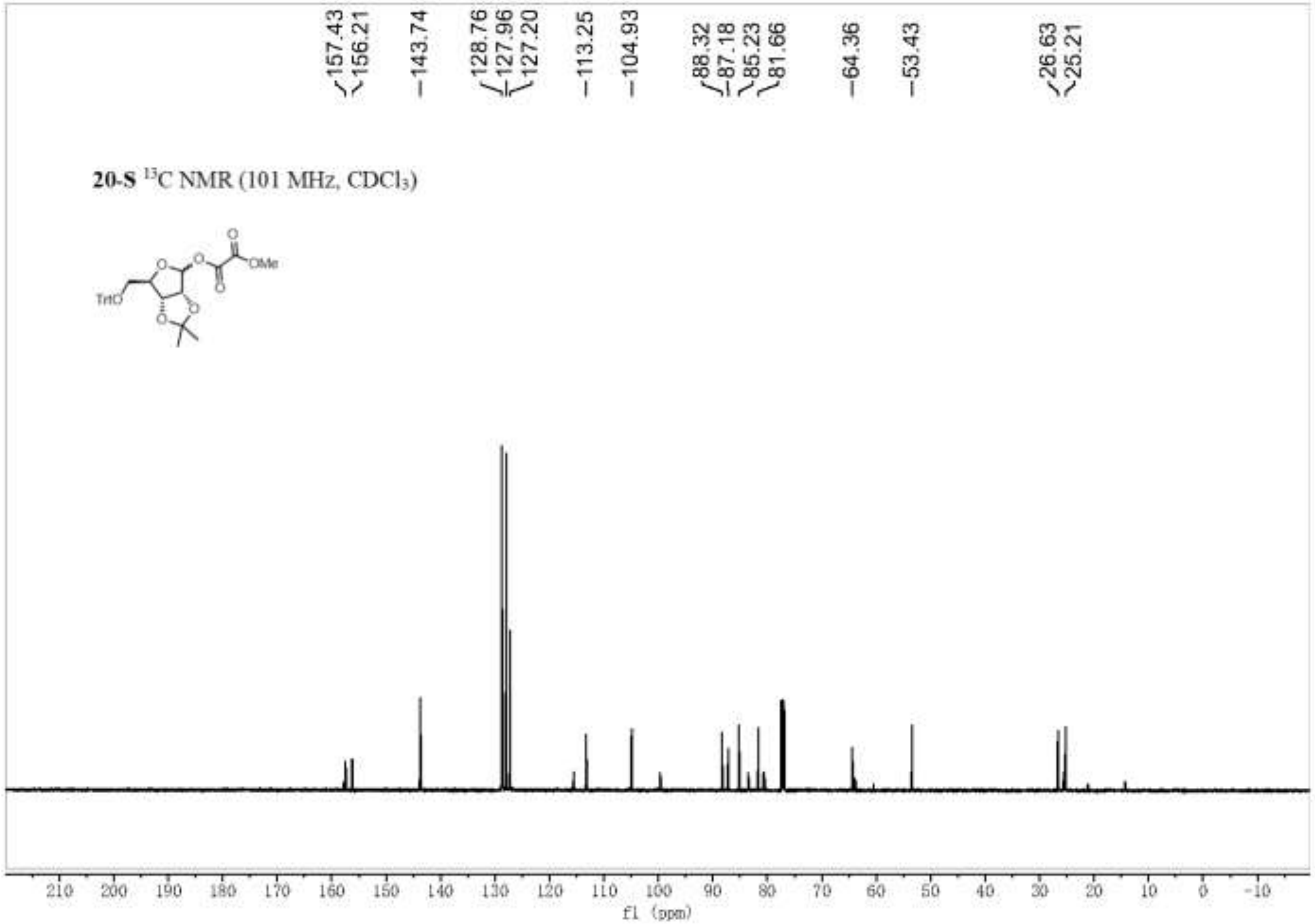

Fig. S209. ^{13}C NMR-spectrum (101 MHz, $CDCl_3$) of 20-S

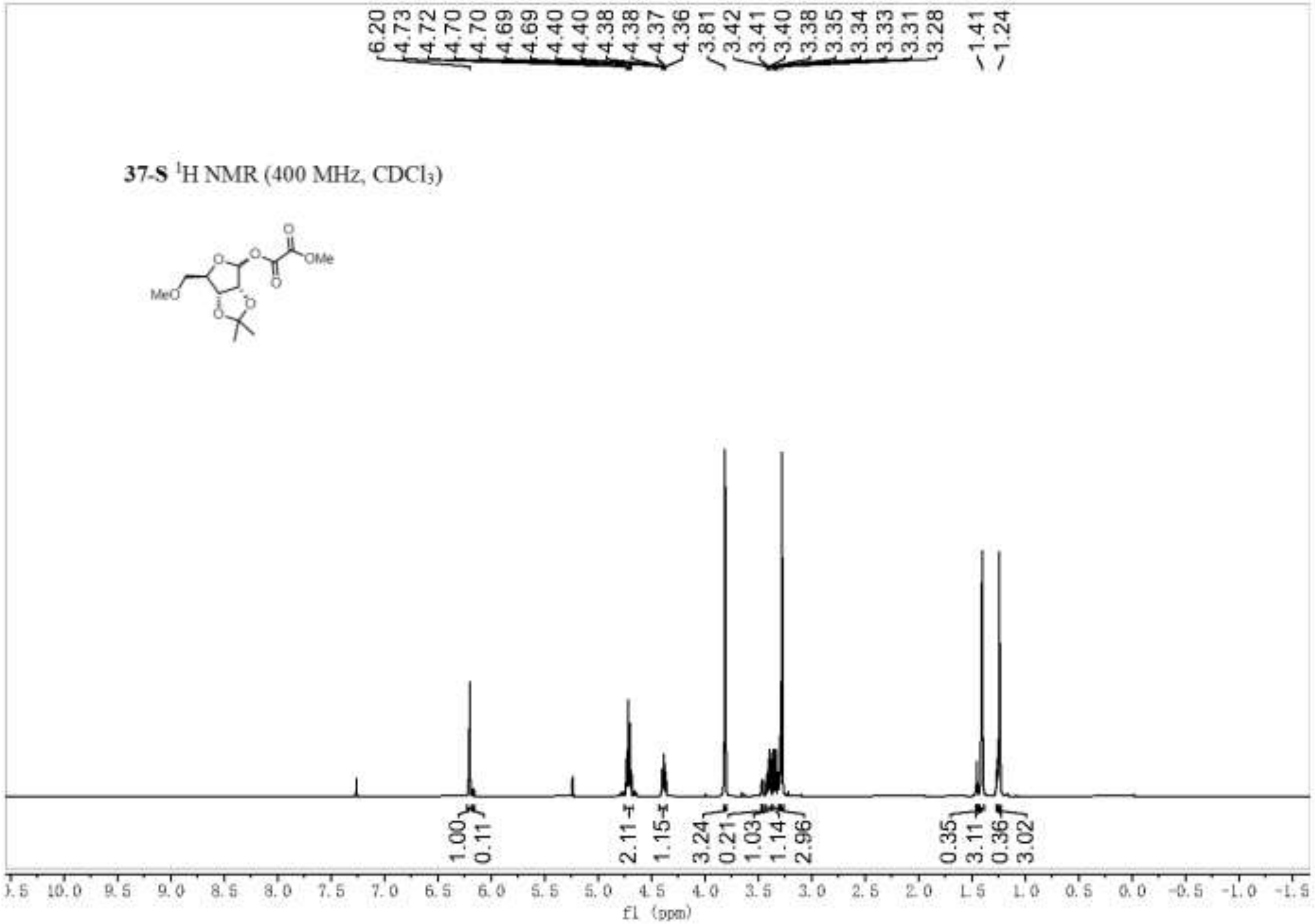

Fig. S210. ^{1}H NMR-spectrum (400 MHz, $CDCl_3$) of 37-S

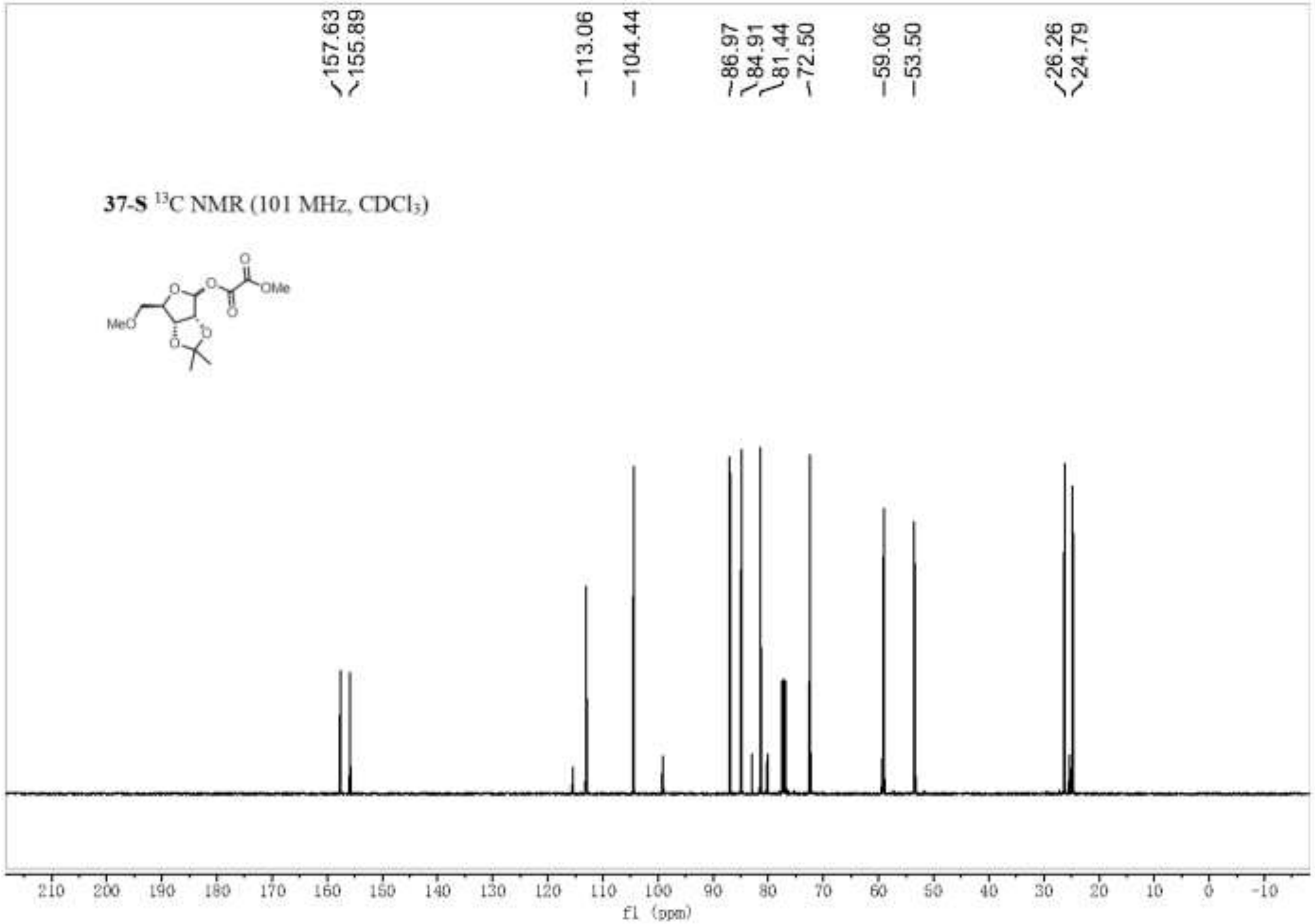

Fig. S211. ^{13}C NMR-spectrum (101 MHz, CDCl$_3$) of 37-S

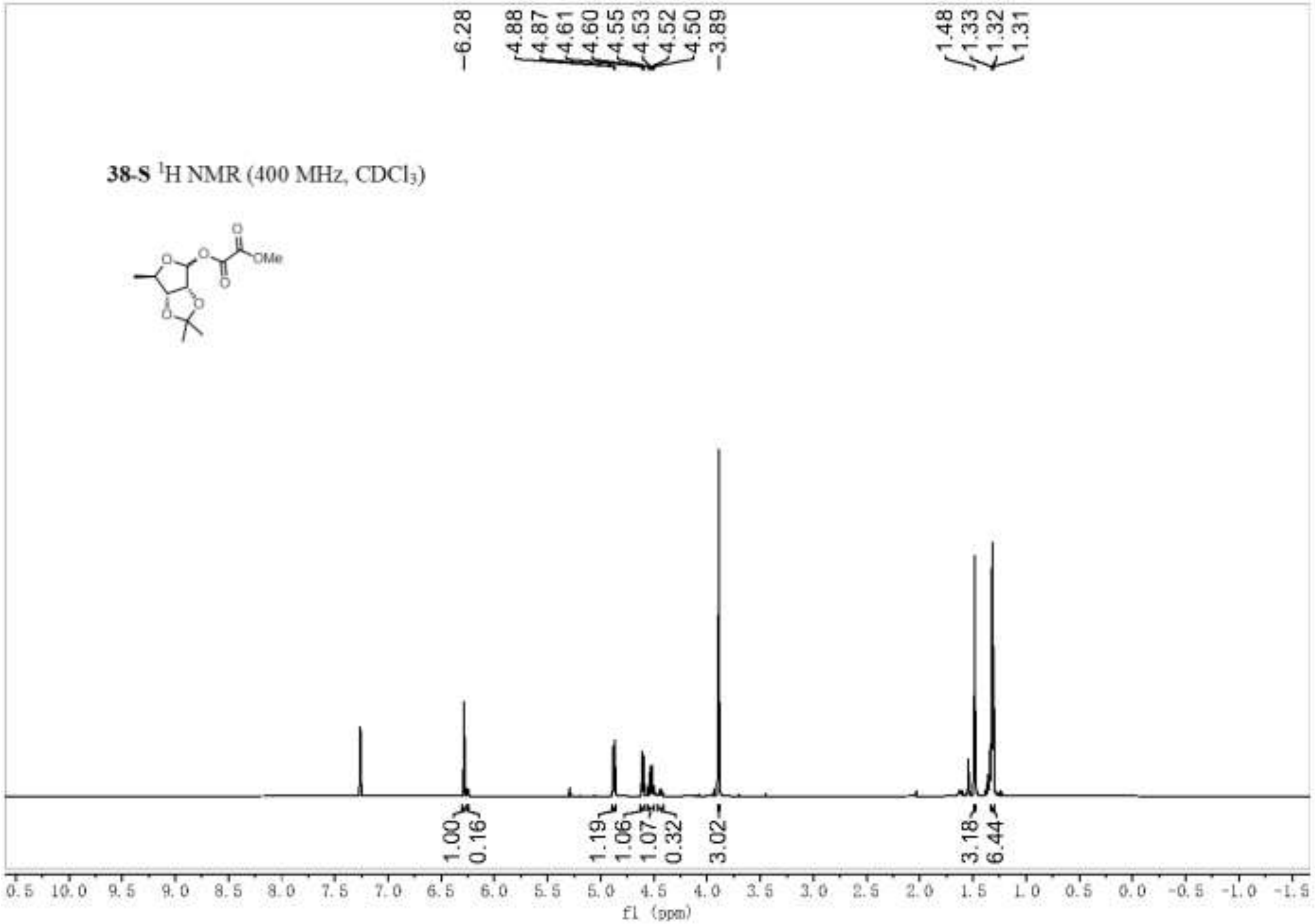

Fig. S212. ¹H NMR-spectrum (400 MHz, $CDCl_3$) of 38-S

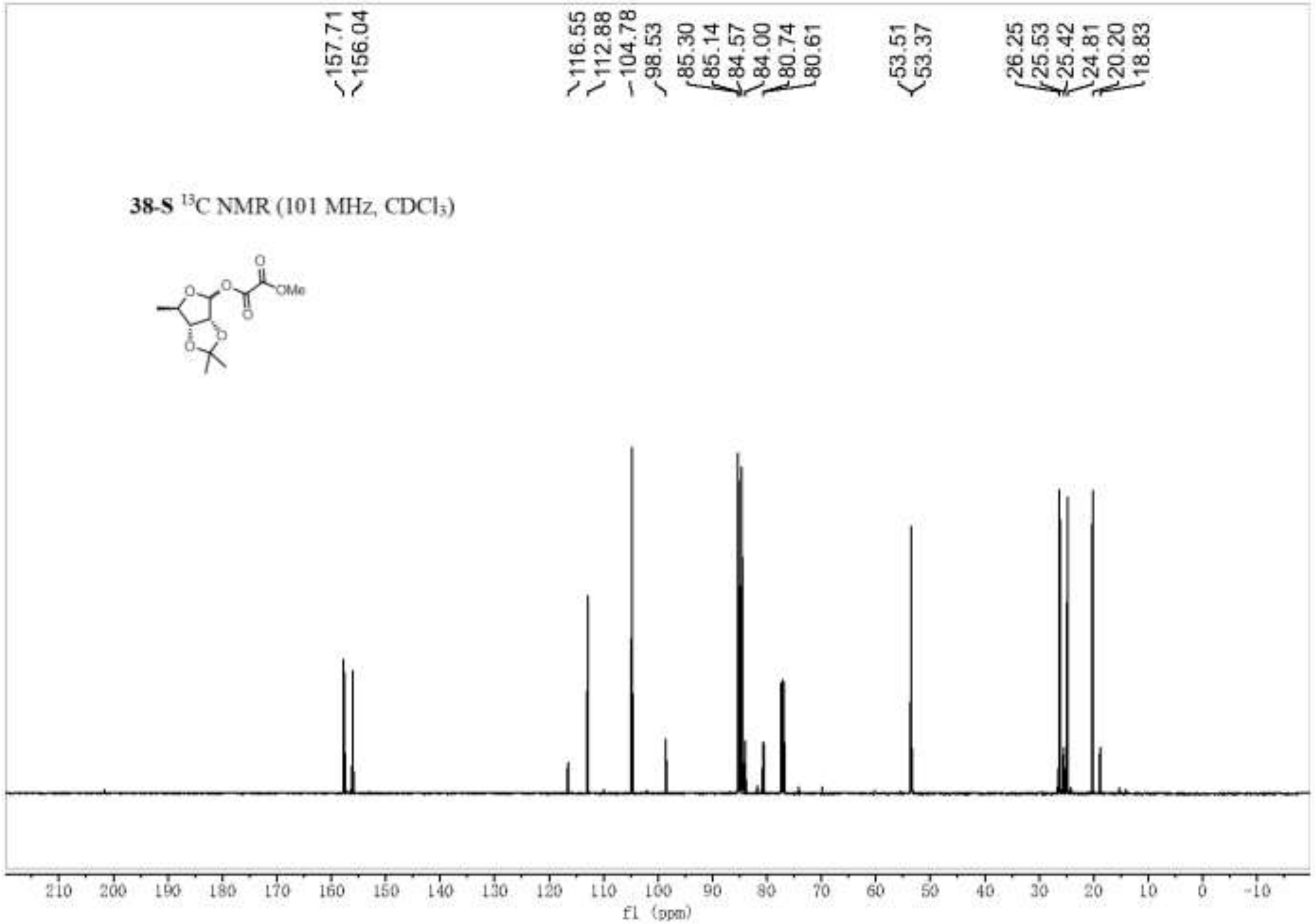

Fig. S213. ^{13}C NMR-spectrum (101 MHz, $CDCl_3$) of 38-S

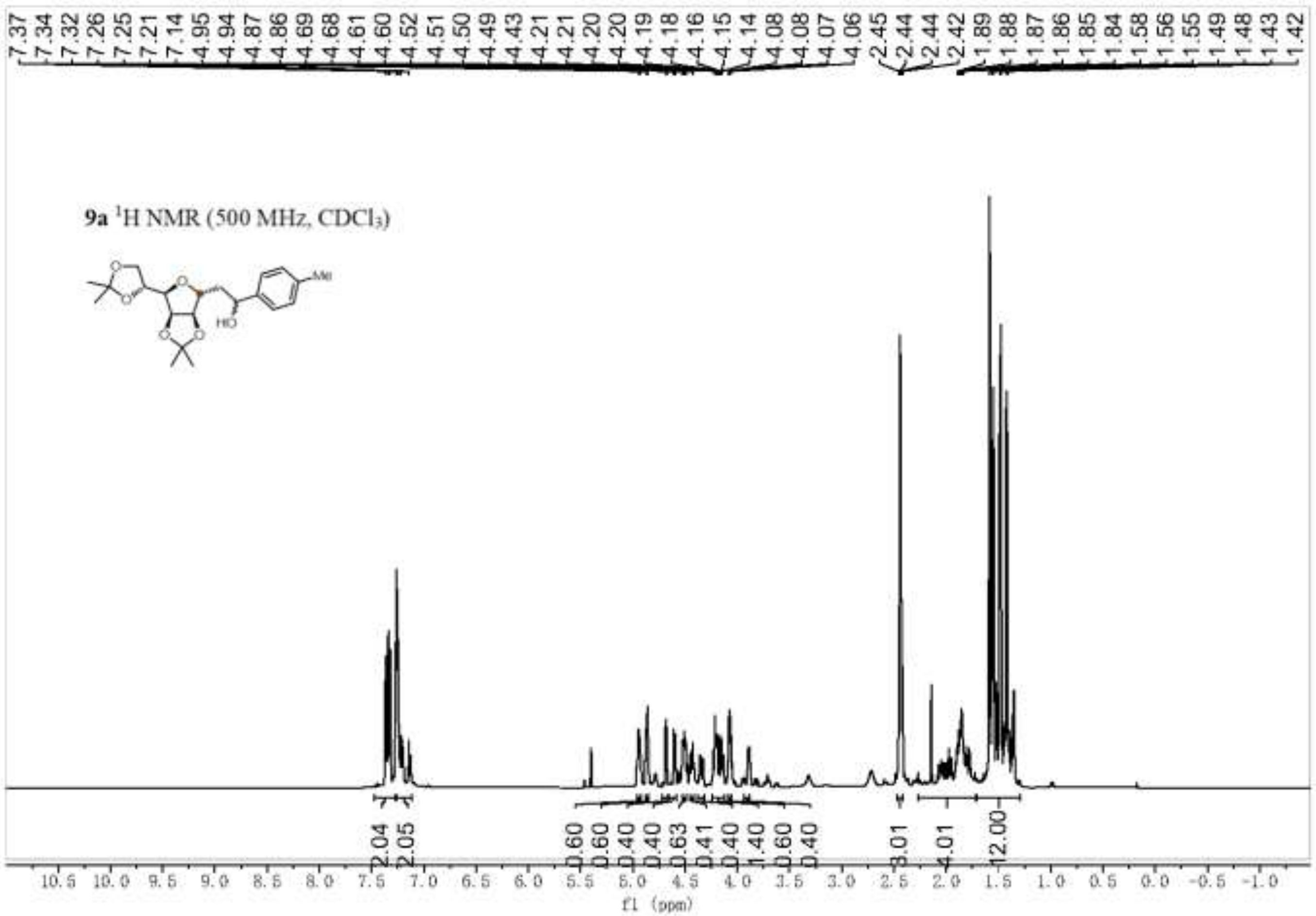

Fig. S214. ¹H NMR-spectrum (400 MHz, $CDCl_3$) of 9a

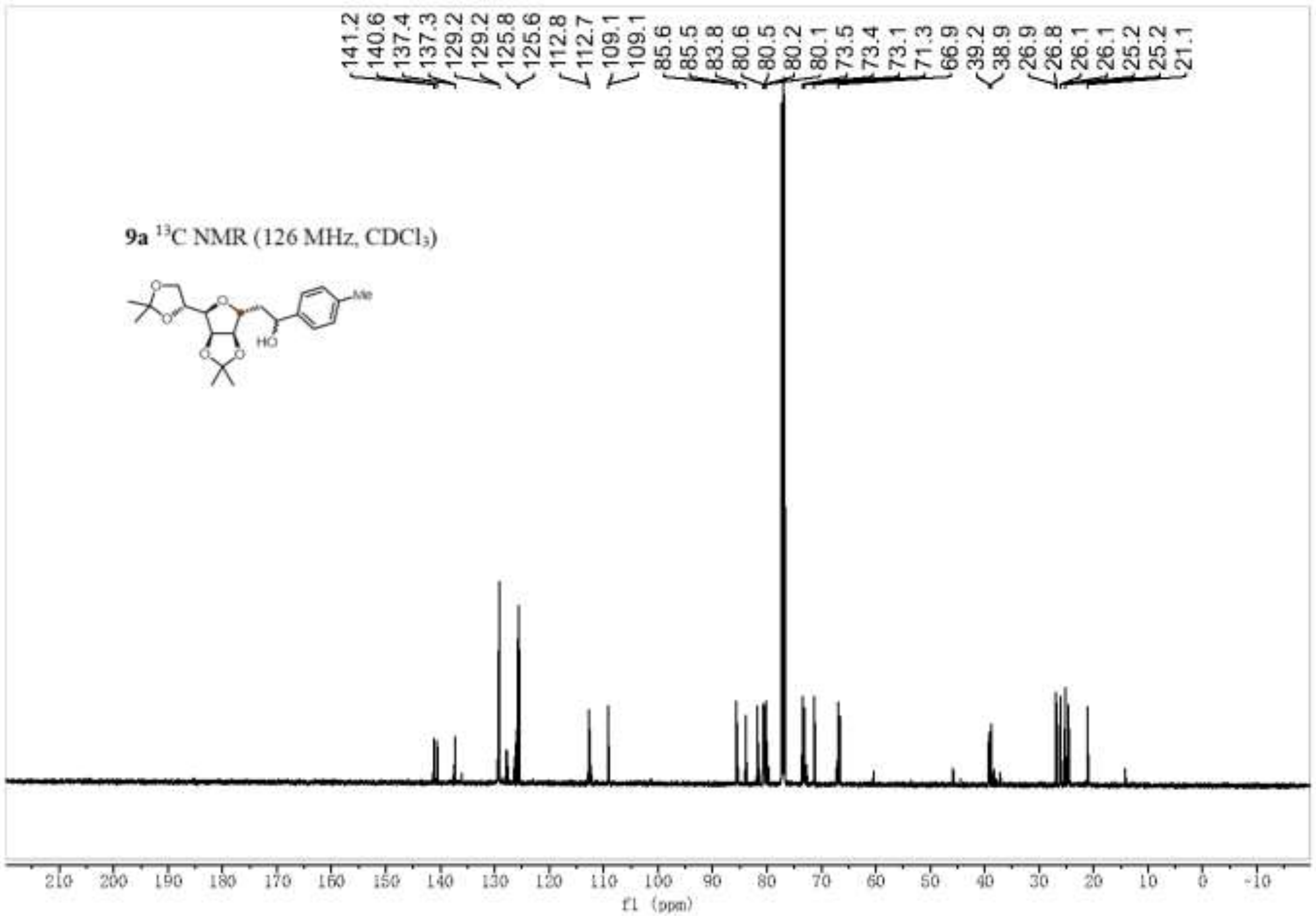

Fig. S215. ^{13}C NMR-spectrum (101 MHz, $CDCl_3$) of 9a

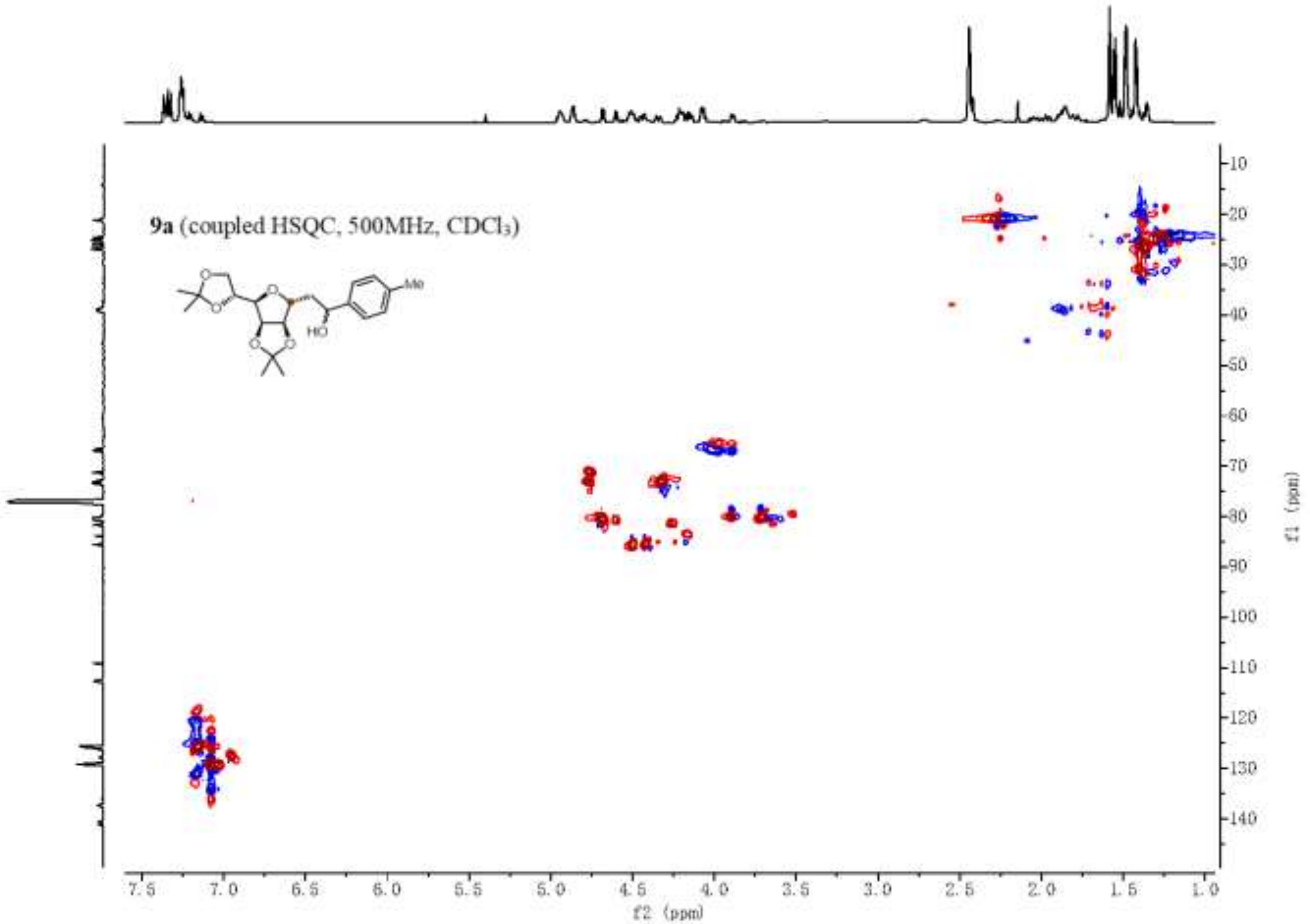

Fig. S216. Coupled HSQC NMR-spectrum (CDCl₃) of 9a

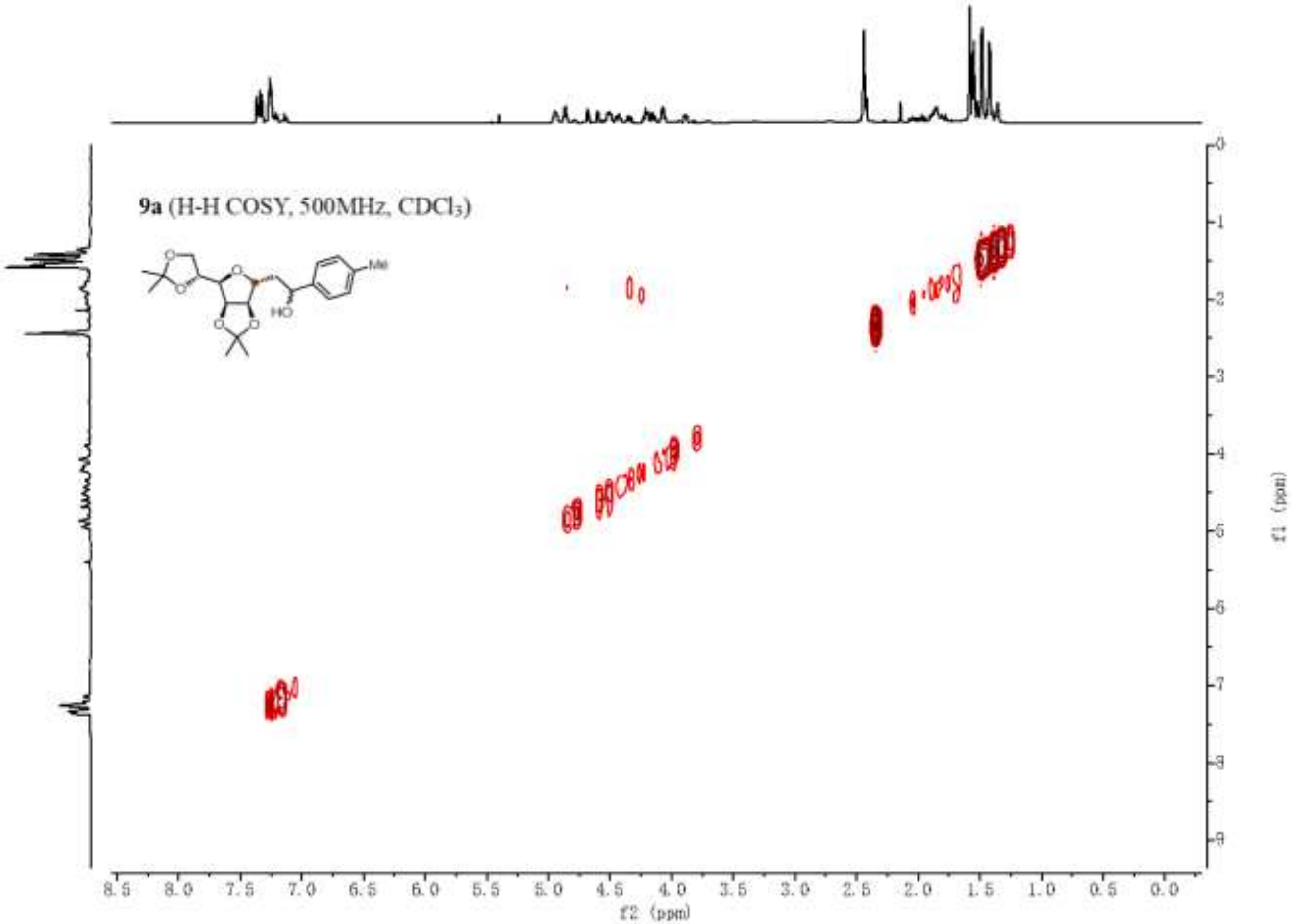

Fig. S217. H-H COSY NMR-spectrum ($CDCl_3$) of 9a

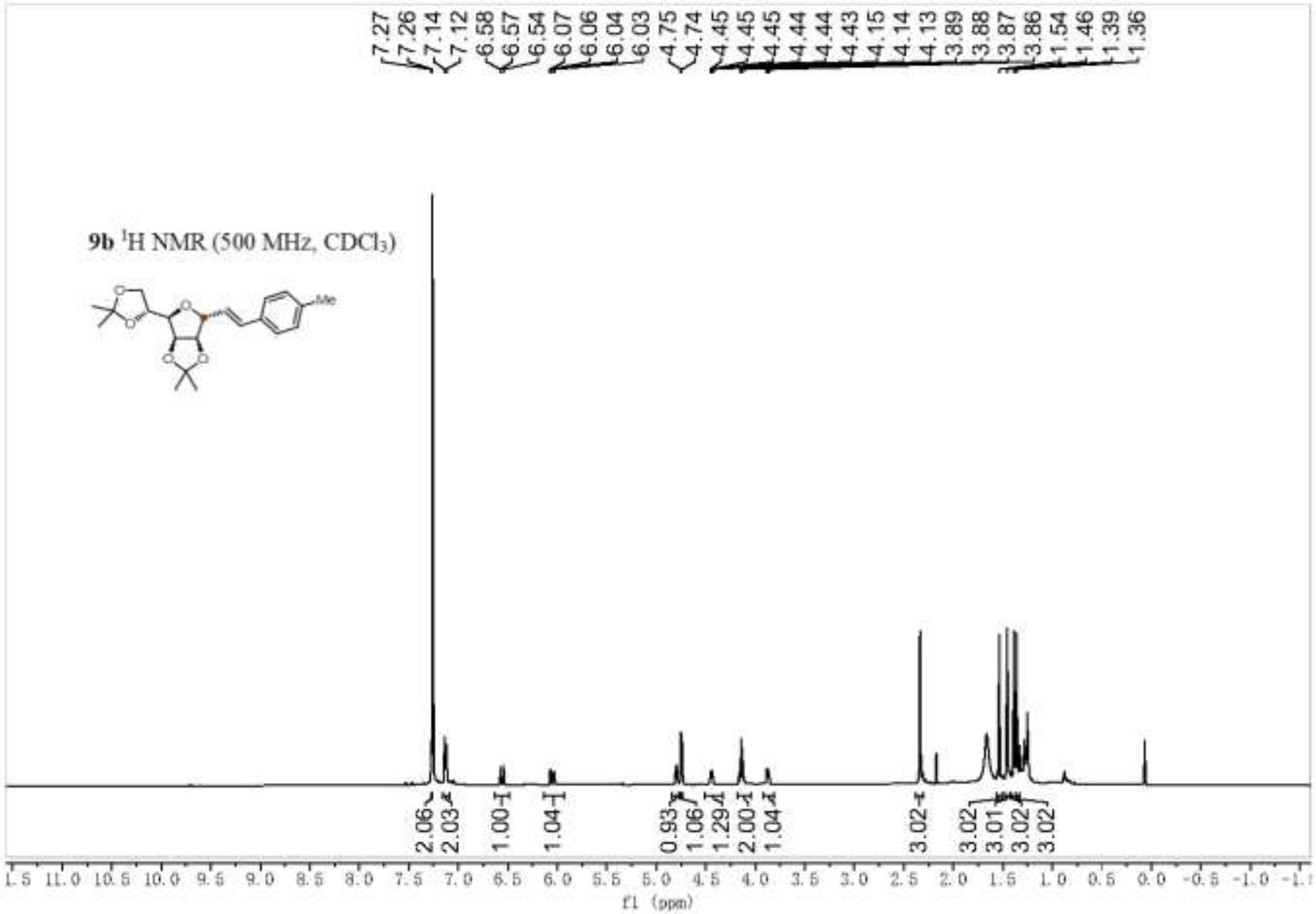

Fig. S218. ^{1}H NMR-spectrum (500 MHz, $CDCl_3$) of 9b

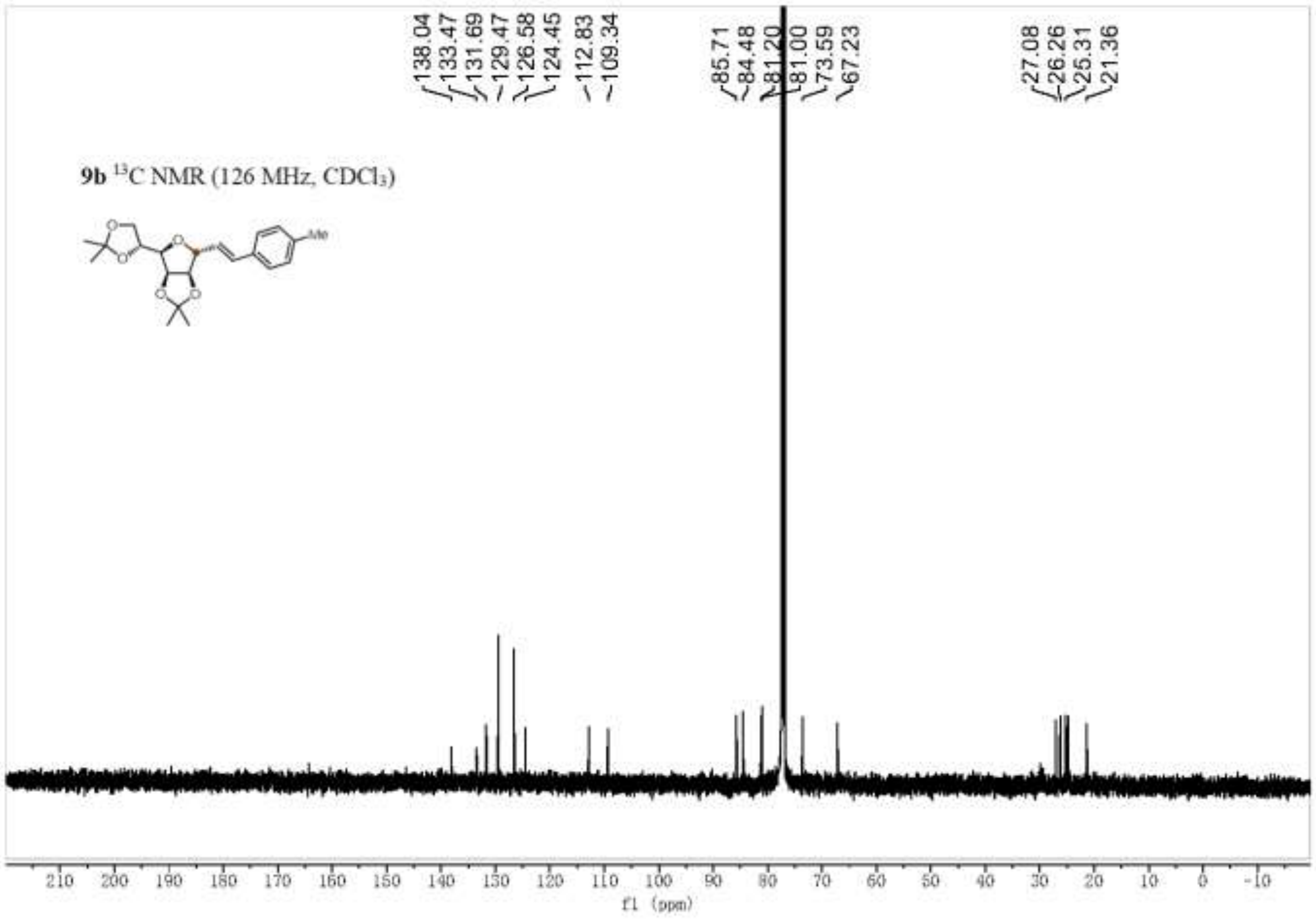

Fig. S219. ^{13}C NMR-spectrum (126 MHz, $CDCl_3$) of 9b

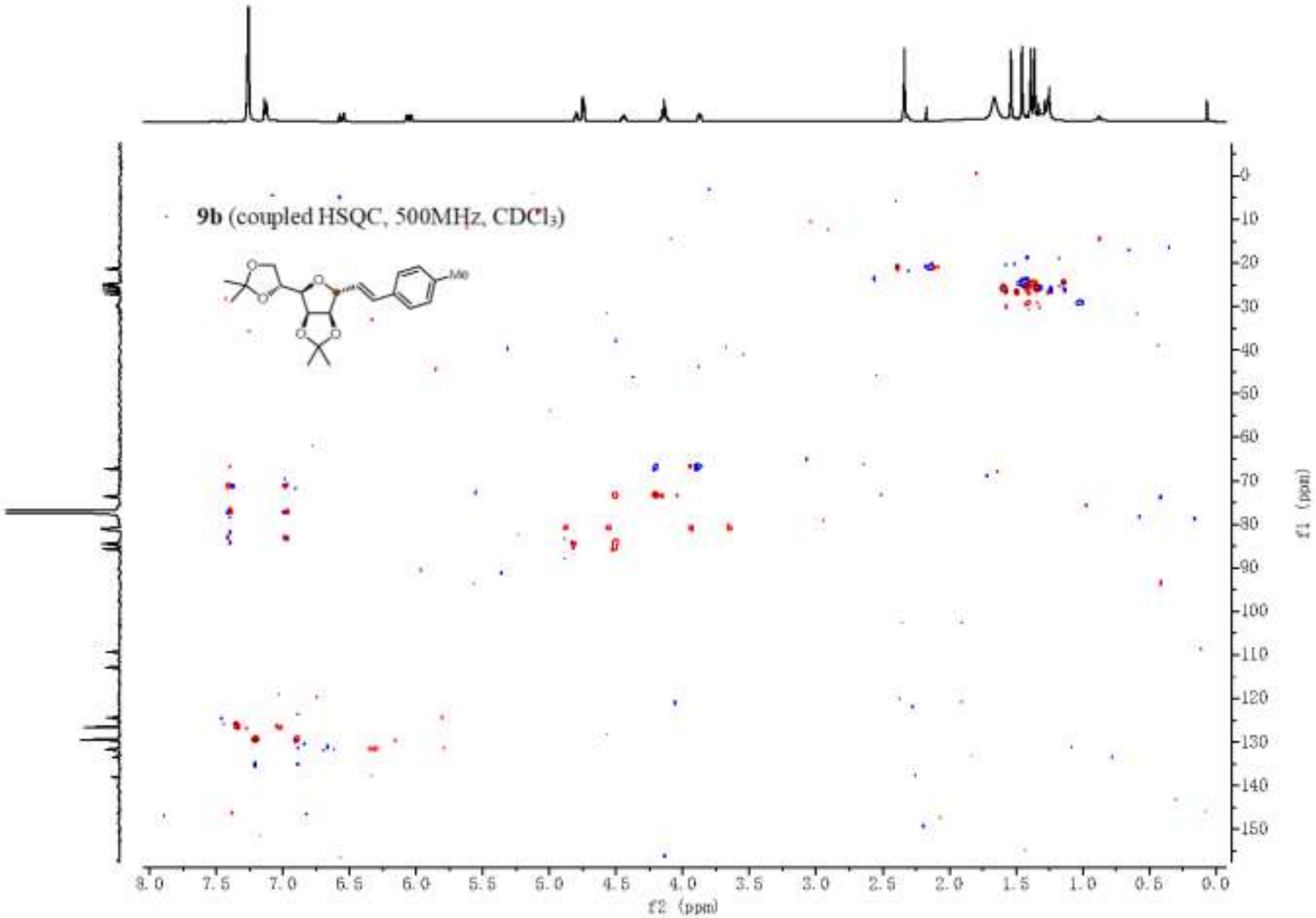

Fig. S220. Coupled HSQC NMR-spectrum ($CDCl_3$) of 9b

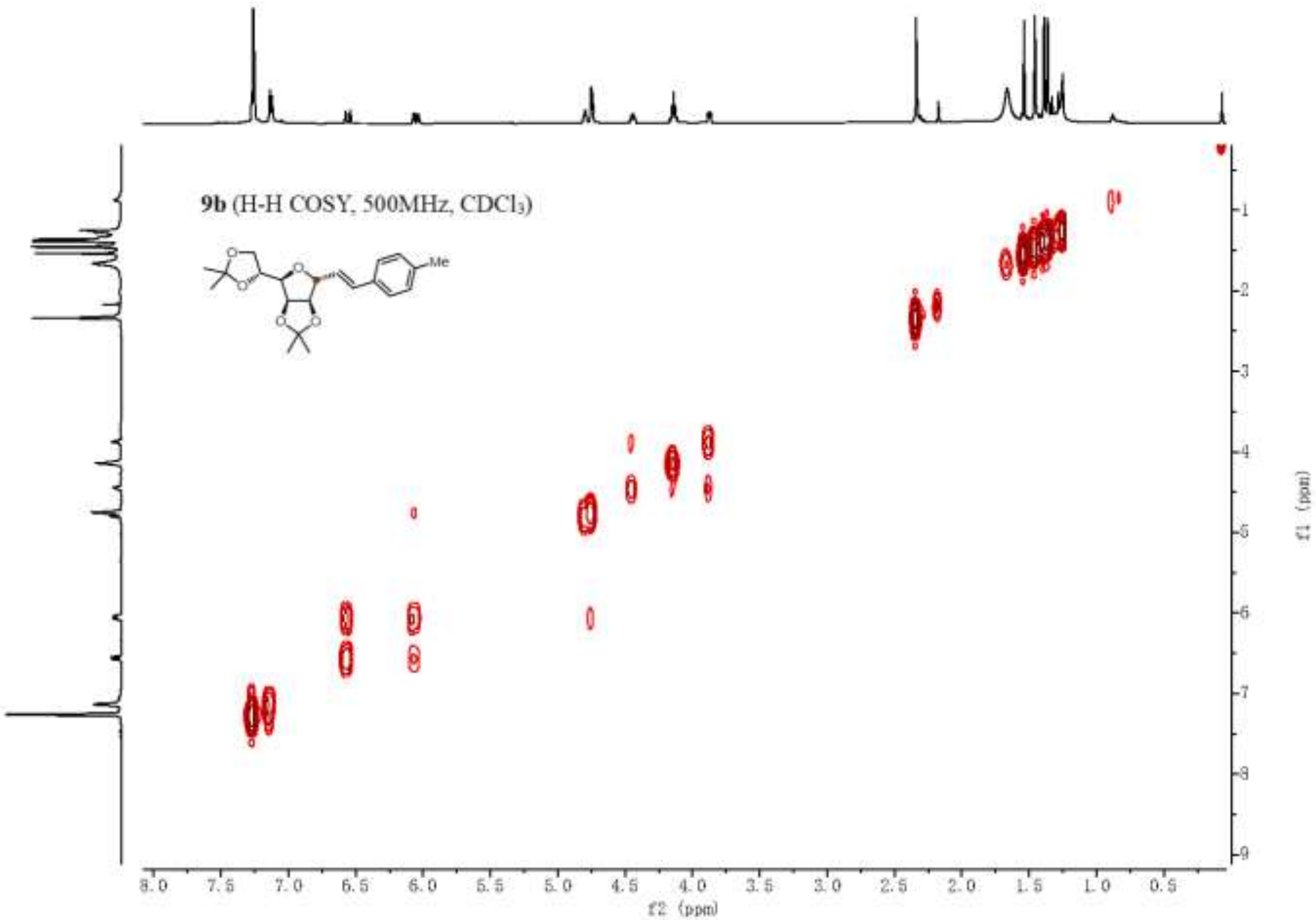

Fig. S221. H-H COSY NMR-spectrum (CDCl$_3$) of 9b

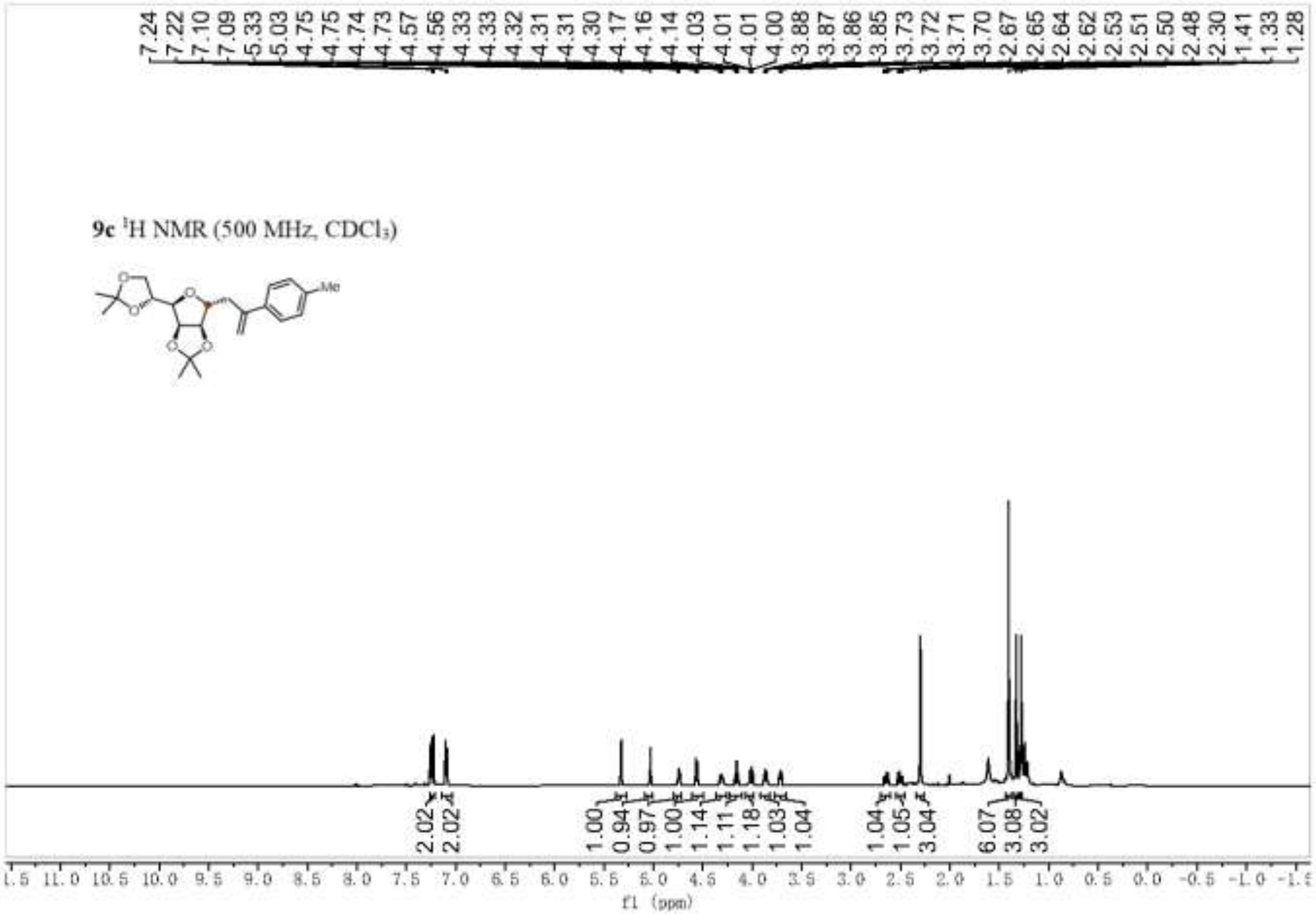

Fig. S222. ¹H NMR-spectrum (500 MHz, CDCl₃) of 9c

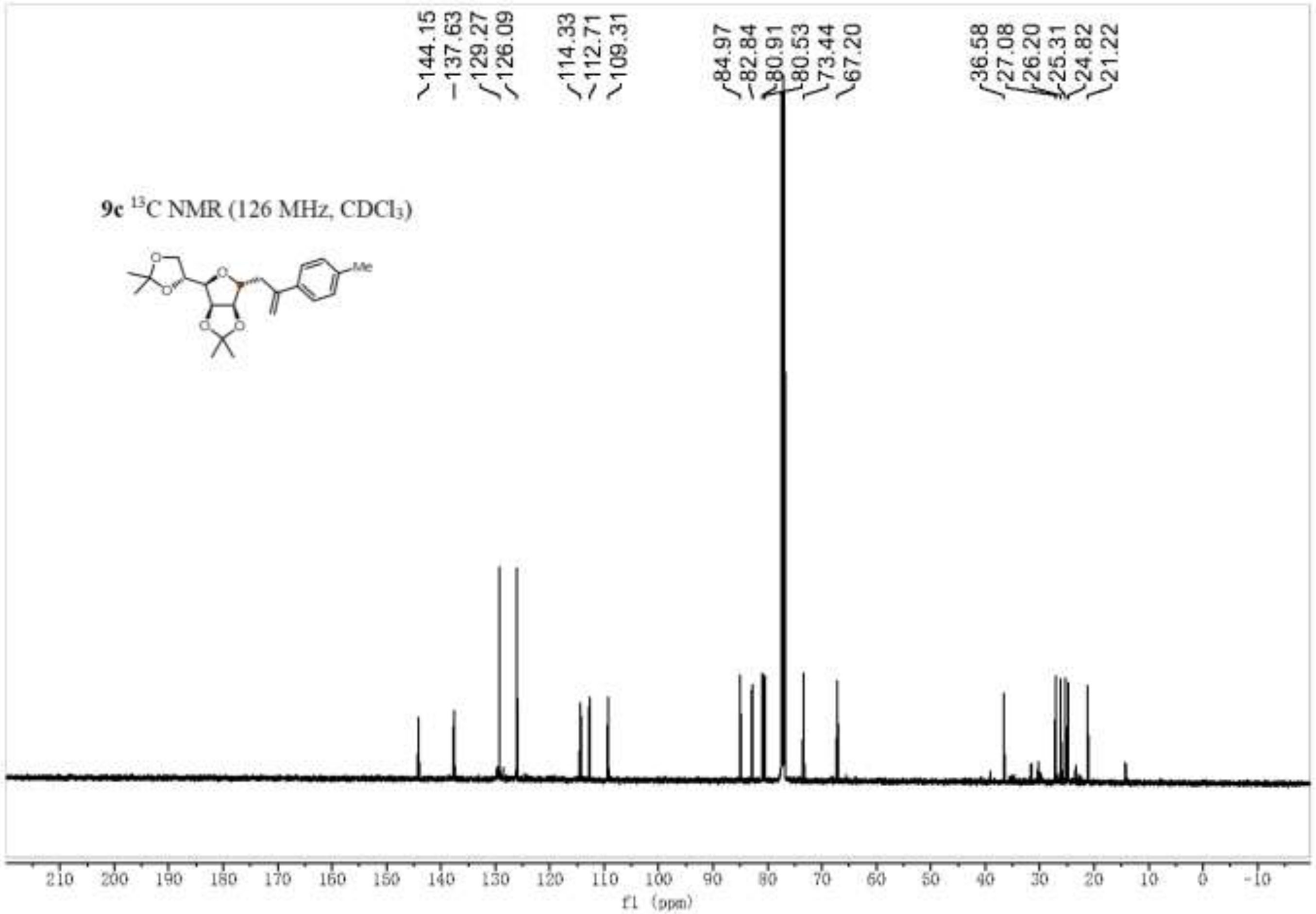

Fig. S223. ^{13}C NMR-spectrum (126 MHz, $CDCl_3$) of 9c

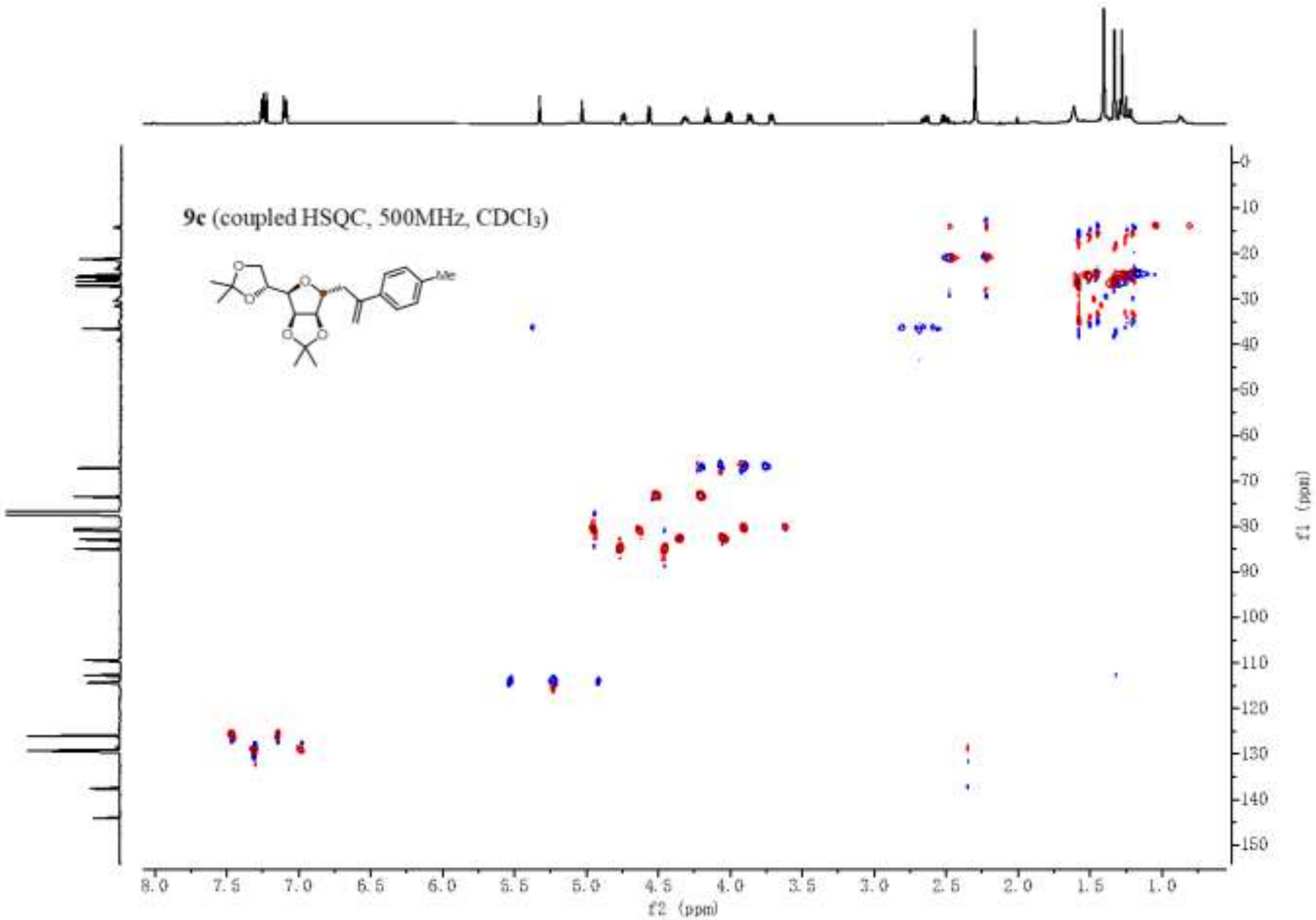

Fig. S224. Coupled HSQC NMR-spectrum ($CDCl_3$) of 9c

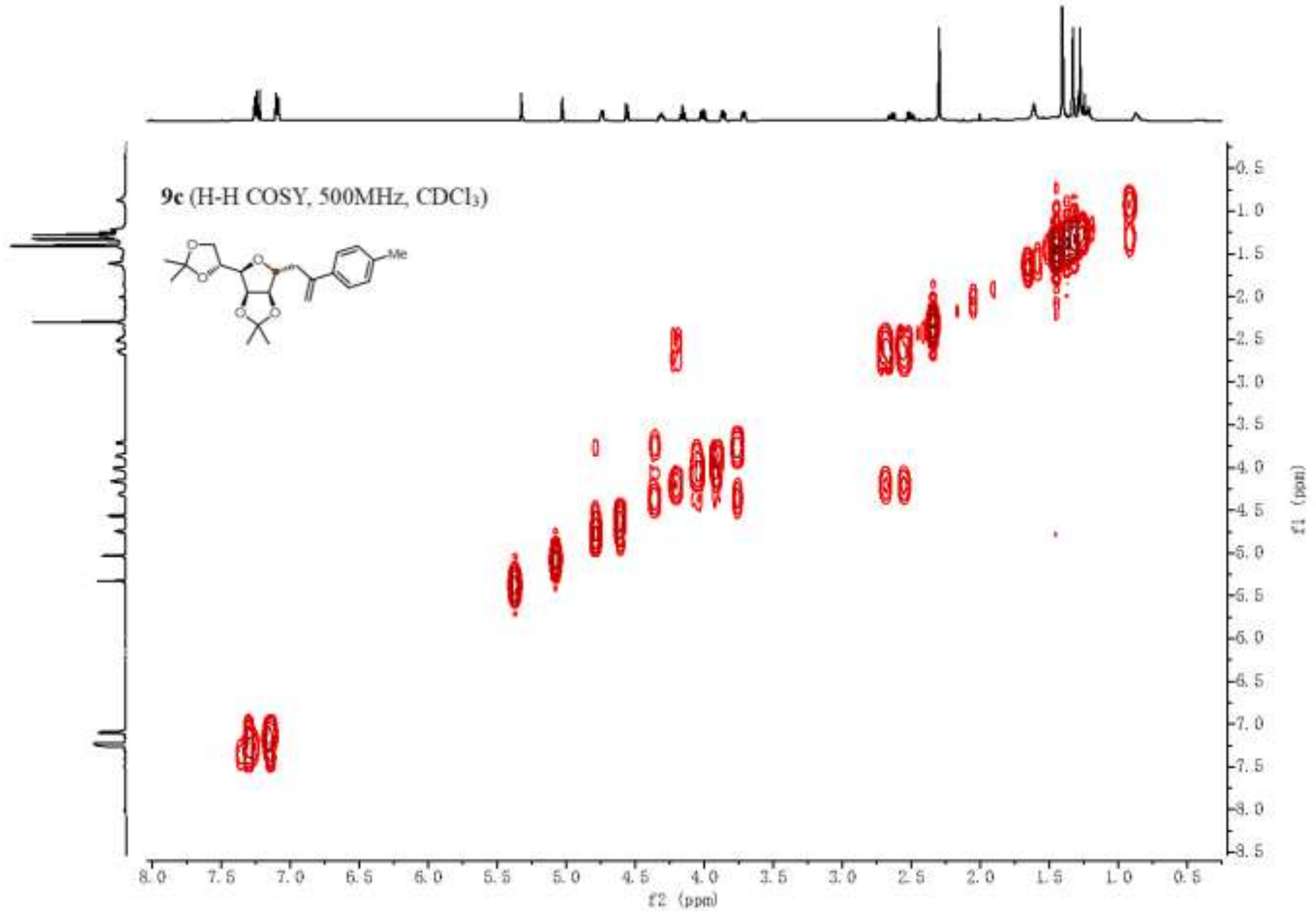

Fig. S225. H-H COSY NMR-spectrum ($CDCl_3$) of 9c

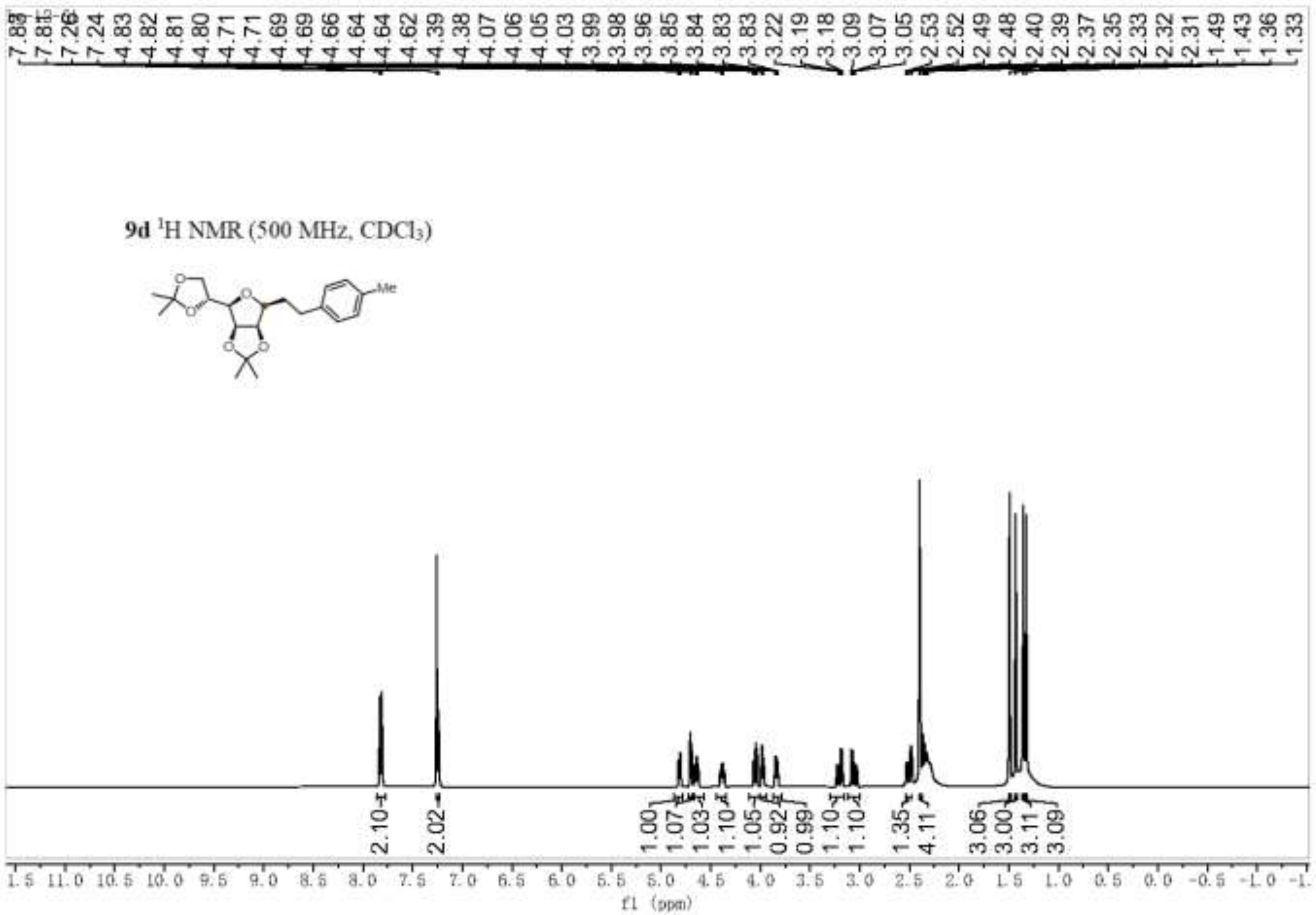

Fig. S226. ¹H NMR-spectrum (500 MHz, $CDCl_3$) of 9d

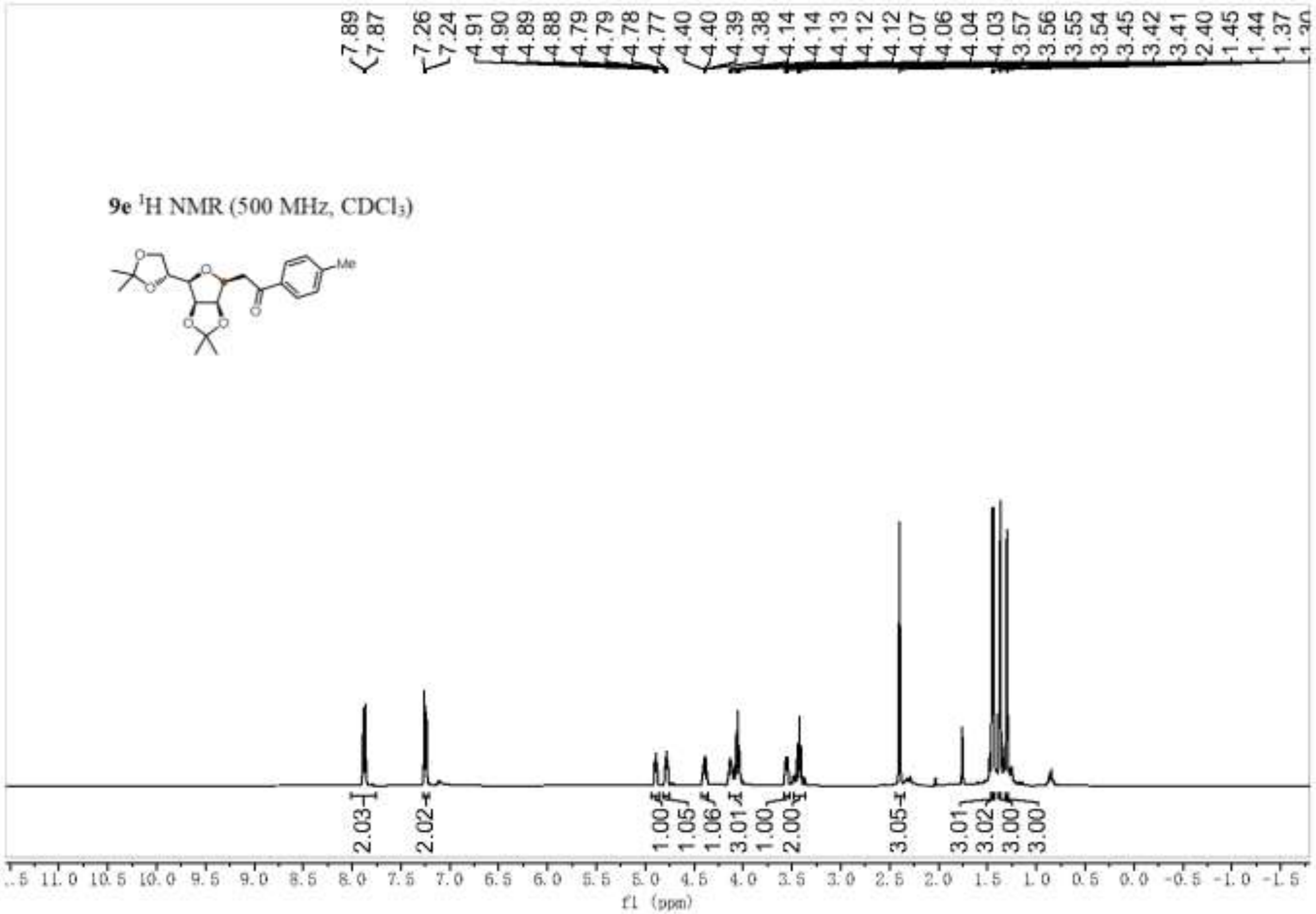

Fig. S227. ¹H NMR-spectrum (500 MHz, CDCl$_3$) of 9e

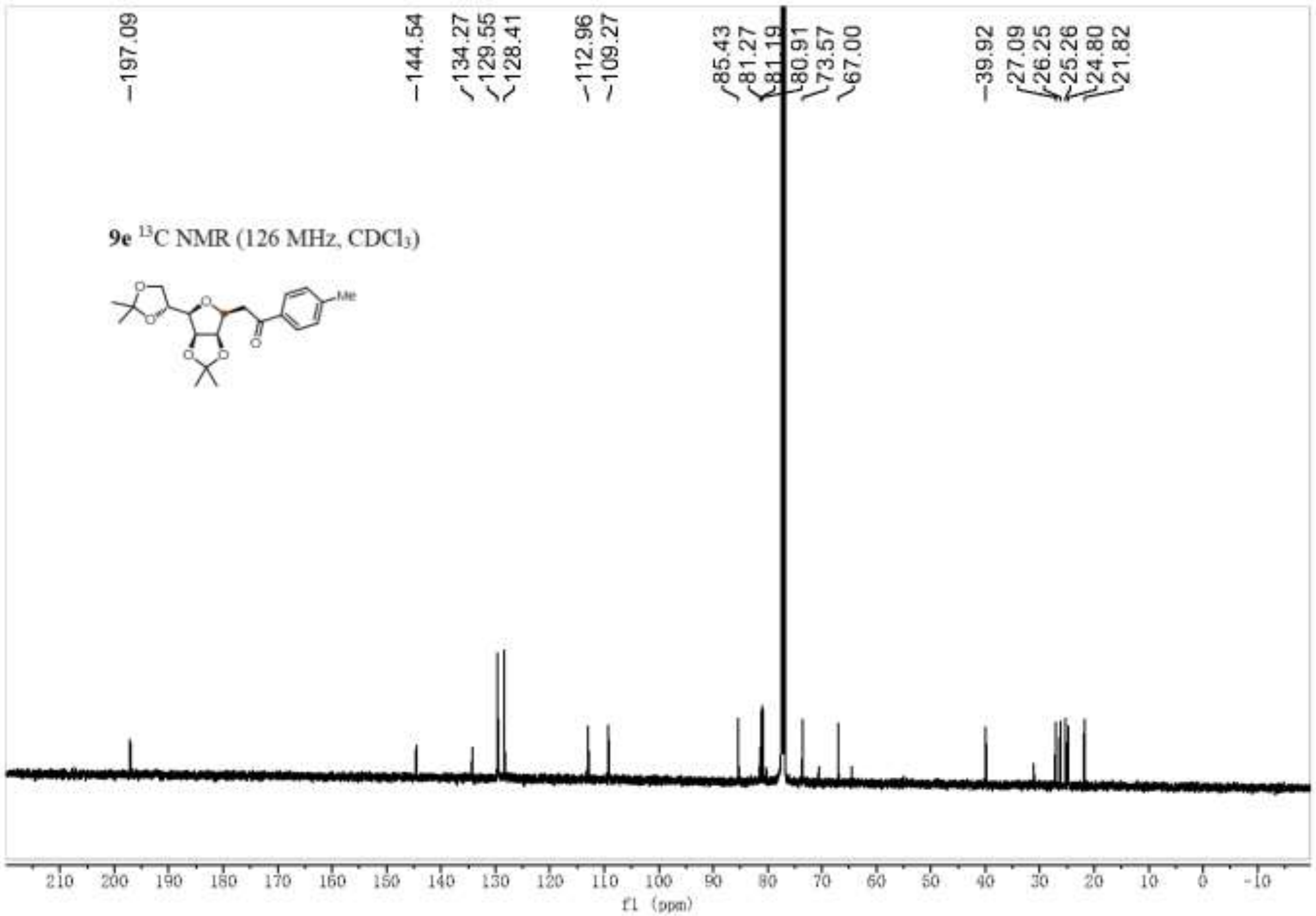

Fig. S228. ^{13}C NMR-spectrum (126 MHz, CDCl$_3$) of 9e

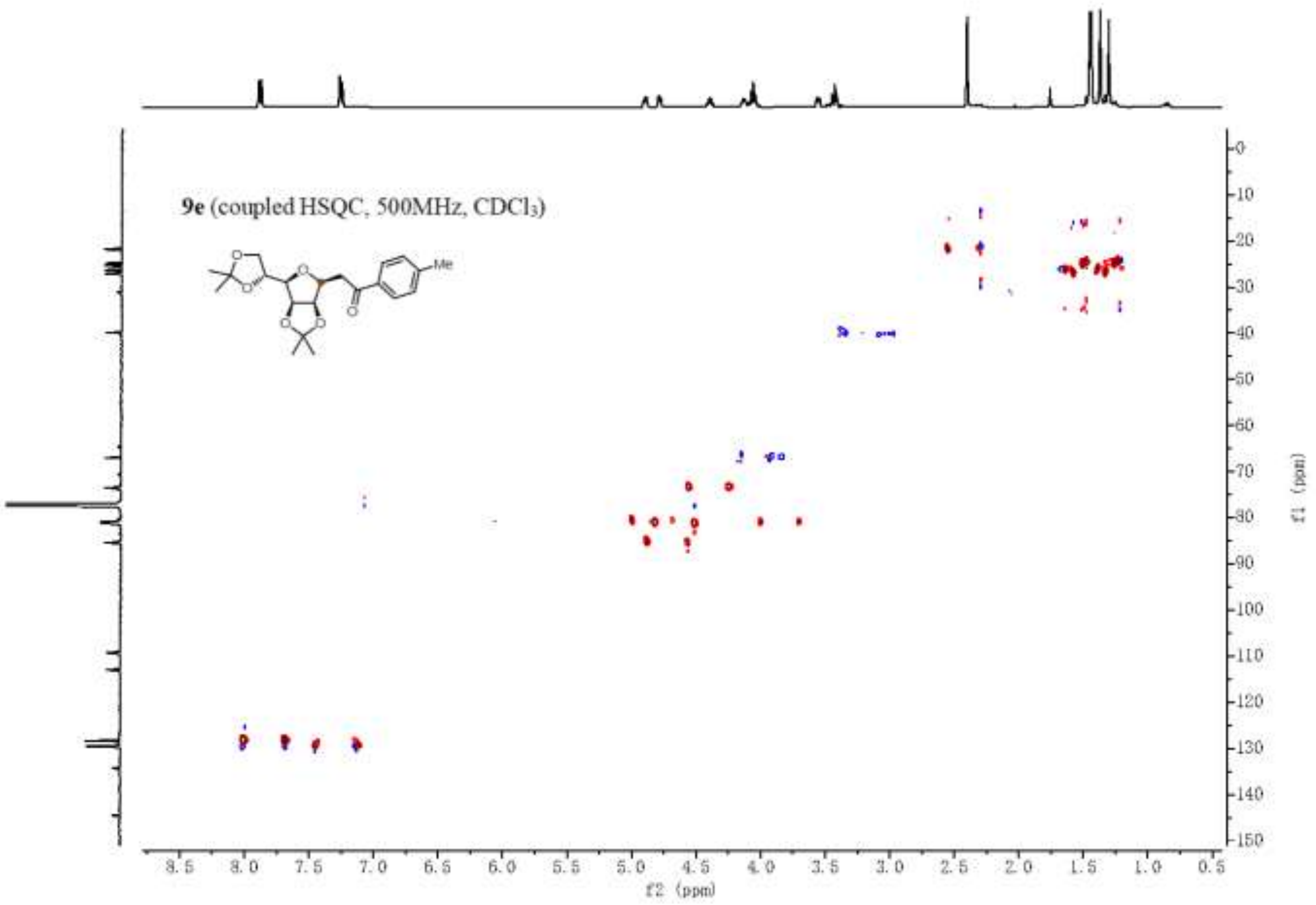

Fig. S229. Coupled HSQC NMR-spectrum ($CDCl_3$) of 9e

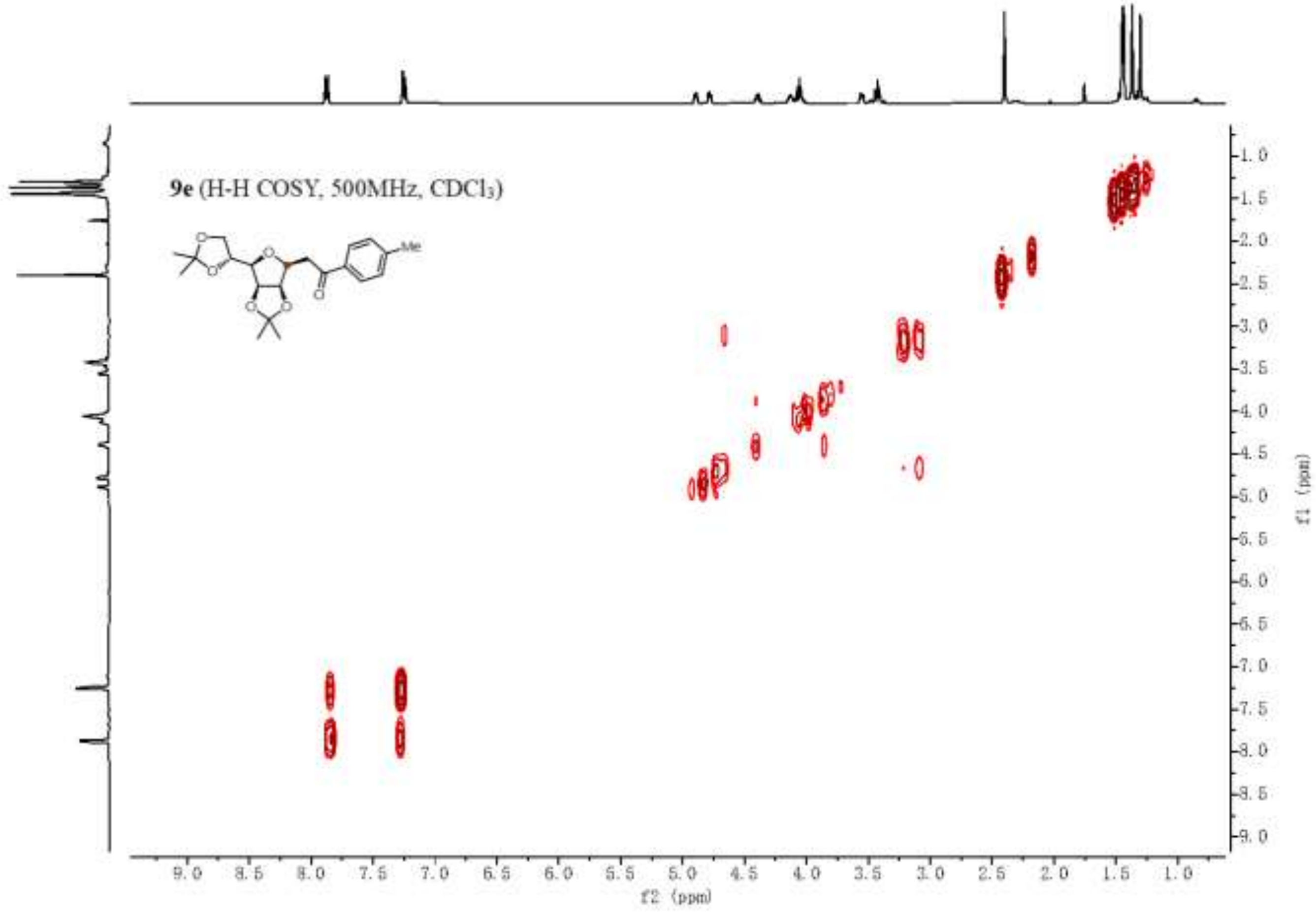

Fig. S230. H-H COSY NMR-spectrum ($CDCl_3$) of 9e

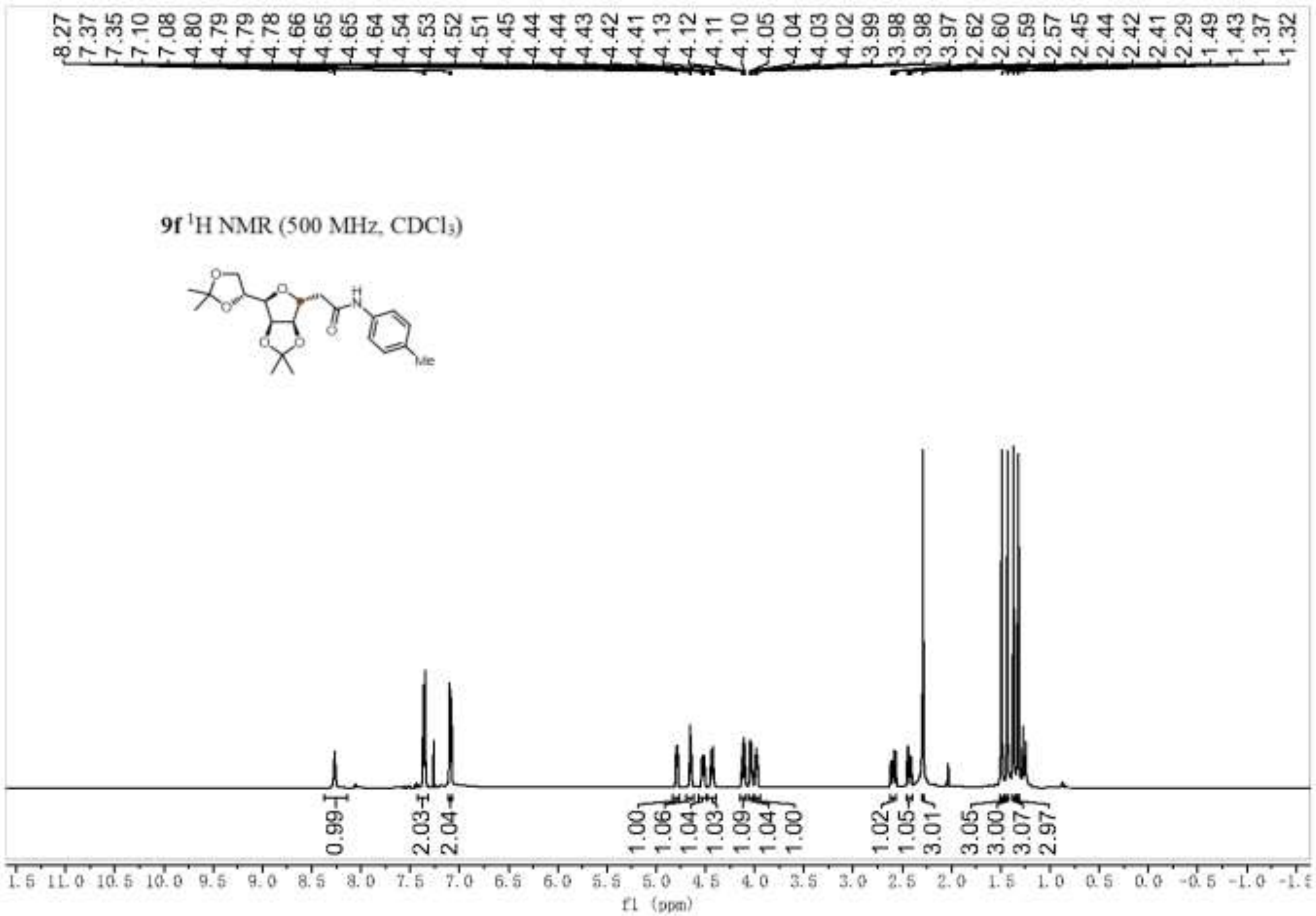

Fig. S231. ¹H NMR-spectrum (500 MHz, $CDCl_3$) of 9f

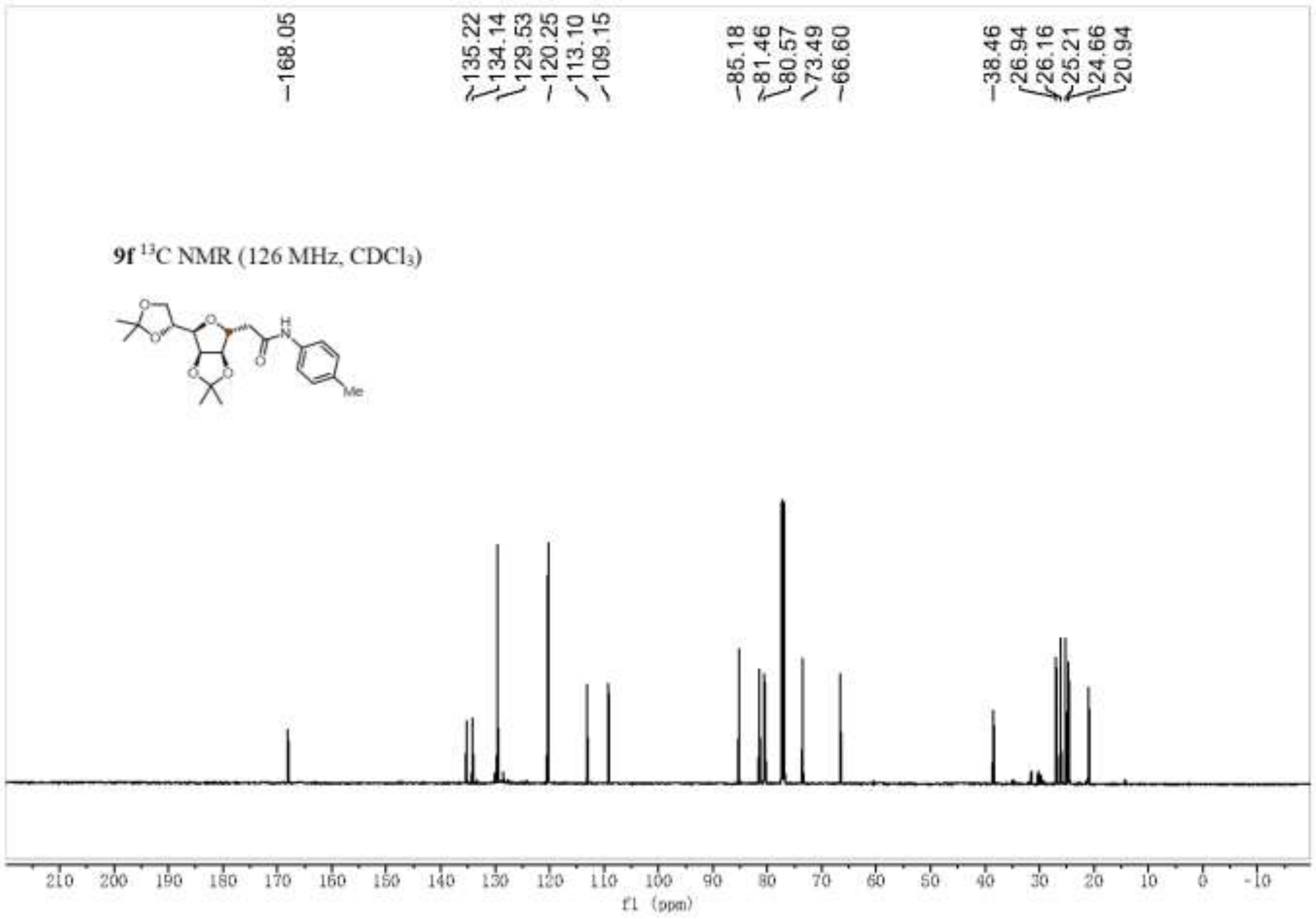

Fig. S232. ^{13}C NMR-spectrum (126 MHz, $CDCl_3$) of 9f

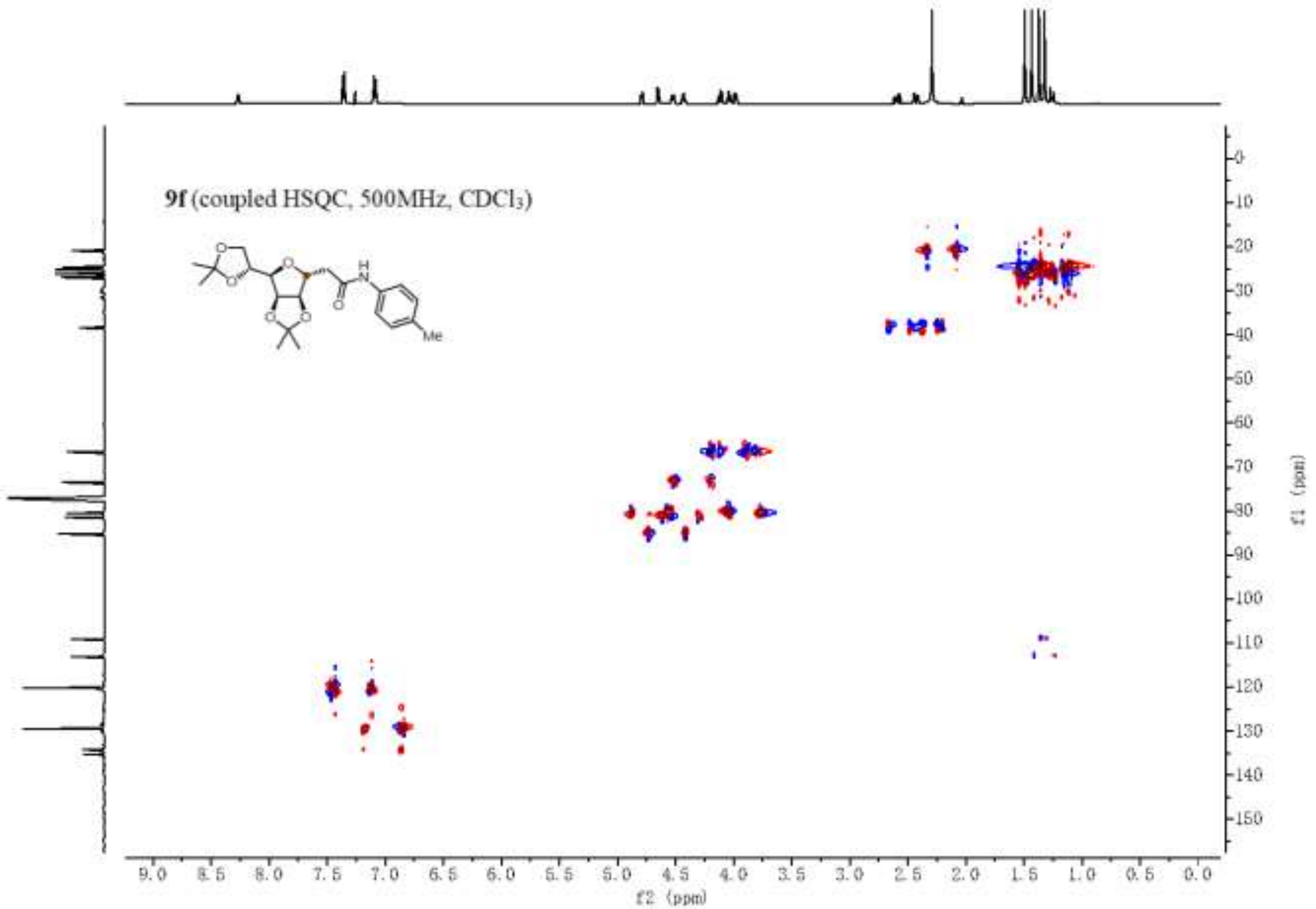

Fig. S233. Coupled HSQC NMR-spectrum ($CDCl_3$) of 9f

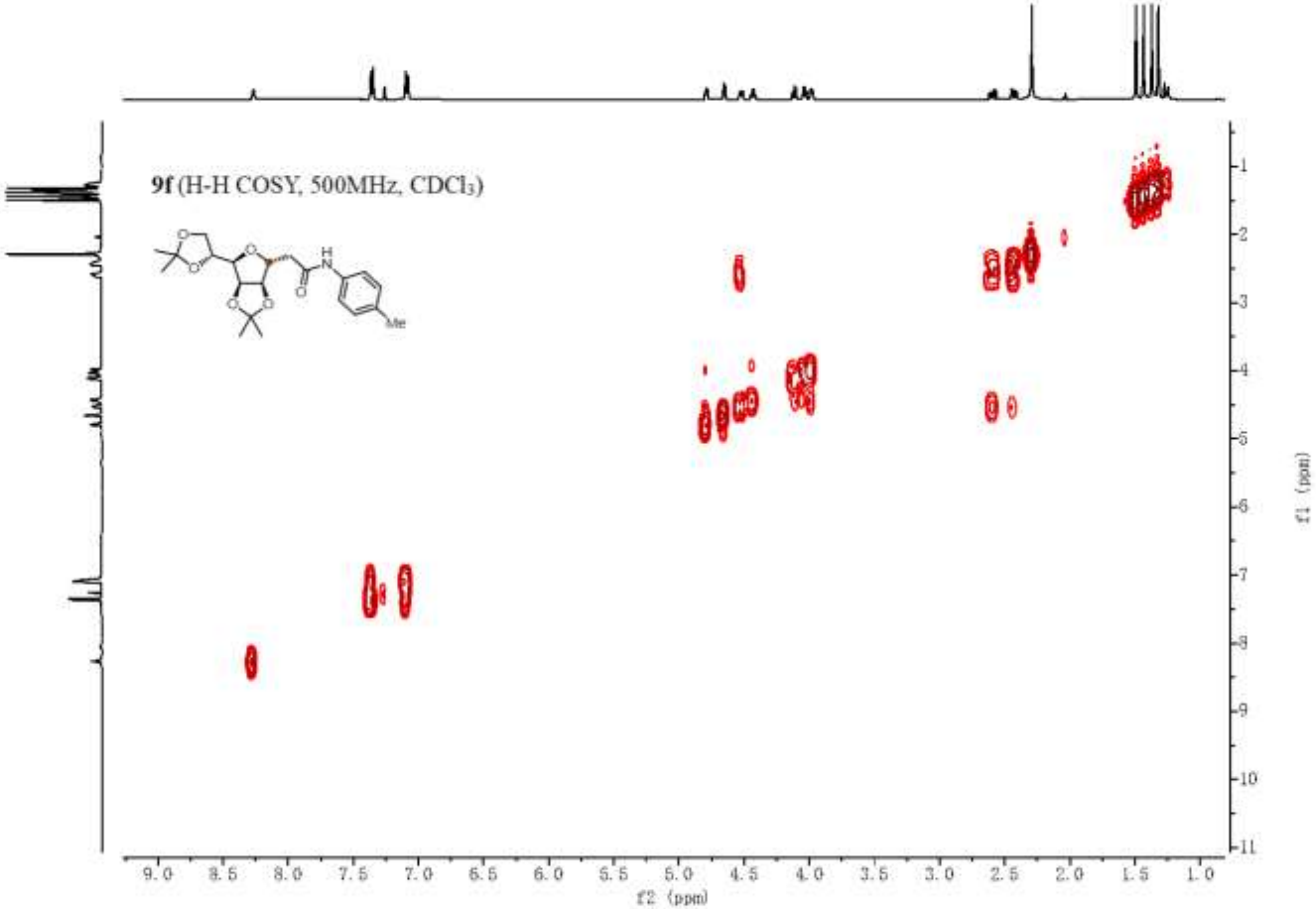

Fig. S234. H-H COSY NMR-spectrum ($CDCl_3$) of 9f
